# ESICM LIVES 2016: part one: Milan, Italy. 1-5 October 2016

**DOI:** 10.1186/s40635-016-0098-x

**Published:** 2016-09-29

**Authors:** L. Bos, L. Schouten, L. van Vught, M. Wiewel, D. Ong, O. Cremer, A. Artigas, I. Martin-Loeches, A. Hoogendijk, T. van der Poll, J. Horn, N. Juffermans, M. Schultz, N. de Prost, T. Pham, G. Carteaux, A. Mekontso Dessap, C. Brun-Buisson, E. Fan, G. Bellani, J. Laffey, A. Mercat, L. Brochard, B. Maitre, LUNG SAFE investigators and the ESICM study group, P. A. Howells, D. R. Thickett, C. Knox, D. P. Park, F. Gao, O. Tucker, T. Whitehouse, D. F. McAuley, G. D. Perkins, T. Pham, J. Laffey, G. Bellani, E. Fan, LUNG SAFE Investigators and the ESICM Trials Group, L. Pisani, J. P. Roozeman, F. D. Simonis, A. Giangregorio, L. R. Schouten, S. M. Van der Hoeven, J. Horn, A. Serpa Neto, E. Festic, A. M. Dondorp, S. Grasso, L. D. Bos, M. J. Schultz, M. Koster-Brouwer, D. Verboom, B. Scicluna, K. van de Groep, J. Frencken, M. Schultz, T. van der Poll, M. Bonten, O. Cremer, J. I. Ko, K. S. Kim, G. J. Suh, W. Y. Kwon, K. Kim, J. H. Shin, O. T. Ranzani, E. Prina, R. Menendez, A. Ceccato, R. Mendez, C. Cilloniz, A. Gabarrus, M. Ferrer, A. Torres, A. Urbano, L. A. Zhang, D. Swigon, F. Pike, R. S. Parker, G. Clermont, C. Scheer, S. O. Kuhn, A. Modler, M. Vollmer, C. Fuchs, K. Hahnenkamp, S. Rehberg, M. Gründling, A. Taggu, N. Darang, N. Öveges, I. László, K. Tánczos, M. Németh, G. Lebák, B. Tudor, D. Érces, J. Kaszaki, W. Huber, D. Trásy, Z. Molnár, G. Ferrara, V. S. Kanoore Edul, H. S. Canales, E. Martins, C. Canullán, G. Murias, M. O. Pozo, J. F. Caminos Eguillor, M. G. Buscetti, C. Ince, A. Dubin, H. D. Aya, A. Rhodes, N. Fletcher, R. M. Grounds, M. Cecconi, M. Jacquet-Lagrèze, M. Riche, R. Schweizer, P. Portran, W. Fornier, M. Lilot, J. Neidecker, J. L. Fellahi, A. Escoresca-Ortega, A. Gutiérrez-Pizarraya, L. Charris-Castro, Y. Corcia-Palomo, E. Fernandez-Delgado, J. Garnacho-Montero, C. Roger, L. Muller, L. Elotmani, J. Lipman, J. Y. Lefrant, J. A. Roberts, R. Muñoz-Bermúdez, M. Samper, C. Climent, F. Vasco, V. Sara, S. Luque, N. Campillo, S. Grau Cerrato, J. R. Masclans, F. Alvarez-Lerma, S. Carvalho Brugger, G. Jimenez Jimenez, M. Miralbés Torner, J. Trujillano Cabello, B. Balsera Garrido, X. Nuvials Casals, F. Barcenilla Gaite, M. Vallverdú Vidal, M. Palomar Martínez, V. Gusarov, D. Shilkin, M. Dementienko, E. Nesterova, N. Lashenkova, A. Kuzovlev, M. Zamyatin, A. Demoule, S. Carreira, S. Lavault, O. Palancca, E. Morawiec, J. Mayaux, I. Arnulf, T. Similowski, B. S. Rasmussen, R. G. Maltesen, M. Hanifa, S. Pedersen, S. R. Kristensen, R. Wimmer, M. Panigada, G. Li Bassi, O. T. Ranzani, T. Kolobow, A. Zanella, M. Cressoni, L. Berra, V. Parrini, H. Kandil, G. Salati, S. Livigni, A. Amatu, A. Andreotti, F. Tagliaferri, G. Moise, G. Mercurio, A. Costa, A. Vezzani, S. Lindau, J. Babel, M. Cavana, D. Consonni, A. Pesenti, L. Gattinoni, A. Torres, for the GRAVITY-VAP TRIAL NETWORK, P. Mansouri, F. Zand, L. Zahed, F. Dehghanrad, M. Bahrani, M. Ghorbani, B. Cambiaghi, O. Moerer, T. Mauri, N. Kunze-Szikszay, C. Ritter, A. Pesenti, M. Quintel, L. M. Vilander, M. A. Kaunisto, S. T. Vaara, V. Pettilä, FINNAKI Study Group, J. L. G. Haitsma Mulier, S. Rozemeijer, A. M. E. Spoelstra-de Man, P. E. Elbers, P. R. Tuinman, M. C. de Waard, H. M. Oudemans-van Straaten, A. M. A. Liberatore, R. B. Souza, A. M. C. R. P. F. Martins, J. C. F. Vieira, I. H. J. Koh, M. Galindo Martínez, R. Jiménez Sánchez, L. Martínez Gascón, M. D. Rodríguez Mulero, A. Ortín Freire, A. Ojados Muñoz, S. Rebollo Acebes, Á. Fernández Martínez, S. Moreno Aliaga, L. Herrera Para, J. Murcia Payá, F. Rodríguez Mulero, P. Guerci, Y. Ince, P. Heeman, B. Ergin, C. Ince, Z. Uz, M. Massey, Y. Ince, R. Papatella, E. Bulent, P. Guerci, F. Toraman, C. Ince, E. R. Longbottom, H. D. Torrance, H. C. Owen, C. J. Hinds, R. M. Pearse, M. J. O’Dywer, Z. Trogrlic, M. van der Jagt, H. Lingsma, H. H. Ponssen, J. F. Schoonderbeek, F. Schreiner, S. J. Verbrugge, S. Duran, T. van Achterberg, J. Bakker, D. A. M. P. J. Gommers, E. Ista, A. Krajčová, P. Waldauf, F. Duška, A. Shah, N. Roy, S. McKechnie, C. Doree, S. Fisher, S. J. Stanworth, J. F. Jensen, D. Overgaard, M. H. Bestle, D. F. Christensen, I. Egerod, The RAPIT Group, A. Pivkina, V. Gusarov, I. Zhivotneva, N. Pasko, M. Zamyatin, J. F. Jensen, I. Egerod, M. H. Bestle, D. F. Christensen, A. Alklit, R. L. Hansen, H. Knudsen, L. B. Grode, D. Overgaard, The RAPIT group, M. Hravnak, L. Chen, A. Dubrawski, G. Clermont, M. R. Pinsky, S. M. Parry, L. D. Knight, B. C. Connolly, C. E. Baldwin, Z. A. Puthucheary, L. Denehy, N. Hart, P. E. Morris, J. Mortimore, C. L. Granger, H. I. Jensen, R. Piers, B. Van den Bulcke, J. Malmgren, V. Metaxa, A. K. Reyners, M. Darmon, K. Rusinova, D. Talmor, A. P. Meert, L. Cancelliere, L. Zubek, P. Maia, A. Michalsen, J. Decruyenaere, E. Kompanje, S. Vanheule, E. Azoulay, S. Vansteelandt, D. Benoit, B. Van den Bulcke, R. Piers, H. I. Jensen, J. Malmgren, V. Metaxa, A. K. Reyners, M. Darmon, K. Rusinova, D. Talmor, A. P. Meert, L. Cancelliere, L. Zubek, P. Maia, A. Michalsen, J. Decruyenaere, E. Kompanje, S. Vanheule, E. Azoulay, S. Vansteelandt, D. Benoit, C. Ryan, D. Dawson, J. Ball, K. Noone, B. Aisling, S. Prudden, A. Ntantana, D. Matamis, S. Savvidou, M. Giannakou, M. Gouva, G. Nakos, V. Koulouras, J. Aron, G. Lumley, D. Milliken, K. Dhadwal, B. A. McGrath, S. J. Lynch, B. Bovento, G. Sharpe, E. Grainger, S. Pieri-Davies, S. Wallace, B. McGrath, S. J. Lynch, B. Bovento, E. Grainger, S. Pieri-Davies, G. Sharpe, S. Wallace, M. Jung, J. Cho, H. Park, G. Suh, O. Kousha, J. Paddle, L. Gamrin Gripenberg, M. Sundström Rehal, J. Wernerman, O. Rooyackers, H. J. de Grooth, W. P. Choo, A. M. Spoelstra-de Man, E. L. Swart, H. M. Oudemans-van Straaten, L. Talan, G. Güven, N. D. Altıntas, M. Padar, G. Uusvel, L. Starkopf, J. Starkopf, A. Reintam Blaser, M. S. Kalaiselvan, A. S. Arunkumar, M. K. Renuka, R. L. Shivkumar, M. Volbeda, D. ten Kate, M. Hoekstra, J. M. van der Maaten, M. W. Nijsten, A. Komaromi, O. Rooyackers, J. Wernerman, Å. Norberg, M. Smedberg, M. Mori, L. Pettersson, Å. Norberg, O. Rooyackers, J. Wernerman, M. Theodorakopoulou, T. Christodoulopoulou, A. Diamantakis, F. Frantzeskaki, M. Kontogiorgi, E. Chrysanthopoulou, M. Lygnos, C. Diakaki, A. Armaganidis, K. Gundogan, E. Dogan, R. Coskun, S. Muhtaroglu, M. Sungur, T. Ziegler, M. Guven, A. Kleyman, W. Khaliq, D. Andreas, M. Singer, R. Meierhans, R. Schuepbach, I. De Brito-Ashurst, F. Zand, G. Sabetian, R. Nikandish, F. Hagar, M. Masjedi, B. Maghsudi, A. Vazin, M. Ghorbani, E. Asadpour, K. C. Kao, L. C. Chiu, C. Y. Hung, C. H. Chang, S. H. Li, H. C. Hu, S. El Maraghi, M. Ali, D. Rageb, M. Helmy, J. Marin-Corral, C. Vilà, J. R. Masclans, A. Vàzquez, I. Martín-Loeches, E. Díaz, J. C. Yébenes, A. Rodriguez, F. Álvarez-Lerma, H1N1 SEMICYUC/GETGAG Working Group, N. Varga, A. Cortina-Gutiérrez, L. Dono, M. Martínez-Martínez, C. Maldonado, E. Papiol, M. Pérez-Carrasco, R. Ferrer, K. Nweze, B. Morton, I. Welters, M. Houard, B. Voisin, G. Ledoux, S. Six, E. Jaillette, S. Nseir, S. Romdhani, R. Bouneb, D. Loghmari, N. Ben Aicha, J. Ayachi, K. Meddeb, I. Chouchène, A. Khedher, M. Boussarsar, K. S. Chan, W. L. Yu, J. Marin-Corral, C. Vilà, J. R. Masclans, J. Nolla, L. Vidaur, J. Bonastre, B. Suberbiola, J. E. Guerrero, A. Rodriguez, H1N1 SEMICYUC/GETGAG working group, N. Ramon Coll, G. Jiménez Jiménez, S. Carvalho Brugger, J. Codina Calero, B. Balsera Garrido, M. García, M. Palomar Martínez, M. Vallverdú Vidal, M. C. de la Torre, E. Vendrell, E. Palomera, E. Güell, J. C. Yébenes, M. Serra-Prat, J. F. Bermejo-Martín, J. Almirall, E. Tomas, A. Escoval, F. Froe, M. H. Vitoria Pereira, N. Velez, E. Viegas, E. Filipe, C. Groves, M. Reay, L. C. Chiu, H. C. Hu, C. Y. Hung, C. H. Chang, S. H. Li, K. C. Kao, A. Ballin, F. Facchin, G. Sartori, F. Zarantonello, E. Campello, C. M. Radu, S. Rossi, C. Ori, P. Simioni, N. Umei, I. Shingo, A. C. Santos, C. Candeias, I. Moniz, R. Marçal, Z. Costa e Silva, J. M. Ribeiro, J. F. Georger, J. P. Ponthus, M. Tchir, V. Amilien, M. Ayoub, E. Barsam, G. Martucci, G. Panarello, F. Tuzzolino, G. Capitanio, V. Ferrazza, T. Carollo, L. Giovanni, A. Arcadipane, M. López Sánchez, M. A. González-Gay, F. J. Llorca Díaz, M. I. Rubio López, E. Zogheib, L. Villeret, J. Nader, M. Bernasinski, P. Besserve, T. Caus, H. Dupont, P. Morimont, S. Habran, R. Hubert, T. Desaive, F. Blaffart, N. Janssen, J. Guiot, A. Pironet, P. Dauby, B. Lambermont, F. Zarantonello, A. Ballin, F. Facchin, G. Sartori, E. Campello, T. Pettenuzzo, G. Citton, S. Rossi, P. Simioni, C. Ori, C. Kirakli, O. Ediboglu, S. Ataman, M. Yarici, F. Tuksavul, S. Keating, A. Gibson, M. Gilles, M. Dunn, G. Price, N. Young, P. Remeta, P. Bishop, M. D. Fernández Zamora, J. Muñoz-Bono, E. Curiel-Balsera, E. Aguilar-Alonso, R. Hinojosa, A. Gordillo-Brenes, J. A. Arboleda-Sánchez, ARIAM-CARDIAC SURGERY PROJECT AUTHORS, I. Skorniakov, D. Vikulova, C. Whiteley, O. Shaikh, A. Jones, M. Ostermann, L. Forni, M. Scott, J. Sahatjian, W. Linde-Zwirble, D. Hansell, P. Laoveeravat, N. Srisawat, M. Kongwibulwut, S. Peerapornrattana, N. Suwachittanont, T. O. Wirotwan, P. Chatkaew, P. Saeyub, K. Latthaprecha, K. Tiranathanagul, S. Eiam-ong, J. A. Kellum, R. E. Berthelsen, A. Perner, A. E. K. Jensen, J. U. Jensen, M. H. Bestle, D. J. Gebhard, J. Price, C. E. Kennedy, A. Akcan-Arikan, A. M. A. Liberatore, R. B. Souza, A. M. C. R. P. F. Martins, J. C. F. Vieira, Y. R. Kang, M. N. Nakamae, I. H. J. Koh, K. Hamed, M. M. Khaled, R. Aly Soliman, M. Sherif Mokhtar, G. Seller-Pérez, D. Arias-Verdú, E. Llopar-Valdor, I. De-Diós-Chacón, G. Quesada-García, M. E. Herrera-Gutierrez, R. Hafes, G. Carroll, P. Doherty, C. Wright, I. G. Guerra Vera, M. Ralston, M. L. Gemmell, A. MacKay, E. Black, C. Wright, R. I. Docking, R. Appleton, M. R. Ralston, L. Gemmell, R. Appleton, C. Wright, R. I. Docking, E. Black, A. Mackay, S. Rozemeijer, J. L. G. Haitsma Mulier, J. G. Röttgering, P. W. G. Elbers, A. M. E. Spoelstra-de Man, P. R. Tuinman, M. C. de Waard, H. M. Oudemans-van Straaten, N. Mejeni, J. Nsiala, A. Kilembe, P. Akilimali, G. Thomas, I. Egerod, A. E. Andersson, A. M. Fagerdahl, V. Knudsen, P-INFECT, K. Meddeb, A. Ben Cheikh, Y. Hamdaoui, J. Ayachi, A. Guiga, N. Fraj, S. Romdhani, N. Sma, R. Bouneb, I. Chouchene, A. Khedher, N. Bouafia, M. Boussarsar, A. Amirian, B. Ziaian, M. Masjedi, C. Fleischmann, D. O. Thomas-Rueddel, A. Schettler, D. Schwarzkopf, A. Stacke, K. Reinhart, E. Filipe, A. Escoval, A. Martins, P. Sousa, N. Velez, E. Viegas, E. Tomas, G. Snell, R. Matsa, T. T. S. Paary, M. S. Kalaiselvan, A. M. Cavalheiro, L. L. Rocha, C. S. Vallone, A. Tonilo, M. D. S. Lobato, D. T. Malheiro, G. Sussumo, N. M. Lucino, F. Zand, V. D. Rosenthal, M. Masjedi, G. Sabetian, B. Maghsudi, M. Ghorbani, A. Sanaei Dashti, A. Yousefipour, J. R. Goodall, M. Williamson, E. Tant, N. Thomas, C. Balci, C. Gonen, E. Haftacı, H. Gurarda, E. Karaca, B. Paldusová, I. Zýková, D. Šímová, S. Houston, L. D’Antona, J. Lloyd, V. Garnelo-Rey, M. Sosic, V. Sotosek-Tokmazic, J. Kuharic, I. Antoncic, S. Dunatov, A. Sustic, C. T. Chong, M. Sim, T. Lyovarin, F. M. Acosta Díaz, S. Narbona Galdó, M. Muñoz Garach, O. Moreno Romero, A. M. Pérez Bailón, A. Carranza Pinel, M. Colmenero, A. Gritsan, A. Gazenkampf, E. Korchagin, N. Dovbish, R. M. Lee, M. P. P. Lim, C. T. Chong, B. C. L. Lim, J. J. See, R. Assis, F. Filipe, N. Lopes, L. Pessoa, T. Pereira, N. Catorze, M. S. Aydogan, C. Aldasoro, P. Marchio, A. Jorda, M. D. Mauricio, S. Guerra-Ojeda, M. Gimeno-Raga, M. Colque-Cano, A. Bertomeu-Artecero, M. Aldasoro, S. L. Valles, D. Tonon, T. Triglia, J. C. Martin, M. C. Alessi, N. Bruder, P. Garrigue, L. Velly, S. Spina, V. Scaravilli, C. Marzorati, E. Colombo, D. Savo, A. Vargiolu, G. Cavenaghi, G. Citerio, A. H. V. Andrade, P. Bulgarelli, J. A. P. Araujo, V. Gonzalez, V. A. Souza, A. Costa, C. Massant, C. A. C. Abreu Filho, R. A. Morbeck, L. E. Burgo, R. van Groenendael, L. T. van Eijk, G. P. Leijte, B. Koeneman, M. Kox, P. Pickkers, A. García-de la Torre, M. de la Torre-Prados, A. Fernández-Porcel, C. Rueda-Molina, P. Nuevo-Ortega, T. Tsvetanova-Spasova, E. Cámara-Sola, A. García-Alcántara, L. Salido-Díaz, X. Liao, T. Feng, J. Zhang, X. Cao, Q. Wu, Z. Xie, H. Li, Y. Kang, M. S. Winkler, A. Nierhaus, E. Mudersbach, A. Bauer, L. Robbe, C. Zahrte, E. Schwedhelm, S. Kluge, C. Zöllner, B. Morton, E. Mitsi, S. H. Pennington, J. Reine, A. D. Wright, R. Parker, I. D. Welters, J. D. Blakey, G. Rajam, E. W. Ades, D. M. Ferreira, D. Wang, A. Kadioglu, S. B. Gordon, R. Koch, M. Kox, J. Rahamat-Langedoen, J. Schloesser, M. de Jonge, P. Pickkers, J. Bringue, R. Guillamat-Prats, E. Torrents, M. L. Martinez, M. Camprubí-Rimblas, A. Artigas, L. Blanch, S. Y. Park, Y. B. Park, D. K. Song, S. Shrestha, S. H. Park, Y. Koh, M. J. Park, C. W. Hong, O. Lesur, D. Coquerel, X. Sainsily, J. Cote, T. Söllradl, A. Murza, L. Dumont, R. Dumaine, M. Grandbois, P. Sarret, E. Marsault, D. Salvail, M. Auger-Messier, F. Chagnon, Apelin Group, M. P. Lauretta, E. Greco, A. Dyson, M. Singer, S. Preau, M. Ambler, A. Sigurta, S. Saeed, M. Singer, L. Topcu Sarıca, N. Zibandeh, D. Genc, F. Gul, T. Akkoc, E. Kombak, L. Cinel, T. Akkoc, I. Cinel, S. J. Pollen, N. Arulkumaran, M. Singer, H. D. Torrance, E. R. Longbottom, G. Warnes, C. J. Hinds, D. J. Pennington, K. Brohi, M. J. O’Dwyer, H. Y. Kim, S. Na, J. Kim, Y. F. Chang, A. Chao, P. Y. Shih, C. T. Lee, Y. C. Yeh, L. W. Chen, M. Adriaanse, Z. Trogrlic, E. Ista, H. Lingsma, W. Rietdijk, H. H. Ponssen, J. F. Schoonderbeek, F. Schreiner, S. J. Verbrugge, S. Duran, D. A. M. P. J. Gommers, M. van der Jagt, S. Funcke, S. Sauerlaender, B. Saugel, H. Pinnschmidt, D. A. Reuter, R. Nitzschke, S. Perbet, C. Biboulet, A. Lenoire, D. Bourdeaux, B. Pereira, B. Plaud, J. E. Bazin, V. Sautou, A. Mebazaa, J. M. Constantin, M. Legrand, Y. Boyko, P. Jennum, M. Nikolic, H. Oerding, R. Holst, P. Toft, H. K. Nedergaard, T. Haberlandt, H. I. Jensen, P. Toft, S. Park, S. Kim, Y. J. Cho, Y. J. Lim, A. Chan, S. Tang, S. L. Nunes, S. Forsberg, H. Blomqvist, L. Berggren, M. Sörberg, T. Sarapohja, C. J. Wickerts, J. G. M. Hofhuis, L. Rose, B. Blackwood, E. Akerman, J. Mcgaughey, I. Egerod, M. Fossum, H. Foss, E. Georgiou, H. J. Graff, M. Kalafati, R. Sperlinga, A. Schafer, A. G. Wojnicka, P. E. Spronk, F. Zand, F. Khalili, R. Afshari, G. Sabetian, M. Masjedi, B. Maghsudi, H. Haddad Khodaei, S. Javadpour, P. Petramfar, S. Nasimi, A. Vazin, B. Ziaian, H. Tabei, A. Gunther, J. O. Hansen, P. Sackey, H. Storm, J. Bernhardsson, Ø. Sundin, A. Bjärtå, A. Bienert, P. Smuszkiewicz, P. Wiczling, K. Przybylowski, A. Borsuk, I. Trojanowska, J. Matysiak, Z. Kokot, M. Paterska, E. Grzeskowiak, A. Messina, E. Bonicolini, D. Colombo, G. Moro, S. Romagnoli, A. R. De Gaudio, F. Della Corte, S. M. Romano, J. A. Silversides, E. Major, E. E. Mann, A. J. Ferguson, D. F. Mcauley, J. C. Marshall, B. Blackwood, E. Fan, J. A. Diaz-Rodriguez, R. Silva-Medina, E. Gomez-Sandoval, N. Gomez-Gonzalez, R. Soriano-Orozco, P. L. Gonzalez-Carrillo, M. Hernández-Flores, K. Pilarczyk, J. Lubarksi, D. Wendt, F. Dusse, J. Günter, B. Huschens, E. Demircioglu, H. Jakob, A. Palmaccio, A. M. Dell’Anna, D. L. Grieco, F. Torrini, C. Iaquaniello, F. Bongiovanni, M. Antonelli, L. Toscani, D. Antonakaki, D. Bastoni, H. D. Aya, A. Rhodes, M. Cecconi, M. Jozwiak, F. Depret, J. L. Teboul, J. Alphonsine, C. Lai, C. Richard, X. Monnet, I. László, G. Demeter, N. Öveges, K. Tánczos, M. Németh, D. Trásy, I. Kertmegi, D. Érces, B. Tudor, J. Kaszaki, Z. Molnár, A. Hasanin, A. Lotfy, A. El-adawy, H. Nassar, S. Mahmoud, A. Abougabal, A. Mukhtar, F. Quinty, S. Habchi, A. Luzi, E. Antok, G. Hernandez, B. Lara, L. Enberg, M. Ortega, P. Leon, C. Kripper, P. Aguilera, E. Kattan, J. Bakker, W. Huber, M. Lehmann, S. Sakka, B. Bein, R. M. Schmid, J. Preti, J. Creteur, A. Herpain, J. Marc, E. Zogheib, F. Trojette, S. Bar, L. Kontar, D. Titeca, J. Richecoeur, B. Gelee, N. Verrier, R. Mercier, E. Lorne, J. Maizel, H. Dupont, M. Slama, M. E. Abdelfattah, A. Eladawy, M. A. Ali Elsayed, A. Mukhtar, A. Pedraza Montenegro, E. Monares Zepeda, J. Franco Granillo, J. S. Aguirre Sánchez, G. Camarena Alejo, A. Rugerio Cabrera, A. A. Tanaka Montoya, C. Lee, F. Hatib, M. Cannesson, P. Theerawit, T. Morasert, Y. Sutherasan, G. Zani, S. Mescolini, M. Diamanti, R. Righetti, A. Scaramuzza, M. Papetti, M. Terenzoni, C. Gecele, M. Fusari, K. A. Hakim, A. Chaari, M. Ismail, A. H. Elsaka, T. M. Mahmoud, K. Bousselmi, V. Kauts, W. F. Casey, S. D. Hutchings, D. Naumann, J. Wendon, S. Watts, E. Kirkman, Z. Jian, S. Buddi, C. Lee, J. Settels, F. Hatib, M. R. Pinsky, P. Bertini, F. Guarracino, C. Trepte, P. Richter, S. A. Haas, V. Eichhorn, J. C. Kubitz, D. A. Reuter, M. S. Soliman, W. I. Hamimy, A. Z. Fouad, A. M. Mukhtar, M. Charlton, L. Tonks, L. Mclelland, T. J. Coats, J. P. Thompson, M. R. Sims, D. Williams, D. Z. Roushdy, R. A. Soliman, R. A. Nahas, M. Y. Arafa, W. T. Hung, C. C. Chiang, W. C. Huang, K. C. Lin, S. C. Lin, C. C. Cheng, P. L. Kang, S. R. Wann, G. Y. Mar, C. P. Liu, M. Lopez Carranza, H. Sancho Fernandez, J. A. Sanchez Roman, F. Lucena, A. Campanario Garcia, A. Loza Vazquez, A. Lesmes Serrano, ARIAM-SEMICYUC Registry Investigators, L. Sayagues Moreira, R. Vidal-Perez, U. Anido Herranz, J. M. Garcia Acuna, C. Pena Gil, J. L. Garcia Allut, P. Rascado Sedes, C. Martin Lopez, E. Saborido Paz, C. Galban Rodriguez, J. R. Gonzalez-Juanatey, A. Vallejo-Baez, M. V. de la Torre-Prados, P. Nuevo-Ortega, A. Fernández-Porcel, E. Cámara-Sola, T. Tsvetanova-Spasova, C. Rueda-Molina, L. Salido-Díaz, A. García-Alcántara, ARIAM Group, J. Aron, R. Marharaj, K. Gervasio, M. Bottiroli, M. Mondino, D. De Caria, A. Calini, E. Montrasio, F. Milazzo, M. P. Gagliardone, A. Vallejo-Báez, M. V. de la Torre-Prados, P. Nuevo-Ortega, A. Fernández-Porcel, E. Cámara-Sola, T. Tsvetanova-Spasova, C. Rueda-Molina, L. Salido-Díaz, A. García-Alcántara, ARIAM group, L. Sayagues Moreira, R. Vidal-Perez, U. Anido, C. Pena Gil, J. M. Garcia Acuna, P. Rascado Sedes, C. Martin Lopez, E. Saborido Paz, J. L. Garcia Allut, C. Galban Rodriguez, J. R. Gonzalez-Juanatey, Y. Hamdaoui, A. Khedher, M. Cheikh-Bouhlel, J. Ayachi, K. Meddeb, N. Sma, N. Fraj, N. Ben Aicha, S. Romdhani, R. Bouneb, I. Chouchene, M. Boussarsar, M. P. R. D. L. Dela Cruz, J. M. Bernardo, F. Galfo, A. Dyson, M. Singer, A. Marino, A. Dyson, M. Singer, C. C. Chao, P. Hou, W. C. Huang, C. C. Hung, C. H. Chiang, W. T. Hung, K. C. Lin, S. C. Lin, Y. J. Liou, S. M. Hung, Y. S. Lin, C. C. Cheng, F. Y. Kuo, K. R. Chiou, C. J. Chen, L. S. Yan, C. Y. Liu, H. H. Wang, P. L. Kang, H. L. Chen, C. K. Ho, G. Y. Mar, C. P. Liu, S. Grewal, S. Gopal, C. Corbett, A. Wilson, J. Capps, W. Ayoub, A. Lomas, S. Ghani, J. Moore, D. Atkinson, M. Sharman, W. Swinnen, J. Pauwels, K. Mignolet, E. Pannier, A. Koch, T. Sarens, W. Temmerman, A. M. Elmenshawy, A. M. Fayed, M. Elboriuny, E. Hamdy, E. Zakaria, A. C. Falk, A. Petosic, K. Olafsen, H. Wøien, H. Flaatten, K. Sunde, J. J. Cáceres Agra, J. L. Santana Cabrera, J. D. Martín Santana, L. Melián Alzola, H. Rodríguez Pérez, T. Castro Pires, H. Calderón, A. Pereira, S. Castro, C. Granja, I. Norkiene, I. Urbanaviciute, G. Kezyte, D. Ringaitiene, T. Jovaisa, G. Vogel, U. B. Johansson, A. Sandgren, C. Svensen, E. Joelsson-Alm, M. A. Leite, L. D. Murbach, E. F. Osaku, C. R. L. M. Costa, M. Pelenz, N. M. Neitzke, M. M. Moraes, J. L. Jaskowiak, M. M. M. Silva, R. S. Zaponi, L. R. L. Abentroth, S. M. Ogasawara, A. C. Jorge, P. A. D. Duarte, L. D. Murbach, M. A. Leite, E. F. Osaku, J. Barreto, S. T. Duarte, S. Taba, D. Miglioranza, D. P. Gund, C. F. Lordani, C. R. L. M. Costa, S. M. Ogasawara, A. C. Jorge, P. A. D. Duarte, H. Vollmer, M. Gager, C. Waldmann, A. T. Mazzeo, R. Tesio, C. Filippini, M. E. Vallero, C. Giolitti, S. Caccia, M. Medugno, T. Tenaglia, R. Rosato, I. Mastromauro, L. Brazzi, P. P. Terragni, R. Urbino, V. Fanelli, V. M. Ranieri, L. Mascia, J. Ballantyne, L. Paton, A. Mackay, P. Perez-Teran, O. Roca, J. C. Ruiz-Rodriguez, A. Zapatero, J. Serra, J. R. Masclans, S. Bianzina, P. Cornara, G. Rodi, G. Tavazzi, M. Pozzi, G. A. Iotti, F. Mojoli, A. Braschi, A. Vishnu, D. Buche, R. Pande, D. L. J. Moolenaar, F. Bakhshi-Raiez, D. A. Dongelmans, N. F. de Keizer, D. W. de Lange, I. Fuentes Fernández, D. Martínez Baño, J. L. Buendía Moreno, R. Jara Rubio, J. Scott, D. Phelan, D. Morely, J. O’Flynn, P. Stapleton, M. Lynch, B. Marsh, E. Carton, C. O’Loughlin, K. C. Cheng, M. I. Sung, M. O. Elghonemi, M. H. Saleh, T. S. Meyhoff, M. Krag, P. B. Hjortrup, A. Perner, M. H. Møller, T. Öhman, T. Sigmundsson, E. Redondo, M. Hallbäck, F. Suarez-Sipmann, H. Björne, C. Hällsjö Sander, KARISMA, M. Cressoni, D. Chiumello, C. Chiurazzi, M. Brioni, I. Algieri, M. Guanziroli, G. Vergani, T. Tonetti, I. Tomic, A. Colombo, F. Crimella, E. Carlesso, A. Colombo, V. Gasparovic, L. Gattinoni, R. El-Sherif, M. Abd Al-Basser, A. Raafat, A. El-Sherif, F. D. Simonis, L. R. A. Schouten, O. L. Cremer, D. S. Y. Ong, G. Amoruso, G. Cinnella, M. J. Schultz, L. D. J. Bos, W. Huber, P. Schmidle, M. Findeisen, P. Hoppmann, J. Jaitner, F. Brettner, R. M. Schmid, T. Lahmer, EXODUS-investigators, E. Festic, G. Rajagopalan, V. Bansal, R. Frank, R. Hinds, J. Levitt, United States Critical Illness and Injury Trials Group/LIPS-B investigators, S. Siddiqui, SICM NICER Group, J. P. Gilbert, K. Sim, C. H. Wang, H. C. Hu, I. J. Li, W. R. Tang, K. C. Kao, P. Persona, A. De Cassai, M. Franco, F. Facchin, C. Ori, S. Rossi, A. Goffi, S. H. Li, H. C. Hu, L. C. Chiu, C. Y. Hung, C. H. Chang, K. C. Kao, B. Llorente Ruiz, J. Lujan Varas, R. Molina Montero, C. Pintado Delgado, O. Navarrete, M. Vazquez Mezquita, E. Alonso Peces, M. A. M. Nakamura, L. A. Hajjar, F. R. B. G. Galas, T. A. Ortiz, M. B. P. Amato, L. Bitker, N. Costes, D. Le Bars, F. Lavenne, D. Mojgan, J. C. Richard, C. Chiurazzi, M. Cressoni, D. Massari, M. Guanziroli, G. Vergani, M. Gotti, M. Brioni, I. Algieri, P. Cadringher, T. Tonetti, D. Chiumello, L. Gattinoni, A. Zerman, M. Türkoğlu, G. Arık, F. Yıldırım, Z. Güllü, I. Kara, N. Boyacı, B. Basarık Aydoğan, Ü. Gaygısız, K. Gönderen, G. Aygencel, M. Aydoğdu, Z. Ülger, G. Gürsel, J. Riera, C. Maldonado Toral, C. Mazo, M. Martínez, J. Baldirà, L. Lagunes, A. Roman, M. Deu, J. Rello, D. J. Levine, R. M. Mohus, Å. Askim, J. Paulsen, A. Mehl, A. T. Dewan, J. K. Damås, E. Solligård, B. O. Åsvold, Mid-Norway Sepsis Research Center, J. Paulsen, Å. Askim, R. M. Mohus, A. Mehl, A. DeWan, E. Solligård, J. K. Damås, B. O. Åsvold, O. Aktepe, A. Kara, H. Yeter, A. Topeli, M. Norrenberg, M. Devroey, H. Khader, J. C. Preiser, Z. Tang, C. Qiu, L. Tong, C. Cai, M. Theodorakopoulou, A. Diamantakis, M. Kontogiorgi, E. Chrysanthopoulou, T. Christodoulopoulou, F. Frantzeskaki, M. Lygnos, O. Apostolopoulou, A. Armaganidis, J. Y. Moon, M. R. Park, I. S. Kwon, G. R. Chon, J. Y. Ahn, S. J. Kwon, Y. J. Chang, J. Y. Lee, S. Y. Yoon, J. W. Lee, The Korean Chungcheong Critical Care Research Group, M. Kostalas, J. Mckinlay, G. Kooner, G. Dudas, A. Horton, C. Kerr, N. Karanjia, B. Creagh-Brown, N. D. Altintas, S. Izdes, O. Keremoglu, A. Alkan, S. Neselioglu, O. Erel, N. Tardif, T. Gustafsson, O. Rooyackers, K. N. MacEachern, M. Traille, I. Bromberg, S. E. Lapinsky, M. J. Moore, Z. Tang, C. Cai, L. Tong, J. L. García-Garmendia, F. Villarrasa-Clemente, F. Maroto-Monserrat, O. Rufo-Tejeiro, V. Jorge-Amigo, M. Sánchez-Santamaría, C. Colón-Pallarés, A. Barrero-Almodóvar, S. Gallego-Lara, C. T. Anthon, R. B. Müller, N. Haase, K. Møller, P. B. Hjortrup, J. Wetterslev, A. Perner, M. Nakanishi, A. Kuriyama, T. Fukuoka, M. A. Abd el Halim, M. H. Elsaid hafez, A. M. Moktar, A. Eladawy, H. M. Elazizy, K. Abdel Hakim, A. Chaari, M. Elbahr, M. Ismail, T. Mahmoud, V. Kauts, K. Bousselmi, E. Khalil, W. Casey, S. H. Zaky, A. Rizk, M. O. Elghonemi, R. Ahmed, J. C. F. Vieira, R. B. Souza, A. M. A. Liberatore, I. H. J. Koh, G. A. Ospina-Tascón, A. F. Garcia Marin, G. J. Echeverry, W. F. Bermudez, H. J. Madriñan-Navia, J. D. Valencia, E. Quiñonez, A. Marulanda, C. A. Arango-Dávila, A. Bruhn, G. Hernandez, D. De Backer, D. Orbegozo Cortes, F. Su, J. L. Vincent, J. Creteur, L. Tullo, L. Mirabella, P. Di Molfetta, G. Cinnella, M. Dambrosio, C. Villavicencio Lujan, J. Leache irigoyen, M. Cartanya ferré, R. Carbonell García, A. Mukhtar, M. Ahmed, M. El Ayashi, A. Hasanin, E. Ayman, M. Salem, A. Eladawy, S. Fathy, H. Nassar, A. Zaghlol, M. F. Aguilar Arzapalo, Å. Valsø, K. Sunde, T. Rustøen, I. Schou-Bredal, L. Skogstad, K. Tøien, C. Padilla, Y. Palmeiro, W. Egbaria, R. Kigli, B. Maertens, K. Blot, S. Blot, E. Santana-Santos, E. R. dos Santos, R. E. D. L. Ferretti-Rebustini, R. D. C. C. D. O. dos Santos, R. G. S. Verardino, L. A. Bortolotto, A. M. Doyle, I. Naldrett, J. Tillman, S. Price, S. Shrestha, P. Pearson, J. Greaves, D. Goodall, A. Berry, A. Richardson, G. O. Odundo, P. Omengo, P. Obonyo, N. M. Chanzu, R. Kleinpell, S. J. Sarris, P. Nedved, M. Heitschmidt, H. Ben-Ghezala, S. Snouda, S. Djobbi, H. Ben-Ghezala, S. Snouda, L. Rose, N. K. J. Adhikari, D. Leasa, D. Fergusson, D. A. Mckim, J. Weblin, O. Tucker, D. McWilliams, F. Doesburg, F. Cnossen, W. Dieperink, W. Bult, M. W. N. Nijsten, G. A. Galvez-Blanco, E. Monares Zepeda, C. I. Olvera Guzman, J. S. Aguirre Sánchez, J. Franco Granillo, J. Santos Stroud, R. Thomson, M. Llaurado-Serra, A. Lobo-Civico, M. Pi-Guerrero, I. Blanco-Sanchez, A. Piñol-Tena, C. Paños-Espinosa, Y. Alabart-Segura, B. Coloma-Gomez, A. Fernandez-Blanco, F. Braga-Dias, M. Treso-Geira, A. Valeiras-Valero, L. Martinez-Reyes, A. Sandiumenge, M. F. Jimenez-Herrera, CAPCRI Study, R. Prada, P. Juárez, R. Argandoña, J. J. Díaz, C. Sánchez Ramirez, P. Saavedra, S. Ruiz Santana, O. Obukhova, S. Kashiya, I. A. Kurmukov, A. M. Pronina, P. Simeone, L. Puybasset, G. Auzias, O. Coulon, B. Lesimple, G. Torkomian, L. Velly, A. Bienert, A. Bartkowska-Sniatkowska, P. Wiczling, O. Szerkus, D. Siluk, J. Bartkowiak-Wieczorek, J. Rosada-Kurasinska, J. Warzybok, A. Borsuk, R. Kaliszan, E. Grzeskowiak, C. Hernandez Caballero, S. Roberts, G. Isgro, D. Hall, G. Guillaume, O. Passouant, F. Dumas, W. Bougouin, B. Champigneulle, M. Arnaout, J. Chelly, J. D. Chiche, O. Varenne, J. P. Mira, E. Marijon, A. Cariou, M. Beerepoot, H. R. Touw, K. Parlevliet, C. Boer, P. W. Elbers, P. R. Tuinman, Á. J. Roldán Reina, Y. Corcia Palomo, R. Martín Bermúdez, L. Martín Villén, I. Palacios García, J. R. Naranjo Izurieta, J. B. Pérez Bernal, F. J. Jiménez Jiménez, Cardiac Arrest Group HUVR, F. Cota-Delgado, M. V. de la Torre-Prados, A. Fernández-Porcel, P. Nuevo-Ortega, E. Cámara-Sola, T. Tsvetanova-Spasova, C. Rueda-Molina, L. Salido-Díaz, A. García-Alcántara, T. Kaneko, H. Tanaka, M. Kamikawa, R. Karashima, S. Iwashita, H. Irie, S. Kasaoka, O. Arola, R. Laitio, A. Saraste, J. Airaksinen, M. Pietilä, M. Hynninen, J. Wennervirta, M. Bäcklund, E. Ylikoski, P. Silvasti, E. Nukarinen, J. Grönlund, V. P. Harjola, J. Niiranen, K. Korpi, M. Varpula, R. O. Roine, T. Laitio, for the Xe-HYPOTHECA study group, S. Salah, B. G. Hassen, A. Mohamed Fehmi, S. Kim, Y. C. Hsu, J. Barea-Mendoza, C. García-Fuentes, M. Castillo-Jaramillo, H. Dominguez-Aguado, R. Viejo-Moreno, L. Terceros-Almanza, S. Bermejo Aznárez, C. Mudarra-Reche, W. Xu, M. Chico-Fernández, J. C. Montejo-González, K. Crewdson, M. Thomas, M. Merghani, L. Fenner, P. Morgan, D. Lockey, E. J. van Lieshout, B. Oomen, J. M. Binnekade, D. A. Dongelmans, R. J. de Haan, N. P. Juffermans, M. B. Vroom, R. Algarte, L. Martínez, B. Sánchez, I. Romero, F. Martínez, S. Quintana, J. Trenado, O. Sheikh, D. Pogson, R. Clinton, F. Riccio, L. Gemmell, A. MacKay, A. Arthur, L. Young, A. Sinclair, D. Markopoulou, K. Venetsanou, L. Filippou, E. Salla, S. Stratouli, I. Alamanos, A. H. Guirgis, R. Gutiérrez Rodriguez, M. J. Furones Lorente, I. Macias Guarasa, A. Ukere, S. Meisner, G. Greiwe, B. Opitz, D. Benten, B. Nashan, L. Fischer, C. J. C. Trepte, D. A. Reuter, S. A. Haas, C. R. Behem, G. Tavazzi, B. Ana, A. Vazir, D. Gibson, S. Price, M. Masjedi, M. R. Hadavi, M. Riahi alam, M. R. Sasani, N. Parenti, F. Agrusta, C. Palazzi, B. Pifferi, R. Sganzerla, F. Tagliazucchi, A. Luciani, M. Möller, J. Müller-Engelmann, G. Montag, P. Adams, C. Lange, J. Neuzner, R. Gradaus, K. H. Wodack, F. Thürk, A. D. Waldmann, M. F. Grässler, S. Nishimoto, S. H. Böhm, E. Kaniusas, D. A. Reuter, C. J. Trepte, T. Sigmundsson, T. Öhman, E. Redondo, M. Hallbäck, M. Wallin, F. Suarez Sipman, A. Oldner, C. Hällsjö Sander, H. Björne, L. Colinas, G. Hernandez, R. Vicho, M. Serna, R. Cuena, A. Canabal, ECOCRITIC group, A. Chaari, K. Abdel Hakim, M. Etman, M. El Bahr, A. El Sakka, K. Bousselmi, A. Arali, V. Kauts, W. F. Casey, O. Bond, P. De Santis, E. Iesu, F. Franchi, J. L. Vincent, J. Creteur, S. Scolletta, F. S. Taccone, Z. Marutyan, L. Hamidova, A. Shakotko, V. Movsisyan, I. Uysupova, A. Evdokimov, S. Petrikov, C. Gonen, E. Haftacı, C. Balci, F. J. Redondo Calvo, N. Bejarano, V. Baladron, R. Villazala, J. Redondo, D. Padilla, P. Villarejo, A. Akcan-Arikan, C. E. Kennedy, M. F. Aguilar Arzapalo, C. Gomez-Gonzalez, S. Mas-Font, A. Puppo-Moreno, M. Herrera-Gutierrez, M. Garcia-Garcia, S. Aldunate-Calvo, NEFROCON Investigators, E. P. Plata-Menchaca, X. L. Pérez-Fernández, M. Estruch, A. Betbese-Roig, P. Cárdenas Campos, M. Rojas Lora, N. D. Toapanta Gaibor, R. S. Contreras Medina, V. D. Gumucio Sanguino, E. J. Casanova, J. Sabater Riera, SIRAKI group, K. Kritmetapak, S. Peerapornratana, P. Kittiskulnam, T. Dissayabutra, K. Tiranathanagul, P. Susantithapong, K. Praditpornsilpa, K. Tungsanga, S. Eiam-Ong, N. Srisawat, T. Winkelmann, T. Busch, J. Meixensberger, S. Bercker, E. M. Flores Cabeza, M. Sánchez Sánchez, N. Cáceres Giménez, C. Gutierrez Melón, E. Herrero de Lucas, P. Millán Estañ, M. Hernández Bernal, A. Garcia de Lorenzo y Mateos, B. Ergin, P. Guerci, P. A. C. Specht, Y. Ince, C. Ince, M. Balik, M. Zakharchenko, F. Los, H. Brodska, C. de Tymowski, P. Augustin, M. Desmard, P. Montravers, S. N. Stapel, R. de Boer, H. M. Oudemans, A. Hollinger, T. Schweingruber, F. Jockers, M. Dickenmann, M. Siegemund, Clinical Intensive Care Research Basel, N. Runciman, M. Ralston, R. Appleton, T. Mauri, L. Alban, C. Turrini, T. Sasso, T. Langer, M. Panigada, P. Taccone, E. Carlesso, C. Marenghi, G. Grasselli, A. Pesenti, P. Wibart, T. Reginault, M. Garcia, B. Barbrel, A. Benard, C. Bader, F. Vargas, H. N. Bui, G. Hilbert, J. M. Serrano Simón, P. Carmona Sánchez, F. Ruiz Ferrón, M. García de Acilu, J. Marin, V. Antonia, L. Ruano, M. Monica, R. Ferrer, J. R. Masclans, O. Roca, G. Hong, D. H. Kim, Y. S. Kim, J. S. Park, Y. K. Jee, Z. Yu xiang, W. Jia-xing, W. Xiao dan, N. Wen long, W. Yu, Z. Yan, X. Cheng, T. Kobayashi, Y. Onodera, R. Akimoto, A. Sugiura, H. Suzuki, M. Iwabuchi, M. Nakane, K. Kawamae, P. Carmona Sanchez, M. D. Bautista Rodriguez, M. Rodriguez Delgado, V. Martínez de Pinillos Sánchez, A. Mula Gómez, J. M. Serrano Simón, P. Beuret, C. Fortes, M. Lauer, M. Reboul, J. C. Chakarian, X. Fabre, B. Philippon-Jouve, S. Devillez, M. Clerc, N. Rittayamai, M. Sklar, M. Dres, M. Rauseo, C. Campbell, B. West, D. E. Tullis, L. Brochard, Y. Onodera, R. Akimoto, H. Suzuki, M. Okada, M. Nakane, K. Kawamae, N. Ahmad, M. Wood, A. Glossop, J. Higuera Lucas, A. Blandino Ortiz, D. Cabestrero Alonso, R. De Pablo Sánchez, L. Rey González, R. Costa, G. Spinazzola, A. Pizza, G. Ferrone, M. Rossi, M. Antonelli, G. Conti, H. Ribeiro, J. Alves, M. Sousa, P. Reis, C. S. Socolovsky, R. P. Cauley, J. E. Frankel, A. L. Beam, K. O. Olaniran, F. K. Gibbons, K. B. Christopher, J. Pennington, P. Zolfaghari, H. S. King, H. H. Y. Kong, H. P. Shum, W. W. Yan, C. Kaymak, N. Okumus, A. Sari, B. Erdogdu, S. Aksun, H. Basar, A. Ozcan, N. Ozcan, D. Oztuna, J. A. Malmgren, S. Lundin, K. Torén, M. Eckerström, A. Wallin, A. C. Waldenström, for the Section on Ethics of the ESICM, F. C. Riccio, D. Pogson, A. C. P. Antonio, A. F. Leivas, F. Kenji, E. James, P. Morgan, G. Carroll, L. Gemmell, A. MacKay, C. Wright, J. Ballantyne, S. Jonnada, C. S. Gerrard, N. Jones, J. D. Salciccioli, D. C. Marshall, M. Komorowski, A. Hartley, M. C. Sykes, R. Goodson, J. Shalhoub, J. R. Fernández Villanueva, R. Fernández Garda, A. M. López Lago, E. Rodríguez Ruiz, R. Hernández Vaquero, C. Galbán Rodríguez, E. Varo Pérez, C. Hilasque, I. Oliva, G. Sirgo, M. C. Martin, M. Olona, M. C. Gilavert, M. Bodí, C. Ebm, G. Aggarwal, S. Huddart, N. Quiney, M. Cecconi, S. M. Fernandes, J. Santos Silva, J. Gouveia, D. Silva, R. Marques, H. Bento, A. Alvarez, Z. Costa Silva, D. Díaz Diaz, M. Villanova Martínez, E. Palencia Herrejon, A. Martinez de la Gandara, G. Gonzalo, M. A. Lopez, P. Ruíz de Gopegui Miguelena, C. I. Bernal Matilla, P. Sánchez Chueca, M. D. C. Rodríguez Longares, R. Ramos Abril, A. L. Ruíz Aguilar, R. Garrido López de Murillas, R. Fernández Fernández, P. Morales Laborías, M. A. Díaz Castellanos, M. E. Morales Laborías, J. Cho, J. Kim, J. Park, S. Woo, T. West, E. Powell, A. Rimmer, C. Orford, N. Jones, J. Williams, C. I. Bernal Matilla, P. Ruiz de Gopegui Miguelena, P. Sánchez Chueca, R. Ramos Abril, M. D. C. Rodríguez Longares, A. L. Ruíz Aguilar, R. Garrido López de Murillas, R. S. Bourne, R. Shulman, M. Tomlin, G. H. Mills, M. Borthwick, W. Berry, D. García Huertas, F. Manzano, F. Villagrán-Ramírez, A. Ruiz-Perea, C. Rodríguez-Mejías, F. Santiago-Ruiz, M. Colmenero-Ruiz, C. König, B. Matt, A. Kortgen, C. S. Hartog, A. Wong, C. Balan, G. Barker, N. Srisawat, S. Peerapornratana, P. Laoveeravat, S. Tachaboon, S. Eiam-ong, J. Paratz, G. Kayambu, R. Boots, M. F. Aguilar Arzapalo, R. Vlasenko, E. Gromova, S. Loginov, M. Kiselevskiy, Y. Dolgikova, K. B. Tang, C. M. Chau, K. N. Lam, E. Gil, G. Y. Suh, C. M. Park, J. Park, C. R. Chung, C. T. Lee, A. Chao, P. Y. Shih, Y. F. Chang, C. H. Lai, Y. C. Hsu, Y. C. Yeh, Y. J. Cheng, V. Colella, N. Zarrillo, M. D’Amico, F. Forfori, B. Pezza, T. Laddomada, V. Beltramelli, M. L. Pizzaballa, A. Doronzio, B. Balicco, D. Kiers, W. van der Heijden, J. Gerretsen, Q. de Mast, S. el Messaoudi, G. Rongen, M. Gomes, M. Kox, P. Pickkers, N. P. Riksen, Y. Kashiwagi, M. Okada, K. Hayashi, Y. Inagaki, S. Fujita, M. N. Nakamae, Y. R. Kang, R. B. Souza, A. M. A. Liberatore, I. H. J. Koh, A. Blet, M. Sadoune, J. Lemarié, N. Bihry, R. Bern, E. Polidano, R. Merval, J. M. Launay, B. Lévy, J. L. Samuel, A. Mebazaa, J. Hartmann, S. Harm, V. Weber

**Affiliations:** 1 Academic Medical Center, University of Amsterdam, Amsterdam, Netherlands; 2 UMCU, Utrecht, Netherlands; 3 Autonomous University of Barcelona, Barcelona, Spain; 4 Hospital São Francisco Xavier, Lisbon, Portugal; 5 CHU Henri Mondor, Medical ICU, Créteil, France; 6 Université de Paris Est-Créteil, Groupe de Recherche Clinique CARMAS, Créteil, France; 7 Hôpital Tenon, APHP, Medical and Surgical ICU, Paris, France; 8 Université Paris Diderot, Sorbonne Paris Cité, UMR 1153, Paris, France; 9 Interdepartmental Division of Critical Care, University of Toronto, Toronto, Canada; 10 University Health Network, University of Toronto, Critical Care Medicine, Toronto, Canada; 11 University of Milan - Bicocca, Health Sciences, School of Medicine and Surgery, Monza, Italy; 12 St Michael’s Hospital, University of Toronto, Anesthesia, Toronto, Canada; 13 CHU d’Angers, Medical ICU, Angers, France; 14 Interdepartmental Division of Critical Care, University of Toronto Saint Michael’s Hospital and Keenan Research Centre, Toronto, Canada; 15 University of Birmingham, Institute for Inflammation and Ageing, Birmingham, UK; 16 University of Sheffield, Mathematics and Statistics Help Centre, Sheffield, UK; 17 Heart of England NHS Foundation Trust, Intensive Care Medicine, Birmingham, UK; 18 Department of Surgery, University Hospitals Birmingham NHS Trust, Birmingham, UK; 19 University Hospitals Birmingham NHS Trust, Anaesthesia and Critical Care Medicine, Birmingham, UK; 20 Queen’s University of Belfast, Wellcome-Wolfson Institute for Experimental Medicine, Belfast, UK; 21 Royal Victoria Hospital, Intensive Care Medicine, Belfast, UK; 22 University of Warwick, Warwick Clinical Trials Unit, Coventry, UK; 23 St Michael’s Hospital, University of Toronto, Critical Care, Toronto, Canada; 24 St Michael’s Hospital, University of Toronto, Anesthesia, Toronto, Canada; 25 University of Milan-Bicocca, Health Sciences, School of Medicine and Surgery, Monza, Italy; 26 University Health Network, University of Toronto, Critical Care Medicine, Toronto, Canada; 27 Academic Medical Center, University of Amsterdam, Amsterdam, Netherlands; 28 University of Bari ‘Aldo Moro’, Bari, Italy; 29 Hospital Israelita Albert Einstein, São Paulo, Brazil; 30 Mayo Clinic, Jacksonville, USA; 31 Mahidol University, Faculty of Tropical Medicine, Bangkok, Thailand; 32 University Medical Center Utrecht, Intensive Care, Utrecht, Netherlands; 33 University Medical Center Utrecht, Julius Center for Health Sciences and Primary Care, Utrecht, Netherlands; 34 Academic Medical Center, University of Amsterdam, Center for Experimental and Molecular Medicine, Amsterdam, Netherlands; 35 Academic Medical Center, University of Amsterdam, Center for Infection and Immunity Amsterdam, Amsterdam, Netherlands; 36 Academic Medical Center, University of Amsterdam, Intensive Care, Amsterdam, Netherlands; 37 Academic Medical Center, University of Amsterdam, Infectious Diseases, Amsterdam, Netherlands; 38 University Medical Center Utrecht, Medical Microbiology, Utrecht, Netherlands; 39 Seoul National University Hospital, Seoul, Republic of Korea; 40 Department of Emergency Medicine, Seoul National University Bundang Hospital, Seongnam-si, Republic of Korea; 41 Department of Emergency Medicine, Seoul National University Boramae Hospital, Seoul, Republic of Korea; 42 Department of Pulmonology, Hospital Clinic of Barcelona, University of Barcelona, Institut D’investigacions August Pi I Sunyer (IDIBAPS), Barcelona, Spain; 43 Centro de Investigación Biomedica En Red-Enfermedades Respiratorias (CibeRes, CB06/06/0028), Barcelona, Spain; 44 Respiratory Intensive Care Unit, Pulmonary Division, Heart Institute, Hospital das Clínicas, University of Sao Paulo, São Paulo, Brazil; 45 Pneumology Department, ISS/Hospital Universitario y Politecnico La Fe, CIBER Enfermedades Respiratorias (CIBERES), Valencia, Spain; 46 Seccion Neumologia, Hospital Nacional Prof. Alejandro Posadas, Palomar, Argentina; 47 Department of Mathematics, University of Pittsburgh, Pittsburgh, USA; 48 Department of Chemical and Petroleum Engineering, University of Pittsburgh, Pittsburgh, USA; 49 University of Pittsburgh, Biostatistics, Pittsburgh, USA; 50 Department of Critical Care Medicine, University of Pittsburgh, CRISMA Center, Pittsburgh, USA; 51 University of Pittsburgh, McGowan Institute for Regenerative Medicine, Pittsburgh, USA; 52 Department of Anesthesiology, University of Greifswald, Greifswald, Germany; 53 University of Greifswald, Greifswald, Germany; 54 University of Greifswald, Institute of Bioinfomatics, University Medicine Greifswald, Greifswald, Germany; 55 St. Johns Medical College, Bangalore, India; 56 Yashoda Hospials, Secundrabad, India; 57 University of Szeged Faculty of Medicine, Anaesthesiology and Intensive Therapy, Szeged, Hungary; 58 Medical University of Vienna, Anaesthesiology and General Intensive Care Medicine, Vienna, Austria; 59 University of Szeged Faculty of Medicine, Surgical Research, Szeged, Hungary; 60 Technische Universität München, Gastroenterology, Munich, Germany; 61 Facultad de Ciencias Médicas, Universidad Nacional de La Plata, Cátedra de Farmacología Aplicada, La Plata, Argentina; 62 Academic Medical Center, University of Amsterdam, Translational Physiology, Amsterdam, Netherlands; 63 St George’s University Hospitals NHS Foundation Trust, Adult Critical Care Directorate, London, UK; 64 St George’s, University of London, London, UK; 65 Hôpital Louis Pradel, Anesthésie Réanimation, Bron, France; 66 University Claude Bernard Lyon 1, Lyon, France; 67 Hôpital Femme Mère Enfant, Lyon, France; 68 Intensive Care Unit, Hospital Virgen del Rocio, Sevilla, Spain; 69 Department of Anaesthesiology, Critical Care, Pain and Emergency Medicine, Nimes University Hospital, Nimes, France; 70 University of Queensland, Burns Trauma Critical Care Research Centre, Brisbane, Australia; 71 Department of Intensive Care Medicine, Royal Brisbane & Womens Hospital, Brisbane, Australia; 72 Hospital del Mar-IMIM, Critical Care, Barcelona, Spain; 73 Hospital del Mar, Pharmacy, Barcelona, Spain; 74 Universidad Autónoma de Barcelona, Barcelona, Spain; 75 Universidad Pompeu Fabra, Barcelona, Spain; 76 CIBERES. Instituto de Salud Carlos III, Madrid, Spain; 77 Hospital Universitari Arnau de Vilanova, Lleida, Spain; 78 N.I. Pirogov National Medical Surgical Center, Anestesiology and Intensive Care, Moscow, Russian Federation; 79 N.I. Pirogov National Medical Surgical Center, Microbiology Laboratory, Moscow, Russian Federation; 80 Pitie-Salpetriere Hospital and Pierre and Marie Curie University, Paris, France; 81 Department of Anaesthesia and Intensive Care Medicine, Aalborg University Hospital, Aalborg, Denmark; 82 Department of Clinical Medicine, Aalborg University, Aalborg, Denmark; 83 Department of Clinical Biochemistry, Aalborg University Hospital, Aalborg, Denmark; 84 Department of Chemistry and Bioscience, Section of Biotechnology, Aalborg University, Aalborg, Denmark; 85 Policlinico Di Milano, Milan, Italy; 86 Hospital Clinic, Barcelona, Spain; 87 National Institutes of Health, Bethesda, USA; 88 Massachusetts General Hospital, Boston, USA; 89 Ospedale Nuovo del Mugello, Borgo San Lorenzo (FI), Italy; 90 Gruppo Ospedaliero San Donato, San Donato M.se, Italy; 91 Arcispedale S. Maria Nuova, Reggio Emilia, Italy; 92 Ospedale San Giovanni Bosco, Torino, Italy; 93 Policlinico San Matteo, Pavia, Italy; 94 Policlinico di Modena, Modena, Italy; 95 Azienda Ospedaliero - Universitaria di Parma, Parma, Italy; 96 Ospedale Città di Sesto San Giovanni, Sesto San Giovanni, Italy; 97 Policlinico Gemelli, Roma, Italy; 98 Azienda Ospedaliero-Universitaria di Parma, Parma, Italy; 99 Azienda Ospedaliero-Universitaria di Parma, Parma, Italy; 100 University Hospital Frankfurt, Frankfurt, Germany; 101 University Hospital Center Zagreb, Zagreb, Croatia; 102 Ospedale Santa Chiara, Trento, Italy; 103 University of Göttingen, Göttingen, Germany; 104 School of Nursing, Shiraz University of Medical Science, Shiraz, Islamic Republic of Iran; 105 Shiraz University of Medical Sciences, Anesthesiology and Critical Care Research Center, Shiraz, Islamic Republic of Iran; 106 Shiraz University of Medical Sciences, Shiraz, Islamic Republic of Iran; 107 University of Milan - Bicocca, Monza, Italy; 108 University of Goettingen, Goettingen, Germany; 109 Fondazione IRCCS Ospedale Maggiore Policlinico, Anesthesia and Critical Care, Milan, Italy; 110 Plug Working Group, ESICM, Milan, Italy; 111 University of Milan, Milan, Italy; 112 University of Helsinki and Helsinki University Hospital, Intensive Care Medicine, Helsinki, Finland; 113 Institute for Molecular Medicine Finland (FIMM), University of Helsinki, Helsinki, Finland; 114 Folkhälsan Institute of Genetics, Folkhälsan Research Center, Helsinki, Finland; 115 VU Medical Centre, Intensive Care Adults, Amsterdam, Netherlands; 116 Federal University of São Paulo, Surgery, São Paulo, Brazil; 117 Universidade Federal de São Paulo, Morphology and Genetics, São Paulo, Brazil; 118 Biological Institute of São Paulo, São Paulo, Brazil; 119 Hospital Santa Lucía, Intensive Care, Cartagena, Spain; 120 Hospital Santa Lucía, Análisis Clínicos, Cartagena, Spain; 121 Hospital Santa Lucía, Cartagena, Spain; 122 Hospita Santa Lucía, Intensive Care, Cartagena, Spain; 123 Department of Physiology, School of Medicine Murcia, Murcia, Spain; 124 Department of Translational Physiology, Academic Medical Center, University of Amsterdam, Amsterdam, Netherlands; 125 Departement of Anaesthesiology and Critical Care Medicine, University Hospital of Nancy, Vandoeuvre-Les-Nancy, France; 126 University of Lorraine, INSERM U1116, Vandoeuvre-Les-Nancy, France; 127 Department of Medical Technical Innovation & Development (MIO), Academic Medical Center, University of Amsterdam, Amsterdam, Netherlands; 128 Academic Medical Centre Amsterdam, Translational Physiology, Amsterdam, Netherlands; 129 Department of Energency Medicine, Harvard Medical School, Boston, USA; 130 Academic Medical Center, University of Amsterdam, Transaltional Physiology, Amsterdam, Netherlands; 131 Department of Cardio-thoracic Surgery, Acibadem University, Istanbul, Turkey; 132 Queen Mary University of London, WHRI, Translational Medicine and Therapeutics, London, UK; 133 Barts Health NHS Trust, Adult Critical Care Unit, London, UK; 134 Barts Health NHS Trust, Trauma Sciences, Blizzard Institute, London, UK; 135 Department of Intensive Care, Erasmus Medical Center, Rotterdam, Netherlands; 136 Department of Public Health, Erasmus Medical Centre, Rotterdam, Netherlands; 137 Department of Intensive Care, Albert Schweitzer Hospital, Dordrecht, Netherlands; 138 Department of Intensive Care, Ikazia Hospital, Rotterdam, Netherlands; 139 Department of Intensive Care, IJsselland Hospital, Rotterdam, Netherlands; 140 Department of Intensive Care, Sint Franciscus Gasthuis, Rotterdam, Netherlands; 141 Department of Intensive Care, Maasstad Hospital, Rotterdam, Netherlands; 142 KU Leuven, Academic Centre for Nursing and Midwifery, Leuven, Belgium; 143 Department of Pediatric Surgery, Intensive Care Unit, Erasmus Medical Center - Sophia Children’s Hospital, Rotterdam, Netherlands; 144 Third Faculty of Medicine, Charles University in Prague, Laboratory for Metabolism and Bioenergetics, Prague, Czech Republic; 145 Third Faculty of Medicine, Charles University in Prague, Centre for Diabetes, Metabolism and Nutrition, Prague, Czech Republic; 146 Department of Anaesthesiology and Intensive Care, Third Faculty of Medicine, Charles University in Prague, Prague, Czech Republic; 147 Nuffield Division of Anaesthetics, Oxford University Hospitals NHS Foundation Trust, Oxford, UK; 148 University of Oxford, Weatherall Institute of Molecular Medicine, Oxford, UK; 149 Oxford University Hospitals NHS Foundation Trust, Systematic Review Initiative NHS Blood & Transplant, Oxford, UK; 150 Department of Haematology, Oxford University Hospitals NHS Foundation Trust, Oxford, UK; 151 Department of Anesthesiology, Nordsjællands Hospital, University of Copenhagen, Hillerød, Denmark; 152 Department of Nursing, Metropolitan University, Copenhagen, Denmark; 153 Nordsjællands Hospital, Research Unit, Hillerød, Denmark; 154 Department of Neuroanesthesiology, University of Copenhagen, Rigshospitalet, Copenhagen, Denmark; 155 University of Copenhagen, Health & Medical Sciences, Copenhagen, Denmark; 156 N.I. Pirogov National Medical Surgical Center, Anestesiology and Intensive Care, Moscow, Russian Federation; 157 N.I. Pirogov National Medical Surgical Center, Moscow, Russian Federation; 158 Department of Anesthesiology, Nordsjællands Hospital, University of Copenhagen, Hillerød, Denmark; 159 University of Copenhagen, Health & Medical Sciences, Copenhagen, Denmark; 160 Department of Neuroanesthesiology, University of Copenhagen, Rigshospitalet, Copenhagen, Denmark; 161 Department of Psychology, University of Southern Denmark, Odense, Denmark; 162 Department of Anesthesiology, Herlev Hospital, University of Copenhagen, Herlev, Denmark; 163 Department of Anesthesiology, Hospital of Horsens, Horsens, Denmark; 164 Department of Nursing, Metropolitan University, Copenhagen, Denmark; 165 Nordsjællands Hospital, Research Unit, Hillerød, Denmark; 166 University of Pittsburgh, School of Nursing, Pittsburgh, USA; 167 Carnegie Mellon University, Robotics Institute, Auton Lab, Pittsburgh, USA; 168 University of Pittsburgh, Critical Care Medicine, Pittsburgh, Pennsylvania USA; 169 Department of Physiotherapy, The University of Melbourne, Melbourne, Australia; 170 Department of Physiotherapy, Royal Melbourne Hospital, Melbourne, Australia; 171 Guy’s and St Thomas’ NHS Foundation Trust and Kings College, London, UK; 172 Guy’s & St Thomas’ NHS Foundation Trust, Lane Fox Respiratory Unit, London, UK; 173 University of South Australia, Member of the International Centre for Allied Health Evidence (iCAHE) and the Sansom Institute, Adelaide, Australia; 174 Department of Physiotherapy, Flinders Medical Centre, Adelaide, Australia; 175 Division of Respiratory and Critical Care, National University Hospital, Singapore, Singapore; 176 Division of Critical Care, University College London Hospitals, London, UK; 177 University College London Hospitals, Institute of Sports and Exercise Health, London, UK; 178 Division of Asthma, Allergy and Lung Biology, Kings College London, London, UK; 179 Division of Critical Care and Pulmonology, University of Kentucky, Kentucky, USA; 180 Department of Anaesthesiology and Intensive Care, Lillebaelt Hospital, Vejle and Kolding, Denmark; 181 University of Southern Denmark, Institute of Regional Health Research, Odense, Denmark; 182 Department of Geriatrics, Ghent University Hospital, Gent, Belgium; 183 Department of Intensive Care, Ghent University Hospital, Gent, Belgium; 184 Department of Anaesthesiology and Intensive Care, Sahlgrenska University Hospital, Gothenburg, Sweden; 185 King’s College Hospital, Critical Care and Major Trauma, London, UK; 186 Department of Internal Medicine, University of Groningen, University Medical Center Groningen, Groningen, Netherlands; 187 Saint-Etienne University Hospital and Jacques Lisfranc Medical School, Saint-Etienne, France; 188 Department of Anaesthesia and Intensive Care Medicine, General University Hospital, 1st Faculty of Medicine, Charles University in Prague, Prague, Czech Republic; 189 Department of Anesthesia, Critical Care, and Pain Medicine, Beth Israel Deaconess Medical Center and Harvard Medical School, Boston, USA; 190 Institut Jules Bordet, ULB, Service des soins Intensifs et Ergences Oncologiques, Bruxelles, Belgium; 191 Dipartimento Emergenza Urgenza Anestesia e Rianimazione, Ospedale Maggiore della Carità Novara, Novara, Italy; 192 Semmelweis University, Budapest, Hungary; 193 Intensive Care Department, Hospital S.António, Porto, Portugal; 194 Department of Anesthesiology and Critical Care, Tettnang Hospital, Tettnang, Germany; 195 Department of Intensive Care, Erasmus Hospital, Rotterdam, Netherlands; 196 Department of Psychoanalysis and Clinical Consulting, Ghent University, Gent, Belgium; 197 Hôpital Saint Louis, Service de Reanimation Médicale, Paris, France; 198 Department of Applied Mathematics and Computer Science and Statistics, Ghent University, Gent, Belgium; 199 Department of Intensive Care, Ghent University Hospital, Gent, Belgium; 200 Department of Geriatrics, Ghent University Hospital, Gent, Belgium; 201 Department of Anaestesiology and Intensive Care, Lillebaelt Hospital, Veijle and Kolding, Denmark; 202 University of Southern Denmark, Institute of Regional Health Research, Odense, Denmark; 203 Department of Anaesthesiology and Intensive Care, Sahlgrenska University Hospital, Gothenburg, Sweden; 204 King’s College Hospital, Critical Care and Major Trauma, London, UK; 205 Department of Internal Medicine, University of Groningen, University Medical Center Groningen, Groningen, Netherlands; 206 Saint-Etienne University Hospital and Jacques Lisfranc Medical School, Saint-Etienne, France; 207 Department of Anaesthesia and Intensive Care, General University Hospital, 1st Faculty of Medicine, Charles University in Prague, Prague, Czech Republic; 208 Department of Anesthesia, Critical Care, and Pain Medicine, Beth Israel Deaconess Medical Center and Harvard Medical School, Boston, USA; 209 Institut Jules Bordet, ULB, Service des Soins Intensifs et Urgences Oncologiques, Bruxelles, Belgium; 210 Dipartimento Emergenza Urgenza Anestesia e Rianimazione, Ospedale maggiore della carità Novara, Novara, Italy; 211 Semmelweis University, Budapest, Hungary; 212 Department of Intensive Care, Hospital S. António, Porto, Portugal; 213 Department of Anesthesiology and Critical Care, Tettnang Hospital, Tettnang, Germany; 214 Department of Intensive Care, Erasmus Hospital, Rotterdam, Netherlands; 215 Department of Psychoanalysis and Clinical Consulting, Ghent University, Gent, Belgium; 216 Hopital Saint Louis, Service de Reanimation Médicale, Paris, France; 217 Department of Applied Mathematics and Computer Science and Statistics, Ghent University, Gent, Belgium; 218 St Georges NHS Foundation Trust, General Intensive Care, London, UK; 219 Papageorgiou General Hospital, ICU, Thessaloniki, Greece; 220 AHEPA University Hospital, ICU, Thessaloniki, Greece; 221 Technological Educational Institutes of Ipeirus, Ioannina, Greece; 222 University Hospital of Ioannina, ICU, Ioannina, Greece; 223 Royal Free Hospital, Intensive Care Unit, London, UK; 224 University Hospital South Manchester, Manchester, UK; 225 Manchester Academic Health Sciences Centre, University of Manchester, Manchester, UK; 226 University Hospital South Manchester, Manchester, UK; 227 Manchester Academic Health Sciences Centre, University of Manchester, Manchester, UK; 228 Department of Critical Care Medicine, Samsung Medical Center, Sungkyunkwan University School of Medicine, Seoul, Republic of Korea; 229 Royal Cornwall Hospital, Intensive Care Unit, Truro, UK; 230 Karolinska University Hospital Huddinge, Stockholm, Sweden; 231 Department of Intensive Care, VU University Medical Center, Amsterdam, Netherlands; 232 Department of Clinical Pharmacology and Pharmacy, VU University Medical Center, Amsterdam, Netherlands; 233 Department of Internal Medicine Division of Intensive Care, Ankara University Faculty of Medicine, Ankara, Turkey; 234 Department of Anaesthesiology and Intensive Care, Tartu University Hospital, Tartu, Estonia; 235 University of Copenhagen, Faculty of Health and Medical Sciences, Section of Biostatistics, Institute of Public Health, Copenhagen, Denmark; 236 Department of Anaesthesiology and Intensive Care, University of Tartu, Tartu, Estonia; 237 Department of Anaesthesiology, Intensive Care, Emergency and Pain Medicine, Lucerne Cantonal Hospital, Lucerne, Switzerland; 238 Department of Critical Care Medicine, Sri Ramachandra University, Chennai, India; 239 Department of Anesthesiology, Sri Ramachandra University, Chennai, India; 240 Department of Intensive Care, University Medical Center Groningen, University of Groningen, Groningen, Netherlands; 241 Departments of Intensive Care and Anesthesiology, University Medical Center Groningen, University of Groningen, Groningen, Netherlands; 242 Karolinska University Hospital Huddinge, Stockholm, Sweden; 243 Karolinska University Hospital Huddinge, Stockholm, Sweden; 244 Attikon University Hospital, ICU, Athens, Greece; 245 Erciyes University, Intensive Care Unit, Kayseri, Turkey; 246 Internal Medicine Department, Erciyes University, Kayseri, Turkey; 247 Clinical Biochemistry Department, Erciyes University, Kayseri, Turkey; 248 Department of Medicine, Division of Endocrinology, Metabolism and Lipids, Emory University, Atlanta, USA; 249 University College London, Bloomsbury Institute of Intensive Care Medicine, London, UK; 250 Department of Anaesthesiology and Intensive Care Medicine, University Hospital Jena, SG Sepsis Research, Jena, Germany; 251 University Hospital Zurich, University Zurich, Surgical Intensive Care Unit, Zurich, Switzerland; 252 Royal Brompton and Harefield NHS Foundation Trust, London, UK; 253 Anesthesiology and Critical Care Research Center, Shiraz University of Medical Sciences, Shiraz, Islamic Republic of Iran; 254 Shiraz University of Medical Sciences, Trauma Research Center, Shiraz, Islamic Republic of Iran; 255 Shiraz University of Medical Sciences, Shiraz, Islamic Republic of Iran; 256 Chang Gung Memorial Hospital, Thoacic Medicine, Kwei-Shan, Taoyuan, Taiwan, Province of China; 257 Chang Gung Memorial Hospital, Taoyuan, Taiwan, Province of China; 258 Critical Care Department, Faculty of Medicine - Beni Suef University, Cairo, Egypt; 259 Critical Care Department, Faculty of Medicine - Cairo University, Cairo, Egypt; 260 Critical Care Department, Hospital Parc de Salut Mar - GREPAC, IMIM, Barcelona, Spain; 261 Pompeu Fabra University (UPF) - CEXS, Barcelona, Spain; 262 CIBERES. Instituto de Salud Carlos III, Madrid, Spain; 263 Department of Anesthesia and Critical Care, Multidisciplinary Intensive Care Research Organization (MICRO). St James’s; University Hospital. Trinity Center for Health Sciences, Dublin, Ireland; 264 Critical Care Department, Hospital Parc Tauli, Sabadell, Spain; 265 Critical Care Department, Hospital de Mataró, Mataró, Spain; 266 Critical Care Department, University Hospital Joan XXIII - IISPV-URV, Tarragona, Spain; 267 Universitat Autònoma de Barcelona, Barcelona, Spain; 268 Hospital Universitari Vall d’Hebron, Critical Care, Barcelona, Spain; 269 University of Liverpool, Institute of Infection and Global Health, Liverpool, UK; 270 Royal Liverpool University Hospital, Intensive Care Unit, Liverpool, UK; 271 Lille University Hospital, ICU, Lille, France; 272 Farhat Hached University Hospital, Medical Intensive Care Unit, Sousse, Tunisia; 273 Ibn Al Jazzar Faculty of Medicine, University of Sousse, Research Laboratory N° LR14ES05, Interactions of the Cardiopulmonary System, Sousse, Tunisia; 274 Chi Mei Medical Center, Tainan, Taiwan, Province of China; 275 Taipei Medical University, Taipei, Taiwan, Province of China; 276 Critical Care Department, Hospital Parc de Salut Mar - GREPAC, IMIM, Barcelona, Spain; 277 Pompeu Fabra University (UPF) - CEXS, Barcelona, Spain; 278 CIBERES. Instituto de Salud Carlos III, Madrid, Spain; 279 Hospital Parc de Salut Mar - GREPAC, IMIM, Barcelona, Spain; 280 Critical care Department, Hospital de Donosti, San Sebastian, Spain; 281 Critical care Department, Hospital la Fe, Valencia, Spain; 282 Critical care Department, Hospital de Valdecillas, Santander, Spain; 283 Critical care Department, Hospital Gregorio Marañon, Madrid, Spain; 284 Critical care Department, University Joan XXIII Hospital - IISPV - URV, Tarragona, Spain; 285 Hospital Universitari Arnau de Vilanova, Lleida, Spain; 286 Consorci Sanitari del Maresme, Intensive Care Unit, Mataró, Spain; 287 Consorci Sanitari del Maresme, Investigation Unit, Mataró, Spain; 288 Hospital Clínico Universitario de Valladolid, Unidad de Investigación Médica en Infección e Immunidad, Valladolid, Spain; 289 Clinica Sagrada Esperança, ICU, Luanda, Angola; 290 Escola Nacional de Saude Publica/ UNL, Lisboa, Portugal; 291 Clinica Sagrada Esperança, Infectiology, Luanda, Angola; 292 Clinica Sagrada Esperança, Luanda, Angola; 293 University Hospital, UHCW NHS Trust, General Critical Care, Coventry, UK; 294 Department of Anaesthetics and Intensive Care, Dudley Group of Hospitals NHS Foundation Trust, Dudley, UK; 295 Division of Thoracic Medicine, Chang Gung Memorial Hospital, Taoyuan, Taiwan, Province of China; 296 Department of Respiratory Therapy, Chang Gung Memorial Hospital, Taoyuan, Taiwan, Province of China; 297 Department of Respiratory Therapy, Chang Gung University College of Medicine, Taoyuan, Taiwan, Province of China; 298 Azienda Ospedaliera di Padova, UOC Anesthesia and Intensive Care Unit, Padua, Italy; 299 Department of Medicine, University of Padua, Thrombotic and Hemorrhagic Diseases Unit, Padua, Italy; 300 Department of Medicine-DIMED, Azienda Ospedaliera di Padova, UOC Anesthesia and Intensive Care Unit, Padua, Italy; 301 Nippon Medical School Hospital, Tokyo, Japan; 302 Intensive Care Department, University Hospital of Santa Maria, CHLN, Lisbon, Portugal; 303 Centre Hospitalier Intercommunal de Villeneuve Saint Georges, Lucie et Raymond AUBRAC, Reanimation Polyvalente - Surveillance Continue, Villeneuve Saint Georges, France; 304 Department of Anesthesia and Intensive Care, IRCCS-ISMETT Mediterranean Institute for Transplantation and Advanced Therapies, Palermo, Italy; 305 IRCCS-ISMETT Mediterranean Institute for Transplantation and Advanced Therapies, Statistics, Research Office, Palermo, Italy; 306 Hospital Universitario Marqués de Valdecilla, Intensive Care Unit, Santander, Spain; 307 Hospital Universitario Marqués de Valdecilla, Rheumathology Service, Santander, Spain; 308 Universidad de Cantabria, Preventive Medicine and Public Health, Santander, Spain; 309 CHU Amiens - Picardie, Cardio Thoracic and Vascular Intensive Care Unit, Amiens, France; 310 Université de Picardie Jules Verne, CURS, Amiens, France; 311 CHU Amiens - Picardie, Cardiac Surgery, Amiens, France; 312 University Hospital of Liège, Medical and Coronary Intensive Care, Liege, Belgium; 313 University of Liège, GIGA Research, Liège, Belgium; 314 University Hospital of Liège, Perfusion School, Liege, Belgium; 315 Department of Medicine (DIMED), University of Padova, Padova, Italy; 316 Emergency Department, Azienda Ospedaliera di Padova, Padova, Italy; 317 Dr. Suat Seren Chest Diseases and Surgery Training Hospital, Intensive Care Unit, Izmir, Turkey; 318 Royal Infirmary of Edinburgh, Ward 118, Edinburgh, UK; 319 Royal Infirmary of Edinburgh, Edinburgh, UK; 320 PAH Harlow, Intensive Care, Harlow, UK; 321 Colchester NHSFT, Intensive Care, Colchester, UK; 322 Hospital Regional Málaga, Intensive Care, Málaga, Spain; 323 Hospital Infanta Margarita, Intensive Care, Cabra, Spain; 324 Hospital Virgen del Rocio, Intensive Care, Sevilla, Spain; 325 Hospital Puerta del Mar, Intensive Care, Cádiz, Spain; 326 Hospital Regional Málaga, Málaga, Spain; 327 Guy’s & St Thomas’ NHS Foundation Trust, London, UK; 328 Sverdlovsk Regional Clinical Hospital 1, Ekaterinburg, Russian Federation; 329 Guys and St Thomas’ NHS Foundation Trust, London, UK; 330 Royal Surrey County Hospital, Guildford, UK; 331 Royal Surrey County Hospital, ICU and SPACeR research group, Guildford, UK; 332 Cheetah Medical, Inc., Director of Clinical Operations, Newton Center, USA; 333 Health Economics Trexin, Chief Data Scientist, Chicago, USA; 334 Cheetah Medical, Inc., Chief Physician Executive, Newton Center, USA; 335 Excellence Center for Critical Care Nephrology, King Chulalongkorn Memorial Hospital, Thai Red Cross and Faculty of Medicine, Chulalongkorn University, Bangkok, Thailand; 336 Division of Nephrology, Department of Medicine, King Chulalongkorn Memorial Hospital, Thai Red Cross and Faculty of Medicine, Chulalongkorn University, Bangkok, Thailand; 337 Department of Critical Care Medicine, Center for Critical Care Nephrology, CRISMA Center, University of Pittsburgh School of Medicine, Pittsburgh, USA; 338 Department of Anesthesiology, King Chulalongkorn Memorial Hospital, Thai Red Cross and Faculty of Medicine, Chulalongkorn University, Bangkok, Thailand; 339 Department of Anaesthesiology and Intensive Care, Nordsjællands Hospital, Hillerød, Denmark; 340 Department of Intensive Care, Rigshospitalet, Copenhagen University Hospital, Copenhagen, Denmark; 341 Department of Biostatistics, University of Copenhagen, Copenhagen, Denmark; 342 Department of Infectious Diseases, Rigshospitalet, Copenhagen University Hospital, CHIP & PERSIMUNE, Copenhagen, Denmark; 343 University of Texas Health Science Center San Antonio, Pediatric Critical Care Medicine, San Antonio, USA; 344 Baylor College of Medicine, Pediatric Cardiology, Houston, USA; 345 Baylor College of Medicine, Pediatric Critical Care Medicine, Houston, USA; 346 Baylor College of Medicine, Pediatric Nephrology, Houston, USA; 347 Federal University of São Paulo, Surgery, São Paulo, Brazil; 348 Federal University of São Paulo, Morphology and Genetics, São Paulo, Brazil; 349 Biological Institute of São Paulo, Sao Paulo, Brazil; 350 Faculty of Medicine, Cairo University, Critical Care Medicine, Cairo, Egypt; 351 Complejo Universitario Carlos Haya, Málaga, Spain; 352 Hospital Parc Tauli, Sabadell, Spain; 353 Hospital Son Llatzer, Palma de Mallorca, Spain; 354 University of Glasgow, Glasgow, UK; 355 Queen Elizabeth Hospital, Critical Care, Glasgow, UK; 356 Centro Universitario de La Costa, Puerto Vallarta, Jalisco Mexico; 1054 Queen Elizabeth University Hospital, Anaesthetics and Intensive Care, Glasgow, UK; 357 Queen Elizabeth University Hospital, Intensive Care Unit, Glasgow, UK; 358 Department of Intensive Care, VU University Medical Center Amsterdam, Adults, Amsterdam, Netherlands; 359 University of Kinshasa, Anaesthesiology and Intensive Care, Kinshasa, Democratic Republic of the Congo; 360 Clinic Caron, Paris, France; 361 University of Kinshasa, Public Heath, Kinshasa, Democratic Republic of the Congo; 362 Hôpital Nord, Reanimation, Marseille, France; 363 Rigshospitalet, Copenhagen University Hospital, Neurointensive Care Unit, Copenhagen, Denmark; 364 University of Gothenburg, The Sahlgrenska Academy, Gothenburg, Sweden; 365 Karolinska Institute, Stockholm, Sweden; 366 Rigshospitalet, Copenhagen University Hospital, Intensive Care Unit, Copenhagen, Denmark; 367 Farhat Hached University Hospital, Medical Intensive Care Unit, Sousse, Tunisia; 368 Farhat Hached University Hospital, Hospital Hygiene Unit, Sousse, Tunisia; 369 Internal Medicine Department, Farhat Hached University Hospital, Sousse, Tunisia; 370 Research Laboratory N° LR14ES05, Interactions of the Cardiopulmonary System, Ibn Al Jazzar Faculty of Medicine, University of Sousse, Sousse, Tunisia; 371 Department of Surgery, Shiraz University of Medical Sciences, Shiraz, Islamic Republic of Iran; 372 Anesthesiology and Critical Care Research Center, Shiraz University of Medical Sciences, Shiraz, Islamic Republic of Iran; 373 Department of Anesthesiology and Intensive Care Medicine, Jena University Hospital, Jena, Germany; 374 Jena University Hospital, Center for Sepsis Control and Care, Jena, Germany; 375 Clinica Sagrada Esperança, ICU, Luanda, Angola; 376 Escola Nacional de Saude Publica/ UNL, Lisboa, Portugal; 377 Clinica Sagrada Esperança, Luanda, Angola; 378 Royal Stoke University Hospital, Critical Care, Stoke-on-Trent, UK; 379 Apollo Hospitals, Intensive Care Medicine, Bangalore, India; 380 SRI Ramachindra University, Intensive Care Medicine, Chennai, India; 381 Hospital Israelita Albert Einstein, ICU, São Paulo, Brazil; 382 Anesthesiology and Critical Care Research Center, Shiraz University of Medical Sciences, Shiraz, Islamic Republic of Iran; 383 International Nosocomial Infection Control Consortium, Buenos Aires, Argentina; 384 Trauma Research Center, Shiraz University of Medical Sciences, Shiraz, Islamic Republic of Iran; 385 Shiraz HIV/AIDS Research Center, Shiraz University of Medical Sciences, Shiraz, Islamic Republic of Iran; 386 Salford Royal NHS Foundation Trust, Critical Care, Salford, UK; 387 Salford Royal NHS Foundation Trust, Manchester, UK; 389 Kocaeli Derince Education and Research Hospital, Kocaeli, Turkey; 390 Department of Anesthesia and Intensive Care, Regional Hospital Liberec, Liberec, Czech Republic; 391 Regional Hospital Liberec, Neurocentre, Liberec, Czech Republic; 392 St Mary’s Hospital, Adult Intensive Care Unit, London, UK; 393 Department of Neurology, Faculty of Medicine, University of Rijeka, Rijeka, Croatia; 394 Department of Anesthesiology, Reanimatology and Intensive Care Medicine, Faculty of Medicine, University of Rijeka, Rijeka, Croatia; 395 Tan Tock Seng Hospital, National Healthcare Group (Singapore), Singapore, Singapore; 396 National University of Singapore, Singapore, Singapore; 397 Hospital Universitario San Cecilio, Granada, Spain; 398 Krasnoyarsk State Medical University, Krasnoyarsk Regional Hospital, Anaesthesiology and Intensive Care, Krasnoyarsk, Russian Federation; 399 Krasnoyarsk Regional Hospital, Krasnoyarsk, Russian Federation; 400 Krasnoyarsk Clinical Regional Hospital, Anaesthesiology and Intensive Care, Krasnoyarsk, Russian Federation; 401 Tan Tock Seng Hospital, National Healthcare Group, Singapore, Singapore; 402 Centro Hospitalar do Médio Tejo, Unidade de Cuidados Intensivos Polivalente, Abrantes, Portugal; 403 Hospital de Santa Maria/CHLN, Lisboa, Portugal; 404 Centro Hospitalar do Médio Tejo, Abrantes, Portugal; 405 Inonu University, Intensive Care, Malatya, Turkey; 406 University of Valencia/School of Medicine, Valencia, Spain; 407 Hospital General Universitario de Castellon, Castellon, Spain; 408 Department of Anaesthesiology & Intensive Care, APHM, CHU Timone, Marseille, France; 409 University School of Medicine, NORT Laboratory, INSERM 1062 INRA 1260, Marseille, France; 410 APHM, CHU Timone, Laboratoire NORT INSERM U 1062 INRA U 1260, Marseille, France; 411 APHM, CHU Timone, Vascular Research Center of Marseille INSERM UMRS 1076, CERIMED, Marseille, France; 412 University of Milan - Bicocca, School of Medicine and Surgery, Milan, Italy; 413 Department of Emergency and Intensive Care, San Gerardo Hospital, Neurointensive Care, Monza, Italy; 414 Department of Otolaryngology, San Gerardo Hospital, Monza, Italy; 415 Hospital municipal Moyses Deutsch, ICU, Sao Paulo, Brazil; 416 Hospital Israelita Albert Einstein, ICU, Sao Paulo, Brazil; 417 Hospital Israelita Albert Einstein, Neurology/Telemedicine, Sao Paulo, Brazil; 418 Hospital Israelita Albert Einstein, Telemedicine, Sao Paulo, Brazil; 419 Hospital municipal Moyses Deutsch, Telemedicine, Sao Paulo, Brazil; 420 Radboud University Medical Center, Intensive Care, Nijmegen, Netherlands; 421 Radboud University Medical Center, Anesthesiology, Nijmegen, Netherlands; 422 Clinical Chemistry Department, University Hospital Virgen de la Victoria / IBIMA, Málaga, Spain; 423 Department of Intensive Care Unit, University Hospital Virgen de la Victoria / IBIMA, Málaga, Spain; 424 West China Hospital of Sichuan University, Chengdu, China; 425 West China Second Hospital of Sichuan University, Chengdu, China; 426 Department of Anaesthesiology, University Medical Center Hamburg-Eppendorf, Hamburg, Germany; 427 Department of Intensive Care Medicine, University Medical Center Hamburg-Eppendorf, Hamburg, Germany; 428 University Medical Center Hamburg-Eppendorf, Institute of Clinical Pharmacology and Toxicology, Hamburg, Germany; 429 Liverpool School of Tropical Medicine, Clinical Sciences, Liverpool, UK; 430 Aintree University Hospital NHS Foundation Trust, Critical Care, Liverpool, UK; 431 University of Liverpool, Institute of Ageing and Chronic Disease, Liverpool, UK; 432 Centers for Disease Control, Bacterial Infection, Atlanta, USA; 433 University of Liverpool, Institute of Infection & Global Health, Liverpool, UK; 434 Radboud University Medical Center, Intensive Care, Nijmegen, Netherlands; 435 Radboud University Medical Center, Virology, Nijmegen, Netherlands; 436 NIZO, Ede, Netherlands; 437 Radboud University Medical Center, Pediatrics, Nijmegen, Netherlands; 438 Fundació Parc Taulí, Sabadell, Spain; 439 CIBERES, Grup 33, Sabadell, Spain; 440 Corporació Sanitària i Universitaria Parc Taulí, Critical Care Center, Sabadell, Spain; 441 Kyung Hee University/Kyung Hee Medical Center, Pulmonary and Critical Care Medicine, Seoul, Republic of Korea; 442 Division of Pulmonary, Allergy and Critical Care Medicine, Department of Internal Medicine, Kangdong Sacred Heart Hospital, Seoul, Republic of Korea; 443 Department of Pharmacology, College of Medicine, Hallym University, Chuncheon, Republic of Korea; 444 Kyung Hee University/Kyung Hee University Hospital at Gangdong, Seoul, Republic of Korea; 445 University of Ulsan College of Medicine/ Asan Medical Center, Seoul, Republic of Korea; 446 Department of Physiology College of Medicine, Kyungpook National University, Daegue, Republic of Korea; 447 Sherbrooke, ICU/Medicine, Sherbrooke, Canada; 448 Sherbrooke, Pharmacology/Physiology, Sherbrooke, Canada; 449 IPS Therapeutic Inc, Sherbrooke, Canada; 450 Division of Medicine, Bloomsbury Institute of Intensive Care Medicine, University College London, London, UK; 451 Bloomsbury Institute of Intensive Care Medicine, University College London, London, UK; 452 Magnus Oxygen Ltd, London, UK; 453 University College London, Faculty of Medical Sciences, London, UK; 454 Lille Univ Hospital, Lille, France; 455 Marmara University, Anesthesiology and Reanimation, Istanbul, Turkey; 456 Marmara University, Pediatric Immunology and Alergy, Istanbul, Turkey; 457 Tubitak Arastırma Merkezi, Gen Engineering, Istanbul, Turkey; 458 Marmara University, Pathology, Istanbul, Turkey; 459 UCL, Bloomsbury Institute of Intensive Care Medicine, London, UK; 460 Barts Health NHS Trust, London, UK; 461 The Bizard Institute, Barts & the London School of Medicine & Dentistry, London, UK; 462 The William Harvey Research Institute, Barts & the London School of Medicine & Dentistry, London, UK; 463 Yonsei University College of Medicine, Anesthesiology, Seoul, Republic of Korea; 464 National Yang-Ming University, Institute of Emergency and Critical Care Medicine, Taipei, Taiwan, Province of China; 465 Department of Surgery, National Taiwan University Hospital, Taipei, Taiwan, Province of China; 466 Department of Nursing, National Taiwan University Hospital, Taipei, Taiwan, Province of China; 467 Department of Anesthesiology, National Taiwan University Hospital, Taipei, Taiwan, Province of China; 468 Department of Surgery, Kaohsiung Veterans General Hospital, Kaohsiung, Taiwan, Province of China; 469 Department of Intensive Care, Bravis Hospital, Roosendaal, Netherlands; 470 Department of Intensive Care, Erasmus Medical Center, Rotterdam, Netherlands; 471 Department of Pediatric Surgery, Intensive Care Unit, Erasmus MC-Sophia Children’s Hospital, University Medical Center, Rotterdam, Netherlands; 472 Department of Public Health, Erasmus Medical Centre, Rotterdam, Netherlands; 473 Marketing Intelligence Department, Zilveren Kruis, Achmea B.V, Leiden, Netherlands; 474 Department of Intensive Care, Albert Schweitzer Hospital, Dordrecht, Netherlands; 475 Department of Intensive Care, Ikazia Hospital, Rotterdam, Netherlands; 476 Department of Intensive Care, IJsselland Hospital, Rotterdam, Netherlands; 477 Department of Intensive Care, Sint Franciscus Gasthuis, Rotterdam, Netherlands; 478 Department of Intensive Care, Maasstad Hospital, Rotterdam, Netherlands; 479 University Medical Center Hamburg-Eppendorf, Center of Anesthesiology and Intensive Care Medicine, Hamburg, Germany; 480 University Medical Center Hamburg-Eppendorf, Institute of Medical Biometry and Epidemiology, Hamburg, Germany; 481 University Hospital Clermont-Ferrand, ICU, Clermont-Ferrand, France; 482 Saint-Louis Hospital, APHP, Burn ICU, Paris, France; 483 University Hospital Clermont-Ferrand, Pharmacy, Clermont-Ferrand, France; 484 University Hospital Clermont-Ferrand, Biostatistics, Clermont-Ferrand, France; 485 Odense University Hospital, University of Southern Denmark, Anaesthesiology and Intensive Therapy, Odense, Denmark; 486 Danish Center for Sleep Medicine, University of Copenhagen, Copenhagen, Denmark; 487 University of Southern Denmark, Vejle, Denmark; 488 University of Southern Denmark, Odense, Denmark; 489 University of Southern Denmark, Odense, Denmark; 490 Lillebaelt Hospital, Kolding, Denmark; 491 Odense University Hospital, Odense, Denmark; 492 Sheikh Khalifa Specialty Hospital, Anesthesiology, Rak, United Arab Emirates; 493 Department of Pulmonology, Sheikh Khalifa Specialty Hospital, Rak, United Arab Emirates; 494 Seoul National University Bundang Hospital, Seongnam, Republic of Korea; 495 Department of Pharmacy, National University of Singapore, Singapore, Singapore; 496 Department of Pharmacy, National University Hospital, Singapore, Singapore; 497 Department of Anesthesiology and Intensive Care, Danderyd Hospital, Stockholm, Sweden; 498 Department of Anesthesiology and Intensive Care, Norrtälje Hospital, Norrtälje, Sweden; 499 Department of Clinical Science and Education, Karolinska Institute, Södersjukhuset, Stockholm, Sweden; 500 Department of Anesthesiology and Intensive Care, Karolinska University Hospital, Stockholm, Sweden; 501 Department of Anesthesiology and Intensive Care, Örebro University Hospital, Örebro, Sweden; 502 Orion Pharma, Stockholm, Sweden; 503 Orion Pharma, Espoo, Finland; 504 Gelre Hospitals Apeldoorn, Intensive Care, Apeldoorn, Netherlands; 505 University of Toronto, Toronto, Canada; 506 University Belfast, Belfast, UK; 507 Clinc of Intensive Care, Malmo, Sweden; 508 Queens University Belfast, Belfast, UK; 509 University of Copenhagen, Copenhagen, Denmark; 510 University of Agdar, Grimstad, Norway; 511 Univeristy of Agdar, Grimstad, Norway; 512 Division of Intensive Care, Cyprus, Cyprus; 513 University of Athens, Athens, Greece; 514 University Cattolica del Sacro Cuore, Turino, Italy; 515 Division of Intensive Care, City unknown, Germany; 516 University Warminsko-Mazwski, Olsztyn, Poland; 517 Anesthesiology and Critical Care Research Center, Shiraz University of Medical Sciences, Shiraz, Islamic Republic of Iran; 518 Department of Drug Abuse Control, Shiraz University of Medical Sciences, Shiraz, Islamic Republic of Iran; 519 Trauma Research Center, Shiraz University of Medical Sciences, Shiraz, Islamic Republic of Iran; 520 Department of Neurosurgery, Shiraz University of Medical Sciences, Shiraz, Islamic Republic of Iran; 521 Department of Critical Care Nursing, Jahrom University of Medical Sciences, Jahrom, Islamic Republic of Iran; 522 Department of Neurology, Shiraz University Of Medical Sciences, Shiraz, Islamic Republic of Iran; 523 Nemazee Hospital, Shiraz University of Medical Sciences, Shiraz, Islamic Republic of Iran; 524 Department of Clinical Pharmacy, Shiraz University of Medical Sciences, Shiraz, Islamic Republic of Iran; 525 Department of Surgery, Shiraz University of Medical Sciences, Shiraz, Islamic Republic of Iran; 526 Karolinska University Hospital, Stockholm, Sweden; 527 University of Oslo, Oslo, Norway; 528 Mitt Universitetet, Östersund, Sweden; 529 Poznan Unversity of Medical Sciences, Poznan, Poland; 530 Medical University of Gdansk, Gdansk, Poland; 531 AOU Maggiore della Carità, Novara, Italy; 532 Azienda Ospedaliero-Universitaria Careggi, Firenze, Italy; 533 Azienda Ospedaliero-Universitaria Careggi, Novara, Italy; 534 Belfast Health and Social Care Trust, Critical Care, Belfast, UK; 535 Queen’s University of Belfast, Centre for Experimental Medicine, Belfast, UK; 536 Southern Health and Social Care Trust, Anaesthetics and Intensive Care, Craigavon, UK; 537 University of Toronto, Critical Care Medicine, Toronto, Canada; 538 Instituto Mexicano del Seguro Social, Intensive Care Unit, Leon, Mexico; 539 Instituto Mexicano del Seguro Social, Radiology Unit, Leon, Mexico; 540 Department for Intensive Care Medicine, Imland Klinik Rendsburg, Rendsburg, Germany; 541 Department for Thoracic and Cardiovascular Surgery, University Hospital Essen, Essen, Germany; 542 Department of Anaesthesiology and Intensive Care Medicine, University Witten/Herdecke, Medical Centre Cologne-Merheim, Cologne, Germany; 543 Policlinico Universitario A. Gemelli, Anesthesia and Intensive Care Medicine, Roma, Italy; 544 St George’s University Hospital, London, UK; 545 Università La Sapienza di Roma, Rome, Italy; 546 Università degli Studi di Parma, Parma, Italy; 547 Hôpital de Bicêtre, Hôpitaux Universitaires Paris-Sud, Université Paris-Sud, Service de Réanimation Médicale, Inserm UMR_S999, Le Kremlin Bicêtre, France; 548 Department of Anesthesiology and Intensive Therapy, University of Szeged, Szeged, Hungary; 549 University of Szeged, Institute of Surgical Research, Szeged, Hungary; 550 Department of Anaesthesiology and General Intensive Care Medicine, Medical University of Vienna, Vienna, Austria; 551 Department of Anesthesia and Critical Care Medicine, Cairo University, Cairo, Egypt; 552 University of la Reunion, Intensive Care Unit, Saint Pierre de la Reunion, France; 553 University of la Reunion, Neurocritical Care Unit, Saint Pierre de la Reunion, France; 554 Anesthesiology Department, University of la Reunion, Saint Pierre de la Reunion, France; 555 Departamento de Medicina Intensiva, Pontificia Universidad Catolica de Chile, Facultad de Medicina, Santiago, Chile; 556 Pontificia Universidad Catolica de Chile, Programa de Medicina de Urgencia, Facultad de Medicina, Santiago, Chile; 557 Technische Universität München, II. Medizinische Klinik, Munich, Germany; 558 Klinikum Köln-Merheim, Cologne, Germany; 559 Asklepios Klinik St. Georg, Hamburg, Ghana; 560 Université Libre de Bruxelles, Brussels, Belgium; 561 Univ. Hospital of Amiens, Amiens, France; 562 CH Beauvais, Beauvais, France; 563 Cairo University, Cairo, Egypt; 564 Kasr Alainy Medical School, Cairo University, Cairo, Egypt; 565 ABC Medical Center, Critical Medicine, Mexico City, Mexico; 566 Edwards Lifesciences, Critical Care, Irvine, USA; 567 University of California Irvine, Biomedical Engineering, Irvine, USA; 568 University of California Los Angeles, Anesthesiology, Los Angeles, USA; 569 Ramathibodi Hospital, Mahidol University, Medicine, Bangkok, Thailand; 570 Santa Maria delle Croci Hospital, Anesthesia and Intensive Care, Ravenna, Italy; 571 King Hamad University Hospital, Intensive Care, Muharraq, Bahrain; 572 Kings College Hospital, London, UK; 573 Defence Science and Technology Laboratory, Salisbury, UK; 574 NIHR Surgical Reconstruction and Microbiology Research Centre, Birmingham, UK; 575 Edwards Lifesciences, Irvine, USA; 576 Edwards Lifesciences, Amsterdam, Netherlands; 577 Department of Critical Care Medicine, University of Pittsburgh, Pittsburgh, USA; 578 Department of Anaesthesia and Critical Care Medicine, Cardiothoracic and Vascular Anaesthesia, University Hospital of Pisa, Pisa, Italy; 579 University Medical Center Hamburg-Eppendorf, Hamburg, Germany; 580 Klinikum Harlaching, München, Germany; 581 Dalhousie University, Halifax, Canada; 582 Cairo University, Anaesthesia, Cairo, Egypt; 583 Cairo University, Cairo, Egypt; 584 University Hospitals of Leicester NHS Trust, Anaesthesia, Critical Care and Pain, Leicester, UK; 585 University of Leicester, Leicester, UK; 586 University Hospitals of Leicester NHS Trust, Emergency Medicine Academic Group, Leicester, UK; 587 Ysbyty Glan Clwyd, Critical Care, Bodelwyddan, UK; 588 Critical Care Department, Faculty of Medicine, Cairo University, Cairo, Egypt; 589 Critical Care Department, Cairo University, Cairo, Egypt; 590 Department of Critical Care Medicine, Kaohsiung Veterans General Hospital, Kaohsiung City, Taiwan, Province of China; 591 Cardiovascular Division, Kaohsiung Veterans General Hospital, Kaohsiung City, Taiwan, Province of China; 592 Hospital Universitario V. de Valme, Medicina Intensiva, Sevilla, Spain; 593 Hospital Universitario V. de Valme, Sevilla, Spain; 594 Complexo Hospitalario Universitario de Santiago de Compostela, Intensive Care, Santiago de Compostela, Spain; 595 Complexo Hospitalario Universitario de Santiago de Compostela, Cardiology, Santiago de Compostela, Spain; 596 Department of Intensive Care Unit, University Hospital Virgen de la Victoria / IBIMA, Málaga, Spain; 597 Kings College Hospital, Intensive Care Unit, London, UK; 598 Anesthesia and Critical Care Medicine, Cardiothoracic Department ‘A. De Gasperis’, ASST Grande Ospedale Metropolitano Niguarda, Milan, Italy; 599 Department of Intensive Care Unit, University Hospital Virgen de la Victoria / IBIMA, Málaga, Spain; 600 Complexo Hospitalario Universitario de Santiago de Compostela, Intensive Care, Santiago de Compostela, Spain; 601 Complexo Hospitalario Universitario de Santiago de Compostela, Cardiology, Santiago de Compostela, Spain; 602 Farhat Hached University Hospital, Medical Intensive Care Unit, Sousse, Tunisia; 603 Cardiology Department, Farhat Hached University Hospital, Sousse, Tunisia; 604 Ibn Al Jazzar Faculty of Medicine, University of Sousse, Research Laboratory N° LR14ES05. Interactions of the Cardiopulmonary System, Sousse, Tunisia; 605 Makati Medical Center, Internal Medicine, Makati, Philippines; 606 University College London, Bloomsbury Institute of Intensive Care Medicine, London, UK; 607 Department of Clinical and Experimental Medicine, University of Messina, Messina, Italy; 608 Magnus Oxygen Ltd, London, UK; 609 University College London, Bloomsbury Institute of Intensive Care Medicine, London, UK; 610 Dipartimento di Fisiopatologia Medico-Chirurgica e dei Trapianti, Università degli Studi di Milano, Milan, Italy; 611 Magnus Oxygen Ltd, London, UK; 612 Emergency Department, Taipei Medical University Hospital, Taipei, Taiwan, Province of China; 613 Taipei Medical University, Taipei, Taiwan, Province of China; 614 Harvard Medical School, Boston, USA; 615 Brigham and Women’s Hospital, Boston, USA; 616 Critical Care Division, Kaohsiung Veterans General Hospital, Kaohsiung City, Taiwan, Province of China; 617 Cardiovascular Division, Kaohsiung Veterans General Hospital, Kaohsiung City, Taiwan, Province of China; 618 Kaohsiung City Government, Fire Bureau, Kaohsiung City, Taiwan, Province of China; 619 Department of Health, Kaohsiung City Government, Kaohsiung City, Taiwan, Province of China; 620 New Cross Hospital, Integrated Critical Care Unit, Wolverhamptom, UK; 621 New Cross Hospital, Critical Care Services, Research and Audit Office, Wolverhamptom, UK; 622 Central Manchester Foundation NHS Trust, Adult Critical Care, Manchester, UK; 623 az Sint-Blasius, Dendermonde, Belgium; 624 Alexandria University, Critical Care Medicine, Alexandria, Egypt; 625 Karolinska University Hospital, CIVA, Stockholm, Sweden; 626 Oslo University Hospital, General Intensive Care Unit - Ullevål, Oslo, Norway; 627 Department of Anaesthesiology, Oslo University Hospital, Oslo, Norway; 628 Oslo University Hospital, General Intensive Care Unit 1 -Rikshospitalet, Oslo, Norway; 629 Haukeland University Hospital, General Intensive Care Unit, Bergen, Norway; 630 Hospital Insular Las Palmas GC, Las Palmas de Gran Canaria, Spain; 631 Department of Economy. University of Las Palmas de Gran Canaria, Hospital Insular Las Palmas GC, Las Palmas de Gran Canaria, Spain; 632 University of Algarve, Departmant of Biomedical Sciences and Medicine, Faro, Portugal; 633 Emergency and Intensive Care Departmant, Centro Hospitalar do Algarve, Hospital de Faro, Faro, Portugal; 634 Centro Hospitalar do Algarve, Hospital de Faro, Faro, Portugal; 635 Faculty of Medicine of Porto, CINTESIS, Porto, Portugal; 636 Vilnius University, Clinic of Anaesthesiology and Intensive care, Vilnius, Lithuania; 637 Vilnius University, Faculty of Medicine, Vilnius, Lithuania; 638 Lithuanian University of Health Sciences, Clinic of Anaesthesiology, Kaunas, Lithuania; 639 Department of Clinical Science and Education, Karolinska Institutet, Södersjukhuset, Stockholm, Sweden; 640 Sophiahemmet University, Stockholm, Sweden; 641 Linnaeus University, Kalmar/Växjö, Sweden; 642 Western Parana State University Hospital, Cascavel, Brazil; 643 Western Parana State University Hospital, Cascavel, Brazil; 644 Royal Berkshire Hospital, Intensive Care Unit, Reading, UK; 645 University of Turin, Anesthesia and Intensive Care, Turin, Italy; 646 University of Turin, Psychology, Torino, Italy; 647 Sapienza University of Rome, Anesthesia and Intensive Care, Rome, Italy; 648 Sapienza University of Rome, Scienze e Biotecnologie Medico Chirurgiche, Rome, Italy; 649 Queen Elizabeth University Hospital, NHS GG&C, Intensive Care Medicine, Glasgow, UK; 650 Hospital del Mar/CIBERES/UPF, Intensive Care, Barcelona, Spain; 651 Vall d’Hebrón University Hospital, Intensive Care, Barcelona, Spain; 652 Anesthesia and Intensive Care, Fondazione IRCCS Policlinico S. Matteo, University of Pavia, Pavia, Italy; 653 Blkapoor Super Speciality Hospital, BLK Centre for Critical Care, New Delhi, India; 654 University Medical Center Utrecht, Intensive Care, Utrecht, Netherlands; 655 Academic Medical Center, University of Amsterdam, Intensive Care, Amsterdam, Netherlands; 656 Dutch National Intensive Care Evaluation Foundation, Amsterdam, Netherlands; 657 Hospital Virgen de la Arrixaca, ICU, El Palmar, Spain; 658 Mater Misericordiae University Hospital, ICU, Dublin, Ireland; 659 Mater Misericordiae University Hospital, Dublin, Ireland; 660 Mater Misericordiae University Hospital, Microbiology, Dublin, Ireland; 661 Chi Mei Medical Center, Internal Medicine, Tainan, Taiwan, Province of China; 662 Critical Care Medicine Department, Kasr Alainy Medical School, Cairo University, Cairo, Egypt; 663 Department of Intensive Care 4131, Copenhagen University Hospital, Copenhagen, Denmark; 664 Department of Anaesthesiology, Surgical Services and Intensive Care Medicine, Karolinska University Hospital, Stockholm, Sweden; 665 Department of Intensive Care Medicine, Hospital de Navarra, Pamplona, Spain; 666 Maquet Critical Care AB, Solna, Sweden; 667 Department of Surgical Sciences, Uppsala University, Hedenstierna Laboratory, Uppsala, Sweden; 668 Dipartimento di Fisiopatologia Medico Chirurgica e dei Trapianti, Università degli Studi di Milano, Milano, Italy; 669 Dipartimento di Anestesia, Rianimazione, Urgenza ed Emergenza, Policlinico Di Milano, Milano, Italy; 670 Plug Working Group, Milan, Italy; 671 Dipartimento di Fisiopatologia Medico-Chirurgica e dei Trapianti, Università degli Studi di Milano, Milano, Italy; 672 Georg-August-University Goettingen, Anesthesiology and Intensive Care Medicine, Goettingen, Germany; 673 Department of Intensive Care Medicine, University of Zagreb, Rebro, Croatia; 674 Critical Care Department, Kasr Al-Aini Hospitals, Cairo University, Cairo, Egypt; 675 Physiotherapy Department, Cairo University, Cairo, Egypt; 676 Academic Medical Center, Amsterdam, Netherlands; 677 University Medical Center Utrecht, Utrecht, Netherlands; 678 University of Foggia, Foggia, Italy; 679 Technische Universität München, II. Medizinische Klinik, Munich, Germany; 680 Städtisches Klinikum München Harlaching, Munich, Germany; 681 Technische Universität München, I. Medizinische Klinik, Munich, Germany; 682 Krankenhaus Barmherzige Brüder, Munich, Germany; 683 Mayo Clinic, Jacksonville, FL USA; 684 Mayo Clinic, Rochester, NY USA; 685 Stanford University, Stanford, CA USA; 686 Khoo Teck Puat Hospital, Singapore, Singapore; 687 Department of Critical Care, Whiston Hospital, Liverpool, UK; 688 Chang Gung University, School of Nursing, College of Medicine, Taoyuan, Taiwan, Province of China; 689 Division of Thoracic Medicine, Chang Gung Memorial Hospital, Taoyuan, Taiwan, Province of China; 690 Department of Nursing, Taipei Tzu Chi Hospital, Buddhist Tzu Chi Medical Foundation, New Taipei City, Taiwan, Province of China; 691 Department of Respiratory Therapy, Chang Gung Memorial Hospital, Taoyuan, Taiwan, Province of China; 692 Emergency Department, Azienda Ospedaliera di Padova, Padova, Italy; 693 Medicine Department, University of Padova, Padova, Italy; 694 Toronto Western Hospital MSNICU, Toronto, Canada; 695 Division of Thoracic Medicine, Chang Gung Memorial Hospital, Taoyuan, Taiwan, Province of China; 696 Hospital Universitario Príncipe de Asturias, Intensive Care Unit, Madrid, Spain; 697 Hospital Universitario Príncipe de Asturias, Pneumology, Madrid, Spain; 698 Pulmonology Division, Hospital das Clínicas da Faculdade de Medicina da Universidade de São Paulo, Heart Institute, São Paulo, Brazil; 699 Cardiology Division, Hospital das Clínicas da Faculdade de Medicina da Universidade de São Paulo, Heart Institute, São Paulo, Brazil; 700 Anesthesiology Division, Hospital das Clínicas da Faculdade de Medicina da Universidade de São Paulo, Heart Institute, São Paulo, Brazil; 701 Hospices Civils de Lyon, Hôpital de la Croix-Rousse - Service de réanimation médicale, Lyon, France; 702 CNRS UMR 5220 - INSERM U1206, CREATIS, Lyon, France; 703 CERMEP, Bron, France; 704 Hospices Civils de Lyon, Hôpital de la Croix-Rousse - Service d’anatomopathologie, Lyon, France; 705 Université de Lyon, Université Lyon I, Lyon, France; 706 Dipartimento di Fisiopatologia Medico-Chirurgica e dei Trapianti, Università degli Studi di Milano, Milano, Italy; 707 Georg-August-University Goettingen, Anesthesiology and Intensive Care Medicine, Goettingen, Germany; 708 Dipartimento di Anestesia, Rianimazione, Urgenza ed Emergenza, Policlinico Di Milano, Milano, Italy; 709 Plug Working Group, Milan, Italy; 710 Department of Critical Care Medicine, Gazi University Faculty of Medicine, Ankara, Turkey; 711 Department of Geriatrics, Gazi University Faculty of Medicine, Ankara, Turkey; 712 Critical Care Department, Vall d’Hebron University Hospital, Barcelona, Spain; 713 Vall d’Hebron Research Institut, Barcelona, Spain; 714 CIBERES, Instituto de Salud Carlos III, Madrid, Spain; 715 Vall d’Hebrón University Hospital, Pneumology, Barcelona, Spain; 716 Vall d’Hebrón University Hospital, Thoracic Surgery, Barcelona, Spain; 717 Department of Medicine, Division of Pulmonary and Critical Care Medicine, University of Texas Health Science Center at San Antonio, San Antonio, TX USA; 718 Department of Circulation and Medical Imaging, Norwegian University of Science and Technology, Trondheim, Norway; 719 Department of Circulation and Medical Imaging, Norwegian University of Science and Technology, Trondheim, Norway; 720 Department of Cancer Research and Molecular Medicine, Norwegian University of Science and Technology, Centre of Molecular Inflammation Research, Trondheim, Norway; 721 Department of Cancer Research and Molecular Medicine, Norwegian University of Science and Technology, Trondheim, Norway; 722 Department of Chronic Disease Epidemiology, Yale University School of Public Health, New Haven, CT USA; 723 Department of Public Health and General Practice, Norwegian University of Science and Technology, Trondheim, Norway; 724 Norwegian University of Science and Technology, Centre of Molecular Inflammation Research, Trondheim, Norway; 725 Department of Circulation and Medical Imaging, Norwegian University of Science and Technology, Trondheim, Norway; 726 St Olavs University Hospital, Clinic of Anaesthesia and Intensive Care, Trondheim, Norway; 727 Norwegian University of Science and Technology, Institute of Cancer Research and Molecular Medicine, Trondheim, Norway; 728 Department of Medicine, Nord Trøndelag Hospital Trust, Levanger Hospital, Levanger, Norway; 729 Yale University School of Public Health, New Haven, CT USA; 730 Department of Infectious Diseases, St Olavs University Hospital, Trondheim, Norway; 731 Department of Public Health and General Practice, Norwegian University of Science and Technology, Trondheim, Norway; 732 Department of Endocrinology, St Olavs University Hospital, Trondheim, Norway; 733 Hacettepe University, Ankara, Turkey; 734 Erasme, Brussels, Belgium; 735 Erasme Hospital, ICU, Brussels, Belgium; 736 The First Affiliated Hospital of Sun Yat-Sen University, Guangzhou, China; 737 Attikon University Hospital, ICU, Athens, Greece; 738 Department of Internal Medicine, Chungnam National University Hospital, Daejeon, Republic of Korea; 739 Chungnam National University Hospital, Clinical Trials Center, Daejeon, Republic of Korea; 740 Department of Internal Medicine, Cheongju St. Mary’s Hospital, Cheongju, Republic of Korea; 741 Department of Internal Medicine, Chungbuk National University Hospital, Cheongju, Republic of Korea; 742 Department of Internal Medicine, Konyang University Hospital, Daejeon, Republic of Korea; 743 Department of Internal Medicine, Konkuk University Chungju Hospital, Chungju, Republic of Korea; 744 Royal Surrey County Hospital, ICU and SPACeR Research Group, Guildford, UK; 745 University of Surrey, Guildford, UK; 746 University of Semmelweis, Budapest, Hungary; 747 Royal Surrey County Hospital, Guildford, UK; 748 Department of Internal Medicine, Ankara University Faculty of Medicine, Intensive Care, Ankara, Turkey; 749 Department of Anesthesiology and Reanimation, Yıldırım Beyazıt University, Medical Faculty, Ankara, Turkey; 750 Department of Biostatistics and Medical Informatics, Yıldırım Beyazıt University, Ankara, Turkey; 751 Department of Biochemistry, Yıldırım Beyazıt University, Medical Faculty, Ankara, Turkey; 752 Karolinska University Hospital / Karolinska Institutet, Stockholm, Sweden; 753 Mount Sinai Hospital, Toronto, Canada; 754 St Georges NHS Foundation Trust, Critical Care Directorate, London, UK; 755 The First Affiliated Hospital of Sun Yat-Sen University, Guangzhou, China; 756 Hospital San Juan de Dios del Aljarafe, Intensive Care Unit, Bormujos, Sevilla Spain; 757 Department of Intensive Care, Copenhagen University Hospital - Rigshospitalet, Copenhagen, Denmark; 758 Copenhagen University Hospital - Rigshospitalet, Center of Inflammation and Metabolism, Copenhagen, Denmark; 759 Copenhagen University Hospital - Rigshospitalet, Centre for Clinical Intervention Research - Copenhagen Trial Unit, Copenhagen, Denmark; 760 Department of Emergency Medicine, Kurashiki Central Hospital, Kurashiki, Japan; 761 Department of General Medicine, Kurashiki Central Hospital, Kurashiki, Japan; 762 Cairo University/Kasr Alainy Medical School, Anethesia, Cairo, Egypt; 763 Intensive Care Department, King Hamad University Hospital, Muharraq, Bahrain; 764 Cairo University, Critical Care, Cairo, Egypt; 765 Federal University of São Paulo, Surgery, São Paulo, Brazil; 766 Federal University of São Paulo, Morphology and Genetics, São Paulo, Brazil; 767 Department of Intensive Care Medicine, Fundación Valle del Lili - Universidad ICESI, Cali, Colombia; 768 Departamento de Medicina Intensiva, Pontificia Universidad Católica de Chile, Facultad de Medicina, Santiago, Chile; 769 Department of Intensive Care Medicine, CHIREC Hospitals, Université Libre de Bruxelles (ULB), Brussels, Belgium; 770 Erasme University Hospital, Université Libre de Bruxelles, Intensive Care, Brussels, Belgium; 771 University of Foggia, Anaesthesia and Intensive Care, Foggia, Italy; 772 Joan XXIII University Hospital, Tarragona, Spain; 773 Cairo University, Cairo, Egypt; 774 Cairo University Medical School, Cairo, Egypt; 775 SSA UADY, Mérida, Mexico; 776 Division on Emergencies and Critical Care, Oslo University Hospital, Oslo, Norway; 777 Divison of Cancer, Oslo University Hospital, Oslo, Norway; 778 Division of Medicine, Oslo University Hospital, Oslo, Norway; 779 Hospital Clinico UC - CHRISTUS, Unidad de Paciente Critico, Santiago, Chile; 780 Universidad de los Andes, Santiago, Chile; 781 Tal Hashomer Hospital, General ICU, Umm el Fahem, Israel; 782 Tel Aviv University, Ramat Gan, Israel; 783 Ghent University, Faculty of Medicine and Health Sciences, Ghent, Belgium; 784 Department of Internal Medicine, Ghent University, Ghent, Belgium; 785 Heart Institute (InCor) do Hospital das Clínicas da Faculdades de Medicina da Universidade de São Paulo, Nursing Coordination, São Paulo, Brazil; 786 Instituto Israelita de Ensino e Pesquisa Albert Einstein, School of Nursing, São Paulo, Brazil; 787 Nursing School of University of São Paulo, São Paulo, Brazil; 788 Hypertension Department, Heart Institute (InCor) do Hospital das Clínicas da Faculdades de Medicina da Universidade de São Paulo, São Paulo, Brazil; 789 The Royal Brompton and Harefield NHS Foundation Trust, Critical Care, London, UK; 790 Frimley Health NHS Foundation Trust, ICU, Frimley, UK; 791 The Royal College of Nursing Critical Care and Inflight Nursing Forum, London, UK; 792 Northumbria University, Newcastle, UK; 793 UKCCNA, Manchester, UK; 794 BACCN, Newcastle, UK; 795 Gertrude’s Children’s Hospital, Nairobi, Kenya; 796 Rush University Medical Center, Chicago, USA; 797 Teaching Department of Emergency and Critical Care Medicine, University of Tunis El Manar, Zaghouan, Tunisia; 798 Teaching Department of Emergency and Critical Care Medicine, University of Tunis El Manar, Zaghouan, Tunisia; 799 University of Toronto, Toronto, Canada; 800 Sunnybrook Health Sciences Centre, Toronto, Canada; 801 London Health Sciences Centre, London, Canada; 802 Ottawa Hospital Research Institute, Ottawa, Canada; 803 Ottawa Hospital, Ottawa, Canada; 804 University Hospitals Birmingham NHS Foundation Trust, Physiotherapy, Birmingham, UK; 805 University Hospitals Birmingham NHS Foundation Trust, Birmingham, UK; 806 Department of Critical Care, University Medical Center Groningen, Groningen, Netherlands; 807 University of Groningen, Artificial Intelligence, Groningen, Netherlands; 808 University Medical Center Groningen, Hospital Pharmacy, Groningen, Netherlands; 809 ABC Medical Center, Intensive Care Unit, Mexico City, Mexico; 810 St Georges NHS Foundation Trust, Cardiothoracic Intensive Care, London, UK; 811 St Georges NHS Trust, London, UK; 812 Nursing Department, Rovira i Virgili University, Tarragona, Spain; 813 Joan XXIII University Hospital, Tarragona, Spain; 814 Dr Josep Trueta University Hospital, Intensive Care Unit, Girona, Spain; 815 Hospital de Sant Joan Despí Moissès Broggi, Intensive Care Unit, Barcelona, Spain; 816 Quiron Salud-Hospital General de Catalunya, Intensive Care Unit, Barcelona, Spain; 817 Verge de la Cinta University Hospital, Intensive Care Unit, Tortosa, Spain; 818 Sant Pau I Santa Tecla University Hospital, Intensive Care Unit, Tarragona, Spain; 819 Joan XXIII University Hospital, Intensive Care Unit, Tarragona, Spain; 820 Verge de la Cinta University Hospital, Intensive Care Unit, Tarragona, Spain; 821 Vall d’Hebrón University Hospital, Barcelona, Spain; 822 Hospital Universitario de Gran Canaria Dr Negrin, Las Palmas de Gran Canaria, Spain; 823 Universidad de Las Palmas de Gran Canaria, Las Palmas de Gran Canaria, Spain; 824 N.N. Blokhin Russian Cancer Research Center, Medical ICU, Moscow, Russian Federation; 825 Department of Anaesthesiology and Critical Care Medicine, Aix Marseille University, CHU Timone, Marseille, France; 826 CHU Pitié-Salpêtrière, Neurosurgical Intensive Care Unit, Paris, France; 827 Aix-Marseille University / CNRS, Institut des Neurosciences de la Timone, UMR7289, Marseille, France; 828 Aix-Marseille University / CNRS, Laboratoire des Sciences de l’Information et des Systèmes, UMR7296, Marseille, France; 829 Poznan Unversity of Medical Sciences, Poznan, Poland; 830 Medical University of Gdansk, Gdansk, Poland; 831 Royal Brompton and Harefield NHS Foundation Trust, London, UK; 832 Cochin Hospital, Medical Intensive Care Unit, Paris, France; 833 Paris Descartes University, Paris, France; 834 Sudden Death Expertise Center, INSERM UMR1018, Paris, France; 835 Emergency Department, Cochin Hospital, Paris, France; 836 Intensive Care Unit of Melun Hospital, Melun, France; 837 Cardiology Department, Cochin Hospital, Paris, France; 838 Cardiology Department, Georges Pompidou European Hospital, Paris, France; 839 VU University Medical Center Amsterdam, Intensive Care, Amsterdam, Netherlands; 840 VU University Medical Center Amsterdam, Amsterdam, Netherlands; 841 Hospital Universitario Virgen del Rocío, Sevilla, Spain; 842 Department of Intensive Care Unit, University Hospital Virgen de la Victoria / IBIMA, Málaga, Spain; 843 Emergency and General Medicine, Kumamoto University Hospital, Kumamoto, Japan; 844 Division of Perioperative Services, Intensive Care Medicine and Pain Management, Turku University, Turku University Hospital, Turku, Finland; 845 Turku University, Turku University Hospital, Heart Center, Turku, Finland; 846 Division of Intensive Care Medicine, Department of Anaesthesiology, Intensive Care and Pain Medicine, Helsinki University, Helsinki University Hospital, Helsinki, Finland; 847 Department of Emergency Medicine and Services, Helsinki University, Helsinki University Hospital, Helsinki, Finland; 848 Department of Cardiology, Helsinki University, Helsinki University Hospital, Helsinki, Finland; 849 Division of Clinical Neurosciences, Turku University, Turku University Hospital, Turku, Finland; 850 Regional Hospital of Zaghouan, Zaghouan, Tunisia; 851 Catholic University of Korea, Seoul, Republic of Korea; 852 National Taiwan University Hospital, Anesthesiology, Taipei, Taiwan, Province of China; 853 Critical Care Department, Hospital 12 de Octubre, Trauma ICU, Madrid, Spain; 854 Bethesda, Maryland USA; 855 North Bristol NHS Trust, Anaesthetics and Intensive Care Medicine, Bristol, UK; 856 Academic Medical Center, University of Amsterdam, Intensive Care, Amsterdam, Netherlands; 857 Academic Medical Center, University of Amsterdam, Clinical Research Unit, Amsterdam, Netherlands; 858 Critical Care Department, Hospital Universitari Mutua Terrassa, Terrassa, Spain; 859 Hospital Universitari Mutua Terrassa, Terrassa, Spain; 860 Queen Alexandra Hospital, Critical Care, Portsmouth, UK; 861 Queen Elizabeth University Hospital, Anaesthetics and Intensive Care, Glasgow, UK; 862 Golden Jubilee National Hospital, Anaesthetics and Intensive Care, Glasgow, UK; 863 KAT Hospital, B ICU and Research Center, Kifisia, Greece; 864 Khoula Hospital, ICU, Muscat, Oman; 865 Carlos Haya Hospital, ICU, Malága, Spain; 866 Carlos Haya Hospital, Málaga, Spain; 867 University Medical Center Hamburg-Eppendorf, Anesthesiology, Hamburg, Germany; 868 University Medical Center Hamburg-Eppendorf, Internal Medicine, Hamburg, Germany; 869 University Medical Center Hamburg-Eppendorf, Hepatobiliary Surgery and Visceral Transplantation, Hamburg, Germany; 870 University of Pavia, Anaesthesia, Intensive Care and Pain Therapy, Pavia, Italy; 871 Intensive Care and Emergency Department, Fondazione Policlinico San Matteo, IRCCS, Anaesthesia, Pavia, Italy; 872 Cardiology Department, Universitat Autònoma de Barcelona, Barcelona, Spain; 873 Cardiology Department, Royal Brompton and Harefield NHS Foundation Trust, London, UK; 874 Royal Brompton and Harefield NHS Foundation Trust, London, UK; 875 Anesthesia and Intensive Care Department, Shiraz University of Medical Sciences, Shiraz, Islamic Republic of Iran; 876 Anesthesia and Intensive Care Department, Anesthesiology and Critical Care Research Center, Shiraz, Islamic Republic of Iran; 877 Shiraz University of Medical Sciences, Shiraz, Islamic Republic of Iran; 878 Shiraz University of Medical Sciences, Radiology, Shiraz, Islamic Republic of Iran; 879 University of Modena, Bologna, Italy; 880 University of Modena, Modena, Italy; 881 Klinikum Kassel, Cardiology and Intensive Care, Kassel, Germany; 882 Department of Anesthesiology, University Medical Center Hamburg-Eppendorf, Hamburg, Germany; 883 Vienna University of Technology, Institute of Electrodynamics, Microwave and Circuit Engineering, Vienna, Austria; 884 Swisstom AG, Landquart, Switzerland; 885 Department of Anaesthesiology, Surgical Services and Intensive Care Medicine, Karolinska University Hospital, Stockholm, Sweden; 886 Department of Physiology and Pharmacology, Karolinska Institute, Stockholm, Sweden; 887 Department of Intensive Care Medicine, Hospital de Navarra, Pamplona, Spain; 888 Maquet Critical Care, Stockholm, Sweden; 889 Department of Surgical Sciences, Hedenstierna Laboratory, Uppsala, Sweden; 890 Hospital Virgen de la Salud, SESCAM, Intensive Care Medicine, Toledo, Spain; 891 Hospital Quironsalud Palmaplanas, Intensive Care Medicine, Palma de Mallorca, Spain; 892 Hospital Marina Salud Denia, Intensive Care Medicine, Murcia, Spain; 893 Hospital Virgen de la Salud, SESCAM, Research Unit, Medical Council, Toledo, Spain; 894 King Hamad University Hospital, Intensive Care, Muharaq, Bahrain; 895 King Hamad University Hospital, Muharaq, Bahrain; 896 Department of Intensive Care, Hopital Erasme, Université Libre de Bruxelles, Brussels, Belgium; 897 Department of Medical Biotechnologies, University of Siena, Anesthesia and Intensive Care Unit, Siena, Italy; 898 N.V. Sklifosovsky Research Institute of Emergency Medicine of the Moscow Healthcare Department, Moscow, Russian Federation; 899 Kocaeli Derince Training Hospital, Intensive Care, Kocaeli, Turkey; 900 Kocaeli Derince Education and Research Hospital, Kocaeli, Turkey; 901 Facultad de Medicina Ciudad Real, Hospital General Universitario de Ciudad Real, Anestesiologia y Reanimacion, Ciudad Real, Spain; 902 Facultad de Medicina Ciudad Real, Hospital General Universitario Ciudad Real, Cuidados Criticos Pediatricos, Ciudad Real, Spain; 903 Hospital General Universitario Ciudad Real, Anestesiologia y Reanimacion, Ciudad Real, Spain; 904 Facultad de Medicina Ciudad Real, Hospital General Universitario Ciudad Real, Cirugía Hepatobiliar, Ciudad Real, Spain; 905 Baylor College of Medicine, Pediatric Critical Care, Houston, TX USA; 906 Baylor College of Medicine, Pediatric Nephrology, Houston, TX USA; 907 SSA UADY, Mérida, Mexico; 908 Hospital U.V. Rocío, Seville, Spain; 909 Hospital General Universitario de Castellon, Castellon, Spain; 910 H Carlos Haya, Malaga, Spain; 911 Hospital de Sagunto, Valencia, Spain; 912 Complejo Hospitalario de Navarra, Pamplona, Spain; 913 Instituto Nacional de Ciencias Médicas y Nutrición Salvador Zubirán, Critical Care, México, Mexico; 914 Hospital Universitari de Bellvitge, Critical Care, L’Hospitalet, Spain; 915 Instituto de Investigacion Biomédica de Bellvitge, L’Hospitalet, Spain; 916 Hospital de la Santa Creu i Sant Pau, Institut de Recerca Cardiovascular, Barcelona, Spain; 917 Hospital de la Santa Creu i Sant Pau, Critical Care, Barcelona, Spain; 918 Department of Medicine, Division of Nephrology, Chulalongkorn University, Bangkok, Thailand; 919 Department of Biochemistry, Chulalongkorn University, Bangkok, Thailand; 920 University Hospital Leipzig, Anesthesia and Intensive Care, Leipzig, Germany; 921 University Hospital Leipzig, Neurosurgery, Leipzig, Germany; 922 Hospital Universitario La Paz, Medicina Intensiva, Madrid, Spain; 923 Academic Medical Center, University of Amsterdam, Translational Physiology, Amsterdam, Netherlands; 924 Erasmus Medical Center, Experimental Anesthesiology, Roterdam, Netherlands; 925 1st Medical Faculty, Charles University, Prague, Czech Republic; 931 Hopital Bichat Claude Bernard, Anaesthesiology and Surgical Critical Care, Paris, France; 932 Centre Hospitalier Sud Francilien, Critical Care Medecine, Corbeil-Essonnes, France; 933 VU University Medical Center, Intensive Care, Amsterdam, Netherlands; 934 University Hospital Basel, Anaesthesia & Intensive Care: Surgical Intensive Care, Basel, Switzerland; 935 University Hospital Basel, Nephrology & Transplant Immunology, Basel, Switzerland; 936 Department of Clinical Research, University Hospital Basel, Basel, Switzerland; 937 Queen Elizabeth University Hospital, NHS GG&C, Glasgow, UK; 938 Fondazione IRCCS Ca’ Granda Ospedale Maggiore Policlinico, Milano, Italy; 939 Plug Working Group, ESICM, Milano, Italy; 940 Sant’Anna Hospital, University of Ferrara, Ferrara, Italy; 941 University of Milan, Milano, Italy; 942 CHU Bordeaux, Réanimation Médicale, Bordeaux, France; 943 CHU Bordeaux, Unité d’Épidemiologie Clinique, Bordeaux, France; 944 Hospital Universitario Reina Sofia, Intensive Care Unit, Córdoba, Spain; 945 Complejo Hospitalario de Jaén, Intensive Care Unit, Jaén, Spain; 946 Critical Care Department, Vall d’Hebron University Hospital, Barcelona, Spain; 947 Universitat Autònoma de Barcelona, Medicine, Barcelona, Spain; 948 Critical Care Department, Parc de Salut Mar. IMIM (Mar Medical Research Institut), Barcelona, Spain; 949 Critical Care Department, Joan XXIII University Hospital, Tarragona, Spain; 950 Instituto de Salud Carlos III, Ciber Enfermedades Respiratorias (Ciberes), Madrid, Spain; 951 Department of Internal Medicine, Dankook University Hospital, Dankook University College of Medicine, Cheonan, Republic of Korea; 952 309th Hospital PLA, Beijing, China; 953 Yamagata University Faculty of Medicine, Anaesthesiology and Critical Care Medicine, Yamagata, Japan; 954 Hospital Universitario Reina Sofia, Intensive Care Unit, Córdoba, Spain; 955 Centre Hospitalier, Intensive Care Unit, Roanne, France; 956 Keenan Research Centre, Li Ka Shing Knowledge Institute, St. Michael’s Hospital, Toronto, Canada; 957 Interdepartmental Division of Critical Care Medicine, University of Toronto, Toronto, Canada; 958 Division of Respiratory Diseases and Tuberculosis, Department of Medicine, Faculty of Medicine Siriraj Hospital, Bangkok, Thailand; 959 Neurophysiologie Respiratoire Expérimentale et Clinique, Sorbonne Universités, Paris, France; 960 Division of Respirology, St. Michael’s Hospital, Toronto, Canada; 961 Department of Anesthesiology, Faculty of Medicine, Yamagata University, Yamagata, Japan; 962 Northern General Hospital, Sheffield Teaching Hospital NHS Trust, Anaesthesia and Critical Care, Sheffield, UK; 963 Hospital Universitario Ramón y Cajal, Unidad de Cuidados Intensivos, Madrid, Spain; 964 Department of Anesthesia and Intensive Care, Catholic University of Rome, Rome, Italy; 965 Centro Hospitalar de Entre o Douro e Vouga, Serviço de Medicina Intensiva Polivalente, Santa Maria da Feira, Portugal; 966 Department of Medicine, Massachusetts General Hospital, Boston, MA USA; 967 Department of Surgery, Massachusetts General Hospital, Boston, MA USA; 968 Spaulding Rehabilitation Hospital, Physical Medicine & Rehabilitation, Boston, MA USA; 969 Harvard Medical School, Biomedical Informatics, Boston, MA USA; 970 Massachusetts General Hospital, Renal Division, Boston, MA USA; 971 Massachusetts General Hospital, Pulmonary and Critical Care Medicine, Boston, MA USA; 972 Brigham and Women’s Hospital, Renal Division, Boston, MA USA; 973 Brigham and Women’s Hospital, Channing Division of Network Medicine, Boston, MA USA; 974 Barts Health NHS Trust, Adult Critical Care Unit, London, UK; 975 Department of Intensive Care, Pamela Youde Nethersole Eastern Hospital, Hong Kong, Hong Kong China; 976 Anesthesiology and Reanimation Department, Ministry of Health, Ankara Training and Research Hospital, Intensive Care Unit, Ankara, Turkey; 977 Ministry of Health, Departmant of Health Services, Ankara, Turkey; 978 Medical Biostatistics Department, University of Ankara, Faculty of Medicine, Ankara, Turkey; 979 Department of Anaesthesiology and Intensive Care, Sahlgrenska University Hospital, Gothenburg, Sweden; 980 University of Gothenburg, Gothenburg, Sweden; 981 Queen Alexandra Hospital, Critical Care, Portsmouth, UK; 982 Hospital de Clínicas de Porto Alegre, Porto Alegre, Brazil; 983 Hospital Moinhos de Vento, Porto Alegre, Brazil; 984 East Surrey Hospital, Intensive Care, Redhill, UK; 985 Queen Elizabeth University Hospital, Anaesthetics and Intensive Care, Glasgow, UK; 986 University of Cambridge, Gonville & Caius College, Cambridge, UK; 987 Papworth Hospital, Papworth Everard, UK; 988 Imperial College London, London, UK; 989 Hospital Clínico Universitario de Santiago de Compostela, Critical Care Unit, Santiago de Compostela, Spain; 990 Hospital Clínico Universitario de Santiago de Compostela, Abdominal Transplant Unit, Santiago de Compostela, Spain; 991 St. George’s Healthcare Trust, Neuro ICU, London, UK; 992 Hospital Universitari Joan XXIII, Intensive Care Unit, Tarragona, Spain; 993 Hospital Universitario de Torrejón, Intensive Care Unit, Madrid, Spain; 994 Department of Preventive Medicine, Hospital Universitari Joan XXIII, Tarragona, Spain; 995 WPK Hopsital, Vienna, Austria; 996 Royal Surrey County Hospital, Guildford, UK; 997 St. George’s Healthcare Trust, London, UK; 998 Hospital de Santa Maria/CHLN, Serviço de Medicina Intensiva, Lisboa, Portugal; 999 Faculdade de Medicina, Universidade de Lisboa, Lisbon, Portugal; 1000 Hospital de Santa Maria/CHLN, Lisboa, Portugal; 1001 Hospital Universitario Infanta Leonor, Intensive Care Unit, Madrid, Spain; 1002 Hospital Universitario Miguel Servet, Medicina Intensiva, Zaragoza, Spain; 1003 Hospital Comarcal Santa Ana, UCI, Motril, Spain; 1004 Hospital Comarcal Santa Ana, Motril, Spain; 1005 Inha University Hospital, Pulmonary and Critical Care, Incheon, Republic of Korea; 1006 Inha University, Internal Medicine, Incheon, Republic of Korea; 1007 Inha University Hospital, Cardiovascular Medicine, Incheon, Republic of Korea; 1008 University Hospital of Wales, Anaesthetics, Cardiff, UK; 1009 Royal Gwent Hospital, Newport, UK; 1010 Hospital Universitario Miguel Servet, Medicina Intensiva, Zaragoza, Spain; 1011 Sheffield Teaching Hospitals NHS Foundation Trust, Sheffield, UK; 1012 University College London Hospitals NHS Foundation Trust, London, UK; 1013 Southampton University Hospitals NHS Trust, Southampton, UK; 1014 Oxford University Hospitals NHS Foundation Trust, Oxford, UK; 1015 Guy’s and St Thomas’ NHS Foundation Trust, London, UK; 1016 Complejo Hospitalario de Granada, Granada, Spain; 1017 Department of Anesthesiology and Intensive Care Medicine, Jena University Hospital, Jena, Germany; 1018 Jena University Hospital, Integrated Research and Treatment Center for Sepsis Control and Care (CSCC), Jena, Germany; 1019 Oxford University Hospitals NHS Trust, OCCULAR Group, Adult Intensive Care Unit, Oxford, UK; 1020 Chulalongkorn university, Medicine, Bangkok, Thailand; 1021 University of Queensland, Brisbane, Australia; 1022 Griffith University, Brisbane, Australia; 1023 SSA UADY, Mérida, Mexico; 1024 NN Blokhin Russisn Cancer Research Center, Moscow, Russian Federation; 1025 Moscow Botkin Clinic, Moscow, Russian Federation; 1026 North District Hospital, Hong Kong, Hong Kong China; 1027 Samsung Medical Center, Critical Care Medicine, Seoul, Republic of Korea; 1028 Samung Medical Center, Medicine, Seoul, Republic of Korea; 1029 Samsung Medical Center, Surgery, Seoul, Republic of Korea; 1030 Department of Anesthesiology, National Taiwan University Hospital, Taipei, Taiwan, Province of China; 1031 Department of Nursing, National Taiwan University Hospital, Taipei, Taiwan, Province of China; 1032 Department of Surgery, National Taiwan University Hospital, Taipei, Taiwan, Province of China; 1033 Ospedale Sant’Anna e San Sebastiano, U.O. di Anestesia e Rianimazione, Caserta, Italy; 1034 Ospedale S. Maria delle Grazie - ASL Napoli 2 Nord, U.O. di Anestesia e Rianimazione, Pozzuoli, Italy; 1035 AOUP Università degli Studi di Pisa, IV U.O. di Anestesia e Rianimazione, Pisa, Italy; 1036 San Marco Hospital, Anesthesia and Intensive Care, Zingonia, Italy; 1037 Department of Intensive Care Medicine, Radboud University Nijmegen Medical Centre, Nijmegen, Netherlands; 1038 Department of Anesthesiology, Radboud University Nijmegen Medical Centre, Nijmegen, Netherlands; 1039 Department of Internal Medicine, Radboud University Nijmegen Medical Centre, Nijmegen, Netherlands; 1040 Department of Pharmacology and Toxicology, Radboud University Nijmegen Medical Centre, Nijmegen, Netherlands; 1041 Department of Cardiology, Canisius Wilhelmina Ziekenhuis, Nijmegen, Netherlands; 1042 Department of Anesthesiology, Asahikawa Medical University, Asahikawa City, Hokkaido Japan; 1043 Department of Emergency Medicine, Asahikawa Medical University, Asahikawa City, Hokkaido Japan; 1044 Federal University of São Paulo, Surgery, São Paulo, Brazil; 1045 Federal University of São Paulo, Morphology and Genetics, São Paulo, Brazil; 1046 Inserm, UMR-S 942, Paris, France; 1047 AP-HP, Department of Anesthesia and Critical Care, Hôpitaux Universitaires Saint Louis-Lariboisière, Paris, France; 1048 Sorbonne Paris Cité, University Paris Diderot, Paris, France; 1049 CHU Nancy, Hôpital Central, Medical Intensive Care Unit, Nancy, France; 1050 AP-HP, Cardiology, Paris, France; 1051 AP-HP, Biochemistry and Molecular Biology, Paris, France; 1052 CHU Nancy, Hôpital Brabois, Medical Intensive Care Unit, Nancy, France; 1053 Danube University Krems, Center for Biomedical Technology, Krems, Austria

## Oral Sessions. ARDS: CLINICAL STUDIES

### A1 Identification of distinct endophenotypes in patients with acute respiratory distress syndrome by unbiased cluster analysis, and their association with mortality

#### L. Bos^1^, L. Schouten^1^, L. van Vught^1^, M. Wiewel^1^, D. Ong^2^, O. Cremer^2^, A. Artigas^3^, I. Martin-Loeches^4^, A. Hoogendijk^1^, T. van der Poll^1^, J. Horn^1^, N. Juffermans^1^, M. Schultz^1^

##### ^1^Academic Medical Center, University of Amsterdam, Amsterdam, Netherlands; ^2^UMCU, Utrecht, Netherlands; ^3^Autonomous University of Barcelona, Barcelona, Spain; ^4^Hospital São Francisco Xavier, Lisbon, Portugal

###### **Correspondence:** L. Bos – Academic Medical Center, University of Amsterdam, Amsterdam, Netherlands

**Introduction:** Pharmacological immunomodulatory interventions in 'acute respiratory distress syndrome' (ARDS) have been unsuccessful in clinical trials [1-3] despite promising results in preclinical studies using animals [4-5]. Poor phenotyping of patients could be responsible for these disappointing results.

**Objectives:** We hypothesized that ARDS patients can be clustered based on concentrations of plasma biomarkers and that such biological endophenotypes are association with clinical outcomes.

**Methods:** Patients were screened for presence of ARDS. Unbiased cluster analysis of plasma concentrations of 20 biomarkers of inflammation, coagulation and endothelial activation at diagnosis of ARDS provided the endophenotypes. A decision tree was then used to predict cluster membership based on a more restricted set of biomarkers. The independent association of endophenotypes with ICU mortality was studied by multivariate logistic regression.

**Results:** Three endophenotypes of ARDS were identified in 771 patients, which we named 'impassive' (N = 383), 'intermediate' (N = 224) and 'reactive' (N = 164), had mortality rates of 16 %, 26 % and 47 %, respectively (P < 0.01). Patients with a 'reactive' endophenotype were younger, had higher disease severity scores, more failing organs and more frequently had an indirect cause for ARDS than patients with an 'impassive' or 'intermediate' endophenotype. A 'reactive endophenotype' was independent from confounders associated with ICU mortality (OR 1.18 [95 % confidence interval: 1.09-1.28]). The concentration of interleukin 10, interleukin 8 and matrix metalloproteinase 8 were sufficient to predict the three endophenotypes.

**Conclusions:** ARDS patients can be clustered into three biological endophenotypes, with different mortality rates. Three easy to measure biomarkers can be used to predict the endophenotype.

**References**

1. Takeda S. *Pulm Pharmacol Ther* 2005.

2. Boyle AJ. *Expert Opin Biol Ther* 2014.

3. Cepkova M. *J Intensive Care Med* 2006.

4. Calfee CS. *Chest* 2007.

5. Beitler JR. *Chest* 2014.

**Grant acknowledgement**

This study is supported by the MARS consortium, a public-private partnership.

**Table 1 (abstract A1).** Endophenotypes versus clinical characteristics

	Impassive endophenotype	N=383	Intermediate endophenotype	N=224	Reactive endophenotype	N=164	P-value
Age	63	(53.5-72)	61.5	(51.8-72)	58	(45-66)	<0.001
Male	252	(65.8)	129	(57.6)	107	(65.2)	0.1
APACHE IV Score	72	(58-92)	83	(67.5-102)	104.5	(84-124)	<0.001
SOFA: Total score	7	(5-9)	9	(7-11)	11	(9-14)	<0.001
PaO2/FiO2	191.1	(138-260)	183.2	(146-234)	173.3	(124-225)	<0.001
PEEP	8	(5-11)	9	(6-12)	12	(10-15)	<0.001
Days free of MV at day 28	20	(8-25)	18 (0-24)	0	(0-18)	<0.001	<0.001
ICU Mortality	61	(15.9)	60	(26.8)	76	(46.3)	<0.001
30-Day Mortality	75	(19.6)	70	(31.2)	78	(47.8)	<0.001

### A2 Acute respiratory distress syndrome with no risk factor of the berlin definition: an ancillary analysis of the LUNG SAFE study

#### N. de Prost^1,2^, T. Pham^3,4,5^, G. Carteaux^1,2^, A. Mekontso Dessap^1,2^, C. Brun-Buisson^1,2^, E. Fan^6^, G. Bellani^7^, J. Laffey^8^, A. Mercat^9^, L. Brochard^10^, B. Maitre^1,2^, LUNG SAFE investigators and the ESICM study group

##### ^1^CHU Henri Mondor, Medical ICU, Créteil, France; ^2^Université de Paris Est-Créteil, Groupe de Recherche Clinique CARMAS, Créteil, France; ^3^Hôpital Tenon, APHP, Medical and Surgical ICU, Paris, France; ^4^Université Paris Diderot, Sorbonne Paris Cité, UMR 1153, Paris, France; ^5^University of Toronto, Interdepartmental Division of Critical Care, Toronto, Canada; ^6^University Health Network, University of Toronto, Critical Care Medicine, Toronto, Canada; ^7^University of Milan - Bicocca, Health Sciences, School of Medicine and Surgery, Monza, Italy; ^8^St Michael's Hospital, University of Toronto, Anesthesia, Toronto, Canada; ^9^CHU d'Angers, Medical ICU, Angers, France; ^10^University of Toronto Saint Michael's Hospital and Keenan Research Centre, Interdepartmental Division of Critical Care, Toronto, Canada

###### **Correspondence:** T. Pham – Hôpital Tenon, APHP, Medical and Surgical ICU, Paris, France

**Introduction:** Patients meeting the Berlin definition criteria for the acute respiratory distress syndrome (ARDS) might lack exposure to one or more “common” risk factors. Such patients might exhibit different clinical phenotype and outcomes than others and constitute an individualized subgroup of patients.

**Objectives:** To compare the clinical presentation and outcome of patients having ARDS with *vs* without risk factors, to determine whether the lack of ARDS risk factor is associated with hospital mortality, and to identify factors associated with hospital mortality in the subgroup of ARDS patients with no risk factors.

**Methods:** Ancillary study of an international, multicenter, prospective cohort study (LUNG SAFE study[1]). Patients meeting ARDS criteria (Berlin definition) on day 1 or 2 of acute hypoxemic respiratory failure onset were included in the study and categorized as having “common” risk factors or not.

**Results:** Among the 2813 patients presenting ARDS in the first 48 h, 266 patients (9.4 %) had no ARDS risk factor identified at admission. Table 2 shows the final ARDS risk factor identified in patients with or without initial risk factor identified.

The patients with no risk factor were older, had more frequent previously known chronic diseases and presented with less severe SOFA (8.7 ± 3.9 vs 9.5 ± 4.1, p < 0.001) and non-pulmonary (5.4 ± 3.9 vs 6.3 ± 4.1, p < 0.001) SOFA scores. ICU mortality was lower in ARDS patients with no risk factor than in others (28.6 % *vs* 34.9 %, p = 0.047), but in-hospital mortality was not (35.7 % *vs* 39.8 %, p = 0.20). The lack of ARDS risk factor was not associated with hospital mortality (adjusted OR = 0.86 [0.65-1.13], p = 0.29). In the subgroup of patients with no ARDS risk factor, age, SOFA, concomitant heart failure, and administration of steroids within 72 hours of ARDS onset were associated with hospital mortality (Table 3).

**Conclusions:** Almost ten percent of patients with ARDS had no risk factor identified and exhibit a different clinical phenotype than others. Future research aimed at studying management strategies in this subgroup of patients is warranted.

**References**

[1] Bellani G, et al. JAMA. 2016 Feb 23;315(8):788800

Trial Registration: ClinicalTrials.gov NCT02010073

**Grant acknowledgement**

The LUNG SAFE study was supported by the ESICM.

**Table 2 (abstract A2).** Risk factors eventually identified N(%)

	ARDS patients with ≥1 risk factor identified upon ARDS diagnosisa (n=2547)	ARDS patients with no risk factor identified upon ARDS diagnosis (n=266)
Pneumonia	1670 (65.6)	13 (4.9)
Non-pulmonary Sepsis	453 (17.8)	2 (0.8)
Aspiration of gastric contents	400 (15.7)	0 (0.0)
Non cardiogenic shock	214 (8.4)	0 (0.0)
Trauma	199 (7.8)	0 (0.0)
Blood transfusion	111 (4.4)	0 (0.0)
Pulmonary vasculitis	24 (0.9)	14 (5.3)
Others	264 (10.4)	16 (6.0)
No risk factor identified	0 (0.0)	219 (82.3)

**Table 3:** (abstract A2).

	OR	95%CI	p-value
Age (for 1 year)	1.03	1.01-1.05	0.003
SOFA score (for 1 point)	1.11	1.03-1.19	0.004
Concomitant heart failure	1.96	1.07-3.63	0.030
Steroids in the 1st 72 h of ARDS	2.66	1.37-5.25	0.004

### A3 The consequences of the acute respiratory distress syndrome in patients undergoing oesophagectomy

#### P.A. Howells^1^, D.R. Thickett^1^, C. Knox^2^, D.P. Park^3^, F. Gao^1,3^, O. Tucker^4^, T. Whitehouse^5^, D.F. McAuley^6,7^, G.D. Perkins^3,8^

##### ^1^University of Birmingham, Institute for Inflammation and Ageing, Birmingham, United Kingdom; ^2^University of Sheffield, Mathematics and Statistics Help Centre, Sheffield, United Kingdom; ^3^Heart of England NHS Foundation Trust, Intensive Care Medicine, Birmingham, United Kingdom; ^4^University Hospitals Birmingham NHS Trust, Department of Surgery, Birmingham, United Kingdom; ^5^University Hospitals Birmingham NHS Trust, Anaesthesia and Critical Care Medicine, Birmingham, United Kingdom; ^6^Queen's University of Belfast, Wellcome-Wolfson Institute for Experimental Medicine, Belfast, United Kingdom; ^7^Royal Victoria Hospital, Intensive Care Medicine, Belfast, United Kingdom; ^8^University of Warwick, Warwick Clinical Trials Unit, Coventry, United Kingdom

###### **Correspondence:** P.A. Howells – University of Birmingham, Institute for Inflammation and Ageing, Birmingham, United Kingdom

**Introduction:** The Acute Respiratory Distress Syndrome (ARDS) is a serious complication following major surgery^1^. ARDS frequently complicates oesophagectomy. The Beta Agonist Lung Injury Prevention Trial (BALTI-P)^2^ provided a large cohort of patients having undergone oesophagectomy who had been systematically screened for ARDS.

**Objectives:** To characterise patients developing ARDS following oesophagectomy and identify risk factors for ARDS in this group.

**Methods:** Data were collected as part of the BALTI-P trial, which included daily assessment of oxygenation. Chest x-rays were assessed by an expert panel. A comparison of Early ARDS (first post-operative 72 hours) and Late ARDS (after 72 hours) was undertaken using univariate and multivariate analysis. Differences in outcome were determined and risk factors for ARDS in this group were identified.

**Results:** There were 83 cases of ARDS, 59 (71 %) were Early and 24 (29 %) were Late. ARDS with associated with longer ICU (Difference 7.18 days (Confidence Interval 5.53, 8.82)) and hospital stay (Difference 5.72 days (CI 4.05, 7.40)), fewer organ failure-free days (Difference -3.23 days (CI -4.36, -2.10)) and fewer ventilator-free days (Difference -6.50 days (CI -7.90, -5.10)). There was no difference in mortality or quality of life score. Late ARDS had the worst outcomes. Early ARDS was associated with increased age (Odds Ratio 1.06 (CI 1.00 to 1.13), p = 0.05) and mid-oesophageal tumours (OR 7.48 (CI 1.62-34.5), p = 0.01), whereas gastro-oesophageal tumours were protective (OR 0.21 (CI 0.05, 0.85), p = 0.03). ARDS was associated with more adverse events.

**Conclusions:** ARDS is associated with adverse patient outcomes and increased healthcare resource utilisation. Patients with mid-oesophageal tumours may be at especially high risk. Further investigations aimed at reducing perioperative ARDS are warranted^3^. The high risk of ARDS following oesophagectomy makes the patient group useful for conducting trials into preventative therapies, as the incidence of ARDS high and the onset is predictable, therefore patients can be approached pre-operatively.

**References**

1. Singh GG, Chandy TT, Sen N. Incidence and outcome of acute lung injury and acute respiratory distress syndrome in the surgical intensive care unit. Indian J Crit Care Med 2014; 18: 659-65.

2. Perkins GD, Gates S, Park D et al. The beta agonist lung injury trial prevention. A randomized controlled trial. Am J Respir Crit Care Med 2014; 189: 674-83.

3. Proudfoot AG, McAuley DF, Griffiths MJ, Hind M. Human models of acute lung injury. Dis Model Mech 2011; 4: 145-53.

**Grant acknowledgements**

The BALTI-prevention study was supported by the National Institute for Health Research. PAH is funded by a grant provided by GlaxoSmithKline, DRT by the Medical Research Council and GDP and FG receive support as National Institute for Health Research Senior Investigators. GDP and DFM are supported as Directors of Research for the Intensive Care Foundation.

### A4 Epidemiology, patterns of care, and outcome of trauma patients with acute hypoxemic respiratory failure: insights from the LUNG SAFE study

#### T. Pham^1^, J. Laffey^2^, G. Bellani^3^, E. Fan^4^, LUNG SAFE Investigators and the ESICM Trials Group

##### ^1^St Michael's Hospital, University of Toronto, Critical Care, Toronto, Canada; ^2^St Michael's Hospital, University of Toronto, Anesthesia, Toronto, Canada; ^3^University of Milan-Bicocca, Health Sciences, School of Medicine and Surgery, Monza, Italy; ^4^University Health Network, University of Toronto, Critical Care Medicine, Toronto, Canada

###### **Correspondence:** T. Pham – St Michael's Hospital, University of Toronto, Critical Care, Toronto, Canada

**Introduction:** There is limited information about the epidemiology, recognition, management, and outcomes of trauma patients with the acute hypoxemic respiratory failure (AHRF).

**Objectives:** To assess the demographics, clinician recognition, ventilation management, use of adjunctive measures, and outcome of trauma patients with AHRF that were enrolled into the LUNG SAFE study.

**Methods:** LUNG SAFE was an international, multicenter, prospective cohort study of patients undergoing invasive or noninvasive ventilation, conducted during four consecutive weeks in the winter of 2014 in a convenience sample of 459 ICUs from 50 countries across 5 continents. In this analysis, we compared data from patients with AHRF secondary to trauma to the general AHRF patient population. We defined this cohort as that group of patients in whom the presence of trauma and/or a pulmonary contusion was considered a risk factor for their AHRF.

**Results:** Of 4,041 patients admitted to participating ICUs with AHRF, 229 (5.7 %) had sustained a trauma and/or a pulmonary contusion [Table]. Trauma patients were younger, and had a higher male predominance, and less comorbidities and lower illness severity compared to the general AHRF patient population. Of the trauma patients that developed ARDS, clinician recognition was 35 %, which lower than that that overall. In regard to ventilator management, tidal volumes and PEEP levels were comparable to that seen in the general AHRF group. The use of non-invasive ventilation was lower, neuromuscular blockade use was comparable (12.2 % versus 14.5 %), while there was less use of prone positioning (0.4 % versus 4.3 %). Outcome of trauma patients with AHRF was better than that in the overall AHRF population, with greater ICU (82.5 % versus 67.4 %) and hospital (81.2 % versus 61.3 %) survival compared to the general AHRF population. Trauma was independently associated with a reduced risk of death in a multivariable analysis.

**Conclusions:** Trauma patients constituted a distinct cohort, being younger, predominantly male, and with less comorbidities, and improved outcome compared to the general AHRF cohort

**References**

1. Bellani G, et al. JAMA. 2016 Feb 23;315(8):788-800

TRIAL REGISTRATION. ClinicalTrials.gov NCT02010073

**Grant acknowledgement**

This study was supported by the ESICM.

**Table 4 (abstract A4).** Characteristics of trauma patients in LUNG SAFE

	Trauma with AHRF N=229 (5.7%)	Non-Trauma with AHRF N=3812 (94.7%)	P value
Age, years, mean ±SD	51.4±19	62±16	<0.001
Male patients, No (%)	175 (76.4)	2336 (61.3)	<0.001
Day 1 SOFA score , mean ±SD	8.5±4.0	9.3±4.1	0.004
P/F ratio , mmHg, mean ±SD	187±66	167±67	<0.001
Vt, mL/kg PBW, mean ±SD	7.6±1.5	7.8±2.1	0.127
Set PEEP, cmH2O, mean ±SD	7.3±2.8	7.8±3.1	0.007

**Table 5 (abstract A4).** Management and outcome of trauma patients

	Trauma with AHRF N=229 (5.7%)	Non-Trauma with AHRF N=3812 (94.7%)	P value
NIV during the 48 first hours, No (%)	8 (3.5)	585 (15.4)	<0.001
Use of Neuromuscular Blockade, No. (%)	28 (12.2)	553 (14.5)	0.340
Use of Prone positioning, No. (%)	1 (0.4)	165 (4.3)	0.017
Duration of invasive mechanical ventilation, median [IQR], days	9 [4-14]	7 [4-14]	0.187
Duration of ICU Stay, median [95%IQR], days	11 [7-20]	9 [5-14]	0.004
ICU Survival, No. (%)	189 (82.5)	2570 (67.4)	<0.001
Duration of Hospital Stay, median [IQR], days	22 [10-39]	16 [8-30]	<0.001
Hospital Survival, No. (%)	186 (81.2)	2335 (61.3)	<0.001

### A5 Prognostication in patients with ARDS using SpO_2_/FiO_2_ and peep cutoffs at onset of ARDS and after 24 hours

#### L. Pisani^1,2^, J.-P. Roozeman^1^, F.D. Simonis^1^, A. Giangregorio^1^, L.R. Schouten^1^, S.M. Van der Hoeven^1^, J. Horn^1^, A. Serpa Neto^1,3^, E. Festic^4^, A.M. Dondorp^1,5^, S. Grasso^2^, L.D. Bos^1^, M.J. Schultz^1,5^

##### ^1^Academic Medical Center, University of Amsterdam, Amsterdam, Netherlands; ^2^University of Bari 'Aldo Moro', Bari, Italy; ^3^Hospital Israelita Albert Einstein, São Paulo, Brazil; ^4^Mayo Clinic, Jacksonville, United States; ^5^Mahidol University, Faculty of Tropical Medicine, Bangkok, Thailand

###### **Correspondence:** L. Pisani – Academic Medical Center, University of Amsterdam, Amsterdam, Netherlands

**Introduction:** Prognostication in patients with ARDS improves when patients are reclassified after 24 hours using PaO2/FiO2 and PEEP cutoffs [1,2]. It is uncertain if SpO2/FiO2 [3] could serve as a non-invasive and continuous surrogate of PaO2/FiO2 in prognostication of ARDS patients. We hypothesized that SpO2/FiO2, in combination with PEEP, is a reliable alternative for PaO2/FiO2 in prognostication of patients with moderate or severe ARDS.

**Objectives:** To investigate whether classification at onset and re-classification after 24 hours using SpO2/FiO2 and PEEP cutoffs allow outcome prognostication in patients with moderate or severe ARDS according to the Berlin definition [4].

**Methods:** A secondary analysis of a large prospective observational study in the mixed intensive care unit of a university hospital in the Netherlands. First, the relation between PaO2/FiO2 and SpO2/FiO2 was determined. Then patients were assigned to four groups, at onset of ARDS and after 24 hours: group I (SpO2/FiO2 ≥ 190 and PEEP < 10 cm H2O), group II (SpO2/FiO2 ≥ 190 and PEEP ≥ 10 cm), group III (SpO2/FiO2 < 190 and PEEP < 10 cm H2O), and group IV (SpO2/FiO2 < 190 and PEEP ≥ 10 cm H2O). The primary outcome was all-cause in-hospital mortality. Secondary outcomes were ICU-, 30-, 90-day- and 1 year mortality and the number of ventilator-free days and alive at day 28.

**Results:** The analysis included 456 patients with moderate or severe ARDS who stayed in the ICU > 24 hours. The relation between PaO2/FiO2 and SpO2/FiO2 was good (R2 = 0.62). Using the predefined cutoffs for SpO2/FiO2 and PEEP, prognostication improved with re-classification after 24 hours (Tables 6 and 7).

**Conclusions:** The SpO2/FiO2 is a reliable alternative for PaO2/FiO2 in prognostication at 24 hours after onset of moderate or severe ARDS.

**References**

1. Villar J, Fernández RL, Ambrós A, et al. Crit Care Med. 2015;43(2):346-353.

2. Bos LD, Cremer OL et al. Intensive Care Med. 2015;41(11):2004-05

3. Rice TW, Wheeler AP, Bernard GR, et al. Chest. 2007;132(2):410-417

4. The ARDS Definition Task Force. JAMA. 2012;307(23):2526-2533

**Grant acknowledgement**

This research was performed within the framework of CTMM, the Center for Translational Molecular Medicine (www.ctmm.nl) project MARS (grant 04I-201).

**Table 6 (abstract A5).** Distribution and Outcomes at baseline

	Group I	Group II	Group III	Group IV	p value	OR (95% CI) group IV vs group I
Baseline ARDS diagnosis	SpO2/FiO2 ≥ 190 and PEEP < 10	SpO2/FiO2 ≥ 190 and PEEP ≥ 10	SpO2/FiO2 < 190 and PEEP < 10	SpO2/FiO2 < 190 and PEEP ≥ 10		
Number of patients (N)	87	43	100	226		
ICU mortality (%)	19.5	23.3	18.0	30.1	0.042	1.77 (0.99 - 3.30)
In-hospital mortality (%)	44.8	30.2	36.0	41.2	0.897	0.85 (0.52 - 1.40)
30-day mortality (%)	27.6	27.9	24.0	33.2	0.276	1.30 (0.75 - 2.23)
90 days mortality (%)	47.1	34.9	41.0	42.0	0.636	0.81 (0.50 - 1.34)
1 year mortality (%)	60.9	41.9	47.0	49.6	0.182	0.63 (0.38 - 1.04)
VFD-28 (days-IQR)	19 (7-24)	22 (6.5-25)	20.5 (6-25)	18 (0-23)	0.070*	-

Data are medians [IQR] or percentages

Abbreviations: *ARDS* acute respiratory distress syndrome, *VFD-28* ventilator free days and alive at day 28

P-value is p-for trend or p for Kruskall-Wallis test*

**Table 7 (abstract A5).** Distribution and Outcomes after 24 hours

After 24h	Group I	Group II	Group III	Group IV	p value	OR (95% CI) group IV vs group I
	SpO2/FiO2 ≥ 190 and PEEP < 10	SpO2/FiO2 ≥ 190 and PEEP ≥ 10	SpO2/FiO2 < 190 and PEEP < 10	SpO2/FiO2 < 190 and PEEP ≥ 10		
Number of patients (N)	213	145	17	81		
ICU mortality (%)	15.5	24.1	35.3	48.2	<0.001	5.06 (2.99 - 9.04)
In-hospital mortality (%)	34.7	35.9	52.9	56.8	<0.001	2.52 (1.50 - 4.25)
30-day mortality (%)	23.9	26.9	47.1	45.7	<0.001	2.64 (1.54 - 4.52)
90 days mortality (%)	38.0	38.6	52.9	56.8	0.003	2.14 (1.28 - 3.62)
1 year mortality (%)	47.4	45.5	58.8	65.4	0.006	2.10 (1.24 - 3.60)
VFD-28 (days - IQR)	23 (15-26)	18 (0-23)	6 (0-17)	0 (0-17)	<0.001*	--

Data are medians [IQR] or percentages

Abbreviations: *ARDS* acute respiratory distress syndrome, *VFD-28* ventilator free days and alive at day 28

P-value is p-for trend or p for Kruskall-Wallis test*

## SEPSIS DIAGNOSIS IN 2016

### A6 Validation of a molecular host response assay to diagnose infection in hospitalized patients admitted to the ICU with acute respiratory failure

#### M. Koster-Brouwer^1,2^, D. Verboom^1,2^, B. Scicluna^3,4^, K. van de Groep^1,2^, J. Frencken^1,2^, M. Schultz^5^, T. van der Poll^3,4,6^, M. Bonten^2,7^, O. Cremer^1^

##### ^1^University Medical Center Utrecht, Intensive Care, Utrecht, Netherlands; ^2^University Medical Center Utrecht, Julius Center for Health Sciences and Primary Care, Utrecht, Netherlands; ^3^Academic Medical Center, University of Amsterdam, Center for Experimental and Molecular Medicine, Amsterdam, Netherlands; ^4^Academic Medical Center, University of Amsterdam, Center for Infection and Immunity Amsterdam, Amsterdam, Netherlands; ^5^Academic Medical Center, University of Amsterdam, Intensive Care, Amsterdam, Netherlands; ^6^Academic Medical Center, University of Amsterdam, Infectious Diseases, Amsterdam, Netherlands; ^7^University Medical Center Utrecht, Medical Microbiology, Utrecht, Netherlands

###### **Correspondence:** M. Koster-Brouwer – University Medical Center Utrecht, Intensive Care, Utrecht, Netherlands

**Introduction:** The differential diagnosis of acute respiratory failure (ARF) in hospitalized patients is extensive and includes congestive heart failure, atelectasis, nosocomial pneumonia, ARDS and sepsis. A novel diagnostic test based on the expression of four RNAs in peripheral blood (SeptiCyte LAB, Immunexpress, Seatle, WA) may facilitate discrimination between infectious and non-infectious causes in this setting [1].

**Objectives:** To explore the diagnostic and prognostic value of SeptiCyte LAB in ARF patients admitted to the ICU from hospital wards.

**Methods:** We enrolled consecutive patients with ARF who had been hospitalized >48 hrs and required prompt intubation in the ICUs of two Dutch university hospitals from 2011 to 2013. All patients fulfilled ≥2 SIRS criteria and/or had an early warning score >5. We excluded patients having an established diagnosis of infection >2 days before ICU admission as well as those with airway obstruction, circulatory arrest, and other pertinent reasons for mechanical ventilation. Blood samples were collected in PAXgene tubes for RNA extraction upon ICU admission and subsequently analyzed on an Applied Biosystems® 7500 fast Dx Real-Time PCR instrument. Test results were categorized into 4 probability bands according to the manufacturer´s specification. Post-hoc infection likelihood of sepsis events within 2 days after ICU admission were based on physician assessments according to validated definitions [2].

**Fig. 1 (abstract A6).**
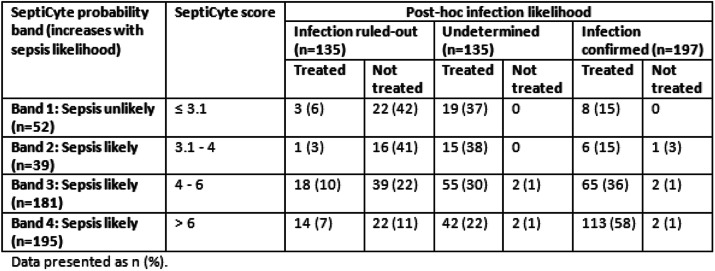
SeptiCyte LAB result versus post-hoc infection likelihood in 467 critically ill patients with acute respiratory failure, stratified by receiving or not receiving antibiotic treatment on the first day in ICU

**Fig. 2 (abstract A6).**
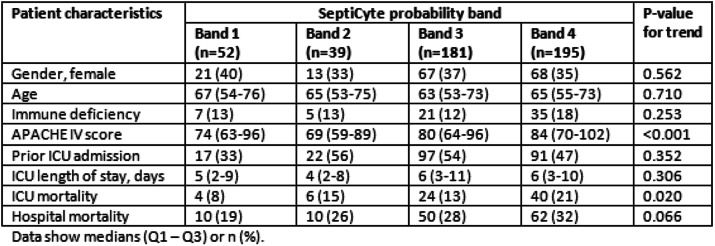
Patient characteristics by SeptiCyte LAB result

**Results:** Sample preparation or processing issues resulted in exclusion of 14 patients, leaving 467/481 (97 %) for final analysis. Of these, 359 (77 %) subjects received antibiotics upon ICU admission, whereas therapy was initiated on a later date in an additional 14 (3 %) patients. Test results correlated with the probability of infection (p < 0.001) (Fig. 1). Among the 415 patients in whom the test classified sepsis as 'likely´, the false positive rate decreased from 17/39 (44 %) to 36/195 (18 %) with higher probability bands. In the 52 patients in whom the test suggested infection to be unlikely we observed 8 cases of confirmed infection (false negative rate 15 %). As 135 patients could not be categorized with certainty (“undetermined”) formal calculation of sensitivity and specificity was precluded. SeptiCyte test results were not affected by age, prior ICU stay, or immune deficiency. Higher scores of the test were indicative of increased severity of disease and mortality (Fig. 2).

**Conclusions:** SeptiCyte LAB is a biomarker assay which may aid clinicians in separating infectious from non-infectious causes of acute respiratory failure in hospitalized patients. In addition, the test may have prognostic utility.

**References**

[1] McHugh L *et al.* Plos Med 2015;12(12):e1001916.

[2] Klein Klouwenberg PM *et al*. CCM 2013;41(10):2373-2378.

**Grant acknowledgement**

We thank Immunexpress for kindly providing lab kits and technical assistance.

**Conflicts of interest:** The authors declare that they do not have a conflict of interest.

### A7 Predictive value of QSOFA for 28-day mortality in emergency department patients with sepsis

#### J.I. Ko^1^, K.S. Kim^1^, G.J. Suh^1^, W.Y. Kwon^1^, K. Kim^2^, J.H. Shin^3^

##### ^1^Seoul National University Hospital, Seoul, Republic of Korea; ^2^Seoul National University Bundang Hospital, Department of Emergency Medicine, Seongnam-si, Republic of Korea; ^3^Seoul National University Boramae Hospital, Department of Emergency Medicine, Seoul, Republic of Korea

###### **Correspondence:** J.I. Ko – Seoul National University Hospital, Seoul, Republic of Korea

**Introduction:** Recently, the 3rd international consensus definitions for sepsis and septic shock have launched. According to the guideline, quick Sequential Organ Failure Assessment (qSOFA) score 2 or more was recommended as clinical criteria to identify sepsis patients outside the ICU. Emergency department (ED) is the major source of ICU admission for sepsis. Therefore, an early recognition of sepsis is essential for the application of bundle therapy in time.

**Objectives:** We wanted to investigate the predictive value of qSOFA for 28-day mortality in ED patients with sepsis.

**Methods:** Patients suspected for old definition of severe sepsis and septic shock were retrospectively identified in 3 urban tertiary hospital EDs from May 2014 to April 2015. Demographic findings, initial vital signs, Glasgow coma scale (GCS), site of infection, initial lactate levels, systemic inflammatory response syndrome criteria (SIRS), qSOFA score, SOFA score, and 28-day mortality were abstracted. Area under the receiver operating characteristics (AUROC) of SIRS, qSOFA, and SOFA to predict 28-day mortality were compared and diagnostic performance of qSOFA 2 or more to predict 28-day mortality was calculated.

**Results:** Total 942 patients were identified during study periods. Among them, 14 patients were excluded because of missing values. Demographic characteristics were described in Table 8.

Patients with qSOFA less than 2 accounted for over half of enrolled patients (493/928, 53.1 %) and over one third of mortality cases (88/231, 38.1 %) (Table 9).

**Fig. 3 (abstract A7).**
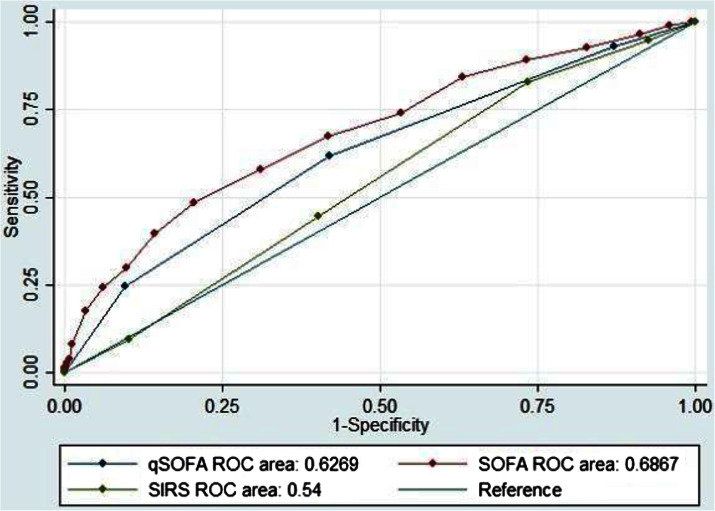
ROC curve to predict 28-d mortality

AUROC of SIRS, qSOFA, and SOFA to predict 28-day mortality were 0.540 (0.500-0.580), 0.627 (0.587-0.667), and 0.687 (0.646-0.727), respectively. (SIRS vs qSOFA [p < 0.001], qSOFA vs SOFA [p = 0.009]) (Fig. 3).

Diagnostic performance of qSOFA 2 or more to predict 28-day mortality was as follows: sensitivity, 61.9 % (55.3 %-68.2 %); specificity, 58.1 % (54.3 %-61.8 %); positive predictive value, 32.9 % (28.5 %-37.5 %); negative predictive value, 82.2 % (78.5 %-85.4 %). Diagnostic performance of SOFA score 2 or more to predict 28-day mortality was as follows: sensitivity, 99.1 % (96.9 %-99.9 %); specificity, 4.2 % (2.8 %-5.9 %); positive predictive value, 25.5 % (22.7 %-28.5 %); negative predictive value, 93.5 % (78.6 %-99.2 %).

**Conclusions:** The current clinical criteria using qSOFA have a better predictive value than SIRS for 28-day mortality in ED patients with sepsis. However, criteria of qSOFA 2 or more can miss one third of mortality cases. Therefore, further assessment of organ failure by SOFA score would be helpful in ED patients with infection.

**Table 8 (abstract A7).** Demographic characteristics

	Total N=928	Survived n=697 (75.1%)	Died n=231 (24.9%)	p value
Age	70.1 (69.3-71.0)	69.2 (68.2-70.2)	72.9 (71.3-74.5)	<.001
Male sex	552 (59.5%)	402 (57.7%)	150 (64.9%)	.051
Infection site				<.001
Respiratory	404 (43.5%)	271 (38.9%)	133 (57.6%)	
Hepatobiliary	186 (20.0%)	152 (21.8%)	34 (14.7%)	
Genitourinary	173 (18.6%)	148 (21.2%)	25 (10.8%)	
Gastrointestinal	67 (7.2%)	52 (7.5%)	15 (6.5%)	
Others	98 (10.6%)	74 (10.6%)	24 (10.4%)

**Table 9 (abstract A7).** qSOFA and 28-day mortality

	qSOFA				
28-d mortality	0	1	2	3	Total
Survived	90 (84.9%)	315 (81.4%)	226 (72.4%)	66 (53.7%)	697 (75.1%)
Died	16 (15.1%)	72 (18.6%)	86 (27.6%)	57 (46.3%)	231 (24.9%)
Subtotal	106	387	312	123	928

### A8 Validation of quick SOFA in a large population of patients with community-acquired pneumonia

#### O.T. Ranzani^1,2,3^, E. Prina^1,2^, R. Menendez^4^, A. Ceccato^1,2,5^, R. Mendez^4^, C. Cilloniz^1,2^, A. Gabarrus^1,2^, M. Ferrer^1,2^, A. Torres^1,2^

##### ^1^Department of Pulmonology, Hospital Clinic of Barcelona, University of Barcelona, Institut D'investigacions August Pi I Sunyer (IDIBAPS), Barcelona, Spain; ^2^Centro de Investigación Biomedica En Red-Enfermedades Respiratorias (CibeRes, CB06/06/0028), Barcelona, Spain; ^3^Respiratory Intensive Care Unit, Pulmonary Division, Heart Institute, Hospital das Clínicas, University of Sao Paulo, São Paulo, Brazil; ^4^Pneumology Department, ISS/Hospital Universitario y Politecnico La Fe, CIBER Enfermedades Respiratorias (CIBERES), Valencia, Spain; ^5^Seccion Neumologia, Hospital Nacional Prof. Alejandro Posadas, Palomar, Argentina

###### **Correspondence:** O.T. Ranzani – Department of Pulmonology, Hospital Clinic of Barcelona, University of Barcelona, Institut D'investigacions August Pi I Sunyer (IDIBAPS), Barcelona, Spain

**Introduction:** The new sepsis consensus introduced the quick SOFA score (qSOFA) as a simple screening tool for the early detection of patients with infection at risk for worse outcome [1]. Community-acquired pneumonia (CAP) is the most common infection responsible for sepsis and external validation of qSOFA is necessary [2]. Interesting, CRB (Confusion, Respiratory rate and Blood pressure), a consolidate score used for CAP, weighs the same variables used in qSOFA with different cut-offs.

**Objectives:** Our aim was to evaluate the performance of qSOFA in patients with CAP and to compare the qSOFA with SIRS, CRB and SOFA scores in predicting mortality.

**Methods:** We included patients with clinical diagnosis of CAP from Hospital Clinic (Barcelona) and Hospital La Fe (Valencia), Spain. Using variables at diagnosis, we calculated SIRS, qSOFA, CRB and SOFA scores. We evaluated the performance of these scores and their additional prediction contribution to a baseline risk model estimated by a logistic regression model including demographic (age, gender) and comorbidity variables (chronic respiratory disease, chronic neurologic disease, liver disease, heart failure, diabetes mellitus, active neoplasia, chronic renal disease, HIV status and etiologic diagnosis). Our primary outcome was in-hospital mortality, and secondary outcomes were in-hospital mortality and/or 3 days of ICU stay and 30 days mortality. We used multiple imputation to deal with missing data.

**Results:** We evaluated 6,874 patients with CAP, mean age 66 (±19) and in-hospital mortality of 442 patients (6.4 %). Discrimination evaluated through the area under the curve (AUC) is in Table 10. SIRS criteria had the worse discrimination performance compared with qSOFA, CRB and SOFA. Calibration plots were comparable among the scores, although overestimation was more pronounced for qSOFA and SOFA scores. When adding the scores to the baseline risk model, the discrimination of Model + SIRS has no change, although improved with qSOFA, CRB and SOFA. Baseline model calibration improved similarly by adding each score in it. Using the cut-off of two points, the sensitivity/specificity for SIRS was 88/22 %, qSOFA 50/82 %, CRB 39/87 % and SOFA 97/23 %. Similar patterns were observed for secondary outcomes and for other measures of performance (Brier Score, IDI).

**Conclusions:** qSOFA, CRB and SOFA were more accurate than SIRS to detect CAP patients with risk to unfavourable outcomes. In this external validation, the cut-off of 2 points for qSOFA had lower sensitivity than expected for a screening tool. CRB outperformed qSOFA and SOFA in terms of calibration and higher specificity in this population.

**References**

1. Singer M, Deutschman CS, et al. The Third International Consensus Definitions for Sepsis and Septic Shock (Sepsis-3). JAMA 2016;315(8):801-10.

2. Prina E, Ranzani OT, Torres A. Community-acquired pneumonia. Lancet 2015;386(9998):1097-108.

**Grant acknowledgement**

A Ceccato is supported by an Long-term ERS Fellowship.

**Table 10 (abstract A8).** Discrimination performance

Scores		Baseline risk model + Scores	
Variable	AUC (95% CI)	Variable	AUC (95% CI)
		Baseline model	0.74 (0.72-0.76)
SIRS criteria	0.58 (0.55-0.60)	Baseline + SIRS	0.75 (0.73-0.77)
qSOFA	0.70 (0.68-0.72	Baseline + qSOFA	0.79 (0.77-0.81)
CRB	0.71 (0.69-0.74)	Baseline + CRB	0.80 (0.78-0.82)
SOFA	0.80 (0.78-0.82)	Baseline + SOFA	0.84 (0.83-0.86)

### A9 Sepsis endotypes defined by heat map clustering

#### A. Urbano^1^, L.A. Zhang^2^, D. Swigon^1^, F. Pike^3^, R.S. Parker^2,4,5^, G. Clermont^2,4,5^

##### ^1^University of Pittsburgh, Department of Mathematics, Pittsburgh, United States; ^2^University of Pittsburgh, Department of Chemical and Petroleum Engineering, Pittsburgh, United States; ^3^University of Pittsburgh, Biostatistics, Pittsburgh, United States; ^4^University of Pittsburgh, CRISMA Center, Department of Critical Care Medicine, Pittsburgh, United States; ^5^University of Pittsburgh, McGowan Institute for Regenerative Medicine, Pittsburgh, United States

###### **Correspondence:** A. Urbano – University of Pittsburgh, Department of Mathematics, Pittsburgh, United States

**Introduction:** Previous studies have linked various cytokine levels and clinical traits to organ failure and mortality, but few have used this data to cluster patients and classify distinct endotypes

**Objectives:** To determine whether clinical biomarkers can define endotypes of sepsis with different rates of 14-day mortality and organ failure

**Methods:** The multicenter, randomized Protocol-Based Care for Early Septic Shock (ProCESS) trial enrolled 1341 patients with septic shock and showed a 16.3 % 14-day mortality. We used hierarchical bi-clustering as an approach to cluster patients using cytokine levels, their early trends and other baseline clinical variables as features. Cytokine levels measured at 0, 6, and 24 hours were included. Variables with high coefficients of variation and significant univariate logistic regression p-values with organ failure outcomes were included as candidate clustering variables. Patient variables were standardized and differences in cluster mortality were examined using the log-rank test. Only patients with complete variable profiles were included in the heat map analysis. After clustering was performed, the association between these clusters, organ failure, defined as a SOFA score of 2 or greater, and 14-day mortality was examined.

**Results:** Unsupervised clustering yielded several subgroups, or endotypes, of interest. Increasing the potential number of subgroups disclosed the emergence of subgroups with distinct clinical feature. At the highest level, patient with high cytokinemia (HC) are proximally distinguished from those with low cytokinemia (LC). Patient with HC further segregated in those with elevated lactate (HCL) (figure), which often had higher than average platelet count. Although HC was associated with generally higher MOF and mortality, HCL portended a particularly poor prognosis. As the dotted line is moved down the top dendrogram, additional subgroups appear, such as patients with acute kidney injury (high blood urea nitrogen (BUN) with low urine output), hyperglycemia, and patients with high systolic blood pressure, high temperature, and high heart rate.

**Conclusions:** Hierarchical clustering identified endotypes of patients at particularly high (or low) risk of organ failure and 14-day mortality. Heat map bi-clustering of septic patients including several domains of information (serum markers, clinical features) is a promising method to group patients into distinct endotypes. Classification of these endotypes can act as a basis for personalized therapies.

**Grant acknowledgement**

NIH R01-GM-105728.

**Fig. 4 (abstract A9).**
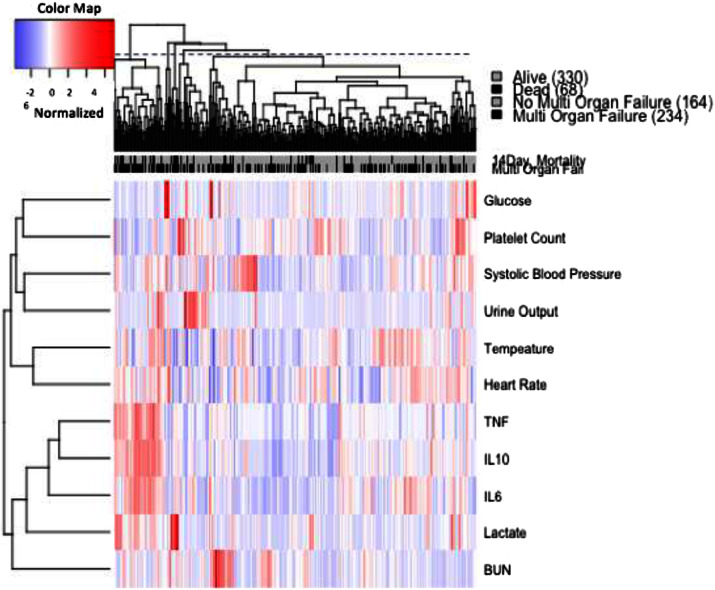
Sepsis endotype heat map

### A10 how sepsis-3 changes previous severe sepsis and septic shock cohorts in respect to mortality rates and length of stay

#### C. Scheer^1^, S.-O. Kuhn^1^, A. Modler^2^, M. Vollmer^3^, C. Fuchs^1^, K. Hahnenkamp^1^, S. Rehberg^1^, M. Gründling^1^

##### ^1^University of Greifswald, Department of Anesthesiology, Greifswald, Germany; ^2^University of Greifswald, Greifswald, Germany, ^3^University of Greifswald, Institute of Bioinfomatics, University Medicine Greifswald, Greifswald, Germany

###### **Correspondence:** C. Scheer – University of Greifswald, Department of Anesthesiology, Greifswald, Germany

**Introduction:** The new sepsis definitions (sepsis-3) are supposed to „offer greater consistency“ for research classification of septic patients [1]. However, it remains to be determined, how sepsis-3 will change the patient population formerly identified by criteria of severe sepsis and septic shock [2].

OBJECTIVE. To investigate the changes in mortality and length of stay data by applying sepsis-3 criteria to a previous patient cohort identified by the former sepsis criteria.

**Methods:** A patient population derived from a previous prospective cohort study performed at the tertiary University Hospital of Greifswald, Germany (ethical approval: BB 133/10) including adult patients with sepsis onset after ICU admission was reanalyzed. Sepsis-3 criteria were applied to the patients previously classified as severe sepsis or septic shock by the consensus conference criteria of 1992.

Mortality rates and length of stay data were compared between severity subgroups defined by the former definitions and sepsis-3, respectively.

**Fig. 5:**
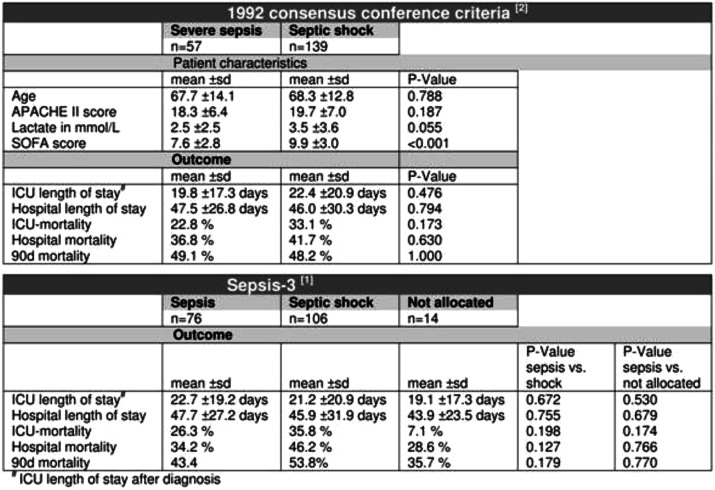
(abstract A10).

**Results:** 196 patients with severe sepsis or septic shock were included. Applying sepsis-3 criteria resulted in 3 subgroups: sepsis, septic shock and patients with neither SOFA increase nor shock. Results are presented in Fig. 5.

**Conclusions:** Sepsis-3 criteria identified patients with a higher risk of mortality. However, results were not significant. Further investigations are needed to determine the impact of sepsis-3 on sepsis classification. Interpreting future research using sepsis-3 should carefully consider potential changes when comparing the results of future trials with previous studies.

**References**

(1) Singer M et al. *JAMA* 2016

(2) Bone R et al. *Chest* 1992

## FLUID MANAGEMENT

### A11 Passive leg raising (PLR) induced carotid flow time (FTC) change can predict fluid responsiveness in mechanically ventilated (MV) patients

#### A. Taggu^1^, N. Darang^2^

##### ^1^St. Johns Medical College, Bangalore, India; ^2^Yashoda Hospials, Secundrabad, India

###### **Correspondence:** A. Taggu – St. Johns Medical College, Bangalore, India

**Introduction:** Fluid administration to maintain organ perfusion is a common practice in critically ill patients. Reliable assessment of fluid responsiveness is difficult. Carotid artery flow is easy to measure bedside.

**Objectives:** To test the hypothesis that changes in carotid artery flow time (corrected flow time, FTc) induced by PLR can predict volume responsiveness in MV patients.

**Methods:** A prospective observation study conducted in MV patients admitted in a mixed ICU between 1st Nov.2013 to 31st Decemeber 2015. All adult patients considered eligible for fluid resuscitation as decided by the attending physician were included. Exclusion criteria: Pregnant patients, head injury , intra-abdominal hypertension, amputees, those with rhythm and known cardiac abnormalities.

**Protocol:** Linear transducer with pulse wave doppler was used on common carotid artery for recording carotid artery FTc. Flo trac (Vigileo Edwardlife sciences-TM) monitor was used to detect changes in stroke volume (SV). All recordings taken at baseline in semi-recumbent positions. Patients were put supine and after 5 minutes, PLR was done. The maximum SV during the PLR was recorded. Carotid FTc was measured at 1, 2 and 3 minutes. Maximum carotid FTc value was considered. Fluid responsiveness was defined as >15 % in SV after volume expansion (VE).

**Results:** Total of 260 patients were taken for the study. Mean age was 58 .5(sD11.2 )years; Male:Female 190:70, BMI (kg/m2) 22.4 (sd 4.2), mean APACHE II score 19 (sD 4.8) with mean mechanical ventilation days of 11.4 (sD 8.2) and mean ICU days of 12.6 (sD9.4). Disease types : 62.5 % Respiratory, 18.5 % Acute febrile illness, 12.50 % CNS and 6.25 % with pancreatitis. Fluid responsiveness were seen in 56.25 % with changes in stroke volume >15 % after VE. Change in Carotid FTc of 24.4 % in response to PLR to predicted volume responsiveness-Sensitivity was 76.8 %, Specificity 89.5 % and ROC Curve of 0.872 (95 % CI 0.56- 1.0).

**Conclusions:** The Carotid FTc increase of 24.4 % during PLR is a reliable predictor of fluid responsiveness in MV patients.

**References**

1. Monnet X, Rienzo M (2005) Esophageal Doppler moni- toring predicts fluid responsiveness in critically ill ventilated patients. Intensive Care Med 31: 1195-201.

2. Dark PM, Singer M(2004) The validity of trans-esophageal Doppler ultrasonography as a measure of cardiac output in critically ill adults. Intensive Care Med 30:2060-6.

**Grant acknowledgement**

None.

**Table 11 (abstract A11).** Baseline characteristics

Parameters	All patients n=260	Responders n=146	Non-responders n=114	p value
Age in years	58.5 (sD 11.2)	58.2 (sD 12.4)	57.9 (sD 10.2)	0.42
Male : Female	145:115	88:58	52:62	0.53
APACHE II	19.0 (sD 4.8)	19.4 (sD 3.9)	19.1 (sD 5.2)	0.84
BMI in Kg/m2	22.4 (sD 4.2)	22.2 (sD 3.2)	23.4 (sD 2.4)	0.47
M V in days	11.4 (sD 8.2)	11.2 (sD 7.4)	11.5 (sD 9.6)	0.85
ICU stay in days	12.6 (sD 9.4)	12.5 (sD 7.5)	12.8 (sD 10.1)	0.89

**Table 12 (abstract A11).** Hemodynamics post PLR and Volume Expansion (VE)

Parameters	Step 1 (base line-recimbent)	Step 2 (PLR-passive leg raising)	P (2, 1)	Step 4 (VE-voulme expansion)	P ( 4, 1)	P (4,2)
RESPONDERS						
HR beats/min	89.4 (sD 19.4)	88.7 (sD 20.4)	0.84	87.8 (sD 19.8)	0.72	0.83
MAP mmHg	68.7 (sD 11.2)	78.4 (sD 11.8)	0.01	83.5 (sD 11.2)	0.001	0.04
SVI ml/m2	34.4 (sD 12.4)	40.1 (sD 18.1)	0.02	45.1 (sD 17.2)	0.005	0.25
FTc millisec	335.15 (sD 23.5)	359.55 (sD 25.46)	0.001	360.52 (sD 26.5)	0.000	0.35
HR beats/min (NON-RESPONDRS)	110.2 (sD 16.4)	115.6 (sD 15.4)	0.76	110.4 (sD 16.8)	0.89	0.82
MAP mmHg	84.6 (sD 14.4)	86.2 (sD 15.7)	0.48	87.3 (sD 14.9)	0.52	0.70
SVI ml/m2	35.4 (sD 11.5)	36.2 (sD 11.6)	0.88	37.2 (sD 11.5)	0.74	0.82
FTc millisec	372.62 (sD 11.7)	381.57 (sD 11.4)	0.03	382.63 (sD 11.6)	0.02	0.74

**Fig. 6 (abstract A11).**
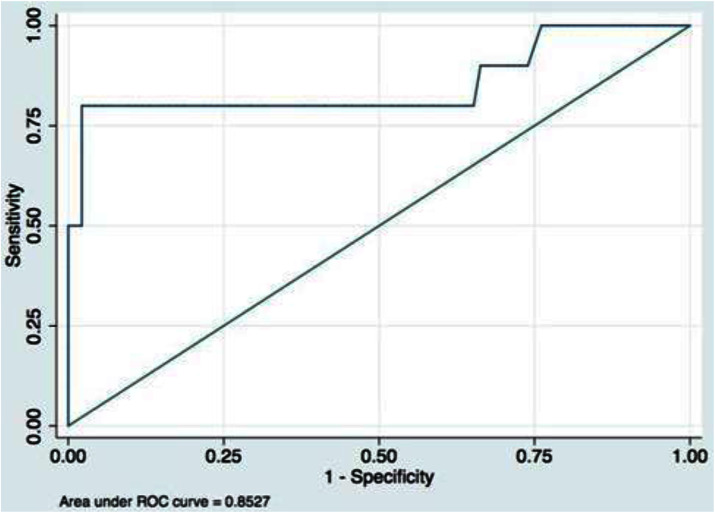
ROC curve for carotid flow time

### A12 Stroke volume targeted resuscitation may be superior to cardiac output or mean arterial pressure based resuscitation in hemorrhagic shock: an animal experiment

#### N. Öveges^1^, I. László^1^, K. Tánczos^1^, M. Németh^1^, G. Lebák^1^, B. Tudor^2^, D. Érces^3^, J. Kaszaki^3^, W. Huber^4^, D. Trásy^1^, Z. Molnár^1^

##### ^1^University of Szeged Faculty of Medicine, Anaesthesiology and Intensive Therapy, Szeged, Hungary; ^2^Medical University of Vienna, Anaesthesiology and General Intensive Care Medicine, Vienna, Austria; ^3^University of Szeged Faculty of Medicine, Surgical Research, Szeged, Hungary; ^4^Technische Universität München, Gastroenterology, Munich, Germany

###### **Correspondence:** N. Öveges – University of Szeged Faculty of Medicine, Anaesthesiology and Intensive Therapy, Szeged, Hungary

**Introduction:** Fluid resuscitation forms the mainstream to ameliorate impaired oxygen delivery during hemorrhagic shock. However, it is a “double-edged-sword” as both over-, and under-filling can be harmful, therefore it should be performed according to adequate physiological end-points. According to a recent survey most physicians still use hypotension as their main target to guide fluid resuscitation (1).

**Objectives:** Our aim was to compare stroke volume (SVI), cardiac output (CI) and mean arterial pressure (MAP) guided fluid resuscitation in a hemorrhagic shock-resuscitation experiment performed on Vietnamese mini pigs.

**Methods:** 39 anaesthetised, mechanically ventilated pigs were randomized into SVI (n = 17), CI (n = 12) and MAP-groups (n = 10). After instrumentation (t_bsl_) the animals were bled till the initial SVI dropped by 50 % (t_0_), and then in each group fluid replacements were performed in 4 equivalent steps (by dividing the t_bsl_-t_0_ values into 4 equal intervals: t_1_-t_4_) by Ringer fundin (RF) solution to reach the baseline values of SVI, CI and MAP, respectively. Invasive hemodynamic measurements and blood gas analyses were undertaken after each step. For statistical analysis General Linear Model, Independent samples T-test and Mann-Whitney U tests were used in SPSS® 23.

**Results:** Similar amounts of blood were drained in all groups (SVI: 17 ± 2, CI: 17 ± 4, MAP: 22 ± 6 ml/kg). There were significant differences among the 3 groups in fluid-replacement ratio: SVI = 3.86 ± 1.21, CI = 2.63 ± 1.31, MAP = 4.51 ± 0.80, p = 0,001. In the SVI-group all variables returned to their baseline or physiological values. Animals in the CI-group remained under-resuscitated as indicated by SVI, central venous oxygen saturation (ScvO_2_), global end diastolic volume (GEDI) and VO_2_/DO_2_ ratio at t_4_ as compared to t_bsl_ (SVI: 31 ± 5 vs. 22 ± 6 ml/m2; ScvO_2_: 79 ± 8 vs. 64 ± 12 %; GEDI: 313 ± 33 vs. 243 ± 47 ml/m2; VO_2_/DO_2_ = 17 ± 10 vs. 47 ± 8 %, p < 0.05, respectively). In the MAP-group most hemodynamic variables (including SVI, CI, pulse pressure variation) returned to their baseline values by t_2_. However, at t_4_ ScvO_2_ and VO_2_/DO_2_ remained significantly lower than at tbsl: ScvO_2_ = 83 ± 5 vs. 64 ± 12 %, VO_2_/DO_2_ = 17 ± 7 vs. 36 ± 14, p < 0.05, respectively. In this group the experiment was terminated when volume replacement exceeded 4.5-times of the drained blood regardless whether the end-point of baseline MAP was reached or not.

**Conclusions:** According to our results, using SVI as goal of resuscitation resulted end-points closest to the physiological baseline values, whilst CI-based resuscitation ended up with under-, and MAP-guided fluid replacement caused over-resuscitation in this experiment. Hence most physicians still use MAP to guide resuscitation, based on these results our practice should carefully be revised.

**References**

1. Cecconi M, Hofer C, Teboul JL, et al. Fluid challenges in intensive care: the FENICE study: A global inception cohort study. Intensive care medicine 2015.

**Grant acknowledgement**

Supported by NKFIH K116689.

### A13 Sublingual and intestinal microcirculatory alterations in hemorrhagic shock and retransfusion

#### G. Ferrara^1^, V.S. Kanoore Edul^1^, H.S. Canales^1^, E. Martins^1^, C. Canullán^1^, G. Murias^1^, M.O. Pozo^1^, J.F. Caminos Eguillor^1^, M.G. Buscetti^1^, C. Ince^2^, A. Dubin^1^

##### ^1^Facultad de Ciencias Médicas, Universidad Nacional de La Plata, Cátedra de Farmacología Aplicada, La Plata, Argentina; ^2^Academic Medical Center, University of Amsterdam, Translational Physiology, Amsterdam, Netherlands

###### **Correspondence:** A. Dubin – Facultad de Ciencias Médicas, Universidad Nacional de La Plata, Cátedra de Farmacología Aplicada, La Plata, Argentina

**Introduction:** Reperfusion injury plays a major role after the resuscitation of hemorrhagic shock. Microcirculatory alterations, however, have not been completely described in this setting.

**Objectives:** To characterize the sublingual and intestinal microcirculation during hemorrhagic shock and retransfusion.

**Methods:** We performed a progressive bleeding followed by retransfusion in anesthetized and mechanically ventilated sheep (n = 10). We also studied a sham group (n = 7). Sublingual and intestinal microcirculation were quantitatively assessed by means of SDF-videomicroscopy. Measurements were performed at baseline (B), first and last step of hemorrhage (H1 and H2), and 30' after retransfusion (R).

**Results:** During hemorrhagic shock, all microcirculatory variables were affected. Blood reinfusion restored intestinal mucosal total and perfused vascular density, and red blood cell velocity (Panels A, B, and D). Proportion of perfused vessels, microvascular flow index, and heterogeneity flow index (Panels C, E, and F) remained altered. A similar pattern was observed in sublingual mucosa except for red blood cell velocity, which was reduced.

**Conclusions:** Reperfusion microvascular injury was mainly characterized by decreased perfusion velocity and increased heterogeneity. In contrast, vascular density was preserved. Sublingual microcirculatory changes mirrored those from intestinal mucosa.

**Grant acknowledgement**

Supported by the grant PICT201000495, Agencia Nacional de Promoción Científica y Tecnológica, Argentina.

**Fig. 7:**
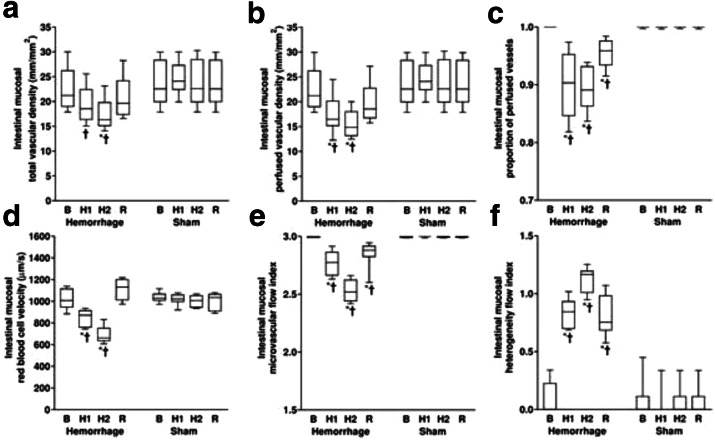
(abstract A13).

### A14 Quantification of stressed volume and systemic vascular compliance in septic and post-cardiac surgical patients after a fluid challenge

#### H.D. Aya^1,2^, A. Rhodes^1,2^, N. Fletcher^1,2^, R.M. Grounds^1^, M. Cecconi^1,2^

##### ^1^St George's University Hospitals NHS Foundation Trust, Adult Critical Care Directorate, London, United Kingdom; ^2^St George's, University of London, London, United Kingdom

###### **Correspondence:** H.D. Aya – St George's University Hospitals NHS Foundation Trust, Adult Critical Care Directorate, London, United Kingdom

**Introduction:** Septic patients may require large amount of intravascular fluid in the initial resuscitation. Intravascular volume can be divided into unstressed and stressed volume (V_s_). V_s_ represents the volume haemodynamically active. The systemic vascular compliance (C_sys_) quantifies the relationship between the change in volume per unit of pressure in the compliance vessels.

**Objectives:** The aim of this study is to compare the C_sys_ and V_s_ between patients after cardiac surgery and septic patients after a fluid challenge.

**Methods:** Patients admitted to the intensive care unit were monitored with invasive arterial blood pressure, a calibrated LiDCO*plus* (LiDCO, UK). Mean systemic filling pressure (Pmsf-arm) was measured using the stop-flow arterial-venous equilibrium method [1]. A fluid challenge of 4 - 5 mL/kg of Hartmann´s solution was performed over 5 minutes. Csys was calculated as Δvolume/ΔPmsf-arm. V_s_ was calculated multiplying C_sys_ times Pmsf-arm at the end of the fluid challenge. Sepsis was defined by the presence of at least 2 systemic inflammatory response syndrome criteria and strong suspicion or evidence of infection. Data are presented as median and interquartile range, and compared using Mann-Whitney U test. *p* values less than 0.05 were considered statistically significant.

**Results:** 18 septic and 19 post-cardiac surgery patients were included in the study. C_sys_ in septic patients was 160.0 (95.8, 240.6) mL/mmHg while in post-cardiac surgical patients was 86.4 (64.7, 144.0) mL/mmHg (*U* = 237.5, *p* = .04). V_s_ was 2785 mL (1951, 5625) in septic patients and 2312 mL (1810, 4650) in post-cardiac surgical patients. There is no evidence of differences in V_s_ (*U* = 195, *p* = .46) between septic and post-cardiac surgical patients.

**Conclusion:** C_sys_ is twice greater in septic patients compared to cardiac surgical patients but V_s_ is similar between these two groups.

**References**

1. Aya HD, Rhodes A, Fletcher N, Grounds RM, Cecconi M (2015) Transient stop-flow arm arterial-venous equilibrium pressure measurement: determination of precision of the technique. J Clin Monit Comput.

**Fig. 8 (abstract A14).**
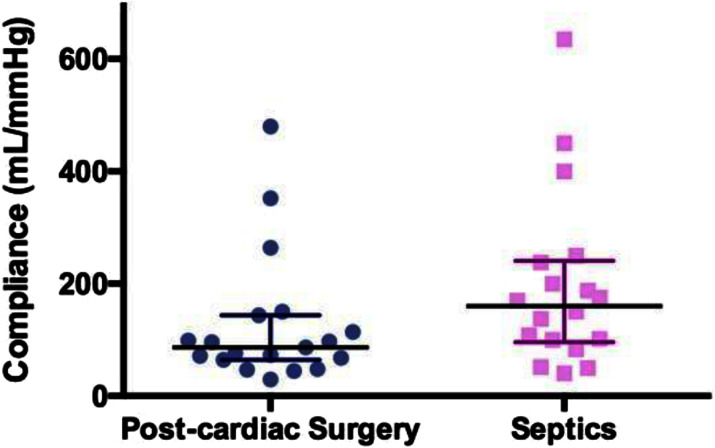
Csys

**Fig. 9 (abstract A14).**
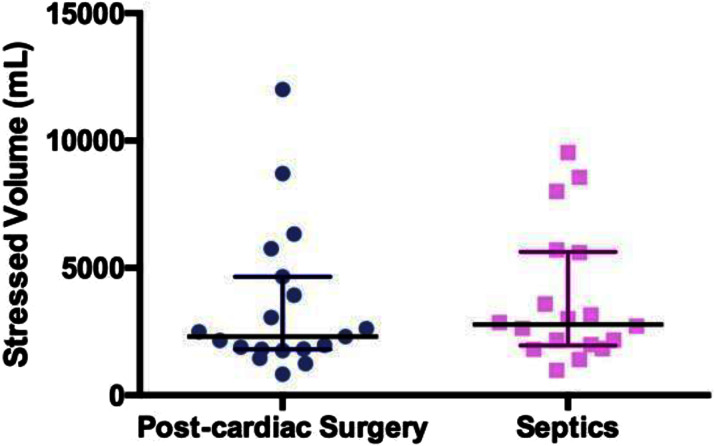
Vs

### A15 Capillary refill time variation during a passive leg raising can predict the effect of a 500 ml fluid load on tissue perfusion

#### M. Jacquet-Lagrèze^1,2^, M. Riche^1^, R. Schweizer^1,2^, P. Portran^1,2^, W. Fornier^1^, M. Lilot^3^, J. Neidecker^1^, J.-L. Fellahi^1,2^

##### ^1^Hôpital Louis Pradel, Anesthésie Réanimation, Bron, France; ^2^University Claude Bernard Lyon 1, Lyon, France; ^3^Hôpital Femme Mère Enfant, Lyon, France

###### **Correspondence:** M. Jacquet-Lagrèze – Hôpital Louis Pradel, Anesthésie Réanimation, Bron, France

**Introduction:** No method is currently available to predict the effect of a fluid challenge on tissue perfusion. Capillary refill time (CRT) has a prognostic value and can assess tissue perfusion during septic shock (1). Passive leg raising simulates a fluid load with the great advantage to be reversible.

**Objectives:** Our hypothesis is that the variation of a digitalized capillary refill time during a passive leg raising could predict the variation of the CRT after a 500 ml fluid load.

**Methods:** Hemodynamic variables [CRT, systolic, diastolic and mean arterial pressure (PAS, PAD, PAM), heart rate (HR), cardiac index (CI) by transpulmonary thermodilution] were recorded, before and after a passive leg raising. And after a 500 ml fluid load. Capillary refill time was recorded with a video camera after a calibrated compression of the skin using a piston. Four CRT acquisition was made at each hemodynamic condition and a posteriori analyzed by two readers ( MR and MJL). The least significant change of the measure was 25 %. Consequently, responders (CRT-R) were defined as patients showing a reduction of CRT after the fluid load of at least 25 %. Others were defined as CRT-NR. Variations were calculated after passive leg raising and after the fluid load. CI responsiveness was define as an increase of 15 % of the CI after the fluid load. Spearman correlation test was used. Mann-Whitney-Wilcoxon tests were used to compare nonparametric independent variables. ROC curve was build and area under the curve were calculated (AUCROC). Confidence intervals (CI) were calculated using a 1000 repetitions bootstrap. The best threshold was determined using the Youden index.

**Results:** Our institution review board (Comité de Protection des Personnes Lyon Sud-Est III) approved the study protocol, 29 patients with circulatory failure were recruited. 11 patients (38 %) were CRT-R, 18 CRT-NR. Passive leg raising induced CRT variations decreased by -41 % (-30 ;-48) in CRT-R and by -4.5 % (-10 ; 19) in CRT-NR (p < 0.001). Correlation between CRT variation after passive leg raising and after the fluid load was r = 0.74 (p < 0.001). AUCROC was 0.93 (CI: 0.83 ; 1.0). The best threshold was -27 %. Sensitivity was 73 (CI: 45-100)%. Specificity was 100 (CI:100-100)% . Among the 13 patients diagnosed with a CI responsiveness, seven patients were also CRT-R.

**Conclusions:** Our method enables a precise and reproducible measurement of the CRT and the detection of a moderate variation of the CRT. Digitalized CRT variation during a passive leg raising predict CRT variation after a fluid load. Our method is, in our knowledge, the first to predict the effect of a fluid load on tissue perfusion.

**References**

(1) Intensive Care Med 2014 40 (7) : 958-64

(2) Am J Respir Crit Care Med 2002 Jul 1 ; 166 (1) : 98-104.

## ANTBIOTIC STEWARDSHIP IN THE ICU

### A16 Factors associated with no de-escalation therapy in critically ill patients

#### A. Escoresca-Ortega, A. Gutiérrez-Pizarraya, L. Charris-Castro, Y. Corcia-Palomo, E. Fernandez-Delgado, J. Garnacho-Montero

##### Hospital Virgen del Rocio, Intensive Care Unit, Sevilla, Spain

###### **Correspondence:** A. Escoresca-Ortega – Hospital Virgen del Rocio, Intensive Care Unit, Sevilla, Spain

**Introduction:** Adequate empiric antimicrobial therapy is crucial in terms of survival in patients with severe infections. However, the use of broad-spectrum antimicrobial treatment is not without its drawbacks.

**Objective:** The aim of this study was to evaluate factors associated with no de-escalation therapy in a cohort of patients admitted to the ICU with severe sepsis.

**Methods:** Prospective and observational study of patients admitted to the ICU with severe sepsis or septic shock January 2008 to July 2013. Appropriate cultures were obtained before initiating broad spectrum antimicrobial therapy and supportive measures were performed following Surviving Sepsis Campaign guidelines. Modification of the antimicrobial regimen after culture results was left at the decision of physician in charge of the patient. The following variables were recorded: demographic characteristics, underlying diseases, severity of illness at admission (APACHE II and SOFA scores), adequacy of empirical antimicrobial therapy, SOFA score at the day of culture results, worst SOFA score in the ICU, development of nosocomial infection, leght of stay and mortality. We also analyzed clinical characteristics, sepsis source, presence of bacteremia and type of pathogen. We used Student´s T test, Mann-Whitney U test or Chi square (as appropriate) to compare the variables. A multivariate analysis was also performed to control for confounding variables in order to asses the factors associated with no de-escalation.

**Results:** Eight hundred and fifty two patients were enrolled and de escalation therapy was performed on two hundred and sixty seven (31,3 %). The median APACHE II and SOFA scores at admission were 18 (13-24) 7 (5-10) respectivily. Up to 648 (76,1 %) patient of total cohort had microbiological documentation. The multivariate analysis showed that the variables associated with not de-escalation therapy were SOFA score the day of culture results (OR 0,942 IC 95 %), previous antimicrobial therapy (OR 0,555 IC 95 %), liver SOFA score (OR 0,213 IC 95 %), pulmonar focus (OR 0,491 IC 95 %) and abdominal focus of infection (OR 0,500 IC 95 %). Blood cultures possitives, microbiological documentation and combination of antibiotic therapy were independent factors associated with de-escalation therapy.

**Conclusions:** De-escalation is a feasible strategy in critically ill patients admitted to the ICU with microbiological documentation, blood cultures positive and those with combination of antibiotic therapy.

**References**

1.- Garnacho-Montero J, Gutiérrez-Pizarraya A, Escoresca-Ortega A, et al. Deescalation of empirical therapy is associated with lower mortality in patients with severe sepsis and septic shock. Intensive Care Med 2014; 40:32-40.

2.- Leone M, Bechis C, Baumstarck K, et al. Deescalation versus continuation of empirical antimicrobial treatment in severe sepsis: a multicenter non-blinded randomized noninferiority trial. Intensive Care Med 2014; 40:1399-1408.

### A17 Influence of renal replacement therapy modalities on population pharmacokinetics of amikacin in critically ill patients undergoing continuous veno-venous haemofiltration and haemodiafiltration

#### C. Roger^1,2^, L. Muller^1^, L. Elotmani^1^, J. Lipman^2,3^, J.Y. Lefrant^1^, J.A. Roberts^2,3^

##### ^1^Nimes University Hospital, Department of Anaesthesiology, Critical Care, Pain and Emergency Medicine, Nimes, France; ^2^University of Queensland, Burns Trauma Critical Care Research Centre, Brisbane, Australia; ^3^Royal Brisbane & Womens Hospital, Department of Intensive Care Medicine, Brisbane, Australia

###### **Correspondence:** L. Muller – Nimes University Hospital, Department of Anaesthesiology, Critical Care, Pain and Emergency Medicine, Nimes, France

**Introduction:** Few data are available to guide amikacin dosing regimens in critically ill patients undergoing continuous renal replacement therapy.^1^ The aim of the study was to describe amikacin pharmacokinetics during continuous veno-venous haemofiltration (CVVH) and continuous veno-venous haemodiafiltration (CVVHDF). We also used Monte Carlo simulations to determine the optimal dosing regimens.

**Methods:** Patients receiving amikacin and undergoing CVVH or CVVHDF were eligible. Blood samples were collected at ten sampling times during a dosing interval and assay using a validated LC-MS/MS method. Population pharmacokinetic analysis and Monte Carlo simulation were undertaken using Pmetrics.

**Results:** Sixteen patients (four patients received both CVVH and CVVHDF) were included and twenty sampling profiles (9 CVVH, 11 CVVHDF) were analysed. A two-compartment linear model best described the data. Patient weight was the only covariate that was associated with drug clearance. The mean (SD) parameter estimates were 25.2 ± 17.3 L for central volume, 0.89 ± 1.17 L/h for rate constant for drug distribution from the central to the peripheral compartment, 2.38 ± 6.60 L/h for rate constant for drug distribution from the peripheral to the central compartment, 4.45 ± 2.35 L/h for haemodiafiltration clearance and 4.69 ± 2.42 for haemofiltration clearance. After accounting for patient weight, CVVH and CVVHDF clearance were 5.19 (±0.74) L/h and 4.08 (±0.50) L/h (p = 0.21), respectively. Dosing simulations for amikacin supported the use of high dosing regimens (≥25 mg/kg) and extended intervals (36 to 48 h) for most patients to achieve the pharmacodynamics/pharmacokinetics target of peak/MIC ≥ 8 for efficacy and the lowest probability of target attainment (PTA) of a minimal concentration ≥ 2.5 mg/L for toxicity.

**Conclusions:** A strategy of extended-interval high doses of amikacin (25 mg/kg 48-hourly) associated with therapeutic drug monitoring (TDM) should be the best approach for aminoglycoside administration in critically ill patients receiving continuous renal replacement therapy.

**References**

1. Taccone FS, de Backer D, Laterre PF, et al. Pharmacokinetics of a loading dose of amikacin in septic patients undergoing continuous renal replacement therapy. Int J Antimicrob Agents 2011;37(6):531-535.

**Grant acknowledgement**

Nimes University Hospital grant.

**Fig. 10 (abstract A17).**
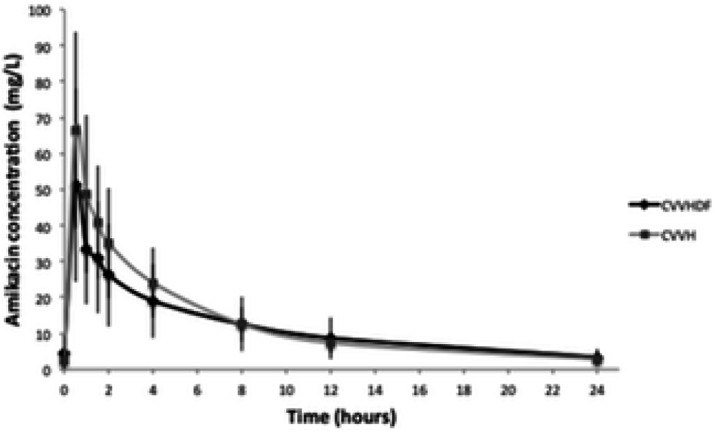
Ciprofloxacin concentration-time profiles

**Fig. 11 (abstract A17).**
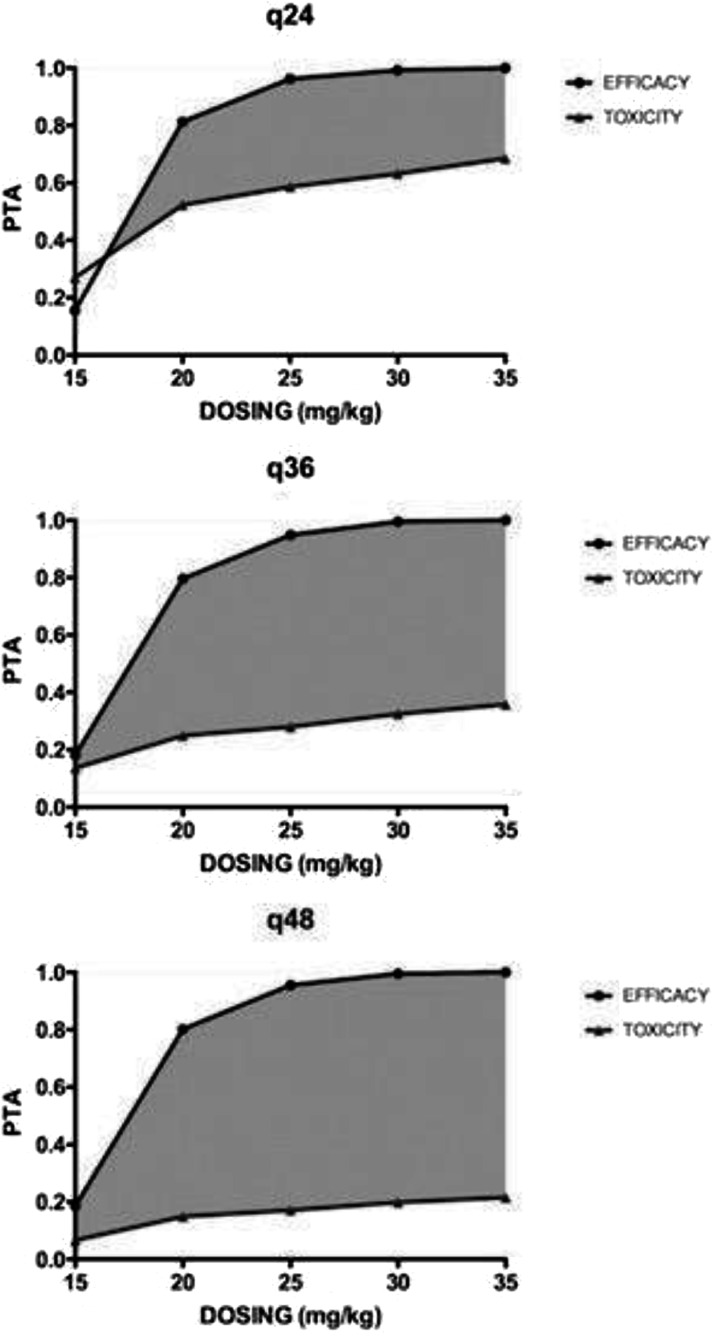
Probability of target attainment efficacy/toxicity

### A18 Clinical factors associated with out of range therapeutic concentrations of linezolid in critical patients admitted to the ICU

#### R. Muñoz-Bermúdez^1^, M. Samper^1^, C. Climent^1^, F. Vasco^1^, V. Sara^1^, S. Luque^2^, N. Campillo^2^, S. Grau Cerrato^2,3^, J.R. Masclans^1,4,5^, F. Alvarez-Lerma^1,3^

##### ^1^Hospital del Mar-IMIM, Critical Care, Barcelona, Spain; ^2^Hospital del Mar, Pharmacy, Barcelona, Spain; ^3^Universidad Autónoma de Barcelona, Barcelona, Spain; ^4^Universidad Pompeu Fabra, Barcelona, Spain; ^5^CIBERES. Instituto de Salud Carlos III, Madrid, Spain

###### **Correspondence:** R. Muñoz-Bermúdez – Hospital del Mar, Pharmacy, Barcelona, Spain

**Introduction:** Linezolid (LNZ) is a widely used antibiotic in critically ill patients as a treatment of infections in which it is certain or suspected the presence of Gram-positive cocci methicillin or vancomycin resistant (CGP -MR).

**Objectives:** To identify clinical factors associated with subtherapeutic concentrations of LNZ (defined as plasma concentrations [Cmin] < 2 ug / ml) or with its overexposure (defined as plasma concentrations [Cmin] > 7.5 ug / ml).

**Methods:** A prospective, observational and descriptive study in one ICU. All patients who received intravenous LNZ (60-minutes infusion) at doses of 600 mg every 12 hours, for treatment of a suspected or confirmed CGP-MR infection in which it was possible to obtain a blood sample (5 ml) after the third day (steady state) for the determination of the Cmin, just before administering the next dose, were included. Patients under renal replacement therapy were excluded. Demographic variables, comorbidities, severity on admission and analytical data were evaluated. LNZ quantification was performed using a high performance liquid chromatography technique (HPLC). Differences between groups were assessed using chi-square for categorical variables and Student's t-test or Mann-Whitney test for continuous variables. Significant variables in the univariate analysis were included in a multivariate model (logistic regression) to identify the variables related to sub/supratherapeutic levels. We considered p < 0.05 to be significant. The predictive value of each formula was calculated using a receiver operator characteristic curve (ROC), and the area under the curve (AUC) was also computed.

**Results:** A total of 103 patients were included. In 58 of them (56.3 %) the C_min_ was < 2 ug / ml and in 30 (29.1 %) the C_min_ was >7.5 ug / ml. Glomerular filtration rate (OR 1.01, 95 % CI 1.00- 1.02; p = 0.002) and diabetes mellitus (OR 0.24, 95 % CI 0.07-0.83; p = 0.02) were independently variables associated with subtherapeutic concentrations. The discrimination ability model obtained an AUC (95 % CI) of 0.808 (0.72 to 0.89). Liver cirrhosis (OR 14.51, 95 % CI 1.35-156.40; p = 0.03) and glomerular filtration rate (OR 0.98, 95 % CI 0.97 to 0.99 p = 0.002) were independently variables associated with supratherapeutic concentrations. The discrimination ability model obtained an AUC (95 % CI) 0.81 (0.72 to 0.898).

**Conclusions:** Variables related to sub and supra-therapeutic LNZ concentrations in critically ill patients admitted to the ICU have been identified. Presence of these variables would require the determination of plasma levels of LNZ. Glomerular filtration rate is the variable that influences both groups.

### A19 Validation of a checklist for high risk for multirresistant bacteria according to the “resistencia zero” project

#### S. Carvalho Brugger, G. Jimenez Jimenez, M. Miralbés Torner, J. Trujillano Cabello, B. Balsera Garrido, X. Nuvials Casals, F. Barcenilla Gaite, M. Vallverdú Vidal, M. Palomar Martínez

##### Hospital Universitari Arnau de Vilanova, Lleida, Spain

###### **Correspondence:** S. Carvalho Brugger – Hospital Universitari Arnau de Vilanova, Lleida, Spain

**Introduction:** Multirresistant bacteria (MRB) development is a growing phenomenon. In 2013, the “Zero Resistance” (RZ) program was launched in Spain, to help prevent the emergence of MRB in critically ill patients. One of its recommendations is to complete a checklist upon patient admission in Intensive Care Unit (ICU) to identify those patients at high risk for colonization or infection by MRB.

**AIMS.** To analyse the effectiveness of the checklist for risk factors (RF) proposed by the RZ project as a way of MRB early detection.

**Methods:** A prospective study from March/2014 to January/2016. All patients admitted to a polyvalent ICU of a general hospital were submitted to the checklist proposed, with the application of contact precaution (CP) strategies for patients with RF for MRB. Bacteriologic swabs (nasal, pharyngeal, axillary and rectal) were routinely performed on all patients admitted, besides diagnostic cultures when necessary. Furthermore, we analysed other pathological variables and comorbidities (diabetes, renal failure, immunosuppression state, neoplasia, cirrhosis, chronic obstructive pulmonary disease -COPD-, organ transplantation, malnutrition or type of admission to ICU - urgent or programmed). Univariate and multivariate analysis of RF for MRB with binary logistic regression were performed. Statistical significance was set at CI 95 %.

**Results:** 1651 patients were admitted. 532 (32,2 %) met some CP criteria. In 136 (8,2 %) were detected one or more MRB, 87 of these (64 %) presented CP criteria according to the checklist. 37 met 1 criteria, 31 met 2 criteria and 19 met 3 or more criteria with accumulation of risk (p < 0,001). In 49 (36 %) MRB carriers it was not identified any of the RF from the checklist. Tables 13 and 14 show risk factors and comorbidities that were significant as added risk for MRB.

**Conclusion:** After comparing to previous analysis, it was seen that, increasing the sample size, almost all RF included in the RZ checklist were predictors of MRB. Despite this, it could not detect 36 % of the patients infected or colonized by MRB. Because of that, we should consider other factors to predict the presence of a MRB on admitted patients to ICU.

**Reference**

J Montero et al (Scientific Expert Committee for the “Zero Resistance” Project). Combatting resistance in intensive care: the multimodal approach of the Spanish ICU “Zero Resistance” program. Critical Care (2015) 19:114.

**Table 13 (abstract A19).** Risk Factors for MRB

RZ CHECK LIST	OR (IC 95%) UNIVARIATE	OR (IC 95%) MULTIVARIATE
Hospitalization >5 days in past 3 months	3,1 (2,1-4,4)	1,7 (1,1- 2,6)
Institutionalized patient	4,2 (2,1-8,6)	3,0 (1,3- 6,9)
Prior MRB colonization/ infection	17,8 (9,4-33,8)	11,3 (5,5-23,2)
Antibiotherapy >7 days in the past month	4,2 (2,9-6,2)	2,1 (1,3- 3,6)
Chronic kidney disease with dialysis	2,0 (0,4-9,3)	NS
Colonization susceptibility (bronchiectasis, cystic fibrosis)	2,4 (1,0-5,4)	NS

**Table 14 (abstract A19).** Relation between comorbidities and MRB

COMORBIDITY	OR (IC 95%) UNIVARIATE	OR (IC 95%) MULTIVARIATE
DM type II	1,5 (1,1-2,2	NS
COPD	1,9 (1,3-3,0)	NS
Malnutrition	1,8 (1,2-2,6)	NS
Cirrhosis	3,9 (2,1-7,1)	2,7 (1,4-5,4)
APACHE II >25	2,46 (1,6-3,8)	2,2 (1,4-3,6)

### A20 Influence of antibiotic stewardship program on etiology and clinical outcomes in bacteriemic patients in a multidisciplinary surgical hospital

#### V. Gusarov^1^, D. Shilkin^1^, M. Dementienko^1^, E. Nesterova^1^, N. Lashenkova^2^, A. Kuzovlev^1^, M. Zamyatin^1^

##### ^1^N.I. Pirogov National Medical Surgical Center, Anestesiology and Intensive Care, Moscow, Russian Federation; ^2^N.I. Pirogov National Medical Surgical Center, Microbiology Laboratory, Moscow, Russian Federation

###### **Correspondence:** V. Gusarov – N.I. Pirogov National Medical Surgical Center, Anestesiology and Intensive Care, Moscow, Russian Federation

**Introduction:** Development of candidemia and bacteremia caused by multidrug-resistant (MDR) pathogens in hospitalized patients significantly increases the risk of adverse outcome [1-3]. The main task of the antibiotic stewardship program (ASP) is to reduce the microbial resistance and decrease incidence of infectious complications caused by MDR pathogens.

**Objectives:** To estimate the etiological structure and clinical outcome in patients with bacteremia in a multidisciplinary hospital in the period before and after the implementation of ASP.

**Methods:** Interventional study with historical controls. The intervention: ASP, including a group of experts on antimicrobial therapy (AMT), AMT and prophylaxis protocols, infection control, internal audit. Study onset: June 2013. Analysis of pre-intervention (Jan. 2011-June 2013) and intervention (July 2013-Dec. 2015) periods was carried out. We assessed incidence of bacteremia caused by methicillin-resistant *S. aureus* (MRSA), vancomycin-resistant *Enterococci* (VRE), ESBL-producing *Enterobacteriaceae*, MDR Gram-negative non-fermenting bacteria (MDR-NFGB), incidence of candidemia*.* The hospital length of stay (LOS) and mortality of patients with bacteremia were secondary end-points.

**Results:** In the pre-intervention period 2905 blood samples were analyzed, 448 (15.4 %) positive blood cultures from 206 patients were obtained; in the interventional period 2742 blood samples were analyzed, 470 (17.1 %) positive blood cultures from 230 patients were obtained.

In the intervention period a significant decrease in the incidence of bacteremia caused by MDR-NFGB from 55 (12.3 %) to 20 (4.3 %), p < 0.001, VRE from 10 (2.2 %) to 0, p = 0.0011 and incidence of candidemia from 29 (6.5 %) to 13 (2.85), p < 0.01, was detected. The frequency of bacteremia caused by ESBL-producing *Enterobacteriae* did not significantly change [91 (20.3 %) vs. 108 (23.0 %), p > 0.05]. Number of MRSA bacteremias remained low [6 (1.3 %) vs. 4 (0.9 %), p > 0.05]. There was a significant reduction of mortality from 30.6 % to 20.3 %, p < 0.025 in the group of patients with bacteremia. The LOS did not change significantly [29 (IQI 19-52) vs. 27 (IQI 17-53), p > 0.05].

**Conclusions:** Implementation of ASP in multidisciplinary surgical hospital allows significantly decrease incidence of candidemia and bacteremia caused by MDR-NFGNB and VRE. As a result - reduction in mortality in patients with bacteremia.

**References**

1. Bassetti M., Merelli M., Ansaldi F. et al. Clinical and Therapeutic Aspects of Candidemia: A Five Year Single Centre Study. PLoS One. 2015;10(5): e0127534.

2. Tam V., Rogers C., Chang K. et al. Impact of multidrug resistant Pseudomonas aeruginosa bacteremia on patient outcomes. Antimicrob Agents Chemother 2010;54(9):3717-22.

3. Blot S., Vandewoude K., Colardyn F. Nosocomial bacteremia involving Acinetobacter baumannii in critically ill patients: a matched cohort study. Intensive Care Med. 2003 Mar;29(3):471-5.

## ADJUNCTIVE INTERVENTIONS IN ACUTE RESPIRATORY FAILURE

### A21 Impact of earplugs and eye mask on sleep in critically ill patients: a prospective polysomnographic study

#### A. Demoule, S. Carreira, S. Lavault, O. Palancca, E. Morawiec, J. Mayaux, I. Arnulf, T. Similowski

##### Pitie-Salpetriere Hospital and Pierre and Marie Curie University, Paris, France

###### **Correspondence:** A. Demoule – Pitie-Salpetriere Hospital and Pierre and Marie Curie University, Paris, France

**Rationale:** Poor sleep is common in intensive care unit (ICU) patients and environmental factors contribute to sleep deprivation and alterations of sleep architecture. The objective of the present study was to evaluate the impact of earplugs and eye masks on sleep architecture in ICU patients.

**Patients and methods:** A single center randomized controlled trial of 64 ICU patients was conducted from July 2011 to December 2013. Patients were randomly assigned to sleep with or without earplugs and a facemask. A polysomnography was performed on the first day and night following inclusion. The primary end point was the proportion of sleep stage 3 + 4. Secondary end points were other descriptors of sleep and major outcome variables.

**Results:** In the intervention group, 33 % of patients did not wear earplugs all night long. The proportion of sleep stage 3 + 4 was 11 [3-23]% in the control group and 13 [6-23]% in the protective group (p = 0.72). Other descriptors of sleep were not different between the two groups except the number of long awaking that was lower in the protective group than in the control group (21 [19-26] vs. 31 [21-47], p = 0.02). There was no difference among the two groups in terms of sleep quality, occurrence of a delirium, ICU length of stay and mortality, anxiety and depression on ICU discharge and day-90 and the incidence of post-traumatic stress disorder.

**Conclusion:** In ICU patients, earplugs and eye mask are not well accepted by patients, do not increase the proportion of sleep stage 3 + 4 but decrease the number of prolonged awakenings. They had no impact on the outcome.

**Grant acknowledgement**

French Ministry of Health (PHRC)

**Note:** This abstract has been previously published and is available at [1]. It is included here as a complete record of the abstracts from the conference.

**References**

1. Demoule A, Carreira S, Lavault S, Pallanca O, Morawiec E, Mayaux J, Arnulf I, Similowski T (2016) Impact of earplugs and eye mask on sleep in critically ill patients: a prospective randomized polysomnographic study. Annals of Intensive Care 6(Suppl 1): S9

### A22 Metabonomics identifies early molecular changes associated with progression into postoperative hypoxemia in cardiac surgery patient: a human model that can provide new insights into the pathophysiology of acute lung injury and potentially identify specific biomarkers of lung tissue injury

#### B.S. Rasmussen^1,2^, R.G. Maltesen^2^, M. Hanifa^2^, S. Pedersen^2,3^, S.R. Kristensen^2,3^, R. Wimmer^4^

##### ^1^Aalborg University Hospital, Department of Anaesthesia and Intensive Care Medicine, Aalborg, Denmark; ^2^Aalborg University, Department of Clinical Medicine, Aalborg, Denmark; ^3^Aalborg University Hospital, Department of Clinical Biochemistry, Aalborg, Denmark; ^4^Aalborg University, Department of Chemistry and Bioscience, Section of Biotechnology, Aalborg, Denmark

###### **Correspondence:** B.S. Rasmussen – Aalborg University Hospital, Department of Anaesthesia and Intensive Care Medicine, Aalborg, Denmark

**Introduction:** Postoperative pulmonary dysfunction after cardiac surgery with the use of cardiopulmonary bypass (CPB) is common, ranging from transient hypoxemia to acute respiratory distress syndrome (ARDS). It is triggered by an inflammatory response, disrupted coagulation, ischemia-reperfusion injury, and oxidative stress. However, as the nadir of partial pressure of arterial oxygen (PaO_2_) appears on the second to third postoperative day, it is of paramount importance to identify patients at risk at an early stage. Determining the progression into hypoxemia is challenging, as no early diagnostic test exists. We hypothesized that metobonomics can provide new insights into the pathogenesis of pulmonary dysfunction, thus potentially enabling the identification of specific biomarkers of lung injury.

**Objectives:** To use a human model of cardiac surgery to study the progression into postoperative hypoxemia by means of blood metabonomics.

**Methods:** Fifty consecutive patients undergoing cardiac surgery were included. Arterial blood samples were collected the day before surgery, and 48 and 72 hours (h) postoperatively. In addition, samples from the pulmonary artery and the left atrium were collected at seven different time points: just before CPB, straight after CPB (0 h), and 2, 4, 8, 20, 48, and 78 h after CPB. Samples were analyzed by nuclear magnetic resonance (NMR) spectroscopy. Statistical methods, including principal component analysis (PCA), partial least-square regression (PLS) and discriminant analysis (PLS-DA), were applied to find metabolite patterns related to surgical trauma and postoperative hypoxemia. Venetian-Blinds cross-validation and permutation testing were used for validation.

**Results:** All patients had a normal preoperative PaO_2_; 11.3 ± 1.3 kPa (mean ± SD). PaO_2_ decreased to 7.9 ± 1.5 kPa at 48 h, and to 7.8 ± 1.4 kPa at 72 h, respectively. Twenty-three patients developed moderate hypoxemia (PaO_2_ ≤ 8 kPa) and 9 patients had severe hypoxemia (PaO_2_ ≤ 6 kPa). NMR showed major shifts in the metabolome during and after surgery. Different metabolites were identified at different timepoints, of which several were directly correlated to the progression into hypoxemia. PLS-DA modeling predicted postoperative hypoxemia at 72 h with 72.2 % sensitivity and 81.0 % specificity based on blood samples collected just before CPB, while PLS models revealed a correlation of r^2^ equal to 0.7. Higher predictive values (>88 % sensitivity, >92 % specificity, and r^2^ > 0.91) were achieved when analyzing blood collected 0, 2, 4, 8, and 20 h postoperatively, demonstrating the ability of metabonomics in early diagnosis.

**Conclusion:** We found that metabonomics may contribute to the detection of early signs of pulmonary dysfunction two-three days before significant postoperative hypoxemia. The study provides novel insights into the underlying mechanisms that trigger progression into hypoxemia, facilitating new hypotheses and treatment options.

### A23 Multicenter randomized clinical trial of lateral-trendelenburg vs. semi recumbent position for the prevention of ventilator-associated pneumonia - the gravity-VAP trial

#### M. Panigada^1^, G. Li Bassi^2^, O.T. Ranzani^2^, T. Kolobow^3^, A. Zanella^1^, M. Cressoni^1^, L. Berra^4^, V. Parrini^5^, H. Kandil^6^, G. Salati^7^, S. Livigni^8^, A. Amatu^9^, A. Andreotti^10^, F. Tagliaferri^11^, G. Moise^12^, G. Mercurio^13^, A. Costa^14^, A. Vezzani^15^, S. Lindau^16^, J. Babel^17^, M. Cavana^18^, D. Consonni^1^, A. Pesenti^1^, L. Gattinoni^19^, A. Torres^2^, for the GRAVITY-VAP TRIAL NETWORK

##### ^1^Policlinico Di Milano, Milan, Italy; ^2^Hospital Clinic, Barcelona, Spain; ^3^National Institutes of Health, Bethesda, United States; ^4^Massachusetts General Hospital, Boston, United States; ^5^Ospedale Nuovo del Mugello, Borgo San Lorenzo (FI), Italy; ^6^Gruppo Ospedaliero San Donato, San Donato M.se, Italy; ^7^Arcispedale S. Maria Nuova, Reggio Emilia, Italy; ^8^Ospedale San Giovanni Bosco, Torino, Italy; ^9^Policlinico San Matteo, Pavia, Italy; ^10^Policlinico di Modena, Modena, Italy; ^11^Azienda Ospedaliero - Universitaria di Parma, Parma, Italy; ^12^Ospedale Città di Sesto San Giovanni, Sesto San Giovanni, Italy; ^13^Policlinico Gemelli, Roma, Italy; ^14^. Azienda Ospedaliero-Universitaria di Parma, Parma, Italy; ^15^Azienda Ospedaliero-Universitaria di Parma, Parma, Italy; ^16^University Hospital Frankfurt, Frankfurt, Germany; ^17^University Hospital Center Zagreb, Zagreb, Croatia; ^18^Ospedale Santa Chiara, Trento, Italy; ^19^University of Göttingen, Göttingen, Germany

###### **Correspondence:** M. Panigada – Policlinico Di Milano, Milan, Italy

**Introduction:** Gravity plays a pivotal role in the pathogenesis of ventilator-associated pneumonia (VAP) (1). In previous laboratory studies (2) the semi-lateral Trendelenburg position (LTP) hindered gravity-driven pulmonary aspiration and avoided VAP.

**Objectives:** To determine whether the LTP vs. the semi-recumbent position (SRP) would reduce the incidence of microbiologically confirmed VAP and to appraise patient's compliance and safety.

**Methods:** We conducted a randomized, single-blind, controlled study in 17 European centers and 1 in North America. A total of 2019 adult patients were screened between 2010 and 2015. 395 patients were randomized - 194 in LTP and 201 in SRP - and analyzed in an intention to treat approach. Patients in LTP were placed in semi-lateral (60°) - Trendelenburg position to achieve an orientation, from the sternal notch toward the mouth, slightly below horizontal, and turned from one side to the other every 6 hours. LTP was encouraged during the first days of mechanical ventilation, but always in compliance with the patient's wish. In the SRP group, the head of the bed was elevated ≥ 30°. Primary outcome was VAP incidence rate, based on quantitative bronchoalveolar lavage fluid culture with ≥ 10^4^ colony-forming units/mL. Secondary outcomes were compliance to the randomized position, length of intubation, duration of intensive care unit and hospital stay, mortality, and adverse events.

**Results:** The trial was stopped after the planned interim analysis for achieving efficacy endpoints and owing to safety concerns. Patients in the LTP and SRP group were kept in the randomized position for 38 % and 90 % of the study time, respectively (p = 0.001). Yet, during the first 48 hours, LTP patients were kept in the randomized position for 50 % of the study time, and SRP patients for 88 % (p = 0.001). In the LTP, the bed was angulated 5.6° in Trendelenburg; while, the head of the bed was elevated 34.1° in the SRP group. Incidence rates of microbiologically confirmed VAP were 0.88 (1/1136 patient-days; 95 % confidence interval [CI], 0.12-6.25) in the LTP group, and 7.19 (8/1113 patient-days; CI 95 %, 3.60-14.37) in the SRP (p = 0.020), relative risk reduction of 0.12 (95 % CI, 0.01-0.91). No statistically significant differences were observed in durations of mechanical ventilation, intensive care unit and hospital stay, and mortality. Vomiting was more common in LTP patients (8.3 % vs. 2.5 % in the SRP, p = 0.013).

**Conclusions:** Critically ill patients positioned in the LTP had a statistically significant reduction in the incidence of VAP, compared with those positioned in the SRP. A comprehensive evaluation of potential LTP contraindications is warranted to enhance safety.

**References**

1) Li Bassi G et al. *Crit Care Med* 2014; 42: e620-7

2) Zanella et al. *Intensive Care Med* 2012; 33: 677-85

**Grant acknowledgement**

ClinicalTrials.gov ID: NCT01138540. The study was endorsed by ECCRN/ESICM. Funding by 2013 ECCRN Clinical Trial Award and Hill-Rom

### A24 The effects of oral rinse with 0.2 % and 2 % chlorhexidine on oropharyngeal colonization and ventilator associated pneumonia in adults' intensive care units

#### P. Mansouri^1^, F. Zand^2^, L. Zahed^3^, F. Dehghanrad^3^, M. Bahrani^3^, M. Ghorbani^3^

##### ^1^School of Nursing, Shiraz University of Medical Science, Shiraz, Islamic Republic of Iran; ^2^Shiraz University of Medical Sciences, Anesthesiology and Critical Care Research Center, Shiraz, Islamic Republic of Iran; ^3^Shiraz University of Medical Sciences, Shiraz, Islamic Republic of Iran

###### **Correspondence:** F. Zand – Shiraz University of Medical Sciences, Anesthesiology and Critical Care Research Center, Shiraz, Islamic Republic of Iran

**Introduction:** Ventilator Associated Pneumonia (VAP) is the most common nosocomial infection in Intensive Care Units (ICUs), which increases the length of ICU stay, duration of mechanical ventilation, and mortality.

**Objectives:** The present study used an oral care protocol and compared the effects of two different concentrations of chlorhexidine on reduction of oropharyngeal colonization and VAP.

**Methods:** This study was performed on 114 patients from trauma, surgery, neurosurgery, and general ICUs randomly allocated to two groups under oral care with 0.2 % and 2 % chlorhexidine solution. A multidisciplinary team approved the oral care protocol. The data were collected using a demographic information form, Apache IV form, Beck oral assessment scale, mucosal-plaque assessment scale, and oropharyngeal swab culture.

**Results:** The results showed a significant reduction in VAP (p = 0.007) and oropharyngeal colonization (p = 0.007) in the group under oral care with 2 % chlorhexidine solution compared to the other group. However, no significant difference was found between the two groups in terms of oropharyngeal adverse effects (p = 0.361).

**Conclusions:** Oral decontamination with 2 % compared to 0.2 % chlorhexidine is a more effective method in prevention of VAP and reduction of oropharyngeal colonization (especially gram-positive).

**Grant acknowledgement**

This study supported by a grant (grant No. 7363) from Vice-chancellor in Research, Shiraz University of Medical Sciences

### A25 Acute consequences of lobar unilateral pulmonary perfusion block in an animal model of high tidal volume ventilation and lavage

#### B. Cambiaghi^1^, O. Moerer^2^, T. Mauri^3,4^, N. Kunze-Szikszay^2^, C. Ritter^2^, A. Pesenti^5^, M. Quintel^2^

##### ^1^University of Milan - Bicocca, Monza, Italy; ^2^University of Goettingen, Goettingen, Germany; ^3^Fondazione IRCCS Ospedale Maggiore Policlinico, Anesthesia and Critical Care, Milan, Italy; ^4^Plug Working Group, ESICM, Milan, Italy; ^5^University of Milan, Milan, Italy

###### **Correspondence:** B. Cambiaghi – University of Milan - Bicocca, Monza, Italy

**Introduction:** Previous studies showed that, in the presence of preserved ventilation, interruption of regional pulmonary blood flow rapidly leads to severe ventilation-induced lung injury (VILI) [1-2]. These data suggest that tissue alkalosis might be a multiplication factor for VILI [3], especially in the presence of high tidal volume (Vt) ventilation and/or pre-existing lung injury.

**Objectives:** We investigated the alterations in blood gases, respiratory mechanics and CT-scan imaging in an animal model of controlled unilateral lobar pulmonary artery occlusion followed by lung lavage and high Vt ventilation.

**Methods:** We report data from 20 pigs (weight 61 ± 2 Kg). Animals were randomly assigned to the following 5 study groups: 1. Five animals underwent right lower lobar endovascular embolization, lung lavage with 500 ml of normal saline solution and high Vt ventilation for 8 hours; 2. Four animals underwent left lower lobar embolization, lavage and high Vt ventilation; 3. Five pigs received lung lavage and high Vt ventilation; 4. Three animals received right lower lobar embolization and low Vt ventilation; 5. Three animals received only low Vt ventilation. In groups 1, 2 and 4 selective embolization of the pulmonary artery branch perfusing the lower lobe of the right or left lung was performed under fluoroscopy guidance by expert radiologist; in group 1-3, Vt was set at 20-25 ml/Kg with plateau pressure ≤40 cmH_2_O and peak pressure ≤50 cmH_2_O, without positive end-expiratory pressure (PEEP) and respiratory rate of 7-9 breaths/minute to control pH. In groups 3 and 5, Vt was 6-8 ml/Kg with PEEP 5 cmH_2_O. After 8 hours, the animals underwent physiologic data collection and chest CT scan at zero PEEP. CT images were analysed offline by custom-made software. Data were analysed by one-way ANOVA and Tukey post-hoc tests.

**Results:** Main parameters describing pathophysiological characteristics of each group after 8 hours are reported in Tables 15 and 16. Lung lavage and high Vt ventilation following regional embolization led to more severe impairment of oxygenation (p < 0.001), especially in the left embolization group. Lung mechanics at the end of the experiment showed similar trend: plateau pressure was higher and respiratory system compliance lower (p < 0.05) in the embolization + lavage + high Vt groups. Finally, CT-scan revealed higher lung weight in groups 1 and 2 (p < 0.05). Interestingly, in the same groups, the non-embolized lungs were heavier, as if embolization triggered more severe ventilation-induced lung oedema that could develop mainly where perfusion was preserved.

**Conclusions:** Regional block of lung perfusion might amplify lung injury due to lavage and high Vt ventilation.

**References**

[1] Slutsky AS *N Engl J Med* 2013

[2] Kolobow T *Inter J Artif Organs* 1981

[3] Ando T *J Int Med Res* 2007

**Grant acknowledgement**

Departmental fundings.

**Table 15 (abstract A25).** Ventilation pattern and gas exchange

	Right embolization + lavage + high Vt (n= 5)	Left embolization + lavage + high Vt (n= 4)	Lavage + high Vt (n= 5)	Right embolization + low Vt ventilation (n= 3)	Low Vt ventilation (n=3)	P-value ANOVA
Vt (ml/kg)	20.1 ± 2.5°#	18.7 ± 2.7°#	23.5 ± 2.3*°#	8.1 ± 0.2	8.0 ± 0.3	<0.001
PEEP (cmH_2_O)	0 ± 0°#	0 ± 0°#	0 ± 0°#	5 ± 0	5 ± 0	<0.001
PaO_2_/FiO_2_	460 ± 95*	102 ± 74	489 ± 121*	477 ± 7*	568 ± 10*	<0.001
PaCO_2_ (mmHg)	35.8 ± 4.3*	52.5 ± 10.1	34.2 ± 10.3*	41.3 ± 3.0	41.3 ± 5.0	<0.05
pH	7.52 ± 0.05	7.39 ± 0.09	7.55 ± 0.09	7.50 ± 0.02	7.51 ± 0.03	<0.05

*p <0.05 vs. left emb. + lavage + high Vt; ° p <0.05 vs. right emb. + low Vt; #p <0.05 vs. low Vt; all by Tukey test

**Table 16 (abstract A25).** Severity of lung injury

	Right embolization + lavage + high Vt (n= 5)	Left embolization + lavage+ high Vt (n= 4)	Lavage + high Vt (n= 5)	Right embolization + low Vt (n= 3)	Low Vt (n=3)	P-value ANOVA
Paw_Plat_ (cmH_2_O)	36 ± 9°#	43 ± 4°#	38 ± 4°#	18 ± 1	19 ± 2	<0.001
Crs (ml/cmH_2_O)	34 ± 6	26 ± 6	39 ± 8*	38 ± 2	38 ± 5	<0.05
CT-scan total lung tissue (g)	222 ± 124	369 ± 160	193 ± 166	43 ± 5	117 ± 28*	<0.05
CT-scan right lung tissue (g)	103 ± 58	270 ± 119	101 ± 88	27 ± 2*	90 ± 31	<0.001
CT-scan left lung tissue (g)	119 ± 68	98 ± 44	91 ± 78	15 ± 3	26 ± 3	0.09

*p <0.05 vs. left emb. + lavage + high Vt; °p <0.05 vs. right emb. + low Vt; #p <0.05 vs. low Vt; all by Tukey test

## EXPERIMENTAL AKI: THE MICROCIRCULATION AND PERFUSION

### A26 Genetic variants in apoptosis pathway genes *BCL2* and *SERPINA4* are not associated with septic acute kidney injury

#### L.M. Vilander^1^, M.A. Kaunisto^2,3^, S.T. Vaara^1^, V. Pettilä^1^, FINNAKI Study Group

##### ^1^University of Helsinki and Helsinki University Hospital, Intensive Care Medicine, Helsinki; Finland; ^2^Institute for Molecular Medicine Finland (FIMM), University of Helsinki, Helsinki, Finland; ^3^Folkhälsan Institute of Genetics, Folkhälsan Research Center, Helsinki, Finland

###### **Correspondence:** L.M. Vilander – University of Helsinki and Helsinki University Hospital, Intensive Care Medicine, Helsinki; Finland

**Introduction:** Acute kidney injury (AKI) is a multifactorial syndrome, but knowledge regarding its pathophysiology and possible genetic background is limited. Recently the first hypothesis free genetic association studies have been published to explore the individual susceptibility to AKI.

**Objectives:** We aimed to replicate the previous associations of candidate polymorphisms (N = 5) with development of AKI (1) using a prospectively collected cohort of septic critically ill patients in Finland.

**Methods:** We included all septic patients with genetic samples among the 2968 FINNAKI study patients. After exclusion of 401 patients (due to underlying chronic kidney disease, lack of DNA or genotyping quality) 837 (of the remaining 2567) had sepsis. AKI was defined according to the KDIGO criteria, considering stages 2 and 3 affected and KDIGO 0 unaffected.

The genotyping was done using iPLEX™ Assay (Agena Bioscience). The genotyped SNPs were rs8094315 and rs12457893 in the intron of the *BCL2* gene, rs2093266 in the *SERPINA4* gene, rs1955656 in the *SERPINA5* gene and rs625145 in the *SIK3* gene. Association analyses were performed using logistic regression with PLINK software.

**Results:** We found no significant associations between SNPs rs8094315 (OR 1.10, p 0.4827), rs12457893 (OR 1.02, p 0.8736), rs2093266 (OR 0.77, p 0.1525), rs1955656 (OR 0.77, p 0.1525) and rs625145 (OR 0.89, p 0.3967) and AKI comparing 354 AKI patients and 299 non-AKI patients (Table 17). AKI patients were significantly older, had higher BMI, more often diabetes and COPD, had received more often colloids before admission to ICU, and had lower platelet count. Multivariate logistic regression analysis with adjustment for sex and these baseline differences did not change our findings.

**Conclusions:** We could not confirm the previously reported associations between five SNPs and development of AKI KDIGO 2/3 in critically ill septic patients.

**References**

1. Frank AJ, Sheu CC, Zhao Y, et al: BCL2 genetic variants are associated with acute kidney injury in septic shock*. *Crit Care Med* 2012;40:2116-2123

**Grant acknowledgement**

This study has been supported by grants TYH 2013343 and 2016243 from the Helsinki University Hospital research funding (V.P.) and a grant from Sigrid Juselius Foundation (V.P.).

**Table 17 (abstract A26).** Associations in univariate and multivariate analysis between SNPs and AKI

SNP	Gene	Genotype available	Major allele/Minor allele	Minor Allele Frequency^a^	Univariate *p*	Adjusted *p* ^b^
rs625145	*SIK3*	653	A/T	0.20/0.21	0.3967	0.4455
rs1955656	*SERPINA5*	653	G/A	0.10/0.12	0.1525	0.1981
rs2093266	*SERPINA4*	653	G/A	0.10/0.12	0.1525	0.1981
rs8094315	*BCL2*	650	A/G	0.24/0.22	0.4827	0.5187
rs12457893	*BCL2*	653	A/C	0.37/0.37	0.8736	0.7715

^a^Cases/controls, ^b^Adjusted for age, BMI, diabetes, COPD, receiving colloids pre ICU, minimum platelet count and sex

### A27 Renal resistive index (RRI) as an early predictor and discriminator of acute kidney injury (AKI) in critically ill patients: a prospective observational cohort study

#### J.L.G. Haitsma Mulier, S. Rozemeijer, A.M.E. Spoelstra-de Man, P.E. Elbers, P.R. Tuinman, M.C. de Waard, H.M. Oudemans-van Straaten

##### VU Medical Centre, Intensive Care Adults, Amsterdam, Netherlands

###### **Correspondence:** J.L.G. Haitsma Mulier – VU Medical Centre, Intensive Care Adults, Amsterdam, Netherlands

**Introduction:** Acute kidney injury (AKI) is a severe complication of critical illness, often accompanied by vasoconstriction in the renal arteries and microcirculation. Its diagnosis is mainly based on a rise in serum creatinine, which is a late phenomenon. The Renal Resistive Index (RRI), measured with Doppler ultrasound, represents a new non-invasive diagnostic tool assessing flow resistance in the renal circulation, early after admission.^(1)^

**Objectives:** To determine whether RRI measured on intensive care unit (ICU) admission is an early predictor and discriminator of AKI development and severity, developing within the first week after ICU admission, and if RRI predicts AKI independently of other AKI risk factors.

**Methods:** Prospective observational cohort study in ICU patients. AKI development and stage were defined by the KDIGO criteria. To increase the likelihood of including patients developing AKI, two cohorts of patients were included within 24 h after ICU admission: Patients with shock and without shock. Patients with eGFR < 30 ml/min were excluded. Besides routine ICU measurements, including risk factors for developing AKI, three study measurements were performed at inclusion: RRI, sublingual sidestream dark field imaging (SDF) to visualise microcirculation, and Bioelectrical Impedance Analysis (BIA) as a marker of fluid status. Uni- and multivariate and ROC curve analyses were performed to assess predictive and discriminative value of RRI and other variables for the development of AKI.

**Fig. 12 (abstract A27).**
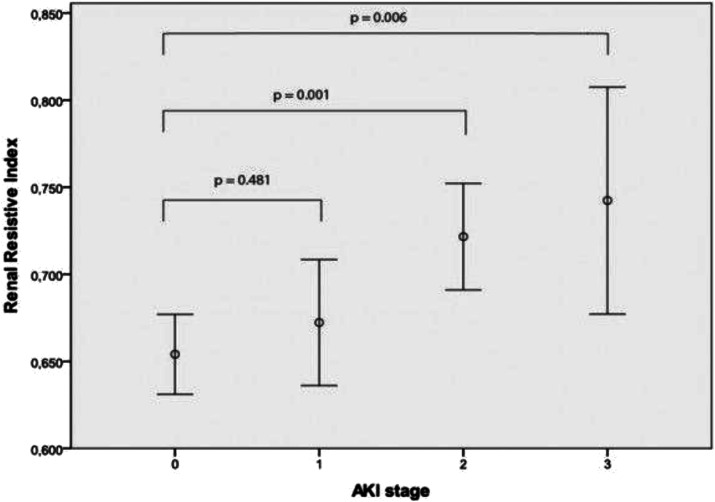
RRI for each AKI stage (mean+95% CI)

**Results:** We included 99 patients, mean age 65, mean APACHE III score 73. 49 patients (49 %) developed AKI within the first week (mean 2.2 days after inclusion). Patients who developed AKI had a significantly higher RRI on inclusion than those who did not: 0.708 (0.687-0.730) vs 0.654 (0.631-0.677), p = 0.001. Compared to patients without AKI, RRI was significantly higher in patients with AKI stage 2 and 3, but not in patients with AKI stage 1 (Fig. 12).

**Fig. 13 (abstract A27).**
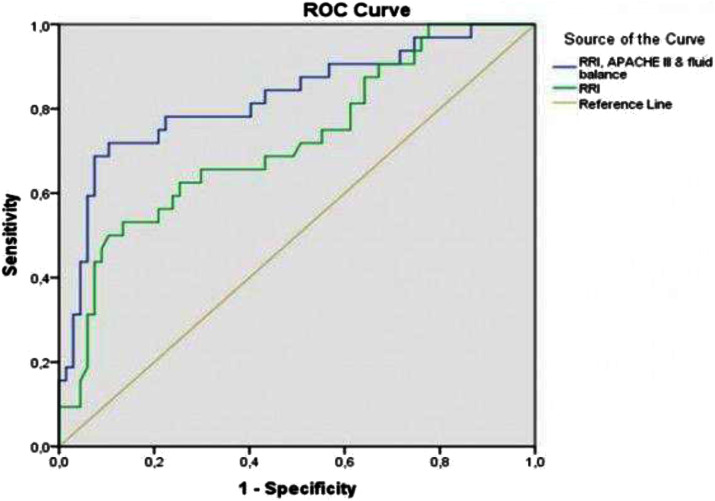
ROC curves for AKI stage 2 and 3

We therefore chose AKI stage 2 and 3 as endpoint in further analysis. On univariate analysis, RRI was a significant predictor of AKI (OR 1.012, 95 % CI 1.006-1.019), along with other parameters including: APACHE III, fluid balance and BIA derived reactance, but not sublingual microcirculation. On multivariate analysis, RRI, APACHE III and fluid balance remained significant. The AUC of RRI for AKI stage 2 and 3 was 0.721 (95 % CI 0.612-0.831). The composite AUC of the multivariate predictors was 0.825 (Fig. 13).

**Conclusions:** In this observational study, RRI on ICU admission was a significant independent early predictor for development of AKI stage 2 and 3 during the first week, but not for AKI stage 1. Of all evaluated predictors, APACHE III, as a marker of severity of disease, and fluid balance were additional independent predictors of AKI, suggesting that other factors than vasoconstriction contribute to AKI development.

**Reference**

1 Darmon et al. *Intensive Care Med* 2011;37(1):68-76

**Grant acknowledgement**

No grants received

### A28 Post-sepsis dysfunction of kidney microcirculation and of parenchyma by SDF and histology. An experimental study

#### A.M.A. Liberatore^1^, R.B. Souza^2^, A.M.C.R.P.F. Martins^3^, J.C.F. Vieira^1^, I.H.J. Koh^1^

##### ^1^Federal University of São Paulo, Surgery, São Paulo, Brazil; ^2^Universidade Federal de São Paulo, Morphology and Genetics, São Paulo, Brazil, ^3^Biological Institute of São Paulo, São Paulo, Brazil

###### **Correspondence:** A.M.A. Liberatore – Federal University of São Paulo, Surgery, São Paulo, Brazil

**Introduction:** Post-sepsis syndrome describe the group of long-term problems that some people with sepsis experience. Acute kidney injury (AKI) is associated with increased short-term mortality of septic patients; however, the exact influence of AKI on long-term mortality has not yet been determined. Herein, we sought to evaluate the impact of sepsis on kidney structures of survivors.

**Methods:** Adult Wistar rats (200 g) were submitted to sepsis [iv. 2 mL *E. coli* 10^8^ (S8) or 10^9^ CFU/ mL (S9), DL60 and DL80, respectively, in 26 hours].Under general anesthesia, the microcirculation of the renal cortex area was monitored by Sidestream Dark Field Imaging (SDF) video-microscopy, at 6 hours (T6h, n = 3/group) and 30 days (T30d, n = 3/group) after sepsis. The kidney samples were evaluated by histology (T0, T6h, T30d) using H.E and PAS dyes.

**Fig. 14:**
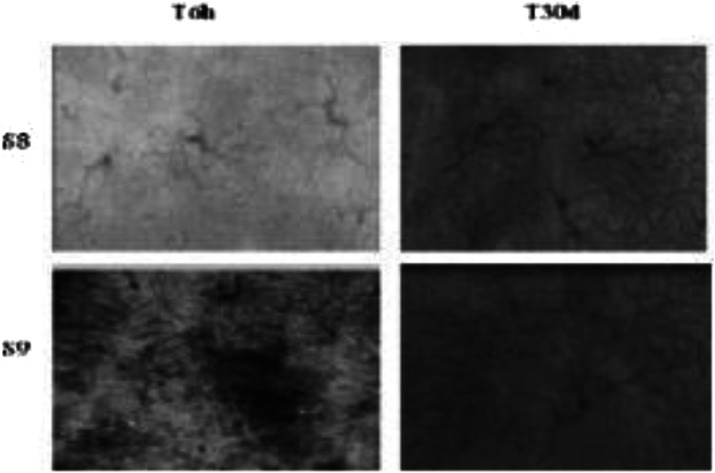
(abstract A28).

**Fig. 15:**
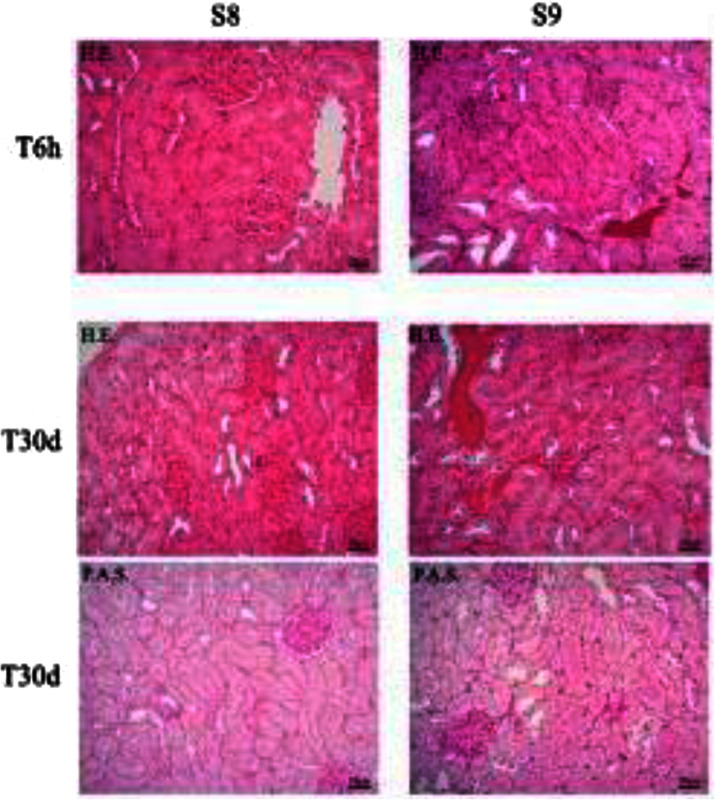
(abstract A28).

**Results:** SDF findings (Fig. 14) of S8-T6h showed broadly distributed microcirculation dysfunction. The outlining of tubules became blurred by their enlargement leading to the compression of tubular lumen and of the peritubular microvessels, suggesting an ongoing obstructive phenomenon in a progressive manner by tubular wall edema. The heterogeneous dysfunction pattern was disseminated in entire screen. S9-T6h showed similar findings, however more intensely. The histology (Fig. 15) confirmed the ongoing obstructive phenomena at S8-T6h, and also showed generalized peritubular microvessels congestion, multiple glomerulus without mesangial area, inflammatory infiltrate in the connective tissue and hyaline degeneration suggestive of ongoing cellular dysfunction/death. The histological results of S9-T6h showed similar pattern with the enlargement of the epithelial cells of convoluted tubules with reduced lumen, and with compressed or dilated peritubular microvessels. In addition, numerous tubular epithelial cells showed membrane injury, nuclear pycnosis and necrosis. At S8-T30d, although the general findings were better, as compared to the early phase sepsis (T6h), the histological findings demonstrated a persistence of significant peritubular and glomerular congestions with intense inflammatory infiltrate in the connective tissues, nucleus contractions suggestive of cell death, and collecting ducts injuries. PAS staining demonstrated widespread hyaline degeneration. The more severe sepsis, S9-T30d group, showed a widespread congestion around the collecting tubules and peritubular congestion of the small and large vessels. Also was observed glomerulus without mesangial areas, intense congestion and hyaline degeneration of the collecting tubules and of the cortex tubules region. The general findings were of the ischemic renal injury pattern. These findings showed that renal dysfunction persists at 30 days after sepsis, and that the presence of any pathological stimuli, may decay the renal physiological capacity quickly, justifying the fragility of renal physiology in patients who survived sepsis.

**Grant acknowledgement**

FAPESP 2011/20401-4.

### A29 Cardiac and renal effects of remote ischaemic preconditioning in diabetic patients undergoing cardiac angiography

#### M. Galindo Martínez^1^, R. Jiménez Sánchez^1^, L. Martínez Gascón^2^, M.D. Rodríguez Mulero^1^, A. Ortín Freire^1^, A. Ojados Muñoz^1^, S. Rebollo Acebes^1^, Á. Fernández Martínez^1^, S. Moreno Aliaga^3^, L. Herrera Para^4^, J. Murcia Payá^1^, F. Rodríguez Mulero^5^

##### ^1^Hospital Santa Lucía, Intensive Care, Cartagena, Spain; ^2^Hospital Santa Lucía, Análisis Clínicos, Cartagena, Spain; ^3^Hospital Santa Lucía, Cartagena, Spain; ^4^Hospita Santa Lucía, Intensive Care, Cartagena, Spain; ^5^School of Medicine Murcia, Department of Physiology, Murcia, Spain

###### **Correspondence:** R. Jiménez Sánchez – Hospital Santa Lucía, Intensive Care, Cartagena, Spain

**Introduction:** Remote ischemic preconditioning (RIPC), has been demonstrated beneficial effects on acute kidney injury (AKI) and/or cardiac function, after coronary angiography (CA), in high risk patients. However, the protective effects by RIPC may be attenuated in diabetic patients

**Objectives:** The purpose of this study was to evaluate the effects of RIPC on the myocardial and renal function as measured by plasma Troponin I and proBNP, and creatinine and cystatin C levels (as an early marker of glomerular function), before and after CA in diabetic patients.

**Methods:** A prospective randomized, single centre pilot trial was conducted at University Hospital Sta. Lucia, Spain. We analyzed 50 diabetic adult patients with acute coronary syndrome admitted to a medical intensive care unit, with a diagnosis of diabetes before admission, undergoing standard CA.

We established two groups: standard CA with remote RPIC (RIPC group) trough intermittent upper-arm ischemia before CA, or without RIPC (control group). We recorded demographical, clinical, and analytical data of the patients, before and after 72 hours of procedure. We analyzed qualitative variables with percentages by categories and square Chi; quantitative variable with mean, standard deviation and T Student; or median, interquartile rank and U Mann Whitney; or regression, depending on the statistical distribution.

**Results:** Patients receiving RIPC showed lower troponin I peak (ng/ml) 3.3 (0.98-13.5) compared to control group 18 (1.76-31.15); p = 0.053; and the NT-proBNP (pg/ml) increasing after 72 hours was -946 (-2977—20); compared to control group 73 (-426- 2210); p = 0.005. Regarding to renal function, basal and after 72 hours, creatinine and cystatine-C plasma levels were similar in both groups. The creatinin (mg/dl) increments after 24 hours were (RIPC; 0(-14-10; control -5.5(-16-8) p > 0.05 and cystacin C (mg/L) RIPC, 3 (-7-11) and control -4 (-9-13) p > 0.05.

The calculated mean Mehran score for both groups in the present study was8 in RIPC group and 8.5 (control group), thus determining the present study population as a group at medium risk of developing AKI. No adverse effects were related to RIPC maneuver in any patient.

**Conclusions:** The RIPC maneuver in diabetic patients before CA demonstrated beneficial on cardiac function without affecting renal parameters. The mechanisms underlying these effects merit further investigation

**References**

1. Jensen, R.V., et al., *Impact of O-GlcNAc on cardioprotection by remote ischaemic preconditioning in non-diabetic and diabetic patients.* Cardiovasc Res, 2013. **97**(2): p. 369-78.

Sivaraman, V., et al., *Preconditioning the diabetic human myocardium.* J Cell Mol Med, 2010. **14**(6B): p. 1740-6.

2. Tsang, A., et al., *Preconditioning the diabetic heart: the importance of Akt phosphorylation.* Diabetes, 2005. **54**(8): p. 2360-4.

### A30 A novel led-based phosphorimeter for measurement of microcirculatory oxygen concentrations in vivo in the kidney

#### P. Guerci^1,2,3^, Y. Ince^1^, P. Heeman^4^, B. Ergin^1^, C. Ince^1^

##### ^1^Academic Medical Center, University of Amsterdam, Department of Translational Physiology, Amsterdam, Netherlands; ^2^University Hospital of Nancy, Departement of Anaesthesiology and Critical Care Medicine, Vandoeuvre-Les-Nancy, France; ^3^University of Lorraine, INSERM U1116, Vandoeuvre-Les-Nancy, France; ^4^Academic Medical Center, University of Amsterdam, Department of Medical Technical Innovation & Development (MIO), Amsterdam, Netherlands

###### **Correspondence:** P. Guerci – Academic Medical Center, University of Amsterdam, Department of Translational Physiology, Amsterdam, Netherlands

**Introduction:** Quantitative measurement of microcirculatory and of tissue oxygen concentration is of prime importance in experimental research. Non-invasive time resolved quenching of Palladium-porphyrin phosphorescence injected into experimental animals has given much fundamental insight in mechanisms of oxygen transport to tissue in health and models of disease. Until now, most of the phosphorimeters used flash lamps as a light excitation source. However, a major drawback of flash lamps is the plasma glow that persists for tens of microseconds after the primary discharge. When a flash lamp produces a pulse, a tail remains causing unwanted further excitation to the initial pulse of the phosphor in a time dependent manner. This generates a complex excitation pulse pattern, which if not taken into account using deconvolution analysis, can lead to inaccurate PO_2_ readings.

**Objectives:** To design and calibrate a new LED-based phosphorimeter (LED-P) that address previous drawbacks of flash-lamp phosphorimeter (FL-P). We validate the LED-P with in vitro and vivo experimental studies

**Methods:** We designed the LED-P using 4 LEDs of different colours (blue, green, yellow, red), each with a narrow-band wavelength (20 nm), providing excitation light pulses within the range of several phosphorescent dyes often use in experimental studies.

We calibrated in vitro the device using the Palladium-porphyrin phosphorescent dye at different temperatures ( 19, 25, 28, 32, 34, 37 and 39 °C) and adjusted pH (6.8, 7.0, 7.2, 7.4, 7.6) with an enzymatic reaction producing step by step decrease of oxygen content and analysing the decay times of phosphorescence (*τ*). The oxygen content was measured with a solid-state polymer optical fiber sensor oxygen optode (POF, Oxy-Mini, World Precision Instruments, USA). In vivo calibration was performed in a rat model by exposing the kidney. We ventilated the animal in different hypoxic conditions (15, 7 and 4 % O_2_) and performed renal ischemia/reperfusion injury by clamping the renal artery.

**Results:** The LED-P exhibited a block shape light pulse without afterglow eliminating the need for deconvolution of the light emission signal coming from the tissue under analysis. A perfect linear regression was observed after plotting decay times ratio (*τ*0/*τ*) and in vitro oxygen concentration (r^2^ = 0.99). In vivo, the LED-P showed similar kidney microvascular PO_2_ compared to previously published works, in hypoxia or ischemia conditions.

**Conclusions:** This new LED-P provides minimally-invasive, accurate and reliable measurements of microvascular PO_2_ in the kidney, without the need of complex mathematical deconvolution of the light emission signal coming from the tissue. LEDs are much cheaper, with a smaller sized and simpler electronics, are more energy efficient, have a longer lifetime. This LED-P provides the ability to choose which LED to enable/disable to excite different phosphorescent dyes within the same measurement.

**Fig. 16 (abstract A30).**
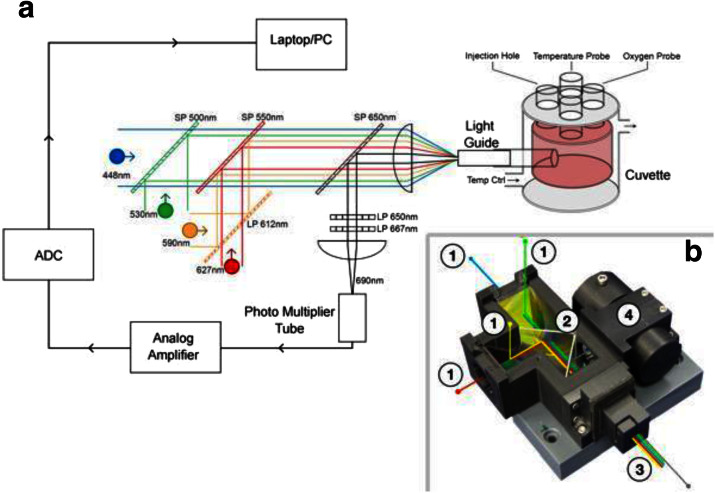
LED phophorimeter schematic drawing

**Fig. 17 (abstract A30).**
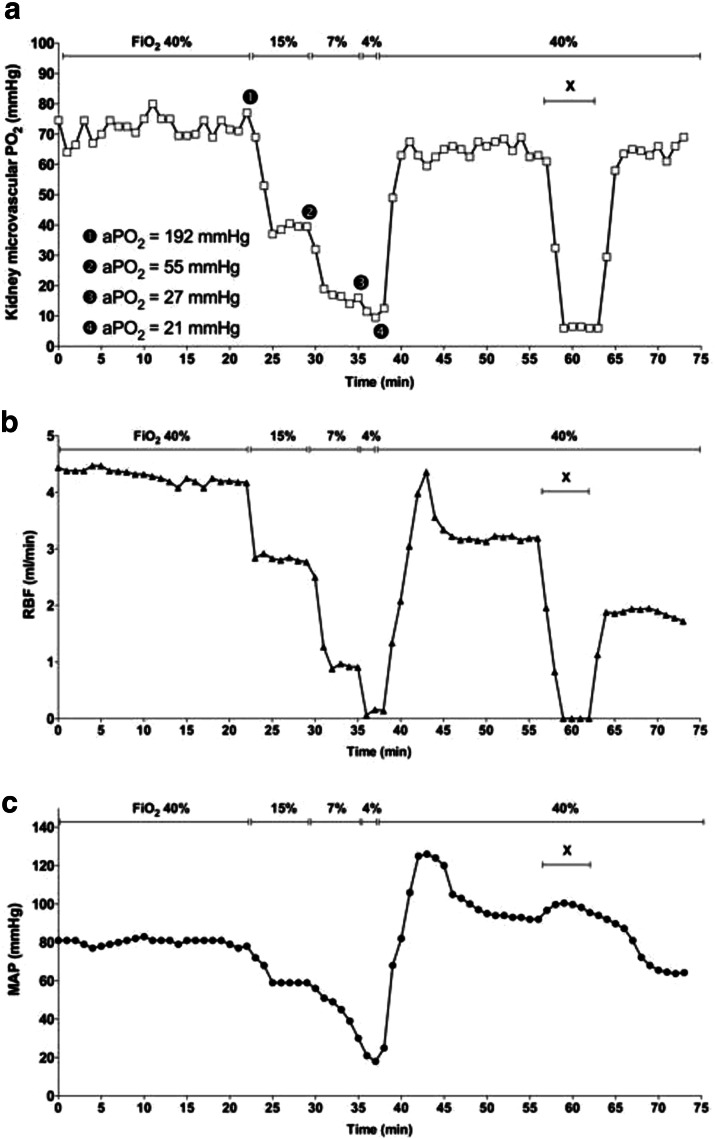
Trends in kidney μPO2 during hypoxia and ischemia

## RESEARCH IN PERIOPERATIVE CARE

### A31 Activated leukocytes in the sublingual microcirculation during coronary bypass graft surgery

#### Z. Uz^1^, M. Massey^2^, Y. Ince^3^, R. Papatella^3^, E. Bulent^3^, P. Guerci^3^, F. Toraman^4^, C. Ince,^3^

##### ^1^Academic Medical Centre Amsterdam, Translational Physiology, Amsterdam, Netherlands; ^2^Harvard Medical School, Department of Energency Medicine, Boston, United States; ^3^Academic Medical Center, University of Amsterdam, Transaltional Physiology, Amsterdam, Netherlands; ^4^Acibadem University, Department of Cardio-thoracic Surgery, Istanbul, Turkey

**Correspondence:** Z. Uz – Academic Medical Centre Amsterdam, Translational Physiology, Amsterdam, Netherlands

**Introduction:** Systemic inflammatory response is a complication that occurs frequently in cardiac surgery patients with extracorporeal circulation (EC). Leucocytosis is a common outcome after cardiac surgery due to surgical trauma, blood contact to nonendothelial surfaces in the EC, endotoxaemia and ischemia reperfusion injury due to aortic cross clamping and release. This induces an activation of complement factors, coagulation pathways and cellular immune response. Leucocyte (Lc) adhesion to the endothelial followed by diapedesis is a key component of the inflammatory response. In the context of cardiac surgery therefore Lc adhesion is expected especially following release of cross clamp. Currently there is no direct method of identifying this behavor of Lc in the microcirculation. This study describes the use of Cytocam IDF imaging (handheld video microscope) as a tool to identify and quantify the behavior of Lc in the sublingual microcirculation of patients during surgery.

**Objectives:** The introduction and application of an algorithm as a method to count non-invasively Lc in the microcirculation.

**Methods:** In this prospective observational study videos of the sublingual microcirculation during, coronary artery bypass surgery with EC, were recorded with the use of the CytoCam (Braedius Medical, Amsterdam, The Netherlands). Two time points were measured, baseline (T0) after induction of anesthesia before CPB, second time point (T1) after release cross clamp.25 patients were recruited, of which 10 were included in this study based on evaluation of the sublingual video images using a quality criteria in order to identify at least one post capillary venule (PCV) where focus of the red blood cells is primarily important. Patients in this group received blood transfusions during surgery.3 clips for each time point with duration of 4 sec (25 frames/sec) in different spots were captured. Identifying a PCV to count the activated Lc: sticking and rolling Lc on the wall of the PCV. Converting these clips into movies with a lower frame rate in order to match the velocity of the leukocytes to that of the frame speed allowed the movement of the rbc's to look unclear. Selecting 2-5 PCV good quality for each clip ( at least one PCV).

**Results:** The rolling/sticking Lc from T0 (7.3 ± 1.5 Lc/PCV/4 sec) to T1 (14.5 ± 0.7 Lc/PCV/4 sec) increased significantly (P = < 0.0001). The systemic leukocytes before (7.2 ± 0.9 x10^9/L ) and after (10.6 ± 1.5 x10^9/L ) surgery also increased significantly (p = 0.0002).

**Conclusions:** This study introduces a bedside methodology using CytoCam IDF imaging for quantifying Lc activation of sublingual microcirculation. Application of this methodology to cardiac surgery identified an increase in microcirculatory activated Lc in parallel to an increase in Lc numbers in the systemic circulation.

**References**

1. A.Kara et al.Current Opinion in Anaesthesiology: February 2016 - Volume 29 - Issue 1 - p 85-93

**Fig. 18 (abstract A31).**
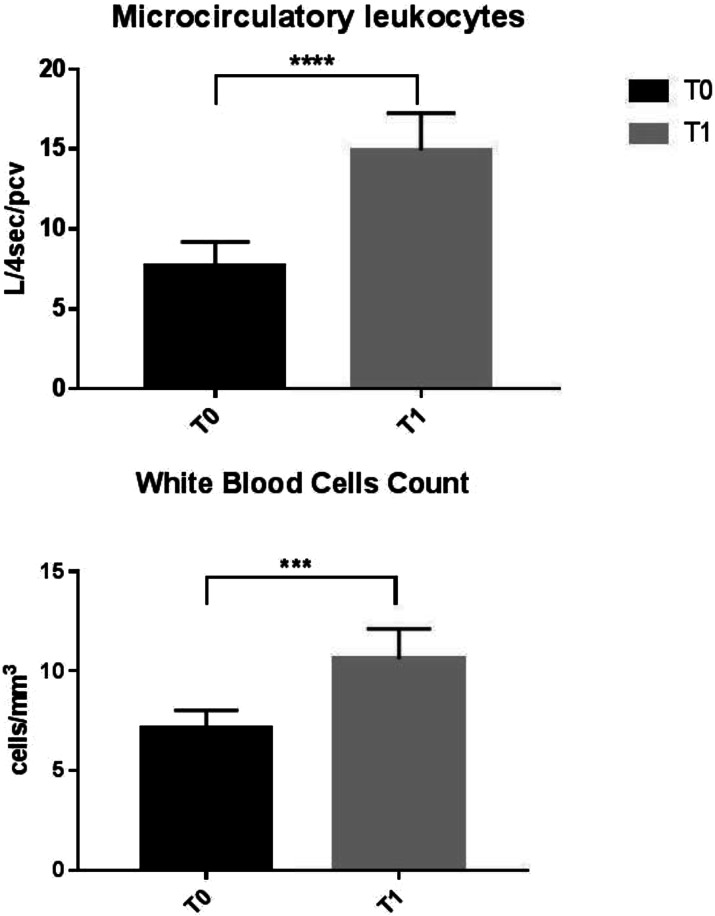
Leukocytes count in systemic and microcirculation

### A32 Granulocyte macrophage colony-stimulating factor and interferon gamma act through different mechanisms in reversing post-operative immune suppression

#### E.R. Longbottom^1,2^, H.D. Torrance^1,3^, H.C. Owen^1^, C.J. Hinds^1,2^, R.M. Pearse^1,2^, M.J. O'Dywer^1,2^

##### ^1^Queen Mary University of London, WHRI, Translational Medicine and Therapeutics, London, United Kingdom; ^2^Barts Health NHS Trust, Adult Critical Care Unit, London, United Kingdom; ^3^Barts Health NHS Trust, Trauma Sciences, Blizzard Institute, London, United Kingdom

###### **Correspondence:** E.R. Longbottom – Queen Mary University of London, WHRI, Translational Medicine and Therapeutics, London, United Kingdom

**Introduction:** We have previously demonstrated that the incidence of infection following major abdominal surgery is 35 %^1^ and is associated with the extent of post-operative immune suppression^2^. It remains unclear which clinically available immune stimulant may best suited as an adjunct to prevent post-operative infection.

**Objectives:** To determine whether Granulocyte macrophage colony-stimulating factor (GM-CSF) and interferon gamma (IFNγ) act through different pathways in reversing defects in monocyte antigen presentation following major abdominal surgery.

**Methods:** Serum was collected from 12 patients undergoing elective abdominal surgery (Research ethics approved). The median patient age was 68 years (IQR 48-74), median operation time was 270mins (IQR 207-320) and 50 % (6) developed nosocomial infection.

Pooled healthy peripheral blood mononuclear cells (PMBCs) were incubated with media containing 30 % serum taken either pre-operatively or 24 hrs post operatively and then with the addition of GM-CSF (10 ng/ml) and INFg 250 IU/ml).

Monocyte human leucocyte antigen-DR (mHLA-DR) membrane density (geometric mean fluorescent intensity) was characterised using flow cytometry following a 20-hour incubation. Cells were then sorted on a BD FACS ARIA IIIu system using CD14 positive selection. Messenger RNA (mRNA) was extracted from the sorted population (>90 % purity post sorting). Genes of interest were quantified using polymerase chain reaction (PCR) using TaqMan**®** labelled primers and real time PCR (ABI HT7900).

Flow cytometry data was analysed using FlowJo. Continuous variables were analysed using a Wilcoxin signed-rank test (JMP (version 11) statistical software).

**Fig. 19 (abstract A32).**
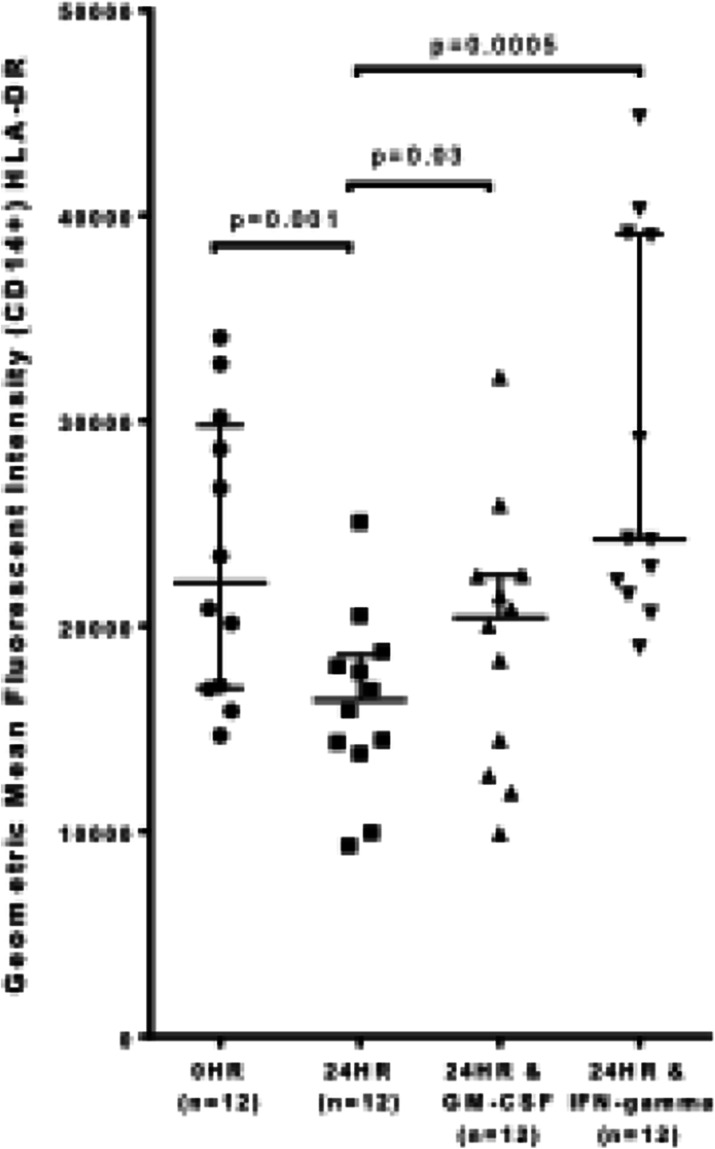
Effects of GM-CSG, INFγ on mHLA-DR geometric mean intesity in post-operative serum. Results are depicted in a dot plot with media and IQR, n=12, 3 independent experiments

**Fig. 20 (abstract A32).**
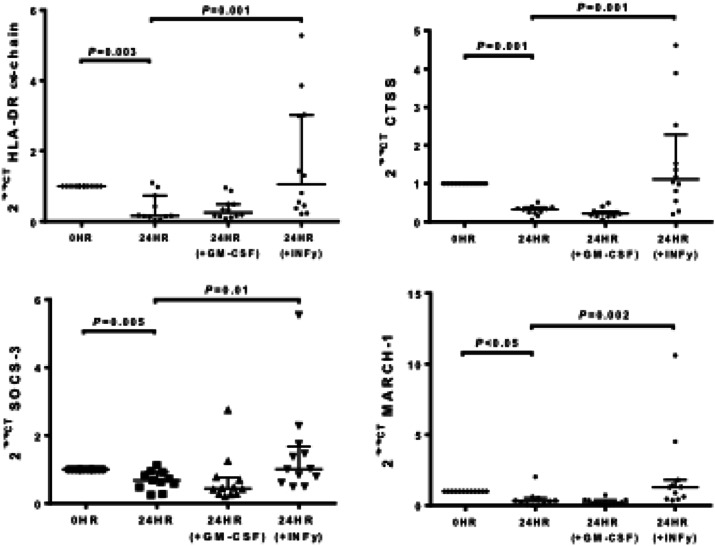
mRNA levels in monocytes incubated with post-operative serum containing either GM-CSF or INFγ. Dot plots, with median and IQR represented, n=12

**Results:** PMBCs incubated with post-operative serum demonstrated a significant reduction in mHLA-DR membrane density (Fig. 19, p = 0.001). The reduction in mHLA-DR density was prevented when co-incubated with GM-CSF and IFNγ (Fig. 19). Incubation with IFNγ but not GM-CSF increased expression of HLA-DRα chain (p = 0.01), Cathepsin S (CTSS) (p = 0.001), suppressor of cytokine signalling 3 (SOCS3) (p = 0.01) and March 1 (p = 0.002) (Fig. 20).

**Conclusions:** These results suggest that these immune stimulants, GM-CSF and IFNγ, exert their effects on monocyte antigen presentation through different signalling pathways. The increased gene expression associated with IFNγ may be indicative of potential therapeutic benefit in reversing post-operative immune suppression.

**References**

**1.** Torrance et al., Curr Opin Anaesthesiol. 2016 Mar 9.[Epub ahead of print]

**2**. Fragkou et al., EJA Volume 31, e-supplemental 52, pg 141AP4-2, June 2014.

**Grant acknowledgement**

NIAA (BJA/Royal College of Anaesthetists) and the European Society of Anaesthesiologists.

### A33 Impact of a tailored multifaceted implementation of the pain, agitation and delirium guidelines in adult critically ill patients on guideline adherence, delirium and clinical outcomes: a prospective multicenter before-after study

#### Z. Trogrlic^1^, M. van der Jagt^1^, H. Lingsma^2^, H.H. Ponssen^3^, J.F. Schoonderbeek^4^, F. Schreiner^5^, S.J. Verbrugge^6^, S. Duran^7^, T. van Achterberg^8^, J. Bakker^1^, D.A.M.P.J. Gommers^1^, E. Ista^9^

##### ^1^Erasmus Medical Center, Department of Intensive Care, Rotterdam, Netherlands; ^2^Erasmus Medical Centre, Department of Public Health, Rotterdam, Netherlands; ^3^Albert Schweitzer Hospital, Department of Intensive Care, Dordrecht, Netherlands; ^4^Ikazia Hospital, Department of Intensive Care, Rotterdam, Netherlands; ^5^IJsselland Hospital, Department of Intensive Care, Rotterdam, Netherlands; ^6^Sint Franciscus Gasthuis, Department of Intensive Care, Rotterdam, Netherlands; ^7^Maasstad Hospital, Department of Intensive Care, Rotterdam, Netherlands; ^8^KU Leuven, Academic Centre for Nursing and Midwifery, Leuven, Belgium; ^9^Erasmus Medical Center - Sophia Children's Hospital, Department of Pediatric Surgery, Intensive Care Unit, Rotterdam, Netherlands

###### **Correspondence:** Z. Trogrlic – Erasmus Medical Center, Department of Intensive Care, Rotterdam, Netherlands

**Introduction:** Delirium in critically ill patients is associated with increased mortality and long-term cognitive decline. The recent Pain, Agitation and Delirium (PAD) guidelines include recommendations for delirium screening, prevention and management, which may help to improve clinical outcomes and reduce delirium burden.

**Objectives:** We aimed to measure the effectiveness of a multifaceted implementation program for improved adherence to the PAD guidelines and associated changes in delirium incidence and duration, length of ICU stay and hospital mortality.

**Methods:** A prospective multicenter before-after study was conducted in six ICUs in the Netherlands between March 2012 and April 2015. The intervention consisted of a two-phase multifaceted tailored implementation of the PAD guidelines. Multiple implementation strategies were applied to change clinical practice (Table 18). Data of all adult ICU patients were collected during three four-month periods:

1. before implementation,
2. after implementation of delirium screening, and
3. after implementation of other PAD guidelines.

The difference in adherence and clinical outcomes between the three periods was assessed with random effects Poisson and logistic regression with a random intercept for patients for outcomes on patient-day level and with logistic and linear regression for outcomes on patient level, adjusted for APACHE II, hospital, age and admission type. Differences were expressed as adjusted rate ratios (aRR), odds ratios (aOR) or beta's with the “before”-period as the reference.

**Results:** A total of 4727 patients were enrolled in the study with a total of 23958 ICU days. Adherence to most PAD guideline recommendations improved significantly whereas early mobilization and reduced benzodiazepine sedation improved only in the last period (Table 19). The incidence of delirium increased from 22 % before to 30 % after implementation (aOR = 1.5, *p* < 0.01) whereas delirium duration decreased from 6.3 before to 3.6 days after implementation (aBeta = -2.6, *p* < 0.01). There were no statistically significant differences in ICU length of stay (aBeta = 0.001, *p* = 0.99); ICU mortality (aOR = 1.2, *p* = 0.17); and hospital mortality (aOR = 1.2, *p* = 0.21) after vs. before the implementation. Only length of mechanical ventilation increased with half a day (aBeta = 0.55, *p* = 0.01).

**Conclusions:** This multifaceted implementation program was effective in improving adherences to multiple key PAD guideline recommendations. Delirium duration decreased significantly in spite of increased incidence probably due to improved screening. We observed differential effects of screening vs. further guideline implementation. However, these improved adherences to guideline recommendations did not translate into measurable improvements of short-term clinical outcomes. To improve clinical outcomes, future investigations on PAD guideline implementation should consider focusing on specific recommendations or targeting long-term outcomes.

**Table 18 (abstract A33).** Implementation strategies used^a^

Category	Guideline implementation
Professional	1. Distribution of educational materials 2. Educational meetings 3. Local consensus processes 4.Outreach visits 5.Local opinion leader 6. Patient-mediated intervention 7. Audit and feedback 8. Reminders 9.Marketing / Tailored interventions 10.Mass media
Organizational	11. Provider oriented interventions 12. Patient oriented interventions 13. Structural interventions
Financial	14.Provider or patient interventions
Regulatory	15.Changes in medical liability 16.Peer review or Licensure

^a^ Implementation strategies used are described according to EPOC (Cochrane Effective Practice and Organization of Care) classification system. Only "Management of patients complaints" strategy was not used in this study

**Table 19 (abstract A33).** Adherence to guideline recommendations

Performance indicator	Period I Before Implementation	Period II After Screening Implementation	Period III After Guideline Implementation	OR or RR (95% CI)	*p*
no. patient-days (n= 4727 patients)	8005	8207	7746		
Delirium screening (assessments / patient-days)	0.80	2.4	2.6	4.6 (4.4, 4.9)	<0.01
Sedation assessments (assessments / patient-days)	0.67	0.76	0.77	5.8 (4.3, 7.9)	<0.01
no. total sedation days (n=2044 patients)	3368	3210	3489		
Light sedation (days / total sedation days)	0.61	0.76	0.74	3.1 (2.5, 3.9)	<0.01
Use of benzodiazepines (days / total sedation days)	0.33	0.31	0.19	0.1 (0.1, 0.2)	<0.01
no. patient-days in patients (n=1766) admitted>48 hours	5978	5868	5834		
Performing Physical Therapy (physical therapy days / patient-days)	0.2	0.44	0.45	5.3 (4.3, 6.6)	<0.01
Performing mobilization (mobilization days / patient-days)	0.1	0.13	0.18	2.3 (1.8, 3.1)	<0.01

### A34 Mitochondrial pathogenesis of propofol infusion syndrome in an vitro model of human skeletal muscle

#### A. Krajčová^1,2^, P. Waldauf^3^, F. Duška^1,3^

##### ^1^Third Faculty of Medicine, Charles University in Prague, Laboratory for Metabolism and Bioenergetics, Prague, Czech Republic; ^2^Third Faculty of Medicine, Charles University in Prague, Centre for Diabetes, Metabolism and Nutrition, Prague, Czech Republic; ^3^Third Faculty of Medicine, Charles University in Prague, Department of Anaesthesiology and Intensive Care, Prague, Czech Republic

###### **Correspondence:** A. Krajčová – Third Faculty of Medicine, Charles University in Prague, Laboratory for Metabolism and Bioenergetics, Prague, Czech Republic

**Introduction:** Propofol infusion syndrome is a rare, but serious adverse effect of a commonly used drug with a very high mortality rate (˃50 %) [1]. The symptoms can occur in various combinations and include: unexplained metabolic acidosis, arrhythmia, Brugada like pattern on electrocardiograph (elevated ST-segment and coved-T wave), cardiac and/or renal failure, rhabdomyolysis, hyperkalaemia, hepatomegaly and hyperlipidaemia. The mechanism of the syndrome is still unknown: experimental studies performed on animal models and clinical features of the syndrome are suggestive of its mitochondrial origin.

**Objectives:** We hypothesize that propofol decreases respiratory chain capacity, inhibits fatty acid oxidation and induces inner mitochondrial membrane uncoupling in a dose-dependent manner. Our study aims to test this hypothesis in vitro by exposing human skeletal muscle-derived cells to a range of propofol concentrations for 4 days.

**Methods:** Skeletal muscle cells were isolated from biopsies obtained from patients (n = 16) undergoing hip replacement surgery and subsequently exposed to a range of propofol resembling clinical concentrations in human plasma during propofol infusion (0, 1, 2.5, 5 a 10 μg/ml) and to lipid vehicle (Intralipid® - IL). After 96 hours of exposure, mitochondrial metabolism was assessed by extracellular flux analysis (Seahorse Biosciences). Oxygen consumption rate (OCR) was measured at baseline and after addition of ATPase inhibitor, mitochondrial uncoupler and complex III inhibitor. Injection of these agents enables to calculate baseline OCR, ATP turnover rate, proton leak through inner mitochondrial membrane and respiratory chain capacity (uncoupled respiration). The capacity of fatty acid oxidation was measured as etomoxir-inhibitable OCR after adding of uncoupler and palmitate. Values presented in Table 20 are expressed as % of baseline OCR.

**Results:** In human skeletal muscle cells exposed to propofol, respiratory chain capacity was decreased and uncoupling of inner mitochondrial membrane was increased. The most significant result was propofol-induced inhibition of fatty acid oxidation to 15 %, respectively 11 % of baseline values (see Table 20). Data are presented as median (interquartile range). Statistically significant results are signed as ^*^ if p-value < 0.05, ^**^ p-value < 0.001.

**Conclusions:** Propofol, in clinically relevant concentrations, is a potent inhibitor of fatty acid oxidation and induces changes in a function of respiratory chain in an in vitro model of human skeletal muscle.

**References**

[1] Krajčová A, Waldauf P, Anděl M, Duška F. Propofol infusion syndrome: a structured review of experimental studies and 153 published case reports. *Crit Care*. 2015 Nov 12;19:398.

**Grant acknowledgement**

The work was supported by grants GAUK 270915 and PRVOUK P31.

**Table 20:** (abstract A34).

Propofol concentration [μg/ml]	0	1.0	2.5	5.0	10.0	Intralipid control
Basal OCR [pmol/min]	114 (91.5-181)	89 (52-159)	112 (87-155)	104 (58-179)	123 (63-207)	100 (73-124)
Proton leak (% OCR)	23 (16-36)	25* (16-53)	26* (17-44)	35* (16-45)	20 (12-28)	26 (13-40)
ATP turnover (% OCR)	77 (64-84)	76* (47.1-84)	74* (56-83)	65* (56-84)	80 (72-88)	74 (60-87)
Maximal respiratory capacity (% OCR)	311 (251-383)	190** (44.3-375)	175** (78.8-290)	260** (150-305)	244 (196-358)	370 (293-433)
Fatty acid oxidation [pmol/min]	75 (40-134)	-	11** (2-34)	-	8** (1-27)	-

### A35 Iron supplementation to treat anaemia in adult critical care patients: a systematic review and meta-analysis

#### A. Shah^1^, N. Roy^2^, S. McKechnie^1^, C. Doree^3^, S. Fisher^3^, S.J. Stanworth^4^

##### ^1^Oxford University Hospitals NHS Foundation Trust, Nuffield Division of Anaesthetics, Oxford, United Kingdom; ^2^University of Oxford, Weatherall Institute of Molecular Medicine, Oxford, United Kingdom; ^3^Oxford University Hospitals NHS Foundation Trust, Systematic Review Initiative NHS Blood & Transplant, Oxford, United Kingdom; ^4^Oxford University Hospitals NHS Foundation Trust, Department of Haematology, Oxford, United Kingdom

###### **Correspondence:** A. Shah – Oxford University Hospitals NHS Foundation Trust, Nuffield Division of Anaesthetics, Oxford, United Kingdom

**Introduction:** Anaemia affects 60-80 % of patients admitted to critical care (ICU).^1^ Allogeneic red blood cell (RBC) transfusions remain the mainstay of treatment for anaemia but are associated with risks^1^ and are costly. Some patients may have true iron deficiency and could benefit from iron replacement, reducing the need for transfusions. However, diagnosing iron deficiency in ICU patients is difficult due to the unreliability of serum ferritin in the context of co-existing inflammation.

**Objectives:** To assess the efficacy and safety of iron supplementation, by any route, in anaemic patients in adult ICUs.

**Methods:** We searched multiple databases (CENTRAL, MEDLINE, EMBASE) for RCTs comparing iron by any route with placebo/no iron. Two investigators independently assessed eligibility and extracted data. The primary outcomes were RBC transfusion requirement, mean number of transfused RBCs and mean haemoglobin (Hb) concentration. Secondary outcomes included mortality, infection, length of hospital stay, health-related quality of life (QoL), mean difference in iron biomarkers (e.g. ferritin) and adverse events. Outcomes were assessed at two time points: (i) short-term - up to 10 days and (ii) mid-term - last measured time point in hospital or end of the trial. Risk of study bias was assessed using Cochrane methodology. Meta-analyses were performed in RevMan v5.3 using random effects models. Continuous variables were reported as mean difference (MD) with 95 % confidence interval (CI); dichotomous variables were reported as relative risk (RR) with 95 % CI.

**Results:** Five trials consisting of 613 patients were included for meta-analysis.^2,3,4,5,6^ Four trials were set in surgical ICUs and one in a mixed ICU. Only one trial was rated at low risk of bias in all domains. There was variation in dosage regimes, from 3x/weekly administration of intravenous iron to daily oral iron for up to 30 days post discharge. Results are shown in Table 21. No trials reported on QoL.

**Conclusions:** Iron supplementation does not reduce RBC transfusion requirements in adult ICU patients. Larger, well-designed trials are needed to investigate the benefits and risks of iron, optimal dosing regimes and strategies to identify patients likely to benefit together with patient-focused outcomes such as QoL after discharge.

**References**

1. Marik PE, Corwin HL. Crit Care Med 2008; 36: 2667-2674.

2. Pieracci FM, Henderson P, Rodney JR et al. Surgical Infection (Larchmt) 2009; 10: 9-19

3. Pieracci FM, Stovall RT, Jaouen B et al. Crit Care Med 2014; 42: 2048-57.

4. Garrido-Martin P, Nassar-Mansur MI, Llana-Ducros R et al. Interact Cardiovasc Thorac Surg. 2012; 15: 1013-1018

5. van Iperen CE, Gaillard CAJM, Kraaijenhagen RJ et al. Crit Care Med 2002; 28: 2773-2778.

6. Madi-Jebara S, Sleilaty GS, Achouch PE et al. J Cardiothorac Vasc Anesth 2004; 18: 59-63.

**Grant acknowledgement**

This research was supported by NHS Blood and Transplant.

**Table 21 (abstract A35).** Outcomes suitable for meta-analysis

Outcome	Trials	Participants	Effect estimate
Requirement for allogeneic RBC transfusion	5	613	RR 0.88 (95% CI 0.74 - 1.06, p=0.18)
Mean number of RBC units transfused	2	183	MD -2.06 units (95% CI -8.24 - 4.12, p=0.51)
Mean Hb (g/dL) at short-term follow-up	3	242	MD -0.25 g/dL; 95% CI -0.79 - 0.28, p=0.35
Mean Hb (g/dL) at mid-term follow-up	3	238	MD 0.21 g/dL; 95% CI -0.13-0.55, p=0.23
Mortality	4	454	RR 1.04 (95% CI 0.43 - 2.52, p=0.92)
Infection	2	347	RR 0.89 (95% CI 0.74 - 1.08, p=0.23)
Mean ferritin (ng/ml) at short-term follow-up	3	241	MD 193.61 ng/ml (95% CI -122.39 - 509.61, p=0.23)
Mean ferritin (ng/ml) at mid-term follow-up	3	235	MD 358.79 ng/ml (95% CI 169.85 - 547.74, p=0.0002)

## THE TOP FIVE ABSTRACTS OF NURSES & ALLIED HEALTHCARE PROFESSIONALS

### A36 Toward a new orientation in post-ICU patients: a qualitative longitudinal analysis of follow-up consultations in the RAPIT-study

#### J.F. Jensen^1^, D. Overgaard^2,3^, M.H. Bestle^1^, D.F. Christensen^1^, I. Egerod^4,5^, The RAPIT Group

##### ^1^Nordsjællands Hospital, University of Copenhagen, Department of Anesthesiology, Hillerød, Denmark; ^2^Metropolitan University, Department of Nursing, Copenhagen, Denmark; ^3^Nordsjællands Hospital, Research Unit, Hillerød, Denmark; ^4^University of Copenhagen, Rigshospitalet, Department of Neuroanesthesiology, Copenhagen, Denmark; ^5^University of Copenhagen, Health & Medical Sciences, Copenhagen, Denmark

###### **Correspondence:** J.F. Jensen – Nordsjællands Hospital, University of Copenhagen, Department of Anesthesiology, Hillerød, Denmark

**Introduction:** Intensive care is lifesaving, but associated with the development of physical, mental and cognitive problems in survivors. Intensive care aftercare has emerged to help patient recovery and return to normal life. Guidelines focus on promoting recovery and improving quality of life. More insight is needed into the mechanisms of intensive care recovery.

**Objectives:** To describe the patient experience of recovery from a longitudinal perspective by analyzing follow-up consultations at three time-points.

**Methods:** The study had a descriptive multicenter longitudinal qualitative design. We selected strategically a sub-sample of 36 consultations with 12 patients from a randomized controlled trial on intensive care recovery from ten Danish intensive care units (ICUs). Data were prospectively collected through the intervention and were audio-recordings of three follow-up consultations (at 3, 5 and 10 months), patient photographs during ICU-stay, and reflection sheets. First consultation focused on patient's narratives of ICU supported by photographs to aid memory. Second and third consultations focused on patient-centred dialogs of what was most important by guided by patients reflection sheets. Thematic analysis and narrative theory were used to explore the mechanisms of recovery after intensive care (1).

**Results:** The basic narrative of recovery was “toward a trajectory of new orientation”. The narrative at 3 months described mortal illness in ICU (Being at Death's door), the narrative at 5 months described ongoing fear of relapse (Still not out of the Woods), and the narrative at 10 months had three potential outcomes: downhill (detour on the road), steady-state (end of the road), or progress (The Road to Recovery). New orientation was obtained in steady-state or progressive recovery, Fig. 21.

**Conclusions:** This study provides a contemporary understanding of the process of intensive care recovery. Recovery evolves through narratives of mortal danger, fear of relapse and different types of progress toward a new orientation in life. Nurse-led follow-up consultations help patients to obtain a sense of coherence during the first year after critical illness. These findings enable healthcare professionals to understand what patients experience during stages of recovery by offering dialogue and supporting the construction of a coherent illness narrative with ICU staff and close relatives. This shared understanding is important to improve nursing and healthcare professionals in the assessment of long-term outcome, and management of patients after intensive care.

**References**

1. Braun V CV (2006) Using Thematic analysis in psychology in Qualitative research in psychology. Universiy of West England, London, http://dx.doi.org/10.1191/1478088706qp063oa, Accessed Feb. 14th, 2015.

**Grant acknowledgement**

The Danish Nursing Organization, The Novo Nordisk Foundation and Nordsjællands Hospital, University of Copenhagen, DK

**Fig. 21 (abstract A36).**
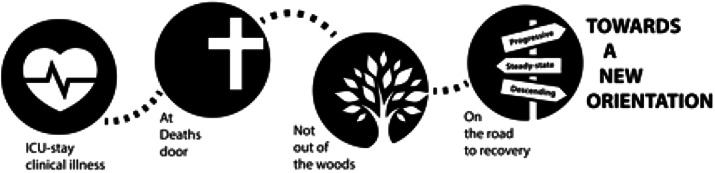
Basic illness narrative across three consultations

### A37 Effect of a skin ointment creating a polymer protective film beneath transparent catheter dressings on skin integrity and risk of dressing disruption

#### A. Pivkina^1^, V. Gusarov^1^, I. Zhivotneva^2^, N. Pasko^1^, M. Zamyatin^2^

##### ^1^N.I. Pirogov National Medical Surgical Center, Anestesiology and Intensive Care, Moscow, Russian Federation, ^2^N.I. Pirogov National Medical Surgical Center, Moscow, Russian Federation

###### **Correspondence:** A. Pivkina – N.I. Pirogov National Medical Surgical Center, Anestesiology and Intensive Care, Moscow, Russian Federation

**Introduction:** Skin breakdown caused by adhesive dressings is a risk factor for central line-associated bloodstream infection (CLABSI). Protective skin products are developed, but it is uncertain whether the use of such products is not associated with a higher rate of dressing disruption, which is also a risk factor for CLABSI [1]. On the other hand, a transparent dressing with a chlorhexidine gluconate (CHG) containing gel pad at the insertion site prevents CLABSI.

**Objectives:** To compare transparent catheter dressings either with or without the application of a protective skin ointment for skin integrity, dressings disruptions and dwell time; in addition, we assessed rates of catheter colonization and CLABSI in transparent dressing either with or without a CHG-pad.

**Methods:** We conducted a monocentric, open-label, randomized, controlled trial (Aug.-Dec./2014) to compare transparent CHG-dressings with use of a skin ointment creating a polymer protective film (Cavilon®) (intervention group) with standard transparent dressings without the skin product (control group). Standard catheter care included 0.5 % CHG in 70 % ethanol for skin preparation before CVC insertion and maximal sterile barriers. Dressings were changed /7 days, or in case of full dressing disruption (revealing the insertion site) or if moisture was present under the dressing.

**Results:** Sixty patients with a central venous catheter (CVC) were enrolled accounting for 60 CVCs and a total of 533 CVC days. Study groups did not differ in sex, age, insertion site and the number of CVC lumens, comorbidities, severity of condition, length of hospital stay before enrollment, or concomitant therapy. Dressing dwell time was higher in the intervention group: 6.3 (SD 1.5) vs 2.0 (SD 1.1) days (p < 0.001). The Table describes reasons for dressing changes and skin integrity after dressing removal.

CVC dwell time did not differ between the groups: 9.7 (SD 3.5) days in the intervention group and 8 (SD 3.9) days in the control group (p > 0.05). Rates of CVC colonization were not different (37.9/1000 CVC days in the intervention group vs. 37.0/1000 CVC days in the control arm [relative risk 1.22, 95 % confidence interval 0.59-2.5]) as were CLABSI rates (6.9/1000 vs 20.6/1000 CVC days [relative risk 0.4, 95 % confidence interval, 0.08-1.9]).

**Conclusions:** The use of a skin ointment creating a polymer protective film beneath transparent dressings results in longer dressing dwell times and less skin breakdown. The application of the skin product does not alter the risk of infection, at least not when used combined with a CHG-impregnated dressing.

**References**

1. Timsit J.F., Bouadma L., Ruckly S. et al. Dressing disruption is a major risk factor for catheter-related infections**.** Crit Care Med. 2012; 40(6): 1714-1707.

**Table 22 (abstract A37).** Reasons for dressing change and skin condition at

Feature	Control group (n=30)	Intervention group (n=30)	p
Reasons for dressing change			
Partial dressing disruption, n (%)	4 (13.3)	7 (23.3)	>0.05
Full dressing disruption (revealing insertion point), n (%)	17 (56.7)	2 (6.7)	<0.001
Moisture presence under the dressing, n (%)	6 (20)	0	0.0093
Skin condition at the CVC site after the dressing removal			
Hyperemia of the insertion site, n (%)	4 (13.3)	1 (3.3)	>0.05
Presence of skin irritations under the dressing, n (%)	1 (3.3)	0	>0.05
Residues of adhesive on the skin around the catheter, n (%)	0	0	>0.05
Presence of discharge from the insertion site, n (%)	2 (6.7)	0	>0.05

### A38 The effectiveness of a recovery program aimed at improving quality of life and sense of coherence in post intensive care patients: a pragmatic multicenter randomized controlled trial, the recovery and aftercare of post intensive care patients (RAPIT) study

#### J.F. Jensen^1^, I. Egerod^2,3^, M.H. Bestle^1^, D.F. Christensen^1^, A. Alklit^4^, R.L. Hansen^1^, H. Knudsen^5^, L.B. Grode^6^, D. Overgaard^7,8^, The RAPIT group

##### ^1^Nordsjællands Hospital, University of Copenhagen, Department of Anesthesiology, Hillerød, Denmark; ^2^University of Copenhagen, Health & Medical Sciences, Copenhagen, Denmark; ^3^University of Copenhagen, Rigshospitalet, Department of Neuroanesthesiology, Copenhagen, Denmark; ^4^University of Southern Denmark, Department of Psychology, Odense, Denmark; ^5^Herlev Hospital, University of Copenhagen, Department of Anesthesiology, Herlev, Denmark; ^6^Hospital of Horsens, Department of Anesthesiology, Horsens, Denmark; ^7^Metropolitan University, Department of Nursing, Copenhagen, Denmark; ^8^Nordsjællands Hospital, Research Unit, Hillerød, Denmark

###### **Correspondence:** J.F. Jensen – Nordsjællands Hospital, University of Copenhagen, Department of Anesthesiology, Hillerød, Denmark

**Introduction:** More ICU programs are emerging to promote psychological recovery after a stay in intensive care unit (ICU) (1). In the Scandinavian countries, patients' discharge rehabilitation plan usually includes physical training, but seldom psychological rehabilitation. To address this gap, we developed a post-ICU recovery program to improve psychological health after intensive care. We hypothesized that a nurse-led recovery program, that helped to construct a coherent illness narrative including person-centered communication, would improve health-related quality of life (HRQOL), sense of coherence (SOC), reduce symptoms of anxiety, depression, and post-traumatic stress (PTSD) in the first year after ICU discharge.

**Objectives:** To investigate the effectiveness of a post-ICU recovery program compared to standard care in improving quality of life in the first year after ICU.

**Methods:** We randomly assigned 386 patients adult (≥18 years) survivors after receiving mechanical ventilation (≥48 hours) to standard care (SC) plus a recovery program or SC alone after discharge from intensive care. It was a nurse-led intervention consisted of patient photographs, three follow-up consultations and reflection sheets.

**Results:** Primary outcome was HRQOL, and secondary outcomes were SOC, anxiety, depression, PTSD assessed at 3 and 12 months after intensive care discharge using *t*-tests. We supplemented outcomes with rehabilitation services within the first year. At 12 months after intensive care the HRQOL scores were unchanged by the intervention (mean difference in the Mental Component Summary score, 0.9 [95 % CI, −1.5 to 3.3; P =0.47]; and in the Physical Component Summary score, 1.1 [95 % CI, −1.3 to 3.5; P = 0.37]). No differences were found for the total score on self-reported SOC, anxiety, depression, and PTSD. The intervention showed a potential effectiveness on the rate of anxiety, when a cutoff score ≥11 was applied, in the complete analysis at 3 months. However, all patients received high level of rehabilitation services as SC within the first year after intensive care.

**Conclusions:** Overall, no beneficial or detrimental effects were found on either primary or secondary outcomes, but the symptoms were generally lower that found in similar studies. Patients maintained good mental health and a strong sense of coherence, but the PTSD score was consistently high. The high quality of standard care in Danish ICUs might help explain the lack of improvement in this study.

**References**

1. Jensen JF et al.,(2015) Impact of follow-up consultations for ICU survivors on post-ICU syndrome: a systematic review and meta-analysis. Intensive Care Med 41: 763-775

2. Egerod I et al., (2011) Constructing the illness narrative: a grounded theory exploring patients´ and relatives´ use of intensive care diaries. Crit Care Med 39: 1922-1928

**Grant acknowledgement**

The Danish Nursing Organization, The Novo Nordisk Foundation and Nordsjællands Hospital, University of Copenhagen, DK

### A39 The value of progressively accrued information during initial post-admission hours in forecasting future cardiorespiratory instability

#### M. Hravnak^1^, L. Chen^2^, A. Dubrawski^2^, G. Clermont^3^, M.R. Pinsky^3^

##### ^1^University of Pittsburgh, School of Nursing, Pittsburgh, United States; ^2^Carnegie Mellon University, Robotics Institute; Auton Lab, Pittsburgh, United States; ^3^University of Pittsburgh, Critical Care Medicine, Pittsburgh, Pennsylvania, United States

###### **Correspondence:** M. Hravnak – University of Pittsburgh, School of Nursing, Pittsburgh, United States

**Introduction:** Enabling clinicians to prospectively identify patients who will later become unstable would enable targeting resources to patients most in need as well as potential application of preventive care.

**Objectives:** To determine the incremental contribution of information progressively available within the first 4 hours of (SDU) admission to improve models forecasting later development of cardiorespiratory instability (CRI), including a novel CRI relative risk score.

**Methods:** Continuous noninvasive vital sign (VS) monitoring data (heart rate [HR], respiratory rate [RR; bioimpedance], oscillometric blood pressure [BP], peripheral oximetry [SpO_2_]) were collected from 1971 stepdown unit (SDU) patients, and CRI episodes defined as VS deviation beyond stability thresholds. Patients with any CRI (cases, n = 918) and those never displaying CRI (controls, n = 1053) were identified. We computed a minute-by-minute integrated CRI risk score based on the method described in [1], using features computed from VS data streams during trailing 15 minute rolling windows and a trained random forest machine learning model. We then computed for each patient a mean risk score aggregated from the risk scores during first 4 hours of SDU stay. Next we built a logistic regression model to forecast whether or not there will be a CRI event in the future. To mimic the temporal availability of data following patient admission, we first entered demographics available at patient admission (age, gender, Charlson Comorbidity Index score) into the model, and then the initial VS (5-minute average of continuous VS data accrued from minutes 10 to 15 after admission), and finally the relative risk score derived in the first 4 hours. We assessed the predictive contribution of information from these 3 progressively accrued categories (demographics, initial VS, 4-hr risk score) by the Area Under Receiver Operating Curve (AUC) in a 10-fold cross validation experiment setup.

**Results:** The risk score derived from admission demographics alone yielded an AUC of 58 ± 0.002 % to forecast future CRI. Adding the initial VS improved the AUC to 64 ± 0.003 %, and with further adding the 4-hr risk score the AUC became 67 ± 0.002 %.

**Conclusions:** A predictive model which incorporates patient data as it becomes available, including a risk score derived within the first 4 hours, progressively improves the models ability to forecast future CRI development. Such forecasting information could enable clinicians to identify those patients who will become unstable in future very soon after admission in order to triage patients needing closer surveillance and potentially apply preemptive interventions.

**References**

[1] Chen L et. Al. Modelling Risk of Cardio-Respiratory Instability as a Heterogeneous Process. AMIA Annu Symp Proc. 2015 Nov 5;2015:1841-50.

**Grant acknowledgement**

NIH NINR R01NR013912

### A40 What are the factors that impact on physical activity and rehabilitation for survivors of critical illness: a systematic review of quantitative and qualitative studies

#### S.M. Parry^1^, L.D. Knight^2^, B.C. Connolly^3,4^, C.E. Baldwin^5,6^, Z.A. Puthucheary^7,8,9^, L. Denehy^1^, N. Hart^3,4,10^, P.E. Morris^11^, J. Mortimore^4^, C.L. Granger^1^

##### ^1^The University of Melbourne, Department of Physiotherapy, Melbourne, Australia; ^2^Royal Melbourne Hospital, Department of Physiotherapy, Melbourne, Australia; ^3^Guy's and St Thomas' NHS Foundation Trust and Kings College, London, United Kingdom; ^4^Guy's & St Thomas' NHS Foundation Trust, Lane Fox Respiratory Unit, London, United Kingdom; ^5^University of South Australia, Member of the International Centre for Allied Health Evidence (iCAHE) and the Sansom Institute, Adelaide, Australia; ^6^Flinders Medical Centre, Department of Physiotherapy, Adelaide, Australia; ^7^National University Hospital, Division of Respiratory and Critical Care, Singapore, Singapore; ^8^University College London Hospitals, Division of Critical Care, London, United Kingdom; ^9^University College London Hospitals, Institute of Sports and Exercise Health, London, United Kingdom; ^10^Kings College London, Division of Asthma, Allergy and Lung Biology, London, United Kingdom; ^11^University of Kentucky, Division of Critical Care and Pulmonology, Kentucky, United States

###### **Correspondence:** S.M. Parry – The University of Melbourne, Department of Physiotherapy, Melbourne, Australia

**Introduction:** Physical activity / rehabilitation forms a pivotal aspect of recovery after critical illness and studies have demonstrated it is safe, feasible and potentially efficacious at improving patient outcomes [1,2]. However, international data demonstrate low levels of mobilisation occur in the ICU[3,4]. A current gap exists between the perceived need and actual practice of implementing physical activity across the recovery continuum.

**Objectives:** To identify, evaluate and synthesise studies examining the barriers and enablers for patients with critical illness to participate in physical activity from the perspective of healthcare providers, patients and caregivers.

**Methods:** Systematic review of articles using electronic databases: MEDLINE, CINAHL, EMBASE, Scopus and Cochrane. Quantitative and qualitative studies which assessed the barriers, or enablers to physical activity for patients with critical illness were included. Registered on PROSPERO (number: CRD42016035454).

**Results:** 79 studies were included. Studies included primarily ICU survivors (69 %, n = 54 studies), healthcare providers (29 %, n = 23 studies) with only one study specifically examining caregivers and patients. Barriers and enablers to physical activity were identified (5 major themes and 28 sub-themes). Patient-level barriers included physical capability (physiological stability, illness severity, sedation, weakness, delirium), psychological influences (fear/motivation) and perceived relevance. Healthcare provider barriers included lack of time/knowledge and expertise, communication, and concern for line safety. Environmental barriers included lack of resources (staffing and equipment), lower prioritisation, and lack of an established rehabilitation pathway post ICU. Enablers included: presence of mobility teams/protocols, designated discipline and overall leaders, teamwork and development of daily care plans.

**Conclusions:** This systematic review has identified the volume of literature demonstrating that barriers and enablers to physical activity are multi-dimensional and span diverse factors. These factors need to be considered when developing rehabilitation interventions to facilitate cultural change in rehabilitation practices across the recovery continuum.

**References**

1. Kayambu, G., R. Boots, and J. Paratz, *Physical Therapy for the Critically Ill in the ICU: A Systematic Review and Meta-Analysis.* Critical Care Medicine, 2013. **41**(6): p. 1543-54.

2. Morris, P.E., et al. *Early intensive care unit mobility therapy in the treatment of acute respiratory failure (Structured abstract)*. Critical Care Medicine, 2008. **36**, 2238-2243.

3. Berney, S., et al., *Intensive care unit mobility practices in Australia and New Zealand: a point prevalence study.* Critical Care and Resuscitation, 2013. **15**(4): p. 260-265.

4. Nydahl, P., et al., *Early mobilisation of mechanically ventilated patients: a one day point prevalence study in Germany.* Critical Care Medicine, 2014. **42**(5): p. 1178-1186.

## END-OF-LIFE-CARE: GET IT RIGHT THE FIRST TIME

### A41 Perceptions of end-of-life decision-making climate among healthcare providers working in european and us icus: differences between nurses and physicians

#### H.I. Jensen^1,2^, R. Piers^3^, B. Van den Bulcke^4^, J. Malmgren^5^, V. Metaxa^6^, A.K. Reyners^7^, M. Darmon^8^, K. Rusinova^9^, D. Talmor^10^, A.-P. Meert^11^, L. Cancelliere^12^, L. Zubek^13^, P. Maia^14^, A. Michalsen^15^, J. Decruyenaere^4^, E. Kompanje^16^, S. Vanheule^17^, E. Azoulay^18^, S. Vansteelandt^19^, D. Benoit^4^

##### ^1^Lillebaelt Hospital, Department of Anaesthesiology and Intensive Care, Vejle and Kolding, Denmark; ^2^University of Southern Denmark, Institute of Regional Health Research, Odense, Denmark; ^3^Ghent University Hospital, Department of Geriatrics, Gent, Belgium; ^4^Ghent University Hospital, Department of Intensive Care, Gent, Belgium; ^5^Sahlgrenska University Hospital, Department of Anaesthesiology and Intensive Care, Gothenburg, Sweden; ^6^King's College Hospital, Critical Care and Major Trauma, London, United Kingdom; ^7^University of Groningen, University Medical Center Groningen, Department of Internal Medicine, Groningen, Netherlands; ^8^Saint-Etienne University Hospital and Jacques Lisfranc Medical School, Saint-Etienne, France; ^9^General University Hospital, 1st Faculty of Medicine, Charles University in Prague, Department of Anaesthesia and Intensive Care Medicine, Prague, Czech Republic; ^10^Beth Israel Deaconess Medical Center and Harvard Medical School, Department of Anesthesia, Critical Care, and Pain Medicine, Boston, United States; ^11^Institut Jules Bordet, ULB, Service des soins Intensifs et Ergences Oncologiques, Bruxelles, Belgium; ^12^Ospedale Maggiore della Carità Novara, Dipartimento Emergenza Urgenza Anestesia e Rianimazione, Novara, Italy; ^13^Semmelweis University, Budapest, Hungary; ^14^Hospital S.António, Intensive Care Department, Porto, Portugal; ^15^Tettnang Hospital, Department of Anesthesiology and Critical Care, Tettnang, Germany; ^16^Erasmus Hospital, Department of Intensive Care, Rotterdam, Netherlands; ^17^Ghent University, Department of Psychoanalysis and Clinical Consulting, Gent, Belgium; ^18^Hôpital Saint Louis, Service de Reanimation Médicale, Paris, France; ^19^Ghent University, Department of Applied Mathematics and Computer Science and Statistics, Gent, Belgium

**Introduction:** Literature depicts differences in perceptions of End-Of-Life (EOL) decision-making (DM) between nurses and physicians.

**Objectives:** To examine perceptions of nurses and physicians in regard to EOL DM in the ICU and to test the hypothesis that the worse the EOL DM climate, the greater the discordance between nurses' and physicians' rating of EOL DM.

**Methods:** Perceptions of EOL-DM among health care providers of 68 adult ICUs in 13 European countries and the US were measured in April-May 2014, using a validated self-assessment questionnaire. The questionnaire existed of 35 questions and was based on the Appropricus questionnaire [1], the IPEQS instrument and the LDBQ questionnaire [2].

**Results:** A total of 2,275 nurses and 717 physicians participated. Response rates were 63.1 % and 62.9 %, respectively. Using factor analyses and cluster analysis, seven meaningful factors (physician leadership, interdisciplinary reflection, not avoiding EOL decisions, mutual respect within the interdisciplinary team, involvement of nurses in EOL, active DM by physicians, and ethical awareness) yielded 4 climates: good, average with and average without involvement of nurses at EOL, and fourth a poor EOL-DM climate.

When looking at overall perceptions, there were for all seven factors significant differences (p < 0.001) between nurses and physicians, with physicians consistently perceiving the EOL-DM climate as more positive as nurses. The largest differences were found in regard to physician leadership (median factor scores -0.09 for nurses and 0.61 for physicians, respectively), interdisciplinary reflection (medians -0.03 and 0.51) and not avoiding EOL decisions (medians -0.07 and 0.37). When looking at differences within the 4 types of EOL-DM, the same general pattern was found, and all differences were statistically significant (p < 0.001). The differences were largest in the two lowest types of EOL-DM: average without nurses and poor EOL-DM climate.

Head nurses generally had a more positive perception of EOL-DM climate compared to other nurses, and senior physicians had a more positive perception than junior physicians. When comparing nurses (without including head nurses) and junior physicians, the physicians still had a significantly higher perception of EOL-DM, except for “Mutual respect within the interdisciplinary team” (p = 0.26).

Physicians having a more positive perception of EOL-DM compared to nurses were found within all 13 participating countries and within the individual ICU's.

**Conclusions:** The poorer the EOL DM climate, the more physicians were likely to overestimate the EOL DM climate compared to nurses.

**References**

[1] Piers, R et al. Inappropriate Care in European ICUs: Confronting Views From Nurses and Junior and Senior Physicians. Chest 2014;146(2):267-275.

[2] Van den Bulcke B et al. The perceived quality of inter-professional ICU teamwork: A single centre intervention study. J Interprofessional Care. In press.

### A42 Perceptions of end-of-life decision making climate among European and US ICU health care providers: development and validation of a self-assessment tool to differentiate end-of-life decision making climates

#### B. Van den Bulcke^1^, R. Piers^2^, H.I. Jensen^3,4^, J. Malmgren^5^, V. Metaxa^6^, A.K. Reyners^7^, M. Darmon^8^, K. Rusinova^9^, D. Talmor^10^, A.-P. Meert^11^, L. Cancelliere^12^, L. Zubek^13^, P. Maia^14^, A. Michalsen^15^, J. Decruyenaere^1^, E. Kompanje^16^, S. Vanheule^17^, E. Azoulay^18^, S. Vansteelandt^19^, D. Benoit^1^

##### ^1^Ghent University Hospital, Department of Intensive Care, Gent, Belgium; ^2^Ghent University Hospital, Department of Geriatrics, Gent, Belgium; ^3^Lillebaelt Hospital, Department of Anaestesiology and Intensive Care, Veijle and Kolding, Denmark; ^4^University of Southern Denmark, Institute of Regional Health Research, Odense, Denmark; ^5^Sahlgrenska University Hospital, Department of Anaesthesiology and Intensive Care, Gothenburg, Sweden; ^6^King's College Hospital, Critical Care and Major Trauma, London, United Kingdom; ^7^University of Groningen, University Medical Center Groningen, Department of Internal Medicine, Groningen, Netherlands; ^8^Saint-Etienne University Hospital and Jacques Lisfranc Medical School, Saint-Etienne, France; ^9^General University Hospital, 1st Faculty of Medicine, Charles University in Prague, Department of Anaesthesia and Intensive Care, Prague, Czech Republic; ^10^Beth Israel Deaconess Medical Center and Harvard Medical School, Department of Anesthesia, Critical Care, and Pain Medicine, Boston, United States; ^11^Institut Jules Bordet, ULB, Service des Soins Intensifs et Urgences Oncologiques, Bruxelles, Belgium; ^12^Ospedale maggiore della carità Novara, Dipartimento Emergenza Urgenza Anestesia e Rianimazione, Novara, Italy; ^13^Semmelweis University, Budapest, Hungary; ^14^Hospital S. António, Department of Intensive Care, Porto, Portugal; ^15^Tettnang Hospital, Department of Anesthesiology and Critical Care, Tettnang, Germany; ^16^Erasmus Hospital, Department of Intensive Care, Rotterdam, Netherlands; ^17^Ghent University, Department of Psychoanalysis and Clinical Consulting, Gent, Belgium; ^18^Hopital Saint Louis, Service de Reanimation Médicale, Paris, France; ^19^Ghent University, Department of Applied Mathematics and Computer Science and Statistics, Gent, Belgium

###### **Correspondence:** B. Van den Bulcke – Ghent University Hospital, Department of Intensive Care, Gent, Belgium

**Introduction:** Literature depicts large differences in end-of-life (EOL) decision making (DM) between countries and ICUs.

**Objectives:** To better conceptualize EOL-DM and to develop and validate a tool to assess EOL-DM climates.

**Methods:** Perceptions of EOL-DM among health care providers (HCPs) of 68 adult ICUs in 13 European countries and the US were measured, using a self-assessment questionnaire in April-May 2014. The EOL-DM climate questionnaire, existing of 35 items, based on the Appropricus questionnaire (11 items concerning EOL care practices) and extended with 24 validated items concerning key conditions to provide good EOL care: interdisciplinary collaboration and communication (Interprofessional Practice and Education Quality Scales (IPEQS, 11 items) and leadership skills (LBDQ,13 items) (1, 2,3). Exploratory and confirmatory factor analysis followed by cluster analysis was used to determine EOL-DM climates.

**Results:** Of the 3610 nurses and 1137 doctors providing ICU bedside care, 63,1 % and 62,9 % participated respectively. Seven meaningful factors were identified (physician leadership, interdisciplinary reflection, not avoiding EOL decisions, mutual respect, involvement of nurses in EOL, active DM by physicians, ethical awareness); which yielded 4 EOL-DM climates: good (17.6 % of the ICUs), average with (+) (17.6 %) and without involvement of nurses at EOL (-) (32.3 %), and poor (54.3 %) (factor analysis p < 0.001). According to HCPs working in a good climate leadership is active and facilitates interdisciplinary reflection and decision-making overall. Within the 'average + ' climate, HCPs perceive their leaders as empowering nurses to share interdisciplinary decision-making at EOL mainly. Stimulating more open ethical awareness overall and being less hesitant in taking timely EOL decisions could be a main goal for the leader in this climate. HCPs working in an 'average -' climate do perceive their leaders as not hesitant to take important decisions (at EOL) however as insufficiently empowering the involvement of nurses in the DM process. HCPs working in a poor climate perceive a need for improvement on all previous factors. This climate was further characterized by poor communication, distrust and low respect.

**Conclusions:** We identified seven key dimensions, from which 4 meaningful types of ICU EOL-DM climates could be discerned. The climates differ mainly in 2 key dimensions: the way of nurse involvement in EOL and in physicians active DM. Our research offers opportunities to develop tailored ICU team interventions.

**References**

[1] Piers, R et al. (2014) Inappropriate Care in European ICUs: Confronting Views From Nurses and Junior and Senior Physicians,Chest,146,(2),267-275.

[2] Van den Bulcke B et al. The perceived quality of inter-professional ICU teamwork: A single centre intervention study, J Interprofessional Care. In press.

[3] Stogdill, RM &Coons, AE (Eds). (1957). Leader behavior description questionnaire. Oxford, England: Ohio State University.

### A43 Nurses' perceptions of aids and obstacles to the provision of optimal end of life care in ICU

#### C. Ryan, D. Dawson, J. Ball, K. Noone, B. Aisling, S. Prudden

##### St Georges NHS Foundation Trust, General Intensive Care, London, United Kingdom

###### **Correspondence:** C. Ryan – St Georges NHS Foundation Trust, General Intensive Care, London, United Kingdom

**Introduction:** There is increasing recognition of the need for comprehensive expertise in the management of EOLC in the ICU. However there appear to be many controversies between professionals regarding optimal provision.

**Objectives:** As a component of a local, on-going quality improvement process in this area, we performed a survey of nurses' perceptions of aids and obstacles to the optimal provision of EoLC.

**Methods:** We modified a previously validated survey tool^1^ and anglicised the language. Nurses were asked to rate both the size and frequency of 20 possible obstacles and 14 possible aids to providing EoLC using a 6 point Likert scale (0-5). The survey was distributed to 120 nursing staff on one adult general critical care unit in March 2015. Confidentiality was assured. For each obstacle and aid, the median and interquartile ranges were determined for the size and frequency. To determine the effect size of each obstacle and aid, the median of the size was multiplied by the median of the frequency. These were then themed

**Results:** Sixty surveys were returned representing a 50 % return rate.

**Conclusions:** This study has highlighted the need to proactively identify a family liaison to cascade information to friends and relatives to allow nurses to concentrate on care delivery. Despite having a poor unit design and lack of privacy, nurses feel they can provide a dignified death and feel that multidisciplinary agreement is an important part of this process.

**References**

1. Kirchoff and Beckstrand, 2000 Critical care nurses´ perceptions of obstacles and helpful behaviour's in providing end-of-life care to dying patients. Am J Crit Care. 2000 Mar;9(2):96-105.

2. Festic E,Wilson ME, Gajic O, Divertie GD, Rabatin JT, 2012 Perspectives of physicians and nurses regarding end-of-life care in the intensive care unit. J Intensive Care Med. 2012 Feb;27(1):45-54. doi: 10.1177/0885066610393465. Epub 2011 Jan 21

**Table 23 (abstract A43).** Effect size and themes obstacles

LARGE OBSTACLES occurring frequently	EFFECT SIZE	THEME
Family requesting constant updates	16	Workload
Poor unit design restricting privacy	16	Environment
Family unable to accept patient dying	12	Family Coping
Nurse caring for family and patient simultaneously	12	Workload
Not knowing patient's wishes for EOLC	12	Staff Coping
SMALL OBSTACLES occurring infrequently	EFFECT SIZE	THEME
Lack of chaplaincy services	2	Logistics
Lack of support from colleagues	3.75	Staff coping
Restrictive visiting hours	4	Logisrics

**Table 24 (abstract A43).** Effect size and themes of aids

LARGE AIDS occurring frequently	EFFECT SIZE	THEME
Multidisciplinary agreement	15	Communication
Provision of a peaceful and dignified scene	15	Environment
Allowing adequate time after death	15	Logistics
LARGE AIDS that occur infrequently	EFFECT SIZE	THEME
Having a designated family liaison	10	Workload
Having unit design that allows privacy	10	Environment
SMALL AIDS	EFFECT SIZE	THEME
Family helping with care	6	Case Specific
Having support outside work	3	Staff coping

### A44 The impact of personality and religiousness of icu personnel on end-of-life decisions

#### A. Ntantana^1^, D. Matamis^1^, S. Savvidou^1^, M. Giannakou^2^, M. Gouva^3^, G. Nakos^4^, V. Koulouras^4^

##### ^1^Papageorgiou General Hospital, ICU, Thessaloniki, Greece; ^2^AHEPA University Hospital, ICU, Thessaloniki, Greece; ^3^Technological Educational Institutes of Ipeirus, Ioannina, Greece; ^4^University Hospital of Ioannina, ICU, Ioannina, Greece

###### **Correspondence:** A. Ntantana – Papageorgiou General Hospital, ICU, Thessaloniki, Greece

**Introduction:** It has been recognized that healthcare workers involved in “End of life” (EoL) decisions may be influenced by cultural, geographical, religious and personal characteristics^1^.

**Objectives:** To investigate possible associations of ICU personnel's aspects of personality and religiousness with attitude towards EoL.

**Methods:** A cross-sectional, multicenter study was conducted in a national level in Greece during June to December 2015. ICU physicians (n = 149) and ICU nurses (n = 320) participated by answering three questionnaires: the main survey questionnaire investigating EoL attitudes^2^, the Eysenck Personality Questionnaire (EPQ)^3^, and the Spiritual and Religious Attitudes Questionnaire (SpREUK)^4^. Different scores of EPQ-Neuroticism, EPQ-Psychoticism and SpREUK-Trust in a higher guidance were recorded in each participant.

**Results:** A high participation rate was recorded (65.7 %). Eighty-four participants preferred to characterize EoL decisions as “passive euthanasia” over “refusal of futile care” (7.6 % of ICU physicians vs.23.3 % of ICU nurses, p < 0.001), 289 participants declared that removing artificial ventilation represented a different approach from other EoL decisions because of the analogy breath = life (51.4 % of physicians vs.75.8 % of nurses, p < 0.001), and 71 participants acknowledged fear of litigation as the major reason for not informing the family about EoL decisions (17.4 % of doctors vs.14.9 % of nurses, p = 0.004). Statistical analysis with multivariate logistic regression identified that attitude towards passive euthanasia could independently be predicted by high neuroticism scores (Odds ratio 1.6, 95%CI 1.1-2.7, p = 0.048), attitude towards withdrawal of artificial ventilation by trust in a higher guidance (Odds ratio 1.7, 95%CI 1.1-2.5, p = 0.010), and fear of litigation by high psychoticism scores (Odds ratio 2.4, 95%CI 1.3-4.8, p = 0.009).

**Conclusions:** The results of this study indicate that specific attitudes towards EoL decisions may be influenced by aspects of personality and religiousness of ICU personnel.

**References**

1. Sprung Ch, Maia P, Bulow H-H, et al. The importance of religious affiliation and culture on end of life decisions in European intensive care units. Intensive Care Med 2007;33:1732-39.

2. Ferrand E, Lemaire F, Regnier B, et al. Discrepancies between perceptions by physicians and nursing staff of intensive care unit end-of-life decisions. Am J Respir Crit Care Med 2003;167:1310-5.

3. Eysenck H.J Eysenck S.B.G. manual of the Eysenck personality questionnaire (EPQ) Hodder and Stoyghton educational London, UK.: 1975.

4. Bussing A, Ostermann T, Matthiessen PF, et al. Role of religion and spirituality in medical patients: confirmatory results with the SpREUK questionnaire. Health Qual Life Outcomes 2005;3:1-10.

### A45 End of life care in the intensive care unit - an audit cycle and quality improvement project

#### J. Aron, G. Lumley, D. Milliken, K. Dhadwal

##### Royal Free Hospital, Intensive Care Unit, London, United Kingdom

###### **Correspondence:** J. Aron – Royal Free Hospital, Intensive Care Unit, London, United Kingdom

**Introduction:** The decision to limit or withdraw active treatment in patients admitted to the intensive care unit (ICU) is multi-factorial (1). Prolonged periods of organ support in elderly, frail patients may not be beneficial and has resource implications. International guidelines suggest that goals of treatment should be set within 72 hours of admission to the ICU (2).

**Objectives:** We aim to identify the frequency of end-of-life decision-making and the effectiveness in communicating these decisions to the ICU team.

**Methods:** A prospective audit cycle was completed involving unplanned ICU admissions during a one-week period. Demographic information, APACHE II score, clinical frailty score (CFS), limitations of treatment (LOT) and awareness of these decisions were all documented. A re-audit using the same methodology was conducted after CFS and communication proformas were introduced for unplanned admissions.

**Results:** 61 patients were admitted as unplanned admissions during the first audit period. Patients were elderly, had a high predicted mortality and a significant proportion were clinically frail (CFS ≥ 5). Mean length of stay (LOS) was prolonged and level of organ support was high. These results are summarised in Table 25.

LOT was instigated in 9 (11.5 %) patients. Age and APACHE II scores were similar in patients with LOT and those with none. Frail patients had a higher LOS and received more organ support compared to non-frail patients. The proportion of patients with LOT remained low despite an increasing LOS. Communication between team members was sub-standard with 33 % of bedside nurses and 44 % of residents being unaware of these decisions.

The re-audit after intervention demonstrated an increase in patients receiving LOT, with a higher proportion of frail patients being represented. These patients also received less organ support and LOT was instigated earlier in their stay. Awareness regarding these decisions amongst all staff improved to 100 %. Feedback regarding the communication tools was excellent.

**Conclusions:** End of life care was performed infrequently in unplanned admissions to the ICU. When instigated, knowledge of these plans was substandard. Introducing CFS on admission to ICU identified patients who were unlikely to benefit from prolonged organ support, thus increasing LOT decisions. The communication sheets improved team communication. This has now become standard practice in two London ICUs.

**References**

1. Le Maguet et al. Prevalence and impact of frailty on mortality in elderly ICU patients: a prospective, multicenter, observational study. Intensive Care Med. 2014 May;40(5):674-82.

2. Dellinger RP, et al for the Surviving Sepsis Campaign Guidelines Committee including the Pediatric Subgroup. Surviving sepsis campaign: international guidelines for management of severe sepsis and septic shock: 2012. Crit Care Med. 2013 Feb;41(2):580-637

**Table 25 (abstract A45).** Results overview from the audit cycle

	No Treatment Limits (first audit)	Treatment Limits (first audit)	Treatment limits after interventions	Changes observed after interventions
Mean Age/range (years)	67.5 (44- 89)	73.33 (47 - 91)	82.5 (62-88)	More elderly population
APACHE II (mean)	22.3	21.8	22.3	No change in disease severity
CFS < 5, n (%)	29 (58.2%)	2 (22%)	2 (12.5%)	
CFS > 5, n (%)	23 (41.8%)	7 (78%)	14 (87.5%)	Higher proportion of frail patients with LOT
LOS > 72 hours n/total, (%)	35/39 (90.0%)	4/39 (10.3%)	8/36 (22.2%)	Higher proportion at 72 hours with LOT
LOS > 30 days, n/total (%)	11/14 (78.6%)	3/14 (21.4%)	7/11 (63.6%)	Higher proportion at 30 days with LOT
Awareness of decisions - Nurses, n (%)	49 (89.1%)	6 (66.7%)	16 (100%)	Improved awareness
Awareness of decisions - Resident, n (%)	51 (92.7%)	5 (55.6%)	16 (100%)	Improved awareness

## PATIENT SAFETY IN THE ICU

### A46 Improvements in the safety and quality of care in four UK NHS hospitals participating in the global tracheostomy collaborative

#### B.A. McGrath^1,2^, S.J. Lynch^1^, B. Bovento^1^, G. Sharpe^1^, E. Grainger^1^, S. Pieri-Davies^1^, S. Wallace^1^

##### ^1^University Hospital South Manchester, Manchester, United Kingdom, ^2^Manchester Academic Health Sciences Centre, University of Manchester, Manchester, United Kingdom

###### **Correspondence:** B.A. McGrath – University Hospital South Manchester, Manchester, United Kingdom

**Introduction:** Tracheostomies are used in the management of around 10 % of ICU admissions. Increasingly complex patients require truly multidisciplinary care to reduce the well described preventable harm that may occur.[1,2] The Global Tracheostomy (Quality Improvement) Collaborative (GTC) offers resources for participating hospital sites. Whilst individual elements of the GTC programme have been shown to be of benefit in individual sites, implementation of the package of resources and the ability of the GTC database to track and benchmark healthcare improvements had not been evaluated.

**Objectives:** The primary aim of the project was to implement the GTC into four diverse NHS hospitals and to evaluate impact on the safety and quality of care delivered. We hypothesised that systematic healthcare improvements would reduce the severity of harm resulting from tracheostomy-related safety incidents and improve surrogate markers of the quality of patient-centred outcomes such as time to first vocalisation.

**Methods:** As part of a Health Foundation funded quality improvement project, we introduced the GTC into four NHS sites in Manchester, England between August 2014 and August 2015. Interventions included multidisciplinary staff educational courses, webinars, standardisation of protocols and development of multidisciplinary tracheostomy teams. Local projects were overseen by local staff and patient champions. Data were collected using the bespoke GTC database and monthly trends in surrogate and patient safety incidents were analysed. Monthly incident rates were analysed using Chi Square test for linear trend.

**Fig. 22 (abstract A46).**
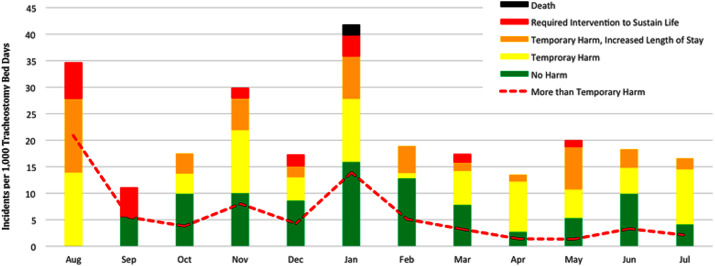
Trends in reported patient safety incidents

**Results:** Over the 12 month data collection period 296 tracheostomy patient admissions were tracked across the four sites with similar demographics to previously reported national data.[1] A total of 124 adverse events were identified affecting 29.8 % patients. Analysis of reported incidents over the duration of the project showed a significant reduction in the severity of harm by month (Chi Square p < 0.01, Fig. 22). There was also a significant trend towards lower harm categories for incidents over the duration of the project (Chi Square test for linear trend, r = -0.21, p < 0.01). Monthly analysis of the dataset for percutaneous tracheostomies showed non-significant trend toward earlier speaking valve use and vocalisation (median slope = -0.17, -0.83 to 0.4) associated with improvements in reported patient satisfaction scores.

**Conclusions:** Our study has demonstrated that meaningful improvements in the safety and quality of care for patients with tracheostomies are possible using improvement methodology advocated by the GTC in diverse NHS Trusts.

**References**

1. ‘On the right trach?’ NCEPOD 2014. www.ncepod.org

2. McGrath BA,BJA 2015;115(2):155-8.

**Grant acknowledgement**

This work was supported by the Health Foundation, an independent charity committed to bringing about better health and health care for people in the UK.

### A47 The impact of multidisciplinary tracheostomy safety teams on hospital length of stay for patients with tracheostomy

#### B. McGrath^1,2^, S.J. Lynch^1^, B. Bovento^1^, E. Grainger^1^, S. Pieri-Davies^1^, G. Sharpe^1^, S. Wallace^1^

##### ^1^University Hospital South Manchester, Manchester, United Kingdom; ^2^Manchester Academic Health Sciences Centre, University of Manchester, Manchester, United Kingdom

###### **Correspondence:** B. McGrath – University Hospital South Manchester, Manchester, United Kingdom

**Introduction:** Around 12-14,000 tracheostomies are performed by intensivists in critically ill patients in England and Wales annually. Patients are increasingly complex and high quality care requires the input of a wide range of multidisciplinary healthcare professionals, including medical, nursing, speech and language pathology and specialist physiotherapy staff. National reports have identified disjointed and uncoordinated care that leads to delays in progressing care.[1,2,3] The Global Tracheotomy (Quality Improvement) Collaborative (GTC) brings together resources from international exemplar centres. Multidisciplinary tracheostomy teams (MDTT) are a key intervention. We introduced GTC MDTT and resources into four diverse NHS sites and performed a detailed evaluation of their impact.

**Objectives:** We hypothesised that systematic healthcare improvements that raised the quality and safety of tracheostomy services would lead to more efficient care, measured by earlier tracheostomy decannulation times and reduced hospital lengths of stay.

**Methods:** We introduced MDTT into four diverse NHS sites in Manchester, England as part of a Health Foundation funded project between August 2014 and August 2015, measuring outcomes using the GTC database. Each site set up a MDTT, supported by the resources of the GTC (via www.globaltrach.org). Webinars offered opportunities for question and answer sessions around how obstacles to changes in care delivery had been overcome. MDTTs reviewed tracheostomy patients weekly and provided a point of contact and education for bedside ICU and ward staff. The largest site (UHSM) kept detailed MDTT records over 40 weeks, with all four sites recording individual patient metrics via the GTC database. Length of stay (LoS) data were plotted monthly with box-and-whisker plots. All data points were retained for non-parametric linear regression analysis.

**Results:** UHSM MDTT undertook 155 reviews, making 184 interventions. There was a significant month-by-month trend towards reducing LoS across all sites for all 296 patients, with median hospital LoS reduced by 6 days over the 12 month project (95%CI 9.96-3.96). For the 214 newly inserted tracheostomies, there was a non-significant trend towards reduced tracheostomy time (median slope -0.05, -0.17 to 0.25). ICU LoS reduced significantly over the duration of the project with a median slope of -0.11 (-0.25 to 0), equating to a reduction of 1.3 days per patient.

**Conclusions:** Introducing MDTT can coordinate and improve the quality and safety of care provided to ICU and ward tracheostomy patients. Improvements are reflected in reductions in ICU and hospital LoS.

**References**

1. McGrath BA, Otolaryngology-Head&Neck Surgery 2015;153(2):167-9

2. McGrath B, Clin Otolaryngol. 2013;38(6):541-545

3. Hettige R, BJIC 2013(Autumn):89-92

**Grant acknowledgement**

Funded by the Health Foundation, an independent charity committed to bringing about better health and health care for people in the UK.

### A49 The relationship between nurse staffing levels and mortality in the pediatric intensive care unit

#### M. Jung, J. Cho, H. Park, G. Suh

##### Samsung Medical Center, Sungkyunkwan University School of Medicine, Department of Critical Care Medicine, Seoul, Korea, Republic of

###### **Correspondence:** M. Jung – Samsung Medical Center, Sungkyunkwan University School of Medicine, Department of Critical Care Medicine, Seoul, Korea, Republic of

**Introduction:** Previous studies showed that low nurse staffing levels were associated with poor outcomes and increased occurrence of adverse events. However, national data are scarce regarding the relationship between the nurse staffing ratio and pediatric patient outcomes.

**Objectives:** To investigate the association between nurse staffing levels and the outcome of pediatric patients in Korean intensive care units (ICUs).

**Methods:** This retrospective cohort study used data from National Health Insurance claims. The included patients were under 18 years old and admitted to ICUs (but not neonatal ICUs) between August 2009 and September 2014. The ICU nurse staffing was graded into nine levels according to the total bed to nurse ratio for each hospital (National Health Insurance policy). The lowest and highest bed-to-nurse ratios were 0.5:1 and 2:1, respectively. We inspected the differences between overall patient mortality and the nursing staff grades at discharge. We also analyzed the mortality of patients who underwent mechanical ventilator care for more than 3 hours according to the nursing staff grades.

**Results:** During the study period, 39,917 medical and surgical pediatric patients were admitted to the ICUs. Mechanical ventilation was administered to 45.9 % of these patients. The median (interquartile range) ICU length of stay was 2 (1-6) days. The overall hospital mortality rate was 5.5 %, and the mortality rate of mechanically ventilated patients was 10.8 %. About half of the patients (18,053; 45.2 %) received the highest grade ICU treatment. The overall mortality rate did not increase with a higher bed-to-nurse ratio (i.e., lower nurse staffing level). However, a trend toward increased mortality was observed in mechanically ventilated patients as the bed-to-nurse ratio increased. The mortality of mechanically ventilated patients was 7.6 % for a bed-to-nurse ratio < 0.5:1, 16.7 % for a 0.63-0.77:1 ratio, 25.2 % for a 0.88-1:1 ratio, and 38.1 % for a > 2:1 ratio.

**Conclusions:** The nursing staff to bed ratio may not be associated with the overall mortality of pediatric patients at discharge. However, a low nursing staff level was associated with a higher mortality in pediatric patients who required mechanical ventilators.

### A50 Exposure keratopathy in critically ill adults: incidence, risk factors and impact of protocolised care

#### O. Kousha, J. Paddle

##### Royal Cornwall Hospital, Intensive Care Unit, Truro, United Kingdom

###### J. Paddle – Royal Cornwall Hospital, Intensive Care Unit, Truro, United Kingdom

**Introduction:** Exposure keratopathy (EK) is a clinical syndrome characterised by incomplete eyelid closure and corneal wetting leading to corneal damage of variable severity and extent. It has an incidence in critically ill patients of between 10 and 60 % (1).

**Objectives:** This study aimed to determine the rate of EK in patients admitted to critical care (ICU) and identify risk factors for developing EK. Using the identified risk factors and experience from the first part of the study, we developed an eye-care protocol. Finally, we studied the effectiveness of the protocol to prevent EK.

**Methods:** We undertook a two-phase prospective cohort single-centre study between November 2014 and August 2015 in a general adult ICU in the United Kingdom. The first phase of the study was observational. In the second phase of the study an eye-care protocol was introduced. Ethical approval was waived by the Trust ethics committee. Inclusion criteria were all patients admitted to ICU. Exclusion criteria were age < 16, known eye disease, patient agitation, and refusal to participate. The patient remained in the study until ICU discharge or withdrawal of active therapy. An eye-care protocol was developed and incorporated into the electronic patient record.All data were collected by a single investigator. Data were APACHE II score, daily SOFA, mechanical ventilation, Richmond Agitation-Sedation Scale, and level of eye care. Ophthalmic assessment was examination of the external eye, eyelids, and ocular surface using a portable slit lamp pre- and post-fluorescein dye instillation. Statistical tests were two-tailed (α = 0.05). Student t-test was used for continuous data and χ2 or Fisher's exact test for binary data. Logistic regression was used to analyse the relationship between EK and independent variables. Relative risk (RR) and Odds ratio (OR) were calculated with 95 % confidence intervals.

**Results:** We studied 371 patients. The overall rate of EK was 21 %. Among mechanically ventilated patients the rate was 54.3 % compared to 5.1 % in patients receiving non-invasive or no ventilatory support: RR = 10.6 (5.5-20.7), p < 0.001. OR for development of EK was 6.8 (3.2-8.0), p = 0.028 with mechanical ventilation and 32.5 (15.3-45.1), p < 0.001 with lagophthalmos. SOFA was associated with EK with OR 1.3 (1.1-1.5), p = 0.016. We found no independent effect of age, sedation or APACHE II score. Following the introduction of the protocol in the second phase of the study, the rate of EK reduced to 2.6 % (3 cases): RR = 7.8 (3.5-0.5), p < 0.001. The association with mechanical ventilation and lagophthalmos remained. Compliance with the protocol was 97 %.

**Conclusions:** EK is common in critically ill patients, and is associated with mechanical ventilation and lagophthalmos. A simple protocol substantially reduced the incidence of EK and was easily achieved in clinical practice.

**Reference**

1. Kuruvilla S, Peter J, David S et al. J Crit Care. 2015:30;400-404

**Grant acknowledgement**

None received

## Poster Corner Sessions: NUTRITION AND HORMONAL DISTURBANCES

### A51 An attenuated rate of leg muscle protein depletion over time is seen in ICU longstayers

#### L. Gamrin Gripenberg, M. Sundström Rehal, J. Wernerman, O. Rooyackers

##### Karolinska University Hospital Huddinge, Stockholm, Sweden

###### **Correspondence:** L. Gamrin Gripenberg – Karolinska University Hospital Huddinge, Stockholm, Sweden

**Introduction:** The loss of muscle mass in critical illness is caused by a mismatch between synthesis and degradation, where synthesis is near-normal, while degradation is enhanced, resulting in a negative protein balance. A muscle mass loss of 10 % per week is reported. Studies supporting this mechanism are almost exclusively from patients during the initial two weeks of critical illness.

**Objectives:** To investigate if this mechanism is valid also in a longer time perspective we studied critical ill patients during days 10-40 of ICU stay.

**Methods:** Critically ill patients on mechanical ventilation (n = 20) were included, multiple times if possible. In total 30 measurements of muscle protein turnover were performed employing a 3-pool model technique with a constant infusion of d5-phenylalanine, leg blood flow measurements, and biopsies to determine tissue enrichments.

**Results:** Net protein balance of leg mixed muscle showed a pattern of becoming less negative over time with regression analyses. This pattern was totally attributable to an increase in protein synthesis rate, while protein breakdown rate, although higher than in healthy subjects, was completely unaltered over time. Net protein balance during days 10-20 (-21 ± 21 nmol phenylalanine/min/100 g muscle) was lower (p = 0.002; T-test) than during days 30-40 (1 ± 11 nmol phenylalanine/min/100 g muscle).

**Conclusions:** The temporal pattern of protein turnover in leg muscle showed a pattern of diminished protein losses over time in surviving patients staying in the ICU. How this pattern relates to nutrition, mobilization, and pharmacology remains to be established.

### A52 Pharmacokinetics of four high-dose regimes of intravenous vitamin C in critically ill patients

#### H.-J. de Grooth^1^, W.-P. Choo^2^, A.M. Spoelstra - de Man^1^, E.L. Swart^2^, H.M. Oudemans-van Straaten^1^

##### ^1^VU University Medical Center, Department of Intensive Care, Amsterdam, Netherlands; ^2^VU University Medical Center, Department of Clinical Pharmacology and Pharmacy, Amsterdam, Netherlands

###### **Correspondence:** H.-J. de Grooth – VU University Medical Center, Department of Intensive Care, Amsterdam, Netherlands

**Introduction:** Critically ill patients exhibit a high degree of vitamin C deficiency at ICU admission and plasma concentrations decrease even more during the following days [1, 2]. High intravenous (iv) doses of vitamin C are required to increase plasma concentrations to normal and supra-normal ranges [2], but the optimal dosage regime in this population remains unclear. Prolonged vitamin C administration may be associated with oxalate kidney stone formation, but the effect of short-term high-dose supplementation on urinary oxalate excretion is unknown.

**Study design and objectives:** We conducted a prospective randomized controlled trial to determine the pharmacokinetics of four high dose regimes of iv Vitamin C in critically ill patients and to measure oxalate excretion.

**Patients:.** Adult patients admitted to the ICU with sepsis or SIRS, with a non-neurological sequential organ failure assessment (SOFA) score >6 and an expected length of ICU stay >96 hours.

**Intervention:** Patients were randomized to either 1 g or 5 g vitamin C twice daily as a 30-min bolus infusion, or to 2 g or 10 g daily as a continuous infusion. Vitamin C administration was continued for 48 hours, so that all patients received a total dose of either 4 g or 20 g vitamin C.

**Measurements:** Concentrations of vitamin C were determined in plasma at t = 0, 1, 2, 4, 8, 12, 24, 36, 48, 72 and 96 hours. Urine vitamin C and oxalate concentrations were determined during the first and last 12 hours of vitamin C administration. NONMEM was used for the pharmacokinetic analysis.

**Fig. 23 (abstract A52).**
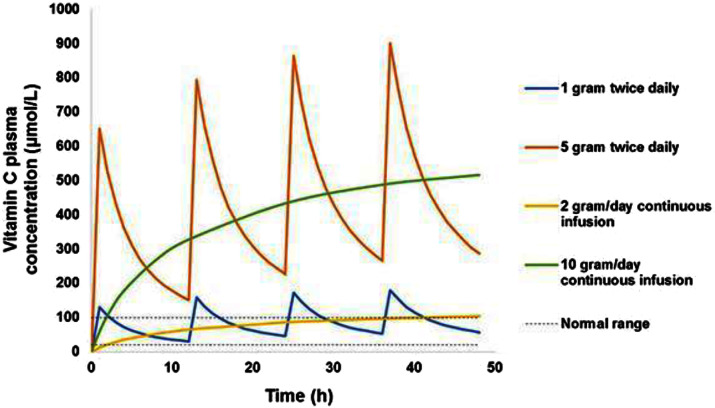
Predicted Vitamin C plasma concentrations

**Results:** Fourteen patients were included: 5 patients received 1 g vitamin C and 5 patients 5 g vitamin C iv twice daily for two days. Two patients received 2 g/day vitamin C and 2 patients received 10 g/day by continuous infusion for two days (Table 26). Four patients (28 %) were vitamin C deficient on admission (<20 μmol/L). A two-compartment pharmacokinetic model best described the data (Fig. 23, model parameters not shown). The urinary excretion of vitamin C and oxalate is shown in Table 27.

**Conclusion:** Normal vitamin C plasma levels are attained with either 1 g iv twice daily or 2 g as continuous infusion. A bolus infusion is needed for rapid achievement of high-normal plasma concentrations. Both high-dose regimes produce supra-normal plasma concentrations. With continuous infusion, urinary vitamin C loss was lower and a higher proportion remained in the body. The urinary excretion of oxalic acid increased with higher vitamin C doses.

**References**

[1] Schorah et al. Total vitamin C, ascorbic acid, and dehydroascorbic acid concentrations in plasma of critically ill patients. Am. J. Clin. Nutr. 63, 760-5 (1996).

[2] Long et al. Ascorbic acid dynamics in the seriously ill and injured. J. Surg. Res. 109, 144-148 (2003).

**Table 26 (abstract A52).** Characteristics of included patients

	B.i.d. bolus regime		Continuous infusion regime	
	2 × 1g / day N = 5	2 × 5g / day N = 5	2g / day N = 2	10g / day N = 2
Age, years: med (min-max)	64 (37 - 79)	67 (59 - 80)	68 (58 - 78)	74.5 (70 - 79)
Gender male: n (%)	4 (80)	3 (60)	1 (50)	1 (50)
Weight, kg: med (min-max)	78 (71 - 119)	72 (42 - 90)	87 (73 - 100)	85 (80 - 90)
Recent surgery: n (%)	4 (80)	3 (60)	0 (0)	1 (50)
Admission SOFA score: med (min-max)	8 (7 - 12)	7 (7 - 7)	8 (7 - 9)	12.5 (11 - 14)
Admission Vitamin C plasma concentration, μmol/L: med (min-max)	44 (19 - 86)	31 (9 - 51)	19 (12 - 25)	26 (20 - 32)

**Table 27 (abstract A52).** Excretion of Vitamin C and oxalate

	*B.i.d. bolus regime*		*Continuous infusion regime*	
	2 x 1g / day N = 4^a^	2 x 5g / day N = 5	2g / day N = 2	10g / day N = 2
Vitamin C excretion 36-48h, mg: med (min - max)				
T = 0-12 hours	569 (183 - 656)	3760 (1905 - 5367)	13 (11 - 15)	1480 (1176 - 1783)
T = 36-48 hours	918 (791 - 1131)	6573 (3975 - 7235)	339 (120 - 558)	3934 (3569 - 4299)
Oxalate excretion 36-48h, mg: med (min - max)				
T = 0-12 hours	30 (13 - 77)	43 (18 - 84)	20 (13 - 27)	36 (26 - 45)
T = 36-48 hours	38 (25 - 82)	76 (27 - 130)	58 (39 - 76)	84 (60 - 108)

Normal oxalate excretion ≤ 20 mg/12h

^**a**^ Missing urine data from 1 patient because of anuric acute kidney failure

### A53 Adrenal exhaustion during prolonged icu stay in critically ill medical patients

#### L. Talan, G. Güven, N.D. Altıntas

##### Ankara University Faculty of Medicine, Department of Internal Medicine Division of Intensive Care, Ankara, Turkey

###### **Correspondence:** L. Talan – Ankara University Faculty of Medicine, Department of Internal Medicine Division of Intensive Care, Ankara, Turkey

**Introduction:** Effects of glucocorticoid therapy for septic shock patients have been the subject of many studies. Following the study by Annane et al, which has shown that some patients develop critical illness related adrenal insufficiency early during the course of sepsis, changes in adrenal functions during sepsis has gained attention. There have been recent reports that adrenal functions may decrease during the course of critical illness, a condition often overlooked. However prevalence of adrenal exhaustion in critically ill medical patients and associated factors are unknown.

**Objectives:** The objective of the study was to determine the prevalence of adrenal exhaustion in medical patients with prolonged critical illness and the factors associated with it.

**Methods:** This observational study was performed in a medical intensive care unit (ICU) of a university hospital. Admission cortisol levels were measured in all septic shock patients initially unresponsive to fluid resuscitation and vasopressors. Consequently, patients with initial cortisol levels >15 μg/dl were followed and cortisol testing was repeated in patients with ongoing vasopressor dependency with no other underlying cause.

**Results:** A total of 37 septic shock patients were screened on ICU admission. Of these, 19 patients had cortisol levels >15 μg/dl and underwent cortisol retesting for ongoing vasopressor need during the same ICU admission. Eleven (%58) had serum cortisol levels < 15 μg/dl. Mean (±SD) age was 70 ± 13.5 years. APACHEII and Sequential Organ Failure Assessment scores on ICU admission were 25.3 ± 6.5 and 10.4 ± 5.2, respectively. Age, gender, APACHEII and SOFA scores, serum albumin, protein, CRP and procalcitonin levels were similar between the groups. However, at the time of cortisol retesting, patients with lower cortisol levels had significantly longer length of ICU stay (p = 0.038). Time interval between two cortisol sampling was (median, IQR) 12 (8-30) days. Time interval between higher and lower cortisol groups did not reach statistical significance. When glucocorticoid therapy was begun in the lower cortisol group, vasopressors were weaned within 48 hours; vasopressor weaning was longer and varied in higher cortisol group.

**Conclusions:** Adrenal response is a dynamic process. During the course of critical illness, as with other organs, adrenal failure may develop. With these findings, adrenal exhaustion seems to be a complication of prolonged critical illness. We suggest prolonged vasopressor dependency should prompt a search for adrenal exhaustion, even if initial cortisol testing results are within normal limits. Identifying patients that could benefit from corticosteroid therapy may be a life-saving measure.

**References**

1. Jorge A, Guzman MD, et al.Adrenal exhaustion in septic patients with vasopressor dependency. J Cri Care 2007; 22, 319-323.

2. Wu JY, Hsu SC, et al.Adrenal insufficiency in prolonged critical illness. Crit Care 2008;12:R65.

**Grant acknowledgement**

None.

### A54 Use of nurse-driven feeding protocol improves enteral caloric intake: observational single centre before-and-after study

#### M. Padar^1^, G. Uusvel^1^, L. Starkopf^2^, J. Starkopf^1,3^, A. Reintam Blaser^3,4^

##### ^1^Tartu University Hospital, Department of Anaesthesiology and Intensive Care, Tartu, Estonia; ^2^University of Copenhagen, Faculty of Health and Medical Sciences, Section of Biostatistics, Institute of Public Health, Copenhagen, Denmark; ^3^University of Tartu, Department of Anaesthesiology and Intensive Care, Tartu, Estonia; ^4^Lucerne Cantonal Hospital, Department of Anaesthesiology, Intensive Care, Emergency and Pain Medicine, Lucerne, Switzerland

###### **Correspondence:** M. Padar – Tartu University Hospital, Department of Anaesthesiology and Intensive Care, Tartu, Estonia

**Introduction:** Early and sufficient enteral intake is associated with reduced ICU morbidity and mortality. Limited data suggests that use of nurse-driven feeding protocol with defined targets may facilitate nutrition and improve the outcomes.

**Objective:** To investigate whether implementation of nurse-driven feeding protocol results in increased enteral caloric intake in critically ill patients during their first week in the ICU.

**Methods:** We performed an uncontrolled before-and-after study. Data of consecutive adult patients, readmissions excluded, treated for at least 7 days in General ICU of Tartu University Hospital, were extracted from existing database. In 2013, nurse-driven feeding protocol was instituted in the department [1]. To analyze outcomes of this implementation, patients treated from 2011 to 2012 were included in Before, and patients from 2014 to 2015 in After group.

**Fig. 24 (abstract A54).**
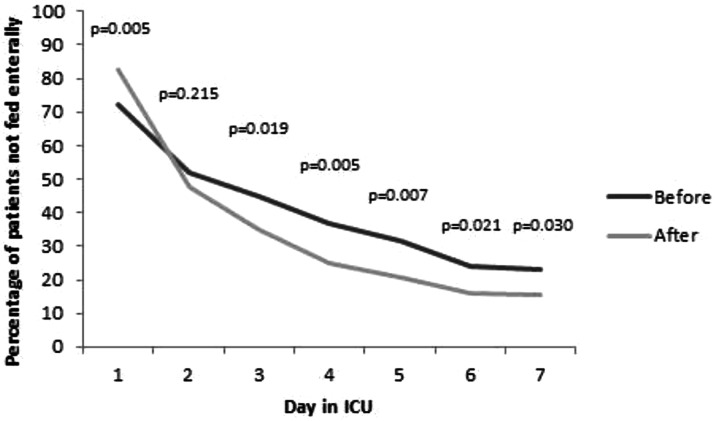
Percentage of patients not fed enterally

**Results:** 231 patients were included in Before and 225 in After group, respectively. The groups are comparable regarding demographics, case-mix and severity of illness. Instalment of feeding protocol resulted in significantly higher cumulative amount of enterally provided calories by day 7 [3165 (1165-5215) kcal in After vs 2360 (450-5075) kcal in Before group, median (IQR), p = 0.043], while less calories were given parenterally [2600 (712-4287) vs 3900 (1725-6645) kcal, p < 0.001]. Cumulative proportion of patients who did not receive any enteral feed was significantly smaller in After group (Fig. 24).

**Fig. 25 (abstract A54).**
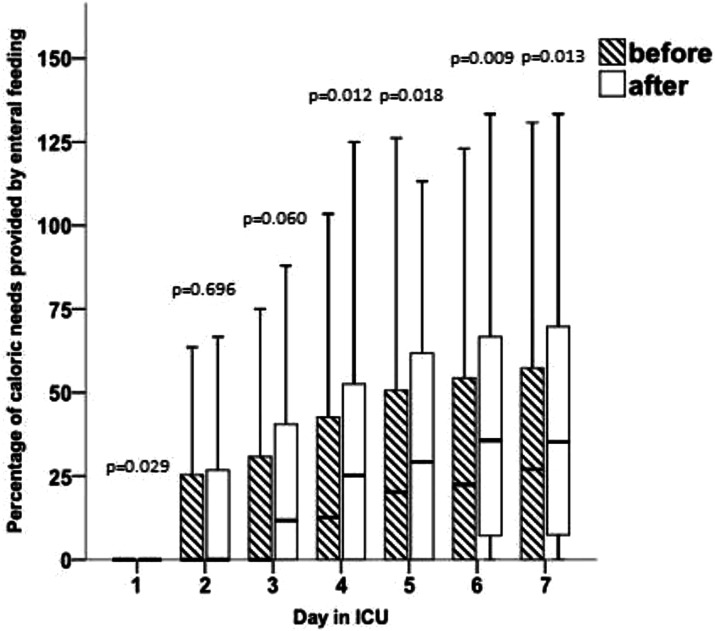
Daily proportions of enteral calories

Percentage of enterally received calories from caloric needs was significantly higher in After group (Fig. 25).

Prevalence of GI symptoms and intra-abdominal hypertension was not different between the groups. ICU length of stay was significantly shorter in After group (11 vs 13 days, respectively, p < 0.001), but no difference was noted in duration of mechanical ventilation and ICU mortality.

**Conclusions:** Use of nurse-driven feeding protocol in ICU patients is associated with improved enteral nutrition without an increase of GI complications.

**References**

1. Kuslapuu M, Jõgela K, Starkopf J, Reintam Blaser A. The reasons for insufficient enteral feeding in an intensive care unit: A prospective observational study. Intensive Crit Care Nurs. 2015 Oct;31(5):309-14

**Grant acknowledgement:**

Ministry of Education and Research of Estonia (IUT34-24)

### A55 Nutritional support in mechanically ventilated patients: are we doing enough?

#### M.S. Kalaiselvan^1^, A.S. Arunkumar^1^, M.K. Renuka^2^, R.L. Shivkumar^1^

##### ^1^Sri Ramachandra University, Department of Critical Care Medicine, Chennai, India; ^2^Sri Ramachandra University, Department of Anesthesiology, Chennai, India

###### **Correspondence:** M.S. Kalaiselvan – Sri Ramachandra University, Department of Critical Care Medicine, Chennai, India

**Introduction:** Enteral nutritional is an essential component of critical care. Adequate nutrition is essential for recovery from critical illness; malnutrition has been associated with poor patient outcomes in intensive care units.

**Objectives:** To estimate the adequacy of nutritional in mechanically ventilated patients and its effect on patient outcomes.

**Methods:** This was a prospective observational study, done over a period of one year(Jan.-Dec. 2015}). All adult patients mechanically ventilated for >48 hours were included in the study. Patients were started on protocol driven continuous enteral nutrition, targeted to achieve 25 Kcal/kg/day of energy and 1 gm/kg/day of protein. Data was collected on demography, admission severity of illness, hemodynamics, subjective global nutritional assessment (SGA), and lead time to initiation and achievement of full feeds, adequacy of energy and proteins supplied reasons for feeds interruptions and complications of enteral nutrition. All data on nutrition was collected till the patient stayed on ventilator. Primary outcome was time to achieve full feeds; secondary endpoints were time to initiate feeds and reason for interrupting feeds.

**Results:** 374 patients fit into the inclusion criteria. Majority were males (64 %), mean age of patients was 55.1(±17.8) years and their BMI was 24(±3.5). APACHE II 22.3 (±5.9) and SOFA 7.2(±2.3) scores were high. 35.4 % of patients were malnourished on nutritional screening(SGA). Respiratory failure (59 %) was the most common reason for intubation followed by neurological deterioration and majority of patients were medical patients

Two patients(2/374) had nasogastric tube malpositioning on X-ray, which required re-insertion. 86.8 % received enteral feeds, 2.4 % received parenteral nutrition and 10.4 % received no feeds

Time to initiate enteral feeds was 12 hours (median) (IQR)(7.5-24) hours and it ranged from 1-130 hours

66.86 % of patients received 100 % of target calories in 39.2(±19.4) hours and 33.1 % of patients didn´t achieve their target calories. 372 interruptions of feeds occurred in 335 patients enterally fed; most common reasons for interruption was weaning (43.8 %) followed by airway related procedures (26.6 %) and these patients were restarted on feeds after 9.8(±5.1) hours following interruption of feeds. Patients received 70 % (mean-1069 Kcal) of their prescribed calories during their ventilator days. Mortality rate was 42.2 %, ICU ALOS and ventilator days were 7.7(±5.3) and 5.9(±4.2) days respectively.

**Conclusions:** Protocol driven enteral nutrition allows early feeding, with minimum interruptions of feeds in mechanically ventilated patients and their is still room for improvement.

**References**

Keng F Yip, Vineya Rai, Kang K Wong.Evaluation of delivery of enteral nutrition in mechanically ventilated Malaysian ICU patients. BMC Anesthesiology 2014, 14:127.

**Fig. 26 (abstract A55).**
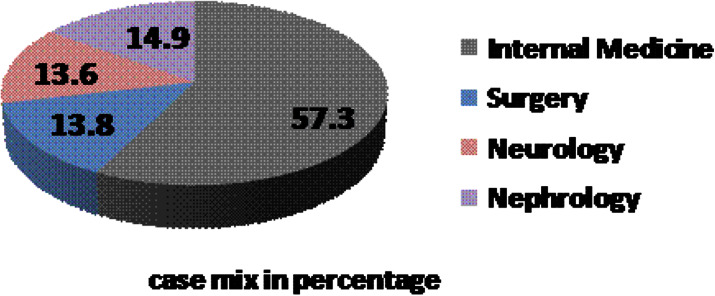
Case mix of patients

**Table 28 (abstract A55).** Enteral nutrition practices in mechanically ventil

Routes of nutrition	n=374(%)
Enteral nutrition	325(86.8)
Parenteral nutrition	9(2.4)
Enteral + parenteral nutrition	1(0.3)
No nutrition	39(10.5)
Reasons for not feeding	n=39(%)
Intestinal obstruction/Post-operative bowel surgery	10(25.6)
Hemodynamic instability	10(25.6)
Poisoning	9(23)
GI bleed	8(20.5)

**Fig. 27 (abstract A55).**
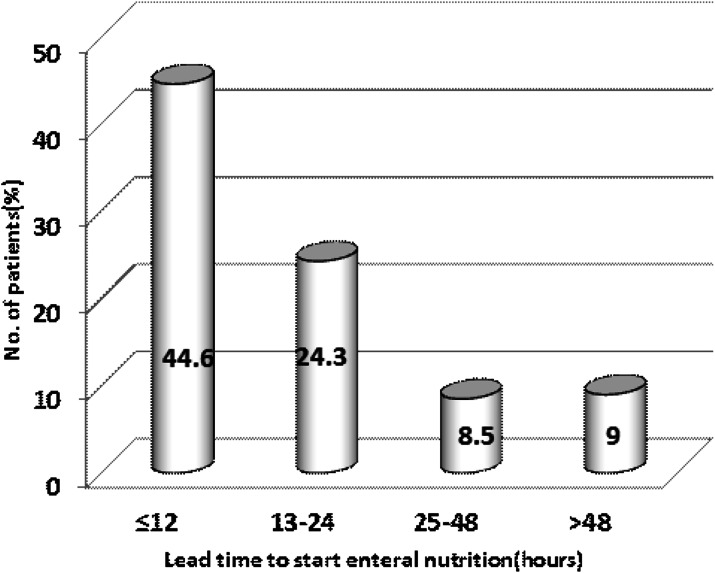
Lead time to initiate feeding(hours)

**Table 29 (abstract A55).** Calories achieved over days

	No.of patients (n)	Calories achieved mean(±SD) Kcal	Percentage of target calories achieved (%)
Day 1	309	656(424)	42.3
Day 2	284	1591(512)	88.7
Day 3	221	1517(512)	90
Day 4	176	1400(517)	73.4

42.3% of targeted calories were achieved on day1

**Fig. 28 (abstract A55).**
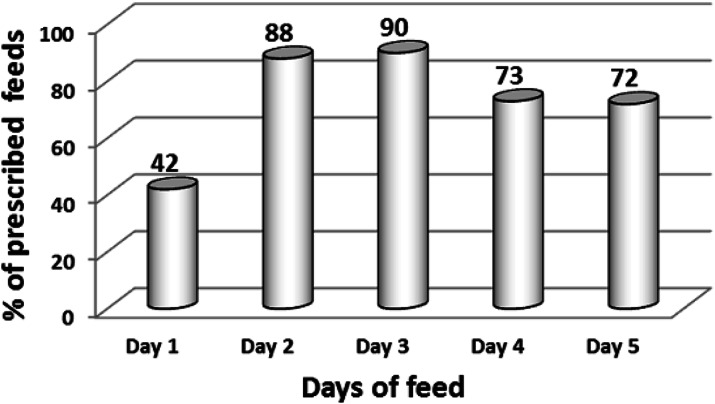
% of feeds received over days

**Table 30 (abstract A55).** Reasons for interrupting feeds

Reasons for interrupting feeds	n=372(%)
Weaning	163(43.8)
Airway-related procedures	99(26.6)
Free water ( for treatment of hypernatremia)	64(17.2)
Preparation for surgery /procedures	39(10.4)
GRV>500ml	10(2.6)
Diarrhoea	10(2.6)
Vomiting	6(1.6)

### A56 Prognostic value of post-operative cortisol in cardiothoracic surgery patients with or without high-dose dexamethasone

#### M. Volbeda^1^, D. ten Kate^1^, M. Hoekstra^1^, J.M. van der Maaten^2^, M.W. Nijsten^1^

##### ^1^University Medical Center Groningen, University of Groningen, Department of Intensive Care, Groningen, Netherlands; ^2^University Medical Center Groningen, University of Groningen, Departments of Intensive Care and Anesthesiology, Groningen, Netherlands

###### **Correspondence:** M. Volbeda – University Medical Center Groningen, University of Groningen, Department of Intensive Care, Groningen, Netherlands

**Introduction:** The recent randomized controlled DECS-trial demonstrated that high-dose dexamethasone before cardiac surgery with cardiopulmonary bypass decreased ICU length of stay [1]. In our center many patients undergo off-pump cardiac surgery for which no dexamethasone is administered. Post-operative cortisol levels might be associated with outcome [2, 3]. In this study we prospectively measured cortisol levels after cardiothoracic surgery in relation with outcome for patients who did or did not receive dexamethasone.

**Methods:** In 2013 and 2014 we prospectively collected post-operative cortisol levels obtained on the first morning after cardiothoracic surgery. Cortisol was determined in serum with a chemi-luminiscence assay. The use of dexamethasone (1 mg/kg) was recorded as well ICU and hospital length of stay and hospital and long-term mortality after an observation period of at least 1.5 years. Patients were divided into low or high cortisol according to the median cortisol measured in the dexamethasone or control patients.

**Results:** We included 927 patients, 70 % males with a mean ± SD age of 63 ± 13 years. Cardiopulmonary bypass was used in 488 (53 %) and high-dose dexamethasone was administered in 439 (47 %) of the operations. ICU and hospital length of stay were 2.3 ± 5.0 and 13.7 ± 12.5 days respectively. Hospital and long-term mortality were 2.2 % and 6.5 % respectively. Median (IQR) cortisol on the first day post-surgery was 600 (195-965) nmol/L (or 22 ug/dL). Median cortisol levels were lower in the dexa group than the control group (P < 0.0001). Patients in the dexa group had a median (IQR) cortisol of 205 (75-535) and were divided into low-cortisol and high cortisol groups (cortisol < =205 and >205 nmol/L respectively). Likewise control patients had a median cortisol of 830 (585-1125) and were divided into < =830 and >830 nmol/L groups. No significant differences in length of stay or mortality were observed between patients with a low or high cortisol level within the dexa and control groups.

**Conclusions:** Cortisol levels obtained one day after cardiac surgery are far lower in dexamethasone treated patients. Both in controls and dexamethasone-treated patients, lower cortisol levels were not associated with a worse outcome.

**References**

[1] Dieleman JM, Nierich AP, Rosseel PM, et al. Intraoperative high-dose dexamethasone for cardiac surgery: a randomized controlled trial. JAMA 2012; 308(17):1761-7.

[2] Venkatesh B, Cohen J, Cooper M. Ten false beliefs about cortisol in critically ill patients. Intensive Care Med 2015; 41:1817-19.

[3] Henzen C, Kobza R, Schwaller-Protzmann B, et al. Adrenal function during coronary artery bypass grafting. Eur J Endocrinol. 2003; 148(6):663-8.

### A57 Comparison of three techniques to estimate albumin catabolic rate

#### A. Komaromi, O. Rooyackers, J. Wernerman, Å. Norberg

##### Karolinska University Hospital Huddinge, Stockholm, Sweden

###### **Correspondence:** A. Komaromi – Karolinska University Hospital Huddinge, Stockholm, Sweden

**Introduction:** Plasma albumin concentration is low in critically ill patients despite a higher than normal synthesis rate. It is demonstrated that the capillary escape rate of albumin is increased, related to an enhanced capillary leakage, but the return to plasma through the lymphatic system is not characterized. Nevertheless it is necessary to postulate an enhanced degradation rate or increased volume of distribution, to explain this finding.

**Objectives:** We explored 3 different techniques to assess albumin turnover and degradation.

**Methods:** Healthy volunteers (n = 10) were studied over 42 days. The first day, albumin synthesis rate was measured in the postabsorptive state by the in vivo incorporation of d5-phenylalanine, and a dose of ^125^I-albumin was given to assess albumin degradation. Degradation rate was measured and calculated by the decay in radioactivity in 2 ways; in urine collected over 24 hours at 3 separate days, and in plasma repeatedly over 42 days.

**Results:** Basal albumin synthesis rate was 118 ± 16 mg/kg/day, equal to degradation rate in steady state. Catabolic rate by urine sampling was 142 ± 31 mg/kg/day (P = 0.022 v. basal), and by plasma 177 ± 29 mg/kg/day (P = 0.001 v. basal).

**Conclusions:** The differences in calculated turnover rates may correspond to effects of feeding or differences in half-lives between exogenous and endogenous albumin. The exact explanation needs to be further investigated.

### A58 The relation between plasma glutamine concentration and endogenous glutamine production

#### M. Smedberg, M. Mori, L. Pettersson, Å. Norberg, O. Rooyackers, J. Wernerman

##### Karolinska University Hospital Huddinge, Stockholm, Sweden

###### **Correspondence:** M. Smedberg – Karolinska University Hospital Huddinge, Stockholm, Sweden

**Introduction:** A low plasma glutamine concentration at ICU admittance is associated with an unfavorable outcome. A number of studies have shown beneficial effects of exogenous glutamine supplementation to unselected critically ill patients. However, recently harm is reported when pharmacological doses of exogenous glutamine is given to underfed unselected ICU patients with ≥ 2 organ failures. The mechanism behind this finding is not understood and more specifically the reason for the low levels is not known.

**Objectives:** The relation between plasma glutamine concentration and glutamine production rates.

**Methods:** Critically ill patients (n = 17) with normal or low plasma glutamine concentration were studied. Glutamine rate of appearance reflection glutamine production rates was determined employing a bolus dose technique, and the decay curves were fitted into a single pool or a 2-pool model.

**Results:** Glutamine rate of appearance was in the range 5 - 15 umol/kg/min in ICU patients with plasma concentrations in the range300-700 umol/L. There was a statistical significant (P < 0.05) relation, but the coefficient of determination was low R^2^ = 0.15. Also the correlation between the 1-pool and 2-pool models was statistically significant (P < 0.01), but the coefficient of determination intermediary R^2^ = 0.60.

**Conclusions:** To understand the results of recent glutamine supplementation studies in the critically ill, the relevance of plasma glutamine levels must be under stood. In healthy volunteers there is a high concordance between 1-pool and 2-pool models to calculate the rate of appearance. The lower agreement in ICU patients indicates that a more extensive modeling may be necessary.

### A59 Effect of enteral versus parenteral nutrition on outcome of mechanically ventilated septic ICU patients

#### M. Theodorakopoulou, T. Christodoulopoulou, A. Diamantakis, F. Frantzeskaki, M. Kontogiorgi, E. Chrysanthopoulou, M. Lygnos, C. Diakaki, A. Armaganidis

##### Attikon University Hospital, ICU, Athens, Greece

###### **Correspondence:** M. Theodorakopoulou – Attikon University Hospital, ICU, Athens, Greece

**Introduction:** The prevalence of malnutrition in ICU has been estimated at up to 40 % with the majority of critically ill patients requiring nutritional support. Traditional teaching suggests that early enteral feeding in mechanically ventilated septic patients is superior to parenteral feeding due to a lower complication rate but neither form of support is without risk.

**Objectives:** In this study, we compared the outcomes in mechanically ventilated septic ICU patients receiving enteral versus parenteral nutrition.

**Methods:** A single centre study of patients admitted to a 25 bed University Hospital ICU over a period of three years. Demographics, severity of illness scores (APACHE and SOFA), BMI and MUST were measured upon admission. Daily nutrition requirements were calculated for each patient. Patients were randomized to enteral(EN) or parenteral nutrition(PN) group within 48 hours of intubation and admission to the unit. Duration of mechanical ventilation, ICU and hospital length of stay (LOS), and mortality rates were recorded.

**Results:** A total of 148 patients (76 men) mechanically ventilated septic patients having a mean(±SD) age of 69.6 ± 19.4 years were studied. All patients met the consensus criteria for sepsis. Baseline characteristics were similar in the two groups. APACHE II and SOFA at study entry were 24 ± 5 and 8 ± 3 respectively. The mean(±SD) BMI was ≈ 21.5 ± 3.4 kg/m^2^. Seventy seven (52,02 %) patients received EN, and sixty nine (47,26 %) received PN. There was no difference between the two groups for age, sex, BMI, and scores. ICU and hospital LOS were similar for both groups. ICU mortality rate was 29.4 % for PN group vs. 27.2 % for EN group indicating no significant difference. Hospital mortality was similar for both groups as well. In the PN fed group however, duration of mechanical ventilation was longer (*p* = .018), but the feeding goal was attained earlier (*p* = .009).

**Conclusions:** In mechanically ventilated septic ICU patients the ICU LOS and the hospital LOS, as well as the ICU and hospital mortality rates of patients receiving PN are not significantly different than those in patients receiving EN. Furthermore, feeding goals can be attained much easier by PN. Duration of mechanical ventilation however appears to be longer in patients receiving PN.

### A60 Association between the route of nutrition and adipokine hormones levels in critically ill patients: a pilot study

#### K. Gundogan^1^, E. Dogan^2^, R. Coskun^1^, S. Muhtaroglu^3^, M. Sungur^1^, T. Ziegler^4^, M. Guven^1^

##### ^1^Erciyes University, Intensive Care Unit, Kayseri, Turkey; ^2^Erciyes University, Internal Medicine Department, Kayseri, Turkey; ^3^Erciyes University, Clinical Biochemistry Department, Kayseri, Turkey; ^4^Emory University, Department of Medicine, Division of Endocrinology, Metabolism and Lipids, Atlanta, United States

###### **Correspondence:** K. Gundogan – Erciyes University, Intensive Care Unit, Kayseri, Turkey

**Introduction:** Adipokine hormones play an important role in regulation of insulin metabolism, body fat distribution and regulation of appetite and satiety. Some adipokine hormones have effects on inflammation and insulin resistance but the relation between these hormones and critical illness and the route of nutrition is not known.

**Objectives:** The aim of this study to determine association between nutrition route and adipokine hormones levels in critically ill patients

**Methods:** This study was performed prospectively in Medical and Surgical ICU at Erciyes University. Patients expected to stay in ICU at least 72 hours and received either parenteral or enteral nutrition included into the study.

**Results:** Total of 26 patients were included into the study and 17 of them were male (65 %). The mean age was 62.8 ± 18.2 years. Total of 14 patients (54 %) were fed via enteral route and 12 patients (46 %) were received parenteral nutrition. The mean APACHE II score was 22.7 ± 7.1. Resistin levels were lower in enteral nutrition group at 24th (p = 0.015) and 72nd hours (p = 0.014) compared to parenteral nutrition group. Baseline, 24.th hours and 72.th hours GLP-1 levels were found to be higher in enteral nutrition group than parenteral nutrition group (p = 0.031, p = 0.006 and p = 0.001 respectively). Adiponectin levels were significantly higher in enteral nutrition group compared to parenteral nutrition group at 72th hour (p = 0.014).

**Conclusions:** Our study showed that enteral nutrition helped to reverse abnormal process in critically ill patients with increasing adiponectin and GLP-1 levels and decreasing resistin levels.

**References**

1. Kwon H, Pessin JE. Adipokines mediate inflammation and insulin resistance. Frontiers in endocrinology. 2013;4:71.

2. Vassiliadi DA, Tzanela M, Kotanidou A, Orfanos SE, Nikitas N, Armaganidis A, et al. Serial changes in adiponectin and resistin in critically ill patients with sepsis: associations with sepsis phase, severity, and circulating cytokine levels. Journal of critical care. 2012;27(4):400-9.

### A61 Molecular mechanisms providing a switch from carbohydrate to fat metabolism in different organs in sepsis

#### A. Kleyman^1,2^, W. Khaliq^1^, D. Andreas^1^, M. Singer^1^

##### ^1^University College London, Bloomsbury Institute of Intensive Care Medicine, London, United Kingdom; ^2^University Hospital Jena, SG Sepsis Research, Department of Anaesthesiology and Intensive Care Medicine, Jena, Germany

###### **Correspondence:** A. Kleyman – University College London, Bloomsbury Institute of Intensive Care Medicine, London, United Kingdom

**Introduction:** A switch from carbohydrate to fat utilization is a hallmark of systemic inflammation. While this adaptive response occurs quickly and allows animals to survive under restricted food supply conditions, it will markedly affect cell metabolism.

**Objectives:** To elucidate molecular mechanisms underlying the substrate switch from carbohydrate to fat in different organs, we studied early alterations in (i) phosphorylation of enzymes involved in energy metabolism: AMP kinase (AMPK, thr172), acetylCoA carboxylase (ACC ser179), pyruvate dehydrogenase (PDK ser293), hormone sensitive lipase (HSL ser563); and (ii) expression of mitochondrial uncoupling proteins 2 and 3 in soleus and gastrocnemius skeletal muscle, liver, kidney and heart in sham-operated and septic rats at 6 h in our well-characterized 72 h fluid-resuscitated rat model of faecal peritonitis

**Methods:** Awake, instrumented yet fully mobile male Wistar rats (325 ± 15 g) received an i.p. injection of 4 μl/g faecal slurry. Fluid resuscitation (50:50 mix of 5 % glucose/Hartmann´s; 10 ml/kg/h) was commenced at 2 h. Control animals were treated identically except for slurry injection. At 6 h, an echo-measured heart rate cut-off of 460 bpm was used to classify animals into predicted survivors SR or non-survivors NSR.(1) Animals were killed and organs were immediately collected into liquid nitrogen. Alterations in protein phosphorylation and expression were studied by Western blot. Normalization was performed to loading control: actin or PFK. Results were presented as mean +/- SE, analyzed using Student's t-test and considered statistically significant when p < 0.05.

**Results:** At 6 h post-sepsis no differences were seen in renal and hepatic phosphorylation of AMPK, ACC, PDH and HSL between sham and septic animals. While cardiac ACC phosphorylation was strongly increased in septic rats, AMPK phosphorylation did not differ, suggesting that ACC phosphorylation was mediated not by AMPK but rather via the glucagon-PKA pathway. The biggest changes were observed in skeletal muscle. AMPK phosphorylation was increased in gastrocnemius and even more so in soleus in septic rats but this was not accompanied by a corresponding increase in ACC phosphorylation. In both muscles PDH phosphorylation markedly increased while PDH fell, suggesting a fall in pyruvate oxidative decarboxylation and glucose usage as a fuel. HSL phosphorylation was strongly increased in soleus in non-survivors. UCP2 and UCP3 levels were not altered in any organ. Table 31.

**Conclusions:** 1) Each organ has its own program for the switch from carbohydrate to fat metabolism. 2) The consequences of these changes on the development of organ dysfunction merit further investigation, as this may lead to novel directed therapeutics.

**References**

(1) Rudiger A. et al. Clin Sci 2013; 124:391-401

**Grant acknowledgement**

ESICM Basic Science Award, Intensive Care Foundation (UK) Young Investigator Award, NIHR

**Table 31:** (abstract A61).

	Sham	Survivors	Non-survivors
Soleus			
Phospho PDH	1.4±0.32	2.85±0.42	5.79±0.42^a,b^
PDH	0.99±0.11	0.78±0.31	0,24±0.03^a,b^
Heart			
Phospho ACC	0.37±0.01	1.37±0.012^a^	1.51±0.012^a^
Phospho AMPK	0.25±0.001	0.32±0.005	0.37±0.004

^a^ p< 0.05 vs control, ^b^ p< 0.05 vs survivors

### A62 Estimation of energy requirements using a standard ventilator

#### R. Meierhans, R. Schuepbach

##### University Hospital Zurich, University Zurich, Surgical Intensive Care Unit, Zurich, Switzerland

###### **Correspondence:** R. Schuepbach – University Hospital Zurich, University Zurich, Surgical Intensive Care Unit, Zurich, Switzerland

**Introduction:** Albeit practical implementation remains a concern, indirect calorimetry (IC) is the first choice technique for estimating energy requirements in critically ill patients.

**Methods:** In our adult mainly surgical intensive care unit (ICU) we retrospectively assessed in 25 patients the utility and practical aspects of estimating the daily energy requirements. We determined energy requirements by IC (Quark, COSMED) and compared the estimates with those calculated by measuring VCO_2_ alone using a ventilator (Evita XL; Draeger) or using formulas based on body mass of body mass index.

**Results:** Our study population was found to have an average body mass of 69.7 kg and according to IC and energy requirement of 1987 kcal/day on average. No correlation (R^2^ = 6E-05) was found when estimates of IC were compared to estimates based on body mass alone. However using an adjusted mass (based on hight and an assumed body mass index of 23) correlation improved (R^2^ = 0.3358). VCO_2_ based estimation of energy requirement only weakly correlated with IC (R^2^ = 0.414). Weak correlation is explained by assuming a fixed respiratory coefficient for ventilator based energy estimates as well as by imperfect correlation of VCO_2_ estimates reported by calorimeter and ventilator (R^2^ = 0.6189)

**Conclusions:** Body parameters such as hight or mass aren't suited to predict the patient's energy needs. Albeit promising and simple, estimation of energy requirement based on ventilator derived VCO_2_ also fails precision. Whether technical improvements on how ventilators calculate VCO2 can render them more suited in daily praxis will have to be determined in future studies.

### A63 Nutritional adequacy of ECMO patients

#### I. De Brito-Ashurst

##### Royal Brompton and Harefield NHS Foundation Trust, London, United Kingdom

**Introduction:** Extracorporeal membrane oxygenation (ECMO) is an advanced treatment to support the critically ill patients with cardiac and/or with severe respiratory failure. It is suggested that ECMO patients are at risk of splanchnic ischemia and thus, enteral feeding is poorly tolerated needing nasojejunal feeding and is potentially unsafe. This study aims to investigate the nutritional adequacy of this patient group.

**Objectives:** To establish feeding tolerance, caloric and protein intake during the initial 5 days of feeding.

**Methods:** A retrospective review of all the patients that received ECMO between January/2000 and December/2014 was conducted in a tertiary critical care unit/ECMO referral centre. Patients were all fed as per unit feeding protocol that recommends early enteral feeding instead of parenteral or delayed enteral nutrition.

**Results:** Data were reviewed for 80 patients for the initial 5 feeding days and is reported as mean and (SD). The average duration of ECMO was 12.7 (9.9) days. Sixty-five patients received venovenous ECMO for respiratory failure whilst the remainder received venoarterial ECMO for cardiac failure. Patients age, Body mass index (BMI) and sofa scores were 44.1 (15.7), 29.1 (8.5) and 15.7 (2.8) respectively. Enteral feeding started at 14.2 (5.8) hrs and reached 83 % target within 72 hrs. Overall, patients had good tolerance to the feed with a mean gastric residual volume (GRV) of 176.6 ml/24 hrs (167.75) during the 5 days. Feeding intolerance, defined as GRV >250 ml, were observed every day with 7, 13, 15, 10 and 8 episodes on days 1, 2, 3, 4 and 5 accordingly. Diarrhoea was observed on 16 % of the patients and mainly on days 3 (15/80), 4 (16/80) and 5 (17/80). Forty-two patients needed prokinectics (metoclopramide) and eight of those were also on erythromycin. Eight patients were on parenteral nutrition to supplement inadequate enteral feeding. Overall patients were receiving >80 % by day 3 onwards. All patients were on nasogastric feeding and nasojejunal feeding was not necessary.

**Conclusion:** Enteral nutrition is well tolerated by patients receiving ECMO, whether in venovenous or venoarterial mode. No serious adverse events were attributable to enteral nutrition in these patients.

## RESPIRATORY INFECTIONS

### A64 Incidence of ventilator associated pneumonia when pantoprazole or ranitidine is used for stress ulcer prophylaxis in critically ill adult patients: a pilot study

#### F. Zand^1^, G. Sabetian^2^, R. Nikandish^3^, F. Hagar^3^, M. Masjedi^1^, B. Maghsudi^1^, A. Vazin^3^, M. Ghorbani^1^, E. Asadpour^1^

##### ^1^Anesthesiology and Critical Care Research Center, Shiraz University of Medical Sciences, Shiraz, Islamic Republic of Iran; ^2^Shiraz University of Medical Sciences, Trauma Research Center, Shiraz, Islamic Republic of Iran; ^3^Shiraz University of Medical Sciences, Shiraz, Islamic Republic of Iran

###### **Correspondence:** G. Sabetian – Shiraz University of Medical Sciences, Trauma Research Center, Shiraz, Islamic Republic of Iran

**Introduction:** Nowadays, hospital acquired infections are the most widespread phenomena among the critically ill patients. Prophylaxis of gastric stress ulcer with acid-suppressive therapy increases the risk of ventilator associated pneumonia (VAP) in critically ill patients(1). While, two meta- analyses comparing PPI and H2RA in terms of prophylactic effectiveness for gastric stress ulcer in critical patients have showed no apparent differences in both groups as regard to upper gastric ulcer prophylaxis, pneumonia and mortality in patients admitted to ICU (2,3).

**Objectives:** Prophylaxis against stress ulcer in mechanically ventilated patients has been considered as a culprit in the development of ventilator associated pneumonia (VAP) in retrospective studies. To test this hypothesis in a mixed medical-surgical adult ICU, we designed a randomized clinical pilot study to determine the necessary sample size for comparing the effect of intravenous ranitidine and pentoprazole on the incidence of VAP.

**Methods:** Patients with at least 48 hours of expected mechanical ventilation were allocated randomly to receive either 50 mg ranitidine (R) every 8 hours or 40 mg pentoprazole (P) every 12 hours intravenously from admission till 48 hours after extubation. VAP diagnosis was according to Clinical Pneumonia Infection Score and positive culture of endotracheal tube aspiration.

**Results:** After recruiting 86 patients during 15 months period and a preliminary analysis, the study was terminated due to very low difference between VAP incidence in R (32.6 %) and P groups (27.9 %; P value 0.63, Odds ratio; 1.24, confidence interval; 0.49-3.13). No statistically significant difference was observed in terms of gastro-intestinal bleeding, ICU and hospital length of stay and mortality between the two groups.

**Conclusions:** VAP incidence is hardly related to the type of stress ulcer prophylaxis agent in our ICU setting with high rate of VAP and low utilization of VAP prophylaxis bundle. To find any such effect a huge number of patients are needed to be recruited in a large randomized clinical trial

**References**

1. Prescott HC, O´Brien JM. Prevention of ventilator-associated pneumonia in adults. F1000 medicine reports. 2010

2. Lin PC, Chang CH, Hsu PI, Tseng PL, Huang YB. The efficacy and safety of proton pump inhibitors vs histamine-2 receptor antagonists for stress ulcer bleeding prophylaxis among critical care patients: a meta-analysis. Critical care medicine. 2010;38(4):1197-205

3. Alhazzani W, Alenezi F, Jaeschke RZ, Moayyedi P, Cook DJ. Proton pump inhibitors versus histamine 2 receptor antagonists for stress ulcer prophylaxis in critically ill patients: a systematic review and meta-analysis. Critical care medicine. 2013;41(3):693-705.

**Grant acknowledgement**

This study was funded by a grant number 4227 from Vice-Chancellery of Research and Technology in Shiraz University of Medical Sciences, Shiraz, Iran.

### A65 Coinfection associated mortality in pneumonia-induced acute respiratory distress syndrome (ARDS)

#### K.-C. Kao^1^, L.-C. Chiu^2^, C.-Y. Hung^2^, C.-H. Chang^2^, S.-H. Li^2^, H.-C. Hu^2^

##### ^1^Chang Gung Memorial Hospital, Thoacic Medicine, Kwei-Shan, Taoyuan, Taiwan, Province of China; ^2^Chang Gung Memorial Hospital, Taoyuan, Taiwan, Province of China

###### **Correspondence:** K.-C. Kao – Chang Gung Memorial Hospital, Thoacic Medicine, Kwei-Shan, Taoyuan, Taiwan, Province of China

**Rationale:** Pneumonia is the leading risk factor of acute respiratory distress syndrome (ARDS). For the critically ill patients with pneumonia, coinfection had higher mortality and longer length of stay in the intensive care unit (ICU). Little is known about the impact of coinfection on the outcomes of pneumonia-induced ARDS patients.

**Objectives:** To evaluate the role of coinfection from bronchoalveolar lavage (BAL) examination on the outcomes of pneumonia-induced ARDS.

**Methods:** We performed a prospective observational study in adult ICUs at the Chang Gung Memorial Hospital from October 2012 to May 2015. Patients were included if they met the Berlin definition of ARDS. The BAL indication were pneumonia-induced ARDS was suspected clinically and no definite microbial was indentified from tracheal aspirate. The BAL specimen was sent for comprehensive microbiological study including bacteria, fungi and virus. Demographics and baseline clinical characteristics were collected on enrollment. The final pathogen identified results and clinical outcomes were analyzed.

**Results:** Totally 19936 receiving invasive mechanical ventilation patients admitted to ICUs with PaO2/FiO2 < 300 mmHg screened, 902 (4.5 %) patients fulfilled the Berlin definition of ARDS. Of these ARDS patients, 205 (22.7 %) patients who were pneumonia-induced ARDS with BAL for pathogen survey were included for analysis. One hundred and forty two (55.7 %) patients were identified with microbiological pathogen. According the identified virus pathogen, these 142 patients were divided as only virus identified group (n = 41, 28.9 %), no virus identified group (n = 60, 42.2 %) and coinfection with other pathogen group (n = 41, 28.9 %). The distribution of ARDS severity were no significant difference between these 3 group patients (p = 0.43). The coinfection group had significantly higher hospital mortality rate than only virus group and no virus group (80.5 % vs 53.7 % and 63.3 %; p = 0.03).

**Conclusions:** The coinfection with virus and other pathogen from BAL was associated with increased mortality in pneumonia-induced ARDS patients.

### A66 Kallistatin level as a novel prognostic marker for community acquired pneumonia (CAP) in critically ill patients

#### S. El Maraghi^1^, M. Ali^1^, D. Rageb^2^, M. Helmy^1^

##### ^1^Faculty of Medicine - Beni Suef University, Critical Care Department, Cairo, Egypt; ^2^Faculty of Medicine - Cairo University, Critical Care Department, Cairo, Egypt

###### **Correspondence:** S. El Maraghi – Faculty of Medicine - Beni Suef University, Critical Care Department, Cairo, Egypt

**Introduction:** CAP is a potentially life threatening disorder despite the advent of potent antibiotics & commonly causes acute respiratory failure with high mortality^1^.Assessment of disease severity & prediction of outcome is essential for appropriate allocation of health care resources & for optimized treatment decisions^2^. Kallistatin, an endogenous tissue kallikrein inhibitor; which protects against inflammation, fibrosis & oxidative stress may be involved in CAP pathogenesis through anti-inflammatory effect^3^.

**Objectives:** To determine whether kallistatin levels have a prognostic value in severe CAP & to correlate it with other biomarkers as CRP,APACHE II ,SOFA, CURB-65 scores & Pneumonia severity index (PSI).

**Methods:** Plasma samples and clinical data were prospectively collected from 30 patients admitted to the Critical Care Department with severe CAP according to IDSA/ATS Criteria^**4**^. Serum Kallistatin levels were collected on days 1 and 4 of ICU admission. It was correlated with APACHE II, SOFA, CURB-65 scores & PSI on admission also with P_O2_/Fi_O2_ ratio and CRP on days 1 and 4.

**Results:** Lower kallistatin levels on days 1 & 4 showed a strong trend toward increased mortality with a p-value of 0.043 & 0.04 respectively. Its level was negatively correlated with APACHE II,SOFA,CURB-65 scores & PSI and with a p-value of 0.036,0.001, 0.102 & 0.001 respectively while it showed a positive linear correlation with Po_2_/Fio_2_ ratio on days 1 & 4 with a P-value of 0.001 & 0.005 respectively. It also showed a negative correlation with CRP on days 1 and 4 with a p-value of 0.001. Its level on days 1 & 4 of ICU admission were significantly decreased in patients who developed septic shock with a p-value of 0.044 & 0.043 respectively and who had ARDS requiring mechanical ventilation with a P-value of 0.001 & 0.005 respectively. Kallistatin cut-off value to predict mortality determined by ROC curve was 6.65ug/ ml on day1 with sensitivity 90 % and specificity 80 % & a p-value 0.022 while was 8.6ug/ml on day 4 with sensitivity 85 % and specificity 77 % & a p-value 0.035.

**Conclusions:** kallistatin may serve as a promising novel marker for prognosis of severe CAP & may be involved in its pathogenesis

**References**

1. Restrepo MI, Mortensen EM, Velez JA, et al: A comparative study of community-acquired pneumonia patients admitted to the ward and the ICU. Chest 2008, 133:610-617.

2. *Lim WS, van der Eerden MM, Laing R, Boersma et al*: Defining community acquired pneumonia severity on presentation to hospital: an international derivation and validation study. Thorax 2003, 58:377-382.

3. Wei-Chieh Lin, Shiou-Ling Lu, Chiou-Feng Lin, et al: Plasma kallistatin levels in patients with severe community-acquired pneumonia.Critical Care 2013, 17:R27.

4. Mandell LA, Wunderink RG, Anzueto A, et al. Infectious Diseases Society of America/American Thoracic Society consensus guidelines on the management of community-acquired pneumonia in adults. Clin Infect Dis 2007;44(Suppl 2): S27-72.

### A67 Clinical impact of delayed diagnosis of influenza A (H1N1)pdm09 in critically ill patients

#### J. Marin-Corral^1^, C. Vilà^1^, J.R. Masclans^1,2,3^, A. Vàzquez^1^, I. Martín-Loeches^4^, E. Díaz^5^, J.C. Yébenes^6^, A. Rodriguez^7^, F. Álvarez-Lerma^1,8^, H1N1 SEMICYUC/GETGAG Working Group

##### ^1^Hospital Parc de Salut Mar - GREPAC, IMIM, Critical Care Department, Barcelona, Spain; ^2^Pompeu Fabra University (UPF) - CEXS, Barcelona, Spain; ^3^CIBERES. Instituto de Salud Carlos III, Madrid, Spain; ^4^Multidisciplinary Intensive Care Research Organization (MICRO). St James's; University Hospital. Trinity Center for Health Sciences, Department of Anesthesia and Critical Care, Dublin, Ireland; ^5^Hospital Parc Tauli, Critical Care Department, Sabadell, Spain; ^6^Hospital de Mataró, Critical Care Department, Mataró, Spain; ^7^University Hospital Joan XXIII - IISPV-URV, Critical Care Department, Tarragona, Spain; ^8^Universitat Autònoma de Barcelona, Barcelona, Spain

###### **Correspondence:** J. Marin-Corral – Hospital Parc de Salut Mar - GREPAC, IMIM, Critical Care Department, Barcelona, Spain

**Objectives:** To assess the clinical and developmental implications of delayed diagnosis of community-acquired influenza A (H1N1) pdm09 infection in critically ill patients admitted to the ICU.

**Methods:** Prospective, observational and multicenter study in 148 Spanish ICU. Data were obtained from GETGAG / SEMICYUC (2009-2015). All patients infected by influenza A (H1N1) confirmed by RT-PCR in the first week of hospital stay were included (viral community-acquired pneumonia). Patients were classified according to the moment of flu diagnosis: early (in the first two days of hospitalization) and late (between the 3rd and 7th day of admission). Demographic, temporary variables, comorbidities, severity at ICU admission, treatment and mortality were evaluated intra-ICU. Logistic regression was used to identify related factors to late diagnosis and Cox regression to determine whether the late diagnosis was an independently variable associated with mortality. The results are presented as odds ratios (OR) and 95 % confidence intervals (CI). We considered p < 0.05 to be significant.

**Results:** 2059 ICU patients diagnosed in the first 7 days of hospital stay were evaluated. 1314 (63.8 %) were early diagnosed and 745 (36.2 %) were late diagnosed. Independent variables associated to late diagnosis were: days of hospital stay until ICU admission (OR 1.254, 95 % CI 1.169 to 1.345; p < 0.001), mechanical ventilation (OR 1.690, 95 % CI 1.259 to 2.268; p < 0.001) and the need of renal replacement (OR 1.509, 95 % CI 1.071 to 2.125; p = 0.019). Clinical presentation as viral pneumonia was a protective variable related to the delay (OR 0.654, 95 % CI 0.495 to 0.881; p = 0.005). Patients with late diagnosis presented higher ICU [8(4-17) vs 10(5-20), p = 0.000] and hospital days of stay [14(8-25) vs 18(10-30), p = 0.000] as well as higher intra-ICU mortality (20.5 % vs 32.6 %, p = 0.000). The diagnosis delay was an independently variable associated with mortality in the multivariate analysis (OR 1.364, 95 % CI 1.029 to 1.808).

**Conclusions:** Influenza A (H1N1)pdm09 diagnosis delay in critically ill patients admitted to the ICU is associated with delayed ICU admission, increased respiratory failure or acute renal failure and increased mortality. Delayed diagnosis is an independently variable associated with mortality.

### A68 Influenza A in severe acute respiratory infection. Timing for ICU admission is important

#### N. Varga, A. Cortina-Gutiérrez, L. Dono, M. Martínez-Martínez, C. Maldonado, E. Papiol, M. Pérez-Carrasco, R. Ferrer

##### Hospital Universitari Vall d'Hebron, Critical Care, Barcelona, Spain

###### **Correspondence:** N. Varga – Hospital Universitari Vall d'Hebron, Critical Care, Barcelona, Spain

**Introduction:** Influenza viruses is one of the main cause of severe acute respiratory infection (SARI). To know its clinical features and prognosis factors are essential for management and outcome.

**Objectives:** To determine the incidence and mortality of patients with SARI and influenza A infection admitted to the ICU; and to identify the most common symptoms and comorbidities among patients affected by SARI and influenza A infection.

**Methods:** A prospective observational study was performed including patients with SARI admitted in ICU of a tertiary care Hospital from november 2014 to march 2015. Influenza infection was diagnosed by RT-PCR. Patients´ demographic, clinical and radiologic features and outcomes were recorded. Pearson ´s chi-square, Fisher, Mann Whitney U tests and logistic regression have been used for statistical analysis. Data are expressed as frequency (percentage) or median (25th-75th interquartile range).

**Results:** During study period 425 patients were admitted to ICU. Seventy-five (17.6 %) were included as SARI (57.3 % male; median age 57(47-67) years old; APACHE II 21(13-27) and SOFA 8(5-10); ICU LOS 8(4-23) days; days from symptom onset to hospital admission 4(1-6)). Main co-morbidities were lung diseases in 44(33 %) patients and cardiovascular diseases in 19(25.3 %) patients. Seventeen (22.7 %) patients presented Influenza A infection, only one did not presented viral pneumonia. Cough, shortness of breath and muscle pain were the most common symptoms in influenza A patients (87.5 %, 68.8 % and 43.8 % respectively). Influenza-like illness was more frequent in influenza A patients (52.9 % vs. 32.8 %; p = 0.13), furthermore most patients with influenza A infection were diagnosed during influenza epidemic period (88.2 % vs. 37.9 %; p < 0.001); all patients with influenza A infection were treated with oseltamivir.

High-flow oxygen therapy was required in 53(70.7 %) SARI patients, 11(14.7 %) non-invasive mechanical ventilation, 46(61.3 %) invasive mechanical ventilation, 30(40 %) inotropic drugs and 11(14.7 %) renal replacement therapy. Influenza A patients presented a similar distribution.

Overall SARI mortality was 20 %, only one with influenza A infection (5.6 % vs. 26.7 %; p = 0.1). APACHE II (25(19-33) vs. 19(11-26); p = 0.03) and SOFA score (12(8-14) vs. 8(5-10); p = 0.003) showed to be a good predictor of mortality at ICU admission. Also, delayed admission to ICU (4(1-16) vs. 1(0-2); p = 0.01) and renal replacement therapy (40 % vs. 6.9 %; p = 0.04) were associated with increased mortality.

Previous renal diseases (OR 10.28 CI 95 % (2.25-46.96); p = 0.003) and oncology diseases (OR 6.8 CI 95 % (1.59-29.11); p = 0.01) were independently associated with mortality.

**Conclusions:** A nonsignificant decrease in mortality was observed in patients with influenza A infection. Early ICU admission could improve SARI prognosis.

### A69 Do routine clinical markers predict discharge in patients with H1N1 influenza?

#### K. Nweze^1,2^, B. Morton^2^, I. Welters^2^

##### ^1^University of Liverpool, Institute of Infection and Global Health, Liverpool, United Kingdom; ^2^Royal Liverpool University Hospital, Intensive Care Unit, Liverpool, United Kingdom

###### **Correspondence:** K. Nweze – University of Liverpool, Institute of Infection and Global Health, Liverpool, United Kingdom

**Introduction:** H1N1 influenza can cause both self-limiting and life-threatening illnesses. Triaging patients who require hospital admission and those who can be safely discharged can be difficult in a pandemic situation. Existing triage tools focus on prediction of hospital admission in H1N1. We assessed the ability of routine clinical markers to predict early discharge in patients with confirmed H1N1 influenza.

**Objectives:** 1) Assess the ability of routine clinical and laboratory variables to predict patients who could safely be discharged home within 24 hours.

2) Compare the predictive value of clinical variables to triaging tools (STSS and SOFA) and the qSOFA criteria.

**Methods:** We reviewed an existing database of patients who presented to the Royal Liverpool University hospital from 2010 - 2011. Inclusion criteria were: patients with H1N1 confirmed by reverse transcriptase polymerase chain reaction who were ≥18 years of age. Exclusion criteria were: unconfirmed cases, patients not seen in the hospital and with missing case notes. Differences in clinical parameters between patients discharged within 24 hours of medical assessment and those admitted to hospital were analysed. A chest X-ray scoring system was also employed to assess the ability of radiographic findings to predict likelihood of discharge.

**Results:** Eighty-six patients were eligible for the study. 17 patients were discharged early and 69 patients were admitted to hospital. P/F ratio and CRP predicted discharge with area under receiver operating characteristic (ROC) curves of 0.788 (CI 0.681-0.894) and 0.763 (CI 0.6377-0.889) respectively, which was higher than triage and bedside tools. The chest radiograph scoring tool did not predict patient discharge (p = 0.191-0.999), but demonstrated very good inter-rater reliability (Cohen's kappa statistic >0.8).

**Conclusions:** P/F ratio and CRP predicted discharge better other clinical parameters. Both were superior to H1N1 specific triage tools described in the literature. P/F ratio is a simple and effective method to determine oxygen exchange. We recommend this tool in the assessment of patients during influenza pandemic to guide management decisions and future work would involve validation in prospective cohorts.

**References**

1. Singanayagam et al (2011) ‘Factors associated with severe illness in pandemic 2009 influenza a (H1N1) infection: implications for triage in primary and secondary care’, J Infect, 63(4), 243-51.

2. Singer et al. (2016) ‘The third international consensus definitions for sepsis and septic shock (sepsis-3)’, JAMA, 315(8), 801-810.

3. Taylor, E. (2015) ‘A chest radiograph scoring system in patients with severe acute respiratory infection: a validation study’, *BMC Med Imaging,* 15, 61.

### A70 Relationship between digestive tract colonization and subsequent ventilator-associated pneumonia related to ESBL-producing enterobacteriaceae

#### M. Houard, B. Voisin, G. Ledoux, S. Six, E. Jaillette, S. Nseir

##### Lille University Hospital, ICU, Lille, France

###### **Correspondence:** M. Houard – Lille University Hospital, ICU, Lille, France

**Introduction:** Ventilator-associated pneumonia (VAP) is the most common ICU-acquired infection. Recently, the incidence of extended-spectrum beta-lactamase producing *Enterobacteriaceae* (ESBLE) has substantially increased in critically ill patients. Identifying patients at risk for VAP related to ESBLE could be helpful to improve the rate of appropriate initial antibiotic treatment, and reduce unnecessary exposure to carbapenems.

**Objectives:** The primary objective was to identify risk factors for VAP related to ESBLE. Secondary objective was to determine the impact of ESBLE on outcome of VAP patients.

**Methods:** This retrospective study was conducted in a single mixed ICU, during a 4-year period. All patients with confirmed VAP were included. VAP was defined using clinical, radiologic and quantitative microbiological data. VAP first episodes were prospectively identified using the continuous surveillance data. Exposure to different risk factors was taken into account until the diagnosis of ESBLE VAP or until ICU discharge, in patients with ESBLE VAP and VAP related to other bacteria, respectively. In all patients, routine screening for ESBL (rectal swab) was performed at ICU admission and once a week.

Patients with ESBLE VAP were compared with those with VAP related to other bacteria using univariate analysis. All significant factors were included in the multivariate logistic regression model.

**Results:** Among the 410 patients with VAP, 43 (10.5 %) had ESBLE VAP. 76 (18.5 %) patients had polymicrobial VAP, and 189 (46 %) had VAP related to multidrug resistant bacteria.

The following factors were significantly associated with higher rates of ESBLE VAP by univariate analysis: female gender, medical admission, ARDS, shock, or infection at admission, antibiotic treatment during the 30 days preceding ICU admission, large-spectrum antibiotic treatment during ICU stay, and digestive tract colonization related to ESBLE. Acute exacerbation of COPD at ICU admission was significantly associated with lower rate of ESBLE VAP by univariate analysis.

Multivariate analysis identified prior ESBLE colonization of the digestive tract as the only independent risk factor for ESBLE VAP (OR [95 % CI] = 23 [10-55], p < 0.001). Whilst the positive predictive value of ESBL digestive colonization (43.6 %) was low, its negative predictive value was excellent (97.3 %) in predicting ESBLE VAP.

Duration of mechanical ventilation (median [IQR], 28 [18, 42] vs 23 [15, 42] d, p = 0.4), length of ICU stay 31 [19, 53] vs 29 [18, 46] d, p = 0.6), and mortality rates (55.8 % vs 50 %, p = 0.48) were similar in ESBLE VAP, compared with VAP related to other bacteria.

**Conclusions:** Digestive tract colonization related to ESBLE is independently associated with ESBLE VAP. Its excellent negative predictive value suggests that patients without ESBLE colonization should not receive carbapenems as part of their initial empirical antibiotic treatment to cover ESBLE.

### A71 Severe acute respiratory infections in a Tunisian ICU

#### S. Romdhani^1^, R. Bouneb^1^, D. Loghmari^1^, N. Ben Aicha^1^, J. Ayachi^1^, K. Meddeb^1^, I. Chouchène^1^, A. Khedher^1^, M. Boussarsar^1,2^

##### ^1^Farhat Hached University Hospital, Medical Intensive Care Unit, Sousse, Tunisia; ^2^Ibn Al Jazzar Faculty of Medicine, University of Sousse, Research Laboratory N° LR14ES05, Interactions of the Cardiopulmonary System, Sousse, Tunisia

###### **Correspondence:** S. Romdhani – Farhat Hached University Hospital, Medical Intensive Care Unit, Sousse, Tunisia

**Introduction:** Severe acute respiratory syndrome (SARS) and H1N1 influenza infection (1) have activated an interest in the surveillance of patients with severe acute respiratory infections (SARI).

**Objectives:** To investigate the local epidemiology, patterns of infections, severity and outcome in patients admitted to the intensive care unit (ICU) as a result of severe acute respiratory infections (SARI).

**Methods:** This is a prospective observational study. All patients admitted to the ICU, from October 1st, 2015 to march 31st, 2016 were screened. Were studied, demographic characteristics, underlying conditions, clinical presentation, therapeutic intervention and outcome. SARI was defined as an acute respiratory illness of recent onset (within 7 days) that includes fever (≥38 °C), cough, and dyspnea requiring overnight hospitalization (2).

**Results:** 33 patients were screened over the study period. Median (IQR) age was 68(18-87) years. 63.6 % were male. Median (IQR) SAPS II, 26(13-64) and SOFA score, 5(2-9). 24(77.2 %) had underlying diseases. The symptoms' onset occurred at a median (IQR) of 4(3-7) days before admission to the ICU. Chest X-ray displayed alveolar consolidation in 19(86.4 %).

On admission to the ICU, Gram-positive and Gram-negative bacteria were found in 5(15 %) and 8(24 %) of SARI patients but rarely atypical bacteria in 1(3 %). Viruses were present in 6(18 %) of the patients *(influenza A-H1N1, 3; A-H3N2, 3).*

22(66.6 %) required invasive mechanical ventilation, 18(54.5 %) vasopressors, 10(45.4 %) corticosteroids, 21(63.6 %) antimicrobial therapy and none was treated with antiviral agents.

Organ failure occurred in 26 (80 %) patients, (respiratory, 14 (53,9 %) ; hemodynamic, 9(34,6 %) and renal, 3(11,5 %)). Median (IQR) length of stay was 6(1-30) days and duration of mechanical ventilation was 4(1-20) days. ICU mortality rates in patients with SARI were, overall, 18(54.4 %) ; 7(45 %) in severe and prolonged hypoxemia and 11 (54 %) with refractory shock.

Univariate analysis identified *respiratory chronic failure* and greater *severity scores* at ICU admission to be associated with an increased risk of ICU death.

**Conclusions:** Since the pandemic influenza A (H1N1) in 2009, the proportion of viral infections had decreased. Whatever, admission to the ICU for SARI remains associated with high morbidity and mortality rates

**References**

1. Rello J, and al. H1N1 SEMICYUC Working Group (2009) Intensive care adult patients with severe respiratory failure caused by Influenza A (H1N1) in Spain. Crit Care 13:R148

2. Sakr Y, Ferrer R, Reinhart K, Beale R, Rhodes A, Moreno R, et al. The Intensive Care Global Study on Severe Acute Respiratory Infection (IC-GLOSSARI): a multicenter, multinational, 14-day inception cohort study. Intensive care medicine. 2016;42(5):817-28.

### A72 Concurrent epidemics of influenza and aspergillosis in Taiwan, 2016

#### K.S. Chan^1^, W.L. Yu^1,2^

##### ^1^Chi Mei Medical Center, Tainan, Taiwan, Province of China; ^2^Taipei Medical University, Taipei, Taiwan, Province of China

###### **Correspondence:** K.S. Chan – Chi Mei Medical Center, Tainan, Taiwan, Province of China

**Introduction:** The epidemic flu in 2016, predominantly influenza A (H1N1), is causing chaotic situations to the health care facilities in Taiwan, including emergency department overcrowding, difficult for patients to find beds in emergency rooms or in ICUs, and shortage of respiratory ventilators as well as extracorporeal membrane oxygenation (ECMO) machines. Death toll continues to rise and is record-breaking. The reasons why so many patients suffered from such a severe influenza that required intensive care is unclear. Some studies have emphasized the importance of *Aspergillus* infection during severe influenza attack.

**Objectives:** To show if there is any correlation between the activity of aspergillosis and influenza infection

**Methods:** Confirmed influenza case was defined at least one positive assay for testing influenza included influenza A and B rapid antigens, real time polymerase chain reaction (PCR), viral isolation and identification for specimens of nasopharyngeal swab and/or lower respiratory tract aspirates. The detection of *Aspergillus* galactomannan (GM) antigen by Platelia *Aspergillus* Ag assay in human serum and bronchoalveolar lavage fluid was used as an aid in the assessment of patients with suspected aspergillosis. A GM index ≥ 0.5 was defined positive for *Aspergillus* antigenemia. Decision of testing *Aspergillus* antigen was made by attending physicians, mostly for patients with worsening pneumonia under oseltamivir and/or routine antibiotic therapy.

**Results:** A total of 1,640 hospitalized medical ICU patients were identified during the observed 7-month period, including 32 (1.95 %) patients had a diagnosis of severe influenza and 24 (1.46 %) patients were positive for *Aspergillus* GM antigen test. Meanwhile, 12 (37.5 %) patients of severe influenza with worsening pneumonia had *Aspergillus* antigenemia. However, only 13 influenza patients were tested for *Aspergillus* GM antigen. The physician-dependent patient selection bias resulted in 92.3 % accuracy. The incidence of patients with influenza and/or positive *Aspergillus* antigenemia in medical ICU patients significantly increased in February 2016 in comparison to previous 6 months in the hospital.

**Conclusions:** Medical imaging and serum galactomannan antigen currently constitute the basis of the screening approach for invasive pulmonary aspergillosis. The presence of *Aspergillus* antigenemia might not represent true invasive *Aspergillus* infection, but a prompt diagnostic workup should be encouraged as most of these patients suffering life-threatening ARDS. The clinical impact of invasive aspergillosis on severe influenza may be more important than previous thought. In conclusion, our study revealed a possible link between invasive aspergillosis and severe influenza patients. Recognition of this potential might help physicians to initiate a prompt diagnostic workup incorporated into strategies of preparedness for the epidemic crisis.

### A73 Prognostic value of nosocomial influenza A (H1N1)pdm09 infection

#### J. Marin-Corral^1^, C. Vilà^1^, J.R. Masclans^1,2,3^, J. Nolla^2,4^, L. Vidaur^5^, J. Bonastre^6^, B. Suberbiola^7^, J.E. Guerrero^8^, A. Rodriguez^3,9^, H1N1 SEMICYUC/GETGAG working group

##### ^1^Hospital Parc de Salut Mar - GREPAC, IMIM, Critical Care Department, Barcelona, Spain; ^2^Pompeu Fabra University (UPF) - CEXS, Barcelona, Spain; ^3^CIBERES. Instituto de Salud Carlos III, Madrid, Spain; ^4^Hospital Parc de Salut Mar - GREPAC, IMIM, Barcelona, Spain; ^5^Hospital de Donosti, Critical care Department, San Sebastian, Spain; ^6^Hospital la Fe, Critical care Department, Valencia, Spain; ^7^Hospital de Valdecillas, Critical care Department, Santander, Spain; ^8^Hospital Gregorio Marañon, Critical care Department, Madrid, Spain; ^9^University Joan XXIII Hospital - IISPV - URV, Critical care Department, Tarragona, Spain

###### **Correspondence:** J. Marin-Corral – Hospital Parc de Salut Mar - GREPAC, IMIM, Critical Care Department, Barcelona, Spain

**Objectives:** To assess the prognostic implications of hospital acquisition of influenza A (H1N1) pdm09 virus in a population of critically ill patients admitted to the ICU.

**Methods:** prospective, observational and multi-center study in 148 Spanish ICU. Data were obtained from the GETGAG / SEMICYUC (2009-2015) registry. All patients infected by influenza A (H1N1) confirmed by RT-PCR were included. Patients were classified into two groups depending on the day of diagnosis of flu: community-acquired (in the first 48 h of hospital admission) and nosocomial infection (after the 7th day of admission and without treatment with oseltamivir). Demographic and temporary variables, comorbidities, severity on admission, treatment and mortality were evaluated. Differences between groups were assessed using chi-square for categorical variables and Student's t-test or Mann-Whitney test for continuous variables. Significant variables in the univariate analysis were included in a multivariate model (logistic regression). We considered p < 0.05 to be significant.

**Results:** 2421 patients with influenza A (H1N1) were included of which 2035 (84 %) were evaluables. 1103 (54.2 %) were classified as influenza A community-acquired infection and 224 (11.0 %) as nosocomial infection. 708 (34.8 %) could'nt be classified in either groups of patients. Patients with nosocomial pneumonia presented: older age (53.47 ± 15.15 vs 48.86 ± 15.33; p < 0.000), higher rates of severity on admission (APACHE II 17 (7) vs 15 (7); p = 0.005 and SOFA 7 (3) vs 5 (3); p = 0.004), more immunosuppression and hematological diseases (20.5 % vs 9.1 %, p = 0.000; 12.5 % vs 5.5 % p = 0.000), more requirements of invasive mechanical ventilatilation (82.7 % vs 66.1 %, p < 0.001) and more days of mechanical ventilation (12 [5-20] vs 8 [3-15], p = 0.000). ICU stay (8 (4-17) vs 12 (5-22), p = 0.001), hospital stay (14 (8-25) vs 20 (12-30), p = 0.000) and mortality (18.8 % vs 39.2 %, p = 0.000) also increased significantly in the group of nosocomial infection. Furthermore, the hospital acquisition of influenza A H1N1 (OR = 1.63, 95%IC 1.33-1.99, p = 0.000), the APACHEII (OR = 1.08, 95%IC 1.06-1.11, p = 0.000), the hematological diseases (OR = 3.19, 95%IC 1.77-5.73, p = 0.000) and the need of renal replacement (OR = 4.20, 95%IC 2.60-6.77, p = 0.000) and mechanical ventilation (OR = 4.34, 95%IC 2.62-7.20, p = 0.000) were independently variables associated with mortality in the multivariate analysis.

**Conclusions:** Patients with nosocomial influenza A (H1N1) pdm09 admitted in the ICU are more seriously ill requiring more resources consumption. The hospital acquisition of influenza A (H1N1) pdm09 in critically ill patients who need to be admitted in the ICU is associated with increased mortality.

### A74 Descriptive analysis of influenza affected patients admitted in ICU from 2010 to 2016

#### N. Ramon Coll, G. Jiménez Jiménez, S. Carvalho Brugger, J. Codina Calero, B. Balsera Garrido, M. García, M. Palomar Martínez, M. Vallverdú Vidal

##### Hospital Universitari Arnau de Vilanova, Lleida, Spain

###### **Correspondence:** S. Carvalho Brugger – Hospital Universitari Arnau de Vilanova, Lleida, Spain

**Introduction:** In 2009 was described the first pandemic of XXI century caused by influenza virus A (H1N1). This virus did not affect only patients in extreme ages of life and significant comorbidity, it also affected young and immunocompetent patients. The antigenic variations these viruses suffer, cause seasonal outbreaks of different extension.

**Objectives:** Describe the characteristics of patients diagnosed by influenza who required admission to an intensive care unit (ICU).

METHODOLOGY: Descriptive analysis including all patients diagnosed with influenza admitted from October 2010 to April 2016 in the ICU of a referral hospital for a population of 450,000 inhabitants. The diagnosis was obtained by PCR on nasal or pharyngeal swabs, sputum or tracheal aspirate. Epidemiological variables, risk factors, severity on admission, administered treatments, life support therapies and mortality were collected.

**Results:** During this period, 52 patients with influenza were admitted with an average of 8.67 per year (0-22). The mean age was 55.38 years (16-86) and 65.38 % were men. Mean APACHE II was 17.56 and mean SOFA was 6.15. Only 16 patients (30.76 %) had been vaccinated. Influenza A was identified in 94.23 % cases (78.85 % influenza A H1N1). Comorbidities presented by the patients were: COPD 14 (26.92 %), hematologic diseases 10 (19.23 %), diabetes mellitus 10 (19.23 %), obesity 9 (17.3 %) and pregnancy 4 (7.69 %). Radiological affectation was observed in 40 patients (76.92 %) at admission. 32 patients required vasoactive drugs, 7 required renal replacement and 4 patients were moved into the prone position (applied in our center since 2013). 94.23 % patients received oseltamivir for an average of 7.7 days (5.98 days on average between onset of symptoms and first dose). 94.23 % received empirical antibiotic therapy. There was respiratory coinfection in 18 cases (44.44 % *S. pneumoniae*). Ventilator-associated pneumonia was not observed in any patient. In 48 patients (92.3 %) was required mechanical ventilation (MV). Initially, 37 patients received non-invasive mechanical ventilation (NIMV) and 11 invasive mechanical ventilation (IMV). NIMV failure occurred in 21 patients (59.96 %) and 20 of them were connected to IMV. Only 4 patients did not require ventilatory support. The mean ICU stay was 12 days. A total of 11 patients (22.92 %) died during their hospital stay, all of them in the ICU. In this group, the mean APACHE II was 18.1 and mean SOFA 7.5, and the days between onset of symptoms and the administration of the first dose of oseltamivir were, on average, 8.72

**Conclusions:** Influenza A H1N1 was identified in most of the patients. The non-survivor subgroup presented a higher rate of immunosuppression, an increased severity at admission and a longer period of time between onset of symptoms and the administration of the first dose of oseltamivir.

### A75 IgG2 as an independent risk factor for mortality in patients with community-acquired pneumonia

#### M.C. de la Torre^1^, E. Vendrell^1^, E. Palomera^2^, E. Güell^1^, J.C. Yébenes^1^, M. Serra-Prat^2^, J.F. Bermejo-Martín^3^, J. Almirall^1^

##### ^1^Consorci Sanitari del Maresme, Intensive Care Unit, Mataró, Spain; ^2^Consorci Sanitari del Maresme, Investigation Unit, Mataró, Spain; ^3^Hospital Clínico Universitario de Valladolid, Unidad de Investigación Médica en Infección e Immunidad, Valladolid, Spain

###### **Correspondence:** M.C. de la Torre – Consorci Sanitari del Maresme, Intensive Care Unit, Mataró, Spain

**Introduction:** Mortality in patients with community-acquired pneumonia (CAP) remains high despite improvements in treatment.

**OBJECTIVE.** To determine immunoglobulin levels in patients with CAP and impact on disease severity and mortality

**Methods:** Observational study. Hospitalized patients with CAP were followed up 30-days. Levels of immunoglobulin G (IgG) and subclasses, immunoglobulin A (IgA) and immunoglobulin M (IgM) were measured in serum on the first 24 hours of CAP diagnosis.

**Results:** 362 patients with CAP — 172 ward-treated and 190 ICU-treated — were enrolled. ICU-treated patients had significantly lower values of IgG1, IgG2, IgG3 subclasses and IgA than ward-treated patients. 38 patients died before 30 days. Levels of IgG2 were significantly lower in non-survivors than survivors (p = .004) and a level of IgG2 < 301 mg/dL was associated with poorer survival according to both the bivariate (HR 4.47; p < .001) and multivariate (HR 3.48; p = .003) analyses.

**Conclusions:** Patients with CAP with IgG2 levels < 301 mg/dL had a poorer prognosis and a higher risk of death. Our study suggests the utility of IgG2 to predict the evolution of CAP and increase support measures or additional treatment.

### A76 VAP: incidence, risk factors and the antimicrobial resistance pattern in an Angolan ICU

#### E. Tomas^1^, A. Escoval^2^, F. Froe^2^, M.H. Vitoria Pereira^3^, N. Velez^4^, E. Viegas^4^, E. Filipe^1^

##### ^1^Clinica Sagrada Esperança, ICU, Luanda, Angola; ^2^Escola Nacional de Saude Publica/ UNL, Lisboa, Portugal; ^3^Clinica Sagrada Esperança, Infectiology, Luanda, Angola; ^4^Clinica Sagrada Esperança, Luanda, Angola

###### **Correspondence:** E. Tomas – Clinica Sagrada Esperança, ICU, Luanda, Angola

**Introduction:** Ventilator-Associated Pneumonia (VAP) is one of the most frequent nosocomial infections in ICU and results in increased mortality, prolonged hospital stay and greater healthcare costs.

**Objectives:** To analyze the frequency, risk factors and the antimicrobial resistance pattern of microbiological agents responsible for VAP in patients admitted in the ICU at Clínica Sagrada Esperança, in 2015.

**Methods:** A retrospective cohort of 99 patients mechanically ventilated for ≥48 h. Data was collected from ICU admission to 28 days or death. The VAP diagnosis was defined according to CDC but required microbiological confirmation. We analyzed the association between risk factors and the occurrence of VAP.

**Results:** The mechanical ventilation utilization ratio was 0.67. The incidence of VAP was 14.9 episodes per 1,000 days of mechanical ventilation. 69 % of cases of VAP were late onset VAP. The reintubation OR ( 95 % CI) 10,714 ( 2.320 to 49.490 ) was identified as independent risk factor for VAP.

VAP was not associated with attributable mortality in the ICU or 28 days mortality (8%vs.49 %; p = 0.013 )vs.(15%vs.51 %; p = 0.011), but was related to a threefold increase in duration of mechanical ventilation (29.8 ± 13.7vs.10.7 ± 6.5; p = 0.000) and increase in hospital stay (20.6 ± 13.7vs.7.0 ± 4.2; p = 0.000).

Gram-negative bacteria were the most common agents in our ICU.

**Conclusions:** To know and close monitoring of the incidence of VAP, microbiological agents associated and its antimicrobial resistance pattern is the key to the adoption of more specific standards ICU rules.

**References**

1- Alp, E., & Voss, A. (2006). Annals of Clinical Microbiology and Antimicrobials, 5 (1), 7.

2- Rosenthal, V. D., Maki, D. G., Mehta, Y., Leblebicioglu, H., Memish, Z. A., Al- Mousa, H. H., … & Apisarnthanarak, A. (2014). American journal of infection control,42(9), 942-956

**Fig. 29 (abstract A76):**
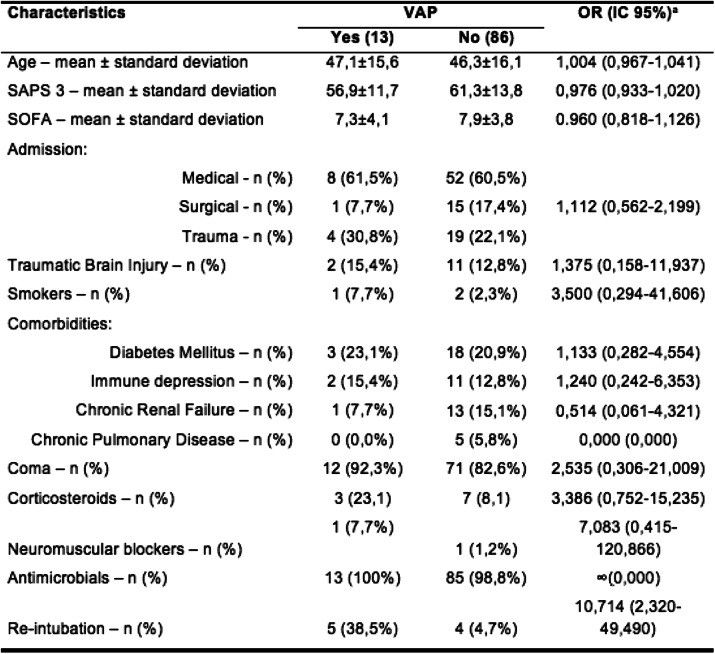
Potential risk factors for VAP

**Fig. 30 (abstract A76).**
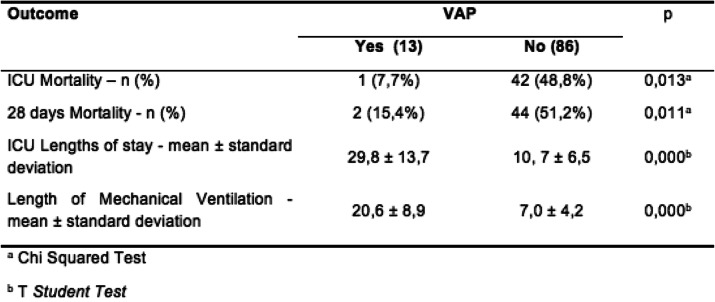
Outcome

### A77 Predictors of positive microbiology using a vap electronic triggering system

#### C. Groves^1^, M. Reay^2^

##### ^1^University Hospital, UHCW NHS Trust, General Critical Care, Coventry, United Kingdom; ^2^Dudley Group of Hospitals NHS Foundation Trust, Department of Anaesthetics and Intensive Care, Dudley, United Kingdom

###### **Correspondence:** C. Groves – University Hospital, UHCW NHS Trust, General Critical Care, Coventry, United Kingdom

**Introduction:** Ventilator Associated Pneumonia (VAP) is the most common hospital acquired infection in those requiring mechanical ventilation. It is associated with prolonged length of stay on the ICU, poorer outcomes and increased cost^1^.There is currently no 'gold standard' definition but a high clinical suspicion is often enough to begin treatment. Diagnosis can be complicated as the signs that are seen with VAP are not uncommon in those who are critically ill in ITU, potentially delaying treatment^1,2^.

**Objectives:** The aim of this project was to establish if there are significant differences between the Triggers which predicted VAPs (VAP triggers) as opposed to triggers which did not predict a VAP (Non VAP triggers) where both groups had triggered an alert.

**Methods:** All data from the Intensive Care Unit electronic recording system (ICIP Phillips) was queried to extract all VAP alerts triggered between June 2009 and March 2012. During this time period there were 28 VAP triggers and 130 non-VAP cases.

We looked at acknowledgment time, differences in values and order of the index parameters between VAP triggers and Non VAP triggers to look for differences suggesting early predictors for positive microbiology and radiology.

Microsoft Excel ® was used to manipulate data for analysis and SPSS Statistics 17.0 ® was used to calculate summary statistics, generate tables, perform the student's t-test analysis and produce graphs. Student's t-test was used to analyse statistical significance between groups.

**Results:** Of the quantitative variables measured, there was no statistically significant difference between the confirmed VAP cases and the non-VAP cases apart from in temperature with non-VAP cases having a higher average temperature, 38.2 °C, compared with 37.89 °C in confirmed VAP.

This notwithstanding we noted a lower mean C-rective protein (CRP), a higher oxygen requirement and a lower White Cell Count (WCC) in confirmed VAP cases.

As would be expected those in the VAP group had secretions most commonly described as creamy with moderate to copious amount.

In terms of trigger order in the confirmed VAP cases, secretion amount was the most common first and second trigger in the VAP alert followed by nonspecific inflammatory markers such as WBC, CRP and temperature.

In the non VAP group, temperature, oxygen requirement (PaO2/FiO2) and WBC were the most common first trigger factors.

**Conclusions:** In conclusion early Secretion quantity and description predict positive microbiology and radiology compared with other parameters such as temperature, WBC, CRP which do not.

**References**

1. Valencia M, Torres L. Ventilator-associated pneumonia. *Curr Opin Crit Care* Feb 2009; 15: (30-35)

2. Hunter JD, Ventilator Associated Pneumonia, *BMJ* May 2012; 344: (e3325-e3331)

**Grant acknowledgement**

No funding to declare.

## ECMO FOR MANAGEMENT OF RESPIRATORY FAILURE

### A78 Driving pressure associated mortality in acute respiratory distress syndrome with extracorporeal membrane oxygenation

#### L.-C. Chiu^1^, H.-C. Hu^1,2,3^, C.-Y. Hung^1^, C.-H. Chang^1^, S.-H. Li^1^, K.-C. Kao^1,2,3^

##### ^1^Chang Gung Memorial Hospital, Division of Thoracic Medicine, Taoyuan, Taiwan, Province of China; ^2^Chang Gung Memorial Hospital, Department of Respiratory Therapy, Taoyuan, Taiwan, Province of China; ^3^Chang Gung University College of Medicine, Department of Respiratory Therapy, Taoyuan, Taiwan, Province of China

###### **Correspondence:** K.-C. Kao – Chang Gung Memorial Hospital, Division of Thoracic Medicine, Taoyuan, Taiwan, Province of China

**Introduction:** The survival predictors and optimal mechanical ventilator (MV) settings in patients with severe acute respiratory distress syndrome (ARDS) undergoing extracorporeal membrane oxygenation (ECMO) are uncertain.

**Objectives:** To evaluate the influence of clinical variables and MV settings on intensive care unit (ICU) mortality for severe ARDS patients treated with ECMO.

**Methods:** We retrospectively reviewed severe ARDS patients who received ECMO due to refractory hypoxemia between May 2006 and October 2015. Serial MV settings during ECMO and factors associated with ICU mortality were analyzed.

**Results:** A total of 158 severe ARDS patients received ECMO were analyzed. Overall ICU mortality was 55.1 %. After ECMO initiation, nonsurvivors had significantly higher peak inspiratory pressure and driving pressure than survivors on day 2, day 3, and day 7 (32.8 ± 6.4 vs 30.6 ± 5.2; 32.9 ± 6.8 vs 30.4 ± 6.0; 33.1 ± 7.1 vs 29.9 ± 5.8 cm H_2_O, *p* < 0.05; 20.9 ± 6.8 vs 18.3 ± 5.9; 21.3 ± 7.2 vs 17.9 ± 6.8; 21.4 ± 7.5 vs 17.7 ± 6.8 cm H_2_O, *p* < 0.05). After multivariate analysis, mean driving pressure above 21 cm H_2_O during first 3 days on ECMO were independently associated with higher death (odds ratio, 2.968; 95 % confidence interval, 1.312-6.712; *p* = 0.009). Other factors independently associated with ICU mortality included ARDS duration before ECMO (odds ratio, 1.005; 95 % confidence interval, 1.001-1.010; *p* = 0.009), and Acute Physiology and Chronic Health Evaluation II score before ECMO initiation (odds ratio, 1.092; 95 % confidence interval, 1.032-1.156; *p* = 0.002).

**Conclusions:** Driving pressure during first 3 days of ECMO support in severe ARDS was independently associated with ICU mortality and level above 21 cm H_2_O was related to higher death. Further large multicenter, prospective, randomized, controlled trials are warranted to confirm our findings.

### A79 Circulating microparticles in patients with severe ARDS undergoing veno-venous ECMO: another piece of the inflammation puzzle?

#### A. Ballin^1^, F. Facchin^1^, G. Sartori^1^, F. Zarantonello^1^, E. Campello^2^, C.M. Radu^2^, S. Rossi^1^, C. Ori^3^, P. Simioni^2^

##### ^1^Azienda Ospedaliera di Padova, UOC Anesthesia and Intensive Care Unit, Padua, Italy; ^2^University of Padua, Thrombotic and Hemorrhagic Diseases Unit, Department of Medicine, Padua, Italy; ^3^Azienda Ospedaliera di Padova, UOC Anesthesia and Intensive Care Unit, Department of Medicine-DIMED, Padua, Italy

###### **Correspondence:** A. Ballin – Azienda Ospedaliera di Padova, UOC Anesthesia and Intensive Care Unit, Padua, Italy

**Introduction:** Acute Respiratory Distress Syndrome (ARDS) is a severe acute inflammatory lung injury with still high mortality, despite current lung protective ventilatory strategy and rescue therapies, such as veno-venous extracorporeal membrane oxygenation (VV-ECMO)^1^. During ARDS, immune system and inflammatory pathways are strongly activated, resulting in a systemic disease^2^. Microparticles (MPs), tiny cell-derived vescicles released from a variety of activated or apoptotic cells, play a role in several disease processes^3^. However, MPs role during vv-ECMO has not been described yet.

**Objectives:** To assess the presence, the origin of circulating microparticles in patients with severe ARDS treated with VV-ECMO, and their relation with the clinical course.

**Methods:** 15 consecutive patients admitted to our ICU for severe ARDS that required VV-ECMO were enrolled. Immediately before (T0), in the 2^nd^ day after placement (T1) and removal (T2) of VV-ECMO, arterial platelet free plasma was collected and analysed by Flow Cytometry to evaluate endothelial (EMPs), platelet-derived (PMPs), leucocyte-derived (LMPs), and tissue factor-bearing (TF-MPs) microparticles levels. Comparisons were drawn between different time points and survivors (n = 8) and non survivors (n = 7) with analysis by two-way ANOVA. P values < 0.05 were considered significantly different.

**Fig. 31 (abstract A79).**
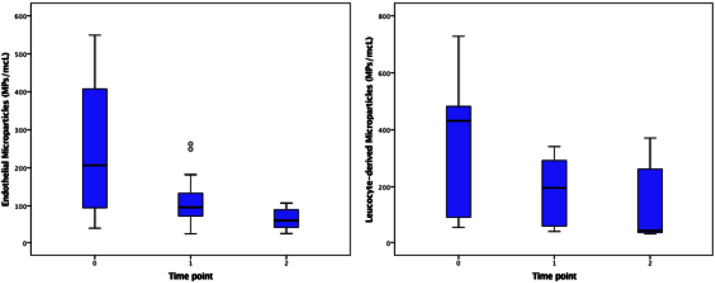
Endothelial and Leucocyte-derived circulating Microparticles (MPs) at T0: just before ECMO, T1: two days after ECMO start, and T2: two days after decannulation

**Results:** We found a progressive and significant reduction in both circulating EMPs (median 207, IQR 95-404 è median 64, IQR 45-90 MPs/mL, p = 0.013) and LMPs (median 432, IQR 93-479 èmedian 39, IQR 35-282 MPs/mL, p = 0.044) between the start of ECMO support (T0) and soon after the decannulation (T2), as shown in Fig. 31. Survivors showed higer levels of LMPs (p = 0.034) compared to non survivors and almost significantly higher levels of EMPs (p = 0.062). Standard laboratory tests (i.e. CRP, PCT, and WBC) did not show any significant difference between the same time points, and between different outcomes. There was no correlation between level of any kind of MPs and heparin dose, bleeding episodes, and mortality.

**Conclusions:** In patients with severe ARDS treated with VV-ECMO, plasma levels of both EMPs and LMPs were significantly elevated just before ECMO support, then gradually reduced during treatment and after decannulation. Moreover, blood passage through the ECMO circuit did not seem to generate more procoagulant, endothelial, leucocyte-derived or platelet-derived MPs, compared to baseline.

**References**

1 Bellani G. JAMA 2016, 315:8, 788-800.

2 Fujishima S. Journal of Intensive Care. 2014, 2:32.

3 McVey M. et al. Am J Physiol Lung Cell Mol Physiol 2012, 303: 364-81.

### A80 Factors affecting the outcome of extracorporeal membrane oxygenation: a single institution experience

#### N. Umei, I. Shingo

##### Nippon Medical School Hospital, Tokyo, Japan

###### **Correspondence:** N. Umei – Nippon Medical School Hospital, Tokyo, Japan

**Introduction:** The use of extracorporeal membrane oxygenation (ECMO) for acute respiratory distress syndrome (ARDS) that is unresponsive to conventional ventilatory management has been increasing rapidly. Therefore, in April 2015, we established a new ECMO program under the Japanese health care system without regionalization.

**Objectives:** This study aimed to determine whether the outcome of our ECMO program is associated with the inter-hospital transfer of patients.

**Methods:** The clinical data of patients treated with ECMO, from April 2015 to March 2016, were collected and analyzed retrospectively. The clinical data of patients who were transferred from other hospitals (transferred group) and those who were not (non-transferred group) were compared before and after the venovenous (VV) ECMO treatment

**Results:** During the study period, 20 patients were treated with VV-ECMO. The median age of the patients was 60 years. The median duration of mechanical ventilation prior to introducing ECMO was 2 days. The median duration of VV-ECMO was 7.2 days, and the total duration of intensive care unit stay was 23 days. Twelve (60 %) patients were successfully weaned from ECMO, and 10 (50 %) patients survived to discharge. However, the median respiratory ECMO survival prediction (RESP) score was -2.5, indicating that the estimated survival rate was low (35 %). Moreover, 70 % of patients were transferred from other hospitals. The results showed that the survival rate was higher for the transferred group than for the non-transferred group (57.1 % vs. 33.3 %, P = 0.6). The proportion of immunocompromised patients in the transferred group was lower than that in the non-transferred group (14.2 % vs. 50.0 %, P = 0.13). Before VV-ECMO treatment, the duration of mechanical ventilation in the transferred group was shorter than that in the non-transferred group (2 vs. 5.5 d, P = 0.047). Moreover, the RESP score for the transferred group was lower than that for the non-transferred group (-1 vs. -4, P = 0.0076). During the treatment with VV-ECMO, the duration of ECMO support in the transferred group was longer than that in the non-transferred group (8.6 vs. 7.0 d, P = 0.030)

**Conclusions:** The clinical outcomes of our program were inferior to those of advanced ECMO centers in Europe and North America. To improve the outcomes of ECMO for ARDS in Japan, our results suggest that regionalization should be employed such that ARDS patients with better RESP scores are transferred to an ECMO center.

**References**

1. Combes, Brodie D, Bartlett R et al. Position paper for the organization of extracorporeal membrane oxygenationprograms for acute respiratory failure in adult patients. Am J Respir Crit Care Med. 2014 Sep 1;190(5):488-96.

### A81 Comparing the prognosis of H_1_N_1_-associated acute respiratory distress syndrome with ARDS from other causes treated with ECMO support

#### A.C. Santos, C. Candeias, I. Moniz, R. Marçal, Z. Costa e Silva, J.M. Ribeiro

##### University Hospital of Santa Maria, CHLN, Intensive Care Department, Lisbon, Portugal

###### **Correspondence:** A.C. Santos – University Hospital of Santa Maria, CHLN, Intensive Care Department, Lisbon, Portugal

**Introduction:** Influenza A (H_1_N_1_)pdm09 pandemic was a determinant event for development of modern extracorporeal life support techniques. It still represents a frequent cause of conventional respiratory support failure, usually presenting with rapidly evolving critical hypoxemia demanding extracorporeal oxygenation (ECMO) rescue treatment.

**Objectives:** Identify characteristics of patients with the most severe forms of H_1_N_1_-associated ARDS in order to promote early identification of patients with putative need for ECMO treatment. Comparison of the ECMO H_1_N_1_-infected population with the ECMO treated population with ARDS from other causes in terms of physiological, functional and biomechanical ventilation parameters to rule prognosis prediction.

**Methods:** Retrospective review of prospectively collected data from a protocol-driven ECMO referral centre, with inter-hospital patient rescue capability and ELSO registered activity.

**Results:** Between 2011 and 2015, sixty one patients were treated with ECMO support for severe acute respiratory distress syndrome (*Quadrox HLS or PLS Oxygenator System*, Maquet). There were 25 patients with H_1_N_1_-associated ARDS and 34 patients with ARDS from other causes (two patients excluded because of insufficient data). H_1_N_1_-patients, when compared with non H_1_N_1_-patients, had similar age (50.0 ± 12.1 vs 46.0 ± 11.3; *p > 0.05*), SAPS II (41.8 ± 14.4 vs 39.3 ± 14.5; *p > 0.05*), pre-ECMO mechanical ventilation duration (4.2 ± 2.0 vs 6.4 ± 5.0 days; *p > 0.05*), hypoxemia degree (65.1 ± 17.9 vs 63.1 ± 9.0 mmHg; *p > 0.05*), static lung compliance at day 1 (22.3 ± 8.3 vs 23.6 ± 9.8 ml/cmH_2_O; *p > 0.05*) and average ECMO run duration (14.8 ± 9.7 vs 12.8 ± 9.4 days; *p > 0.05*). Patients with H_1_N_1_-ARDS had higher body mass index (32.2 ± 7.3 vs 29.6 ± 4.5; *p < 0.05*), higher Murray scores from more diffuse lung involvement (3.56 ± 0.30 vs 3.28 ± 0.34; *p < 0.05*), lower levels of PaCO_2_ (56.6 ± 18.3 vs 67.0 ± 20.2 mmHg; *p < 0.05*) and higher levels of pH (7.33 ± 0.8 vs 7.26 ± 0,11; *p < 0.05*) translating less severe alveolar ventilation compromise. Global survival was 73 % with no difference between the groups, and prognosis prediction scores were also similar (ECMOnet: 4.0 ± 1.6 vs 4.7 ± 2.1; *p > 0,05;* LIPS: 7.5 ± 1.8 vs 6.3 ± 2.4; *p > 0,05*).

**Conclusions:** Patients with H_1_N_1_-associated severe ARDS presented with predominantly hypoxemic respiratory failure, with more diffuse bilateral lung disease, higher Murray scores and less effective ventilation compromise, but those characteristics did not result in worse outcomes when compared with patients with severe ARDS from other causes.

### A82 In moderate to severe ARDS patients with severe respiratory acidosis, can we improve the arterial pH and make ultra-protective ventilation with the introduction of an extra-corporeal circulation CO2 removal (ECCO2r) technique

#### J.F. Georger, J.P. Ponthus, M. Tchir, V. Amilien, M. Ayoub, E. Barsam

##### Centre Hospitalier Intercommunal de Villeneuve Saint Georges, Lucie et Raymond AUBRAC, Reanimation Polyvalente - Surveillance Continue, Villeneuve Saint Georges, France

###### **Correspondence:** J.F. Georger – Centre Hospitalier Intercommunal de Villeneuve Saint Georges, Lucie et Raymond AUBRAC, Reanimation Polyvalente - Surveillance Continue, Villeneuve Saint Georges, France

**Objective:** In patients with moderate to severe ARDS and respiratory acidosis we can introduce ECCO2r to enable protective ventilation or ultra-protective ventilation. We don't know the results we can obtain after the introduction of ECCO2R in a population of ARDS with respiratory acidosis.The objective of this study is to describe if we can at the same time improved the blood pH and allow ultra-protective ventilation.

**Methods:** We retrospectively included patients who received ECCO2r for ARDS with respiratory acidosis between august 2014 and march 2016 in our ICU. The ECCO2R was performed with ILAACTIVE® device (Novalung®) with a Minilung® or ILA® membrane. The sweep gas was oxygen at 10 l/min. The vascular access was a 24 F dual light catheter in femoral position. The blood flow in the membrane was around 1.5 l/min. The ECCO2R was introduced in patients with PaO2/fiO2 ratio between 80 and 150 and acidosis. All the patients was ventilated in controled ventilation with 6 ml/kg (PBW) of tidal volume (Vt) and a respiratory rate (RR) above 25/min. All the patient was sedated with midazolam and Sufentanyl and if necessary we used neuromuscular blocking agent. If is necessary the clinician in charge of the patient performed prone position session during 16H.We collect pH, PaCO2, the PaO2/FiO2ratio, Vt, RR, PEEPtotal and driving pressure, before ECCO2r and at 4 h the initiation of ECCO2r then at J1, J2, J3. We compared the parameters with repeated measures ANOVA test.

**Results:** We included 16 patients, 9 males and 7 females. The average for the age was 67 years (36-84), for the BMI was 32.2 (22-60). The cause of the ARDS was a pneumonia for 14 patients, a cellulitis for 1 patient and a septicemia for the last patient. On eleven patients, we performed at least one prone position session, during 16 hours, with ECCO2R. The average duration of treatment by ECCO2R was 11 days (2-28).The mortality was 50 %, none patient died by a complication of the ECCO2R. We didn't have any hemorrhage complication on the catheter for ECCO2R.

The evolution of parameters was in the Table 32.

**Conclusion:** The ECCO2r for ARDS patients with respiratory acidosis helps to normalize the pH and decreased Vt and RR to make ultra-protective ventilation and decrease the driving pressure. In this type of ARDS with severe hypercapnia, others studies are necessary to know if this kind of procedure can improve the mortality of this patients.

**Table 32 (abstract A82).** Evolution of the parameters

	before ECCO2R	H4	J1	J2	J3
pH	7.20 (7.14-7.25)	7.33±(7.29-7.37)	7,33± (7.28-7.38)	7.37± (7.32-7.41)	7.35± (7.32-7.38)
PaCO2 (mmHg)	67 (59-75)	44± (39-49)	47± (39-54)	46± (40-52)	51± (43-59)
PaO2/fiO2	111 (91-132)	126 (103-149)	161 (136-186)	134 (110-157)	151 (123-179)
Vt (ml/kg (PBW))	6.1 (5.5-6.7)	4.4± (3.5-5.2)	4.4± (3.6-5.2)	4.2± (3.1-5.2)	4.3± (3.5-5.1)
RR (/min)	30.6 (28.3-32.8)	25.5 (21.1-29.9)	24.9± (21.4-28.5)	23.9± (20.3-27.5)	23.4± (19.7-27.2)
PEEP total (cmH2O)	14.8 (12.8-16.7)	15.4 (13.0-17.7)	15.2 (13.1-17.4)	13.9 (11-16.1)	14.9 (12,4-17,3)
Driving pressure(cmH2O)	11.9 (9.4-14.3)	8.6± (6.2-11.1)	8.6± (6.3-10.9)	9.6± (7.7-11.6)	8.8± (6.8-10.8)

(± mean that p< 0.05 compared to before ECCO2R)

### A83 Restrictive transfusion strategy in VV-ECMO for ARDS: ISMETT experience 2011-2015

#### G. Martucci^1^, G. Panarello^1^, F. Tuzzolino^2^, G. Capitanio^1^, V. Ferrazza^1^, T. Carollo^1^, L. Giovanni^1^, A. Arcadipane^1^

##### ^1^IRCCS-ISMETT Mediterranean Institute for Transplantation and Advanced Therapies, Department of Anesthesia and Intensive Care, Palermo, Italy; ^2^IRCCS-ISMETT Mediterranean Institute for Transplantation and Advanced Therapies, Statistics, Research Office, Palermo, Italy

###### **Correspondence:** G. Martucci – IRCCS-ISMETT Mediterranean Institute for Transplantation and Advanced Therapies, Department of Anesthesia and Intensive Care, Palermo, Italy

**Introduction:** In critically ill patients evidence suggests a conservative transfusion strategy. Some paper questioned the standard use of Hb 7 mg/dl trigger considering comorbidities and clinical picture. [1] Thresholds for transfusion of PRBC in ECMO are still a matter of debate, because PRBC given to increase DO2 act on a critical point of patients with ARDS and consumption coagulopathy and bleeding are frequent in ECMO. ELSO guidelines suggest to keep Hb at a normal value (12-14 g/dl) and frequently 2-3 PRBC units are transfused daily, but also in this setting have been reported lower Hb trigger for transfusion. [2]

**OBJECTIVE.** Describe transfusion practice in VV-ECMO for severe ARDS (Berlin definition) at ISMETT (2011-2015)

**Methods:** Our blood management strategy is based on:

1. Hb level 8-10 according to SvO2, metabolic and perfusion data
2. transfusion using antileukocyte filters
3. low dose anticoagulation by heparin (aPTT range 40-50)
4. anticoagulation stop in case of bleeding
5. autotransfusion of the blood in the circuit at decannulation if possible.

Retrospective observation analysis from electronic medical charts. Data management performed by STATA 13.1

**Results:** Results are reported as mean value +/- standard deviation or total number and percentage. In case of high variability median and interquartile range are reported and marked as *. During the selected period we run 59 ECMO for severe ARDS due to: H1N1 (28), Bacterial Pneumonia 15), Politrauma (8), Post-pneumonectomy (2), Pneumocystis Pneumonia (2), Lung graft failure and pneumonia (2), Complicated pleural empyema (1), Chemical pneumonia (1).

Main results are 78 % of ECMO weaning and 71.2 % of ICU discharge.

**Conclusion:** Transfusion practice at or institution has not a definite trigger, and since several years we abandoned the suggested Hb value tacking more into account functional data. This is possible thanks to high blood flow reached and high efficiency of oxygenators. Decision is left to the care team considering actual DO2, SvO2, hemodynamic parameters, lactates, comorbidities, sepsis, circuit function. We reach a 78 % weaning from ECMO (2016 ELSO registry 66 %) with main result of 5 patients without transfusion and a median rate of PRBC transfusion of 125 ml/day of ECMO support that is higher than reports that use lower Hb trigger, but probably are quite restrictive considering the severity of patients supported.

Related to the transfusion strategy we report a 18 % of bleeding needing a treatment (stop anticoagulation or surgical/endoscopic) and 8.5 % of patients needing FFP and 23 % needing PLT.

**References**

1. Lelubre C, et al. Red blood transfusion strategies in critically ill patients: lessons from recent randomized clinical studies. Minerva Anestesiol 2016; Jan Epub

2. Agerstrand CL, et al. Blood conservation in extracorporeal membrane oxygenation for acute respiratory distress syndrome. Ann Thorac Surg 2015;99(2):590-5

**Table 33 (abstract A83).** Demographics

N.	Male N (%)	Age years	BMI	SAPS II	Sofa	LOS preECMO	Mech. Vent. preECMO	PaO2/FiO2	Murray score
59	49 (83)	43±12	29±5	42±12	9±3	5 (6)*	4 (7)*	63±17	3.5±0.25

**Table 34 (abstract A83).** Transfusion parameters

Mean HTC	ECMO Days	PRBC total ml/pt	PRBC Day ml/pt/day	No PRBC N. (%)	FFP N. (%)	PLT N. (%)	Bleeding N. (%)
30±3	13 (14)*	1500(3100)*	125(141)*	5 (8.5)	5 (8.5)	14(23)	11(18)

**Table 35 (abstract A83).** Predictive score and outcomes

Preserve score	ECMOnet score	RESPSCORE	ECMO survivors N. (%)	ICU discharge N. (%)	CRRT N. (%)	Mech.Vent. post ECMO	LOS ICU post ECMO	LOS TOT Hospital
4±2	5.8±1.5	1(6)*	46(78)	42 (71.2)	32 (54)	10 (12)*	14.5 (12)*	30 (24)*

### A84 Prealbumin level and body mass index before ECMO initiation as prognostic factors in patients with lung transplantation

#### M. López Sánchez^1^, M.A. González-Gay^2^, F.J. Llorca Díaz^3^, M.I. Rubio López^1^

##### ^1^Hospital Universitario Marqués de Valdecilla, Intensive Care Unit, Santander, Spain; ^2^Hospital Universitario Marqués de Valdecilla, Rheumathology Service, Santander, Spain; ^3^Universidad de Cantabria, Preventive Medicine and Public Health, Santander, Spain

###### **Correspondence:** M. López Sánchez – Hospital Universitario Marqués de Valdecilla, Intensive Care Unit, Santander, Spain

**Introduction:** Indications for extracorporeal membrane oxygenation (ECMO) use in lung transplantation (LT) are: bridge to transplantation, intraoperative extracorporeal respiratory and/or circulatory support and treatment of primary graft dysfunction (PGD) in postoperative period. Several mortality risk factors pre-ECMO initiation has been investigated. Prealbumin and body mass index (BMI) are predictors of mortality in critically ill patients.

**Objectives:** To estimate if nutritional state at the beginning of ECMO is associated with mortality.

**Methods:** Retrospective observational study between January 2009 - March 2016, in 12 beds intensive care unit (ICU) of a tertiary hospital center. Inclusion criteria for ECMO entry: patients listed for LT as a bridge to transplant, intraoperative extracorporeal respiratory and/or circulatory support and PGD. ECMO systems: centrifugal pump and polymethylpentene membrane oxygenation with Bioline® coated circuits and cannulas.

Demographic data (included BMI), level of prealbumin and time of mechanical ventilation (MV) were collected at the time of ECMO initiation. ECMO indications, type of ECMO support and ICU mortality were also recorded. Prealbumin level and BMI were compared between survivors and non-survivors. Continuous variables, reported as mean ± standard deviation (SD) or median with range were compared using the Student t-test. Categorical variables were compared using the X^2^ test. Analysis was performed using SPSS ver. 22.0 and value of p ≤ 0.005 was considered to be statistically significant.

**Results:** 33 patients (p), 22 male and 11 females were included with a median age of 49.63 years (SD ± 13.37, interval 16-66. Median simplified Acute Physiology Score (SAPS II) was 38.63 ± 12.97. Pulmonary fibrosis was the most frequent diagnosis (13 p). In 16 p (50 %) MV was started before ECMO support, with a median of 24.27 hours (interval 0-146). Intraoperative support was used in 17 p (2 p respiratory, 10 p cardiac and 5 p both support); bridge to LT in 8 p (4 p respiratory, 3 p cardiac and 1 p both support); PGD was the indication in 8 p. In 4 p ECMO was placed after cardiopulmonary bypass. Type of ECMO support was peripheral venous-arterial in 19 p, central venous- arterial in 2 p and venous-venous in 12 p. ICU mortality was 42.42 %. Median BMI in survivors was 24.85 ± 5.19 and 24.99 ± 4.16 in nonsurvivors. Median prealbumin level in survivors was 14.09 ± 4.69 and 14.28 ± 10.66 in nonsurvivors. No statistical significance was observed in BMI or prealbumin level between survivors and nonsurvivors.

**Conclusions:** In this review with ECMO patients and LT, there are not statistically significant differences in prealbumin level and BMI between survivors and nonsurvivors at the time ECMO initiation.

**References**

1. Wagner K, Risnes I, Abdelnoor M, Karlsen HM, Svennevig JL. Is It possible predict outcome in pulmonary ECMO? Analysis of pre-operative risk factors. Perfusion 2008;23:95-9.

### A85 VV-ECMO in ARDS: toward a lower anticoagulation ratio?

#### E. Zogheib^1,2^, L. Villeret^1^, J. Nader^3^, M. Bernasinski^1^, P. Besserve^1^, T. Caus^2,3^, H. Dupont^1,2^

##### ^1^CHU Amiens - Picardie, Cardio Thoracic and Vascular Intensive Care Unit, Amiens, France; ^2^Université de Picardie Jules Verne, CURS, Amiens, France; ^3^CHU Amiens - Picardie, Cardiac Surgery, Amiens, France

###### **Correspondence:** E. Zogheib – CHU Amiens - Picardie, Cardio Thoracic and Vascular Intensive Care Unit, Amiens, France

**Introduction:** Venovenous ECMO (VV-ECMO) is a rescue therapy in refractory ARDS. Despite technological progress and enhanced biocompatibility, anticoagulation with unfractionated heparin remains the most often used as recommended. Bleeding complications remains a main cause of increasing morbidity and mortality in patients under VV-ECMO.

**Objectives:** We aimed to study the effectiveness of the dosage of unfractionated heparin (CAW and heparinization, anti-Xa) in patients on VV-ECMO for ARDS.

**Methods:** We performed a retrospective observational study from 2008 to the first quarter 2015. Patients' baseline characteristics, the dose of UFH anticoagulation and biological data of anticoagulation (APTT, anti-Xa, HEMOCHRON ACT, ATIII, fibrinogen) were collected and we studied the incidence of haemorragic or thromboembolic events under UFH anticoagulation.

**Results:** 48 patients with ARDS requiring VV-ECMO were included,with a majority of men (35 (63.6 %) vs 20 (36.4 %) (p < 0.5) The mean age was. 47 years (+/- 15 years). In 35.4 % of cases, a double-lumen cannula (Avalon®) was used (mean diameter of 27GB), while the others had a femorà-jugular cannulation with an average diameter of femoral vein to 25GB. UFH anticoagulant was used in most patients. 33 % of patients did not have UFH during installation of VV-ECMO. The average value of APTT was 35 +/- 8 secondes, the anti-Xa to 0.14 +/- 0.06. The average of the Hemochron was 171 +/- 23 sec. The values of UFH administered averaged 778 +/- 601 IU / h IVSE. Bleeding complications were noted in 56.25 % of cases. None of our patients had ECMO thrombosis cannulas or oxygenator. 39 % of patients had positive anti-PF4 and only 11 patients had a change in anticoagulation.

**Conclusions:** Anticoagulation used in our study appears lower than recommendations without increasing thromboembolic complications, with persistence of a high rate of bleeding complications with or without a direct connection with ECMO. Does these results reinforce the idea of lowering the anticoagulation ratio on patients under VV-ECMO?

**Note:** This abstract has been previously published and is available at [2]. It is included here as a complete record of the abstracts from the conference.

**References**

1. Extracorporeal life support for patients with acute respiratory distress syndrome: report of a Consensus Conference. Ann Intensive Care. 2014 May 24;4:15

2. Zogheib E, Nader J, Villeret L, Guilbart M, Pesserve P, Caus T, Dupont H (2016) VV-ECMO on ARDS: towards a lower coagulation ration? Ann Intensive Care 6(Suppl 1):P10

**Grant acknowledgement**

ECMO 80

### A86 Effect of citrate anticoagulation on CO2 extraction during low flow extracorporeal veno-venous CO2 removal therapy

#### P. Morimont^1,2^, S. Habran^2^, R. Hubert^2^, T. Desaive^2^, F. Blaffart^3^, N. Janssen^2^, J. Guiot^1^, A. Pironet^2^, P. Dauby^2^, B. Lambermont^1^

##### ^1^University Hospital of Liège, Medical and Coronary Intensive Care, Liege, Belgium; ^2^University of Liège, GIGA Research, Liège, Belgium; ^3^University Hospital of Liège, Perfusion School, Liege, Belgium

###### **Correspondence:** P. Morimont – University Hospital of Liège, Medical and Coronary Intensive Care, Liege, Belgium

**Introduction:** Low flow extracorporeal veno-venous CO2 removal therapy (ECCO2RT) in addition to mechanical ventilation is used to remove CO2 while allowing protective ventilation (PV) during ARDS. However, this technique requires anticoagulation that may induce severe bleeding in critically ill patients (1). An alternative method consists in using citrate anticoagulation.

**Objectives:** The aim of this study was to assess the effect of citrate anticoagulation on CO2 extraction during ECCO2RT.

**Methods:** This study was conducted on an experimental model of severe hypercapnic acidosis performed in 2 groups of 3 pigs. In the first group (heparin group), pigs were anticoagulated with a standard protocol of unfractionated heparin while citrate was used for ECCO2RT device anticoagulation in the second group (citrate group). After sedation, analgesia and endotracheal intubation via a cervical tracheostomy, pigs were connected to a volume-cycled ventilator (tidal volume 10 mL/Kg, respiratory rate 20/min, FiO2 1.0, PEEP 5 cmH2O). Severe hypercapnic acidosis was obtained by reducing tidal volume by half. ECCO2RT was started in both groups when arterial pH was lower than 7.2. Pump Assisted Lung Protection (PALP, Maquet, Germany) system was used with two small cannulas to remove CO2. Blood flow in the PALP was successively set at 200, 400, 600 and 0 mL/min, each setting lasting 60 minutes. Sweep gas flow was set at 10 L/min. CO2 extraction, arterial pH, PaCO2 as well as systemic and pulmonary pressures were continuously followed.

**Results:** CO2 extraction is depicted in graph 1. Mean arterial pH was normalized to 7.37 ± 1.4 at an extracorporeal blood flow of 400 mL/min, coming from 7.11 ± 1.3. Arterial pH did not significantly changed in the citrate group as compared to the heparin group.

**Conclusions:** Using citrate anticoagulation during ECCO2RT is feasible. A trend toward better CO2 extraction was observed in the citrate group but not statistically significant as compared to the heparin group.

**References**

(1) Kluge S et al., Intensive Care Medicine 2012, 38: 1632-1639. Avoiding invasive mechanical ventilation by extracorporeal carbon dioxide removal in patients failing noninvasive ventilation.

**Grant acknowledgement**

Leon Fredericq Foundation of the University of Liège, Belgium

**Fig. 32:**
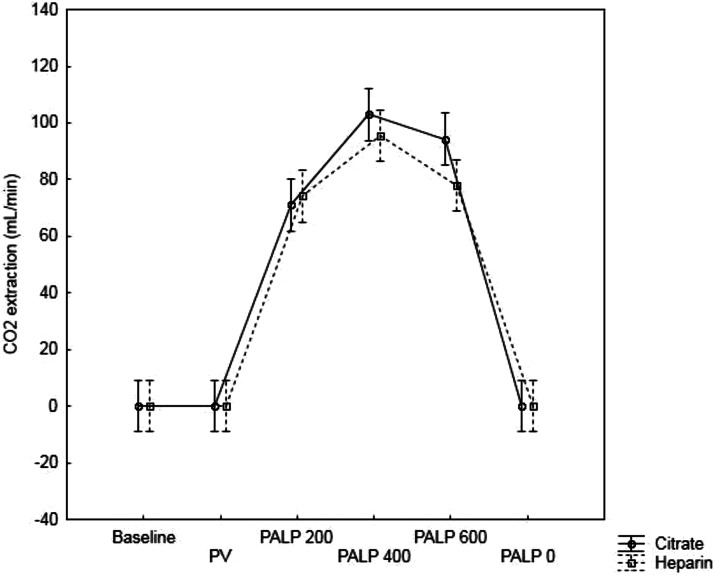
(abstract A86).

### A87 Point-of-care rotational thromboelastometry and platelet aggregometry during ECMO for ARDS

#### F. Zarantonello^1^, A. Ballin^1^, F. Facchin^1^, G. Sartori^1^, E. Campello^1^, T. Pettenuzzo^1^, G. Citton^1^, S. Rossi^2^, P. Simioni^1^, C. Ori^1^

##### ^1^University of Padova, Department of Medicine (DIMED), Padova, Italy; ^2^Azienda Ospedaliera di Padova, Emergency Department, Padova, Italy

###### **Correspondence:** F. Zarantonello – University of Padova, Department of Medicine (DIMED), Padova, Italy

**Introduction:** Extracorporeal Membrane Oxygenation (ECMO) is one of the rescue therapies in severe acute respiratory distress syndrome (ARDS)^1^. The need for anticoagulation and the activation of clotting cascade caused by ECMO circuit put patients at risk of bleeding and thrombosis. Conventional coagulation tests fail to fully describe such a complicated process, but the use of point of care (POC) methods like thromboelastometry and platelet aggregometry may provide additional information on clot development, lysis and platelet activity. However, the role of POC tests during veno-venous (VV) ECMO for ARDS still remains unclear^2^.

**Objectives:** To assess the association between POC tests and episodes of hemorrhage during ECMO.

**Methods:** Blood samples for rotational thromboelastometry (ROTEM®) and aggregometry (Multiplate®) were collected immediately before starting ECMO, every 2 days for the first 10 days and 48 hours after the removal of the circuit. Prothrombin time (PT), activated partial thromboplastin time (aPTT), platelet count (PLT), anti-Xa activity, plasma fibrinogen levels and activated clotting time (ACT) were recorded. Bleeding episodes were defined as severe when associated with the administration of at least 2 units of packed red blood cells (RBC).

**Fig. 33:**
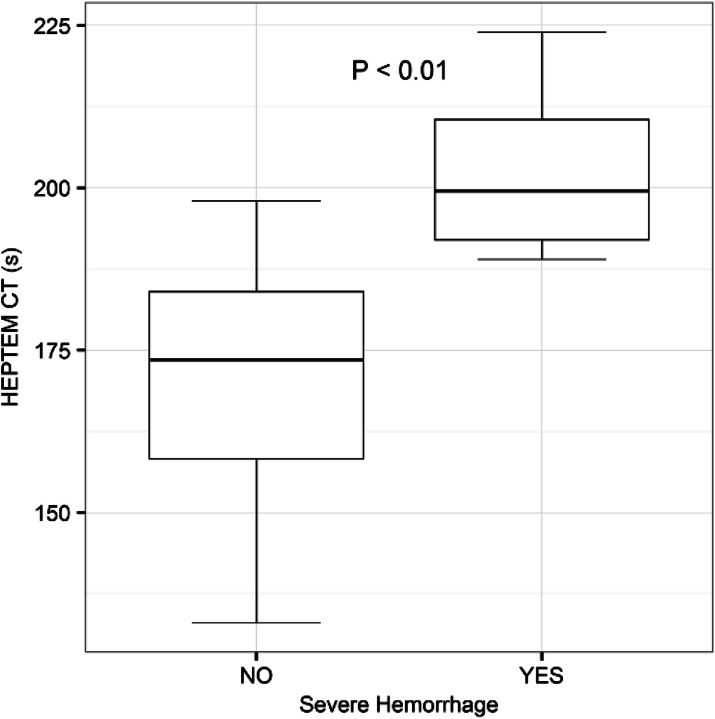
(abstract A87).

**Fig. 34:**
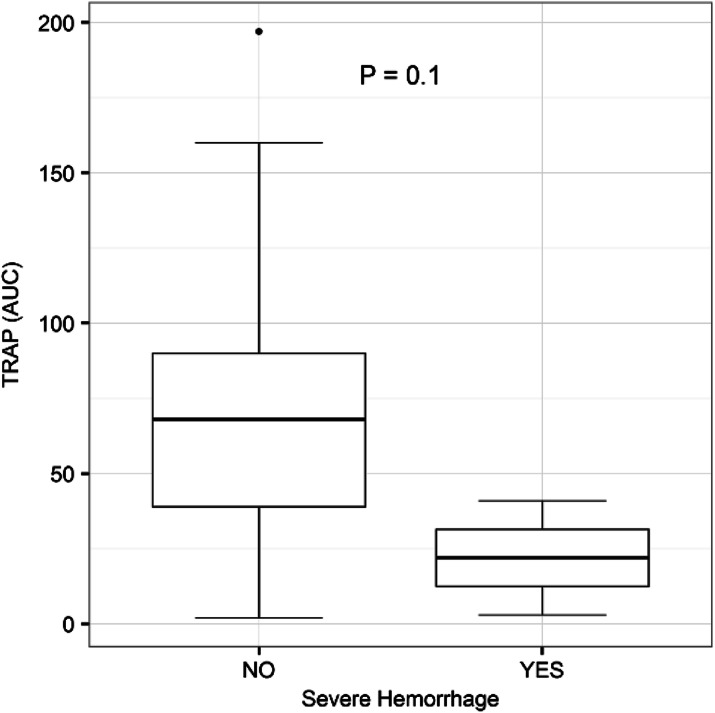
(abstract A87).

**Results:** We enrolled 15 severe ARDS patients requiring VV-ECMO. ECMO median duration was 9 (interquartile range 8-14.5) days. Mortality was 53.3 %. We found a strong correlation between maximum clot firmness (MCF) and amplitude of the curve at 10 minutes (A10) in all ROTEM tests (Rho > 0.96, p < 0.001). 67 % of patients had at least one episode of bleeding. 30.5 % of the hemorrhages were severe. PLT was lower in bleeding compared to non-bleeding group (p = 0.02). Among the ROTEM tests, heparinase modified thromboelastometry clotting time and clot formation time (HEPTEM CT and CFT) were significantly higher in bleeding group (p < 0.05) (Fig. 33). The POC tests evaluating the intrinsic and extrinsic coagulation pathways, as well as the thrombin receptor activating peptide-6 test (TRAP-AUC) assessing platelet aggregometry, were different between groups, even though not statistically significant (Fig. 34). There was no difference in PT, aPTT, anti-Xa activity, fibrinogen and ACT between groups.

**Conclusions:** ROTEM A10 is strongly correlated with the MCF, providing a fast coagulation assessment during ECMO. Bleeding episodes are not predictable using conventional laboratory coagulation tests, except for PLT. We found an association only between HEPTEM CT and CFT and hemorrhage. Increasing the sample size might identify other POC tests useful to predict and monitor hemorrhagic and thrombotic complications.

**References**

1. A-NZ ECMO influenza investigators. Extracorporeal Membrane Oxygenation for 2009 Influenza A(H1N1) Acute Respiratory Distress Syndrome. JAMA 2009; 302: 1888-95

2. Bolliger D et al. Point-of-care coagulation management algorithms during ECMO support: are we there yet? Minerva Anestesiol. 2016 Mar 30.

### A88 Pilot study of extracorporeal carbon dioxide removal in chronic obstructive pulmonary disease patients with late NIV failure

#### C. Kirakli, O. Ediboglu, S. Ataman, M. Yarici, F. Tuksavul

##### Dr. Suat Seren Chest Diseases and Surgery Training Hospital, Intensive Care Unit, Izmir, Turkey

###### **Correspondence:** C. Kirakli – Dr. Suat Seren Chest Diseases and Surgery Training Hospital, Intensive Care Unit, Izmir, Turkey

**Introduction:** Noninvasive ventilation (NIV) is routinely used in hypercapnic patients with an acute exacerbation of COPD. Extracorporeal CO_2_ removal (ECCO_2_R) techniques are being evaluated in patients who have NIV failure but the feasibility of these techniques in late NIV failure (after 24 hours) is unknown (1,2).

**Objectives:** We tried to assess the feasibility of a single site ECCO_2_R system in hypercapnic patients with an acute exacerbation of COPD who had late NIV failure.

**Methods:** Five hypercapnic COPD patients who were admitted to the ICU with an acute exacerbation whose respiratory acidosis did not improve despite 24 hours of NIV were enrolled in this single center, prospective, feasibility trial. A low flow single site ECCO_2_R system (ALung Technologies, Pittsburgh, Pa) was used. The primary endpoint was the time to normalization of arterial blood gas pH levels (above 7.35). Data are expressed as median (25^th^-75^th^ percentiles).

**Results:** The pH and PaCO_2_ levels at ICU admission were 7.24 (7.17-7.27) and 92 (75-125) mmHg respectively. pH and PaCO_2_ levels just before the initiation of ECCO_2_R were 7.29 (7.27-7.33) and 93 (70-104) mmHg respectively. ECCO_2_R was started after 72 (36-170) hours of NIV treatment. Arterial blood gas pH levels came to normal ranges after 22 (12-43) hours of ECCO_2_R treatment. In one patient ECCO_2_R was stopped due to clotting in the circuit. No other complications were observed regarding the use of ECCO_2_R system.

**Conclusions:** ECCO_2_R systems can be an alternative in COPD patients who are having late NIV failure. The system seems to be safe due to its low flow characteristics but clotting complication must be kept in mind. Further studies are needed to emphasize these results and also test the impact of this procedure on other outcomes like ventilator free days, duration of ICU stay, cost and mortality.

**References**

1. Burki NK, Mani RK, Herth FJ, Schmidt W, Teschler H, Bonin F, Becker H,

Randerath WJ, Stieglitz S, Hagmeyer L, Priegnitz C, Pfeifer M, Blaas SH, Putensen C, Theuerkauf N, Quintel M, Moerer O. A novel extracorporeal CO(2) removal system: results of a pilot study of hypercapnic respiratory failure in patients with COPD. Chest. 2013 Mar;143(3):678-86. doi: 10.1378/chest.12-0228.

2. Kluge S, Braune SA, Engel M, Nierhaus A, Frings D, Ebelt H, Uhrig A, Metschke M, Wegscheider K, Suttorp N, Rousseau S. Avoiding invasive mechanical ventilation by extracorporeal carbon dioxide removal in patients failing noninvasive ventilation. Intensive Care Med. 2012 Oct;38(10):1632-9.

### A89 Experience of extracorporeal support using pumped veno-veno extracorporeal carbon dioxide removal in ventilated patients: with severe acute respiratory failure

#### S. Keating^1^, A. Gibson^2^, M. Gilles^2^, M. Dunn^2^, G. Price^2^, N. Young^2^

##### ^1^Royal Infirmary of Edinburgh, Ward 118, Edinburgh, United Kingdom; ^2^Royal Infirmary of Edinburgh, Edinburgh, United Kingdom

###### **Correspondence:** N. Young – Royal Infirmary of Edinburgh, Edinburgh, United Kingdom

**Introduction:** Severe acute respiratory distress syndrome (ARDS) is associated with a mortality of 46.1 %[1]. Currently only low tidal volume ventilation has been proven to reduce mortality in all patients with ARDS[2]. This is frequently difficult to achieve in the presence of hypercapnia. Decreases in ventilatory driving pressure have been strongly associated with ICU survival[3]. It has been hypothesised that ventilation combining reduced tidal volumes with extracorporeal carbon dioxide removal (ECCOR) may result in further improvements in mortality[4], and prospective randomised trials are planned [5].

**Objectives:** To assess in patients with severe ARDS and acidosis secondary to hypercapnia if by using veno-veno ECCOR there can be a normalisation of pH and reduction of peak airway pressures. Secondary outcomes - survival to hospital discharge and complications of therapy.

**Methods:** Data on ventilatory and arterial blood gas parameters before and during therapy was prospectively collected and entered into the Extracorporeal Life Support Organisation (ELSO) registry in line with national guidance[6]. The Hemolung RAS (A Lung technologies) was used to provide ECCOR.

**Results:** 4 patients received ECCOR using the Hemolung. All patients had an improvement in the degree of acidosis with mean hydrogen ion concentration falling from 72.65 nM (pH 7.14) to 46.59 nM (pH 7.33) over the first 24 hours, with a mean PaCO2 fall from 13.5 kPa to 8.7 kPa. Peak airway pressures dropped from 31.5 cmH2O to 29.1 cmH2O over the same time period. There were no direct complications of therapy. 3 patients survived to hospital discharge.

**Discussion:** Using ECCOR locally in patients with severe ARDS it is possible to lower the hydrogen ion concentration improving the degree of acidosis. This corresponds to a trend in lower peak airway pressures, which in this small case series does not reach statistical significance. There were no complications of the therapy and mortality was shown to be lower then quoted in other ARDS trials [1].

**References**

1. Bellani et al.: Epidemiology, Patterns of Care, and Mortality for Patients With Acute Respiratory Distress Syndrome in Intensive Care Units in 50 Countries. JAMA. 2016;315(8):788-800

2. ARDS Network: Ventilation with lower tidal volumes as compared with traditional tidal volumes for acute lung injury and the acute respiratory distress syndrome. New England Journal of Medicine 2000, 342:1301-1308

3. Amato et al.: Driving Pressure and Survival in the Acute Respiratory Distress Syndrome. New England Journal of Medicine 2015; 372:747-755

4. Bein et al.: Lower tidal volume strategy (3 ml/kg) combined with extracorporeal CO2 removal versus 'conventional' protective ventilation (6 ml/kg) in severe ARDS. Intensive Care Med 2013 39:847-856

5. pRotective vEntilation with veno-venouS lung assisT in respiratory failure http://www.nets.nihr.ac.uk/projects/hta/1314302 (accessed 2/4/16)

6. https://www.nice.org.uk/guidance/ipg428 (accessed 2/4/2016)

### A90 Our experience with A-V ECCO2r device (Novalung iLA) in the district general hospital setting

#### P. Remeta^1^, P. Bishop^2^

##### ^1^PAH Harlow, Intensive Care, Harlow, United Kingdom; ^2^Colchester NHSFT, Intensive Care, Colchester, United Kingdom

###### **Correspondence:** P. Remeta – PAH Harlow, Intensive Care, Harlow, United Kingdom

**Introduction:** We would like to present our experience with use of arterio-venous extracorporeal membrane carbon dioxide removal (ECCO2R) device- Novalung iLA in the District General Hospital setting.

**Objectives:** We analysed our data to review our practice in use of ECCO2R in respiratory failure.

**Methods:** Over the period of 5 years we used arterio-venous ECCO2R device Novalung iLA in 10 patients, with overall 50 % mortality. We analysed retrospectively only 8 patients due to lack of complete documentation in 2 patients (survivors).

**Results:** From a reviewed sample of 8 patients three patients (37 %) survived. Most common diagnosis in our population was pneumonia (6), others were ARDS post cardiac arrest (1) and refractory asthma (1). We compared our patients in groups of survivors and non-survivors.

Mean age of survivors was 29 (18-39) vs 70.8 (59-80) of non-survivors. Mean APACHE score was 13.6 vs 21. Two of three survivors had single organ failure, one required CRRT for AKI. All patients in non-surviving group had multi-organ failure. Mean time to initiation of ECCO2R support post ITU admission was 39 vs 80 hours (11-75 vs 6.5-174 hours).

In all patients mean paCO2 before initiation of ECCO2R was 10.89 kPa, after the first hour 7.59 kPa and after 24 hours 5.9 kPa. No ECCO2R device associated complications in survivors dgroup, one limb ischaemia in non-survivors group.

**Conclusion:** Our analysis showed that the use of ECCO2R device significantly improved CO2 elimination in all our patients, allowed us to use lung protective ventilation strategy. Survival of 37 % in our small group is clearly influenced by selection of our patients.

We tried to identify a group of patients who would most likely benefit from this invasive intervention. In our small group of patients were early initiation, lower APACHE score, single organ failure and young age factors predicting good outcome.

We continue to audit use of ECCO2R device on our unit and currently using veno-venous ECCOR2 device which eliminates the risk of significant complication of A-V ECCOR2 - limb ischaemia.

### A91 Resolving dudes:IABP before CABG or levosimendan after?

#### M.D. Fernández Zamora^1^, J. Muñoz-Bono^1^, E. Curiel-Balsera^1^, E. Aguilar-Alonso^2^, R. Hinojosa^3^, A. Gordillo-Brenes^4^, J.A. Arboleda-Sánchez^5^, ARIAM-CARDIAC SURGERY PROJECT AUTHORS

##### ^1^Hospital Regional Málaga, Intensive Care, Málaga, Spain; ^2^Hospital Infanta Margarita, Intensive Care, Cabra, Spain; ^3^Hospital Virgen del Rocio, Intensive Care, Sevilla, Spain; ^4^Hospital Puerta del Mar, Intensive Care, Cádiz, Spain; ^5^Hospital Regional Málaga, Málaga, Spain

###### **Correspondence:** M.D. Fernández Zamora – Hospital Regional Málaga, Intensive Care, Málaga, Spain

**Introduction:** Meta-analyses suggest that levosimendan is superior to traditional inotropes, with decreased postoperative morbidity, improved hemodynamic function and decreased myocardial injury but at no time has been compared to the balloon counterpulsation.

**Methods:** Observational prospective admitted patients in ARIAM registry from February2010 to September 2012 in 13 public and private hospitals with CCV. We retrospectively compared the patients who underwent CABG who were treated with IABP before surgery and those who received levosimendan during surgery or immediately after surgery. We analysed clinical and epidemiological data of both groups as well as complications, hospital stays and mortality. We used the Mann Whitney U test and Fischer´s exact test with an alpha maximum of 5 % for the comparison of variables according to necessity.

**Results:** 949 patients were involved CABG in the time period studied. Of these, 17 patients were treated with BCIAO before surgery and 96 received levosimendan. No differences in age or comorbidities between the two groups analysed or extracorporeal circulation time (p > 0.05). However, functional status at the time of surgery was worse in the group BCIAo (NYHA III or IV 43.8 % from 22.2 %) p = 0.011, and the risk score used (EuroSCORE p = 0, 0001), 30 % of patients had no postoperative complications, similar in both groups. There were no differences in hospital stay or mortality in both groups. The group of patients treated with levosimendan developed lower rate of heart failure, renal failure (Creat > 2 mg / dl) and prolonged mechanical ventilation than the group that received BCIAO (p < 0.05). However, the levosimendan group required in 4.2 % of patients while it was not necessary in the other group.

**Conclusions:** levosimendan in heart coronary artery bypass surgery decreases postoperative morbidity (heart failure, renal failure or prolonged mechanical ventilation)with an increase in the need for CRRT. No differences were found in mortality or hospital stay.

**References**

Levosimendan Versus an Intra-aortic Balloon Pump in Adult Cardiac Surgery Patients. JCTVA 25:1154.(Wessex Cardiothoracic Centre, University of Southampton, Southampton General Hospital, Southampton, United Kingdom). The Calcium Sensitizer Levosimendan Gives Superior Results to Dobutamine in Postoperative Low Cardiac Output Syndrome R L Levina et al Servicios de Cirugía y Recuperación Cardiovascular, Hospital Universitario de la Universidad Abierta InterAmericana y del Hospital Francés, Buenos Aires, Argentina

## FLUID BALANCE, THERAPIES AND OUTCOMES IN AKI

### A92 Association between fluid overload at initiation of renal replacement therapy and outcome in critically ill patients: with acute kidney injury

#### I. Skorniakov^1,2^, D. Vikulova^2^, C. Whiteley^1^, O. Shaikh^1^, A. Jones^1^, M. Ostermann^3^

##### ^1^Guy's & St Thomas' NHS Foundation Trust, London, United Kingdom; ^2^Sverdlovsk Regional Clinical Hospital 1, Ekaterinburg, Russian Federation, ^3^Guys and St Thomas' NHS Foundation Trust, London, United Kingdom

###### **Correspondence:** M. Ostermann – Guys and St Thomas' NHS Foundation Trust, London, United Kingdom

**Introduction:** Fluid overload is associated with worse outcomes in critically ill patients.

**Objectives:** The aim of this study was to investigate the impact of fluid overload (FO) at initiation of renal replacement therapy (RRT) in critically ill patients with acute kidney injury (AKI).

**Methods:** We performed a retrospective analysis of all patients who were treated with RRT for AKI in the multi-disciplinary Intensive Care Unit (ICU) at a university hospital in London (UK) between 2012 - 2015.Total cumulative fluid balance on day of initiation of RRT was used to describe fluid accumulation and estimated in % of baseline body weight (BW). FO was defined as fluid accumulation greater than 10 % of BW. We collected data related to patient demographics, anthropometrics and SOFA score. Outcomes were hospital mortality and length of stay in ICU.

**Fig. 35:**
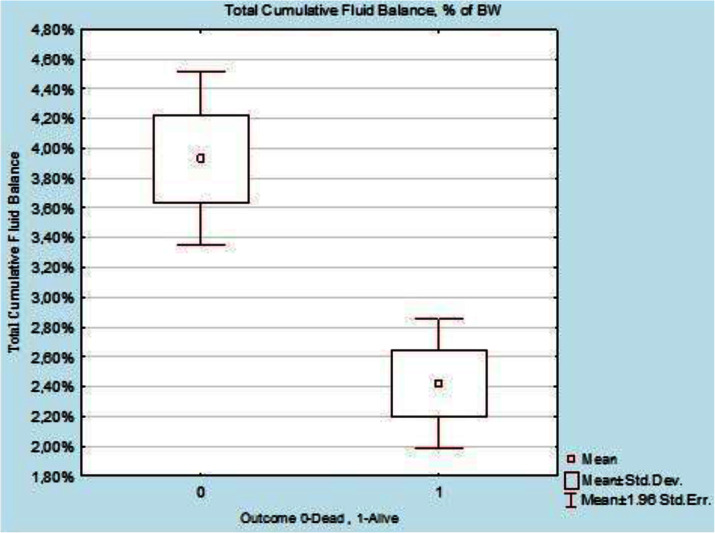
(abstract A92).

**Results:** 1129 patients received RRT for AKI of whom 42 % died in hospital. There was a significant difference in cumulative fluid balance at initiation of RRT between hospital survivors and non-survivors. (Fig. 35)

108 patients (9.6 %) had FO >10 %. They had a significantly higher hospital mortality (X^2^ = 5,89; p = 0,015) and longer stay in ICU (19.6 days versus 12.8; p < 0,001) but also higher SOFA score compared to patients with FO ≤10 %. (Table 36)

**Conclusions:** In critically ill patients with AKI, fluid overload at initiation of RRT was associated with a significantly higher mortality and longer stay in ICU.

**Grant acknowledgement:** This research was solely supported by the European Renal Association-European Dialysis and Transplantation Association (ERA-EDTA): short-term Young Fellowship grant.

**Table 36:** (abstract A92).

	Patients with FO (n=108)	Patients without FO (n=1018)	p
Days in ICU, mean (SD)	19.6 (15.7)	12.8 (14.9)	<0.0001
SOFA score on day of RRT, mean (SD)	11.4 (3.7)	9.9 (3.5)	<0.0001

### A93 Fluid prescription in hospitalised patients with renal failure: evidence for a therapeutic index for volume therapy

#### L. Forni^1^, M. Scott^2^, J. Sahatjian^3^, W. Linde-Zwirble^4^, D. Hansell^5^

##### ^1^Royal Surrey County Hospital, Guildford, United Kingdom; ^2^Royal Surrey County Hospital, ICU and SPACeR research group, Guildford, United Kingdom; ^3^Cheetah Medical, Inc., Director of Clinical Operations, Newton Center, United States; ^4^Health Economics Trexin, Chief Data Scientist, Chicago, United States, ^5^Cheetah Medical, Inc., Chief Physician Executive, Newton Center, United States

###### **Correspondence:** L. Forni – Royal Surrey County Hospital, Guildford, United Kingdom

**Introduction:** AKI complicating acute illness has significant implications with volume resuscitation seen as integral to the management of AKI^1,2^. There has been much recent debate regarding overall volume balance with important treatment implications^3^.

**Objectives:** We decided to examine the relationship between fluid prescription and patient outcomes in patients with AKI

**Methods:** From the Premier database we identified a study population of 62,695 from 493 hospitals. Patients were admitted to the ICU with AKI on the first day of the hospitalisation, received at least 1 L of Day 1 fluids and survived to Day 2. A multivariate model was built to predict mortality with a case-mix adjustment model applied to the data set in order to adjust for severity of illness together with premorbid assessment.

**Results:** Mean age was 65 years, 55 % male, 75 % emergencies, 43 % diabetic and 36 % had CKD. Mean LOS: 8.7 days with 4.6 days in the ICU. Average Day 1 fluids were 3.7 L (median 3.1 L), lowest in those without pressors (3.2 L) and highest in those with MV and septic shock (5.4 L). Hospital mortality was 16.5 % for Day 1 survivors, varying from 7.8 % in those with no MV nor pressors (NMNP), to 53 % among those with MV and pressors (MVAP). Significant associations between volume of Day 1 fluids and hospital survival was seen. Overall mortality was ca.15 %,19.4 % and 29.3 % for those receiving 1-5 L of fluid, 6 L fluid and (9 + L) respectively.For NMNP cases (57 %) both actual and expected mortality rates decreased with increased fluid volume (8.3 % at 1 L, 6.6 % at 9 + L). Patients receiving pressors and MV (17 %) exhibited minimal variation in expected mortality but presented with a decrease in mortality from low to middle ranges (23.2 % at 1 L to 15.7 % at 5 L) and an increase in mortality from middle to high ranges (15.7 % at 5 L to 25.4 % at 8 L). The highest severity group exhibited no change in actual or expected mortality with fluid administration. Septic shock requiring MV (13 % of cases) had no variation in mortality across the lower groups of day 1 fluid use (1 L to 5 L). However, higher ranges of 6 + L (38 % of the group) had increased actual mortality rates (40 % at 6 L to 45 % at 9 + L), higher than predicted.

**Conclusions:** A potential for both under resuscitation and over resuscitation is observed in patients with AKI who received treatment with vasopressors in those requiring pressor and MV support a clear association existed between volume excess and outcomes emphasizing the need for a better understanding of individual fluid needs in this important population.

**References**

1. Waikar SS, Liu KD, Chertow GM. The incidence and prognostic significance of acute kidney injury. Current opinion in nephrology and hypertension. 2007;16(3):227-36.

2. KDIGO clinical practice guideline for acute kidney injury. Kidney Int Suppl. 2012;2:1-138.

3. Ostermann M, Straaten HM, Forni LG. Fluid overload and acute kidney injury: cause or consequence? Critical care. 2015;19(1):443.

**Fig. 36 (abstract A93).**
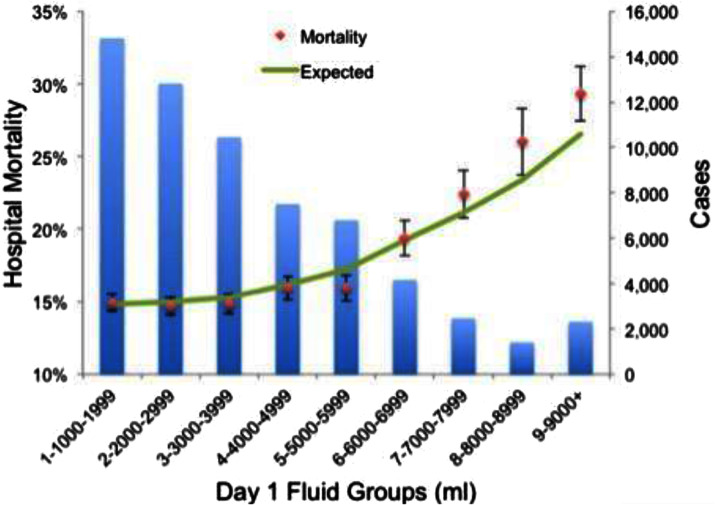
Mortality vs Fluid Groups

### A94 Acute kidney injury associated laparoscopic abdominal surgery, a prospective cohort study

#### P. Laoveeravat^1,2,3^, N. Srisawat^1,2,4^, M. Kongwibulwut^5^, S. Peerapornrattana^1,2^, N. Suwachittanont^1^, T.-O. Wirotwan^1^, P. Chatkaew^5^, P. Saeyub^5^, K. Latthaprecha^5^, K. Tiranathanagul^2^, S. Eiam-ong^2^, J.A. Kellum^3^

##### ^1^Excellence Center for Critical Care Nephrology, King Chulalongkorn Memorial Hospital, Thai Red Cross and Faculty of Medicine, Chulalongkorn University, Bangkok, Thailand; ^2^Division of Nephrology, Department of Medicine, King Chulalongkorn Memorial Hospital, Thai Red Cross and Faculty of Medicine, Chulalongkorn University, Bangkok, Thailand; ^3^Center for Critical Care Nephrology, CRISMA Center, Department of Critical Care Medicine, University of Pittsburgh School of Medicine, Pittsburgh, United States; ^4^Center for Critical Care Nephrology, CRISMA Center, Department of Critical Care Medicine, University of Pittsburgh school of Medicine, Pittsburgh, United States; ^5^Department of Anesthesiology, King Chulalongkorn Memorial Hospital, Thai Red Cross and Faculty of Medicine, Chulalongkorn University, Bangkok, Thailand

###### **Correspondence:** P. Laoveeravat – Excellence Center for Critical Care Nephrology, King Chulalongkorn Memorial Hospital, Thai Red Cross and Faculty of Medicine, Chulalongkorn University, Bangkok, Thailand

**Introduction:** Laparoscopic abdominal surgery has been widely used to reduce length of stay and complications from open abdominal surgery. During the operation, pneumoperitoneum needs to be created for better visualization of operating fields. This is followed by decreased urine output and resulted in acute kidney injury. However, we do not know the effect of pneumoperitoneum and factors inducing acute kidney injury (AKI).

**Objectives:** We aimed to 1) show the incidence of AKI in patients undergone laparoscopic abdominal surgery and 2) propose a set of risk factors associated with the development of AKI following laparoscopic abdominal surgery

**Methods:** All patients underwent laparoscopic abdominal surgery at King Chulalongkorn Memorial hospital, Bangkok, Thailand were prospectively enrolled between 2012 and 2013 (n = 64). Two were excluded due to preexisting chronic kidney disease and NSAIDs use in previous 1 week. Baseline characteristics, laboratory results, and introperative data were prospectively recorded in case record forms. Urine neutrophil gelatinase-associated lipokalin (NGAL) was measured as the surrogate marker. AKI was identified by KDIGO criteria. Characteristics were analyzed by student´s t-test and nonparametric test. Factors associated with AKI were identified using the logistic regression and the area under the receiver operating characteristics curve (AUC).

**Results:** Of the 62 patients receiving laparoscopic abdominal surgery, 12(19 %) developed postoperative AKI. The mean age, initial blood pressure, and initial glomerular filtration rate were not different between AKI and non-AKI groups. The peak serum creatinine was seen at 24 hours postoperatively. AKI patients had significantly increased urine NGAL level at 24 hours postoperatively compared to non-AKI (p = 0.01). Mean operative time, inflation time, and exposure index, defined by the product of inflation time and intra-abdominal pressure were significantly higher in AKI compared with non-AKI patients (p < 0.05 for all). Duration of intraoperative hypotension, amount of blood loss and intravenous fluid were not different between groups. By multivariate analysis, exposure index was significantly associated with postoperative AKI, with adjusted odds ratio (95 % CI) of 1.59 (1.02-2.38). AUCs of inflation time, operation time, and exposure index were 0.68, 0.67, and 0.67, respectively (p < 0.05).

**Conclusions:** Postoperative AKI can occur in patients undergone laparoscopic abdominal surgery. A larger cohort is required to confirm these findings.

**References**

1. Chiu AW, Chang LS, Birkett DH, et al. The impact of pneumoperitoneum, pneumo-retroperitoneum, and gasless laparoscopy on the systemic and renal hemodynamics. J Am Coll Surg. 1995 Nov;181(5):397-406.

**Grant acknowledgement**

None.

### A95 The incidence and outcome of fluid overload in intensive care unit patients with acute kidney injury

#### R.E. Berthelsen^1^, A. Perner^2^, A.E.K. Jensen^3^, J.U. Jensen^4^, M.H. Bestle^1^

##### ^1^Nordsjællands Hospital, Dept. of Anaesthesiology and Intensive Care, Hillerød, Denmark; ^2^Rigshospitalet, Copenhagen University Hospital, Dept. of Intensive Care, Copenhagen, Denmark; ^3^University of Copenhagen, Dept. of Biostatistics, Copenhagen, Denmark; ^4^Rigshospitalet, Copenhagen University Hospital, CHIP & PERSIMUNE, Dept. of Infectious Diseases, Copenhagen, Denmark

###### **Correspondence:** R.E. Berthelsen – Nordsjællands Hospital, Dept. of Anaesthesiology and Intensive Care, Hillerød, Denmark

**Introduction:** The incidence of acute kidney injury (AKI) in critically ill patients approximates 50 % with an odds ratio for death as high as 7.18 [1]. Patients with AKI often have impaired excretion of salt and water[2]. On the other hand, intravenous fluids are part of the management of AKI, which increases the risk of fluid overload in these patients.

**Objective:** We examined the association between fluid overload and outcome in ICU patients with AKI.

**Method:** Retrospective cohort study of adult ICU-patients from two university hospitals in Denmark admitted between Jan 1st 2012 and Dec 31st 2013. All cases of AKI were identified according to the creatinine criteria of the KDIGO definition. Fluid data was missing in 13 patients (1.5 %), and these were excluded from the analysis.

The association between cumulative fluid balance and the risk of death/renal recovery at day 28 was estimated with joint modeling techniques [3]. The cumulative fluid balance was dichotomized with cutoffs at 5 % and 10 % of admission body weight at any day during the first five days of admission. The longitudinal submodel was fitted using mixed effects logistic regression, and the survival submodel was a Cox model adjusted for age, gender, severity of disease (SAPS II, KDIGO grade) and use of life support (norepinephrine, mechanical ventilation and dialysis).

The study was approved by the national board of health, who waived the need for consent.

**Fig. 37 (abstract A95).**
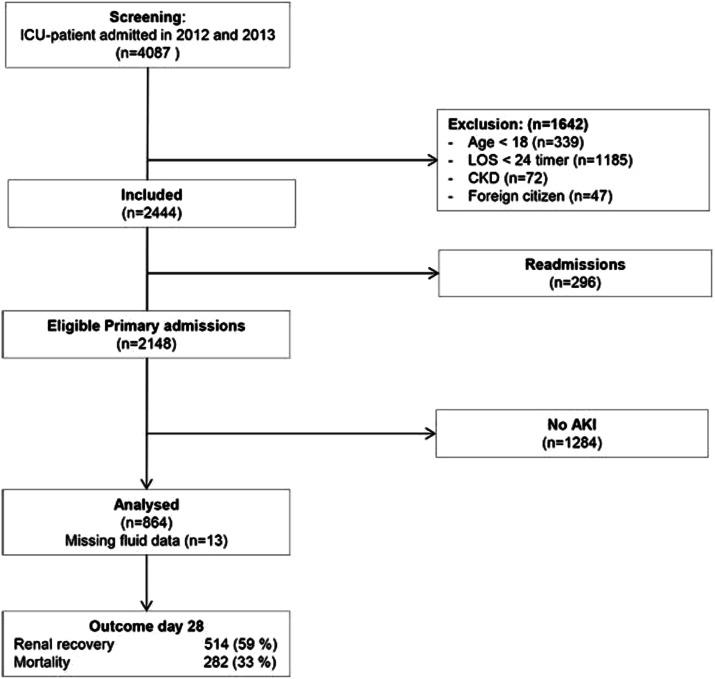
Study flowchart

**Fig. 38 (abstract A95).**
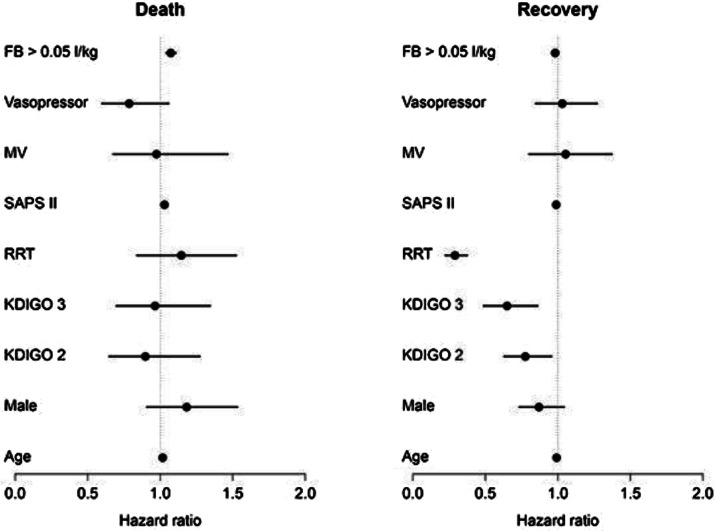
FB = fluid balance, MV = mechanical ventilation, RRT = renal replacement therapy

**Results:** We screened 4087 patients and identified 864 with AKI (Fig. 37) of whom 461 and 255 developed fluid overload above respectively 5 % and 10 % of bodywieght (BW) during their first 5 days in ICU (Fig. 38). At day 28, 514 of the AKI patients had renal recovery and 282 had died (Table 37).

Fluid overload >10 % BW at any day during the first 5 days in ICU was associated with a hazard ratio (HR) for death of 1.08 (p < 0.001); for > 5 % BW fluid overload the HR for death was 1.07 (p < 0.001). In contrast, there was no association between fluid accumulation and renal recovery.

**Conclusion:** In our cohort of 864 patients with AKI, 255 developed fluid overload (10 % BW) during the first 5 days in ICU. Fluid overload, even at 5 % BW, was associated with increased mortality after adjusting for risk factors, but fluid overload was not associated with renal recovery.

**References**

[1] Hoste EAJ, Bagshaw SM et al. Epidemiology of acute kidney injury in critically ill patients: the multinational AKI-EPI study. Intensive Care Med 2015;41:1411-23.

[2] Bellomo R, Kellum J a, Ronco C. Acute kidney injury. Lancet 2012;380:756-66.

[3] Rizopoulos D. Joint models for longitudinal and time-to-event data. 1st ed. London: Chapman & Hall/CRC Biostatistics Series; 2012.

**Grant acknowledgement**

This study was funded by the Department of Anaesthesiology, Nordsjællands Hospital.

**Table 37 (abstract A95).** Patient characteristics

	All patients (N = 864)	Fluid Balance > 5% BW (N = 461)	Fluid Balance < 5% BW (N = 367)
Age, median (IQR), years	68 (59-76)	68 (59-76)	68 (58-77)
Male gender, N(%)	528 (61 %)	267 (58 %)	237 (65 %)
SAPSS II, median (IQR)	50 (39-63)	54 (43-66)	46 (36-59)
KDIGO score ≥ 2, N(%)	405 (47%)	237 (51 %)	155 (42 %)
Mechanical ventilation, N(%)	728 (84 %)	401 (87 %)	300 (82 %)
Renal replacement therapy, N(%)	277 (32 %)	171 (37 %)	97 (26 %)
Vasopressor use, N(%)	593 (69 %)	347 (75 %)	232 (63 %)
Renal recovery, N (%)	514 (60 %)	247 (54 %)	246 (67 %)
Mortality, N(%)	282 (33 %)	192 (42 %)	85 (23 %)

### A96 Staging of cardiorenal syndrome for outcome prediction in pediatric acute decompensated heart failure

#### D.J. Gebhard^1^, J. Price^2^, C.E. Kennedy^3^, A. Akcan-Arikan^3,4^

##### ^1^University of Texas Health Science Center San Antonio, Pediatric Critical Care Medicine, San Antonio, United States; ^2^Baylor College of Medicine, Pediatric Cardiology, Houston, United States; ^3^Baylor College of Medicine, Pediatric Critical Care Medicine, Houston, United States; ^4^Baylor College of Medicine, Pediatric Nephrology, Houston, United States

###### **Correspondence:** A. Akcan-Arikan – Baylor College of Medicine, Pediatric Critical Care Medicine, Houston, United States

**Introduction:** Subtle worsening of renal function is associated with adverse outcomes in pediatric patients (pts) with heart failure. Cardiorenal syndrome (CRS) is a recently coined term underscoring the co-existence of cardiac and renal dysfunction and stresses the bidirectional nature of the heart-kidney interactions. While well defined in adult populations, available pediatric data is scarce. Acute kidney injury (AKI) consensus definitions could offer a standardized approach to CRS definition and could help stratify patients.

**Objectives:** To apply three consensus definitions of AKI previously validated in heterogeneous pediatric populations (pediatric Risk, Injury, Failure (pRIFLE), Acute Kidney Injury Network (AKIN), and Kidney Disease Improving Global Outcomes (KDIGO) to assess CRS burden and association with outcomes

**Methods:** Retrospective study of all pts admitted with acute decompensated heart failure (ADHF).Failure of medical therapy was defined as mechanical support, transplantation or death. CRS was defined as admission with ADHF and concomitant AKI according to KDIGO, AKIN or pRIFLE creatinine criteria.

**Results:** 75 pts (47 % male, 5.5 years (IQR 0.8-15.4)) were included. Hospital LOS was 48.9 ± 59.1 days, 33 pts (44 %) had a prior diagnosis of heart failure, 39 (52 %) did not have a baseline creatinine. FMT happened in 25 pts(33 %), mortality was 13 % (10/75). AKIN and KDIGO were identical in diagnosing and staging CRS so only KDIGO data are presented. CRS on admission was present in 63 %(47/75) by pRIFLE vs 27 % (20/75) by KDIGO (p < 0.001). pRIFLE identified 27 additional pts with CRS on admission compared to KDIGO (21 R, 5 I, 1 F). CRS on admission either by pRIFLE or KDIGO did not have good prediction for FMT (AUC 0.58 vs 0.64, respectively), similar to any stage of CRS reached during admission by either definition (AUC 0.58 any pRIFLE stage vs 0.61 any KDIGO stage), whereas peak CRS stage reached during admission had good predictive ability for FMT for both (AUC 0.79 for both). 35 and 24 pts still fulfilled CRS criteria by pRIFLE and KDIGO, respectively, at discharge, Of the 47 pRIFLE and 20 KDIGO admission CRS pts, 25 (53 %) and 15 (75 %) had persistent CRS at discharge. KDIGO stage of CRS on discharge was associated with readmission (p < 0.001) but pRIFLE stage was not (p = 0.25)(AUC 0.63 for KDIGO vs 0.5 for pRIFLE for readmission).

**Conclusions:** CRS was very common in pediatric pts with ADH, persisted at discharge, and renal function at discharge predicted readmission. Peak KDIGO stage predicted composite outcome of death, transplant, or mechanical support; discharge KDIGO stage predicted readmission. KDIGO staging outperformed pRIFLE staging for outcome prediction in pediatric CRS. This finding needs to be validated in a prospective multicenter study.

### A97 Early aggressive fluid therapy and kidney: consequences in sepsis survivors. An experimental study

#### A.M.A. Liberatore^1^, R.B. Souza^2^, A.M.C.R.P.F. Martins^3^, J.C.F. Vieira^1^, Y.R. Kang^1^, M.N. Nakamae^1^, I.H.J. Koh^1^

##### ^1^Federal University of São Paulo, Surgery, São Paulo, Brazil; ^2^Federal University of São Paulo, Morphology and Genetics, São Paulo, Brazil; ^3^Biological Institute of São Paulo, Sao Paulo, Brazil

###### **Correspondence:** A.M.A. Liberatore – Federal University of São Paulo, Surgery, São Paulo, Brazil

**Introduction:** Microcirculatory dysfunction is an important triggering event of organ dysfunction in sepsis, and the fluid therapy is essential for improvement of the hemodynamic, but the ideal fluid strategy in sepsis has not been developed yet. Aggressive-fluid therapy is controversial and the consequences for the kidney dysfunction are little known. This study investigated the effect of an early phase aggressive-fluid therapy at the kidney microcirculation and tubular structures.

**Methods:** Adult Wistar rats (200 g) were submitted to sepsis {iv. 2 mL *E. coli* 10^8^ CFU/mL (S8), DL60 in 26 hours}, or sepsis and Ringer Lactate infusion (30 mL/kg/20 min), 30 minutes after the sepsis challenge. Under general anesthesia, the microcirculation of the renal cortical area was monitored by Sidestream Dark Field Imaging (SDF) video-microscopy, at 6 hours (T6h - n = 3/group) and 30 days (T30d - n = 3/group) after sepsis. The tissue samples were evaluated by histology (T6h,T30d) by HE and PAS staining.

**Fig. 39:**
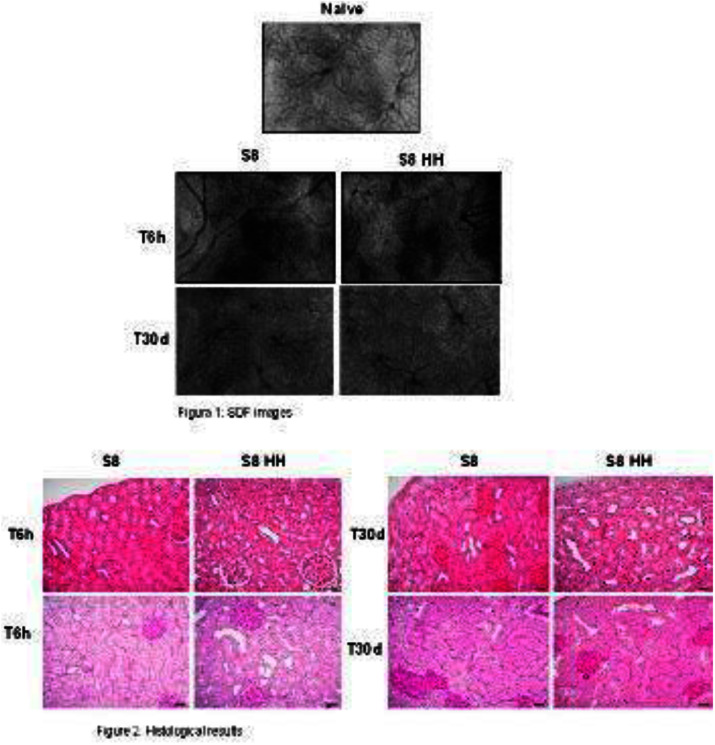
(abstract A97).

**Results and conclusions:** SDF at T6h showed broadly distributed microcirculation and tissue dysfunction at both groups (Fig. 39). The outlining of tubules became blurred by their enlargement, with compression of tubular lumen and of the peritubular microvessels, suggesting an obstructive phenomenon by cellular edema. Histology. After 6 hours of sepsis, animals treated with aggressive fluid infusionshowed less peritubular congestion, preserved mesangial space and lower areas with hyaline degenerations and better preservation of the tubular lumen compared to sepsis without aggressive overhydrating. PAS staining showed a very slight hyaline degeneration, better preservation of tissue architecture, lower occurrence of cellular death, suggesting that the aggressive fluid therapy in the early stage of sepsis minimizes the severity of vascular and tissue injury in the kidney. The live animals treated with the aggressive fluid showed less peritubularmicrovesselscongestion compared to S8, better preservation of the mesangial space, minor hyaline degeneration, and less cell death in deeper regions of the cortexafter a month of recovery. However, the general appearance showing a kidney limitation for venous blood drainage. Clearly, the aggressive-fluid therapy attenuates renal damage compared to S8. The results suggest that animals treated with aggressive fluid therapy, at the early stage of sepsis, have better conditions to respond against new harmful challenges to the kidney. Besides, the recover process after sepsis seems to be partial, justifying the occurrence of the post-sepsis syndrome.

**Grant acknowledgement**

FAPESP 2011/20401-4.

### A98 Non invasive adjustment of fluid status in critically ill patients on renal replacement therapy. Role of electrical cardiometry

#### K. Hamed, M. M Khaled, R. Aly Soliman, M. Sherif Mokhtar

##### Faculty of Medicine, Cairo University, Critical Care Medicine, Cairo, Egypt

###### **Correspondence:** K. Hamed – Faculty of Medicine, Cairo University, Critical Care Medicine, Cairo, Egypt

**Introduction:** Electrical Cardiometry allows measurement of fluid status using thoracic fluid content(TFC),cardiac output,cardiac index,systemic vascular resistance index which could be ideal noninvasive hemodynamic monitoring for patients undergoing hemodialysis(HD.

**Objectives:** Investigating relation between changes in TFC and amount of fluid removal during HD session and to monitor hemodynamic parametersto avoid episodes of hemodynamic compromise during HD session.

**Methods:** Thirty critically ill patients on HD were enrolled.Clinical assessment of volume overload and hemodynamics(BP,MAP,CVP),monitored by Electrical Cardiometry ICON® before HD and all through sessions.

**Results:** Out of studied patients males represented 46.7%n = 14with mean age 48 ± 16 years.There was positive correlation between UFvolume and TFC (r = 0.410, P = 0.025). Out of the 30pts studied 18pts60%were hemodynamically stable vs 12pts40% had hypotension represented non responders group and had lower TFC compared to hemodunamically stable group (26.45kohm-1 vs 37.8kohm-1) P value 0.004 indicating that they were hypovolemic. Out of the 30pts studied 18pts60%weren´t congested vs 12pts40%were remained persistently congested after accomplishing HD session with significantly higher TFC when compared to those get red off congestion (43.14 ± 9.9kohm-1 vs 25.44 ± 5.5kohm-1) P value 0.0001 indicating that they were still hypervolemic. Using analysis of ROC curve TFC at 25.34kohm-1 was significantly predictor of hypotension with P value0.002, AUC83.4 %, sensitivity67%, specificity100%. Also TFC cutoff value predicting persistent congestion was 37.02kohm-1 with P value0.0001, AUC95.8 %, sensitivity83%, specificity100%.

**Conclusions:** Electrical Cardiometry is evolving noninvasive tool for adjusting fluid status of critically ill patient on RRT using thoracic fluid content as indicator of fluid status that could be used to avoid hemodynamic instability and persistent volume overload and congestion during and after HD session.

**References**

1. Palmer B.Fand Henrich W.L,Recent advances in the prevention and management of intradialytic hypotension.J Am Soc Nephrol,2008.19(1): p.8-11

2. Rosner M.Hand Ronco C,Techniques for the assessment of volume status in patients with end stage renal disease.Semin Dial,2014.27(6):p.538-41

3. Malik V,Subramanian A,et al,Correlation of Electric Cardiometry

and Continuous Thermodilution Cardiac Output Monitoring Systems.World Journal

of Cardiovascular Surgery,2014.4:p.101-108

4. Wynne J.L,Ovadje L.O,et al,Impedance cardiography:a potential monitor for hemodialysis.J Surg Res,2006.133(1):p.55-60

5. Osypka M.J,An introduction to Electrical cardiometry c.a.O. company, Editor 2009

6. De Nicola A and Sucre M.J,Impedance cardiography in the estimation of hemodynamic and fluid status of coma patients during continuous venovenous hemodiafiltration Critical Care,2009.13(1):p.202

7. van de Water J.M,Mount B.E,et al,TFC(thoracic fluid content):a new parameter for assessment of changes in chest fluid volume.Am Surg,2005.71(1):p.81-6

### A99 Design of a protocol for the estimation of functional renal reserve in critical care patients

#### G. Seller-Pérez^1^, D. Arias-Verdú^1^, E. Llopar-Valdor^2^, I. De-Diós-Chacón^3^, G. Quesada-García^1^, M.E. Herrera-Gutierrez^1^

##### ^1^Complejo Universitario Carlos Haya, Málaga, Spain; ^2^Hospital Parc Tauli, Sabadell, Spain, ^3^Hospital Son Llatzer, Palma de Mallorca, Spain

###### **Correspondence:** D. Arias-Verdú – Complejo Universitario Carlos Haya, Málaga, Spain

**Introduction:** Acute Kidney Injury (AKI) is believed to carry a good prognosis but recent reports have raised concern about long-term outcome. Assessment of renal functional reserve after AKI can be of aid for ascertaining recovery of kidney function.

**Objective:** To develop a method for the estimation of renal reserve in the critical care setting.

**Methods:** Exploratory study. We selected 8 patients (4 men/women) between 20 to 50 years, without known previous renal disease who did not develop AKI during ICU stay. Patients were not receiving drugs that could interfere with renal function, were stable and already recovered from their initial problem, but still with a bladder catheter, a IV line and a nasogastric tube for enteral feeding. After administration of a load of 20 gr of proteins by enteral route, a creatinine clearance (CrCl) was calculated for each of the next 6 hours, with a timely collection of urine and a sample of serum creatinine at the end of every period. For the analysis we performed u-Mann-Whitney and Kruskal-Wallis non-parametric test. Data as mean (mean error standard) or median (percentiles 25-75).

**Fig. 40:**
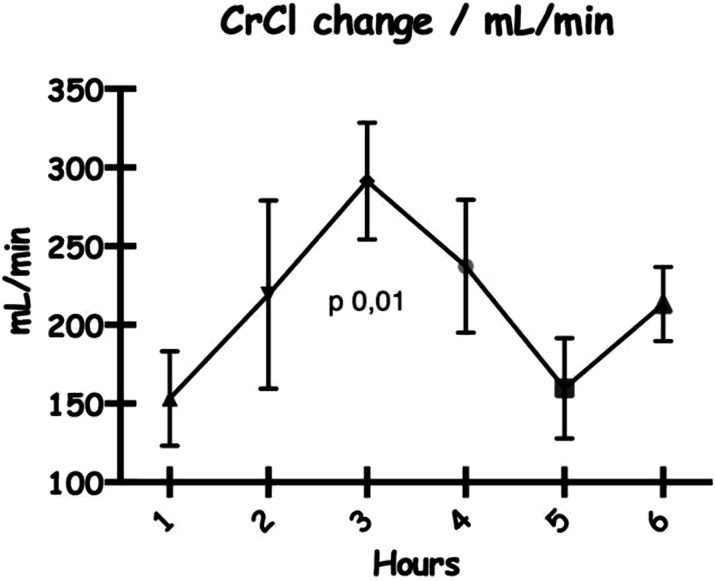
(abstract A99).

**Results:** Age 41.13 (3.2) years, 50 % men and base CrCl 163 (19.51) mL/min. Median percentage of change between base and maximum CrCl was 123.5 % (78.2-143.2) and because a pick was detected between 3 and four hours after protein load we analysed these two hours together, finding a median change of 80.7 % (69.95-144). Changes between the first and third hours were significant either for absolute values (p 0.023) or % (p 0.031). Hourly changes in CrCl are presented in Fig. 40

**Conclusions:** A protein load by enteral route is followed by an early rise in creatinine clearance. The profile of this response lets us propose a creatinine clearance 3-4 hours after a load of 20 gr of proteins as a quick and easy way to estimate renal reserve. Our next goal should be to define the profile of response for different kind of patients and degrees of renal dysfunction.

### A100 Nephrology follow-up of patients with acute kidney injury requiring renal replacement therapy in critical care

#### R. Hafes^1^, G. Carroll^2^, P. Doherty^2^, C. Wright^2^

##### ^1^University of Glasgow, Glasgow, United Kingdom; ^2^Queen Elizabeth Hospital, Critical Care, Glasgow, United Kingdom

###### **Correspondence:** R. Hafes – University of Glasgow, Glasgow, United Kingdom

**Introduction:** Evidence has established that there is strong link with Acute Kidney Injury (AKI) and progression to chronic kidney disease (CKD) and end stage renal failure (ESRF) [1]. After AKI, 9 % have permenant loss of kidney function and 9-13 % are dialysis dependent post hospital discharge[2]. Renal followup is recommended for patients who have had AKI whilst critically ill [3].

**Objectives:** To assess whether patients that received renal replacement therapy (RRT) in ICU had a nephrology follow up, and determine if there was a need for the service.To assess if there were any difference in mortality between the groups.

**Methods:** We performed a retrospective longitudinal cohort study analysis of all patients that received RRT after an AKI in the Greater Glasgow and Clyde Hospitals over one year. Renal function at 3-6 months pre-admission and post discharge were examined by comparing urea, creatinine and eGFR values. Nephrology follow up status was also investigated. We excluded patients that had renal baselines out with the 3-6 month period.Paired t-test analysis was used to analyze pre-admission vs post discharge renal baselines in normal eGFR patients

**Results:** We sampled 130 admissions with 68 patients surviving discharge from ICU. 28 patients (41 %) had a nephrology follow-up. The remaining 49 patients (59 %) were not followed up. 39 patients (57 %) had an abnormal eGFR at 3-6 months, and of these 24 (61 %) were followed up. 4 patients (6 %) died in each group .

**Conclusions:** Renal follow up rate after RRT in critical care is low despite 37 % having an abnormal eGFR. An efficient followup service is required.

**References**

1. Uchino S, Kellum JA, Bellomo R, et al. Acute renal failure in critically ill patients: a multinational, multicenter study. JAMA 2005; 294:813-8.3.

2. Acute kidney injury leading to chronic kidney disease and long-term outcomes of acute kidney injury: the best opportunity to mitigate acute kidney injury? Contrib Nephrol 2011; 174:182-90.7.

3. Kirwan CJ, Blunden MJ, Dobbie H, James A, Nedungadi A, Prowle JR. Critically ill patients requiring acute renal replacement therapy are at an increased risk of long-term renal dysfunction, but rarely receive specialist nephrology follow-up. Nephron. 2015; 129(3): 164-70.

### A101 Chronic kidney disease by stage secondary to diabetes

#### I.G. Guerra Vera

##### Centro Universitario de La Costa, Puerto Vallarta Jalisco, Mexico

**Introduction** The risk of chronic kidney disease increases with the time of evolution of type 2 diabetes and chronic metabolic control. In people over 40 years occur a progressive loss of glomerular filtration rate corresponding to 1 mL per year. This is associated with progressive deterioration of renal tissue replacement by fibrous tissue, which involves progressive glomeruroesclerosis, tubulointerstitial fibrosis and nephrosclerosis.

**Objectives** To determine the evolution time of diabetes and prevalence for stages on chronic kidney disease. *Material and method*: A cross-sectional and descriptive study was done on 150 patients diabetic type 2. There were included all of them that have more than 5 years of evolution on the diabetes type 2, the sample was calculated with the averages formula for finite population and the selection was simple random. Sociodemographic variables and health variables were studied, the stage of chronic kidney disease were estimated by the Cockcroft-Gault equation. The statistic analysis included averages, percentages and confidence intervals.

**Methods** A cross- sectional, descriptive, observational, retrospective study was conducted which included adult patients that were 50 years and older, and had 10 year diabetes mellitus type 2 diagnosis, without complications mentioned in the Nephrology Unit in Hospital General de Mexico, Mexico City.

**Results** Regarding the study population, 57 % are female, the average age was 62.12 years and mean glucose was 165.23 mg / dL. The time evolution of diabetes 2 patients in stage 5 was 20.05 years and in patients with stage 1 was 11.05 years. The average creatinine clearance in stage 2 was 75.10 mL / min and in stage 5 10.33 mL / min. 13 % of the population was in stage 4 and a similar percentage (15 %) in stage 5. The time evolution of stage 1 to stage 5 was 10.10 years and stage 3 to 4, 1.5 years.

**Conclusions** Chronic kidney disease is a public health problem that affects health systems around the world. Today his studio is preferably focused on the population undergoing dialysis treatment in its various forms which lies in stage 5; However, a comprehensive approach to chronic kidney disease in all its stages is necessary to have information about the condition; hence the importance of this study, in which the time evolution of diabetes and population analyzed by stage of chronic kidney disease. The diabetic patient with chronic kidney disease is not flattering; it is distinguished by short evolution times between the stages and high population percentage on stages 4 and 5.

**Reference(s)**

Wild S, Roglic G, Green A, Sicree R, King H. Globla prevalence of Diabetes: estimates for the year 2000 and projections for 2030. Diabetes care 2004;27:1047-1053.

Eknoyan G, LAmeire N, EckardKU, Kasiske BL, et al. KDIGO 2012, clinical practice guideline for the evaluation and Management of Chronic Kidney Disease. 2013;3.

## Fluid balance, therapies and outcomes in aki

### A102 Outcomes of patients requiring renal replacement therapy in intensive care: a ten year retrospective study

#### M. Ralston, L. Gemmell, A. MacKay, E. Black, C. Wright, R.I. Docking, R. Appleton

##### Queen Elizabeth University Hospital, Anaesthetics and Intensive Care, Glasgow, United Kingdom

###### **Correspondence:** M. Ralston – Queen Elizabeth University Hospital, Anaesthetics and Intensive Care, Glasgow, United Kingdom

**Introduction** Approximately 4 % of patients in Intensive Care Units (ICU´s) require renal replacement therapy (RRT) during their admission, though this figure ranges from 1-25 % depending on the particular hospital (1). Previous studies suggest that patients requiring RRT in ICU have a 60 % in-hospital mortality rate, compared with an overall mortality rate of 19 % for patients admitted to ICU.

(1.2).

**Objectives** The aim of the study was to compare demographic and physiological characteristics of patients requiring RRT in ICU with those who did not require it, and to investigate the impact of receiving RRT on length of unit stay and mortality on ultimate hospital discharge.

**Methods** The study is a multi-centre retrospective observational cohort study. It uses data from January 1st 2005 to December 31st 2014 from three teaching hospital intensive care units with 18 combined beds and an associated tertiary referral renal service in Glasgow. The data used was collected prospectively from the Wardwatcher service. Demographic details, severity scores, physiological parameters and information on length of unit stay and in-hospital mortality was gathered. Standardised Mortality Ratios (SMRs) were calculated using the APACHE II predicted mortality scores as the denominator.

**Results** Data for a total of 10549 patients was collected, of whom 13.9% (1471) received RRT during their admission. The mean duration of RRT was 5.0 +/- 0.2days. Standardised mortality ratios between the two groups is comparable at 1.04 for the RRT group, and 10.6 for the non RRT group. The results for the two groups are shown below.

**Conclusions** Patients receiving RRT had a mortality rate on ultimate hospital discharge of 52.6 %, roughly in line with previous studies, and nearly twice the rate in those not requiring RRT. (1). These patient also stayed in ICU for over twice as many days as those not requiring RRT. It is interesting to note, however that the SMR is actually marginally lower for those receiving RRT than not. Patients who require RRT at some point during their ICU stay also have significantly more deranged physiology during their first 24hours of admission, as evidenced by the urine output, potassium, urea and creatinine values for the two groups.

**Reference(s)**

1. Uchino S, Kellum JA. Acute renal failure in critically ill patients: a multinational, multicenter study. JAMA:2005:294(7): 813-8.

2. Scottish Intensive Care Society Audit group. Audit of Critical Care in Scotland 2014: reporting on 2013. Edinburgh, Uk: NHS National Services Scotland; 2014.

**Table 38:** (abstract A102).

	RRT (n=1471)	No RRT (n=9078)	p value
Age (years)	59.3 ± 0.8	56.1 ± 0.4	<0.001
Previous chronic RRT (%)	10	1.3	<0.001
APACHE II	24.7 ± 0.5	15.7 ± 0.2	<0.001
Mortality on discharge (%)	52.6	28.2	<0.001
Urine output 1st 24hrs (mls)	625 ± 43.4	1646 ± 25.7	<0.001
Highest K+ in 1st 24hrs (mmol/l)	5.0 ± 0.1	4.3 ± 0.1	<0.001
Highest Urea in 1st 24hrs (mmol/l)	19.7 ± 0.7	9.0 ± 0.2	<0.001
Highest creatinine 1st 24hrs (mmol/l)	337 ± 12	118 ± 2	<0.001
Unit stay (days)	8.6 ± 0.5	3.8 ± 0.1	<0.001

### A103 Outcomes of patients requiring renal replacement therapy in intensive care: a 10 year retrospective study

#### M.R. Ralston, L. Gemmell, R. Appleton, C. Wright, R.I. Docking, E. Black, A. Mackay

##### Queen Elizabeth University Hospital, Intensive Care Unit, Glasgow, United Kingdom

###### **Correspondence:** M.R. Ralston – Queen Elizabeth University Hospital, Intensive Care Unit, Glasgow, United Kingdom

**Introduction** Approximately 4 % of patients in intensive care units (ICUs) require renal replacement therapy (RRT) during their admission, though this figure ranges from 1-25 % depending on the particular hospital (1). Studies have suggested that patients requiring RRT in ICU have roughly a 60 % in-hospital mortality rate, compared with an overall mortality rate of 19 % for patients admitted to ICU (1,2).

**Objectives** The aim of the study was to compare demographic and physiological characteristics of patients requiring RRT in ICU with those who did not require it, and to investigate the impact of receiving RRT on length of unit stay and mortality on ultimate hospital discharge.

**Methods** The study is a multi-centre retrospective observational cohort study. It uses data from January 1, 2005 to December 31, 2014 for three intensive care units (ICUs) based in teaching hospitals, with 18 beds combined, and an associated tertiary renal service. The data used was collected prospectively for the Wardwatcher™ service. Demographic details, severity scores, physiological parameters and information on length of unit stay and in-hospital mortality were gathered. Standardised Mortality Ratios (SMRs) were calculated using the APACHE II predicted mortality scores as the denominator.

**Results** Data for a total of 10549 patients was collected, of whom 13.9 % (1471) received RRT during their admission. The mean duration of RRT was 5.0 ± 0.2 days. The results for the two groups are shown below:

**Conclusions** Patients receiving RRT had a mortality rate on ultimate hospital discharge of 52.6 %, roughly in line with previous studies, and nearly twice the rate in those not requiring RRT (1). These patients also stayed in ICU for over twice as many days as those not requiring RRT. It is interesting to note, however, that the SMR is actually lower for those receiving RRT than not. Patients who require RRT at some point during their ICU stay also have significantly more deranged physiology during their first 24 hours of admission, as evidenced by the urine output, potassium, urea and creatinine values for the two groups.

**References**

1. Uchino S, Kellum JA, Bellomo R, Doig GS, Morimatsu H, Morgera S, et al. Acute renal failure in critically ill patients: a multinational, multicenter study. JAMA. 2005 Aug 17;294(7):813-8.

2. Scottish Intensive Care Society Audit Group. Audit of Critical Care in Scotland 2014: Reporting on 2013. Edinburgh, UK: NHS National Services Scotland; 2014.

**Table 39 (abstract A103).** Demographics and physiology

	Received RRT	Did not receive RRT	P-value
Male gender	60.8%	58.4%	0.002
Age (years)	59.3±0.8	56.1±0.4	<0.001
Previous chronic RRT (%)	10	1.3	<0.001
APACHE II score	24.7±0.5	15.7±0.2	<0.001
APACHE II predicted mortality (%)	50.6±1.3	26.0±0.5	<0.001
Urine output in 1st 24h (ml)	625±43.4	1646±25.7	<0.001
Highest potassium in 1st 24h (mmol/l)	5.0±0.1	4.3±0.1	<0.001
Highest urea 1st in 24h (mmol/l)	19.7±0.7	9.0±0.2	<0.001
Highest creatinine in 1st 24h	337±12	118±2	<0.001

**Table 40 (abstract A103).** Outcomes

	Received RRT	Did not receive RRT	P-value
Unit stay (days)	8.6±0.5	3.8±0.1	<0.001
Mortality on ultimate hospital discharge (%)	52.6	28.2	<0.001
SMR	1.04	1.08	

### A104 Renal resistive index in critically ill patients with and without shock: a cross-sectional study

#### S. Rozemeijer, J.L.G. Haitsma Mulier, J.G. Röttgering, P.W.G. Elbers, A.M.E. Spoelstra-de Man, P.R. Tuinman, M.C. de Waard, H.M. Oudemans-van Straaten

##### VU University Medical Center Amsterdam, Department of Intensive Care, Adults, Amsterdam, Netherlands

###### **Correspondence:** S. Rozemeijer – VU University Medical Center Amsterdam, Department of Intensive Care, Adults, Amsterdam, Netherlands

**Introduction** Acute Kidney Injury (AKI) is a severe complication of shock. Pathophysiological pathways include renal vasoconstriction and endothelial damage to microvessels, thereby impairing micro- and macrovascular flow. Microvascular flow can be measured by sublingual Sidestream Dark Field imaging. Renal macrocirculation can be assessed with Renal Resistive Index (RRI), (*peak systolic flow velocity* - *end diastolic flow velocity)*/ *peak systolic velocity*, using Doppler ultrasound, obtained from the intrarenal arcuate or interlobar arteries. High RRI is a predictor of persistent AKI (1). Whether RRI reflects the systemic circulation or renal microcirculation is not well known.

**Objectives** To determine whether RRI is elevated in patients with shock and to relate RRI to concomitant markers of the systemic circulation, the sublingual microcirculation, hydration state and renal function.

**Methods** We performed a prospective observational cohort study in critically ill patients admitted to the ICU between August 2015 and February 2016. Patients with shock and patients without shock were included < 24-h after ICU admission. Shock was defined as persistent hypotension or low cardiac index (<2 L/min) despite adequate fluid resuscitation and the need of vasopressors. Deferred consent was obtained. At inclusion, three study measurements were performed: RRI, SDF and Bioelectral Impedance Analysis (BIA) to assess fluid status (resistance) and membrane integrity (reactance). Uni- and multivariate analyses were computed to determine the relation between potential determinants and the RRI.

**Fig. 41 (abstract A104).**
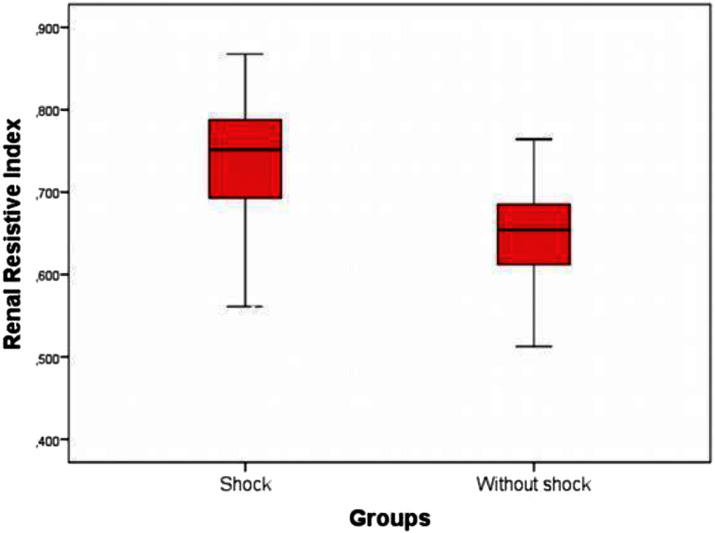
Boxplot of the distribution of the RRI

**Results** Forty patients with shock and 52 without shock were included. Patients with eGFR < 30 mL/min were excluded. Mean age was 69 (60-76) vs. 67 (59-76) yrs. and APACHE III score was 81 (63-107) vs. 57 (45-70) (p < 0.001). Shock patients had a higher RRI than patients without shock (median, 0.751 (0.692-0.788) vs. 0.654 (0.610-0.686); p < 0.001), (Fig. 41). On univariate analysis, high age, APACHE III score, vasopressor support, pulse pressure index (PPI: (systolic-diastolic)/systolic blood pressure), central venous pressure and positive fluid balance, and low mean arterial pressure (MAP), reactance/m and creatinine clearance were the markers most significantly associated with high RRI (p < 0.01). Markers of the microcirculation were not. On multivariate analysis, vasopressor support, higher PPI, lower MAP and lower Xc/m remained as independent determinants of RRI (n = 89, Adj. R^2^ = 0.472).

**Conclusions** Critically ill patients with shock have a higher RRI than patients without shock. High RRI was associated with renal dysfunction on the one hand and a disturbed systemic circulation (vasopressor support, high PPI and low MAP) and poor cellular membrane resistance on the other, but not with markers of microcirculation. These findings support the concept that shock-induced AKI is associated with renal vasoconstriction and cellular damage.

**Reference**

1. Darmon, ICM. 2011;37:68.

## Sepsis, epidemiology and outcome

### A105 "Surviving sepsis campaign": faisability and impact in a university hospital of Sub-Saharan region

#### N. Mejeni^1^, J. Nsiala^1,2^, A. Kilembe^1^, P. Akilimali^3^

##### ^1^University of Kinshasa, Anaesthesiology and Intensive Care, Kinshasa, The Democratic Republic of the Congo, ^2^Clinic Caron, Paris, France, ^3^University of Kinshasa, Public Heath, Kinshasa, The Democratic Republic of the Congo

###### **Correspondence:** J. Nsiala – University of Kinshasa, Anaesthesiology and Intensive Care, Kinshasa, The Democratic Republic of the Congo

**Introduction** Severe sepsis state constitute a worlwide public health problem. (1,2).Thus SSC has edicted recommendations to improve the management of this pathology.(3)But some authors have expressed reserves on the implementation of these recommendations in low income countries. (4)

**Objectives** To evaluate the faisability and the impact of the recommendations of SSC on adults in septic shock in University Hospital of Kinshasa.

**Methods** We conducted a quasi-experimental prospective study of twelve months from 1^st^ February 2014 to 28 february 2015 .This study was divided into 2 phases of 6 months each, before the protocol or the pre-protocol phase and after the protocol or the post-protocol phase. Adults (over 16 years old) with septic shock within this period were included. Pre-protocol group were treated as usual. After establishing a local protocol based on the SSC recommendations, post-protocol group received during the earlier 6 hours intravenous fluids,vasopressors, blood transfusion and large broad spectrum antibiotics. The next 24 hours, was devoted to organs´ failure management. Our 2 endpoints were the compliance according to the SSC recommendations and the mortality between both groups.

**Results** 72 patients were included. 33 during the first phase and 39 in the second phase. Patients' characteristics were similar in both group. The infection site responsible of septic shock was mainly pulmonary (24 %) , cutaneous (21 %), urinary (19 %) and abdominal (13 %). Post-protocol group received more intravenously fluids (+1229 ml); catecholamines (+20 %) as well as blood transfusion (X3) than those of the pre-protocol group. They were also more likely to achieve the target mean arterial pressure (36.6 versus 82.1 %) and to receive appropriate antibiotics (0 versus 12.8 %). The compliance to SSC bundles was significantly improved, passing from 0 % before protocol to 50 % after protocol. We also observed a significant decrease of the mortality, of 17 % in 6 months.

**Conclusions** SSC recommendations can be apply in a country of subsaharan Africa with a significant reduction of mortality.

**References**

1. Alberti C, Brun-Buisson C, Burchardi H, and al (2002)Epidemiology of sepsis and infection in ICU patients from an international multicentre cohort study. Intensive Care Med 28:108-121

2. Angus DC, Linde-Zwirble WT, Lidicker J, Clermont G, and al: Epidemiology of severe sepsis in the United States: analysis of incidence, outcome, and associated costs of care. Crit Care Med 2001, 29:1303-1310.

3. Dellinger RP, Levy MM, Rhodes A,and al. Surviving sepsis campaign: international guidelines for management of severe sepsis and septic shock: 2012. Crit Care Med. 2013 Feb; 41(2):580-637.

4. Baelani L,Jochberger S,Laimer T and al: Availibilityof critical care resources to treat patients with severe sepsis and septic shock in Africa:a self-reported,continent wide survey of anaesthesia providers.Crit Care.2011,15(1):R10

### A106 Venovenous extracorporeal membrane oxygenation device-related infections and colonizations

#### G. Thomas

##### Hôpital Nord, Reanimation, Marseille, France

**Introduction** Venovenous (VV) extracorporeal membrane oxygenation (ECMO) has become a widely accepted treatment option for life-threatening acute respiratory failure. To date, nosocomial infections or bloodstream infections occurring during ECMO support have been reported but few studies are related to infections directly attributable to ECMO devices.

**Objectives** To evaluate infection and colonization rates related to ECMO devices in VV-ECMO adult patients.

**Methods** We prospectively included all consecutive adult patients treated with VV-ECMO for at least 48 hours in a referral regional ECMO center. At the time of ECMO removal, we systematically performed blood cultures, swabs cultures on insertion cannula site (femoral and jugular) and intravascular cannula extremities cultures. Then, we classified each ECMO support according to the infectious status in three groups;

1. Uninfected/Uncolonized ECMO device,
2. ECMO device colonization,
3. ECMO device infection. Impact on outcome was assessed.

**Results** Ninety-nine patients underwent one hundred and three VV-ECMO, representing 1472 ECMO-days. The ECMO device infection rate was 6.8 per 1000 ECMO-days (10 events, 9.7 % of ECMO support) including 7 ECMO device-related bloodstream infections (4.7 per 1000 ECMO-days). The ECMO device colonization rate was 22.4 per 1000 ECMO-days (33 events, 32 % of ECMO support). Coagulase negative staphylococcus was the most frequently organism responsible for ECMO device infections (8/10, 80 %) and ECMO device colonization (20/33, 60.6 %). No difference was observed between the three groups, regarding days of mechanical ventilation, both ICU length of stay and mortality, and in hospital mortality. We observed a longer ECMO duration in the ECMO-device colonization group as compared with the Uninfected/Uncolonized ECMO-device group [12 days (9-20 days) versus 5 days (5-16 days); respectively, *p* < 0.05].

**Conclusions** At the time of ECMO device removal, we reported a low incidence of infection related to the devices. Further studies are needed to evaluate the benefits of systematic strategies using chlorhexidine-impregnated dressing to reduce the rate of colonization.

**References**

1) Brodie D, Bacchetta M (2011) Extracorporeal membrane oxygenation for ARDS in adults. N Engl J Med 365:1905-1914.

2) Schmidt M, Bréchot N, Hariri S, et al. (2012) Nosocomial infections in adult cardiogenic shock patients supported by venoarterial extracorporeal membrane oxygenation. Clin Infect Dis Off Publ Infect Dis Soc Am 55:1633-1641.

3) Bizzarro MJ, Conrad SA, Kaufman DA, et al. (2011) Infections acquired during extracorporeal membrane oxygenation in neonates, children, and adults. Pediatr Crit Care Med J Soc Crit Care Med World Fed Pediatr Intensive Crit Care Soc 12:277-281.

### A107 Experience of necrotizing soft tissue infection in the acute stage: content analysis of diaries written by close family in Denmark and Sweden (p-infect, family)

#### I. Egerod^1^, A.E. Andersson^2^, A.-M. Fagerdahl^3^, V. Knudsen^4^, P-INFECT

##### ^1^Rigshospitalet, Copenhagen University Hospital, Neurointensive Care Unit, Copenhagen, Denmark; ^2^University of Gothenburg, The Sahlgrenska Academy, Gothenburg, Sweden; ^3^Karolinska Institute, Stockholm, Sweden; ^4^Rigshospitalet, Copenhagen University Hospital, Intensive Care Unit, Copenhagen, Denmark

###### **Correspondence:** I. Egerod – Rigshospitalet, Copenhagen University Hospital, Neurointensive Care Unit, Copenhagen, Denmark

**Introduction** Severe necrotizing soft tissue infection (NSTI) is a life threatening bacterial disease that spreads quickly to cutis, sub-cutis and fasciae with an estimated mortality of 24 %^1^. Incidence in Denmark is 50-100 cases per year and in Sweden 150-200 (1-2 in 100.000). The fulminant course of NSTI progresses in the matter of hours requiring immediate diagnosis and treatment to save lives and limbs. Little is known of the human cost of the disease for patient and family in the short and long term^2^.

**Objectives** The objective was to explore the lived experience of the family during the acute stage of NSTI using diaries written by close family members.

**Methods** The study had a multicenter, binational, qualitative explorative design using diaries written by close family members (n = 17) during the acute stage of the trajectory starting in the intensive care unit (ICU) at the hospitals where NSTI is centralized in Denmark and Sweden. Family was defined in its broadest terms as spouse, partner, blood-relation, neighbor or friend. Qualitative content analysis and investigator triangulation were used. The study was part of the P-INFECT-study investigating the patient and family experience of NSTI from different perspectives. Patient and family involvement was applied in constructing the study.

**Results** The mean age of the patients was 62 years with a range of 34-92. The mean length of stay in ICU was 5.5 days with a range of 1-12 days. Main themes identified: I Trajectory, II Treatment, III Patient & Family. The first theme led to the description of the typical course, The NSTI trajectory model. The second theme focused on informational needs of the family, and the third theme identified issues of importance to the close family: Being close to the patient, Being worst for the family, Fearing relapse, Network and travel, and reflections on Life and death.

**Conclusions** NSTI is generally unknown to patient and family and comes as a shock. Patients in our sample received rapid treatment in the ICU and the worst consequences were avoided. Nevertheless, close family, including spouses were faced with the sudden risk of disfigurement or death in the patient as they coped with ambivalence of being several places at once, lack of sleep and lack of certainty. We recommend the provision of systematic information during the acute stage, including knowledge on what to expect.

**References**

^1^Hua et al. Prognostic factors in necrotizing soft-tissue infections (NSTI): A cohort study. J.Am.Acad.Dermatol. 2015;73:1006-12.

^2^Hakkarainen et al. Moving beyond survival as a measure of success: understanding the patient experience of necrotizing soft-tissue infections. J.Surg.Res. 2014;192:143-9.

**Grant acknowledgment**

The Lundbeck Foundation, R86-A3514.

### A108 Icu-acquired infections in a Tunisian medical intensive care unit

#### K. Meddeb^1^, A. Ben Cheikh^2^, Y. Hamdaoui^1^, J. Ayachi^1^, A. Guiga^3^, N. Fraj^1^, S. Romdhani^1^, N. Sma^1^, R. Bouneb^1^, I. Chouchene^1^, A. Khedher^1^, N. Bouafia^2^, M. Boussarsar^1,4^

##### ^1^Farhat Hached University Hospital, Medical Intensive Care Unit, Sousse, Tunisia; ^2^Farhat Hached University Hospital, Hospital Hygiene Unit, Sousse, Tunisia; ^3^Farhat Hached University Hospital, Internal Medicine Department, Sousse, Tunisia; ^4^Research Laboratory N° LR14ES05, Interactions of the Cardiopulmonary System, Ibn Al Jazzar Faculty of Medicine, University of Sousse, Sousse, Tunisia

###### **Correspondence:** K. Meddeb – Farhat Hached University Hospital, Medical Intensive Care Unit, Sousse, Tunisia

**Introduction** Giving their serious underlying conditions followed by the need of invasive procedures, ICU-patients are highly at risk to develop ICU-acquired infections representing an additional mortality factor.

**Objective** To estimate incidence of ICU-acquired infections in a Tunisian medical intensive care unit.

**Methods** A prospective study was conducted from September 2015 over the span of a year. All consecutive ICU patients, in whom ICU length of stay was over 48 h, were included for surveillance till discharge or death.

The data collected were, patients characteristics, initial diagnosis, SAPS II, ventilation modality, conditions of invasive catheters insertion *(peripheral venous catheter, central venous catheter, urinary catheter).* When an infection declares, the diagnosis is retained according to the Centers for Disease Control and Prevention National Healthcare Safety Network (CDC/NHSN) definition for each type of infection.

**Results** One hundred patients were included over a 7-month period. They were 55 ± 19 years mean aged. History included, diabetes mellitus, 26 % ; COPD, 25 % ; immunocompromised, 12 %. 69 % had antibiotics at admission. Mean SAPS II, 31 ± 19. 64 % were mechanically ventilated. Central venous catheters were inserted in 70 % of cases. Mean length of stay, 11 ± 10 days. Mean duration of invasive mechanical ventilation, 12 ± 9 days.

25(25 %) patients developed ICU-acquired infections. Overall ICU-acquired infections density incidence was 22.7events/1000patient days. 13 % developed ICU-acquired pneumonia. ICU-acquired pneumonia density incidence was 16,6/1000ventilator days. 9(9 %) patients were diagnosed with central line related infection with a density incidence of 13/1000central line days. 3(3 %) patients developed peripheral catheter related infections.

*Acinetobacter baumannii* was isolated in tracheal aspirates in all ICU-acquired pneumonia cases.

Overall mortality in patients who developed ICU-acquired infections was 72 %.

**Conclusion/commentary** Compared to the International Nosocomial Infection Control Consortium (INICC) report 2007-2012 (1), the present study showed similar rates for ICU-acquired pneumonia (16.6 vs 16.8/1000ventilator days), however, central line-associated infections rate was higher (13 vs 4.9/1000central line days).

**References**

1. Rosenthal V, Maki D, Mehta Y, Leblebicioglu H, Memish Z, Al-Mousa H et al. International Nosocomial Infection Control Consortium (INICC) report, data summary of 43 countries for 2007-2012. Device-associated module. American Journal of Infection Control. 2014;42(9):942-956.

### A109 A retrospective study of infections in a surgical ICU in Iran

#### A. Amirian^1^, B. Ziaian^1^, M. Masjedi^2^

##### ^1^Shiraz University of Medical Sciences, Department of Surgery, Shiraz, Islamic Republic of Iran; ^2^Anesthesiology and Critical Care Research Center, Shiraz University of Medical Sciences, Shiraz, Islamic Republic of Iran

###### **Correspondence:** A. Amirian – Shiraz University of Medical Sciences, Department of Surgery, Shiraz, Islamic Republic of Iran

**Objectives** This study was designed to investigate the clinical and microbiological characteristics of infections in a surgical intensive care unit (SICU) of a university hospital in Iran.

**Methods** It was a retrospective study of all patients who developed an infection in our ICU during a 5 year period from January 2011 up to December 2015 .

**Results** A total of 1963 consecutive patients were admitted in our ICU during this period. Among them, 569 (28.9 %) developed 1024 infections. The mean age of patients was 42.5 ± 4.8 years. 324 (56.9 %) of patients were women. Infections were: ventilator-associated pneumonia (25.9 %), bloodstream (16.7 %), surgical site (12.8 %), central venous catheter (11.1 %) and urinary tract infection (10.2 %). The microorganisms found, in order of frequency were: Acinetobacter baumannii (27.9 %), enterobacteriaceae (16.1 %), Klebsiella pneumoniae (13.3 %), Candida albicans (8.9 %), Pseudomonas aeruginosa (5.4 %) and Staphylococcus aureus (3.8 %). Microorganisms were highly resistant to antibiotics in 18.0 % of cases. The complication and mortality rates were 67.2 % and 48.1 %.

**Conclusions** Infections are one of the leading causes of morbidity and mortality in SICUs due to high incidence, drug resistance and rate of associated complications. Ventilator-associated pneumonia was the leading cause of ICU infections in our study and Acinetobacter baumannii was the most frequently found organism in our patients.

### A110 Benchmarking severe sepsis incidence in Germany: accuracy of different ICD-10 coding strategies in administrative data

#### C. Fleischmann^1,2^, D.O. Thomas-Rueddel^1,2^, A. Schettler^1^, D. Schwarzkopf^1,2^, A. Stacke^1^, K. Reinhart^1,2^

##### ^1^Jena University Hospital, Department of Anesthesiology and Intensive Care Medicine, Jena, Germany; ^2^Jena University Hospital, Center for Sepsis Control and Care, Jena, Germany

###### **Correspondence:** C. Fleischmann – Jena University Hospital, Department of Anesthesiology and Intensive Care Medicine, Jena, Germany

**Introduction** Various ICD code abstraction strategies are used to identify severe sepsis cases in administrative data, but reliable data on their validity is scarce.

**Objectives** To assess the accuracy of severe sepsis coding by validation trough clinical chart review and to identify reasons for wrong- or non-coding.

**Methods** A random sample of 1120 patients stratified by hospital length of stay and ICU admission status admitted to an academic medical center in Germany between 2007 and 2013 was selected. Severe sepsis patients were identified by the following ICD-10 abstraction strategies applied to patients' primary and secondary discharge diagnoses:

1. clinical sepsis codes (R codes),
2. explicit approach (microbiological + clinical sepsis codes [R codes]), and
3. implicit approach (infection + organ dysfunction codes).

Gold standard was the diagnosis of sepsis according to ACCP/SCCM consensus criteria based on the review of full patient charts by four independent physicians. Predictive accuracies of abstraction strategies were compared correcting for stratified sampling by using sampling weights. Following the analysis, false negatives and false positives were reviewed to determine reasons for misclassification.

**Results** From 937 charts from adult patients that were accessible in full, 81 patients with severe sepsis/septic shock. Sensitivity, specificity, positive and negative predictive values are shown in Tab. 1. Overall, explicit coding strategies are limited in their sensitivity, but have a better positive prediction than implicit coding strategies. Identification strategies based on clinical and/or microbiological sepsis codes risk underestimating true sepsis incidences by 1.4-2.2-fold, whereas indirect coding strategies carry the risk of overestimation. In explicitly identified cases which were coded false positive (n = 13), 23 % organ dysfunction was not caused by infection (23 %), infection or organ dysfunction were not documented in the chart (54 %) or patients did not meet two or more SIRS criteria (23 %). For the implicit approach, false-positives (n = 90) resulted from concurrence of infection and organ dysfunction without causality (51 %) or that infection or organ dysfunctions were not identifiable retrospectively by chart review (40 %). 9 % did not meet the SIRS criteria. In implicitly false-negatives (n = 21), organ dysfunction, infection or both were not coded in 67 %, 14 % and 17 % of cases, respectively.

**Conclusions** Existing ICD coding strategies differ in their accuracy in identifying septic patients in hospital discharge databases and thus may over- or underestimate true sepsis incidences. Standardized and valid coding strategies for severe sepsis are needed to ensure comparability between epidemiological and intervention studies based on administrative data.

**Grant acknowledgement**

The CSCC is funded by the German Federal Ministry of Education and Research, Germany, FKZ: 01EO1502.

**Fig. 42 (abstract A110).**
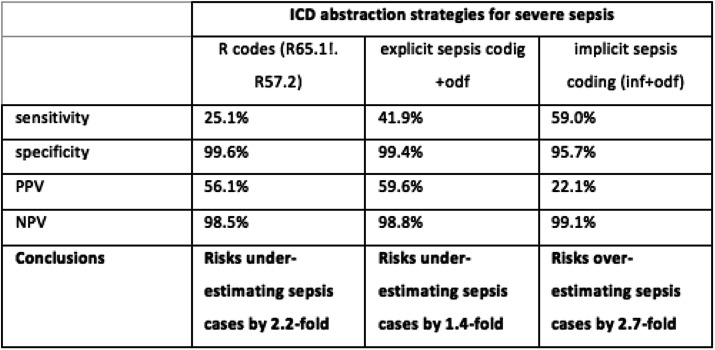
Validity of different ICD abstraction stategies

### A111 Knowledge of healthcare workers on hospital acquired infection in an Angolan ICU

#### E. Filipe^1^, A. Escoval^2^, A. Martins^3^, P. Sousa^2^, N. Velez^3^, E. Viegas^3^, E. Tomas^3^

##### ^1^Clinica Sagrada Esperança, ICU, Luanda, Angola; ^2^Escola Nacional de Saude Publica/ UNL, Lisboa, Portugal; ^3^Clinica Sagrada Esperança, Luanda, Angola

###### **Correspondence:** E. Tomas – Clinica Sagrada Esperança, Luanda, Angola

**Introduction** The level of healthcare workers knowledge on Hospital Acquired Infection (HAI) international recommendations strongly influences its increase.

**Objectives** To describe the level of knowledge of ICU doctors, nurses and physiotherapists at Sagrada Esperança Clinic on HAI international recommendations.

**Methods** It was applied to ICU doctors, nurses and physiotherapists the questionnaire used in the EVIDENCE study, which includes 50 comprehensive questions concerning bloodstream infection, catheter-associated infection, ventilator-associated pneumonia, urinary catheter-associated infection, surgical site infection, hands washing and general knowledge on HAI. We validated the number of correct answers and rated the staff as GOOD (50-40); MEDIUM (39 to 30); ENOUGH (29-25) and POOR (<25).

**Results** Out of 50 healthcare workers invited to participate, we received and analyzed a total of 28 completed questionnaires, with a response rate of 50 % - 8 doctors (28.5 %), 16 nurses (57.1 %), 4 physiotherapists (14.3 %). Overall, mean age ± standard deviation was 41 ± 6.98 - doctors: 44.2 ± 6.2; Nurses: 41.0 ± 7.0; physiotherapists: 34.5 ± 6.9. Most participants were females (57.4 %). The mean number of years working in the ICU was 10.4 ± 7.6 - doctors: 12.4 ± 6.4; nurses: 12.5 ± 7.6; physiotherapists: 2.0 ± 7.1. Fourteen (50 %) healthcare workers received training in HAI in the past three years, ten (35.7 %) did not attend to any training and four (14.3 %) omitted. Mean of correct answers: 13.1 ± 7.0 and wrong answers: 14.9 ± 7.0; (P = 0,22). No healthcare worker was rated as GOOD; three were rated as MEDIUM (all doctors); eight as ENOUGH (three doctors and five nurses); seventeen as POOR (two doctors, ten nurses and four physiotherapists). Comparing the results, we found a significant difference between doctors and nurses (P = 0.022) and between doctors and physiotherapists (P = 0.005). There was no difference between nurses and physiotherapists (P = 0.071).

**Conclusions** Healthcare workers did not show, in this study, good knowledge on Hospital Acquired Infection international recommendations.

**Reference**

1) Irene Ocran, Daniel Nii Aryee Tagoe. Asian Pac J Trop Dis 2014; 4(2): 135-139

### A112 Assessment of clinic-microbiological profile as mortality predictors in patients with sepsis

#### G. Snell, R. Matsa

##### Royal Stoke University Hospital, Critical Care, Stoke-on-Trent, United Kingdom

###### **Correspondence:** G. Snell – Royal Stoke University Hospital, Critical Care, Stoke-on-Trent, United Kingdom

**Introduction** Sepsis is one of the leading causes of mortality amongst ICU patients [1]. However there is a paucity of information on the variables that could advise the physician to predict mortality.

**Objectives** To identify risk factors associated with poor prognosis in patients with sepsis on the critical care unit.

**Methods** A retrospective, observational study performed in a tertiary referral University Hospital over 12 months (September 2014 to September 2015). Inclusion: All patients admitted to the ICU with a diagnosis of sepsis and coded by the audit team. Exclusion: Age less than18 years and patients admitted with end of life care plan. Data collection: Case notes were reviewed and the following details were recorded in a predesigned data collection sheet: demographic profile, admission diagnosis, APACHE II score, antibiotics, co-morbidities, survival and length of stay. Ethical approval was not sought as study considered a service evaluation.

**Results** We included 236 patients (n = 236). The mortality rate was 38.1 %. The demographic details of the patients can be seen in the table.

It was noted that age and APACHE II score at admission significantly are associated with high mortality. Mortality increased proportionately with time to administration of antibiotics although not significantly. It was noted that the most common source of infection was the respiratory system (64 %) as demonstrated in the graph below.

The most common antibiotic used was piperacillin-tazobactam. Mortality was higher in patients with a respiratory or soft tissue/bone source of infection, the lowest mortality was seen in infections of the nervous system.

**Conclusions** Our study demonstrates that increased age, high APACHE II score and delay in administration of antibiotics are associated with higher mortality in sepsis related admissions to ICU. We aim to develop a scoring system identifying different variables which will contribute to informed decision making in sepsis related admissions.

**Reference** [1] Vincent JL, Akr Y, Sprung CL, et al. Sepsis in European intensive care units: results of the SOAP study. Crit Care Med 2006 Feb; 34(2): 344-53

**Table 41 (abstract A112).** Demographic Data

	Survived (n=90)	Death prior to ITU Discharge (n=146)	P value
Age (yrs) (SD +/- SEM)	59.5 ± 1.246	64.01 ± 1.411	0.0208
Apache II (SD +/- SEM)	19.25 ± 0.541	22.8 ± 0.7393	0.0002
Time to antibiotics (minutes) (SD +/- SEM)	128.9 ± 15.05	165.4 ± 24.5	0.1971
Male: Female (n=136:100)	81:65	55:35	
Admitted from Emergency Dept. (n=80)	46	34	
Admitted from Acute Medical Unit (n=45)	29	16	
Admitted from other medical ward (n=49)	25	24	
Admitted from Surgical ward (n=48)	33	15	
Admitted from theatre (n=14)	13	1

**Fig. 43 (abstract A112).**
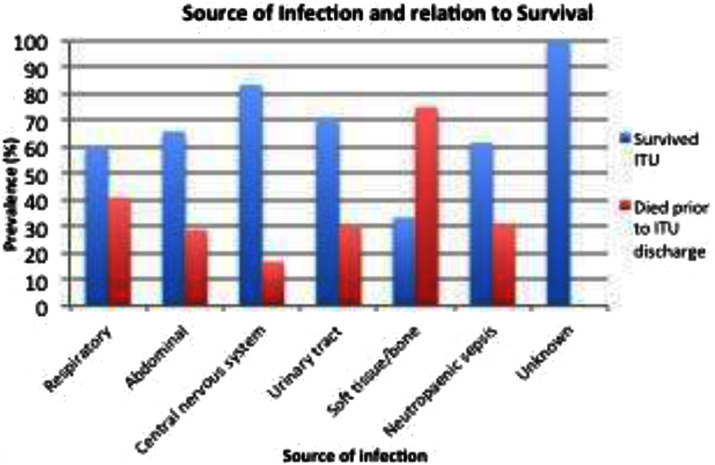
Source of Infection and Survival

### A113 Clinical profile and outcomes of patients with severe sepsis: a prospective analysis from an intensive care unit in India

#### T.T.S. Paary^1^, M.S. Kalaiselvan^2^

##### ^1^Apollo Hospitals, Intensive Care Medicine, Bangalore, India; ^2^SRI Ramachindra University, Intensive Care Medicine, Chennai, India

###### **Correspondence:** T.T.S. Paary – Apollo Hospitals, Intensive Care Medicine, Bangalore, India

**Introduction** Sepsis is the leading cause of ICU admissions, associated with high mortality. Very few Indian data is available to identify the incidence of severe sepsis/ septic shock and their outcomes.

**Objectives** To identify the incidence, risk factors and outcomes of patients with severe sepsis/septic shock

**Methods** Prospective observational study, done in a multidisciplinary ICU over a period of 18 months. We included all adult patients admitted to ICU with features of severe sepsis/septic shock as per SCCM/ACCP guidelines. Data collection was done on demography, co-existing illness, parameters to assess Acute Physiology and Chronic Health Evaluation (APACHE) II and Sequential Organ Failure Assessment (SOFA) scores and other relevant lab data including vital parameters. Data concerning the source of infection was obtained. Organ failures and other supportive measures taken were captured. Primary outcome data on mortality was collected and secondary outcome data on ventilator days, ICU length of stay (ALOS) and ventilator free days were captured.

**Results** 1162 patients were screened and 356 patients had severe sepsis; incidence of severe sepsis was 30.6 %, mortality rate was 51.6 %. Admission APACHE II (23.37 ± 9.47) and SOFA (7.58 ± 4.05) scores were high. Most common source of infection was from respiratory tract (37.2 %) followed by urinary tract (10.3 %) and intra-abdominal (9.5 %) infections. 62.9 % of patients required ventilator support, 25.5 % of patients required vasopressor support despite adequate fluid resuscitation, more than one third of patients required renal replacement therapies (35.7 %). Hematocrit, total leucocyte count, serum bilirubin and SOFA scores were significantly higher among non-survivors.

**Conclusions** Incidence of severe sepsis was high and was associated with poor patient outcomes.

**References**

• Angus DC, Linde-Zwirble WT, Lidicker J et al (2001) Epidemiology of severe sepsis in the United States: analysis of incidence, outcome, and associated costs of care. Crit Care Med 29:1303-1310

• Dellinger RP (2003) Cardiovascular management of septic shock. Crit Care Med 31:946-955

• Martin GS, Mannino DM, Eaton S et al (2003) The epidemiology of sepsis in the United States from 1979 through 2000. N Engl J Med 348:1546-1554

• Linde-Zwirble WT, Angus DC (2004) Severe sepsis epidemiology: sampling, selection, and society. Crit Care 8:222-226

• Dombrovskiy VY, Martin AA, Sunderram J et al (2007) Rapid increase in hospitalization and mortality rates for severe sepsis in the United States: a trend analysis from 1993 to 2003. Crit Care Med 35:1414- 1415.

### A114 Quality of concurrent cleaning in icu beds: effects of an educational initiative

#### A.M. Cavalheiro, L.L. Rocha, C.S. Vallone, A. Tonilo, M.D.S. Lobato, D.T. Malheiro, G. Sussumo, N.M. Lucino

##### Hospital Israelita Albert Einstein, ICU, São Paulo, Brazil

###### **Correspondence:** A.M. Cavalheiro – Hospital Israelita Albert Einstein, ICU, São Paulo, Brazil

**Introduction** Inadequate ICU bed concurrent cleaning is associated with increased risk of nosocomial infections in critically ill patients. Healthcare provider training and continuous education have been associated with better outcomes in several settings. **Objectives** To evaluate the microbiological impact of training a critical care staff in concurrent cleaning of ICU beds.

**Methods design** Before-and-after study. Intervention: Training program in concurrent cleaning for critical care healthcare providers. Period and setting: From January 2014 to October 2014 in a mixed private ICU. A pretest questionnaire was applied to all participating nurses to assess initial knowledge of concurrent cleaning. After training a new test was applied and the participating nurse was deemed approved if answers correctly at least 80 % of the questions. Microbiological burden was evaluated before training and 30 days by a quantitative ATP essay. The ICU bed was considered clean if quantitative assay resulted was less or equal to 150 URL. If quantitative essay result was higher than 150 URL the ICU bed was considered contaminated and a new cleaning process initiated. Each microbiological burden evaluation included three different moments, as follows: (1) before patient admission, (2) six hours after admission, and (3) 24 hours after admission.

**Results** A total of 60 ICU beds were included, being 30 before training and 30 after training. We included in training 269 critical care healthcare providers. The pre and post-test was answered by all participating providers, which answered correctly to 73 % and 93 % of the questions, respectively. The microbiological quantitative results before training were 259 (±101) URL, 209 (±103) URL and 261(±101) URL for before admission, six hours after admission and 24 hours after admission, respectively. Thirty days after training, the results were 169 (±110) URL, 61 (±21) URL and 52 (±20) URL for before admission, six hours after admission and 24 hours after admission, respectively. The microbiological burden significantly reduced after training before admission (p = 0.001), six hours after admission (p < 0.001) and 24 hours after admission (p < 0.001).

**Conclusions** A training program in concurrent cleaning for critical care healthcare providers was effective in reducing the burden of microbiological specimens in ICU beds.

**References**

Avila MAG, Bocchi SCM. Telephone confirmation of a patient´s intent to be present for elective surgery as a strategy to reduce absenteeism. Rev Esc Enferm USP. 2013;47(1):193-7.

Rapparini C, Saraceni V, Lauria LM, Barroso PF, Vellozo V, Cruz M, et al. Occupational exposures to bloodborne pathogens among healthcare workers in Rio de Janeiro, Brazil J Hosp Infect. 2007;65(2):131-7

**Grant acknowledgement**

None.

### A115 Impact of a multimodal infection control approach on the incidence of catheter related hospital acquired infections in a medical-surgical ICU. A two year study of international nosocomial infection control consortium (INICC) in Shiraz, Iran

#### F. Zand^1^, V.D. Rosenthal^2^, M. Masjedi^1^, G. Sabetian^3^, B. Maghsudi^1^, M. Ghorbani^1^, A. Sanaei Dashti^4^, A. Yousefipour^1^

##### ^1^Anesthesiology and Critical Care Research Center, Shiraz University of Medical Sciences, Shiraz, Islamic Republic of Iran; ^2^International Nosocomial Infection Control Consortium, Buenos Aires, Argentina; ^3^Trauma Research Center, Shiraz University of Medical Sciences, Shiraz, Islamic Republic of Iran; ^4^Shiraz HIV/AIDS Research Center, Shiraz University of Medical Sciences, Shiraz, Islamic Republic of Iran

###### **Correspondence:** F. Zand – Anesthesiology and Critical Care Research Center, Shiraz University of Medical Sciences, Shiraz, Islamic Republic of Iran

**Introduction** Prevention of hospital acquired infections is among the most intricate problems in critical care setting.

**Objectives** This study sought to assess the effect of a multidimensional approach developed by our team on the reduction of catheter related hospital acquired infection (CR-HAI) rates in the patients hospitalized in an adult intensive care unit (AICU) in an INICC member hospital in Shiraz, Iran.

**Methods** The study was divided into two periods: During the baseline period, we conducted active prospective surveillance of VAP, VAE, CLABSI and CAUTI using the CDC and NHSN definition and INICC methods. During the intervention period, we implemented a multidimensional approach for VAP, VAE, CLABSI and CAUTI in addition to performing active surveillance. This multidimensional approach included a bundle of infection control interventions including hand hygiene, active screening, contact precaution and environmental cleaning. The baseline rates of CR-HAI were compared to the rates obtained after intervention, and we analyzed the impact of our interventions by Z score.

**Results** The two years of study period were divided to 4 epochs with six-month duration. 621 patients were admitted in the ICU with 6190 bed days. Central line utilization ratio was 65 percent.The rate of CLABSI was reduced from 21 to 7 per thousand catheter days (p < 0.007). The incidence of VAP plus VAE decreased from 26 to 11 per thousand mechanical ventilation days (p < 0.0128) while ventilator utilization ratio was 51 percent. The rate of CAUTI also decreased from 23 to 9 per thousand catheter days (p < 0.0126).

**Conclusions** The implementation of this multidimensional approach for CR-HAI was associated with a significant reduction in the CLABSI, VAP plus VAE and CAUTI rates in the participating AICU.

**Fig. 44:**
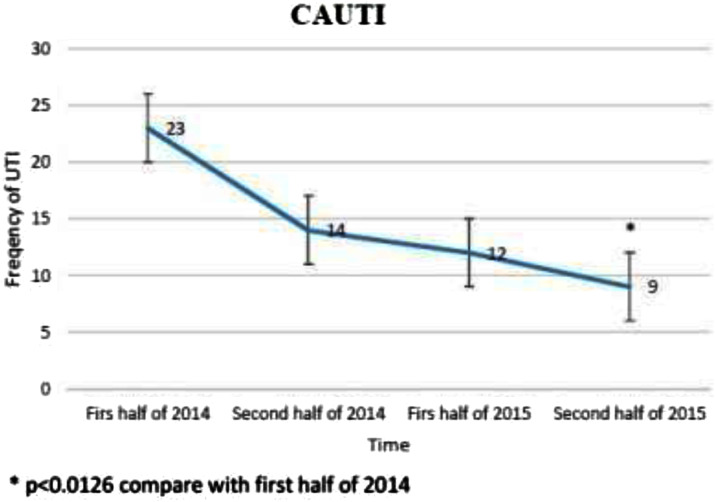
(abstract A115).

**Fig. 45:**
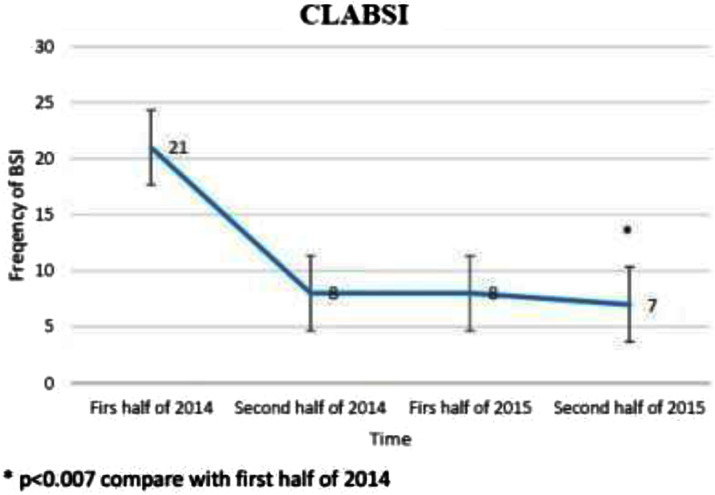
(abstract A115).

**Fig. 46 (abstract A115).**
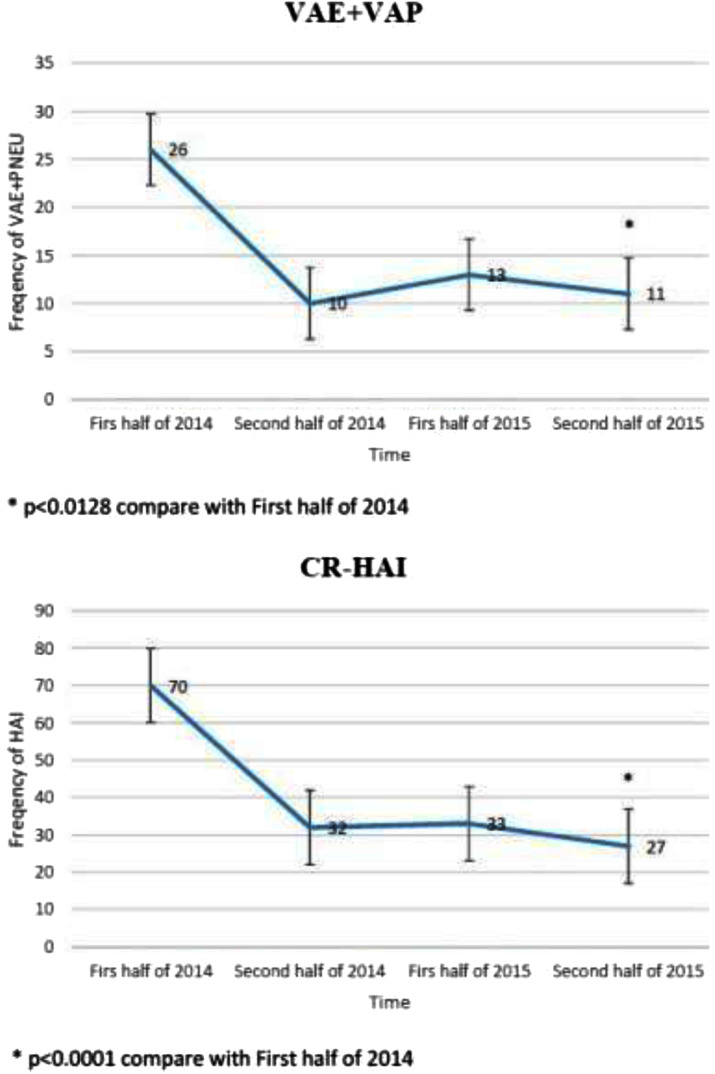
VAE + VAP

### A116 The root of the problem - what do we learn from root cause analysis?

#### J.R. Goodall^1^, M. Williamson^2^, E. Tant^2^, N. Thomas^2^

##### ^1^Salford Royal NHS Foundation Trust, Critical Care, Salford, United Kingdom; ^2^Salford Royal NHS Foundation Trust, Manchester, United Kingdom

###### **Correspondence:** J.R. Goodall – Salford Royal NHS Foundation Trust, Critical Care, Salford, United Kingdoms

**Introduction** At Salford Royal Foundation Trust(SRFT), all HCAIs involving infection with methicillin resistant staph aureus (MRSA) and Clostridium difficile (C. diff.) are fully investigated using the process recommended by the National Patient Safety Agency, which includes the use of 'Root Cause Analysis (RCA)', where the events surrounding such infections are fully described and compared with acceptable practice.

The sharing of outcomes of such investigations is an essential part of the process; unless the results are disseminated lessons cannot be learnt by staff involved. The NHS national patient safety agency describes a seven stage process to patient safety, which outlines this process clearly.^1^

**Objectives** To how assess how effectively the results of investigations into infection with MRSA and C.diff are disseminated to staff working in the CCU at SRFT.

**Methods** On 22.11.2015, all CCU staff on duty were asked to complete a questionnaire, and to do so without reference to additional sources of information (eg noticeboards, colleagues and websites).

The survey asked participants the following questions:

· How long ago was the last C.diff/MRSA infection on CCU?

· Do you know what the RCA into this infection showed?

Participants were then asked for additional comments, including how they had heard about the results of the investigations, and for ideas for on how such information could be more effectively shared.

**Results** 33 members of staff participated in our study and all groups from the MDT were represented.

Most of the staff were not aware of the time since the most recent infections, as can be seen in Table 42.

Perhaps even more worryingly, fewer than one third of the participants were aware what the investigation into either of these HCAIs had shown.

**Conclusions** On the day of the survey, the most recent cases of MRSA and C.diff had been fully investigated and the results of the RCAs into the events had been published. Despite the completion of these investigations, our survey shows the learning points raised had only been shared with a small percentage of the critical care team, limiting the impact of the investigative work.

As a result of this work, we intend to involve more members of the MDT in RCAs, and change the way results of such investigations are communicated with the critical care team to ensure any lessons can be learnt by a wider group of individuals.

**References**

1. Seven steps to patient safety. An overview guide for NHS staff. London: The National Patient Safety Agency 2nd Edition, April 2004

**Table 42 (abstract A116).** Number of staff indicating time since most recent C.diff and MRSA acquisitions

	<1 month	2-3 months	3-6 months	6-12 months	12-18 months	>18 months
C. difficile	9	9	4^a^	7	4	0
MRSA	2	13^b^	3	7	6	2

^a^ Most recent C.diff acquisition occurred on 25.06.15 (5 months before the survey)

^b^ Most recent MRSA acquisition occurred on 26.09.16 (2 months before the survey)

### A117 Withdrawn

### A118 Prevalence of intensive care infections in the research center of Kocaeli, Turkey

#### C. Balci, C. Gonen, E. Haftacı, H. Gurarda, E. Karaca

##### Kocaeli Derince Education and Research Hospital, Kocaeli, Turkey

###### **Correspondence:** C. Balci – Kocaeli Derince Education and Research Hospital, Kocaeli, Turkey

**Introduction** ICU infections increases the ICU mortality and length of hospital stay is extended. Micro-organsims that cause infections in intensive care varies according to country and type of intensive care.

**Objectives** The aims of this study were to determine the the intensive care infections (ICI) prevalence in Derince Kocaeli Educational Hospital, to ascertaine risk factors, to describe the pathogens associated with intensive care infections

**Methods** In our study, we aimed to identify the infectious agent retrospectively. Point-prevalence survey in march 2012 concerning all patients who had been in the intensive care unit for at least 48 hours. A retrospective analysis of 1780 patients who underwent ICU therapy between 2012 and March 2016 was performed. The primary outcomes of our study, we aimed to identify the infectious agent retrospectively. The secondary outcomes of our study, was to identify areas of infections in intensive care.

**Results** 1780 patients undergoing enfection were screened. Infectious agents are as respectively Pseudomonas aeroginosa, Acinetobacter baumania and Klebsiella pneumonia . The area of infection, respectively: lower respiratory tracts, urinary tract and blood circulation. There were 1780 cases admitted to the ICU. ICU infectious agents per cent respectively; Pseudomonas auroginosa (%28.7), Acinetobacter baumania ( %14.29), Klebsiella pneumonia (%28,57).

**Conclusions** We think that is appropriate to the profile of the factors of intensive care infections in intensive care in Turkey. At the end of the study, there was no resistance in Acinetobacter colistin. Risk factors associated with infection were longer of hospital stay, precence comorbidity and mulitrauma. Still in Turkey, the first in the intensive care unit is located Gram negative factors.

**References**

Year in review in **Intensive Care** Medicine 2012. II: Pneumonia and **infection**, sepsis, coagulation, hemodynamics, cardiovascular and microcirculation, **critical care** organization, imaging, ethics and legal issues

Massimo Antonelli, Marc Bonten, Maurizio Cecconi, Jean Chastre, Giuseppe Citerio, Giorgio Conti, J. Randall Curtis, Goran Hedenstierna, Michael Joannidis, Duncan Macrae, Salvatore M. Maggiore, Jordi Mancebo, Alexandre Mebazaa, Jean-Charles Preiser, Patricia Rocco, Jean-François Timsit, Jan Wernerman, Haibo Zhang

**Intensive Care** Med. 2013 March; 39(3): 345-364. Published online 2013 January 5. doi: 10.1007/s00134-012-2804-

## Therapeutic interventions in neurointensive care

### A119 Is there a time for an individualized therapeutic plasma exchange protocol in patients with Guillain-Barré syndrome?

#### B. Paldusová^1^, I. Zýková^1^, D. Šímová^2^

##### ^1^Regional Hospital Liberec, Department of Anesthesia and Intensive Care, Liberec, Czech Republic; ^2^Regional Hospital Liberec, Neurocentre, Liberec, Czech Republic

###### **Correspondence:** B. Paldusová – Regional Hospital Liberec, Department of Anesthesia and Intensive Care, Liberec, Czech Republic

**Introduction** Plasmapheresis (therapeutic plasma exchange - TPE) is an established treatment option in the therapy of Guillain-Barré syndrome (GBS). 3 randomized controlled trials established its efficiency between 1985 and 1995. So far the recommended number of TPE sessions is four, but the optimum TPE protocol (number of exchanges and volumes exchanged) remains to be established (1). Also the role of TPE in patients with GBS who fail to respond to therapy and who relapse after therapy remains to be determined (1). TPE is expected to be a logical therapeutic option in diseases in which pathogenesis is linked with a biological substance with a relatively high molecular weight, a slow rate of formation and a distribution in the vascular space. TPE is used in the treatment of diseases in which the pathogenesis is associated with abnormal circulating pathogenic autoantibodies (2). The role of antibodies against gangliosides in GBS is established only in Miller Fisher variant, in other types of GBS the role of antibodies still remains to be determined. But as the optimum TPE protocol (especially in patients who fail to respond to therapy and in patiens who relapse after standard TPE protocol) has not been determined, it is a question, whether in these patients the role of antibodies should not be considered and the number of TPE sessions should not be individualized and adapted to these results.

**Objectives** We wanted to determine the relationship of antibodies to gangliosides in patients with GBS and TPE and its influence on TPE protocol.

**Methods** We prospectively screened patients with GBS treated by TPE for antibodies to gangliosides before, during and after TPE series and adapted the number of TPE to the neurological status and results of antibodies.

**Results** In 2014 we had and unexpected number of GBS patients at our ICU. 4 patients were admitted to our ICU during the period of 3 months.In all 4 patients we chose TPE as an initial therapy. We tested the patients for antibodies against gangliosides before, during and after the TPE protocol and adapted the number of TPE to the clinical course of the disease and to the results of antibodies against gangliosides. In patients with positive antibodies and a remaining neurological defficiency or a relapse we repeated a series of TPE.

**Conclusions** The studies on TPE protocols and GBS are 20 years old. The optimal TPE protocol remains to be established. The question is whether the efficacy of TPE should not at least in patients with severe neurological symptoms and in patients who fail to respond to standard TPE protocol be individualised according to the results of antibodies to gangliosides.

**References**

1. Cortese I., Chaudhry V., et al. Evidence-based guideline update:Plasmapheresis in neurological disorders. Neurology 2011,Jan 18;76(3):294-300.

2. Williams ME, Balogun RA. Principles of separation:Indications and Therapeutic Targets for Plasma Exchange.Clin J Am Soc Nephrol 2014,Jan 7;9(1):181-190

**Table 43 (abstract A119).** Patients with GBS

Patients	Type of GBS	No. of TPE	Dates of TPE	Results of antibodies	Results of antibodies	Results of antibodies	Results of antibodies	Outcome
Male, 28 years	AMAN	15	6 June-1 July	16 June -positive	16 July- positive			recovered
Female, 29 years	AIDP	9 TPE, 5 TPE (relaps)	5 - 19 June 29 July-11 Aug	6 June - positive	29 July borderline	7 August borderline	11 August negative	relapse, recovered
Male, 58 years	AMSAN	13	29 June-22 July	9 July borderline	25 July negative			recovered
Male, 59 years	AIDP	9	9 July-24 July	9 July- positive	16 July- positive	25 July borderline		recovered

### A120 Failure of potassium regulation following barbiturate coma for traumatic brain injury - can we predict complications?

#### S. Houston, L. D'Antona, J. Lloyd, V. Garnelo-Rey

##### St Mary's Hospital, Adult Intensive Care Unit, London, United Kingdom

###### **Correspondence:** S. Houston – St Mary's Hospital, Adult Intensive Care Unit, London, United Kingdom

**Introduction** Traumatic brain injury (TBI) is a common cause of morbidity in Europe. Managing raised intracranial pressure (ICP) with barbiturate coma is a final option in medical management^1^. Interactions between thiopentone and potassium have been reported^2^ and have been attributed to mortality in our ICU.

**Objectives** To review the frequency, complications and contributing factors in the loss of potassium homeostasis during and after a thiopentone infusion.

**Methods** Patients who were prescribed thiopentone by infusion from March 2011-May 2015 were selected. Serum potassium was recorded at baseline and 6 hourly until 72 hours or infusion stopped.

Following cessation, potassium levels were recorded 6 hourly for 60 hours unless death occurred. Potassium replacement during the 72 hours and the use of insulin were noted. Complications following infusion were reviewed.

**Results** In total 50 patients were prescribed thiopentone. Eight received a bolus and three had other indications. A further 3 patients had a duration under 6 hours and were excluded. Thirty-six patients were reviewed with 1 patient dying during infusion. Patient characteristics are shown in Table 44.

Hypokalaemia (potassium < 3.5) developed in 25 patients (69.4 %) during infusion with the mean lowest at 12 hours (Fig. 49). Subsequently 11 (31.4 %) patients developed hyperkalaemia (potassium > 5.5), mean peak 12 hours after.

Three patients died due to cardiac arrest with hyperkalaemia following infusion and 2 patients required filtration to control potassium.

Insulin use, duration of infusion, weight, potassium replacement or presence of hypokalaemia had no statistical significance relating to loss of regulation (Tables 45 and 46).

**Conclusions** After fatalities attributed to hyperkalaemia post barbiturate coma, we aimed to assess the occurrence and any contributing factor. A case series^2^ highlighted the potassium replacement as a significant variable. This was not shown here, possibly due to restrictive replacement in patients during infusion. We did find an earlier fall in potassium following the start of infusion and an earlier peak after cessation. That no variable showed significance suggests a lack of understanding of the cause of dysregulation.

This study was limited by sample size, likely due to limiting barbiturate coma to a final attempt at ICP control. Further large trials may be required to identify variables predicting hyperkalaemia.

Overall, with little to indicate which patients will develop hyperkalaemia, ICU staff should beware this potentially fatal complication.

**References**

1. Brain Trauma Foundation, American Association of Neurological Surgeons, Congress of Neurological Surgeons, et al. Guidelines for the management of severe traumatic brain injury. Introduction J Neurotrauma 2007; 24 Suppl 1:S1.

2. Ng, S.Y., Chin, K.J., Kwek, T.K. Dyskalaemia associated with thiopentone barbiturate coma for refractory intracranial hypertension: a case series. Intensive Care Med. 2011;37:1285-1289

**Table 44:** (abstract A120).

Patient characteristics	Number (%) / Mean ± SD
Age	41 ± 15
Male	26 (72%)
Female	10 (27%)
Weight	76 ± 13 kg
Duration of infusion	80 ± 55 hours
Insulin during infusion	17 (47%)

**Fig. 47:**
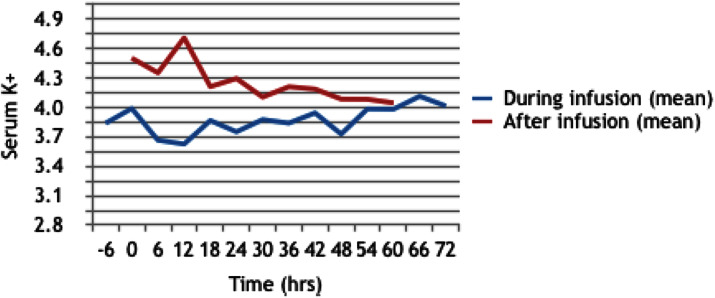
(abstract A120).

**Table 45 (abstract A120).** Hypokalaemia during infusion

	Hypokalaemia	No hypokalaemia	
Insulin	13	5	
No Insulin	12	6	chi-square p=0.88
Duration (hours- mean) ± SD	85.68 ± 52	66.27 ± 56	t-test p=0.32
Initial potassium	3.8	3.8	
Weight ± SD	78.2 ± 12.7	73 ± 15	t-test p=0.29

**Table 46 (abstract A120).** Hyperkalaemia following infusion]

	Hyperkalaemia	No hyperkalaemia	
Insulin	6	11	
No insulin	5	13	chi-square p=0.63
Duration (hours- mean) ± SD	67.7 ± 27	86.8 ± 63	t-test p=0.34
Weight ± SD	81.2 ± 16	74.9 ± 11	t-test p=0.19
K+ replacement (mmol) ± SD	272 ± 202.8	216 ± 139.3	t-test p=0.34
Hypokalaemia during infusion	9	16	
No hypokalaemia	2	8	chi-square p=0.36

### A121 IL-1β and TGF β concentration in cerebrospinal fluid in patients with primary intraventricular hemorrhage (IVH) treated with clot lysis with low-dose tissue plasminogen activator (TPA)

#### M. Sosic^1^, V. Sotosek-Tokmazic^2^, J. Kuharic^2^, I. Antoncic^1^, S. Dunatov^1^, A. Sustic^2^

##### ^1^Faculty of Medicine, University of Rijeka, Department of Neurology, Rijeka, Croatia; ^2^Faculty of Medicine, University of Rijeka, Department of Anesthesiology, Reanimatology and Intensive Care Medicine, Rijeka, Croatia

###### **Correspondence:** M. Sosic – Faculty of Medicine, University of Rijeka, Department of Neurology, Rijeka, Croatia

**Introduction** Inflammation is a key player in brain damage and increased production of pro-inflammatory and lower levels of the anti-inflammatory cytokines in periphery blood and cerebrospinal fluid (CSF) are associated with poorer clinical outcome after primary intraventricular hemorrhage (IVH).

**Objectives** The aim of this study was to investigate changes in concentration of interleukin (IL)-1β and Transforming Growth Factor β (TGF β) in CSS of patients with IVH and external ventricular drainage treated with catheter-based clot lysis with low-dose tissue plasminogen activator (tPA).

**Methods** Thirty adult patients with IVH were enrolled in the prospective study. The patients were divided in two groups: group A was treated with 3x1 mg/12 h tPA (15 pts; age 69 (60-74); male 7) and control group B with placebo (15 pts; age 62 (59-68); male 8). Intracerebral hemorrhage score on admission was equal in both groups (ICH = 3 (2-3)). Concentration of IL-1 β and TGF β in CSF were determined 24 hours (day 1), 3 and 7 days after the start of clot lysis.

**Results** In the patients from group A the concentrations of IL- 1β were significantly lower on day 3 (19,5 (11,5-28,5) vs. 39,7 (16,8-50,5); p = 0,022) and on day 7 (9 (0,2-13,3) vs. 50 (22,6-65); p < 0,001) and not significantly lower on day 1 (26 (12,3-38,2) vs. 37,8 (15,4-59,3); p = 0,15), while the concentrations of TGF β were significantly higher on day 1 (2781 (1987,7-3611 vs. 1982,7 (1777,4-1985); p = 0,009) and day 3 (2179 (1889-2249,3) vs. 1679 (1652,8-1777,4); p = 0,002) and not significantly higher on day 7 (2321 (1787,7-2057,3) vs. 2057,3 (1756,8-1862); p = 0,51).

**Conclusion** Intraventricular clot lysis with low-dose tPA probably amplifies the initial anti-inflammatory response and diminishes inflammatory response at a later stage.

### A122 Incidence and bacteriology profile of infectious complications in barbiturate coma therapy for refractory intracranial hypertension due to traumatic brain injury

#### C.T. Chong^1^, M. Sim^2^, T. Lyovarin^2^

##### ^1^Tan Tock Seng Hospital, National Healthcare Group (Singapore), Singapore, Singapore; ^2^National University of Singapore, Singapore, Singapore

###### **Correspondence:** C.T. Chong – Tan Tock Seng Hospital, National Healthcare Group (Singapore), Singapore, Singapore

**Introduction** Bone marrow suppression, leucopenia and infectious complications have been reported during the use of barbiturate coma therapy (BCT) for refractory intracranial hypertension. However, these studies have mainly involved small sample sizes. Thus, although effective in lowering intracranial pressure, barbiturate-mediated infections may severely limit the potential life saving utility of BCT.

**Objectives** /**Methods** We conducted a retrospective cohort study of all patients (n = 72) receiving thiopentone BCT for the control of refractory intracranial hypertension in a neurosurgical intensive care unit over 4 years. We collected data including changes in cell count, procalcitonin levels and incidence of clinically diagnosed infections. The microbiological profile of the organisms isolated and antibiotics prescribed were also analysed.

**Results** The mean pre-induction WBC count was 15.1 ± 12.2 x 109 L; 91.7 % of patients experienced a decrease in WBC count after induction with a mean maximal decrease in white cell count of 9.15 x 109 L. The incidence of leucopenia and neutropenia were 34.7 % and 2.8 %, respectively. Procalcitonin levels were generally raised as early as first day of BCT. The incidence of clinical infections was 46.2 %. Pneumonia (n = 15) and blood stream infections (n = 9) accounted for majority of the infections. The main causative organisms causing pneumonia were Klebsiella pneumonia (n = 4), Staphyloccus aureus (n = 2) and Pseudomonas aeruginosa (n = 2). The main cultured organisms from blood stream infections were Pseudomonas aeruginosia (n = 3), Klebsiella pneumonia (n = 1), Enterococcus cloacae (n = 1) and Acinetobacter baumannii with Staphyloccus aureus (n = 1). Out of 40 positive cultures, Klebsiella pneumonia (n = 14, 1 was multiresistant) and Staphyloccus Aureus (n = 9, 4 were methicillin-resistant) constituted the commonest bacteria isolated.

**Conclusions** Leucopenia and infections (predominantly pneumonia) are common complications in patients on BCT for refractory intracranial hypertension.

**References**

1. Frenette A, Perreault M, Lam S, Williamson D. Thiopental-Induced Neutropenia in Two Patients with Severe Head Trauma. Pharmacotherapy. 2007;27(3):464-471.

2. Stover J, Stocker R. Barbiturate coma may promote reversible bone marrow suppression in patients with severe isolated traumatic brain injury. Eur J Clin Pharmacol. 1998;54(7):529-534

**Fig. 48 (abstract A122).**
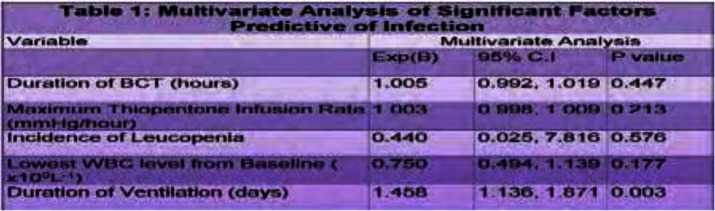
Predictive Factors for Infections

**Table 47 (abstract A122).**
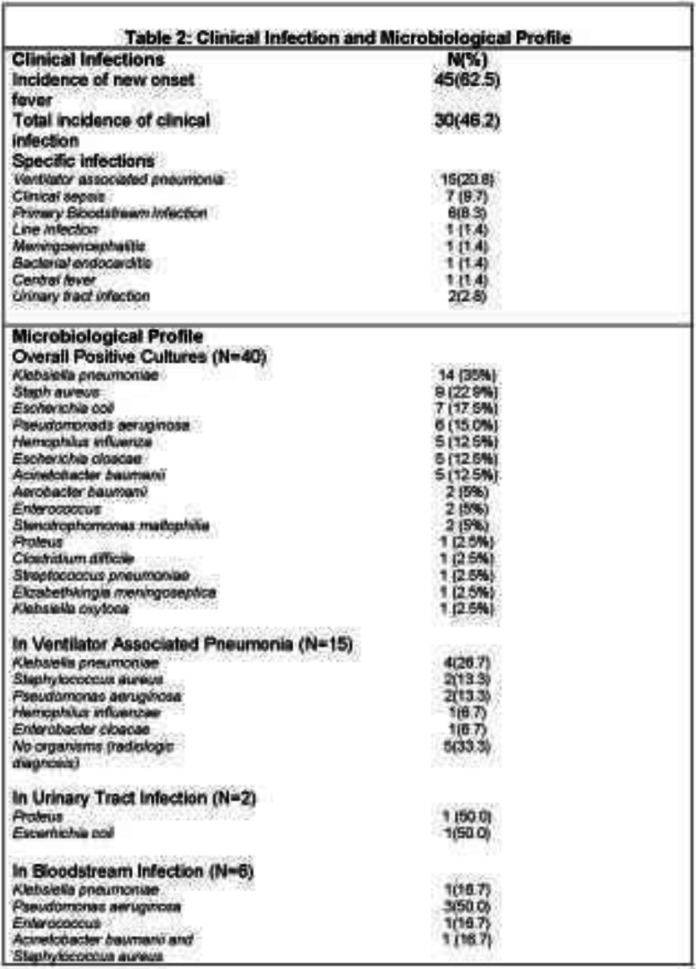
Clinical Infections and Bacteriological Profile

### A123 Impact of new recomendations of acute ischemic stroke and neuroradiology studies in the outcomes in patients treated in a polyvalent ICU of a university hospital during 15 months

#### F.M. Acosta Díaz, S. Narbona Galdó, M. Muñoz Garach, O. Moreno Romero, A.M. Pérez Bailón, A. Carranza Pinel, M. Colmenero

##### Hospital Universitario San Cecilio, Granada, Spain

###### **Correspondence:** F.M. Acosta Díaz – Hospital Universitario San Cecilio, Granada, Spain

**Introduction** Stroke is a disease with high morbidity and mortality, it constitutes a main medical cause of permanent disability in adulthood, and in Spain it is one of the leading causes of death. The intravenous thrombolysis is an approved treatment in selected patients that with the new recomendations this group could be bigger. Advances in imaging studies for acute ischemic stroke are largely due to the development of new efficacious treatments carried out in the acute phase. The computed tomography (CT) perfusion studies and CT angiography facilitates the selections of patients who are likely to benefit from appropriate early treatment.

**Objectives** To analyze the impact of new recomendations for acute ischemic stroke treatment in the selection of patients for treatment and the outcomes in patients atended in a polyvalent ICU during the last year (2015) and the first trimester of this year.

**Methods** Consecutive registry of patients seen in the last year (2015) and the first trimester of this year whom were suffering acute stroke in the ICU of a university hospital. Descriptive analyze of the registry during this period.

**Results** During 2015 were approached as suffering an acute stroke 28 patients, 13 women (46 %) and 15 men (54 %) with a medium age of 62.3 y.o.; in whom were treated with thrombolysis 9 (32 %) patients being this treatment considerate as efective in 1 (11 %) patient, reporting up to 3 cases (33 %) of bleeding in CT control. In 19 patients thrombolysis was not applied and the causes were: thrombolysis contraindicated (4 cases), with out indication of thrombolysis (4 cases) and improvement of disability (11 cases).

During the first trimester of this year we had 18 patients, 6 women (33 %) and 12 men (67 %) with a medium age of 63.6 y.o.; thrombolysis was appplied in 10 patients (55 %), being efective the treatment in 3 (30 %) cases with no one case of bleeding in CT control. And in the 8 patients in whom thrombolysis was not applied the causes were: thrombolysis contraindicated and with out indication of thrombolysis with 4 cases for every group. Improvement of disability is not considerated as cause of no thrombolysis.

**Conclusions** The new recomendations in acute ischemic stroke and the advances in neuroradiology allow a better approach of patients, is increasing the patients whom benefits of this treatment, and the effectiveness seems to increase, in the same way the adverse results seem to remain in low cases.

### A124 Conduction of passive verticalization in patients with ischemic stroke who are on mechanical ventilation

#### A. Gritsan^1^, A. Gazenkampf^1^, E. Korchagin^2^, N. Dovbish^3^

##### ^1^Krasnoyarsk State Medical University, Krasnoyarsk Regional Hospital, Anaesthesiology and Intensive Care, Krasnoyarsk, Russian Federation; ^2^Krasnoyarsk Regional Hospital, Krasnoyarsk, Russian Federation; ^3^Krasnoyarsk Clinical Regional Hospital, Anaesthesiology and Intensive Care, Krasnoyarsk, Russian Federation

###### **Correspondence:** A. Gritsan – Krasnoyarsk State Medical University, Krasnoyarsk Regional Hospital, Anaesthesiology and Intensive Care, Krasnoyarsk, Russian Federation

**Introduction** Acute ischemic stroke is a leader in terms of mortality and disability. Early rehabilitation significantly improves the outcome of treatment. One of the most effective methods of early rehabilitation is passive verticalization, aimed at prevention and elimination of complications associated with long-stay patient in the supine position.

One of the relative contraindications for verticalization is to carry out mechanical ventilation (AV).

**Objectives** To assess the effectiveness of early passive verticalization conducted against the background of mechanical ventilation in patients with ischemic stroke.

**Methods** The analysis of 55 and medical history of patients with a diagnosis - ischemic stroke. It formed two groups - the addition of verticalization (Group 1 - 32 persons) and without a verticalization (Group 2 - 23 people). All patients were treated according to the Recommendations AHA / ASA 2013. All patients underwent mechanical ventilation respirators "Hamilton C2 " (Switzerland). In verticalization carried out by verticalization table «Beka Hospital» (Germany). All patients conducted severity rating scales for SOFA, NIHHS, CGS at admission and on the first, 3rd, 5th, 7th, 10th. At these stages were assessed respiratory status.

**Results** In both groups assessed results are presented in Table 48. Starting ventilator patients in both groups were required at different times substantially on average, and the eighth day in both groups were diagnosed with pneumonia.The frequency of hemodynamic disturbances and duration of inotropic support were comparable in both groups. The mortality rate in group 1 was significantly lower than in group 2, which explains the increase in the period of mechanical ventilation and stay of patients in the ICU and in the hospital. Analysis on GOS the surviving patients showed no significant differences.

## Conclusions

1. The use of early passive verticalization in patients with ischemic stroke on the background of mechanical ventilation does not lead to a deterioration in the neurological and somatic status.

2. Applying early verticalization in patients with ischemic stroke significantly reduces mortality and increases the time of mechanical ventilation, the patient´s stay in the intensive care ward and in the hospital.

**Table 48 (abstract A124).** The results of treatment (M ± m)

Indicators	Group 1	Group 2	P
Starting MV, day	2.8±0.6	2.8±0.8	>0.05
Ventilator days	34.4±4.3	19.1±3.3	<0.05
Staying in ICU, day	46.2±4.9	35.1±4.9	>0.05
Pneumonia,abs (%) Diagnosis (day of hospitalization)	10 (31.2) 8±1.5	8 (34.8) 8±3.4	>0.05 >0.05
Vasopressor, abs(%) Duration of treat., day	6 (18.8) 66.6±13.4	9 (39.1) 47.6±5.8	>0.05 >0.05
Daeths, abs (%)	12 (37.5)	18 (78.3)	<0.01
COS, ball	3.5±0.3	3.4±0.4	>0.05
28-th day: - Died, abs (%)	0	5 (21.7)	<0.01
- Alive, he is in the ICU , abs (%)	17 (53.1)	7 (30.5)	>0.05

### A125 Comparison of the efficacy of oral oxycodone and oral codeine in the treatment of post-craniotomy pain

#### R.M. Lee, M.P.P. Lim, C.T. Chong, B.C.L. Lim, J.J. See

##### Tan Tock Seng Hospital, National Healthcare Group, Singapore, Singapore

###### **Correspondence:** R.M. Lee – Tan Tock Seng Hospital, National Healthcare Group, Singapore, Singapore

**Introduction** Post-craniotomy pain has been reported to be moderate to severe. Management of post-craniotomy pain is inadequate in many cases, yet is limited by the side effects of opioids. Codeine has been the mainstay of treatment of post-craniotomy pain in our institution, due to its safer side effect profile when compared to more potent opioids. However, its effectiveness may be limited due to the need to be de-methylated before it has any analgesic effect and this process is subject to inter-individual variability.

**Objectives** Our primary objective was to determine if there is a difference in the mean pain VAS scores in the oxycodone and codeine groups at 24 hours. Secondary objectives were to compare pain VAS scores at 48 and 72 hours and to compare the incidence of excessive sedation, depression of respiratory rate and GCS.

**Methods** A randomized, double blinded controlled trial was used to evaluate the efficacy of oral oxycodone versus oral codeine. 40 patients were randomized to the control group of codeine (n = 20) or the experimental group receiving oxycodone (n = 20), in addition to regular oral paracetamol for both groups of patients. Analgesia was to be administered according to a strict protocol. Patients were reviewed by blinded assessors closely in the 1^st^ day and then subsequently once a day at the 48^th^ and 72^nd^ hour post-operatively.

**Results** A total of 36 patients were analysed (4 patients dropped out due to post-surgical complications). The mean pain score at 24 hours was 1.85 ± 1.60 and 2.78 ± 1.92 (p = 0.110) in the codeine and oxycodone group respectively. There were also no statistically significant difference in the sedation scores, respiratory rate and GCS scores.

**Conclusions** Oral oxycodone is as effective as oral codeine in the management of post-craniotomy pain.

Our local population also seemingly has very mild pain after a craniotomy (mean pain VAS scores 1-3), as compared to what was reported in the literature. One deduction is that compared to the Western population, our population probably has minimal genetic variability in the ability to metabolise codeine. Our population may all be efficient metabolisers, thus allowing codeine to be as effective as oxycodone. Also, as our patients generally had mild pain, codeine may be adequate analgesia for them.

As there is no difference in adverse effects, oxycodone may also be as safe as codeine, in bioequivalent doses.

Hence, oxycodone can be considered as an effective alternative to codeine.

### A126 Early and 60-days mortality and its causes in patients undergoing intravenous thrombolysis for ischemic stroke

#### R. Assis^1^, F. Filipe^2^, N. Lopes^3^, L. Pessoa^3^, T. Pereira^3^, N. Catorze^3^

##### ^1^Centro Hospitalar do Médio Tejo, Unidade de Cuidados Intensivos Polivalente, Abrantes, Portugal; ^2^Hospital de Santa Maria/CHLN, Lisboa, Portugal, ^3^Centro Hospitalar do Médio Tejo, Abrantes, Portugal

###### **Correspondence:** R. Assis – Centro Hospitalar do Médio Tejo, Unidade de Cuidados Intensivos Polivalente, Abrantes, Portugal

**Introduction** Ischemic Stroke still constitutes the major cause of death in Portugal. With the widespread investment in information to the population and creation of a net of reference hospitals with established protocols of Intravenous Fibrinolysis and endovascular treatment, mortality and morbidity have declined.

**Objectives** The objective of this study is characterize the population undergoing Intravenous Fibrinolysis with alteplase at the Intensive Care Unit of the researchers' hospital, verify the early (48 hours) and 60-days mortality after admission,and identify the causes of death.

**Methods** Observational retrospective study based on information acquired from the clinical records of patients admitted in the Intensive Care Unit for Intravenous Thrombolysis between the 1^st^ of January 2010 and 31^st^ December 2015, and its statistical analysis.

**Results** Among the total of patients included (n = 102), 63 % were male with an average of 70 years old.The average admission National Institutes of Health Stroke Scale (NIHSS) score was 14 and the average Symptoms-to-Needle time was 156 minutes. There was a total of 4 deaths in the first 48 h after admission and 19 deaths between 48 hours and 60 days post admission. Of the early deaths, 2 were due to intracerebral hemorrhage and the other 2 accounted for progression of ischemic disease, unresponsive to thrombolysis. The highest cause of death at 60 days was Aspiration Pneumonia (10), followed by progression of ischemic disease (5), Intracerebral Hemorrhage (2), Septic Shock (1) and 1 prehospital death, with no reference to cause of death on clinical records. The average NIHSS score at twelve hours of patients who died between 48 hours and 60 days was 20.

**Conclusions** With the application of adequate guidelines and evaluation of the patients proposed to Fibrinolysis, early deaths accounted for 17 % of total deaths and were attributed to non effectiveness of fibrinolytic therapy or its hemorrhagic complications. Deaths occurring at 60 days post admission occurred in patients with higher NIHSS scores, revealing important neurological dysfunction. The most frequent cause of death was Aspiration Pneumonia. Being so, it is important to apply prevention measures to patients during ICU and hospital stay, in order to reduce

Aspiration Pneumonia's incidence and allow the patient to start physiotherapy as soon as possible to regain lost functionality. Patients suffering stroke should be evaluated by a multidisciplinary team involving Neurology, Internal Medicine and Physiotherapy at regular periods to adequately assist them to resume their lives.

**References**

1. H Brønnum-Hansen, et al, Stroke. 2001; 32: 2131-2136

2. E Golestanian Crit Care Med. 2009 Dec;37(12):3107-13

### A127 Effects of propofol and midazolam on motor coordination and analgesia: a comparative analysis

#### M.S. Aydogan

##### Inonu University, Intensive Care, Malatya, Turkey

**Introduction** Propofol and midazolam are known to be excellent drugs for sedation for intensive care units.

**Objectives** This study aimed to compare the sedative and analgesic effects and the recovery profiles of propofol and midazolam in a rat model by conducting motor coordination tests (rotarod-accelerod test) and by evaluating the analgesic response times by conducting hot plate and tail flick tests.

**Methods** Rats were randomly divided into the following 4 groups on the basis of the treatment received. The first group received 600 μg/kg/min propofol, the second, 83 μg/kg^/^min midazolam; and the third, 83 μg/kg^/^min morphine; the fourth was a control group. The rats were placed on a rotating rod and tested first at the slowest speed (5 rpm), followed by a speed of 10 rpm, and then with 10-rpm speed increments at speeds up to 40 rpm. The speed was set to increase from 1 to 79 rpm within 4 and 10 min in the accelerod test. Pain reflexes in response to a thermal stimulus were measured at 0, 10, 20, and 60 min after the drug injection by using the hot plate and tail flick tests. The neurobehavioral status, including sensory and motor function, was assessed every 30 min until normal functioning resumed by an investigator who was blinded to the groups.

**Results** At all the tested speeds, the midazolam-injected rats remained on the rotarod longer than did the propofol-injected rats. Furthermore, in the 10 min accelerod test, the midazolam-injected rats remained for a longer duration than did the propofol-injected rats. The latency time for the hot plate test was significantly higher at 10 min in the propofol group than in the midazolam group. At 10 and 20 min, the latency time was greater in the propofol group than in the midazolam group. Further, the latency time at 10 min for the tail flick test was greater in the propofol group than in the midazolam group. Propofol enhanced sensory blockade to a greater extent at 90 and 120 min than midazolam did at the corresponding time points. Further, the duration of complete motor blockade was significantly greater in the propofol group than in the midazolam group.

**Conclusions** For achieving a long-term analgesic benefit, propofol treatment may be more effective than midazolam treatment. Propofol may be preferable sedation applications in intensive care units as it affords faster onset of recovery of motor coordination performance.

**References**

1. Bhana N, Goa KL, McClellan KJ. Dexmedetomidine. Drugs 2000; 59: 263-268.

2. Bittner B, González RC, Isel H, Flament C. Impact of Solutol HS 15 on the pharmacokinetic behavior of midazolam upon intravenous administration to male Wistar rats. Eur J Pharm Biopharm. 2003;56(1):143-6.

### A128 Neuroprotective effects of ranolazine by preventing apoptosis and necrosis induced by Aβ_1-42_

#### C. Aldasoro^1,2^, P. Marchio^1^, A. Jorda^1^, M.D. Mauricio^1^, S. Guerra-Ojeda^1^, M. Gimeno-Raga^1^, M. Colque-Cano^1^, A. Bertomeu-Artecero^1^, M. Aldasoro^1^, S.L. Valles^1^

##### ^1^University of Valencia/School of Medicine, Valencia, Spain; ^2^Hospital General Universitario de Castellon, Castellon, Spain

###### **Correspondence:** C. Aldasoro – University of Valencia/School of Medicine, Valencia, Spain

**Introduction** Ranolazine (Rn), a drug used for the treatment of chronic angina pectoris (1), has been proposed for the management of epileptic disorders for its ability to decrease neuronal excitability by blocking late inward sodium current (late INa) in the central nervous system (2, 3). We recently demonstrated in primary cultures that Rn could act as a neuroprotective drug by promoting astrocyte viability, preventing necrosis and apoptosis, inhibiting inflammatory phenomena and inducing anti-inflammatory and antioxidant agents (4). Amyloid-β peptide 1-42 (Aβ_1-42_), a protein involved in the pathogenesis of Alzheimer´s disease, produce glial activation, inflammatory response and oxidative stress that can lead to neuronal death (5).

**Objectives** Under the hypothesis that ranolazine acts as a neuroprotective drug, the present study focuses on the effects ranolazine on astrocytes exposed to Aβ_1-42_ toxic peptide.

**Methods** We incubated rat astrocytes in primary cultures for 24 hours with Rn (10^−7^, 10^−6^ and 10^−5^ M), Aβ_1-42_ (15 μM) or Rn (10^−6^ M) + Aβ_1-42_ (15 μM). Cell viability and proliferation were measured using MTT conversion assay and LDH release assay. Apoptosis was determined by Caspase 3 activity assay.

**Results** In cultured astrocytes, Rn significantly increased cell viability and proliferation at any concentration tested, and decreased LDH leakage and Caspase 3 activity indicating less cell death. Aβ_1-42_ significantly decreased cell viability compared to control astrocytes. Incubation with Rn (10^−6^ M) prevented the decrease in cell viability induced by Aβ_1-42_. Rn decreased LDH release to the medium (15 % with Rn 10^-6^M and 20 % with Rn 10^-5^). Toxic peptide increased LDH release in about 75 % and incubation with Rn (10^-6^) lowered by 60 % LDH levels, indicating a protective effect against Aβ_1-42_.

**Conclusions** Ranolazine increases cell viability and prevents necrosis and apoptosis induced by Aβ_1-42_, suggesting that Rn could act as a neuroprotective drug in situations associated with oxidative stress or inflammation.

**References**

(1) Belardinelli L et al: J Pharmacol Exp Ther. 2013; 344:23-32.

(2) Park YY et al: J Neurophysiol. 2013; 109: 1378-1390.

(3) Peters CH et al: Br J Pharmacol. 2013; 169: 704-716.

(4) Vallés SL et al: Aging Cell. 2008; 7: 112-118.

(5) Aldasoro M et al: PLoS One. 2016 Mar 7;11(3):e0150619.

### A129 Functional isotope imaging evaluation of terutroban efficiency in a proinflammatory rat model of subarachnoid haemorrhage (SAH)

#### D. Tonon^1^, T. Triglia^1^, J.-C. Martin^2^, M.-C. Alessi^3^, N. Bruder^1^, P. Garrigue^4^, L. Velly^1^

##### ^1^APHM, CHU Timone, Dept of Anaesthesiology & Intensive Care, Marseille, France; ^2^University School of Medicine, NORT Laboratory, INSERM 1062 INRA 1260, Marseille, France; ^3^APHM, CHU Timone, Laboratoire NORT INSERM U 1062 INRA U 1260, Marseille, France; ^4^APHM, CHU Timone, Vascular Research Center of Marseille INSERM UMRS 1076, CERIMED, Marseille, France

###### **Correspondence:** D. Tonon – APHM, CHU Timone, Dept of Anaesthesiology & Intensive Care, Marseille, France

**Introduction** Delayed cerebral ischaemia (DCI) (1) is the first cause of morbidity after subarachnoid haemorrhage (SAH). F2isoprostanes and eicosanoids were found in the cerebrospinal fluid (CSF) of patients with DCI. These potent vasoconstrictors induce platelet aggregation and mediate inflammation by a thromboxaneprostaglandine (TP) receptor binding (2).

**Objectives** The aim of our study was first to estimate the occurrence of DCI in a proinflammatory state using an high omega 6 polyunsaturated fatty acid (w6) diet and secondly to evaluate the efficiency of terutroban (TER) a TP receptor inhibitor.

**Methods** Ninety wistar rats (400 g) were randomly assigned to one of 5 groups: a double 250 μL intracisternal injection of autologous arterial blood (SAH groups) (3) or artificial CSF (CSF group) was performed. To induce a proinflammatory state animals were fat with w6 during 6 weeks before SAH procedure (SAH_w6/SAH_w6 + TER). TER was administered (30 mg/kg/day) during 5 days following SAH (SAH + TER/SAH_w6 + TER groups). Evaluation of uptakes of 3 [99mTc]radiolabeled agents was achieved using microSPECT/CT imaging: HMPAO at D5 for cerebral perfusion quantification; DTPA at D3 for blood brain barrier (BBB) integrity study; and AnnexinV at D4 for apoptotic activity study. ANOVA followed by Student ´s t test.

**Results** HMPAO uptake analysis showed a significant decrease in the SAH group (figure). DTPA and AnnexinV uptake were also significantly increased in the SAH group compare to the CSF group. Proinflammatory state before SAH dramatically decreased HMPAO uptake (figure); increased DTPA (0.37 ± 0.04 vs. 0.43 ± 0.01 Mbeq/mm3; P < 0.05) and AnnexinV (0.39 ± 0.03 vs. 0.48 ± 0.03 Mbeq/mm3; P < 0.05). TER significantly counteracted the decrease in HMPAO uptake (figure) and the increase in DTPA uptake (P < 0.05) and in AnnexinV uptake (P < 0.001) induced by SAH.

**Conclusions** For the first time, a proinflammatory SAH rat model of DCI has been described. microSPECT study shows that a proinflammatory diet dramatically increases apoptosis and DCI. TER improved hypoperfusion, BBB disruption and apoptosis. TP receptor antagonists could be promising treatments after SAH.

**References**

1. Vergouwen, M. D. I. et al. Cerebral Infarction After Subarachnoid Hemorrhage Contributes to Poor Outcome by Vasospasm-Dependent and -Independent Effects. **Stroke** 42, 924-929 (2011).

2. Félétou, M. et al. The thromboxane/endoperoxide receptor (TP): the common villain. **J. Cardiovasc. Pharmacol.** 55, 317-332 (2010).

3. Dusick, J. R. et al. A minimally-invasive rat model of subarachnoid hemorrhage and delayed ischemic injury. **Surg. Neurol. Int.** 2, 99 (2011).

**Grant acknowledgement**

The authors gratefully acknowledge use of the services and facilities of the NORT Laboratory (INSERM U1062 INRA U1260)

**Fig. 49 (abstract A129).**
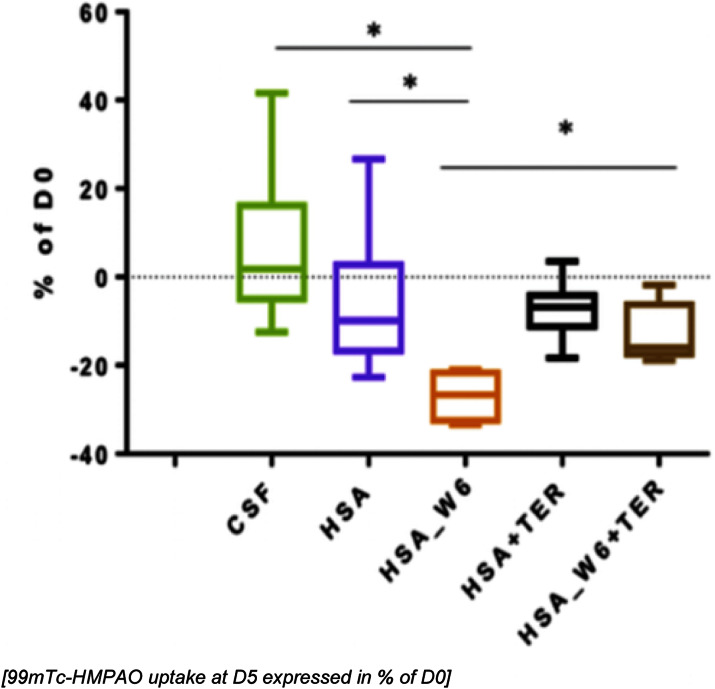
99mTc-HMPAO uptake at D5 expressed in % of D0

### A130 Tracheostomy in brain injured patients: a single center retrospective study on 170 consecutive patients

#### S. Spina^1^, V. Scaravilli^1^, C. Marzorati^1^, E. Colombo^2^, D. Savo^2^, A. Vargiolu^2^, G. Cavenaghi^3^, G. Citerio^1,2^

##### ^1^University of Milan - Bicocca, School of Medicine and Surgery, Milan, Italy; ^2^San Gerardo Hospital, Neurointensive Care, Department of Emergency and Intensive Care, Monza, Italy; ^3^San Gerardo Hospital, Department of Otolaryngology, Monza, Italy

###### **Correspondence:** S. Spina – University of Milan - Bicocca, School of Medicine and Surgery, Milan, Italy

**Introduction** Brain injured critically ill patients have often impaired airways reflexes and require long-term mechanical ventilation and tracheostomy, which is a standard of care. Many issues still remain unclear, for example the optimal timing (early versus late), the better technique and the effect on ICP in brain injured patients.

**Objectives** To describe the approach and the systemic/intracranial effects of tracheostomy in severe brain injured patients in a neurocritical intensive care unit (San Gerardo Hospital, Monza, Italy).

**Methods** All consecutive adult patients undergone tracheostomies from 2010 to 2015 were included. A retrospective analysis of prospectively-collected data retrieved from a digital PDM system was performed (demographics, procedures characteristics, and blood gas analyses, intracranial pressure (ICP), mean arterial pressure (MAP), cerebral perfusion pressure (CPP)). Data are reported as median (interquartile range) prior to/during/after tracheostomy. A repeated measures analysis of variance was utilized to assess the effects of tracheostomy.

**Results** Preliminary data are based on 170 patients (58 % male and 42 % female, 67 (56-73) years old, GCS at admission 7 (6-9)) admitted for intracranial hemorrhage (29 %), subarachnoid hemorrhage (22 %), trauma (21 %), stroke (12 %). Tracheostomy was performed at 10 (7-13) days from admission for compromised neurological status (89 %, GCS at tracheostomy 7 (6-8)). Direct laryngoscopy Fantoni´s translaryngeal technique (TLT), Percutwist, surgical, standard TLT and Dolphin were used in 63 %, 18 %, 11 %, 6 % and 2 % of the cases. ENT specialists and intenstivists performed 46 % and 54 % of the tracheostomy, respectively. No deleterious effect on recorded parameters was detected (see Table 48). Four lesions of the tracheal rings were documented.

**Conclusions** In a large cohort of brain-injured patients tracheostomy performed one week after the initial insult is safe.

**Table 49:** (abstract A130).

	Baseline (4 hours prior to tracheostomy)	Tracheostomy (Worse measurement during tracheostomy)	After (8 hours after tracheostomy)
ICP (mmHg)	8 (5 - 11)*	12 (7 - 17)	9 (6 - 11)*
MAP (mmHg)	83 (72 - 94)	84 (71 - 100)	80 (68,5 - 90)*
CPP (mmHg)	77 (65 - 87)	75,5 (60 - 91)	73 (62 - 81)
PaO2/FiO2 (mmHg)	345 (299,5 - 392)	-	351 (294 - 400)°
pH	7,43 (7,41 - 7,45)	-	7,43 (7,41 - 7,45)
PaCO2 (mmHg)	42,5 (39,5 - 46)	-	41,5 (38 - 45)°

*ICP* intracranial pressure, *MAP* mean arterial pressure, *CPP* cerebral perfusion pressure, *PaO2* arterial PO2, *FiO2* inspired fraction of oxygen, *PaCO2* arterial PCO2

*) p < 0.001 vs. Tracheostomy, °) p < 0.05 vs. Baseline.

### A131 Thrombolysis with intravenous alteplase in ischemic stroke with support from the neurologist telemedicina at a secondary hospital, experience - Municipal Hospital Moyses Deutsch - Sao Paulo Brazil

#### A.H.V. Andrade^1,2^, P. Bulgarelli^1,2^, J.A.P. Araujo^1,2^, V. Gonzalez^1,2^, V.A. Souza^1,2^, A. Costa^3^, C. Massant^3^, C.A.C. Abreu Filho^1,2^, R.A. Morbeck^4^, L.E. Burgo^5^

##### ^1^Hospital municipal Moyses Deutsch, ICU, Sao Paulo, Brazil; ^2^Hospital Israelita Albert Einstein, ICU, Sao Paulo, Brazil; ^3^Hospital Israelita Albert Einstein, Neurology/Telemedicine, Sao Paulo, Brazil; ^4^Hospital Israelita Albert Einstein, Telemedicine, Sao Paulo, Brazil; ^5^Hospital municipal Moyses Deutsch, Telemedicine, Sao Paulo, Brazil

###### **Correspondence:** A.H.V. Andrade – Hospital municipal Moyses Deutsch, ICU, Sao Paulo, Brazil

**Introduction** The paradigm for the treatment of ischemic stroke hyperacute changed in recent years with the Introduction of thrombolytic - Alteplase (rt-PA), the efficacy directly related to the time interval between the onset of symptoms and drug administration. Hospital Moyses Deutsch, in the southern city of São Paulo, is indicated for the use of rtPA intravenously in patients with ischemic stroke frame according to the protocol established by the service since 2011. It is necessary to ask neurological evaluation the distance trough of the Hospital Israelita Albert Einstein Telemedicine.

**Objectives** Demonstrate experience the use of intravenous alteplase clinical practice with Neurologist Telemedecine support, checking the efficiencies and procedure safety.

**Methods** Retrospective study, in all cases of ischemic stroke who received intravenous alteplase, from December 2011 to February 2016. The protocol indicates the use of alteplase for patients with inclusion criteria, the period between the onset of symptoms and hospital admission up to 4.5 hours and no contraindication to the use of thrombolytics, NIHSS (National Institutes of Health Scale course) calculated on admission and 24 hours after thrombolysis. Computed tomography (CT) on admission and after 24 hours. Solicitation evaluation required by neurological Telemedicine Hospital Israelita Albert Einstein shortly after the conclusion of the TC Cranio. The rt-PA dose of 0.9 mg / kg, 10 % of the remaining bolus dose continuous infusion over 1 hour to a maximum dose of 90 mg.

**Results** Alteplase was used in 64 patients with diagnosis of stroke ischemic. Evaluation Neurology Telemedicine trough has been triggered in 51 cases. Time between onset of symptoms and drug administration, 19 patients less than 90 minutes between 37 90 and 180 minutes, 8 patients between 180-360 minutes .The average NIHSS at admission was 18, with 42 patients showed a reduction of 5 or more the points NIHSS score within the first 24 hours. 8 patient non-symptomatic intracranial hemorrhage and 6 symptomatic intracranial hemorrhage and 9 deaths during the period.

**Conclusions** All patients receiving alteplase in the recommended time interval and underwent CT Cranio control. Some cases were not triggered by the evaluation of Neurology Telemedicine. There was improvement in NIHSS score similar percentage observed in reference studies. The protocol implementation has been adequate excellent support of Telemdicina Neurology team. Good profitability of time and therapeutic efficacy. The mortality that correlated with the severity of patients and the NIHSS admission. This data ratifies the safety of intravenous Alteplase use in the treatment of Ischaemic Stroke.

**Reference(s)**

The ECASS III trial: December 17, 2012,

Meyer BC, Raman R, Hemmen T, et al. Efficacy of site-independent telemedicine in the STRokE DOC trial:a randomised, blinded, prospective study.Lancet Neurol 2008;7

**Grant acknowledgement**

No conflicts of concern

## Sepsis, host response

### A132 Safety, tolerability, and immunomodulatory effects of EA-230 in humans

#### R. van Groenendael^1^, L.T. van Eijk^2^, G.P. Leijte^1^, B. Koeneman^1^, M. Kox^1^, P. Pickkers^1^

##### ^1^Radboud University Medical Center, Intensive Care, Nijmegen, Netherlands; ^2^Radboud University Medical Center, Anesthesiology, Nijmegen, Netherlands

###### **Correspondence:** R. van Groenendael – Radboud University Medical Center, Intensive Care, Nijmegen, Netherlands

**Introduction** The systemic inflammatory response syndrome (SIRS) can lead to pronounced tissue damage and is a frequent cause of multi-organ failure and mortality in Intensive Care units^1^. SIRS can be elicited by a variety of insults, such as sepsis, trauma, and major surgery, and no specific therapy is currently in routine use. EA-230 is a newly developed synthetic linear tetrapeptide derived from the β-human chorionic gonadotropin hormone (β-hCG), which has shown promising anti-inflammatory and tissue-protective effects in animal studies^2,3^.

**Objectives** To investigate the tolerability, safety and immunomodulatory effects of EA-230 in humans.

**Methods** We conducted a double blind, placebo controlled, dose-escalating randomized clinical trial in 60 healthy volunteers. The study was carried out in two phases. In the first phase (n = 24), safety and tolerability was established for escalating doses of EA-230 (30, 90, and 180 mg/kg). In the second phase (n = 36), the same doses were used to assess the effects of EA-230 on systemic inflammation during experimental human endotoxemia. At t = 0 hours, 2 ng/kg *E. Coli* endotoxin was administered i.v. followed by a 2-hour continuous i.v. infusion of EA-230 or placebo. Levels of circulating cytokines and adhesion molecules as well as body temperature and flu-like symptoms were assessed. Furthermore, effects on renal function were investigated using plasma clearance of iohexol.

**Fig. 50 (abstract A132).**
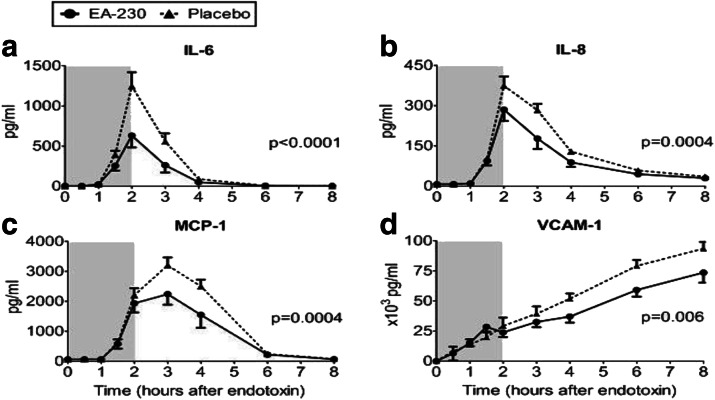
Plasma levels of inflammatory mediators and adhesion molecules during endotoxemia. (A) Interleukin-6, (B) Interleukin-8, (C) Monocyte Chemotactic Protein-1, (D) Vascular Cell Adhesion Molecule-1. Data are represented as means with SEM of n = 7 in the EA-230 180 mg/kg group and n = 12 in the placebo group. Gray box indicates the period in which the active group received EA-230. P-values between groups were calculated using repeated measures two-way analysis of variance (ANOVA, interaction term)

**Fig. 51 (abstract A132).**
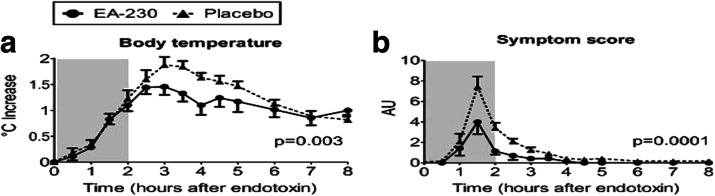
Body temperature (A) and symptom score (B) during endotoxemia. Data are represented as means with SEM of n = 7 in the EA-230 180 mg/kg group and n = 12 in the placebo group. Gray box indicates the period in which the active group received EA-230. P-values between groups were calculated using repeated measures two-way analysis of variance (ANOVA, interaction term)

**Results** EA-230 was well tolerated and showed an excellent safety profile. Treatment with the highest dose of EA-230, but not with lower doses, resulted in a significant attenuation of the endotoxin-induced increase in plasma levels of IL-6, IL-8, IL-1RA, MCP-1, MIP-1α, and MIP-1β (IL-6, IL-8, and MCP-1 shown in Fig. 50a, b, and c), and the adhesion molecule VCAM-1 (Fig. 50d). Furthermore, the highest dose of EA-230 reduced fever and flu-like symptoms (Fig. 51). Endotoxemia resulted in a marked increase in GFR, but no differences between groups were observed.

**Conclusion** Administration of EA-230 is safe and results in attenuation of the systemic inflammatory response in humans. These promising results pave the way for a phase IIb clinical trial to assess the anti-inflammatory and tissue-protective effects of EA-230 in patients.

**References**

1. Vincent JL, Sakr Y, Sprung CL, et al. Sepsis in European intensive care units: results of the SOAP study. Critical care medicine 2006;34:344-53.

2. Khan NA, Benner R. Human chorionic gonadotropin: a model molecule for oligopeptide-based drug discovery. Endocrine, metabolic & immune disorders drug targets 2011;11:32-53.

3. van den Berg HR, Khan NA, van der Zee M, et al. Synthetic oligopeptides related to the [beta]-subunit of human chorionic gonadotropin attenuate inflammation and liver damage after (trauma) hemorrhagic shock and resuscitation. Shock 2009;31:285-91.

### A133 Quartiles of immunoglobulin g concentrations and prognosis in patients with severe sepsis and septic shock

#### A. García-de la Torre^1^, M. de la Torre-Prados^2^, A. Fernández-Porcel^2^, C. Rueda-Molina^2^, P. Nuevo-Ortega^2^, T. Tsvetanova-Spasova^2^, E. Cámara-Sola^2^, A. García-Alcántara^2^, L. Salido-Díaz^2^

##### ^1^University Hospital Virgen de la Victoria / IBIMA, Clinical Chemistry Department, Málaga, Spain; ^2^University Hospital Virgen de la Victoria / IBIMA, Department of Intensive Care Unit, Málaga, Spain

###### **Correspondence:** M. de la Torre-Prados – University Hospital Virgen de la Victoria / IBIMA, Department of Intensive Care Unit, Málaga, Spain

**Introduction** Mortality from sepsis and septic shock remains high. Results of trials on intravenous immunoglobulins (IVIG) as adjunctive therapy for sepsis have showed controversies.

**Objectives** The aim of this study was to identify factors for predicting prognosis in patients with severe sepsis (SS) or septic shock (SSh) according to quartiles of immunoglobulin G (IgG) concentrations

**Methods** It is a cohort study of 133 critically ill adults admitted in a polivalent Intensive Care Unit of a University Hospital. Demographic data, clinical parameters and IgG levels were determinated within 24 hours from SS or SSh onset, defined according to Surviving Sepsis Campaign (SSC) criteria.

The patients were divided into four groups (quartiles) based on the 25th, 50th and 75th percentiles of their initial level of IgG. We tested for differences in baseline characteristics by IgG interval using a Kruskal-Wallis test for continuous data or a Chi Square test for categorical data and reported the median and interquartile ranges. A logistic regression model was adjusted for potential confounders as age, APACHE II score, SOFA score, number of organ failure (NOF) and presence of shock on admission. Statistical analysis was performed using SPSS 15.0 for Windows (SPSS Inc. Chicago, IL, USA)

**Results** We analyzed 133 consecutive episodes of SS (16.5 %) or ShS (83.5 %) admitted in the ICU. The median age of the study sample was 62 (inter-quartile range: 48.5-70.5) years old; male: 62.4 %. The main sources of infection were: respiratory tract 36.8 % and intra-abdomen 28.6 % and 69.9 % had medical pathology. 28-day mortality was 21.8 %.

Quartiles of serum IgG concentration were: quartile 1 (Q1: 607 mg/dL or less), quartile 2 (Q2: 607-792 mg/dL), quartile 3 (Q3: 792.1-976 mg/dL) and quartile 4 (Q4: 976.1 mg/dL or greater). The median IgG concentrations of each quartile were 525 mg/dL in Q1, 695 mg/dL in Q2, 881 mg/dL in Q3 and 1340 mg/dL in Q4, (p < 0.001). The differences between these quartiles shown no significant difference in APACHE II, SOFA score, number of organ failure (NOF) neither 28 day-mortality. Surprisingly the patients in Q4 had the higher 28-day mortality (30.3 %) compared with the other quartiles (OR 0.998, CI 0.996 to 1, P = 0.072), following by patients in Q1 (21.1 %).

**Conclusions** Our studied did not show prognostic value with low levels of serum IgG within 24 hours from SS or SSh onset. High levels of IgG within 24 hours from SS or SSh may be a risk factor for increase 28 day-mortality in septic patients; should be further investigated in this field.

**Reference(s)** Alejandria MM, Lansang MAD, Dans LF, Mantaring III JB. Intravenous immunoglobulin for treating sepsis, severe sepsis and septic shock (Review). The Cochrane Library 2013, Issue 9: 1-107

**Table 50 (abstract A133).** Characteristics Sever Sepsis/Septic Shock patients

	IGG <607 N=33	IGG= 607-792 N=33	IGG=792.1-976 N=34	IGG >976 N=33	p
Age (years)	61 [48-72.5]	60 [42-70]	65.5 [52.5-72.5]	65 [51-72]	ns
APACHE II	25 [19-30]	25 [20-28]	24 [21-30.25]	27 [21-31]	ns
Sepsis Related Organ Failure Assessment (SOFA)	9 [7-11]	9 [8-11.5]	9 [7.75-11]	10 [7-12]	ns
Number organs failure (NOF)	3 [3-5]	4 [3-4.5]	4 [3-4.25]	4 [2.5-4.5]	ns
IgG (mg/dL)	525 [457.5-571]	695 [655-731,5]	881 [833.5-926.5]	1340 [1125-1620]	<0.001
IgA (mg/dL)	145 [110-195]	190 [148-236]	222.5 [149.75-294.5]	385 [289.5-550]	<0.001
IgM (mg/dL)	51.1 [34-126.5]	47.5 [35-78.7]	97.3 [66.3-128]	111 [65.7-148.5]	0.006
28-day mortality (%)	21.2	18.2	17.6	30.3	ns
Septic Shock (%)	90.9	78.8	85.3	78.8	ns

### A134 Phenotypic changes and impaired function of peripheral γδT cells in patients with sepsis

#### X. Liao^1^, T. Feng^2^, J. Zhang^1^, X. Cao^1^, Q. Wu^2^, Z. Xie^1^, H. Li*^2^, Y. Kang*^1^

##### ^1^West China Hospital of Sichuan University, Chengdu, China; ^2^West China Second Hospital of Sichuan University, Chengdu, China

###### **Correspondence:** X. Liao – West China Hospital of Sichuan University, Chengdu, China

**Introduction** T lymphocytes play fundamental roles in the immunological response to sepsis. γδ T cells are a new subset of T lymphocytes that represent a small population of immune cells, exhibit features of both innate and adaptive immunity, and play an indispensable role in host defense, immune surveillance and homeostasis. Recent studies found massive loss of gamma delta T (γδT) cells in patients with sepsis. However, little did we know about their function changes and role in such a pathological status.

**Objectives** This study was designed to evaluate the phenotype and function of peripheral γδ T cells in patients with severe sepsis and septic shock, and its association with prognosis.

**Methods** This prospective observational study was conducted in three ICUs of a university hospital. A total of 107 patients, consecutively admitted and diagnosed with severe sepsis or septic shock (excluding previous immunosuppression) and 30 healthy controls, were enrolled. Surface markers (CD69, NKG2D, PD-1) and intracellular cytokines (IFN-γ, IL-17, IL-10, TGF-β) of γδT cells isolated from peripheral blood were analyzed by flow cytometry. Results were also correlated with clinical outcome.

**Results** Septic patients displayed decreased percentage of γδT cells and NKG2D expression, and increased CD69, pro- (IFN-γ, IL-17) and anti-inflammatory (IL-10, TGF-β) intracellular cytokines as compared to healthy controls. After stimulation of γδ T cells *in vitro* by pamidronate (PAM) or phorbol-myristate acetate (PMA) plus ionomycin, both CD69 expression and IFN-γ secretion significantly reduced in septic patients as compared to healthy controls, 19.041 ± 11.74 % vs. 52.47 ± 19.84 % and 23.66 ± 17.37 % vs. 70.47 ± 16.41 %, respectively, p < 0.001. Importantly, these decreased expressions were more pronounced in nonsurvivors as compared to survivors. Using multi-regression logistic method to adjust factors that impacted patient outcome, IFN-γ secretion after stimulation and SOFA score were independent risk factors associated with patient death, OR: 0.937 (95 % CI: 0.893-0.982) and 1.248 (95 % CI: 1.056-1.474), respectively, p < 0.05.

**Conclusions** Our results showed pronounced changes in γδT cell phenotype and function in septic patients. This finding provides novel insights into the role of γδT cells in sepsis.

**Reference(s)**

1. Andreu-Ballester JC, Tormo-Calandin C, Garcia-Ballesteros C, et al: **Association of gammadelta T cells with disease severity and mortality in septic patients**. *Clin Vaccine Immunol* 2013, **20**(5):738-746.

2. Matsushima A, Ogura H, Fujita K, et al: Early Activation of γδ T Lymphocytes in Patients with Severe Systemic Inflammatory Response Syndrome. Shock 2004, 22(1):11-15.

**Grant acknowledgement**

This work was supported by the National Natural Science Foundation of China (No. 81471848).

### A135 Analysis of endogenous substrates and inhibitors of Nitric Oxide Synthase (NOS) in sepsis

#### M.S. Winkler^1^, A. Nierhaus (equal)^2^, E. Mudersbach^3^, A. Bauer^1^, L. Robbe^1^, C. Zahrte^2^, E. Schwedhelm^3^, S. Kluge^3^, C. Zöllner^1^

##### ^1^University Medical Center Hamburg-Eppendorf, Department of Anaesthesiology, Hamburg, Germany; ^2^University Medical Center Hamburg-Eppendorf, Department of Intensive Care Medicine, Hamburg, Germany; ^3^University Medical Center Hamburg-Eppendorf, Institute of Clinical Pharmacology and Toxicology, Hamburg, Germany

###### **Correspondence:** M.S. Winkler – University Medical Center Hamburg-Eppendorf, Department of Anaesthesiology, Hamburg, Germany

**Introduction** Hallmarks for sepsis severity include loss of vascular and immunological homeostasis. NO is an important vasodilatator and loss of NO may contribute to impaired microcirculation. Moreover, NO is involved in protein modification, regulation of transcription factors and production of superoxide anions. L-Arginine (L-Arg) is substrate of NO producing synthase (NOS) and its homolog, L-homoarginine (h-Arg), is the competitive substrate. Asymmetric dimethylarginine (ADMA) is an endogenous NOS inhibitor (see reference).

**Objectives** Therefore we sought to investigate whether L-Arg, h-Arg or ADMA are altered in septic patients. In addition we analyzed mRNA expression levels of dimethlyarginin-dimethylamino-hydrolase 2 (DDAH2), the ADMA degrading enzyme in peripheral blood monocytes (PBMC) of sepsis patients.

**Methods** Blood from 129 sepsis patients and 25 healthy controls was drawn and analyzed. L-Arg, h-Arg and ADMA concentrations were measured by mass spectrometry. In peripheral blood monocytes DDAH2 mRNA expression was measured by quantitative PCR (qPCR). All parameters were correlated with Sequential Organ Failure Assessment Score (SOFA) score for sepsis severity.

**Results** We did not observe any difference of NOS substrate L-arg between controls and patients. In contrast the concentration of h-Arg in blood was significant decreased in patients (P < 0.01), whereas ADMA concentration was increased in patients (P < 0.01). Both h-Arg and ADMA concentrations were associated with diseases severity. Spearman-rank analysis revealed a positive association between SOFA score and blood levels of ADMA with rho of 0.25 (P < 0.01). A negative association of h-Arg with the SOFA score was determined, however this was not significant. In ROC analysis ADMA emerged as the most powerful indicator of organ dysfunction, followed by the SOFA score, whereby both parameters yielded almost identical AUCs. Furthermore, in PBMCs DDAH2 expression was decreased in patients and significant lower in patients with sepsis related organ dysfunction (P < 0.05).

**Conclusions** In blood of septic patients we found increased concentrations of the endogenous NOS inhibitor ADMA together with decreased mRNA expression for DDAH2 in PBMCs. These measurements may influence systemic and intracellular NO levels in patients with sepsis and may contribute to the septic phenotype of microcirculatory and immunological collapse during sepsis. Decreased concentration of h-Arg the competitive substrate of NOS may be a reaction to restore NO equilibrium during sepsis. Further prospective studies are needed to confirm the results in a larger cohort of non-septic patients and controls.

**Reference(s)**

Bogdan C, Nat Immunol. 2001 Oct;2(10):907-16.

Leiper J, Arterioscler Thromb Vasc Biol. 2015 Jun;35(6):1382-92.

Lupp C, Crit Care. 2013 May 29;17(3):311.

**Grant acknowledgement**

None.

### A136 Augmented passive immunotherapy with P4 peptide improves phagocyte activity in severe sepsis

#### B. Morton^1^, E. Mitsi^1^, S.H. Pennington^1^, J. Reine^1^, A.D. Wright^1^, R. Parker^2^, I.D. Welters^3^, J.D. Blakey^1^, G. Rajam^4^, E.W. Ades^4^, D.M. Ferreira^1^, D. Wang^1^, A. Kadioglu^5^, S.B. Gordon^1^

##### ^1^Liverpool School of Tropical Medicine, Clinical Sciences, Liverpool, United Kingdom; ^2^Aintree University Hospital NHS Foundation Trust, Critical Care, Liverpool, United Kingdom; ^3^University of Liverpool, Institute of Ageing and Chronic Disease, Liverpool, United Kingdom; ^4^Centers for Disease Control, Bacterial Infection, Atlanta, United States; ^5^University of Liverpool, Institute of Infection & Global Health, Liverpool, United Kingdom

###### **Correspondence:** B. Morton – Liverpool School of Tropical Medicine, Clinical Sciences, Liverpool, United Kingdom

**Introduction** Antimicrobial resistance threatens to undermine treatment for severe infection; new therapeutic strategies are urgently needed. Augmented passive immunotherapy with P4 peptide is a novel therapeutic strategy that increases phagocytic activity *in vitro* and rescues moribund septic mice.

**Objectives** Our aim was to determine *ex vivo* P4 activity in a target population of patients admitted to critical care with severe infection.

**Methods** We prospectively recruited UK critical care unit patients with severe sepsis and observed clinical course (≥3 months post discharge). Blood samples were taken in early (≤48 hrs post-diagnosis, n = 54), latent (seven days post-diagnosis, n = 39) and convalescent (3-6 months post-diagnosis, n = 18) phases of disease. The primary outcome measure was killing of opsonised *S.pneumoniae* by neutrophils with and without P4 peptide stimulation. We also used a flow cytometric whole blood phagocytosis assay to determine phagocyte association and oxidation of intraphagosomal reporter beads.

**Results** P4 peptide increased neutrophil killing of opsonised pneumococci by 8.6 % (C.I. 6.35 - 10.76, p < 0.001) in all phases of sepsis, independent of infection source and microbiological status. This represented a 54.9 % increase in bacterial killing compared to unstimulated neutrophils (15.6 %) in early phase samples. Similarly, P4 peptide treatment significantly increased neutrophil and monocyte intraphagosomal reporter bead association and oxidation, independent of infection source.

**Conclusions** We have extended in vitro and mice models work to demonstrate P4 peptide significantly increases phagocytosis and bacterial killing in samples from a target patient population with severe sepsis. This study supports the rationale for augmented passive immunotherapy as a therapeutic strategy in severe sepsis.

**Grant acknowledgement**

This work was supported by a Medical Research Council (UK) Confidence in Concept Award.

### A137 Endotoxin-induced immune suppression does not influence the local immune response induced by a subsequent challenge with live-attenuated influenza vaccine in humans *in vivo*

#### R. Koch^1^, M. Kox^1^, J. Rahamat-Langedoen^2^, J. Schloesser^3^, M. de Jonge^4^, P. Pickkers^1^

##### ^1^Radboud University Medical Center, Intensive Care, Nijmegen, Netherlands; ^2^Radboud University Medical Center, Virology, Nijmegen, Netherlands; ^3^NIZO, Ede, Netherlands, ^4^Radboud University Medical Center, Pediatrics, Nijmegen, Netherlands

###### **Correspondence:** R. Koch – Radboud University Medical Center, Intensive Care, Nijmegen, Netherlands

**Introduction** It is well-known that bacterial sepsis induces an immunosuppressed state, impairing the host's ability to clear the primary infection and increasing vulnerability for secondary bacterial or fungal infections[1]. However, whether bacterial sepsis affects the subsequent response to influenza infection is unknown, as is the safety of administration of live attenuated influenza vaccines (LAIVs) in immunosuppressed patients. Experimental human endotoxemia induces systemic inflammation that mimics bacterial sepsis and subsequent development of immune suppresion[2].

**Objectives** To investigate the effects of human endotoxemia on the immune response elicited by a subsequent challenge with the LAIV Fluenz (surrogate for influenza infection[3]).

**Methods** In a randomized, placebo-controlled study 30 healthy male subjects received intravenous placebo (NaCl, n = 15) or endotoxin (*E. coli* LPS 2 ng/kg, n = 15), followed by administration of quadrivalent Fluenz on day 7 in all subjects. Nasal wash samples were obtained to measure viral shedding and inflammatory mediators. Symptoms were recorded daily using an online symptom diary.

**Fig. 52 (abstract A137).**
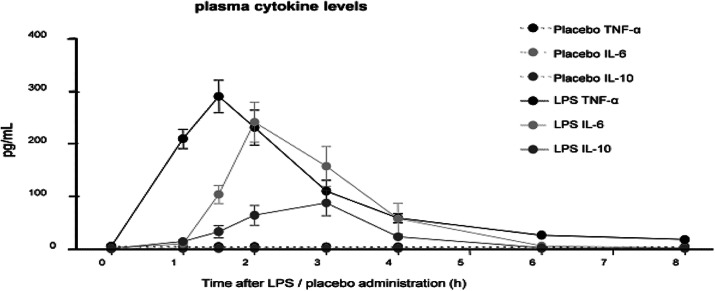
Plasma levels of the cytokines TNF-α, IL-6 and IL-10 over time in subjects that received an intravenous administration of endotoxin or placebo at T = 0. Data are represented as mean with SEM

**Fig. 53 (abstract A137).**
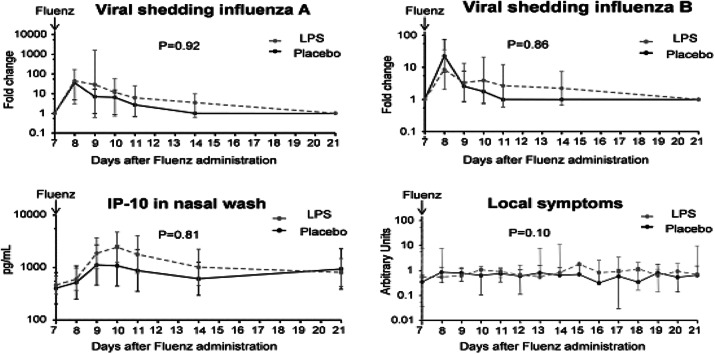
Viral shedding in nasal wash of the influenza A and B strain (upper panels), levels of IP-10 in nasal wash (lower left panel) and local symptoms (lower right panel) following intranasal administration of Fluenz in subjects that were administered LPS or placebo one week before. Data are presented as Geometric means with 95 % CI. P-values are calculated on area under curves using Man Whitney U t-tests

**Results** LPS administration resulted in a typical increase in plasma levels of cytokines, which was absent in the placebo group (Fig. 52). Following Fluenz challenge, viral shedding for at least one of the four influenza strains present in the vaccine was 12/15 (80 %) in the LPS-Fluenz group compared with 13/15 (87 %) in the placebo-Fluenz group. The increase in viral shedding of the influenza A and B strains was similar between groups (Fig. 53, upper panels). Likewise, the Fluenz-induced increase in levels of the chemokine IP-10 in nasal wash, as well as local symptoms, were not different between the LPS-Fluenz and placebo-Fluenz group (Fig. 53, lower panels).

**Conclusions** While human endotoxemia attenuates the inflammatory response of a second challenge with endotoxin with approximately 70 %[2], it does not influence the Fluenz-induced local immune response and viral shedding. These data suggest that the immune response to a bacterial compound does not alter the response to a subsequent viral infection.

**References**

1. Hotchkiss RS, Monneret G, Payen D, (2013) Immunosuppression in sepsis: a novel understanding of the disorder and a new therapeutic approach. The Lancet infectious diseases 13: 260-268

2. Leentjens J, Kox M, Koch RM, Preijers F, Joosten LA, van der Hoeven JG, Netea MG, Pickkers P, (2012) Reversal of immunoparalysis in humans in vivo: a double-blind, placebo-controlled, randomized pilot study. Am J Respir Crit Care Med 186: 838-845

3. Carter NJ, Curran MP, (2011) Live attenuated influenza vaccine (FluMist(R); Fluenz): a review of its use in the prevention of seasonal influenza in children and adults. Drugs 71: 1591-1622

**Grant acknowledgement**

This work was part of the Immunoforce project, supported by an EFRO grant (2011-013287).

### A138 Effect of methotrexate in a murine model of sepsis

#### J. Bringue^1^, R. Guillamat-Prats^2^, E. Torrents^3^, M.L. Martinez^3^, M. Camprubí-Rimblas^1^, A. Artigas^2,3^, L. Blanch^1,2^

##### ^1^Fundació Parc Taulí, Sabadell, Spain; ^2^CIBERES, Grup 33, Sabadell, Spain; ^3^Corporació Sanitària i Universitaria Parc Taulí, Critical Care Center, Sabadell, Spain

###### **Correspondence:** J. Bringue – Fundació Parc Taulí, Sabadell, Spain

**Introduction** Sepsis is a severe infection with a hyperinflammatory response mediated by cytokines that can induce acute lung injury and multi-organ dysfunction. It is the most common cause of death in intensive care units and currently, there is no specific drug treatment for this disease. That is why new therapeutic alternatives are essential to be found^1^. Methotrexate (MTX) is an immunosuppressant currently used in autoimmune disease; it acts by decreasing lymphocyte proliferation and cytokines production^2^.

**Objectives** The aim of this study is to evaluate the effect of MTX in inflammation caused by sepsis, focusing on systemic and lung injury. Our main hypothesis is that treatment with MTX reduces damage and control the inflammatory response in both the lung and systemic level.

**Methods** Sepsis was induced by a cecal ligation and puncture (CLP) in Sprague-Dawley rats (300-325 g). 6 hours later we did a surgical source control and administered antibiotics, fluids and analgesics. In addition, we administered to one group MTX i.p. (2.5 mg/kg). 48 h later the animals were sacrificed and samples of lung tissue, bronchoalveolar lavage and blood were collected.

Groups:

- Sham
- Sham + MTX
- CLP
- CLP + MTX

Survival, Body weight and lung weight were mesured. Neutrophils, macrophages, lymphocytes and protein concentration in bronchoalveolar lavage were quantified. Different T cells subsets in blood were analysed. Molecular markers related with inflammation, infiltration and damage were evaluated by qRT-PCR in lung tissue.

**Fig. 54 (abstract A138).**
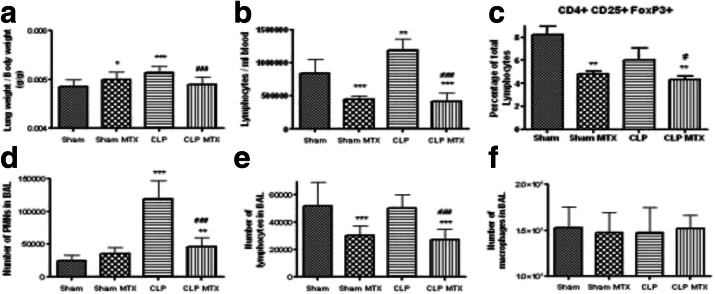
(A) Lung relative weight (B) Lymphocytes per ml of blood (C) Percentrage of Treg Lymphocytes (characterized by the expression of CD4, CD25, FoxP3 markers). (D)(E)(F) Total number of PMNs, Lymphocytes and Macrophages present in broncoalveolar lavage (BAL). All values are represented as mean±SEM. *p<0.05 vs Sham and #p<0.05 vs CLP. **/## p<0.01, ***/### p<0.001 (Sham and Tham MTX n=13, CLP and CLP MTX n=17)

**Results** Results show a decrease in circulating lymphocytes, Treg lymphocytes and lung weight in groups treated with MTX compared to the septic group (Fig. 54a-c). The cellular content in alveolus in the CLP MTX group shows a decrease in cell infiltration of polimorfonuclear cells and lymphocytes compared to CLP group, while the number of alveolar macrophages is not altered in the different groups (Fig. 54d-f).

**Fig. 55 (abstract A138).**
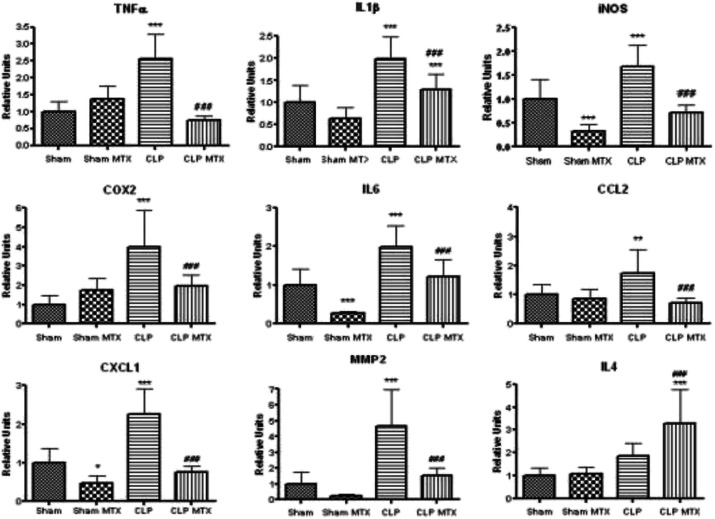
Relative expression of molecular markers in lung tissue homogenate corrected with GADPH as a control gen;calculated with RT-qPCR. All values are represented as mean±SEM. *p<0.05 vs Sham and #p<0.05 vs CLP. **/## p<0.01, ***/### p<0.001 (Sham and Tham MTX n=13, CLP and CLP MTX n=17)

Expression of the inflammatory cells recruitment, matrix remodelling and proinflmatory markers show an increase in septic rats, while with MTX treatment exhibit a reduction. Finally, MTX cause an increase of the expression of the anti-inflammatory cytokine IL4 (Fig. 55).

**Conclusions** MTX administration in an animal model of sepsis reduces systemic and lung injury causes by sepsis. This drug inhibits cytokine cascade and recruitment of pro-inflammatory cells in the lung.

**References**

1.Ware LB, Koyama T, Zhao Z, Janz DR, Wickersham N, Bernard GR, May AK, Calfee CS, Matthay MA Biomarkers of lung epithelial injury and inflammation distinguish severe sepsis patients with acute respiratory distress syndrome. *Crit Care*. 2013 Oct 24;17(5):R253

2.Tian H, Cronstein BN. Understanding the mechanisms of action of methotrexate: implications for the treatment of rheumatoid arthritis. *Bull NYU Hosp Jt Dis.* 2007;65(3):168-73.

**Grant acknowledgement**

CIR2015/009, Fundació Parc Taulí and CIBERES

### A139 Autophagy mediates neutrophil extracellular trap formation during sepsis

#### S.Y. Park^1^, Y.B. Park^2^, D.-K. Song^3^, S. Shrestha^3^, S.H. Park^4^, Y. Koh^5^, M.J. Park^4^, C.-W. Hong^6^

##### ^1^Kyung Hee University/Kyung Hee Medical Center, Pulmonary and Critical Care Medicine, Seoul, Republic of Korea; ^2^Division of Pulmonary, Allergy and Critical Care Medicine, Department of Internal Medicine, Kangdong Sacred Heart Hospital, Seoul, Republic of Korea; ^3^Hallym University, Department of Pharmacology, College of Medicine, Chuncheon, Republic of Korea; ^4^Kyung Hee University/Kyung Hee University Hospital at Gangdong, Seoul, Republic of Korea; ^5^University of Ulsan College of Medicine/ Asan Medical Center, Seoul, Republic of Korea; ^6^Kyungpook National University, Department of Physiology College of Medicine, Daegue, Republic of Korea

###### **Correspondence:** S.Y. Park – Kyung Hee University/Kyung Hee Medical Center, Pulmonary and Critical Care Medicine, Seoul, Republic of Korea

**Introduction** Neutrophils are key effectors in the host´s immune response to sepsis. Excessive stimulation or dysregulated functions of neutrophils are considered responsible for the pathogenesis of sepsis.

**Objectives** We report that neutrophil autophagy primes neutrophils for increased neutrophil extracellular trap (NET) formation, which is responsible for host survival during sepsis.

**Method** We studied patients with community-acquired pneumonia who have been admitted to the intensive care unit. To investigate this hypothesis, we isolated neutrophils from community-acquired pneumonia patients on day 1 (PD1) and day 3 (PD3). Then we determined the ROS generation, NETs Formation, surface expression of granule markers (CD63, CD66b, CD35.

**Results** Neutrophils isolated from septic patients expressed high levels of the autophagy-specific protein LC3 and primed neutrophils for NET formation in response to subsequent phorbol 12-myristate 13-acetate (PMA) stimulation. These neutrophils showed decreased mean lobe counts and distinct changes in surface marker expression (CD62L^dim^/CD64^brt^). In contrast, neutrophils isolated from non-surviving septic patients showed dysregulated autophagy and decreased responses to PMA stimulation. In a mouse model of sepsis, autophagy augmentation improved survival via a NET-dependent mechanism. Our study provides important insights into the role of autophagy in neutrophils during sepsis.

**Conclusion** Neutrophil autophagy could be an attractive therapeutic target for the treatment of sepsis.

**Reference(s)**

1. R. S. Hotchkiss, G. Monneret, D. Payen, Sepsis-induced immunosuppression: from cellular dysfunctions to immunotherapy, *Nat Rev Immunol* **13**, 862-874 (2013).

2. J.-L. Vincent, S. M. Opal, J. C. Marshall, K. J. Tracey, Sepsis definitions: time for change, *Lancet* **381**, 774-775 (2013).

3. R. S. Hotchkiss, I. E. Karl, The pathophysiology and treatment of sepsis, *N Engl J Med* **348**, 138-150 (2003).

4. R. S. Hotchkiss, E. R. Sherwood, Immunology. Getting sepsis therapy right, *Science* **347**, 1201-1202 (2015).

5. D. S. Weiss, Y. Weinrauch, A. Zychlinsky, Neutrophil extracellular traps kill bacteria, *Science* **303**, 1532-1535 (2004).

### A140 Elabela, a novel APJ receptor agonist, limits cardio-renal dysfunction and improves fluid homeostasis during experimental sepsis

#### O. Lesur^1^, D. Coquerel^1^, X. Sainsily^2^, J. Cote^2^, T. Söllradl^2^, A. Murza^2^, L. Dumont^2^, R. Dumaine^2^, M. Grandbois^2^, P. Sarret^2^, E. Marsault^2^, D. Salvail^3^, M. Auger-Messier^2^, F. Chagnon^1^, Apelin Group

##### ^1^Sherbrooke, ICU/Medicine, Sherbrooke, Canada; ^2^Sherbrooke, Pharmacology/Physiology, Sherbrooke, Canada; ^3^IPS Therapeutic Inc, Sherbrooke, Canada

###### **Correspondence:** O. Lesur – Sherbrooke, ICU/Medicine, Sherbrooke, Canada

**Introduction** Septic shock with Acute kidney injury are common in critically ill patients. The apelinergic system improves cardiac functions, decreases vascular tones and exhibits diuretic properties. These effects are held by two distinct endogenous peptides: Apelin-13 (APL-13) a dominant bioactive fragment of the apelin family mainly expressed in the cardiovascular system, and ELABELA (ELA) a recently discovered ligand of APJ-R described to regulate cardiovascular development and mainly expressed in kidney tissues.

**Objectives** ELABELA vs. APL-13 during sepsis-induced cardio-renal syndrome.

**Methods** Sepsis induced by cecal ligature and puncture (CLP) in adult rats. Myocardial impact of ELA vs. APL-13 on healthy and septic isolated-heart assessed by Langendorff apparatus *ex vivo*. Peptides and fluid resuscitation intra-venous infusions through osmotic and/or syringe pumps in healthy vs. CLP rats *in vivo*. Monitoring of myocardial functions by echocardiography, and *in vivo* left ventricular (LV) hemodynamics through pressure-volume probing 24 h after CLP induction. Water intake and urine output recording, heart; kidney and blood collection for subsequent biological assays.

**Results** ELA as well as APL-13 stimulated left ventricular ino- and lusitropy of healthy and septic hearts *ex vivo* (sepsis, dP/dtmax from baseline 10pM; APL-13: 92 %; ELA: 83 %). Both APL-13 and ELA also improved survival significantly 72 h after sepsis induction (survival, CLP: 0 %, CLP + APL13: 33 %,

p < 0.05 vs. CLP; CLP + ELA: 50 % p < 0.05 vs. CLP). CLP-induced myocardial dysfunction *in vivo* was counteracted by APL-13 and ELA infusion as shown by a higher cardiac index (CI: Sham 20.1 mL/min/100 g; CLP 8.4 p < 0.01 vs Sham; CLP + APL-13 17.9 p < 0.05 vs. CLP; CLP + ELA 20.5 p < 0.05 vs. CLP) and an improved left ventricular pressure-volume relationship. Nonetheless, ELA-treated CLP rats were the only ones

i) displaying a significant improvement of LV filling as shown by the increased E-wave velocity (Sham: 101; CLP: 68; CLP + APL-13: 82, CLP + ELA: 97 cm.s^-1^ p < 0.05 vs. CLP) and LV end-diastolic volume (Sham: 378; CLP: 264; CLP + APL-13: 333, CLP + ELA: 448 μL p < 0.05 vs. CLP),
ii) exhibiting a higher plasma volume (Sham: 40.2 ml.kg^-1^; CLP: 36.9; CLP + APL-13: 38.2; CLP + ELA: 41.4 p < 0.05 vs. CLP) and
iii) limiting CLP-induced drop of urine output with limitation of free water clearance. Moreover, ELA improved creatinine clearance and reduced KIM-1 & micro-albuminuria in CLP rats.

**Conclusions** Modulation of the apelinergic system by exogenous APL-13 or ELA infusion improves survival and cardiovascular dysfunction after sepsis induction. The recently discovered ELA displays an added-value by improving hemodynamics and limiting kidney dysfunction and injury. ELABELA is a potential new therapeutic drug to support cardio-renal dysfunction during sepsis.

**Reference(s)**

Murza A et al. J Med Chem. 2016. PIMD26986036

**Grant acknowledgement**

Heart&Stroke foundation, Merck-Dhome FMSS grant, CRSNG SEP, MITACS

### A141 Fever and mitochondrial uncoupling in sepsis

#### M.P. Lauretta^1^, E. Greco^2^, A. Dyson^2,3^, M. Singer^2^

##### ^1^Bloomsbury Institute of Intensive Care Medicine, University College London, Division of Medicine, London, United Kingdom; ^2^Bloomsbury Institute of Intensive Care Medicine, University College London, London, United Kingdom; ^3^Magnus Oxygen Ltd, London, United Kingdom

###### **Correspondence:** M.P. Lauretta – Bloomsbury Institute of Intensive Care Medicine, University College London, Division of Medicine, London, United Kingdom

**Introduction** The major sources of body heat production are muscular activity, oxidation of food, and uncoupled mitochondrial respiration due to proton leak. The cause of fever in sepsis is unknown, particularly in sedated, ventilated patients who neither perform much voluntary skeletal muscle activity nor shiver, and are often not fed in the acute early phase. Furthermore, the usual vasodilated state implies increased heat loss, so this must be exceeded by heat generation to generate a pyrexia.

**Objectives** To determine the association between fever and uncoupled mitochondrial respiration, by comparing the effects of exogenous uncoupling with dinitrophenol in sham and septic rats.

**Methods** Awake, male Wistar rats (approx. 300 g body weight), previously instrumented with tunnelled carotid and jugular lines, were placed in metabolic cages to continuously monitor whole body oxygen consumption (VO_2_). Core temperature was measured intermittently with a rectal probe. Sepsis was induced by i.p. injection of faecal slurry. Sham animals received i.p. saline. Intravenous fluid resuscitation (10 ml/kg/h crystalloid) was started at 2 h. At 6 and 24 h, animals received the mitochondrial uncoupler, dinitrophenol (DNP) (30 mg/kg), given over 1 h. Statistical differences comparing pre- and post-DNP were assessed using a paired T-test. P values < 0.05 were considered statistically significant.

**Results** Sham animals were euthermic at 6 and 24 h, and their VO_2_ was similar to baseline values. DNP produced significant rises in VO_2_ (20 % and 22 %, respectively) and temperature (>1 °C) at both timepoints (p < 0.05). By contrast, septic animals were febrile pre-DNP at 6 h (p < 0.05) but not 24 h, and DNP only induced small, non-significant rises in temperature. No change in VO_2_ was seen at either timepoint.

**Conclusions** Whereas sham animals generated an appropriate rise in temperature and VO_2_ in response to mitochondrial uncoupling with DNP, septic rats could only generate a small rise in temperature and a minimal VO_2_ response. This implies that mitochondrial uncoupling was already increased in septic rats (thus explaining the pyrexia) and could not be increased further by exogenous uncoupling. It is also possible that upstream inhibition of the electron transport chain may have prevented the response to DNP seen in septic animals. This possibility is under further investigation.

**Reference**

Rolfe DF, Brown GC. Physiol Rev. 1997; 77:731-58.

**Fig. 56:**
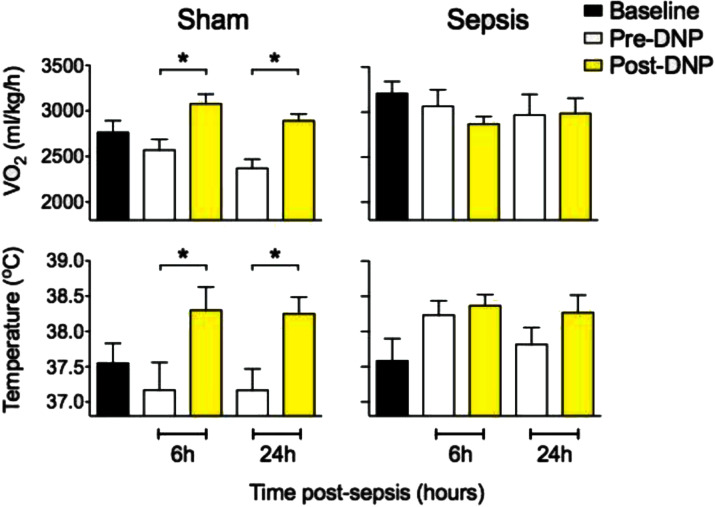
(abstract A141).

### A142 Ubiquitin-proteasome system involvement in critical illness acquired myopathy according to muscle type

#### S. Preau^1,2^, M. Ambler^1^, A. Sigurta^1^, S. Saeed^1^, M. Singer^1^

##### ^1^University College London, Faculty of Medical Sciences, London, United Kingdom; ^2^Lille Univ Hospital, Lille, France

###### Correspondence: S. Preau – University College London, Faculty of Medical Sciences, London, United Kingdom

**Introduction** Critical illness acquired myopathy in rats is characterized by homogeneous muscle atrophy (1). Conversely, histological abnormalities are heterogeneous among muscle types: oxidative muscles show patchy alterations (myofascitis, necrosis), while glycolytic types demonstrate normal patterns. Activation of the ubiquitin-proteasome system (UPS) is responsible for 80-100 % of myofibrillar protein breakdown in skeletal muscle. Whether UPS activation is dependent on the oxidative and glycolytic muscle type during critical illness is unknown.

**Objectives** To characterize UPS activation by skeletal muscle type in a long-term peritonitis model

**Methods** Male Wistar rats were followed for up to 2 weeks after intraperitoneal injection of the yeast cell wall constituent, zymosan or n-saline. Soleus (oxidative, slow twitch muscle), and gastrocnemius (mixed glycolytic-oxidative, fast twitch muscle) were harvested from both the zymosan and control group 2, 7 and 14 days after the insult. Caspase-, trypsin-, and chemotrypsin-like activities of the 26S proteasome were measured by enzymatic assay. Protein expression of activated caspase-3, muscle-specific ubiquitin ligases (MuRF1 and MAFbx), and polyubiquitinated proteins were assessed by Western blots at all time points. Protein expression of caspase-3 specific fragments of myofibrillar actin was assessed at day 7.

**Results** Weight loss was not statistically different for soleus versus gastrocnemius in the zymosan group (-26 ± 11 % versus -16 ± 9 %, p = 0.17) at day 2. Catalytic activity of the 26S proteasome was increased at day 2 in soleus, and days 2 and 7 in gastrocnemius. Soleus displayed upregulation of MuRF1 at days 2 and 14. Gastrocnemius displayed both activation of MuRF1 and MAFbx at day 2 and 7. Activated caspase-3 and polyubiquitinated proteins were increased at days 2, 7 and 14 in soleus but not in gastrocnemius. Caspase-3 specific fragments of myofibrillar actin were increased in soleus, but not in gastrocnemius. Results are summarized in the Table.

**Conclusions** In a rodent model of long-term peritonitis, oxidative and glycolytic muscles show some similarities (e.g. up-regulation of ubiquitin ligases, early proteasome activation) but also marked differences in caspase activation, polyubiquitination and duration of proteasome activation.

**Reference(s)** Hill NE et al. Detailed characterization of a long-term rodent model of critical illness and recovery. Crit Care Med. 2015;43:e84-96.

**Table 51 (abstract A142).** Caspase-3 and ubiquitin-proteasome system

	Soleus			Gastrocnemius		
Day	2	7	14	2	7	14
Activated caspase-3	+	+	+	<->	<->	<->
Actin fragments		+			<->	
MuRF-1	+	<->	+	+	+	<->
MAFbx	<->	<->	<->	+	+	<->
Polyubiquitinated proteins	+	+	+	<->	<->	<->
26S proteasome	+	<->	<->	+	+	<->

### A143 Immunomodulator effects of stem cell application in experimental sepsis model

#### L. Topcu Sarıca^1^, N. Zibandeh^2^, D. Genc^2^, F. Gul^1^, T. Akkoc^3^, E. Kombak^4^, L. Cinel^4^, T. Akkoc^2^, I. Cinel^1^

##### ^1^Marmara University, Anesthesiology and Reanimation, Istanbul, Turkey; ^2^Marmara University, Pediatric Immunology and Alergy, Istanbul, Turkey; ^3^Tubitak Arastırma Merkezi, Gen Engineering, Istanbul, Turkey; ^4^Marmara University, Pathology, Istanbul, Turkey

###### **Correspondence:** L. Topcu Sarıca – Marmara University, Anesthesiology and Reanimation, Istanbul, Turkey

**Introduction** Mesenchymal Stem Cells (MSCs) application is a candidate for preventing organ dysfunctions in sepsis treatment algorithm (1, 2).

**Objectives** In our study, we aimed to investigate the effects of intravenous application of Dental Follicule Derived Mesenchymal Stem Cells (DF-MSCs) on cytokine balance, T regulator levels and ileal tissue histopathology.

**Methods** 38- Sprague Dawley male rats were seperated into five groups. I.Group was control; II. Group was healty + DF-MSC administrated, III. Group was cecal ligation-perforation(CLP), IV. Group was CLP + administration of DF-MSC at zero hour and V. Group was CLP + administration of DF-MSC at fourth hour. At the 24th hour CD4 + CD25 + FoxP3 + (Treg) levels, lymphocyte proliferation and cytokine levels were analyzed from the spleen of sacrificed rats. Histopathologic evaluation of ileal tissue was done by using Chiu score.

**Results** Migration of Green Fluorescent Protein marked MSC(GFP, DF-MSC) to damaged ileocecal epithelium was shown by in Vivo Imaging system(iVIS) studies.

Lympocyte proliferation was greater and CD8 + T lymphocyte levels were lower (18.1 ± 0.5 vs 14.3 ± 0.8) (P < 0.05) in group III, IV and V compared to control one (34.8 ± 7.8 vs 69.1 ± 5.7). Lymphocyte proliferation, cytokine and lymphocyte levels were not different between group III and IV. Treg levels were higher (2.7 ± 0.4 vs 4.4 ± 0.2) and IL-10 levels were lower (1410 ± 120 vs 806 ± 64) (p < 0.05) in group V compared to group III. In addition, TNFalfa levels were tendency to decrease in group V . Ileal damage in group V was lesser than the group III according to Chiu score.

I.group (control): Normal ileal mucosae (Chiu 0)

II.group (CLP + PBS): Naked lamina propria due to denudation of surface epithelium (Chiu 4)

III.group (CLP + 0.h MSC): Disintegration of surface epithelium (Chiu 3)

IV.group (CLP + 4.h MCS): Subepiethelial space formation in apex of villi (Chiu 1)

**Conclusions** There is no known data in the literature about the human derived DF-MSC on treatment of sepsis in experimental models. Our results suggest that intravenous application of DF-MSC results in migration of stem cells to damaged ileoceceal tissue where they can function and protect the integrity of ileal tissue. Our findings showed that immunomodulation is ensured with the supression of proinflamatory(TNF alpha) and anti-inflammatory(IL-10) cytokines together with the increase of Treg ratio and this might be a new therapeutic approach to treat sepsis related organ dysfunctions.

**Reference(s)**

1. Chao, Y.H., et al., An increase in CD3 + CD4 + CD25+ regulatory T cells after administration of umbilical cord-derived mesenchymal stem cells during sepsis. PLoS One, 2014. 9(10): p. e110338.

2. Sung, P.H., et al., *Apoptotic adipose-derived mesenchymal stem cell therapyprotects against lung and kidney injury in sepsis syndrome caused by cecal ligation puncture in rats.* Stem Cell Res Ther, 2013. **4**(6): p. 155.

**Fig. 57 (abstract A143).**
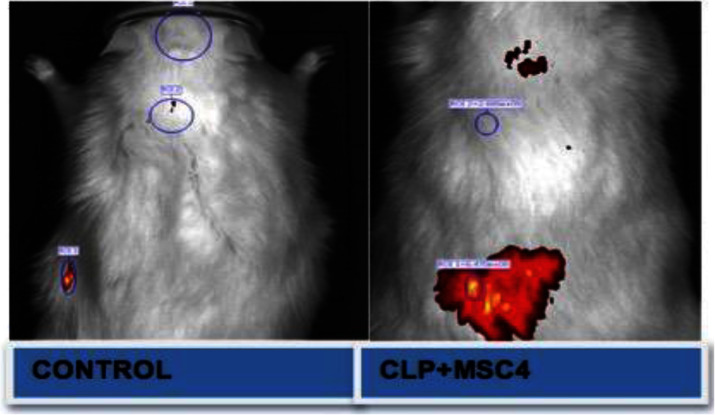
In Vivo Imaging (IVIS) of GFP, DF-MSCs

**Fig. 58 (abstract A143).**
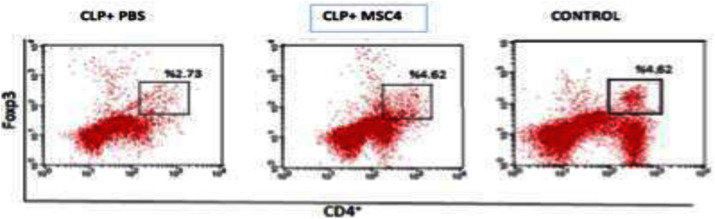
Flowcytometric analysis of Treg cells

**Fig. 59 (abstract A142).**
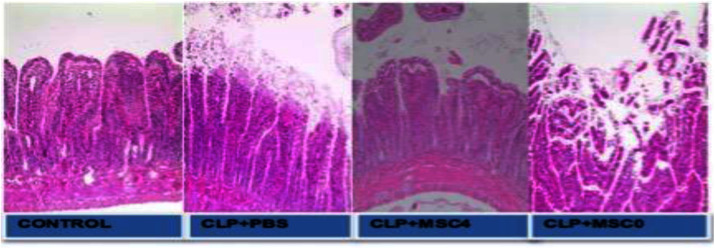
Histopathologi analysis of ilear tissue

### A144 Septic serum alters mitochondrial function in a kidney slice model of septic acute kidney injury

#### S.J. Pollen, N. Arulkumaran, M. Singer

##### UCL, Bloomsbury Institute of Intensive Care Medicine, London, United Kingdom

###### **Correspondence:** S.J. Pollen – UCL, Bloomsbury Institute of Intensive Care Medicine, London, United Kingdom

**Introduction** Increasing evidence is being presented which suggests that modulation of mitochondrial bioenergetics may play a key role in the apparent paradox between the clinical and biochemical presentation of acute kidney injury (AKI) observed in sepsis, and the lack of cell death, maintenance of tissue oxygenation, and eventual recovery.^1^ Furthermore, evidence from in vitro studies have indicated that humoral mediators carried in the circulation may play a role in the mitochondrial dysfunction observed in sepsis that result in organ dysfunction unrelated to haemodynamic changes.^2^

**Objectives** Determine if exposure to septic serum modulates mitochondrial function in a live naïve kidney slice. The mitochondrial functions probed were: mitochondrial membrane potential (MMP), redox state, and reactive oxygen species (ROS) generation.

**Fig. 60 (abstract A144).**
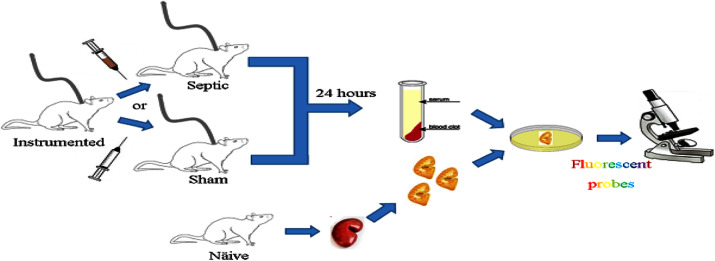
General overview of the relationship between the *in vivo* and *ex vivo* models of sepsis described in the text. Rats are instrumented and following surgical recovery are administered with an intraperitoneal injection of either faecal slurry or normal saline. At 24 hours the animals are culled and blood removed. The blood is processed into serum and used for further experiments using slices of kidney tissue from a naïve rate. Serum and kidney slices incubated together may be examined for dynamic changes in mitochondrial function using fluorescent probes under a two-photon laser microscope

**Methods** Live naïve kidney slices (200 μm thick) were exposed to serum from 24 hour sham operated or septic rats for 90 minutes and imaged with a confocal microscope using fluorescent dyes to detect dynamic changes in mitochondrial function (Fig. 60).

Tetramethylrhodamine methyl ester (TMRM) is an indicator of MMP whose signal decreases with MMP depolarisation. Dihydroethidium (HEt) is an indicator of ROS whose signal increases with increasing ROS. NADH is constitutively fluorescent and can be used as a marker of the redox state.

**Fig. 61 (abstract A144).**
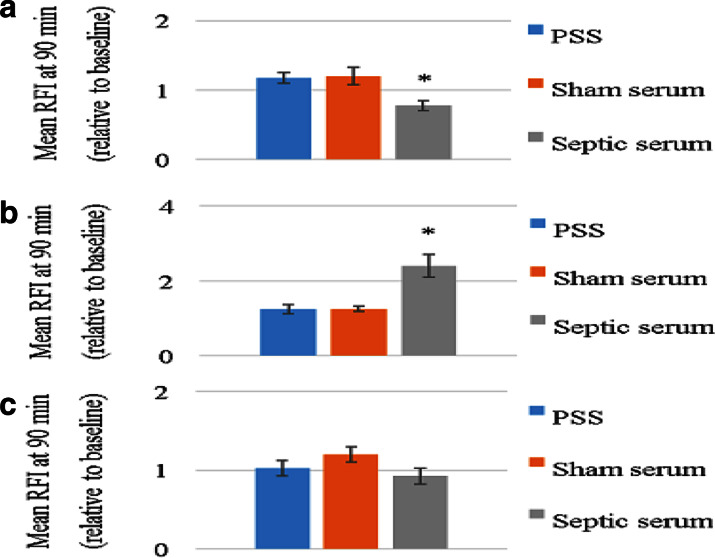
a) Mitochondrial membrane potential. Mean tetremethylrhodamine methyl ester RFI at 90 min (relative to baseline) for kidney slices exposed to PSS, sham serum or septic serum. b) Reactive oxygen species generation. Mean dihydroethidium RFI at 90 min (relative to baseline) for kidney slices exposed to PSS, sham serum or septic serum. c) Redox state. Mean NADH RFI at 90 min (relative to baseline) for kidney slices exposed to PSS, sham serum or septic serum. * = p < 0.05, n = 9, RFI = relative fluorescence intensity, PSS = physiological saline solution

**Results** Septic serum caused a decrease in MMP, an increase in ROS, but no change in NADH at 90 minutes exposure compared to baseline (Fig. 61). Sham serum did not cause any change from baseline and was comparable to slices exposed only to a physiological saline solution.

**Conclusions** The decrease in MMP seen during exposure to septic serum could be indicative of either increased uncoupling or decreased electron transport chain activity. In the first case one would expect a parallel decrease in NADH signal while in the second one would expect an increase in NADH. In this study, NADH did not change in the septic exposed slices and so it is not clear which of the two proposed scenarios is more likely.

Mitochondria are an established source of cellular ROS and in this ex-vivo model, ROS increased significantly following exposure to septic serum. ROS may be a both a mediator and a consequence of mitochondrial dysfunction and the relationship between the two should be explored further.

This study has suggested that humoral factors within septic serum are capable of causing mitochondrial dysfunction in an ex-vivo kidney slice model.

**Reference(s)**

1 Singer, M. The role of mitochondrial dysfunction in sepsis-induced multi-organ failure. Virulence 5, 66-72, doi:10.4161/viru.26907 (2014).

2 Pathak, E., MacMillan-Crow, L. A. & Mayeux, P. R. Role of mitochondrial oxidants in an in vitro model of sepsis-induced renal injury. J Pharmacol Exp Ther 340, 192-201, doi:10.1124/jpet.111.183756 (2012).

**Grant acknowledgement**

UCL Medical School MBPhD Programme and Astor Foundation Scholarship

### A145 Post-traumatic deficits in antigen presentation are mediated via IL-10 dependent pathways and reversible with interferon gamma (IFN-γ) or granulocyte-macrophage colony-stimulating factor (GM-CSF) treatment

#### H.D. Torrance^1,2,3^, E.R. Longbottom^1,3^, G. Warnes^2^, C.J. Hinds^1,3^, D.J. Pennington^2^, K. Brohi^1,2^, M.J. O'Dwyer^1,3^

##### ^1^Barts Health NHS Trust, London, United Kingdom; ^2^The Bizard Institute, Barts & the London School of Medicine & Dentistry, London, United Kingdom; ^3^The William Harvey Research Institute, Barts & the London School of Medicine & Dentistry, London, United Kingdom

###### **Correspondence:** H.D. Torrance – Barts Health NHS Trust, London, United Kingdom

**Introduction** The incidence of ICU-acquired infection following major trauma is greater than 60 % (1). A hyperacute increase in IL-10 (2) as well as a reduction in monocyte HLA-DR (mHLA-DR) expression (3) has been documented following injury and these are associated with the development of late nosocomial infection.

**Objectives** To evaluate the role of IL-10 mediated pathways in post-traumatic immune suppression and to assess the reversibility of this phenomenon.

**Methods** Serum was collected from consecutive patients suffering severe blunt polytrauma at admission and again at 24 hours (24HR). Age and sex matched healthy volunteers served as controls.

Pooled peripheral blood mononuclear cells (PMBCs) from healthy volunteers were incubated for 20 hrs with media containing 30 % serum from either trauma patients or healthy volunteers. These were cultured in the presence or absence of granulocyte-macrophage Colony-stimulating factor (GM-CSF, 200 ng/mL), Interferon Gamma (IFN-γ, 250 International Units) or an IL-10 neutralising antibody (10 ng/mL). Monocyte HLA-DR (mHLA-DR) membrane density (*geometric* mean florescent intensity (*g*MFI)) was characterised on a BD FACS ARIA IIIu flow cytometer. Data are displayed as median and interquartile range (IQR) and analysed with non-parametric statistics.

**Fig. 62:**
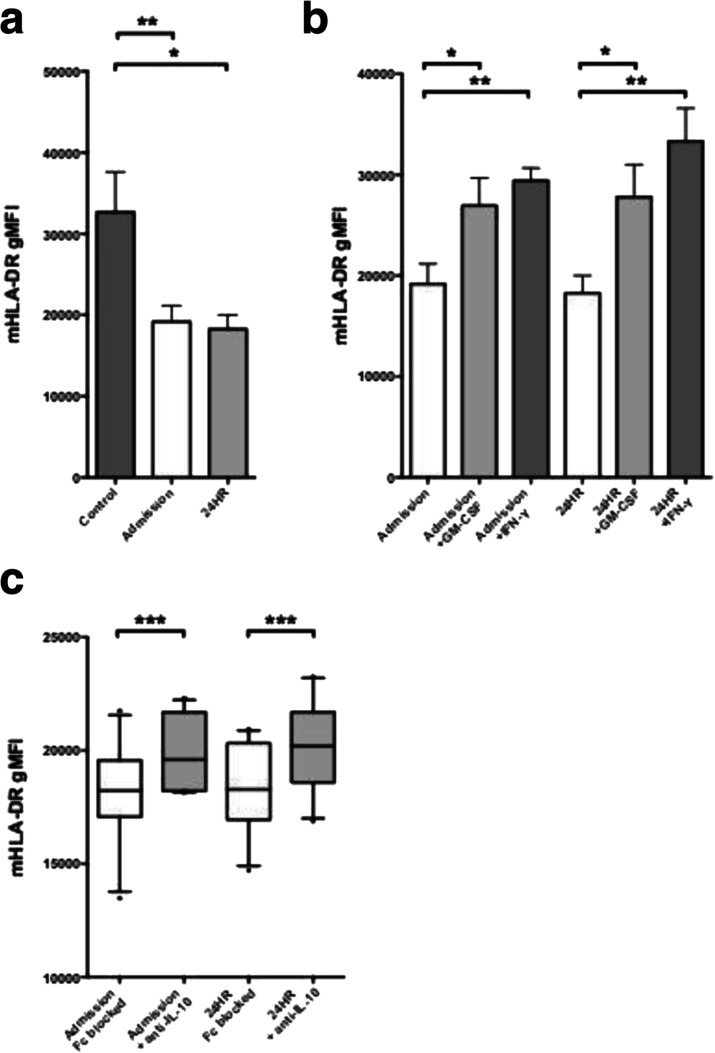
(abstract A145).

**Results** Ten polytrauma patients with a median Injury Severity Score (ISS) of 38 (IQR 29-50) were recruited. This cohort was 80 % male with a median age of 27 (IQR 23-50). There was a decrease in antigen density of mHLA-DR on healthy donor PBMCs when incubated with admission (*P* < 0.01) or 24HR (*P* < 0.05) polytrauma serum, compared to incubation with serum from age and sex matched controls (Fig. 62a). Culturing in the presence of IFN-γ (*P* < 0.01) or GM-CSF (*P* < 0.05) prevented this decrease in antigen density (Fig. 62b). Pre-incubation with an IL-10 neutralising antibody partially reversed the detrimental effects of the incubation with polytrauma trauma serum (*P* < 0.001; Fig. 62c).

**Conclusions** Serum obtained from polytrauma patients induces an immunosuppressive response through an IL-10 dependent pathway, which is reversible with IFN-γ or GM-CSF treatment.

**Reference**

1. Torrance HD *et al. Ann Surg*. 2015; 261:751-759.

2. Torrance HD *et al. Crit Care.* 2013; 17(Suppl. 2),P27.

3. Cheron A *et al*. Crit Care. 2010;14(6):R208.

**Grant acknowledgement**

Royal College of Surgeons of England

Barts & the London Charity

## Perioperative intensive care and delirium

### A146 Volatile sedation via anesthetic conserving device: does this have qualification as a next player of ICU sedation instead of conventional intravenous sedation? A meta-analysis

#### H.Y. Kim, S. Na, J. Kim

##### Yonsei University College of Medicine, Anesthesiology, Seoul, Republic of Korea

###### **Correspondence:** H.Y. Kim – Yonsei University College of Medicine, Anesthesiology, Seoul, Republic of Korea

**Introduction** Prolonged use of intravenous (IV) drugs for intensive care unit (ICU) sedation is associated with accumulation and difficulty of control. After a new volatile anesthetic conserving device (ACD) was introduced, the use of volatile anesthetic agents for sedation in ICU has emerged. Though many trials have shown the effectiveness of sedation using volatile agents in several aspects, these studies have been too small to identify significant effects.

**Objectives** To access the overall efficacy for volatile sedation compared with IV sedation in the ICU patients.

**Methods** We reviewed for all publications from PubMed, Embase, and the Cochrane database on ICU sedation comparing volatile anesthetics (sevoflurane or isoflurane) using ACD with IV agents (propofol or midazolam ± remifentanil). And we performed meta-analysis on eligible studies. Two reviewers independently assessed studies for inclusion and extracted data. The Cochrane Collaboration methodology was used. Standardized mean differences (SMD) with 95 % confidence intervals (CIs) were estimated.

**Results** Sixteen trials and 942 patients were included in our meta-analysis. Extubation time from termination of sedation comparing volatile with intravenous sedatives was significantly shorter in the volatile sedation group than IV sedation group (SMD -1.062, 95 % CIs -1.311 - -0.813; p < 0.001). In addition, the ICU length of stay (LOS) was significantly shorter in the volatile sedation group than IV sedation group (SMD -0.183, 95 % CIs -0.350 - -0.016, p = 0.032).

**Conclusions** We found evidence that ICU sedation with volatile ACD provides the shorter extubation time from termination of sedation and the ICU LOS

**References**

1. Misra S, Koshy T. A review of the practice of sedation with inhalational anaesthetics in the intensive care unit with the AnaConDa((R)) device. Indian J Anaesth. 2012;56(6):518-23.

2. Soukup J, Scharff K, Kubosch K, Pohl C, Bomplitz M, Kompardt J. State of the art: sedation concepts with volatile anesthetics in critically Ill patients. Journal of critical care. 2009;24(4):535-44.

**Grant acknowledgement**

The authors thank Ha Yan Kim (Medical statistician, Yonsei University College of Medicine, Seoul, Korea) for her help with statistical analysis.

**Fig. 63 (abstract A146).**
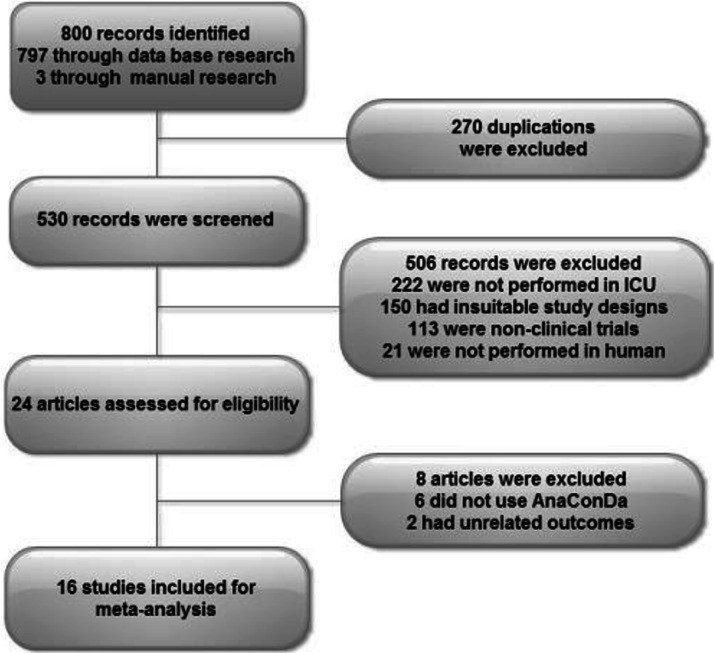
Flow chart for the study selection process

**Fig. 64 (abstract A146).**
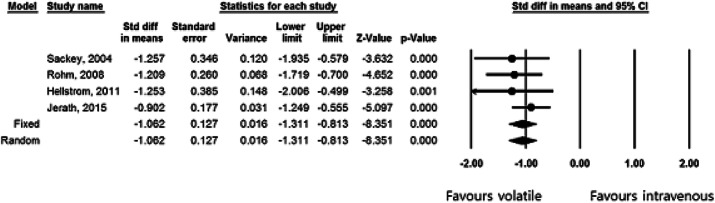
Forest plot of extubation time

**Fig. 65 (abstract A146).**
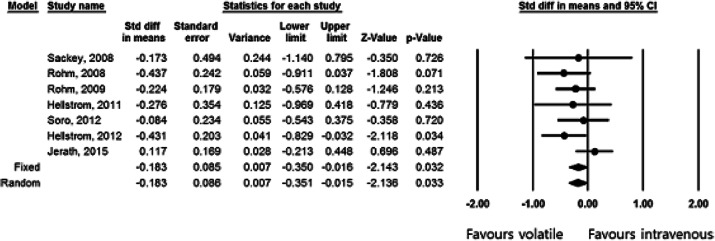
Forest plot of ICU length of stay

### A147 Comparison of hemodynamics between dexmedetomidine and propofol for sedation in patients with abdominal surgery

#### Y.-F. Chang^1,2,3^, A. Chao^4^, P.-Y. Shih^4^, C.T. Lee^4^, Y.C. Yeh^4^, L.-W. Chen^1,5^

##### ^1^National Yang-Ming University, Institute of Emergency and Critical Care Medicine, Taipei, Taiwan, Province of China; ^2^National Taiwan University Hospital, Department of Surgery, Taipei, Taiwan, Province of China; ^3^National Taiwan University Hospital, Department of Nursing, Taipei, Taiwan, Province of China; ^4^National Taiwan University Hospital, Department of Anesthesiology, Taipei, Taiwan, Province of China; ^5^Kaohsiung Veterans General Hospital, Department of Surgery, Kaohsiung, Taiwan, Province of China

###### **Correspondence:** Y.-C. Yeh – National Taiwan University Hospital, Taipei, Taiwan, Province of China

**Introduction** The clinical practice guideline for the management of agitation of the American College of Critical Care Medicine suggests that either propofol or dexmedetomidine may be preferred over sedation with benzodiazepine to improve clinical outcomes in critically ill intubated patients.

1. Recent study have revealed that dexmedetomidine reduces incidence and shortens duration of postoperative delirium.
2. It remains unknown that whether the side effects of bradycardia and hypotension of dexmedetomidine may affect cardiac output or stroke volume in critically ill patients.

**Objectives** The aim of this study is to compare the hemodynamic effects between dexmedetomidine and propofol for postoperative sedation in patients with abdominal surgery.

**Methods** This is a randomized controlled clinical trial (ClinicalTrials.gov ID: NCT02393066). 60 patients undergoing abdominal tumor surgery were enrolled in the study and randomly allocated to Propofol group or Dexmedetomidine group. Cardiac index and stroke volume index were measured by a continuous non-invasive cardiac output monitoring using bioreactance-based technique. Opioid requirement, urine output, length of ICU stay and hospital stay were compared between the two groups.

**Results** The baseline heart rate and mean arterial pressure were not significantly different between the two groups.

During the treatment period, heart rate and mean arterial pressure were significantly lower in the Dexmedetomidine group than in the Propofol group.

No severe bradycardia no hypotension was noted in both groups. The cardiac index and stroke volume index were not significantly different between the two groups.

The mean length of hospital stay was shorter in the Dexmedetomidine group than in the Propofol group (20.3(10.1) vs 20.6(12.6) days, p = 0.037).

**Conclusions** Our results support that the cardiac output and stoke volume were not significantly different between sedation with dexmedetomidine or propofol. We found that postoperative sedation with dexmedetomidine shortens the length of hospital stay in patients with abdominal surgery.

**Reference(s)**

1. Barr J, Fraser GL, Puntillo K, et al. Clinical practice guidelines for the management of pain, agitation, and delirium in adult patients in the intensive care unit. Crit Care Med 2013;41(1):263-306.

2. Djaiani G, Silverton N, Fedorko L, et al. Dexmedetomidine versus Propofol Sedation Reduces Delirium after Cardiac Surgery: A Randomized Controlled Trial. Anesthesiology 2016;124(2):362-368.

**Grant acknowledgement**

Supported, in part, by research grant NTUH.105-A125 from the National Taiwan University Hospital.

**Table 52 (abstract A147).** Patient Characteristics

	Propofol	Dexmedetomidine	P value
Patient number	29	31	
Age (y/O)	70(10)	71(12)	0.64
Gender(male/female)	16 / 13	19 / 12	0.79
Height (cm)	159 (9)	161 (10)	0.55
Weight (kg)	59.8 (8.5)	61.1 (16.9)	0.70
Baseline heart rate (bpm)	75 (9)	76 (10)	0.74
Baseline NIBP MAP(mm Hg)	93 (8)	93 (11)	0.85
APACHE II score	13 (3)	13 (4)	0.46
Infusion dose	0.42 (0.08) mg/kg/h	0.17 (0.06) mcg/kg/h

**Table 53 (abstract A147).** Hemodynamic Parameters and Patient Outcomes

	Propofol (n = 29)	Dexmedetomidine (n = 31)	P value
Heart rate_2h (bpm)	87(13)	77(11)	0.003
Heart rate_6h (bpm)	88(17)	75(12)	0.001
Mean arterial pressure_2h (mm Hg)	94(11)	83(14)	0.001
Mean arterial pressure_6h (mm Hg)	89(13)	76(10)	<0.001
Blood sugar level_6h (mg/dL)	201(60)	183(43)	0.228
Equivalent morphine consumption_24h (mg)	35.1(22.5)	39.8(29.5)	0.488
Urine output_24h (mL)	1769(684)	1629(711)	0.440
Length of ICU stay (h)	47.8(28.0)	60.0(44.1)	0.209
Length of hospital stay (day)	26.6(12.6)	20.3(10.1)	0.037

**Table 54 (abstract A147).** Hemodynamic Parameters and Patient Outcomes

	Propofol (n = 29)	Dexmedetomidine (n = 31)	P value
Heart rate_2h (bpm)	87(13)	77(11)	0.003
Heart rate_6h (bpm)	88(17)	75(12)	0.001
Mean arterial pressure_2h (mm Hg)	94(11)	83(14)	0.001
Mean arterial pressure_6h (mm Hg)	89(13)	76(10)	<0.001
Blood sugar level_6h (mg/dL)	201(60)	183(43)	0.228
Equivalent morphine consumption_24h (mg)	35.1(22.5)	39.8(29.5)	0.488
Urine output_24h (mL)	1769(684)	1629(711)	0.440
Length of ICU stay (h)	47.8(28.0)	60.0(44.1)	0.209
Length of hospital stay (day)	26.6(12.6)	20.3(10.1)	0.037

**Fig. 66 (abstract A147).**
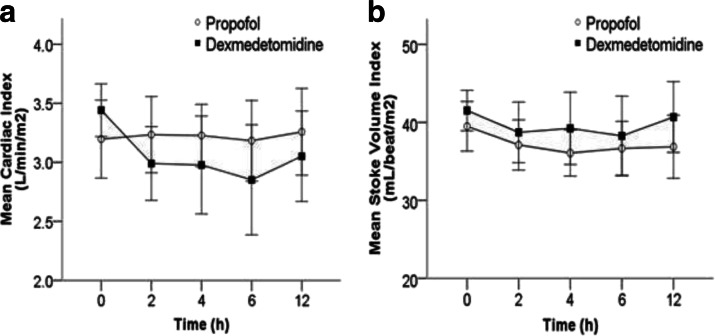
Cardiac index and Stroke Volume Index

### A148 Impact of delirium screening tool on prognostic value of delirium for hospital mortality in critically ill patients: a sub-analysis of a large multicentre prospective implementation study in the Netherlands

#### M. Adriaanse^1^, Z. Trogrlic^2^, E. Ista^3^, H. Lingsma^4^, W. Rietdijk^2,5^, H.H. Ponssen^6^, J.F. Schoonderbeek^7^, F. Schreiner^8^, S.J. Verbrugge^9^, S. Duran^10^, D.A.M.P.J. Gommers^2^, M. van der Jagt^2^

##### ^1^Bravis Hospital, Department of Intensive Care, Roosendaal, Netherlands; ^2^Erasmus Medical Center, Department of Intensive Care, Rotterdam, Netherlands; ^3^Erasmus MC-Sophia Children's Hospital, University Medical Center, Department of Pediatric Surgery, Intensive Care Unit, Rotterdam, Netherlands; ^4^Erasmus Medical Centre, Department of Public Health, Rotterdam, Netherlands; ^5^Zilveren Kruis, Achmea B.V., Marketing Intelligence Department, Leiden, Netherlands; ^6^Albert Schweitzer Hospital, Department of Intensive Care, Dordrecht, Netherlands; ^7^Ikazia Hospital, Department of Intensive Care, Rotterdam, Netherlands; ^8^IJsselland Hospital, Department of Intensive Care, Rotterdam, Netherlands; ^9^Sint Franciscus Gasthuis, Department of Intensive Care, Rotterdam, Netherlands; ^10^Maasstad Hospital, Department of Intensive Care, Rotterdam, Netherlands

###### **Correspondence:** M. Adriaanse – Bravis Hospital, Department of Intensive Care, Roosendaal, Netherlands

**Introduction** Delirium is common in the critically ill and is an important independent predictor of mortality. To be able to accurately manage delirium, screening for its presence with the Confusion Assessment Method for the Intensive Care Unit (CAM-ICU) or the Intensive Care Delirium Screening Checklist (ICDSC) is essential.

**Objective** To assess the impact of delirium screening with a validated screening tool and the tool used (CAM-ICU versus ICDSC) on the prognostic value of delirium for hospital mortality.

**Methods** A prospective multicenter before-after implementation intervention study was conducted in six ICUs between March 2012 and April 2015 in the Netherlands. The intervention consisted of a multifaceted implementation of the Pain, Agitation and Delirium guidelines. For this sub-analysis, all consecutive adult patients who were admitted to ICU, excluding those with primary neurological diagnoses, were included during three 4-month periods. We discerned a before-period (no routine daily delirium screening with CAM-ICU or ICDSC; delirium was considered present when noted in medical or nursing charts or when antipsychotics were given) and after-period (after multifaceted implementation of daily delirium screening; three hospital using CAM-ICU and three ICDSC). The primary outcome was the association of delirium, assessed with or without screening and with ICDSC or CAM-ICU, with hospital mortality. Multivariable logistic regression analysis was used with adjustment for covariables age, APACHE II, admission type (medical, or elective/emergency surgery) and hospital (only for before- vs. after-period analysis). Interactions between covariables were tested. We further assessed the primary outcome in the before- vs. after-period (screening -/+), and in the after-period (screening+) when assessed with CAM-ICU versus ICDSC, by entering these as covariates in the regression model.

**Results** 4033 patients were included (before- and after-period: 1385 and 2632 patients, 15 patients had missing data on covariables). Delirium was independently associated with hospital mortality in crude analysis (OR 1.95, *p* < 0.001, **Table** 55). There was significant interaction between APACHE II and delirium (p < 0.001). After adjustment the OR for delirium was 8.61, but the effect was much stronger in the patients with a higher APACHE II score above the median value (≤15; OR 1.68, *p* = 0.07 versus >15; OR 19.0, *p* < 0.001). Mortality risk of delirium in the after-period compared with the before-period and with CAM-ICU versus ICDSC (after-period) did not differ (before versus after-period, OR 0.82, *p* = 0.079 and ICDSC versus CAM-ICU, OR 1.03, *p* = 0.831).

**Conclusions** In this large multicentre prospective study, we confirmed the independent association of ICU delirium with hospital mortality but found significant interaction with APACHE II. The screening instrument used (CAM-ICU versus ICDSC) did not influence the delirium-associated risk of in-hospital death.

**Table 55 (abstract A148).** Association delirium - hospital mortality

	(1) Hospital mortality	(2) Hospital mortality
Delirium during ICU admission^1^	1.95***	8.61***
	(1.62 - 2.34)	(4.46 - 16.63)
APACHE II score		1.19***
		(1.17 - 1.21)
Delirium during ICU * APACHE II (interaction term)		0.91***
		(0.89 - 0.94)
Number of patients (n)	4033	4017*

1 Any delirium diagnosis in before- and after-period (with or without screening) Coefficients are odds ratios (ORs) and their 95% confidence intervals (95% CI). (1)=crude analysis, (2) =adjusted analyses with the following covariates next to those shown in table: age, hospital (n=6), admission type

*** p<0.001, ** p<0.01, *numbers differ due to small numbers of patients with missing covariable values (not imputed)

### A149 Pain assessment in the unconscious patient- a comparison of three different analgesia-indices with clinical signs in a prospective observational clinical study

#### S. Funcke^1^, S. Sauerlaender^1^, B. Saugel^1^, H. Pinnschmidt^2^, D.A. Reuter^1^, R. Nitzschke^1^

##### ^1^University Medical Center Hamburg-Eppendorf, Center of Anesthesiology and Intensive Care Medicine, Hamburg, Germany; ^2^University Medical Center Hamburg-Eppendorf, Institute of Medical Biometry and Epidemiology, Hamburg, Germany

###### **Correspondence:** S. Funcke – University Medical Center Hamburg-Eppendorf, Center of Anesthesiology and Intensive Care Medicine, Hamburg, Germany

**Introduction** Levels of analgesia under sedation are traditionally evaluated by clinical signs such as an increase in heart rate (HR), blood pressure, lacrimation and defensive movements. However, the patients' analgesic level under sedation is hard to determine in the intensive care setting, as these clinical signs are not specific. Recently, the assessment of the anti-/ nociception balance by different monitoring devices was described. The Analgesia Nociception Index (ANI) (MetroDoloris, Lille, France) is derived from HR variability and provides an index between 0 (nociception) and 100 (analgesia). The Surgical Pleth Index (SPI) (GE Healthcare, Helsinki, Finland) derived from HR and pulse wave amplitude measured with photoplethysmography displays the parasympathetic tone as index between 0 and 100. Pupillary reflex dilatation (PRD) following a noxious event is measured with video recording (AlgiScan, IDMed, Marseille, France).

**Objectives** This prospective observational clinical study was designed to evaluate the diagnostic accuracy of the analgesic indices in assessing the level of analgesia under sedation compared to clinical signs.

**Methods** After obtaining ethics approval and informed consent, 37 surgical patients were anaesthetized pre-operative with propofol to a bispectral index (BIS) 30-60. A laryngeal mask was inserted and remifentanil was increased step-wise to a dose of 0.05, 0.10, 0.15 and 0.2 μg/kg/min. After ensuring a steady-state period, two different standardized painful stimuli were applied. Tetanic stimulation (80 mA, 30 sec., 50Hz), the most common used noxious event in clinical studies, was compared to Bromm's pain model, a direct intracutaneous stimulation (80 mA, 30 sec., 2Hz) of Aδ- and C-fibres [1]. All stimulations were accompanied by recordings of SPI, ANI, PRD, HR, mean arterial pressure (MAP) and BIS. Sensitivity and specificity in detecting the painful stimulus were compared by calculating the area under the curve (AUC) of the Receiver Operating Characteristic curves.

**Fig. 67:**
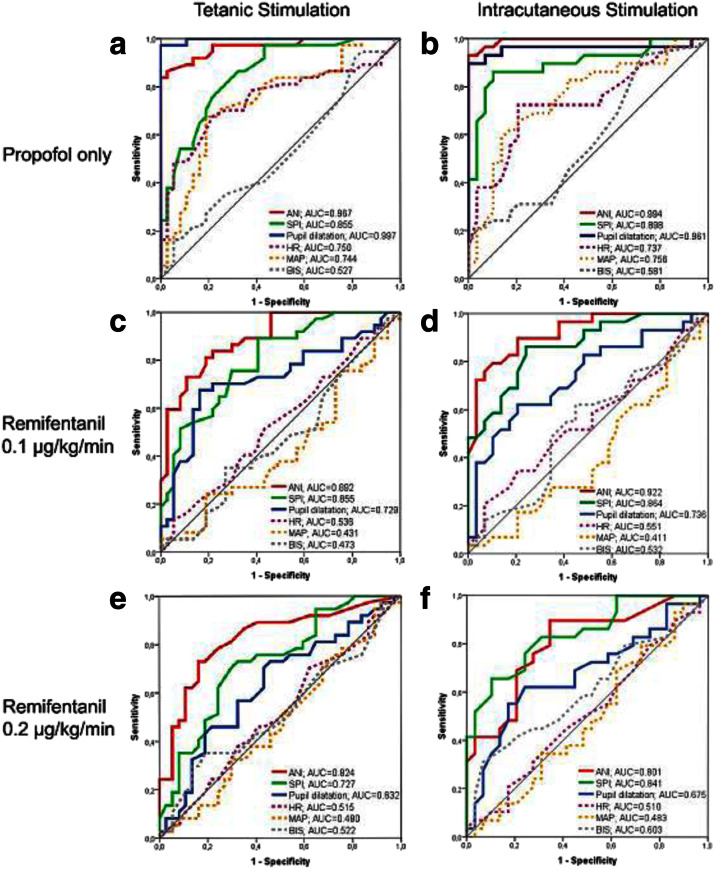
(abstract A149).

**Results** Under propofol sedation, sensitivity and specificity of ANI (AUC = 0.97 and 0.99), SPI (AUC = 0.86 and 0.90) and PRD (AUC = 1.00 and 0.96) for detecting both painful stimuli were high compared to HR, MAP and BIS (AUC = 0.75 and 0.74, 0.74 and 0.76 and 0.53 and 0.58, resp., Fig. 67a + b). Likewise, with propofol sedation and remifentanil 0.2 mcg/kg/min, sensitivity and specificity of ANI (AUC = 0.82 and 0.80.), SPI (AUC = 0.73 and 0.84) and PRD (AUC = 0.63 and 0.68) for detecting both painful stimuli were higher compared to HR, MAP and BIS (AUC = 0.52 and 0.51, 0.48 and 0.48 and 0.52 and 0.60, resp., Fig. 67c-f).

**Conclusions** All three analgesic indices are superior in detecting both painful stimuli under sedation compared to clinical signs. BIS, as a marker of sedation, is confirmed to be no marker of the analgesic level.

**Reference(s)**

[1] Bromm et al. Neurophysiological evaluation of pain. Electroencephalogr Clin Neurophysiol. 1998;107:227-53.

**Grant acknowledgement**

None.

### A150 Sevoflurane pharmacokinetics during a procedural sedation in critically ill burn patients: a comparative study

#### S. Perbet^1^, C. Biboulet^1^, A. Lenoire^2^, D. Bourdeaux^3^, B. Pereira^4^, B. Plaud^2^, J.-E. Bazin^1^, V. Sautou^3^, A. Mebazaa^2^, J.-M. Constantin^1^, M. Legrand^2^

##### ^1^University Hospital Clermont-Ferrand, ICU, Clermont-Ferrand, France; ^2^Saint-Louis Hospital, APHP, Burn ICU, Paris, France; ^3^University Hospital Clermont-Ferrand, Pharmacy, Clermont-Ferrand, France; ^4^University Hospital Clermont-Ferrand, Biostatistics, Clermont-Ferrand, France

###### **Correspondence:** S. Perbet – University Hospital Clermont-Ferrand, ICU, Clermont-Ferrand, France

**Introduction** The use of sevoflurane in ICU is possible with the Anaconda® system. Its properties could be interesting during a procedural sedation for repetitive dressings of burn patients in ICU.

**Objectives** To evaluate the pharmacokinetics of sevoflurane administration during a procedural sedation in a population of burn patients compared to a standard population of critically ill patients

**Methods** Burn patients (body surface area burned (BSAB) with 3rd degree between 20 and 50 %) requiring procedural sedation and control patients were enrolled. Sevoflurane was administered with an expired fraction target of 2 %. Plasma concentrations of sevoflurane, the hexafluoroisopropanolol (HFIP) and free fluoride ions were recorded at different times. The Kinetic Pro (Wgroupe, France) was used for pharmacokinetic analysis.

**Results** Twelve burn patients (BSAB 36 ± 11 %) and 12 controls (mean age of 49 ± 17 years, respectively, vs 55 ± 17 years (p = 0.43) and mean body mass index 26 ± 3 vs 25 ± 2 kg / m2, p = 0.21) were included. The average plasma concentration of sevoflurane was 70.4 ± 37.5 mg / l in burn patients and 57.2 ± 28.1 mg / l in controls at the end of sedation (p = 0.58) with a slower decrease in burn (p = 0.02 after discontinuation of sedation). The volume of distribution was higher (47 against 22 l l) and the half-life was longer (1.2 hours against 0.7 h) in burn patients. The HFIP free rates were higher in burn patients (2.6 mg / L vs 1.6 mg / mL 30 minutes after discontinuation of sedation, p = 0.02) but remained below 6 mg / ml . The average plasma fluoride was not different at day 1 (21.6 ± 12.1 mmol / L vs 38.1 ± 34.6 mmol / L, p = 0.16) and J2 (10.3 ± 7.1 mmol / L vs 15.6 ± 18.8 mmol / L, p = 0.41). The values of urea and plasma creatinine remained stable.

**Conclusions** The study of the pharmacokinetics of the administration of sevoflurane with Anaconda® system in burns highlights an increased volume of distribution and a slower elimination. The times of wash-in and wash-out were very correct with a conserved maneuverability.

### A151 Sleep in ICU: the role of environment

#### Y. Boyko^1^, P. Jennum^2^, M. Nikolic^2^, H. Oerding^3^, R. Holst^4^, P. Toft^1^

##### ^1^Odense University Hospital, University of Southern Denmark, Anaesthesiology and Intensive Therapy, Odense, Denmark; ^2^Danish Center for Sleep Medicine, University of Copenhagen, Copenhagen, Denmark; ^3^University of Southern Denmark, Vejle, Denmark; ^4^University of Southern Denmark, Odense, Denmark

###### **Correspondence:** Y. Boyko – Odense University Hospital, University of Southern Denmark, Anaesthesiology and Intensive Therapy, Odense, Denmark

**Introduction** Sleep and wakefulness are strong interconnected stages regulated by complex mechanism in the basal brain. Disturbed sleep impairs function of nervous and immune systems and metabolism. There has been much focus on sleep in ICU during the recent years: disturbed sleep causes prolonged stay in ICU, increased morbidity, including delirium, and mortality. Several studies reveal pathological sleep patterns in critically ill patients. Critically ill patients in the busy environment of an ICU are exposed to a range of different disturbances such as the high level of noise and light, procedures, mechanical ventilation, medication, and the critical illness itself.Polysomnography (PSG) has never been used in studies on critically ill patients in an ICU to test whether environmental changes improve sleep in this patient group.

**Objectives** We hypothesised that improvement of the intensive care environment would lead to better sleep quality in critically ill mechanically ventilated patients.

**Methods** This study was conducted in a general 8-bed ICU, Vejle Hospital, Denmark. The night-intervention ´Quiet routine' protocol was planned as a bundle of procedures, directed towards improvement of ICU environment between 10 pm and 6 am. Noise levels during control and intervention nights were recorded to control for the intervention. Adult patients with relevant contact on mechanical ventilation were randomized after informed consent to the intervention either during the first or the second night of the study. We monitored sleep by PSG. The standard, American Academy of Sleep Medicine's (AASM's), sleep scoring criteria were insufficient for the assessment of polysomnograms (PSGs). Accordingly, AASM's classification was extended by supplemental criteria for sleep scoring in critically ill patients, suggested by Watson et al. The PSGs were assessed by an expert in sleep medicine blinded to the intervention.

**Results** 19 patients were included in the study. Two patients were excluded due to deterioration.Sound level analysis showed insignificant effect of the intervention on noise reduction (p = 0.3).The analysis of PSGs in accordance with Watson's classification, revealed, only 42 % of the patients exhibited characteristics of normal sleep, while 58 % of the patients had only pathologic patterns.

**Conclusions** Unpredictable factors related to critically ill patients' status hindered implementing the environmental intervention and resulted in insignificant noise reduction. We only found 42 % of the patients having characteristics of normal sleep. The presence of normal sleep characteristics was not associated with the environmental intervention.

**Reference(s)**

Watson et al. Atypical sleep in ventilated patients: empirical electroencephalography findings and the path toward revised ICU sleep scoring criteria. Critical care medicine. 2013;41(8):1958-67.

**Grant acknowledgement**

Region Southern Denmark

**Fig. 68 (abstract A151).**
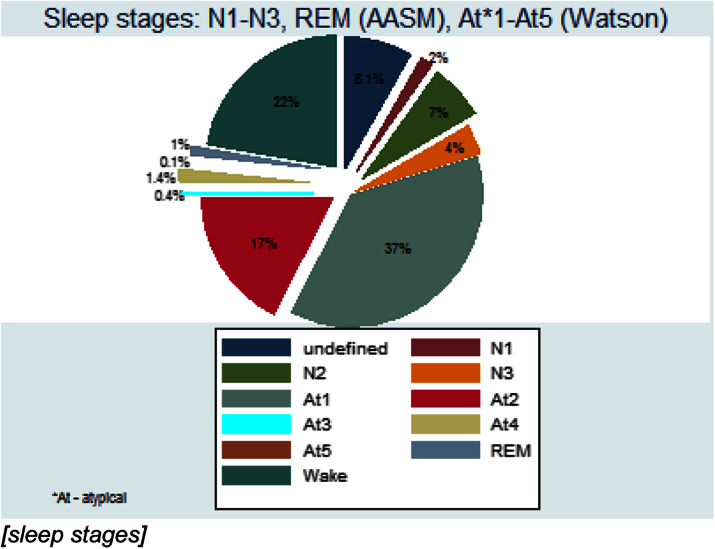
Sleep stages

### A152 Pressure ulcers - preventable by non-sedation? A substudy of the nonseda-trial

#### H.K. Nedergaard^1,2^, T. Haberlandt^2^, H.I. Jensen^1,2^, P. Toft^1,3^

##### ^1^University of Southern Denmark, Odense, Denmark; ^2^Lillebaelt Hospital, Kolding, Denmark; ^3^Odense University Hospital, Odense, Denmark

###### **Correspondence:** H.K. Nedergaard – University of Southern Denmark, Odense, Denmark

**Introduction** Pressure ulcers increase the patients risk of serious infection, and are associated with a higher mortality [1,2]. Critically ill patients are often sedated, leading to sustained periods of immobility. Clinical experience suggests that non-sedated patients are easier to mobilize and change position in bed more often. We therefore hypothesize that non-sedation might prevent pressure ulcers.

Objective To assess whether non-sedation is associated with fewer pressure ulcers.

**Methods** Retrospective data on patients included in the multicenter NONSEDA trial, at the Kolding trial site (DK) per March 15, 2016 (145 patients). Participants were randomised to either standard care of sedation with a daily wake up or to non-sedation during mechanical ventilation.

It is not possible to blind whether a patient is sedated or not. However, the ICU-nurses who performed the clinical assessments and registrations were unaware that we were investigating the incidence of pressure ulcers. All clinical data were extracted and interpreted by the principal investigator before the patient's randomization status was revealed.

If we encountered difficulties ascertaining whether an ulcer was acquired in the ICU or already present at admission, we assumed that the ulcer was ICU-acquired to avoid risk of underestimation.

Primary outcome: total number of pressure ulcers acquired in the ICU, described by grade (I-IV) and localization.

**Results** 65 ICU-acquired or assumed ICU-acquired pressure ulcers were identified. There were no significant differences between groups regarding sex, age, BMI, APACHE II or SAPS II. There were 34 grade 1 pressure ulcers (sedated: 18, non-sedated: 16), 29 grade 2 ulcers (sedated: 17, non-sedated: 12) and 2 grade 3 ulcers (sedated: 1, non-sedated: 1), with no significant difference between groups. Concerning localization, results were grouped into three: sacrum, heels and caused by equipment (for example at nostril from oxygen catheter or at wrist from arterial line). The localization of the ulcers were significantly different (p = 0.04): sacrum (sedated: 36 %, non-sedated: 21 %), heels (sedated: 33 %, non-sedated: 17 %), from equipment (sedated: 31 %, non-sedated: 62 %).

**Conclusions** There were no significant difference in the incidence of pressure ulcers in the two groups. An interesting difference in the localization of the pressure ulcers was found. The sedated patients mainly had ulcers in the classical localizations, namely sacrum and heels, whereas the non-sedated mainly had ulcers related to equipment, in diverse localizations such as the face, limbs and genitalia. Considering the long-term prognosis, ulcers deriving from equipment are easier to relieve and must be expected to heel faster.

**Reference(s)**

1. Cooper, L. et al. The prevention and management of pressure ulcers: summary of updated NICE guidance. J. Wound Care 2015.

2. Cox, J. Predictors of pressure ulcers in adult critical care patientes. Am J Crit Care 2011.

### A153 The impact of sleep on delirium development in critically ill patients

#### S. Park^1^, S. Kim^2^, Y.-J. Cho^3^

##### ^1^Sheikh Khalifa Specialty Hospital, Anesthesiology, Rak, United Arab Emirates; ^2^Sheikh Khalifa Specialty Hospital, Department of Pulmonology, Rak, United Arab Emirates; ^3^Seoul National University Bundang Hospital, Seongnam, Republic of Korea

###### **Correspondence:** S. Park – Sheikh Khalifa Specialty Hospital, Anesthesiology, Rak, United Arab Emirates

**Introduction** Delirium is an acute confusional state that is common in the intensive care unit (ICU). Many critically ill patients treated in the ICU experience sleep disruption. Disrupted sleep in the ICU has been proposed as a potential risk factor for delirium, but the evidence is sparse.

**Objectives** This study was undertaken to identify the sleep status for the development of delirium in non-sedated critically ill patients.

**Methods** This prospective study was conducted in medical ICU of a tertiary referral hospital. Polysomnography recording was performed over 24 hour to assess the quantity and quality of sleep. Delirium was measured daily using the Confusion Assessment Method for the ICU.

**Results** Total 20 patients were enrolled. Their median APAPCHE II score was 19 (IQR : 16 - 27). Median total sleep time was 03:43 (hh:mm, IQR: 00:49 - 06:10). The majority of sleep was stage 1 (median 03:02 [00:47 - 04:34]) with scant stage 2 (median 00:00 [00:00 - 00:46]), REM (median 00:00 [00:00 - 00:15]) and absent stage 3. Delirium was developed in 4 patients (20 %). In multivariable analysis, the duration of ICU stay more than 5 days was independently associated with delirium incidence (P = 0.042). We also found that patients who stayed more than 5 days in ICU showed significant reduction in night sleep time compared to patients who stayed less than 5 days (00:42 ± 0:46 vs 2:04 ± 1:25, P = 0.012), despite of similar total sleep time.

**Conclusions** The quantity and quality of sleep in critically ill patients were poor. The long duration of ICU stay disrupted circadian rhythm which might contribute to the development of delirium in critically ill patients.

### A154 Evaluation of a sedation protocol in mechanically ventilated patients in a medical intensive care unit: a pilot study

#### Y.J. Lim^1^, A. Chan^1^, S. Tang^2^

##### ^1^National University of Singapore, Department of Pharmacy, Singapore, Singapore; ^2^National University Hospital, Department of Pharmacy, Singapore, Singapore

###### **Correspondence:** Y.J. Lim – National University of Singapore, Department of Pharmacy, Singapore, Singapore

**Introduction** Mechanically ventilated patients frequently receive analgesia and sedation to manage pain and agitation in the intensive care unit (ICU). Current clinical practice guidelines advocate the maintenance of light target levels of sedation along with the use of structured sedation protocols and daily sedation interruption. A sedation protocol was therefore developed and implemented in a medical intensive care unit (MICU) at a tertiary hospital to standardize the management of analgesia and sedation in mechanically ventilated patients.

**Objectives** To evaluate the impact of a sedation protocol on patient outcomes in mechanically ventilated patients.

**Methods** This observational before-after study was conducted in the MICU at the National University Hospital (NUH), Singapore between September 2014 and March 2015. The sedation protocol incorporated daily sedation interruption and routine objective assessments of pain, agitation and delirium by nurses and advocated titration of analgesia and sedation to maintain a target Richmond Agitation Sedation Scale (RASS) range of -2 to 0.

Data were collected retrospectively from electronic patient records. The primary outcome was the duration of mechanical ventilation, and the main secondary outcomes included the number of ventilator-free days at day 28, ICU length of stay, ICU mortality and 28-day mortality.

**Results** The study included 53 and 41 patients before and after protocol implementation respectively. There was significant improvement in the percentage of ICU days with daily sedation interruption in the post-implementation period (23.6 % vs 35.9 %, p = 0.0087). There was a trend for decreased duration of mechanical ventilation after implementation (4.45 ± 4.2 days vs 2.55 ± 2.7 days respectively; adjusted effect estimate 0.70, [95 % CI 0.47-1.43], p = 0.067) and a trend for increased ventilator-free days at day 28 post implementation (18.35 ± 11.1 days vs 24.01 ± 7.1 days, adjusted effect estimate 1.64, [95 % CI 0.99-2.70], p = 0.053). The mean ICU length of stay was similar in both groups (6.07 ± 6.3 days vs 4.84 ± 4.4 days, adjusted effect estimate 0.90, [95 % CI 0.64-1.26], p = 0.527), and the ICU and 28-day mortality did not demonstrate any significant differences between groups (13.2 % vs 4.9 %, p = 0.290 and 9 % vs 7.3 %, p =0.219 respectively). The rate of protocol adherence was low, with 68.3 % of patients (n = 28) in the protocol group who had deviations from the sedation protocol.

**Conclusions** For mechanically ventilated patients, the use of a standardized sedation protocol may reduce the duration of mechanical ventilation. Barriers to protocol use should be identified to improve protocol adherence.

**Reference(s)**

Barr, J., et al., *Clinical practice guidelines for the management of pain, agitation, and delirium in adult patients in the intensive care unit.* Crit Care Med, 2013. **41**(1): p. 263-306.

**Grant acknowledgement**

This study did not receive any grants.

### A155 Effect of dexmedetomidine on weaning from mechanical ventilation in intensive care patients

#### S.L. Nunes^1^, S. Forsberg^2,3^, H. Blomqvist^4^, L. Berggren^5^, M. Sörberg^6^, T. Sarapohja^7^, C.-J. Wickerts^1^

##### ^1^Danderyd Hospital, Department of Anesthesiology and Intensive Care, Stockholm, Sweden; ^2^Norrtälje Hospital, Department of Anesthesiology and Intensive Care, Norrtälje, Sweden; ^3^Karolinska Institute, Södersjukhuset, Department of Clinical Science and Education, Stockholm, Sweden; ^4^Karolinska University Hospital, Department of Anesthesiology and Intensive Care, Stockholm, Sweden; ^5^Örebro University Hospital, Department of Anesthesiology and Intensive Care, Örebro, Sweden; ^6^Orion Pharma, Stockholm, Sweden, ^7^Orion Pharma, Espoo, Finland

###### **Correspondence:** S.L. Nunes – Danderyd Hospital, Department of Anesthesiology and Intensive Care, Stockholm, Sweden

**Introduction** Mechanically ventilated intensive care patients are traditionally sedated to assure analgesia, anxiolysis and comfort. This might have negative effects such as prolonged mechanical ventilation (MV) and longer length of stay (LOS). Dexmedetomidine has been shown to shorten time to extubation, but its role in the weaning process is not fully elucidated.

**Objectives** To determine whether sedation regimes affect the weaning process.

**Methods** This was a non-interventional, multicenter, retrospective study. Lightly to moderately sedated intubated adult patients mechanically ventilated for ≥ 24 h were included. SAPS III scores were measured at admission. After admission to the intensive care unit (ICU) and until fit for weaning all patients were sedated with standard of care according to their respective clinics protocol. During weaning period patients sedated with only dexmedetomidine (DEX) were compared to those sedated with midazolam and/or propofol- standard of care (SOC) or SOC + dexmedetomidine (SOCDEX) concerning weaning time. Weaning time, was defined as time from “fit for weaning” to extubation. Total time on MV and LOS in the ICU were measured. Amount of sedatives and analgesics used as well as anxiety and ICU delirium were recorded using NuDESC/CAM-ICU. Measurements of 15D and PTSS were obtained for evaluation of quality of life and incidence of Post-Traumatic Stress Disorder (PTSD) at 2-4 months after ICU discharge.

**Results** A total of 152 patient records were reviewed and included in the study: DEX (31), SOC (67) and SOCDEX (54). The DEX group could be more rapidly weaned as compared to the SOC (p = 0.040) group and SOCDEX (p < 0.001) group, despite longer time in MV prior to weaning 77.2/37.0/50.0 hours of DEX/SOC/SOCDEX respectively. The ICU LOS was shortest for patients in the SOC group 4.9 days (d) compared to SOCDEX: 6.9 d and DEX: 7.0 d. The SAPS III scores were similar in all three groups 61.4/60.6/59.8 of DEX/SOC/SOCDEX patients respectively. Anxiety during weaning and after extubation was present in 0/5/12 and 9/10/15 of DEX/SOC/SOCDEX patients respectively. Delirium was present in 1/1/2 (50 assessed) and 0/1/3 (65 assessed) of DEX/SOC/SOCDEX patients during weaning and at ICU discharge respectively. Quality of life at follow-up as measured by 15D showed better scores in the DEX group as compared to SOCDEX (p = 0.040 ) but not better than SOC (p = 0.175 ). At this point SOCDEX patients showed a tendency for more symptoms related to severe pain, breathing troubles, nightmares and severe anxiety as compared to DEX and SOC. Very few patients fulfilled the PTSD criteria with no differences among groups.

**Conclusions** Dexmedetomidine, when used as single sedation, contributes to a shorter weaning period. These patients tend to describe better quality of life at 2-4 months after ICU discharge.

**Grant acknowledgement**

Orion Pharma sponsored study

### A156 Describing sleep and sedation practices in the ICU: a multinational survey

#### J.G.M. Hofhuis^1^, L. Rose^2^, B. Blackwood^3^, E. Akerman^4^, J. Mcgaughey^5^, I. Egerod^6^, M. Fossum^7^, H. Foss^8^, E. Georgiou^9^, H.J. Graff^6^, M. Kalafati^10^, R. Sperlinga^11^, A. Schafer^12^, A.G. Wojnicka^13^, P.E. Spronk^1^

##### ^1^Gelre Hospitals Apeldoorn, Intensive Care, Apeldoorn, Netherlands; ^2^University of Toronto, Toronto, Canada; ^3^University Belfast, Belfast, United Kingdom; ^4^Clinc of Intensive Care, Malmo, Sweden; ^5^Queens University Belfast, Belfast, United Kingdom; ^6^University of Copenhagen, Copenhagen, Denmark; ^7^University of Agdar, Grimstad, Norway; ^8^Univeristy of Agdar, Grimstad, Norway; ^9^Division of Intensive Care, Cyprus, Cyprus; ^10^University of Athens, Athens, Greece; ^11^University Cattolica del Sacro Cuore, Turino, Italy; ^12^Division of Intensive Care, Germany; ^13^University Warminsko-Mazwski, Olsztyn, Poland

###### **Correspondence:** J.G.M. Hofhuis – Gelre Hospitals Apeldoorn, Intensive Care, Apeldoorn, Netherlands

**Introduction** Sleep disturbances are common in critically ill patients treated in the intensive care unit (ICU) with the potential for serious consequences and long-term effects on health outcomes and patient morbidity.

**Objectives** Our aim was to describe sleep management and sedation practices of adult ICUs in ten countries and to evaluate roles and responsibilities of the ICU staff in relation to key sleep and sedation decisions.

**Methods** A multicenter, self-administered survey sent to nurse managers of adult ICUs across 10 countries. The questionnaire comprised four domains: sleep characteristics of the critically ill; sleep and sedation practices; non-pharmacological and pharmacological interventions used to improve sleep; and the autonomy and influence of nurses on sleeping practices in the ICU.

**Results** Overall response rate was 66 % (range 32 % UK to 100 % Cyprus), providing data from 522 ICUs. In all countries, the most frequent patient characteristic perceived to identify sleep was lying quietly with closed eyes (N = 409, 78 %) (range 92 % Denmark to 36 % Italy). The most commonly used sedation scale was the Richmond Agitation-Sedation Score (RASS) (N = 220, 42 %) (range 81 % UK to 0 % Denmark, Cyprus where most ICUs used the Ramsay score). In most ICUs, selection of sleep medication (N = 265, 51 %) and assessment of effect (N = 309, 59 %) was performed by physicians and nurses based on collaborative discussion. In a minority of ICUs (N = 161, 31 %), decisions and assessments were made by physicians alone. The most commonly used (in all countries) non-pharmacological intervention to promote sleep was reducing ICU staff noise (N = 473, 91 %) (range 100 % Denmark, Norway to 78 % Canada). Only 95 ICUs (18 %) used earplugs on a frequent basis (range 0 % Greece, Cyprus, Denmark to 57 % Sweden). Propofol was the drug used most commonly for sedation (N = 359, 69 %) (range 96 % Sweden to 29 % Canada). Chloral hydrate was used by only 63 (12 %) ICUs (range 0 % Greece, Cyprus, Denmark, Italy to 56 % Germany). Sedation scales were used on a routine basis by 77 % of the 522 ICUs. Participants scored nursing autonomy for sleep and sedation management as moderate; median score of 5 (scale of 0 to 10), range 7 (Canada, Greece, Sweden) to 4 (Norway, Poland). Nursing influence on sleep and sedation decisions was perceived considerable; median score 8, range 9 (Denmark) to 5 (Poland).

**Conclusions** We found considerable across country variation in sleep promotion and sedation management practices though most have adopted a sedation scale as recommended in professional society guidelines. Most ICUs in all countries used a range of pharmacological and non-pharmacological interventions to promote sleep. Most units reported inter-professional decision-making with nurses perceived to have substantial influence on sleep/sedation decisions.

### A157 Implementation of a protocol to control pain, agitation and delirium in the patients admitted in the intensive care unit with opioid drug dependency; a feasibility study

#### F. Zand^1^, F. Khalili^1^, R. Afshari^2^, G. Sabetian^3^, M. Masjedi^1^, B. Maghsudi^1^, H. Haddad Khodaei^4^, S. Javadpour^5^, P. Petramfar^6^, S. Nasimi^7^, A. Vazin^8^, B. Ziaian^9^, H. Tabei^1^

##### ^1^Anesthesiology and Critical Care Research Center, Shiraz University of Medical Sciences, Shiraz, Islamic Republic of Iran; ^2^Department of Drug Abuse Control, Shiraz University of Medical Sciences, Shiraz, Islamic Republic of Iran; ^3^Trauma Research Center, Shiraz University of Medical Sciences, Shiraz, Islamic Republic of Iran; ^4^Shiraz University Of Medical Sciences, Department of Neurosurgery, Shiraz, Islamic Republic of Iran; ^5^Jahrom University of Medical Sciences, Department of Critical Care Nursing, Jahrom, Islamic Republic of Iran; ^6^Shiraz University Of Medical Sciences, Department of Neurology, Shiraz, Islamic Republic of Iran; ^7^Nemazee Hospital, Shiraz University of Medical Sciences, Shiraz, Islamic Republic of Iran; ^8^Shiraz University Of Medical Sciences, Department of Clinical Pharmacy, Shiraz, Islamic Republic of Iran; ^9^Shiraz University of Medical Sciences, Department of Surgery, Shiraz, Islamic Republic of Iran

###### **Correspondence:** B. Ziaian – Shiraz University of Medical Sciences, Department of Surgery, Shiraz, Islamic Republic of Iran

**Introduction** Opioid drug dependency is not uncommon worldwide and withdrawal syndrome is a major clinical concern when these patients are admitted in intensive care unit (ICU) with low levels of consciousness.

**Objectives** We hypothesized if a software could be designed to estimate daily need of these patients to opioids and if a protocol could be designed and implemented to concomitantly control pain, agitation and delirium (PAD) and prevent withdrawal signs in this population during ICU admission.

**Methods** A multidisciplinary team designed the software and protocol. Methadone was used to prevent withdrawal syndrome and pain was assessed hourly, by Behavioral Pain Scale and controlled by morphine or fentanyl. Level of sedation was also assessed hourly, by Richmond Agitation-Sedation Scale and controlled by midazolam or propofol, according to the protocol. Delirium was checked by Confusion Assessment Method for ICU, once in every working shift.

**Results** Thirty patients with history of opium dependency were recruited during an 8-month period in 2 mixed medical-surgical ICU's. The protocol was effective to completely prevent the withdrawal syndrome in 24 patients (80 %). The average need to methadone was 14.5 ± 22.2 mg in the patients.The pain, sedation and delirium were evaluated and documented by the staff in 97, 98 % and 56 % of situations, respectively. Pain and sedation scores were within acceptable limits in 93 and 98 % of occasions, respectively. Delirium occurred in 2 patients during the ICU stay.

**Conclusions** Implementation of a PAD protocol and using a software, especially designed for the opium dependent patients is feasible. Management of PAD could be effectively done with a multidisciplinary approach, along with prevention of withdrawal syndrome.

### A158 Measuring pain - a validation of physiological and self-rated measurements, and an investigation of the relationship between them

#### A. Gunther^1^, J.O. Hansen^2^, P. Sackey^1^, H. Storm^2^, J. Bernhardsson^3^, Ø. Sundin^3^, A. Bjärtå^3^

##### ^1^Karolinska University Hospital, Stockholm, Sweden; ^2^University of Oslo, Oslo, Norway, ^3^Mitt Universitetet, Östersund, Sweden

###### **Correspondence:** H. Storm – University of Oslo, Oslo, Norway, ^3^Mitt Universitetet, Östersund, Sweden

**Introduction** The numeric rating scale (NRS) and skin conductance responses per second (NSCR) are both used as methods to assess pain in the perioperative setting (1). An experimental study was conducted to investigate the relationship between NRS and NSCR, and how these measures are related to anxiety and degree of pain stimulation.

**Methods** Eighteen volunteers were exposed to conditions simulating ICU circumstances using pictorial emotional stimuli (neutral, positive, negative), authentic ICU sound (noise, no noise) and electrocutaneous stimulation (pain, no pain). The electrical stimulation was individually titrated prior to the experiment to induce moderate pain (40 mm > VAS < 60 mm) and NSCR was measured throughout the experiment. All possible combinations of conditions resulted in twelve 60 second sessions, each followed up by NRS for pain, and ratings of arousal and valence as indicators of experienced anxiety.

**Results** Both NRS and NSCR increased in the pain conditions (NRS: *M* = 3.95, [*SD* = 1.78]; NSCR: 0.22 [0.09]), compared to no pain (NRS:0.00 [0.00]; NSCR: 0.09 [0.08]), *t*(18) = 9.45, *p* < .001 and *t*(18) = 8.72, *p* < .001, respectively. There was no change over time in NRS nor NSCR. Significant positive correlations were found between NSCR and the magnitude of the electrical stimulation, *r*(18) = .48, *p* = .046, and also between NRS and the anxiety index (mean of arousal and valence ratings), *r*(18) = .60, *p* = .009. In the pain condition, 3 (emotion) x 2 (noise) ANOVAs of NSCR, NRS, and anxiety showed that both NRS and anxiety were sensitive to the experimental manipulations, shown by main effects of both emotion, *F*(2,34) = 7.54, *p* = .002 and *F*(2,34) = 16.89, *p* < .001 (for NRS and anxiety respectively), and main effects of noise, *F*(1,17) = 8.67, *p* = .009 and *F*(1,17) = 9.78, *p* = .006 (for NRS and anxiety respectively), with elevated ratings of both pain and anxiety in negative conditions compared to ratings in both positive and neutral conditions. NSCR was not influenced by emotion or noise in the pain condition. However, Helmert's contrasts of emotion state only, showed significantly larger NSCR in emotional conditions compared to the neutral, *F*(1,17) = 5.21, *p* = .036.

**Conclusions** Both NRS and NSCR are reliable indicators of pain, and the correspondence between NSCR and actual pain stimulation moreover validates the use of NSCR as a measure of pain in patients. However, NRS is also sensitive to the contextual setting and anxiety, which NSCR is not. A discussion, whether to administer analgesic or ataractic drugs during the perioperative stage when NRS is moderate or higher, is warranted.

**Reference(s)**

1. Günther A, Schandl A, Bernhardsson J, Bjärtå A, Wållgren M, Sundin Ö, Alvarsson J, Bottai M, Martling CR, Sackey P. Pain rather than induced emotions and ICU sound increases skin conductance variability in healthy volunteers. Submitted.

**Grant acknowledgement**

None

### A159 The pharmacokinetics of propofol in ICU patients undergoing long-term sedation

#### A. Bienert^1^, P. Smuszkiewicz^1^, P. Wiczling^2^, K. Przybylowski^1^, A. Borsuk^2^, I. Trojanowska^1^, J. Matysiak^1^, Z. Kokot^1^, M. Paterska^1^, E. Grzeskowiak^1^

##### ^1^Poznan Unversity of Medical Sciences, Poznan, Poland; ^2^Medical University of Gdansk, Gdansk, Poland

###### **Correspondence:** A. Bienert – Poznan Unversity of Medical Sciences, Poznan, Poland

**Introduction** According to the recent guideline (2013), non-benzodiazepine drugs, like propofol, are preferred to benzodiazepines in sedation of ICU patients undergoing mechanical ventilation due to decreased duration of *mechanical ventilation*, shortened ICU *stay*, lower risk of patients death and decreased costs of treatment. Propofol is a relatively well known drug, nevertheless the influence of various factors connected with patients, like demographics, health status or co-administered drugs on the propofol pharmacokinetics has not been fully understood.

**Objectives** The aim of our study was to examine the pharmacokinetics of propofol in a heterogeneous group of patients sedated in an ICU. The specific objective was to investigate the influence of different variables monitored and patients' health status descriptors, like SOFA or presence of sepsis, on the PK of propofol.

**Methods** The propofol concentration-time profiles were obtained from 29 patients. All the subjects were evaluated according to APACHE II score and SOFA score, whereas the level of sedation was applied according to modified Ramsay Sedation score to achieve a sedation score of 3-4. Non-linear mixed-effects modelling in NONMEM (Version 7.3.0, Icon Development Solutions, Ellicott City, MD, USA) was used to analyse the observed data. Blood samples for propofol assay were collected from the patients' arteries on every day of the infusion, at the selected time points after its termination. The propofol concentration in the plasma was measured within eight weeks by HPLC method with a fluorescence detector. Non-parametric bootstrap and visual predictive check were conducted to evaluate the adequacy of the induced model to describe the observations.

**Results** Propofol pharmacokinetics was best described with a three-compartment disposition model. A typical value of propofol clearance (1.46 L/min) approximated liver blood flow. The volume of distribution at steady state was high 955.1 L, but consistent with other studies on ICU patients. We were unable to identify any statistically significant covariate relationships between PK parameters and opioid type, SOFA score at admission, APACHE II, predicted death rate, reason for admission to the ICU (sepsis, trauma or surgery), gender, body weight, age, infusion duration and C-reactive protein.

**Conclusions** The population PK model was successfully developed to describe the time course of propofol concentration in ICU patients undergoing prolonged sedation. Despite a very heterogeneous group of patients, consistent PK profiles were observed.

**Reference(s)**

Eleveld DJ, Proost JH, Cortínez LI, Absalom AR, Struys MM. General purpose pharmacokinetic model for propofol. Anesth Analg 2014; 118: 1221-37.

Gradwohl-Matis I, Mehta S, Dünser MW. What´s new in sedationstrategies? Intensive Care Med. 2015; 41: 1696-9.

**Grant acknowledgement**

This project was supported by the grant 2015/17/B/NZ7/03032 founded by Polish National Science Centre.

## Assessment of preload and fluid responsiveness

### A160 Early variations of pulse pressure variation, cardiac cycle efficiency and dicrotic pressure to predict fluid challenge success

#### A. Messina^1^, E. Bonicolini^2^, D. Colombo^1^, G. Moro^1^, S. Romagnoli^2^, A.R. De Gaudio^2^, F. Della Corte^3^, S.M. Romano^2^

##### ^1^AOU Maggiore della Carità, Novara, Italy; ^2^Azienda Ospedaliero-Universitaria Careggi, Firenze, Italy; ^3^Azienda Ospedaliero-Universitaria Careggi, Novara, Italy

###### **Correspondence:** A. Messina – AOU Maggiore della Carità, Novara, Italy

**Introduction** Fluid challenge (FC) is commonly used to increase cardiac index and oxygen delivery. A Tidal volume (Vt) > 8 ml/kg, which is required to guarantee the efficacy of dynamic index of fluid responsiveness in predicting fluid responsiveness, is nowadays not recommended in operating room (1).

The identification of early variation of hemodynamic variables may act as a clinical target or a safety limit to stop infusion. This approach has been successfully used to assess fluid responsiveness in critically ill patients ventilated with low tidal volume (2).

We evaluated the early variations after a FC in operating room of cardiac cycle efficacy (CCE) and dicrotic pressure (P_dic_) to improve baseline pulse pressure variation (PPV) reliability and predict fluid responsiveness.

**Methods** 40 consecutive adult patients scheduled for elective abdominal surgery and ventilated with Vt

<8 ml/kg. FC was performed according to anesthetist indication and consisted of 500 ml of crystalloids infused over 10 minutes (responders = increase in cardiac index ≥ 15 %).

Bivariate analysis was performed using Student t test, Wilcoxon rank sum test, and Pearson's test as appropriate for continuous and categorical variables. Significance was set at p < 0.05. Within-group changes from baseline were analyzed by ANOVA with Bonferroni post hoc tests adjusting for multiple comparisons.

**Results** Overall, 42.5 % of patients were fluid responders. Baseline AUCs for PPV, CCE and P_dic_ were 0.76, 0.63 and 0.60, respectively. The bivariate analysis identified a model able to predict fluid responsiveness in 91 % of patients (AUC 0.92; Y = 24.9 + PPV*1.243 + Pdic*0.911 + CCEnor*0.004). In responders, the reduction of PPV during fluid challenge was statistically significant between baseline and minute 3 (p < 0.01), baseline and minute 4, 5 and 10 (p < 0.001). The increase of CCE during fluid challenge was statistically significant between baseline and minute 4 (p < 0.05). The increase of dicrotic pressure during fluid challenge was statistically significant between baseline minute 4 and 10 (p < 0.01), baseline and minute 5 (p < 0.001).

**Fig. 69:**
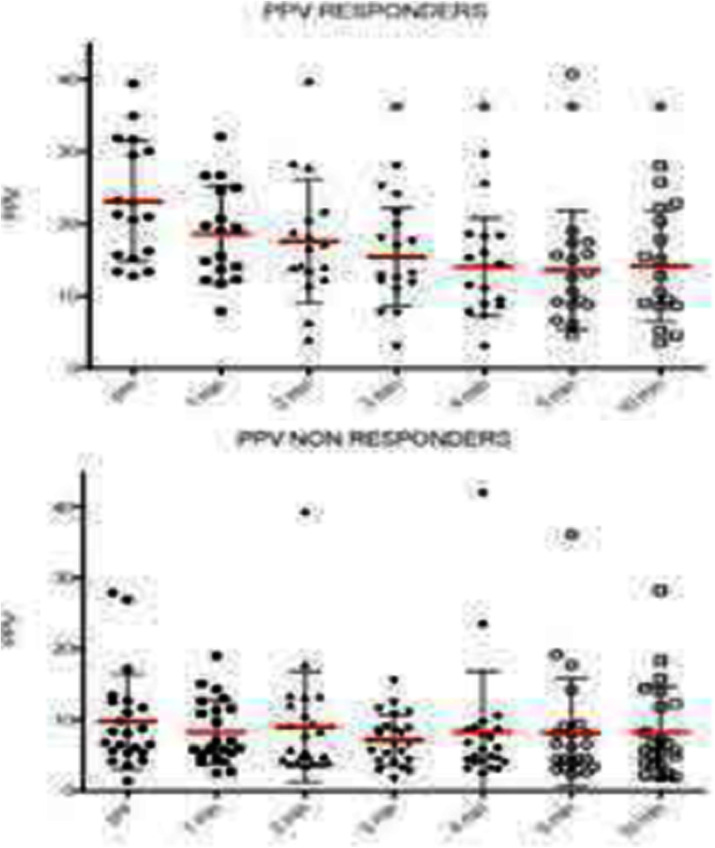
(abstract A160).

**Fig. 70:**
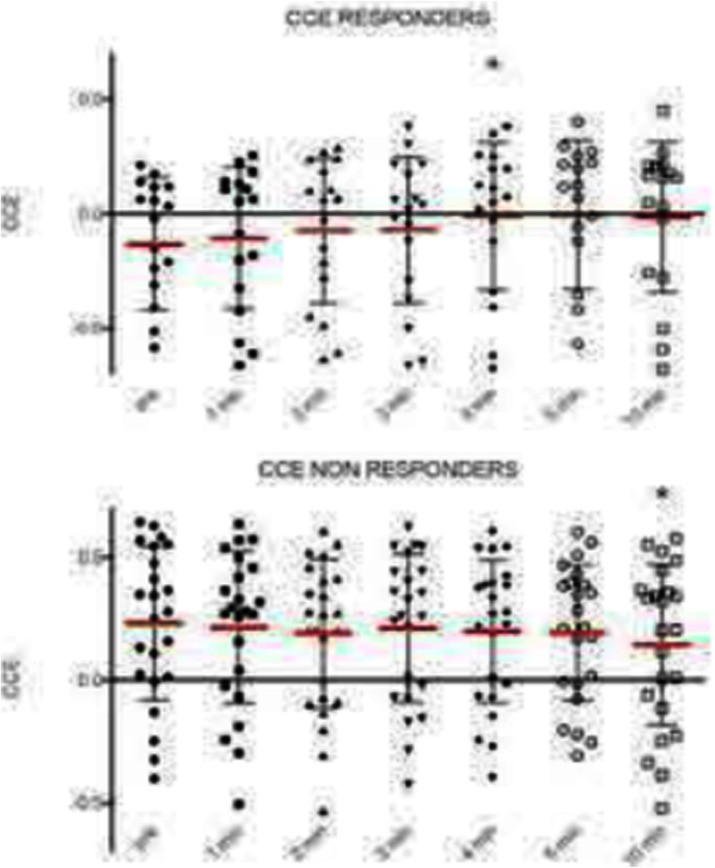
(abstract A160).

**Fig. 71:**
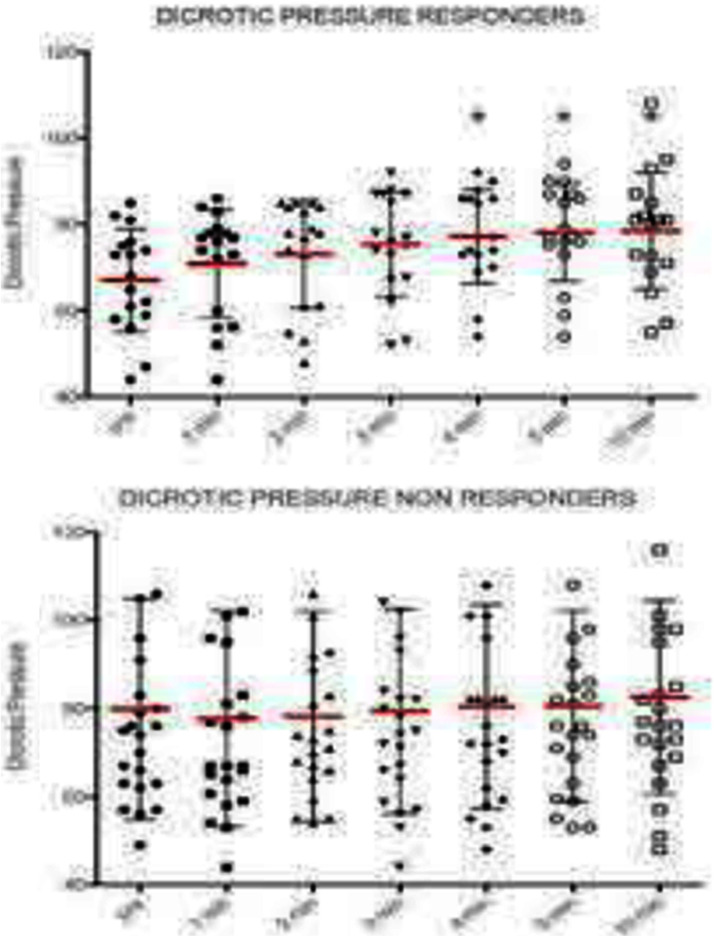
(abstract A160).

In non responders, PPV and dicrotic pressure did not change significantly during the FC while CCE was significantly reduced from baseline to minute 10 (p < 0.05) (Figs. 69, 70, 71).

**Discussion** In this pilot study the baseline values of CCE and P_dic_ significantly increased reliability of PPV. During the FC nor the PPV, neither P_dic_ significantly changed in non responders, while the CCE was reduced at the end of the FC. In responders, the reduction of PPV and the increase of CCE and P_dic_ were significant and the first time point of significance was minute 3 for PPV and P_dic_ and minute 4 for CCE, potentially acting as an early safety-limit for the FC.

**References**

1. A. Guldner et al. *Anesthesiology* 123(3):692-713, 2015.

2. J. Mallat et al. *Br J Anaesth* 115(3):449-56, 2015.

### A161 Conservative fluid management or deresuscitation for patients with sepsis or acute respiratory distress syndrome: a systematic review and meta-analysis

#### J.A. Silversides^1,2^, E. Major^1^, E.E. Mann^1^, A.J. Ferguson^3^, D.F. Mcauley^1,2^, J.C. Marshall^4^, B. Blackwood^2^, E. Fan^4^

##### ^1^Belfast Health and Social Care Trust, Critical Care, Belfast, United Kingdom; ^2^Queen's University of Belfast, Centre for Experimental Medicine, Belfast, United Kingdom; ^3^Southern Health and Social Care Trust, Anaesthetics and Intensive Care, Craigavon, United Kingdom; ^4^University of Toronto, Critical Care Medicine, Toronto, Canada

###### **Correspondence:** J.A. Silversides – Belfast Health and Social Care Trust, Critical Care, Belfast, United Kingdom

**Introduction** A positive fluid balance is associated with worse outcomes in the critically ill [1,2], and it has been suggested that conservative fluid administration or deresuscitation (active removal of fluid using diuretics or renal replacement therapy (RRT)) may be beneficial following haemodynamic stabilisation [3].

**Objectives** We performed a systematic review and meta-analysis to evaluate the efficacy and safety of conservative or deresuscitative fluid strategies in adults and children with acute respiratory distress syndrome (ARDS), sepsis, or systemic inflammatory response syndrome (SIRS) following initial resuscitation [4].

**Methods** We searched Medline, Embase, and the Cochrane central register of controlled trials without restrictions, and manually searched conference proceedings for the last 5 years. Two reviewers independently assessed publications. We included randomised controlled trials comparing two or more fluid regimens in which fluid balance differed, and observational studies investigating the relationship between fluid volume administered or fluid balance achieved and patient outcomes. We excluded studies published before 1980, studies of neonatal, post-cardiac surgical, or heart failure patients, and observational studies with fewer than 50 participants.

**Fig. 72 (abstract A161).**
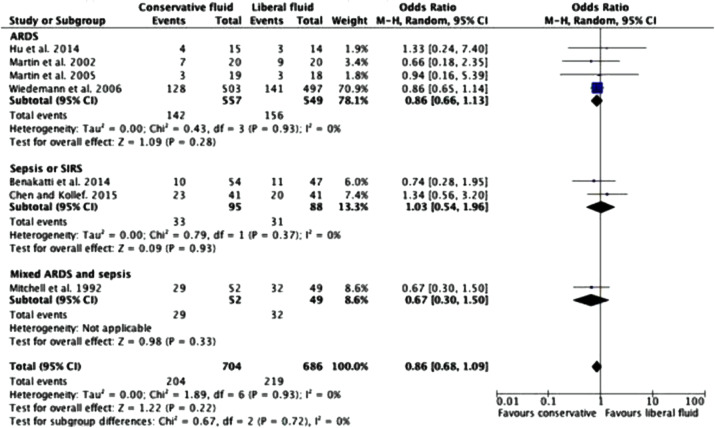
Mortality; conservative vs liberal

**Results** In a meta-analysis of the 7 included randomised trials (n = 1390), we found a non-significant reduction in mortality with a conservative or deresuscitative fluid strategy (pooled odds ratio 0.86, 95 % confidence interval (CI) 0.68-1.09) compared to a liberal strategy or usual care (Fig. 72). We found a non-significant reduction in RRT use with a conservative or deresuscitative fluid strategy (2 studies, pooled odds ratio 0.73, 95 % CI 0.51-1.05). Four trials reported shorter length of ICU stay or increased number of ICU-free days, and 2 studies reported shorter duration of mechanical ventilation or increased ventilator-free days with a conservative or deresuscitative fluid strategy compared to a liberal strategy or usual care. Marked clinical heterogeneity was present.

**Conclusions** A conservative or deresuscitative approach to fluid management may improve patient outcomes, and does not appear to increase the incidence of acute kidney injury or RRT use. Large randomised trials comparing alternative fluid regimens are needed to determine the optimal approach to fluid management in critically ill patients.

**Reference(s)**

1. Murphy CV et al. Chest. 2009;136:102-9.

2. Vincent JL et al. Crit Care Med. 2006;34:344-53.

3. Wiedemann HP et al. N Engl J Med. 2006;354:2564-75.

4. Silversides JA et al. Syst Rev. 2015;4:162.

**Grant acknowledgement**

This work was supported by a doctoral fellowship award to JS from Northern Ireland HSC research and development division.

### A162 Correlation between the distensibility index of inferior vena cava collapsability and pulse pressure variation in shock patients

#### J.A. Diaz-Rodriguez^1^, R. Silva-Medina^2^, E. Gomez-Sandoval^1^, N. Gomez-Gonzalez^1^, R. Soriano-Orozco^1^, P.L. Gonzalez-Carrillo^1^, M. Hernández-Flores^1^

##### ^1^Instituto Mexicano del Seguro Social, Intensive Care Unit, Leon, Mexico; ^2^Instituto Mexicano del Seguro Social, Radiology Unit, Leon, Mexico

###### **Correspondence:** J.A. Diaz-Rodriguez – Instituto Mexicano del Seguro Social, Intensive Care Unit, Leon, Mexico

**Introduction** Volume expansion is the first-line treatment in the majority of cases of acute circulatory failure, but only 50 % of patients in shock respond to fluid challenge (FC) in a conventional manner, the use of dynamic measurements can increase the prediction of this patients

**Objectives** The goal of this study was to determine the correlation of the distensibility index of inferior vena cava (dIVC) and pulse pressure variation (PPV) in patients with shock which received FC.

**Methods** This prospective, observational correlational study was conducted at the Intensive Care Unit, Centro Médico Nacional del Bajío UMAE 1, in León Guanajuato, between December 2014 to December 2015. Patients were admitted over 18 years, shock, mechanical ventilation and hypoperfusion (lactate, mean arterial pressure, urine output, and ΔPCO_2_). PPV and dIVC was measured in the admission, after the FC we analyzed hemodynamic variables. We excluded patients with arrhythmias, tidal volume >8 ml/kg, unconventional modes of mechanical ventilation. Statistical analysis was performed using SPSS program version 19.

**Results** We included 66 patients, mean age of 47.19 years, female gender 53 % male 47 %; the mean APACHE II and SOFA were 14 and 8 points respectively; more frequent admission diagnosis was sepsis (31.8 %); the most frequent hypoperfusion criteria was lactate >2 mmol/L (33.3 %); We found a positive correlation between dIVC and PPV with r = 0.642(p = < 0.05). After FC there was an increase of cardiac index 0.6196 ± 0.53 l/min/m2, a decrease of lactate 0.68 ± 0.49 mmol/L, an increase urine output of 0.26 ± 0.23 ml/kg/hour, and decrease ΔPCO_2_ 3.37 ± 1.7 mmHg with T 5.214(p = < 0.05), U 4.834(p = 0.009), U 3.057(p = 0.07) and U -5.863(p = < 0.05) respectively.

**Conclusions** There is a positive correlation between dIVC and PPV, in patients with shock which received FC in a conventional manner.

**Reference(s)**

1. Marik PE, Monnet X, Teboul JL. Hemodynamic parameters to guide fluid therapy. Marik et al. Annals of Intensive Care 2011, 1:1 2. Monnet X, Teboul JL. Assessment of volume responsiveness during mechanical ventilation: recent advances. *Critical Care* 2013, 17:217

3. Cecconi M, De Backer D, Antonelli M, Beale R, Bakker J, Hofer C. Consensus on circulatory shock and hemodynamic monitoring. Task force of the European Society of Intensive Care Medicine. Intensive Care Med (2014) 40:1795-1815

**Grant acknowledgement**

This study did not receive any grant from any funding agency.

**Fig. 73 (abstract A162).**
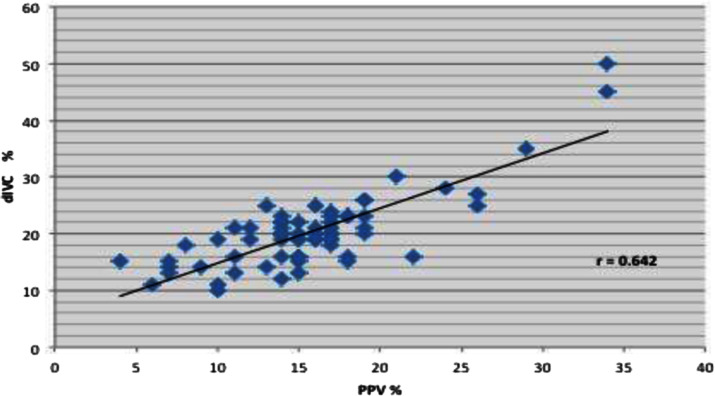
Correlation between distensibility index of inferior vena cava and pulse pressure variation

### A163 Prediction of fluid responsiveness after cardiac surgery with hemodynamic changes induced by peep-elevation

#### K. Pilarczyk^1,2^, J. Lubarksi^2^, D. Wendt^2^, F. Dusse^2,3^, J. Günter^2^, B. Huschens^2^, E. Demircioglu^2^, H. Jakob^2^

##### ^1^Imland Klinik Rendsburg, Department for Intensive Care Medicine, Rendsburg, Germany; ^2^University Hospital Essen, Department for Thoracic and Cardiovascular Surgery, Essen, Germany; ^3^University Witten/Herdecke, Medical Centre Cologne-Merheim, Department of Anaesthesiology and Intensive Care Medicine, Cologne, Germany

###### **Correspondence:** K. Pilarczyk – Imland Klinik Rendsburg, Department for Intensive Care Medicine, Rendsburg, Germany

**Introduction** In contrast to static parameters, e.g. central venous pressure (CVP), dynamic variables representing cardiorespiratory interactions, e.g. stroke volume variation, allow excellent prediction of fluid responsiveness (FR). A recently published study suggested that positive end-expiratory pressure (PEEP) induced changes of CVP or mean arterial pressure (MAP) might represent a promising tool to evaluate FR in ventilated patients with sepsis.

**Objectives** We evaluated the ability of hemodynamic changes produced by an increase in PEEP to predict FR in ventilated adult patients after cardiac surgery.

**Methods** 50 consecutive hemodynamically stable patients undergoing cardiac surgery with use of cardiopulmonary bypass were enrolled in this study. Hemodynamic monitoring included pulmonary artery catheter (PAC) and transesophageal echocardiography (TEE). Hemodynamic variables were assessed at 4 different time points: 1. Baseline (PEEP 6.0 ± 1.2 cm H_2_O), 2. PEEP challenge (PEEP + 10 cm H_2_O for 5 minutes), 3. Baseline 2 (with reduction of PEEP to initial value), 4. After a crystalloid fluid challenge of 6 ml/kgBW. Fluid responsiveness was defined as increase of stroke volume (SV) >15 % after fluid challenge measured by PAC.

**Results** 58 % of patients (n = 29) were fluid responders. The PEEP induced changes in CVP, MAP, pulse pressure (PP) and pulmonary capillary wedge pressure (PWP) were comparable between non-responders (Δ CVP 2.5 ± 5.2 mmHg, ΔMAP 4.1 ± 10.3 mmHg, ΔPP: 11.4 ± 15.2 mmHg, Δ PCWP 1.9 ± 3.4 mmHg ) and responders (Δ CVP 3.3 ± 4.5 mmHg, Δ MAP 7.6 ± 8.6 mmHg, ΔPP: 7.5 ± 17.1, ΔPCWP 3.7 ± 4.,1 mmHg) (p = n.s.). Accordingly, there was no correlation between PEEP-induced changes in CVP, MAP, PP and PCWP and changes in SV after a fluid challenge and the area under the curve (AUC) for predicting fluid responsiveness was low (CVP:0.584, MAP:0.653, PP: 0.557, PCWP:0.644). In contrast, increase in SV after PEEP challenge was significantly higher in fluid-responders than non-responders (8.6 ± 7.7 vs. 1.3 ± 12.1 ml, p = 0.023) predicting FR with an AUC of 0.750 (p < 0.001).

**Conclusion** Our data suggest that PEEP-induced changes of CVP, MAP, PP and PCWP in mechanically ventilated patients after cardiac surgery cannot predict fluid responsiveness whereas an increase of SV under PEEP elevation appears to be a good parameter to predict fluid responsiveness.

### A164 Fluid administration and mortality in septic shock patients: role of fluid balance and PH and electrolytes alterations

#### A. Palmaccio, A.M. Dell'Anna, D.L. Grieco, F. Torrini, C. Iaquaniello, F. Bongiovanni, M. Antonelli

##### Policlinico Universitario A. Gemelli, Anesthesia and Intensive Care Medicine, Roma, Italy

###### **Correspondence:** A. Palmaccio – Policlinico Universitario A. Gemelli, Anesthesia and Intensive Care Medicine, Roma, Italy

**Introduction** Fluid administration is one of the principal therapy adopted in order to achieve early haemodynamic stabilization in septic patients. Despite international guidelines promote rapid volume expansion in the early stages of shock [1], several studies have recently shown that excessive fluid balance in septic patients is correlated with increased mortality [1]

**Objectives** This study aims to investigate the impact of fluid balance on mortality in septic patients along with any associated electrolytes (strong ion difference-SID) and pH imbalance

**Methods** This pilot retrospective study enrolled approximately 10 % of the adult patients admitted to the intensive care unit (ICU) of the Gemelli University Hospital with a diagnosis of severe sepsis/septic shock. We excluded pregnant women and those who survived less than 48 hours. We collected all the data concerning daily fluid balance, arterial blood gases (ABG), the hemodynamics and daily SOFA, until day 28 of ICU stay or discharge. Fluid balance at 24 hours was divided into quartiles for subsequent analysis. Mortality was assessed at 90 days.

**Results** 61 patients were included in this pilot analysis from the 1^st^ January 2008 to 30 June 2015, age 66 ± 14 years , SAPS II 51 [IQR 38.75-64]. The 36 patients who died had higher blood lactate (4.3 ± 3.7 vs. 2.1 ± 1.7 mmol/L, p = 0.007), developed more frequently acute kidney injury (AKI) (44.4 % vs. 16 %, p = 0.027) and presented a more positive fluid balance at 48, 72 and 96 hours (2228 ± 539 vs. 784 ± 465 ml, p = 0.048; 2225 ± 824 vs. -181 ± 479 ml, p = 0.016; 2189 ± 922 vs. -475 ± 154.7 ml, p < 0.01, respectively). A non significant trend toward increased mortality in the 3^rd^ (dead 40 % vs. alive 60 %) and 4th quartile (dead 73.3 % vs alive 26.7 %) of fluid balance at 24 hours, was also found. No significant differences in terms of pH and electrolytic alterations have emerged, though a trend towards higher SID in the 2^nd^ quartile was evident (28 ± 6 vs. 31 ± 5 vs. 28 ± 5 vs. 27 ± 6 vs. 29 ± 6, respectively p = 0.13). Multivariate analysis, including SAPS II on, Charlson's comorbidity score AKI and blood lactate on admission confirmed the association between positive fluid balance and 90-day survival even after adjustment for several confounding factors (OR 0.54 96 % CI 0.30-0.96, p = 0.035).

**Conclusions** In our population positive fluid balance was independently associated with increased mortality at 90 days, but without any significant differences in terms of SID and pH.

**Reference(s)**

[1] Dellinger RP, et al., Surviving Sepsis Campaign Guidelines Committee including the Pediatric S, (2013) *Surviving sepsis campaign: international guidelines for management of severe sepsis and septic shock: 2012*. Critical care medicine 41: 580-637

[2] Boyd JH, et al., (2011) *Fluid resuscitation in septic shock: a positive fluid balance and elevated central venous pressure are associated with increased mortality*. Critical care medicine 39: 259-265

### A165 A systematic review and meta-analysis of the fluid challenge technique in anaesthesia and intensive care

#### L. Toscani^1,2^, D. Antonakaki^1^, D. Bastoni^1,3^, H.D. Aya^1^, A. Rhodes^1^, M. Cecconi^1^

##### ^1^St George's University Hospital, London, United Kingdom; ^2^Università La Sapienza di Roma, Rome, Italy; ^3^Università degli Studi di Parma, Parma, Italy

###### **Correspondence:** L. Toscani – St George's University Hospital, London, United Kingdom

**Introduction** The gold standard to evaluate fluid responsiveness and guide fluid administration, in both critically ill and surgical patients, is the fluid challenge technique (FC). Recent evidence has highlighted an important variability in the current practice of this technique. Different techniques may lead to different results and different clinical decisions.

**Objectives** The aim of this study is to describe the fluid challenge techniques reported in fluid responsiveness studies and clinical trials and to assess whether there is a difference in the proportion of “responders” depending on the type of fluid, volume and duration of infusion.

**Methods** We conducted a systematic review using Medline and EMBASE.

The inclusion criteria for the studies were: the use of the FC as a test of cardiac preload; the inclusion of a description of the FC ( reporting volume, type of fluid and duration of infusion); a reported definition of fluid responsiveness; a reported proportion of responders and non-responders;clinical setting(ICU or operative theatre).Included studies were examined in full and subjected to quantifiable analysis.The predictors were categorized as follows: volume (<500 ml, 500 ml and >500 ml); fluid type (colloids or crystalloids); infusion rate (<15 min, between 15 and 30 min, ≥30 min). The proportion of responders was compared.

**Results** 76 studies were included in the analysis. 7 studies(12 %)used less than 500 mL, 46 studies(79,3 %)used 500 mL and 5 studies(8,6 %) used more than 500 mL. The proportion of responders in the group of < 500 mL is .48(median:.49 IQR: .39-.57), in the group of 500 mL is .57(median = .56, IQR = .44-.68) and in the group of > 500 mL is .57 (median = .50, IQR = .38-.79). There is no evidence to suggest that the proportion of responders changes with the volume used (H = 1,79 p = .40).

23 (31,5 %)studies reported the use of crystalloids, whilst 50 (68,5 %) studies used colloids. In 3 studies the type of fluid was not clearly reported. There is no evidence to suggest that the proportion of responders changes between colloids and crystalloids (M = .53, SD = .15 vs M = .56, SD = .15, F(1,71) = .85, p = .35). The duration of infusion was <15 min in 19 studies(27,9 %), between 15 and 30 min in 23 studies(33,8 %) and ≥30 min in 26 studies (39,2 %).The proportion of responders changes across the different infusion times (F(2,65) =4,16 p = .02).Planned contrast revealed that the proportion of responders in the group of < 15 min(M = .60)and the group of 15 to 30 min(M = .57) were similar, but both were significantly different from the proportion of responders in the group with an infusion time ≥30 min (M = .49, F(2,65) = 4.17, p = 0.02).

**Conclusions** Most studies assessing fluid responsiveness use 500 mL of colloids infused over 30 minutes or more. A long infusion rate decrease the proportion of fluid responders.

**Reference**

Cecconi, Maurizio, et al. "Fluid challenges in intensive care: the FENICE study." Intensive care medicine 41.9 (2015):1529-1537.

**Grant acknowledgement**

SGUL

### A166 Predicting volume responsiveness by using combined end-expiratory and end-inspiratory occlusion tests with echocardiography and oesophageal Doppler

#### M. Jozwiak, F. Depret, J.-L. Teboul, J. Alphonsine, C. Lai, C. Richard, X. Monnet

##### Hôpital de Bicêtre, Hôpitaux Universitaires Paris-Sud, Université Paris-Sud, Service de Réanimation Médicale, Inserm UMR_S999, Le Kremlin Bicêtre, France

###### **Correspondence:** M. Jozwiak – Hôpital de Bicêtre, Hôpitaux Universitaires Paris-Sud, Université Paris-Sud, Service de Réanimation Médicale, Inserm UMR_S999, Le Kremlin Bicêtre, France

**Introduction** In patients under mechanical ventilation, end-expiratory and end-inspiratory occlusions induce changes in cardiac preload that may be used to test preload dependence.

**Objectives** To test whether volume responsiveness can be predicted by the effects of respiratory occlusions on the stroke volume (SV) estimated by transthoracic echocardiography or measured by oesophageal Doppler.

**Methods** In 35 mechanically ventilated patients, we measured the pulse contour analysis-derived cardiac index (PiCCO2). SV was estimated by the measure of the velocity-time integral of the left ventricular outflow tract by transthoracic echocardiography (n = 29) or measured by oesophageal Doppler (n = 6) during the 5 last seconds of 15-second end-inspiratory and end-expiratory occlusions, separated by 1 minute, and after the subsequent infusion of 500-mL saline. Patients where volume expansion induced an increase in cardiac index ≥15 % were defined as “volume-responders”.

**Results** Volume expansion increased cardiac index by more than 15 % (2.71 ± 0.78 to 3.27 ± 0.86 L/min/m^2^, p < 0.05) in 16 patients. During the end-expiratory occlusion, SV increased more in responders than in non-responders (increase by 10 ± 5 % *vs.* 2 ± 1 %, respectively, p < 0.0001). Similarly, during the end-inspiratory occlusion, SV decreased more in responders than in non-responders (decrease by 11 ± 5 % *vs.* 5 ± 3 %, respectively, p < 0.0001). Volume responsiveness was predicted by an end-expiratory-induced increase in SV > 4 % (sensitivity = 94 % [95 % confidence interval, CI: 70-100 %]; specificity = 100 % [95%CI: 82-100 %]). If the absolute values of SV changes recorded during end-expiratory plus end-inspiratory occlusions were added, volume responsiveness was predicted by an increase in SV >12 % (sensitivity = 94 % [95%CI: 70-100 %]; specificity = 95 % [95%CI: 74-100 %]). This predictive accuracy for volume responsiveness was not better than for the SV changes recorded during the only end-expiratory occlusion (area under the ROC curve: 0.98 (95%CI: 0.86-1.00) *vs.* 0.94 (95%CI: 0.81-0.99), respectively, p = 0.46).

**Conclusions** The sum of the absolute values of changes in SV recorded during consecutive end-expiratory and end-inspiratory occlusions reliably predict volume responsiveness. This prediction is not better than for the changes in SV during the only end-expiratory occlusion. Nevertheless, combining end-expiratory and end-inspiratory occlusions induces larger SV changes and increases the value of the threshold, what is more compatible with the precision of echocardiography and oesophageal Doppler.

### A167 Physiological volume replacement ratio can be reached in experimental hemorrhage model

#### I. László^1^, G. Demeter^1^, N. Öveges^1^, K. Tánczos^1^, M. Németh^1^, D. Trásy^1^, I. Kertmegi^2^, D. Érces^2^, B. Tudor^3^, J. Kaszaki^2^, Z. Molnár^1^

##### ^1^University of Szeged, Department of Anesthesiology and Intensive Therapy, Szeged, Hungary; ^2^University of Szeged, Institute of Surgical Research, Szeged, Hungary; ^3^Medical University of Vienna, Department of Anaesthesiology and General Intensive Care Medicine, Vienna, Austria

###### **Correspondence:** I. László – University of Szeged, Department of Anesthesiology and Intensive Therapy, Szeged, Hungary

**Introduction** Acute bleeding is a life threatening condition requiring immediate and adequate interventions. Adequate fluid resuscitation is the cornerstone of maintaining and correcting oxygen delivery (1). According to Starling's “3-compartment model”, 4-times more crystalloids should have the same volume effect as colloids. However, this volume-replacement ratio remains a controversial issue as this may be affected by the degradation of the endothelial glycocalyx layer often found in the critically ill.

**Objectives** Our aim was to compare colloid and crystalloid based fluid resuscitation during an experimental stroke volume index (SVI) guided hemorrhage and resuscitation model in Vietnamese mini-pigs.

**Methods** In this experiment 15 anesthetized and mechanically ventilated pigs were randomized to receive colloid (Voluven®,HES, n = 7) or crystalloid (Ringerfundin®,RF, n = 8) infusion. Animals were bled till baseline SVI (Tbsl) dropped by 50 % (T0), followed by resuscitation until initial SVI was reached (T4) in four steps. Statistics were performed by using SPSS® 23.0 and statistical analysis were tested by General Linear Model, Independent samples T-test and Mann-Whitney U test, as appropriate.

**Results** Hemodynamic changes during the experiment did not show clinically relevant differences between the groups. At Tbsl the SVI values were similar (HES: 34 ± 8 ml/m^2^, RF: 33 ± 4 ml/m^2^), after bleeding, the SVI decreased by the planned 50 % to T0 (HES: 17 ± 4, RF: 15 ± 2 ml/m^2^) and returned to its initial value by T4 (HES: 34 ± 7, RF: 32 ± 3 ml/m^2^). Cardiac index(CI) also decreased (Tbsl, HES: 3.25 ± 0.23, RF: 3.14 ± 0.19 l/min/m^2^; T0, HES: 1.58 ± 0.27, RF: 1.84 ± 0.40 l/min/m^2^) and reached a higher value by T4 (HES: 3.99 ± 0.54, RF: 3.39 ± 0.36 l/min/m^2^, p = 0,006). There was a significant increase in heart rate over time (Tbsl, HES: 95 ± 19; RF: 97 ± 18 beats/min; T4, HES: 117 ± 17, RF: 102 ± 14 beats/min, p‹0.05). Similar amount of blood was shed in both groups (HES: 553 ± 206 ml, RF: 506 ± 107 ml) but the animals received significantly less resuscitation fluid (623 ± 208, 1754 ± 602 ml, p = 0.002, respectively) and total fluid in the HES-group (1029 ± 252, 2010 ± 600 ml, p = 0.006, respectively). The volume replacement ratio was significantly different between the HES median and RF-groups: median = 0.87 [IQR: 0.78-1.83], 3.06 [2.93-4.57], p = 0,002, respectively).

**Conclusions** Our results showed that in healthy pigs the volume-replacement ratio follows the Starling's principle. This indicates that in acute bleeding events, like in trauma and during surgery when the glycocalix is most likely to be still intact, colloids may be beneficial as hemodynamic stability can be achieved faster than with crystalloids.

**Reference(s)**

1. Tánczos K, Németh M, Trásy D, et al. Goal-Directed Resuscitation Aiming Cardiac Index Masks Residual Hypovolemia: An Animal Experiment. Biomed Res Int 2015;2015:160979.

**Grant acknowledgement**

Supported by NKFIH K116689.

### A168 The ability of venous-arterial carbon dioxide gap and central venous oxygen saturation to predict fluid responsiveness by passive leg raising test

#### A. Hasanin, A. Lotfy, A. El-adawy, H. Nassar, S. Mahmoud, A. Abougabal, A. Mukhtar

##### Cairo University, Department of Anesthesia and Critical Care Medicine, Cairo, Egypt

###### **Correspondence:** A. Abougabal – Cairo University, Department of Anesthesia and Critical Care Medicine, Cairo, Egypt

**Introduction** Passive leg raising (PLR) test has been a popular method used for detection of fluid responsiveness (FR) in patients with acute circulatory failure. The most important limitation with PLR test is the need to a real-time cardiac output (CO) monitor to trace the patient hemodynamic response to PLR. Increased end-tidal Co_2_ with PLR has been reported as a surrogate of increased CO in prediction of FR (1), however, the use of end-tidal Co_2_ is limited to mechanically ventilated patients. Increased mixed venous oxygen saturation was another surrogate of increased CO in prediction of FR with PLR (2), however, this parameter needs insertion of pulmonary artery catheter. Although central venous oxygen saturation (Scvo_2_) has been a surrogate of mixed venous oxygen saturation, however Scvo_2_ was not investigated in prediction of FR. Venous-Arterial Co_2_ gap (VA-Co_2_ gap) is defined as the difference between central venous and arterial Co_2_ partial pressure. VA-Co_2_ gap increases in cases of tissue hypoperfusion (3) and decreases after CO improvement.

**Objectives** We investigated the role of the change in VA-Co_2_ gap and the change in Scvo_2_ after PLR in prediction of FR

**Methods** We included 42 patients with acute circulatory failure with elevated serum lactate. PLR test was performed to detect FR. Fluid responders were defined as patients with increased stroke volume by 10 % (measured by ICON cardiometry device) after PLR test. In addition to demographic and hemodynamic data, arterial and central venous blood gases were obtained before and after PLR. The predictive ability of the change in VA-Co2 gap and the change in Scvo_2_ with PLR to predict FR was obtained using area under receiver operating characteristic (AUROC) curve.

**Results** Fluid responders were 11 patients (26 %). AUROC for the change in VA-Co_2_ gap in prediction of FR was 0.786(95 % CI: 0.625-0.895). Sensitivity was 73 % and specificity was 65 % at cutoff value of 0.5 mmHg increase in VA-Co_2_ gap. AUROC for the change in Scvo_2_ in prediction of FR was 0.608(95 % CI: 0.443-0.756). Sensitivity was 64 % and specificity was 63 % at cutoff value of 2 % decrease in Scvo_2_.

**Conclusions** The change in VA-Co_2_ gap with PLR is superior to the change in Scvo_2_ in prediction of FR in patients with acute circulatory failure. VA-Co_2_ gap can be considered in settings where CO monitoring is not feasible.

**References**

1. Monnet X, Bataille A, Magalhaes E, Barrois J, Le Corre M, Gosset C, et al. End-tidal carbon dioxide is better than arterial pressure for predicting volume responsiveness by the passive leg raising test. Intensive Care Med 2013;39:93-100.

2. Kuiper et al. Mixed venous O2 saturation and fluid responsiveness after cardiac or major vascular surgery. J Cardiothorac Surg 2013;8:189

3. Robin E, Futier E, Pires O, Fleyfel M, Tavernier B, Lebuffe G, et al. Central venous-to-arterial carbon dioxide difference as a prognostic tool in high-risk surgical patients. Crit Care 2015;19:227.

**Fig. 74 (abstract A168).**
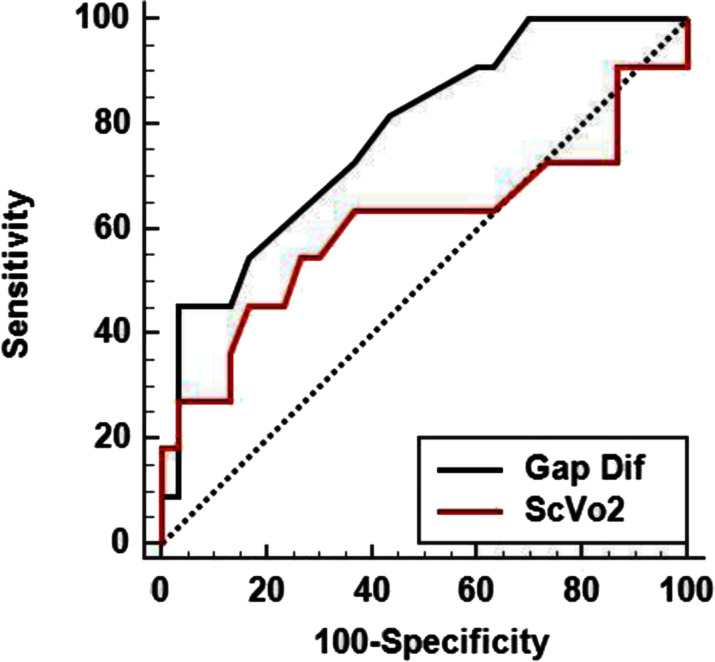
AUROC for Co2 Gap and ScVo2 to predict FR

### A169 Carotid doppler to predict fluid responsiveness in intensive care unit: a mini fluid challenge study

#### F. Quinty^1^, S. Habchi^2^, A. Luzi^3^, E. Antok^1^

##### ^1^University of la Reunion, Intensive Care Unit, Saint Pierre de la Reunion, France; ^2^University of la Reunion, Neurocritical Care Unit, Saint Pierre de la Reunion, France; ^3^University of la Reunion, Anesthesiology Department, Saint Pierre de la Reunion, France

###### **Correspondence:** F. Quinty – University of la Reunion, Intensive Care Unit, Saint Pierre de la Reunion, France

**Introduction** Fluid management is one of the most difficult tasks in critical care medicine. A recent study shows that in current practice, fluid administration is not evidence based despite a lot of tools available for physician (1). However many of them are invasive or not always applicable depending on clinical context. Carotid doppler, a non invasive and easy-to-use method, has shown excellent predictive values to monitor fluid responsiveness after passive leg raising (2). Otherwise, mini fluid challenge is described as a reliable alternative to classical fluid challenge to prevent fluid overload.

**Objectives** To assess if variation of carotid doppler flow can predict fluid responsiveness after a mini fluid challenge.

**Methods** This prospective observational study was performed from September to December 2015. Patients requiring volume expansion were eligible for enrollment. Patients less than 18 years old, with carotid stenosis or poor insonation were excluded. We recorded the variation of carotid doppler flow after 100 cc of cristalloids (ΔDc100) over 1 min and the variation of velocity time integral (VTI) after an additional infusion of 400 cc over 14 min assessed by transthoracic echocardiography. The cardiac output (CO) was calculated as CO = VTI x Heart Rate x Aortic Surface . A patient whose CO increased by 15 % following 500 cc (ΔCO500) was consider as a fluid responder. If mechanical ventilation was required, a lung protective strategy was applied.

Statistics: Spearman's correlation test was used. The receiver operating curve (ROC) and grey zone were defined for ΔDc100.

**Results** In total, 30 patients were included. Diagnosis admission were severe sepsis/septic shock (22), brain injury (4) and post operative (4). Sixty five percent of patients were ventilated and 45 % required vasopressor support. There was no difference between responders and non responders. Fourteen patients (45 %) were volume responders. Dc100 increased by 32 % +/- 24 % in the responders compared with 10 % +/- 8 % in the non responders (p < 0.001). ΔDc100 was strongly correlated with ΔCO500 (r 0,78 ; p < 0,001). The best threshold of ΔDc100 was 14 % with a sensitivity and specificity of 93 % and 82 % respectively.

The areas under the ROC curve of ΔDc100 was 0,91 +/- 0,01. After bootstrapping (1000 resamples) a grey zone ranging between 11 and 20 % was observed in up to 29 % of patients.

**Conclusion** Our study shows that variation of carotid doppler after a mini fluid challenge can predict fluid responsiveness in heterogeneous critical care population.

**Reference(s)**

(1) Cecconi M, Hofer C, Teboul JL et al (2015) Fluid challenges in intensive care: the FENICE study: A global inception cohort study. Intensive Care Med ; 41(9):1529-37

(2) Marik PE, Levitov A, Young A et al (2013). The use of bioreactance and carotid Doppler to determine volume responsiveness and blood flow redistribution following passive leg raising in hemodynamically unstable patients. Chest ; 143:364-70

### A170 Peripheral perfusion response to the first fluid resuscitation predicts mortality in patients with sepsis-related acute circulatory dysfunction admitted to the emergency department

#### G. Hernandez^1^, B. Lara^2^, L. Enberg^2^, M. Ortega^1^, P. Leon^1^, C. Kripper^2^, P. Aguilera^2^, E. Kattan^1^, J. Bakker^1^

##### ^1^Pontificia Universidad Catolica de Chile, Facultad de Medicina, Departamento de Medicina Intensiva, Santiago, Chile; ^2^Pontificia Universidad Catolica de Chile, Programa de Medicina de Urgencia, Facultad de Medicina, Santiago, Chile

###### **Correspondence:** G. Hernandez – Pontificia Universidad Catolica de Chile, Facultad de Medicina, Departamento de Medicina Intensiva, Santiago, Chile

**Background** Sepsis-related acute circulatory dysfunction is a life threatening condition. Peripheral perfusion as a marker of hypoperfusion could be used to trigger initial fluid resuscitation (FR). The response of peripheral perfusion to FR in patients with abnormal perfusion could potentially identify high-risk patients.

**Objective** Our aim was to study the effects of the first protocolized FR on capillary refill time (CRT) and other perfusion parameters, and the relationship of the response with outcome.

**Methods** Prospective observational study including patients with sepsis and acute circulatory dysfunction just admitted to the Emergency department (ED) and in whom an initial FR was indicated according to standard care.

Peripheral perfusion and laboratory assessments were performed before and after protocolized FR. Follow-up of patients until hospital discharge. CRT responders were defined as patients who were able to maintain normal CRT or to normalize abnormal CRT values after FR.

**Results** One hundred consecutive patients were included. Of 30 patients with an abnormal CRT at admission, 23 (77 %) normalized CRT after initial FR. CRT responders showed a significant decrease in heart rate and lactate, presented less organ dysfunction and requirement of mechanical ventilation. Hospital mortality was significantly lower in CRT responders when compared to non-responders (9.6 % vs. 55.6 %; p < 0.001). In logistic regression analysis only CRT was significantly related to hospital mortality. This association was maintained after adjusting for baseline severity.

**Conclusions** Patients with sepsis-related acute circulatory dysfunction that exhibit normal CRT after early FR have low mortality rates. In contrast, failure to improve peripheral perfusion in response to initial FR is a strong predictor of mortality. This finding could be very important for the ED or limited-resource settings since it could help to decide on additional diagnostic and treatment options.

### A171 Prediction of fluid responsiveness in patients with assisted mechanical ventilation: a comparison of the "fluid responsiveness index" FRI to CVP, global end-diastolic volume index gedvi and stroke volume variation SVV

#### W. Huber^1^, M. Lehmann^1^, S. Sakka^2^, B. Bein^3^, R.M. Schmid^1^

##### ^1^Technische Universität München, II. Medizinische Klinik, Munich, Germany; ^2^Klinikum Köln-Merheim, Cologne, Germany; ^3^Asklepios Klinik St. Georg, Hamburg, Ghana

###### **Correspondence:** W. Huber – Technische Universität München, II. Medizinische Klinik, Munich, Germany

**Introduction** Appropriate fluid support is crucial in critical care. To guide fluid resuscitation filling pressures such as central venous pressure CVP, volumetric parameters (e.g. global end-diastolic volume index GEDVI) and dynamic parameters of fluid responsiveness (FR) like stroke volume variation SVV are used. Prediction of FR is particular difficult in patients with spontaneous breathing, since the use of SVV is usually restricted to patients under controlled ventilation. Under these circumstances passive leg raising PLR and “mini volume challenges” can be used for intermittent assessment of FR. Recently “fluid responsiveness index” FRI has been introduced as experimental marker of FR. FRI is derived from a proprietary algorithm based on the continuous analysis of arterial and central-venous pressures by a special software provided for scientific use with the PiCCO-2 device (Pulsion Medical Systems SE, Feldkirchen, Germany).

**Objectives** It was the aim of our study to compare the prediction of FR (defined as an increase in stroke volume index ≥10 %) by FRI, GEDVI, CVP and SVV after a volume challenge (VC) with 7 mL/kg crystalloid within ≤30 minutes in 27 patients under assisted mechanical ventilation and equipped with TPTD-monitoring irrespective of the study.

**Methods** All haemodynamic parameters were measured immediately before and after the VC with a modified PiCCO-2-device providing continuous FRI. The study was approved by the local Ethics Committee. Statistics: SPSS 23.

**Results** 20 male, 7 female patients; APACHE-II 25 ± 6. FRI (ROC-AUC 0.881; p = 0.005) and GEDVI (AUC 0.778; p = 0.041) significantly predicted an increase in stroke volume index ≥10 %, whereas neither CVP (AUC 0.675; p = 0.199) nor SVV were predictive (AUC 0.702; p = 0.137). FRI was significantly higher in fluid-responders than in non-responders (0.60 ± 0.20 vs 0.20 ± 024; p = 0.003), while GEDVI was significantly lower in responders (657 ± 109 vs. 797 ± 170 mL/m^2^; p = 0.042). CVP and SVV were not different between responders and non-responders. A cut-off of 0.42 for FRI-provided a sensitivity of 83 % and a specificity of 81 % regarding an increase in stroke volume index ≥10 %. GEDVI values below 800 mL/m^2^ predicted FR with a sensitivity of 83 % and a specificity of 62 %. In binary regression analysis only FRI (p = 0.015) was independently associated to the primary endpoint.

Similarly, FRI (AUC 0.827; p = 0.025) and GEDVI (AUC 0.809; p = 0.034) predicted an increase in CI ≥10 % after VC, whereas CVP and SVV were not predictive. Finally, only FRI predicted an increase in cardiac power index CPI ≥10 % (AUC 0.812; p = 0.008).

**Conclusions** FRI significantly and independently predicts FR in patients with spontaneous assisted ventilation. Furthermore, GEDVI is significantly associated to FR in these patients, while CVP and SVV were not predictive. With regard to the limited number of patients included, confirmatory validation studies are required.

### A172 The use of venous return gradient to predict fluid responsiveness in critically ill patients

#### J. Preti, J. Creteur, A. Herpain

##### Université Libre de Bruxelles, Brussels, Belgium

###### **Correspondence:** J. Preti – Université Libre de Bruxelles, Brussels, Belgium

**Introduction** Fluid expansion (FE) is a major issue in the management of critically ill patients. Accurate and practical tools to predict fluid responsiveness are still lacking. Venous return gradient (dVR) is a major component of venous return and therefore of right heart preload and cardiac output. dVR is the difference between mean systemic filling pressure (Pmsf) and central venous pressure (CVP) or right atrial pressure (RAP). Pmsf can be estimated by measuring the static filling pressure of the arm (Parm). This later has shown to be a useful predictor of fluid responsiveness in cardiac surgery patients [1].

**Objective** The aim of the study was to evaluate if dVR is a reliable variable to predict fluid responsiveness.

**Methods** Twenty-nine critically ill patients requiring FE were included in this prospective observational study. In each patient, Parm was measured three times before FE from the arterial pressure curve obtained from a radial artery catheter 30 sec after occluding the arterial flow by a cuff inflated up to 50 mmHg above systolic blood pressure. The average value of triplicate measurements was calculated. Stroke volume was estimated either by Swan-Ganz catheter or transthoracic echocardiography. A positive response to the fluid expansion was defined as a SV increased by at least 10 %. The optimal cut offs were chosen using a receiver operating characteristic curve (ROC) analysis and identifying the maximal Youden's index. Sensitivity, specificity, and their approximate 95 % confidence intervals were computed.

**Results** In the group of patients who responded to the fluid expansion (n = 19), the mean CVP/RAP was 8 mmHg ±3, the mean Parm was 21 mmHg ±7 and the mean dVR was 13 mmHg ±7. In non-responders patients (n = 10), the mean CVP/RAP was 8 mmHg ±3, the mean Parm was 26 mmHg ±6 and the mean dVR was 18 mmHg ±6. The best parameter in order to predict fluid responsiveness was dVR, with an area under the curve of 0,77 (95 % confidence interval 0,57-0,97); a dVR cut-off of < 15 mmHg predicting a successful FE with a sensitivity of 74 % and a specificity of 90 %. In comparison, for Parm, the area under the curve was 0,73 (95 % confidence interval 0,54-0,92); a Parm cut-off of < 24 mmHg predicting a successful FE with a sensitivity of 74 % and a specificity of 70 %.

**Conclusion** Venous return gradient estimated by arm occlusion pressure is at least as good as Parm for the prediction of fluid responsiveness in critically ill patients.

**Reference(s)**

[1] Geerts, B.F., et al., Arm occlusion pressure is a useful predictor of an increase in cardiac output after fluid loading following cardiac surgery. Eur J Anaesthesiol, 2011.

**Grant acknowledgement**

None

### A173 The effectiveness of echocardiography to evaluate preload dependency in septic shock with left systolic heart failure - dyspred study

#### J. Marc^1^, E. Zogheib^1^, F. Trojette^1^, S. Bar^1^, L. Kontar^1^, D. Titeca^1^, J. Richecoeur^2^, B. Gelee^2^, N. Verrier^2^, R. Mercier^2^, E. Lorne^1^, J. Maizel^1^, H. Dupont^1^, M. Slama^1^

##### ^1^Univ. Hospital of Amiens, Amiens, France; ^2^CH Beauvais, Beauvais, France

###### **Correspondence:** J. Marc – Univ. Hospital of Amiens, Amiens, France

**Introduction** The ability of echocardiography and analysis of mitral profile to predict fluid responsiveness in a septic shock with left systolic heart failure is difficult to manage.

**Objective** The objective of the study was to evaluate the ability of mitral profile and its evolution with a test of passive leg raising to discriminate fluid responsiveness in septic shock with left systolic heart failure.

**Methods** 60 patients in septic shock and left systolic failure, monitored by transthoracic echocardiography (TTE) and continuous measurement of cardiac output (CO) (catheter pulmonary artery catheter (PAC) or transpulmonary thermodilution (TPTD)) were included. Mitral profile (E, A, E/A, E´ lateral, E´ septal, E´ average, E/E´), CO, pulmonary artery occlusion pressure (PAOP) and extravascular lung water (EVLW)) were collected before volume expansion (VE), after passive leg raising (PLR) and after VE with 500 ml of crystalloid solution. Variation of each hemodynamic values after VE (Δ(VE)) and after PLR (Δ(PLR)) was performed. The left systolic heart failure was defined with left ventricle ejection fraction (LVEF) ≤ 40 %. Patients were classified into two groups according to their response after VE measured by thermodilution: responders (R) defined by an increase ≥ 15 % of CO, and non-responders (NR).

**Results** Of the 60 patients monitored by TTE, 28 (46 %) with also TPTD and 32 (54 %) with PAC. 25 (42 %) were R and 35 (58 %) NR. All were under norepinephrine (1.1 gamma/kg/min +/- 0.4). There was no significant difference between R and NR with E, A, E/A (1.67 +/- 0.85 vs 1.40 +/- 0.97), E´ average (7.5 +/- 2.5 vs 7.1 +/- 2.8 ), E/E´ average (12.5 +/- 6.1 vs 13.5 + / 7.5). It was not found correlation between the E/E ´and PAOP (p = 0.48) nor with the EVLW (p = 0.78). 33 % (10/30) of NR had an E/E´ lat < 8 and 28 % (9/25) of R had an E/E´ lat > 15. ΔCO(VE) (p < 0.05) and ΔE ´ average(VE) (p < 0.05) were higher in the R while ΔE/E´ lat (VE) (p < 0.05) and ΔE/E ´ average (VE) (p < 0.05) were higher in NR. ΔE´(VE) was correlated with the ΔCO(VE) independently with LVEF (r = 0.39 p < 0.05). He was found a positive correlation between ΔCO(EV) and ΔCO(PLR) (r = 0.58 p < 0.05). The ΔCO(PLR) AUC with an optimal threshold at 12 % to predicted fluid responsiveness was 0.890 p < 0.05) with 92 % sensitivity and 85 % specificity. ΔE/E ´average(PLR) was correlated with ΔE/E´ average(VE) (r = 0.29 p < 0.05).

**Conclusions** In decompensated patients with septic shock and left systolic heart failure, analysis of mitral profile alone not be reliable in predicting left filling pressure nor in predicting fluid responsiveness. The PLR and the variation of the mitral profile (ΔE/E´ average and ΔE´ lat) would secure VE in this population.

## Cardiovascular monitoring 2

### A174 The effect of fluid balance on extra-vascular lung water assessed by lung ultrasound & electrical cardiometry: a prospective cohort study

#### M.E. Abdelfattah^1^, A. Eladawy^2^, M.A. Ali Elsayed^1^, A. Mukhtar^1^

##### ^1^Cairo University, Cairo, Egypt; ^2^Kasr Alainy Medical School, Cairo University, Cairo, Egypt

###### **Correspondence:** A. Eladawy – Kasr Alainy Medical School, Cairo University, Cairo, Egypt

**Introduction** Fluid balance is one of the most frequently manipulated clinical care variables in the ICU. The risks associated with invasive monitoring and its relationship to heightened mortality make the evaluation and utilization of other modalities for tracking volume status in critically ill patients vitally important.^1^

**Objectives** To correlate the three day cumulative fluid balance (CFB) with extravascular lung water assessed by LUS & electrical cardiometry by the end of the 3^rd^ day & to assess the diagnostic accuracy of electrical cardiometry in assessment of thoracic fluid content (TFC) in comparison to lung ultrasound.

**Methods** Three day CFB was measured in the 1^st^ three days of ICU stay. Lung ultrasound score was obtained by scanning 12-rib interspaces. & the sum of B-lines yielded a score (0-36) ^2^. Electrical cardiometry was used to assess TFC (considered abnormal if exceeding a predetermined value). E/e´ ratio was done to assess fluid load; for further correlation with CFB, lung score, & thoracic fluid content (TFC). All measurements were done on day 1 & day 3. Other variables collected were age, gender, cardiac output, APACHE II score, medical or surgical patient, mechanical ventilation, length of ICU stay & ICU mortality.

**Results** 30 patients were enrolled. The median (IQR) cumulative 3-day fluid balance was -600 (-2225, 437). Ten patients (33.3 %) had positive fluid balance & 20 patients (67.7 %) had negative fluid balance. The median (IQR) of TFC at day 1 was 52 (35.5, 58) and at day 3 was 46 (37, 52.75).

There was no significant correlation between 3-day cumulative fluid balance with either LUS, Ee´ ratio, or TFC (Table 56).

There was a significant correlation between TFC & LUS on day 1 & day 3 (r = 0.610, p < 0.01), (r = 0.4, p = 0.05) respectively. There was no relationship between Ee´ ratio & both TFC & LUS on day 1, it became significant on day 3 (Table 57).

TFC correlated significantly with LUS in patients with negative fluid balance but not in those with positive fluid balance. By the same token, LUS was correlated significantly with Ee´ in patients with negative balance only. However, no correlation was found between TFC & Ee´ in patients with either negative or positive fluid balance (Table 58)**.**

**Conclusions** There is a good relationship between TFC & LUS for assessment of extra-vascular lung water. However the relationship between CFB & extra-vascular lung water is poor. Future larger studies are warranted to develop predictive equation for assessment of the amount of extravascular lung water from TFC.

**Reference(s)**

1. Martin GS, Ely EW, Carroll FE, Bernard GR. Findings on the Portable Chest Radiograph Correlate With Fluid Balance in Critically Ill Patients. 2007; 2087-95.

2. Volpicelli G, Mussa A, Garofalo G, Cardinale L, Casoli G, Perotto F, et al. Bedside lung ultrasound in the assessment of alveolar-interstitial syndrome. Am J Emerg Med. 2006 Oct;24(6):689-96.

**Table 56 (abstract A174).** Correlation between CFB, TFC, Ee' (Day 3)

Cumulative Fluid balance		
	r-value	p-value
TFC	0.25	0.18
LUS	-0.107	0.57
Ee´	-0.002	0.99

**Table 57 (abstract A174).** Correlation between TFC, Ee' & LUS (Day 1 & Day 3)

	Day 1		Day 3	
	r-value	p-value	r-value	p-value
TFC & LUS	0.610	<0.01	0.358	0.052
TFC and Ee´	0.287	0.124	0.393	0.032
LUS and Ee´	0.242	0.198	0.426	0.019

**Table 58 (abstract A174).** Correlation between TFC, LUS, Ee' (Day 3)

	Positive- balanced patients (n=10)		Negative balanced patients (n=20)	
	r-value	p-value	r-value	p-value
TFC & LUS	0.186	0.6	0.566	0.009
TFC & Ee´ratio	0.628	0.052	0.283	0.227
LUS & Ee´ratio	0.256	0.47	0.516	0.02

### A175 Estimation of maximal oxygen uptake (VO2max), as a prognostic marker in patients with sepsis and septic shock in intensive care unit

#### A. Pedraza Montenegro, E. Monares Zepeda, J. Franco Granillo, J.S. Aguirre Sánchez, G. Camarena Alejo, A. Rugerio Cabrera, A.A. Tanaka Montoya

##### ABC Medical Center, Critical Medicine, Mexico City, Mexico

###### **Correspondence:** A. Pedraza Montenegro – ABC Medical Center, Critical Medicine, Mexico City, Mexico

**Introduction** Obtaining the maximum oxygen consumption (VO2max) in a direct way, requires equipment and trained personnel, which is not available in most intensive care units. We modify the formula from Uths Niels et al: VO2max = 15 x (maximum heart rate during exercise / heart rate at rest), replacing the maximum rate during exercise to maximum heart rate for age and frequency at rest for heart rate 24 hours from admission to intensive care unit. VO2max modified = 15 x maximum heart rate for age / heart rate at 24 hours.

**Objective** To evaluate the usefulness of maximum oxygen uptake determination in patients with sepsis and septic shock as a prognostic marker.

**Methods** Cohort, prospective, longitudinal, analytical study. Patients over 18 years old, admitted to ICU of ABC Medical Center with diagnosis of sepsis. The maximum oxygen consumption at admission of patients with sepsis was calculated, and compared according groups based on VO2max modified. A bivariate analysis was performed, using Chi2, ROC curve and relative risk.

**Results** 78 patients were analyzed, 42 men (54 %), aged 68+ 16 years old, with 57 % primary site of infection from the lungs, followed by urinary ( 19 % ), with MODS 6 ± 3 points, 39 % and 61 % with sepsis and septic shock respectively, 21 patients died (27 %). ROC curve was obtained for VO2max at 24 hours modified, with an AUC 0.70, CI from 0.64 to 0.89; p = 0.03, with a cutoff of 25 ml/kg/min, sensitivity and specificity of 70 % to 70 % respectively. Patients with VO2max higher than 25 ml/kg/min have a RR of 0.4 (CI 0.2-0.7) for mortality at 30 days.

**Conclusions** Modified VO2max could be a useful tool to identify septic patients with adequate reanimation. This trial suggests that a VO2max value calculated at 24 hours from ICU admission, greater than 25 ml/kg/min is a reflection of good reanimation, however; future researches are needed to corroborate the prognostic utility and to set a goal of reanimation.

**Bibliography:**

1. Uth, N., Sorensen, H., et al. (2004). Estimation of VO2max from the ratio between HRmax and HRrest - the Heart Rate Ratio Method. Eur J Appl Physiol. 91, 111-115.

2. Harms, F., Bodmer, S., et al. (2015). Cutaneous mitochondrial respirometry: non-invasive monitoring of mitochondrial function. J Clin Monit Comput. 29, 509-519.

3. Koutlianos, N., Dimitros, E., et al. (2013). Indirect estimation of VO2max in athletes by ACSM´s equation: valid or not?. Hippokratia. 17 (2), 136-140.

### A176 Feasibility of real-time prescriptive analytics to make predictions and suggest decision options for the prevention of hypotension during ICU stay

#### C. Lee^1,2^, F. Hatib^1^, M. Cannesson^3^

##### ^1^Edwards Lifesciences, Critical Care, Irvine, United States; ^2^University of California Irvine, Biomedical Engineering, Irvine, United States; ^3^University of California Los Angeles, Anesthesiology, Los Angeles, United States

###### **Correspondence:** C. Lee – Edwards Lifesciences, Critical Care, Irvine, United States

**Background** Patients in critical care settings are often at risk of developing hypotension, which can lead to poor outcomes. To address this need for early detection of hypotensive events, we have developed a hypotension probability indicator (HPI™). A hypotensive event was defined by any time period where MAP < 65 mmHg. After training the HPI™ model on 3,000 ICU and surgical patients, we tested it on an independent data set and demonstrated sensitivity and specificity > 80 % for detection of an event respectively at 5, 10, and 15 minutes prior to its start. To give further clinical value to the HPI™ as a decision support index, there is a need for understanding the underlying reasons for a patient trending towards hypotension. In this study, we evaluate the use of stroke volume (SV), cardiac output (CO), stroke volume variation (SVV), dynamic elastance (Ea dyn), systemic vascular resistances (SVR), and dp/dt to classify patients into 4 prescriptive hypotensive groups: 1) Decreased preload, 2) Decreased afterload, 3) Decreased contractility, and 4) Uncertain.

**Methods** Data used in this study came from the MIMIC II MIT database (n = 326). Arterial pressure waveforms from these patients were processed through FloTrac (Edwards Lifesciences) for calculation of mean arterial pressure (MAP), CO, SV, SVV, Ea dyn, dp/dt, and SVR. All data was annotated for events as defined previously. Events were then classified into 4 groups based on % change from 15 to 0 minutes prior to event: 1) Decreased preload (decrease in SV, CO, and increase in SVV); 2) Decreased afterload (decrease in Ea and SVR); 3) Decreased contractility (decrease in dP/dt); and 4) Uncertain (did not meet any criteria). Any events that met more than 1 group criteria were not used in the analysis to avoid any overlap. After classification of events, % changes of SV, CO, SVV, Ea dyn, SVR, and dP/dt from 15 to 10 minutes prior to event and 15 to 5 were evaluated to assess if an event's underlying cause could be identified early on. A t-test was used to assess significant difference (p < 0.05) in mean % changes by group.

**Fig. 75 (abstract A176).**
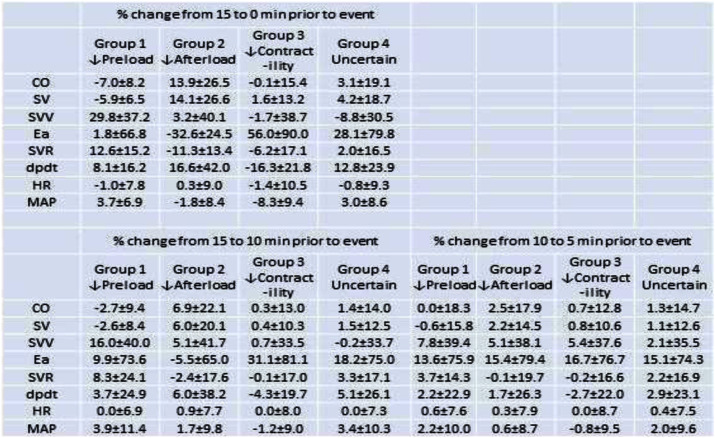
% change values (mean ± standard deviation) from 15 to 0 minutes prior to event (used in classification of groups); 15 to 10 min; and 10 to 5 min

**Results / conclusions** There were 25,419 total hypotensive events. Group 1 contained 1,200 events, 2 had 2,066, 3 had 7,290, and 4 had 5,283. 9,560 events were not used in analysis due to data outliers or meeting more than 1 group criteria. Overall, each group's 5 minute % change profile was different at 5 and 10 minutes (Fig. 75). % change in CO, SVV, and SV were significantly different when comparing Group 1 to 2, 3, and 4 at 10 minutes. % change in Ea dyn and SVR were significantly different when comparing Group 2 to 1, 3, and 4 at 10 minutes prior to event. % change in dP/dt were all significantly different when comparing Group 3 to 1, 2, and 4 at 10 and 5 minutes prior to event. In conclusion, the underlying cause of a hypotensive event can potentially be classified into 1 of 4 prescriptive groups up to 10 minutes prior to the start of an event.

### A177 Diastolic blood pressure, static arterial elastance, dynamic arterial elastance and arterial resistance during fluid challenge in septic shock: a pilot study

#### P. Theerawit, T. Morasert, Y. Sutherasan

##### Ramathibodi Hospital, Mahidol University, Medicine, Bangkok, Thailand

###### **Correspondence:** P. Theerawit – Ramathibodi Hospital, Mahidol University, Medicine, Bangkok, Thailand

**Introduction** The dynamic arterial elastance (EaDyn) is used to predict the rising of mean arterial pressure (MAP) from fluid challenge in fluid responders. However, according to ventricular-arterial coupling, arterial resistance (Rart), not arterial elastance should be a predictor for MAP responsiveness

**Objectives** Our study aimed to understand the relationship between arterial resistance variables and arterial elastance variables, and analyzed their performances to predict MAP responders in septic shock patients received fluid challenge (FC).

**Methods** The Rart was MAP divided by cardiac output. The EaStat was pulse pressure (PP) divided by stroke volume (SV) and the EaDyn was pulse pressure variation (PPV) divided by stroke volume variation (SVV). We obtained these parameters at baseline and at the end of fluid challenge (FC). We performed FC with 1,000 ml of normal saline solution infused in 30 minutes. The relationship among variables was determined by correlation coefficient. The performance was determined by the area under the ROC curve.

**Results** A total of 28 septic shock subjects were included consecutively. The mean APACHE II, SOFA and lactate were 30.93 ± 7.34, 10.93 ± 4.10 and 6.71 ± 5.07 respectively. All subjects received fluid therapy prior to enrollment. The mean MAP at baseline was 61.36 ± 8.27 mmHg. The mean Rart at baseline was 14.48 ± 6.21 mmHg.min/L. The mean DBP, EaStat and EaDyn at baseline were 46.9 ± 38.31 mmHg, 1.03 ± 0.52 mmHg/L and 1.22 ± 0.78 respectively. The mean Rart at baseline was similar to the mean Rart at the end of FC (14.48 ± 6.21 mmHg.min/L vs. 13.96 ± 6.34 mmHg.min/L; p = 0.259). At baseline, the diastolic blood pressure (DBP) and EaStat correlated to the Rart (r = 0.428;p = 0.023 vs. r = 0.551;p = 0.002). At end of FC, the DBP and EaStat correlated to the Rart (r = 0.55;p = 0.002 vs. r = 0.69;p < 0.0001). No correlation was observed between the EaDyn and Rart. Only DBP at baseline showed significant difference between MAP responders and non-responders (41.00 ± 6.43 mmHg vs. 50.76 ± 7.13 mmHg; p = 0.001). The area under the ROC curve of DBP at baseline to predict MAP responsiveness were 0.845 (95%CI 0.699-0.991). The value of DBP at baseline < 42 mmHg predicted MAP responders with 63.6 % sensitivity and 94.1 % specificity. Fluid responders had the mean DBP at baseline significantly lower than the non-responders (43.60 ± 7.35 mmHg vs. 50.77 ± 7.91 mmHg; p = 0.02), whereas the Rart, EaStat and EaDyn were similar in both groups.

**Conclusions** The Rart remained stable throughout period of FC. Even though we found the correlation between DBP, EaStat and Rart, but the meaning of DBP, arterial elastance and arterial resistance are not identical. In a group of septic shock with very low DBP, the DBP, not arterial resistance and elastance variables, correlated to increasing of MAP and CO after FC. Thus we may use it as a predictor of MAP responders instead of the EaDyn.

### A178 Effectiveness and safety of arterial catheterization with BBraun Introcan Safety 3

#### G. Zani, S. Mescolini, M. Diamanti, R. Righetti, A. Scaramuzza, M. Papetti, M. Terenzoni, C. Gecele, M. Fusari

##### Santa Maria delle Croci Hospital, Anesthesia and Intensive Care, Ravenna, Italy

###### **Correspondence:** G. Zani – Santa Maria delle Croci Hospital, Anesthesia and Intensive Care, Ravenna, Italy

**Introduction** In high-risk surgical patients arterial catheterization is needed to monitoring blood pressure and perform blood gas analysis, both in the operating room and ICU. The Seldinger technique is considered the gold standard, but many catheters are recently proposed to be as effective and safe.

**Objectives** Evaluate effectiveness and safety of BBraun Introcan Safety 3 for arterial catheterization in adult high-risk surgical patients scheduled to postoperative ICU stay, compared to Seldinger devices.

**Methods** BBraun Introcan Safety 3, Vygon Leader-Cath and Arrow Arterial Catheterization Set were analysed for arterial cannulation in adult high-risk surgical patients scheduled to postoperative ICU stay, for the follow parameters: adequate size, easy recognition of the vascular space, easy Introduction, traumaticity, cannula fixation, efficacy in blood pressure monitoring and taking blood samples for the first 96 hours, safety. The catheter was placed in the operating room before induction of general anesthesia and maintained during ICU stay. A questionnaire was administrated to the physician after arterial cannulation and each parameter was score as poor (2 points), middling (4 points), sufficient (6 points), good (8 points) or excellent (10 points); the maximum score was 70/70 points. Failures and complications were recorded. Appropriate statistical analysis was performed to compare effectiveness and safety of the three devices and p < 0.05 was considered to be significant. The non-inferiority margin was defined as 5 %.

**Results** 100 arterial cannulations for each device were performed by 10 medical doctors with a mean experience in arterial catheterization of 8 years. Seldinger kits obtained the best score (56/70 points), but Introcan Safety 3 resulted to be non-inferior (54/70 points), with a 4 % of failure rate. No statistical differences were founded for adequate size, easy recognition of the vascular space, easy Introduction, traumaticity, cannula fixation, efficacy in blood pressure monitoring and taking blood samples during the first 96 hours. Introcan Safety 3 emerged to be the safer device, due to its anti-reflux and anti-puncture systems (p < 0.05). No severe complications were reported during and after procedures.

**Conclusions** BBraun Introcan Safety 3 resulted a safe and effective device to perform arterial catheterization in adult high-risk surgical patients scheduled to postoperative ICU stay.

**References**

1. Gershengorn HB, et al: Variation of arterial and central venous catheter use in United States intensive care units. Anesthesiology 2014, 120:650-64.

### A179 Cardiac output monitoring: comparison between transthoracic echocardiographic measurements and transpulmonary thermodilution

#### K.A. Hakim, A. Chaari, M. Ismail, A.H. Elsaka, T.M. Mahmoud, K. Bousselmi, V. Kauts, W.F. Casey

##### King Hamad University Hospital, Intensive Care, Muharraq, Bahrain

###### **Correspondence:** K.A. Hakim – King Hamad University Hospital, Intensive Care, Muharraq, Bahrain

**Introduction** The transpulmonary thermodilution (TPTD) technique of cardiac output monitoring which applies the Stewart Hamilton principle is well established in terms of cardiac output accuracy.(1) However, it is an invasive procedure that involves various risks.(2).

Researchers are continuously exploring potential less invasive alternatives. Transthoracic echocardiographic left ventricular outflow tract (LVOT) measurements is a recognized tool for assessing the cardiac output noninvasively. However, there is paucity of definitive data concerning its accuracy.

**Objectives** We aimed to validate the noninvasive transthoracic echocardiographic estimates of the stroke volume against the stroke volume measurements obtained invasively by the TPTD technique.

**Methods** Twenty successive critically ill patients in whom a PiCCO™ cardiac output monitor (9 female; 11 male; mean (SD) age 66 (12.9) years) were the subject of this study. We compared 20 pairs of stroke volume (SV) readings obtained simultaneously from the TPTD component of the PiCCO™ cardiac output monitor and from transthoracic echocardiography (LVOT diameter and velocity time integral).

**Results** The averaged values of SV measurements from Echocardiography compared to the TPTD were 61 (20) vs. 67 (28) ml. The SV measurements from Echocardiography and TPTD showed a significant correlation (p = 0.015), the mean bias was 6.1 ml and the 95 % limits of agreement (mean difference ± 1.96 SD) were 47.04 to -34.79 ml.

**Conclusions** Monitoring of the cardiac output noninvasively using transthoracic echocardiography is a reproducible feasible option.

**References**

1. Reuter DA, Huang C, Edrich T, Shernan SK, Eltzschig HK. Cardiac output monitoring using indicator-dilution techniques: basics, limits, and perspectives. Anesthesia and analgesia. 2010;110(3):799-811.

2. Kornbau C, Lee KC, Hughes GD, Firstenberg MS. Central line complications. International journal of critical illness and injury science. 2015;5(3):170-8.

**Fig. 76 (abstract A179).**
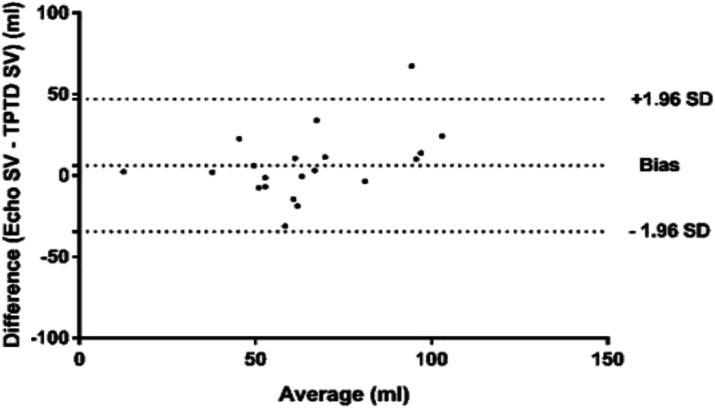
Bland-Altman plot of the differences between Echocardiography & TIPD SV against their averages

**Fig. 77 (abstract A179).**
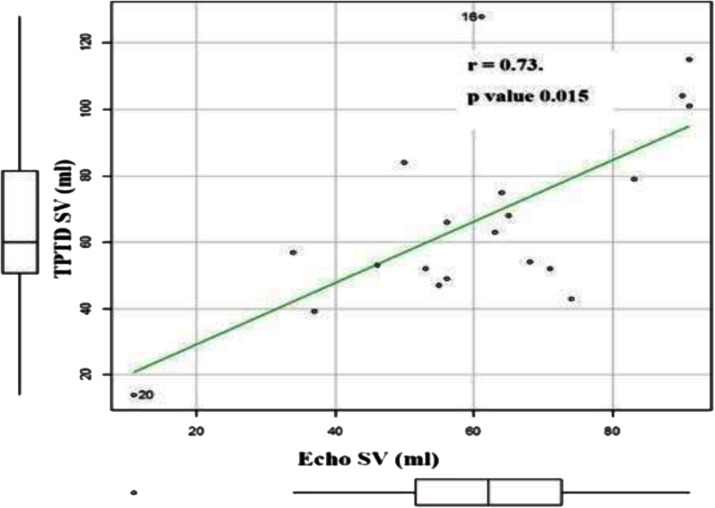
Linear regression of pollsed data for Echocardiography SV against TPTD SV

### A180 Early microcirculatory dysfunction predicts poorer outcomes in a porcine model of complex traumatic haemorrhagic shock and resuscitation and is a potential therapeutic target

#### S.D. Hutchings^1,2^, D. Naumann^3^, J. Wendon^1^, S. Watts^2^, E. Kirkman^2^

##### ^1^Kings College Hospital, London, United Kingdom; ^2^Defence Science and Technology Laboratory, Salisbury, United Kingdom; ^3^NIHR Surgical Reconstruction and Microbiology Research Centre, Birmingham, United Kingdom

###### **Correspondence:** S.D. Hutchings – Kings College Hospital, London, United Kingdom

**Background** Traumatic Hemorrhagic Shock (THS) is a leading cause of preventable death following severe traumatic injury. The microcirculation is the ultimate structure concerned with tissue perfusion, and is therefore of primary importance during THS. The microcirculation was examined in a large animal model of THS, in order to investigate the effects of microcirculatory dysfunction during resuscitation.

**Methods** Baseline standard microcirculatory parameters were obtained for 22 large white pigs using sublingual Incident Dark Field (IDF) video-microscopy. All animals were subjected to a standardized hind-limb injury followed by a controlled haemorrhage of approximately 35 % of blood volume (shock phase). This was followed by 60 min of fluid resuscitation with either 0.9 % saline or component blood products and a target SBP of 80 mmHg (early resuscitation phase). All animals were then given blood products to a target SBP of 110 mmHg for 120 min (mid resuscitation phase), and a further 100 min (late resuscitation phase). IDF readings were obtained at the mid point of each of these phases. Cardiac output was measured using a pulmonary artery catheter. Animals were divided into above average (A) and below average (B) perfused vessel density (PVD) groups based on the lowest recorded PVD measurement taken during the shock and early resuscitation phases.

**Fig. 78:**
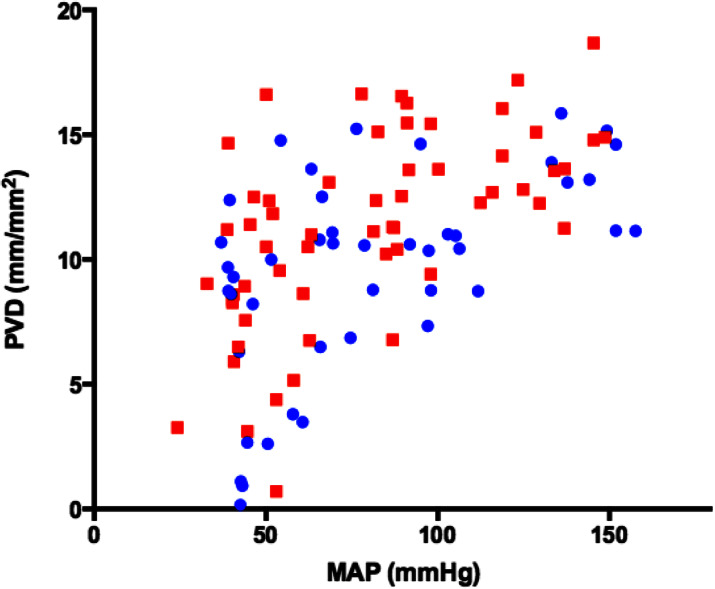
(abstract A180).

**Fig. 79:**
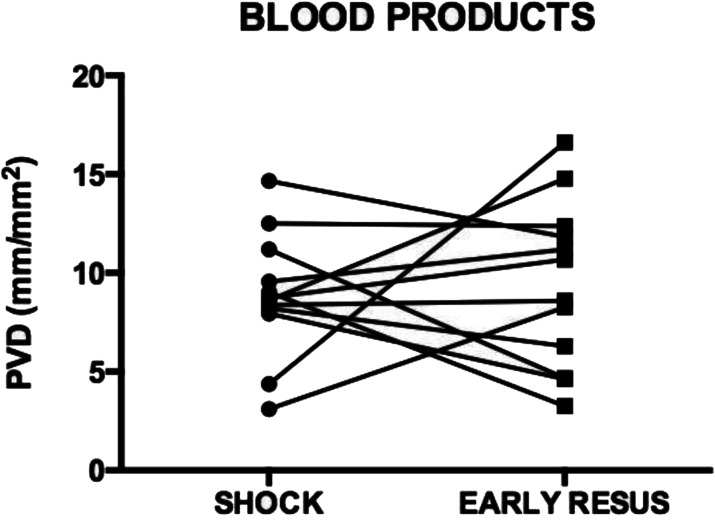
(abstract A180).

**Fig. 80:**
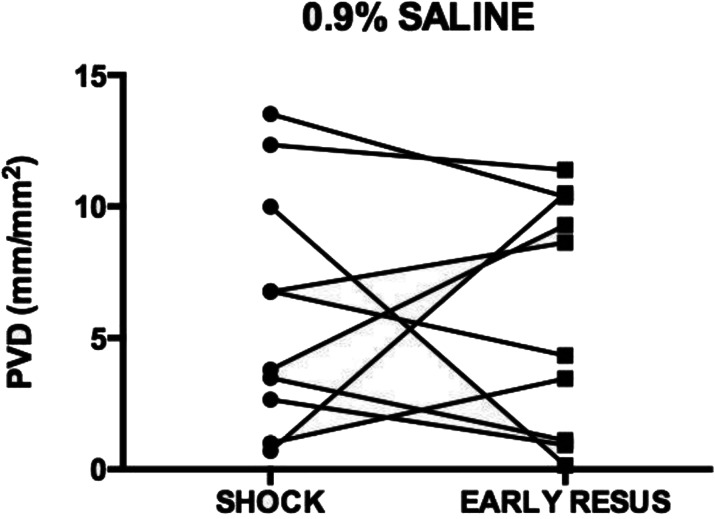
(abstract A180).

**Results** During shock and early resuscitation Group A (n = 10) had a mean PVD of 10.5 (SD ± 2.5) mm/mm^2^, and Group B (n = 12) 5.5 (SD ± 4.1) mm/mm^2^. During the later resuscitation phases, Group A maintained a significantly higher PVD than Group B. Group A initially had a higher cardiac output but the difference between the groups narrowed as resuscitation progressed. At the end of resuscitation group A had significantly lower plasma lactate, higher lactate clearance, lower standard base deficit, and smaller mixed venous - arterial CO_2_ gradient. There was no significant difference in blood pressure between the two groups at any stage. There was a wide spread of PVD for a given blood pressure, especially during the shock and early (hypotensive) resuscitation phases (Fig. 78). The choice of initial resuscitation fluid appeared not to produce differing effects in terms of microcirculatory perfusion (Figs. 79 and 80).

**Note:** This abstract has been previously published and is available at [1]. It is included here as a complete record of the abstracts from the conference.

**References**

1. Hutchings SD, Naumaan DN, Watts S, Wilson C, Burton C, Wendon J, Kirkman E (2016) Microcirculatory perfusion shows wide inter-individual variation and is important in determining shock reversal during resuscitation in a porcine experimental model of complex traumatic hemorrhagic shock. Intensive Care Medicine Experimental 4:17.

**Conclusions** Early changes in microvascular perfusion are key determinants in subsequent tissue perfusion following fluid resuscitation, and appear unrelated to pressure based parameters. Choice of initial resuscitation fluid appears to have little impact on microcirculatory perfusion during resuscitation from traumatic haemorrhagic shock. Microcirculatory parameters may be more reliable markers of physiological insult than global haemodynamic parameters, and are potential targets for goal-directed resuscitation.

### A181 Predicting hypotension in intensive care unit patients

#### Z. Jian^1^, S. Buddi^1^, C. Lee^1^, J. Settels^2^, F. Hatib^1^, M.R. Pinsky^3^

##### ^1^Edwards Lifesciences, Irvine, United States; ^2^Edwards Lifesciences, Amsterdam, Netherlands; ^3^University of Pittsburgh, Department of Critical Care Medicine, Pittsburgh, United States

###### **Correspondence:** Z. Jian – Edwards Lifesciences, Irvine, United States

**Introduction** Patients in critical care settings are often at risk of developing hypotension, which can increase the risk of complications. Early hypotension typically manifests as subtle changes of physiological signals that can go unnoticed at first. We used machine learning (ML) techniques to develop a predictive algorithm for hypotensive episodes, by exploiting arterial pressure waveform characteristics.

**Objectives** To test the accuracy of the hypotension probability algorithm on ICU patients.

**Methods** ML techniques based on multivariate logistic regression analysis were used to construct a mathematical model for predicting hypotension expressed as a hypotension probability indicator (HPI™). A hypotensive episode was defined as MAP < 65 mmHg for at least 1 minute. Multiple hemodynamic features were extracted from the arterial pressure waveform and used as predictor variables in the model. The model was trained and cross-validated with data from a 3,000 patient clinical database.

478 ICU patients from multiple clinical sites that were not used in the calibration set were used as a test set for the final validation of HPI™. Patients included septic shock (189), post cardiac surgery (208), cardiogenic shock (32), post liver transplantation (19), and others (30, respiratory failure, post non cardiac surgery, etc.). The patient demographics are listed in Table 58. Radial arterial pressure waveforms were recorded with a FloTrac™ sensor (Edwards Lifesciences, Irvine, CA). The waveforms were passed to the algorithm to calculate the hypotension probability. The receiver operating characteristic (ROC) analysis was used to assess algorithm performance.

**Fig. 81:**
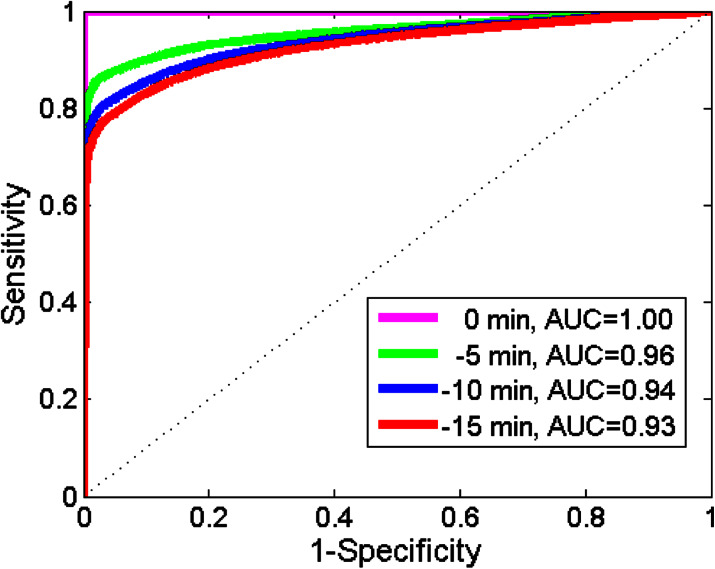
(abstract A181).

**Results** 16,078 hypotensive events were registered, on average 34 events per patient with a duration of 11 (±89) minutes per event. The algorithm was able to predict hypotension with a sensitivity and specificity of 90 %, 87 % and 86 %, for 5, 10, and 15 minutes prior to the event, respectively. The area under the curve (AUC) is 0.96, 0.94, and 0.93, for 5, 10, and 15 minutes prior to the event, respectively (Fig. 81).

**Conclusions** These data suggest that HPI™ is capable of predicting hypotensive events with high sensitivity and specificity in ICU patients, up to 15 minutes prior to event.

**Table 59:** (abstract A181).

Age, years, mean (SD)	62 (15)
Weight, kg, mean (SD)	81 (24)
Height, cm, mean (SD)	170 (11)
Gender	478 (170 F, 308 M)
Duration of ICU time, hours, mean (10th, 90th percentile)	108 (26, 219)

### A182 Predicting hemodynamic derangement in septic shock: a machine learning approach

#### P. Bertini, F. Guarracino

##### University Hospital of Pisa, Department of Anaesthesia and Critical Care Medicine, Cardiothoracic and Vascular Anaesthesia, Pisa, Italy

###### **Correspondence:** P. Bertini – University Hospital of Pisa, Department of Anaesthesia and Critical Care Medicine, Cardiothoracic and Vascular Anaesthesia, Pisa, Italy

**Introduction** Machine learning is an emerging technique that enables computers to learn from data without been explicitly programmed [1], in the medical field has been used for classification and prediction analysis in both supervised and supervised fashion [2]. Cardiomechanics determinants end-systolic and arterial elastances and ventricular arterial coupling (VAC) in the critical care patient treated for hemodynamic derangement has been recently investigated [3].

**Objectives** In this study we aimed to identify the possibility of a machine learning approach to classify hemodynamic data in the intensive care unit.

**Methods** We used three non linear supervised machine learning approaches (multilayer perceptron neural network, decision tree and gaussian support vector machine) to classify responders vs non responders from a dataset of 115 patients using Matlab R2015b software. The classification models were run three times with respect to independent variables: mean arterial pressure (MAP), arterial elastance (Ea), VAC.

**Results** Best performance of the classification models was carried out by decision trees, in all runs.

With accuracy of 86.7 % with respect to MAP increase, 96.8 % with respect to VAC decrease and 80 % with respect to Ea increase.

**Conclusions** In this analysis we demonstrated the feasibility of a machine learning approach to hemodynamic data analysis in the intensive care patient. Decision tree was found to be the most effective technique in analysing hemodynamic data. The models built allowed prediction for responders vs non responders with respect to independent variable VAC decrement with an accuracy above 96 %.

**Reference(s)**

1. Simon P: Too Big to Ignore: The Business Case for Big Data: Wiley; 2013.

2. Chen L, Dubrawski A, Wang D, Fiterau M, Guillame-Bert M, Bose E, Kaynar AM, Wallace DJ, Guttendorf J, Clermont G *et al*: Using Supervised Machine Learning to Classify Real Alerts and Artifact in Online Multisignal Vital Sign Monitoring Data. *Critical care medicine* 2016.

3. Guarracino F, Ferro B, Morelli A, Bertini P, Baldassarri R, Pinsky MR: Ventriculoarterial decoupling in human septic shock. *Critical care* 2014, 18(2):R80.

### A183 A novel approach for evaluation of preload independent left ventricular contractile cardiac function by means of thermodilution and pulse contour analysis in an experimental animal model

#### C. Trepte^1^, P. Richter^2^, S.A. Haas^1^, V. Eichhorn^3^, J.C. Kubitz^1^, D.A. Reuter^1^

##### ^1^University Medical Center Hamburg-Eppendorf, Hamburg, Germany; ^2^Klinikum Harlaching, München, Germany; ^3^Dalhousie University, Halifax, Canada

###### **Correspondence:** C. Trepte – University Medical Center Hamburg-Eppendorf, Hamburg, Germany

**Introduction** Evaluating and monitoring contractile cardiac function is a key element in hemodynamic management of critically ill patients. However, evaluation of intrinsic contractile cardiac function is difficult in a clinical setting.

**Objectives** Aim of the study was to evaluate a novel approach of assessing load-independent left-ventricular contractility based on pulse contour analysis (rate of aortic maximum pressure rise (dP/dt_Ao_)) and estimation of end-diastolic volume (VED) by transcardiopulmonary thermodilution (TCPTD) in an experimental animal model in pigs.

**Methods** 16 domestic pigs were studied. dP/dt_Ao_ as evaluated by pulse contour analysis was related to VED by TCPTD. Direct measurement of rate of maximum pressure rise in left ventricle (dP/dt_LV_) related to VED (cdP/dt_LV_) served as experimental reference of preload independent contractility. [1] Measurements were carried out in normal cardiac function and experimentally impaired cardiac function (continuous infusion of verapamil) during a wide modification of cardiac preload (withdrawal of blood 20 ml kg ^-1^ bodyweight).

**Results** While impairment of contractile cardiac function by continuous infusion of verapamil resulted in significant changes of cdP/dt_Ao_ and cdP/dt_LV_ (p < 0.05), neither in normal as well as in impaired cardiac function did cdP/dt_Ao_ and cdP/dt_LV_ present significant changes during preload modifications (p > 0.05).

**Conclusions** Estimation of cdP/dt_Ao_ by means of pulse contour analysis and thermodilution provides reliable assessment of preload-independent left ventricular contractility and its changes in an experimental animal model.

**Reference(s)**

1. Kass DA, Maughan WL, Guo ZM, Kono A, Sunagawa K, Sagawa K, (1987) Comparative influence of load versus inotropic states on indexes of ventricular contractility: experimental and theoretical analysis based on pressure-volume relationships. Circulation 76: 1422-1436

**Grant acknowledgement**

The study was supported by departmental funds

### A184 Transesophageal Doppler corrected systolic flow time versus central venous pressure as a predictor for fluid responsiveness in septic shock patients

#### M.S. Soliman^1^, W.I. Hamimy^2^, A.Z. Fouad^2^, A.M. Mukhtar^2^

##### ^1^Cairo University, Anaesthesia, Cairo, Egypt; ^2^Cairo University, Cairo, Egypt

###### **Correspondence:** M.S. Soliman – Cairo University, Anaesthesia, Cairo, Egypt

**Background** Aortic corrected flow time (FTc) is easily measured by Doppler techniques. Recent data using transoesophageal Doppler suggest that it may predict fluid responsiveness in critical care. This use of FTc has not previously been evaluated in septic shock, only one preliminary study have incorporated transcutaneously measured FTc. Denoting its importance in prediction of fluid responsiveness in septic patient Furthermore, no comparison has been made between transesopahgeal FTc and central venous pressure(CVP).

**Objective** The aim of our study was to compare the impact of using FTc versus CVP as a guide for fluid resuscitation in septic shock on stroke volume denoting cardiac responsiveness for fluid administration.

**Methods** This was a prospective study of 46 consecutive adult septic shock patients (in sinus rhythm) 44 patients were mechanically ventilated, treated with intravenous fluid challenge (500 ml over 15 minutes) guided with CVP in control group and guided by FTC in Doppler group assessment incorporating transesophageal aortic Doppler (CardioQ®) measurements in a surgical tertiary intensive care unit. Stroke volume (SV), mechanical ventilation days, length of stay and mortality of both groups were recorded

**Results** Percent change in stroke volume strongly correlated with baseline FTc (r = -0.6831, P = 0.000) but not central venous pressure (r = -0.0864, P = 0.56). Baseline FTc < 332 ms discriminated responders from non-responders [AUC = 0.989, 95 % confidence interval = 0.954 to 1.023; P = 0.01)]. Regarding survival, there were no statistically significant differences between both CVP& FTC groups.

**Conclusion** Transesophageal aortic Doppler is a simple, non-invasive tool of guiding fluid therapy in patients with severe sepsis and septic shock. FTC change was a better predictor of fluid responsiveness than CVP in septic shock.

**Reference(s)**

1. Girbes ARJ, Groeneveld ABJ. Circulatory optimization of the patient with or at risk for shock. Clin Intensive Care 2000; 11: 77-88.

2. Moore FA, McKinley BA, Moore EE. The next generation in shock resuscitation. Lancet 2004; 363: 1988-1996

3. Tuchschmidt J and Sharma O: Impact of hemodynamic monitoring in a medical intensive care unit. Grit Care Med; 1987;15: 840-843.

4. Dalen JE and Bone RC: Is it time to pull the pulmonary artery catheter? JAMA; 1996; 276: 916-918.

5. Polanczyk CA, Rohde LE, Goldman L. et al.: Right heart catheterization and cardiac complications in patients undergoing non-cardiac surgery: an observational study. JAMA; 2001; 286(3):309-314.

6. Groeneveld AB. Vascular pharmacology of acute lung injury and acute respiratory distress syndrome. Vascul Pharmacol 2002; 39:247-256.

7. Groeneveld AB, Polderman KH. Acute lung injury, over hydration or both? Crit Care 2005; 9: 136-137.

8. Sakr Y, Vincent JL, Reinhart K, et al. On behalf of the “Sepsis Occurrence in Acutely Ill Patient” investigators : High tidal volume and positive fluid balance are associated with worse outcome in acute lung injury. Chest 2005.

9. Linde-Zwirble WT, Angus DC: Severe sepsis epidemiology: Sampling, selection, and society. Crit Care 2004; 8: 222-226

10. Dellinger RP, Levy MM, Carlet JM, et al.: Surviving Sepsis Campaign: International guidelines for management of severe sepsis and septic shock: 2013. Crit Care Med 2008; 36: 296 -327

11. Sturgess DJ, Pascoe RLS, Scalia G, Venkatesh B. A comparison of trans-cutaneous Doppler corrected flow time, b-type natriuretic peptide and central venous pressure as predictors of fluid responsiveness in septic shock: a preliminary evaluation. Anaesth Intensive Care. 2010;38:336-341.

### A185 Infrared thermal imaging of the hand during vascular occlusion in healthy volunteers

#### M. Charlton^1^, L. Tonks^2^, L. Mclelland^3^, T.J. Coats^3^, J.P. Thompson^1^, M.R. Sims^2^

##### ^1^University Hospitals of Leicester NHS Trust, Anaesthesia, Critical Care and Pain, Leicester, United Kingdom; ^2^University of Leicester, Leicester, United Kingdom; ^3^University Hospitals of Leicester NHS Trust, Emergency Medicine Academic Group, Leicester, United Kingdom

###### **Correspondence:** M. Charlton – University Hospitals of Leicester NHS Trust, Anaesthesia, Critical Care and Pain, Leicester, United Kingdom

**Introduction** Vascular occlusion tests (VOT) have been utilized alongside near infrared spectroscopy as a means of dynamically investigating microcirculatory function (1), with correlation demonstrated between derangement in muscle tissue oxygenation and mortality (2). Human skin has an exquisite microcirculatory blood supply which can be visualized by means of infrared thermography. Thermography may therefore be a potentially useful non-contact, real-time monitor of microcirculatory function.

**Objectives** To investigate the thermal infrared profile of the palm during a VOT in healthy volunteers.

**Methods** Participants were recruited from faculty and students at the University of Leicester. Room temperature was confirmed between 19-21oC throughout data acquisition and participants were allowed a period of acclimatization prior to measurement. Baseline blood pressure was measured in the left forearm. The right forearm was then placed on a bench with a blood pressure cuff placed around it. A FLIR T650sc thermal imaging camera was placed at a distance of 1 meter from the participant. Thermal video recording was commenced and the blood pressure cuff was inflated to 50 mmHg above the previously measured systolic pressure for a total of 3 minutes. Recording continued for 5 minutes following deflation of the cuff. Average palm temperatures were measured using a 100 x 100 pixel sample. Data were extracted using FLIR Tools + software and analyzed using Microsoft Excel and R-Studio.

**Fig. 82 (abstract A185).**
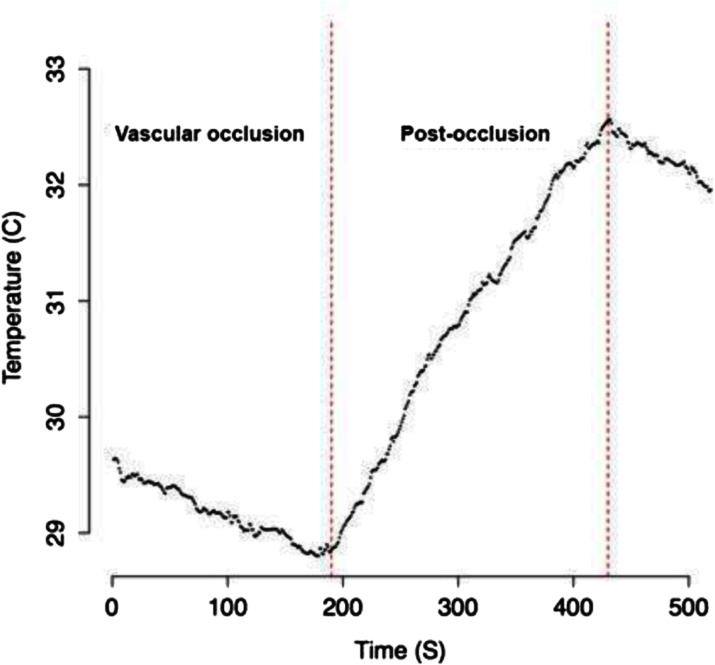
Palm temperature during VOT

**Results** Data were collected from 34 healthy volunteers (19 male), mean age 23 years [range 20-49] on 4 non-consecutive days. Two distinct stages of cooling and reheating were seen, with a rapid post-occlusion/reheating slope demonstrated at the end of the VOT in all volunteers [Fig. 82]. The mean rate of reheating in the palm was 0.015 °C.s^-1^ [SD 0.009 °C.s^-1^]. This is comparable to the mean gradient found when determining a linear model for the same stage, returning a result of 0.0148 °C.s^-1^ [SD 0.008 °C.s^-1^].

**Conclusion** From these results it can be concluded that the rate of reheating per second measured using infrared thermography following a vascular occlusion test in healthy volunteers is 0.015 ^+^/_-_ 0.009 °C.s^-1^. It is our hypothesis that this gradient will be prolonged in patients with sepsis. If demonstrated to be true, this technique could be used as a non-invasive diagnostic technique in sepsis.

**References**

(1) Mesquida J, Gruartmoner G, Espinal C. Skeletal muscle oxygen saturation (StO2) measured by near-infrared spectroscopy in the critically ill patients. Biomed Res Int 2013;2013:502194.

(2) Creteur J, Carollo T, Soldati G, Buchele G, De Backer D, Vincent JL. The prognostic value of muscle StO2 in septic patients. Intensive Care Med 2007 Sep;33(9):1549-1556.

### A186 Fice echo - is it really useful? Dr Dafydd Williams ICM St3 Ysbyty Glan Clwyd, Bodelwyddan, North Wales

#### D. Williams

##### Ysbyty Glan Clwyd, Critical Care, Bodelwyddan, United Kingdom

**Introduction** Focused Intensive Care Echo (FICE) is becoming an increasingly important tool in the intensivists' arsenal of haemodynamic assessment. It has the ability to diagnose structural and dynamic problems, is non-invasive, and inter-user variability is limited. Rigorous training and CPD is essential in order to prevent mismanagement based on incorrect findings. In the UK the Intensive Care Society administers FICE accreditation with this becoming compulsory for critical care trainees in future.

**Methods** Every patient who undergoes diagnostic FICE has the result documented in their notes and in an audit folder. This follows a standardised “sticker”. All results documented in this folder, ranging between December 2014 and January 2015, were collated and analysed in a spreadsheet. The aim was to assess how FICE impacts on care at a district general critical care unit.

**Results** 57 patients underwent documented FICE

37 % of these scans occurred during the weekend

Indications for scan: Haemodynamic Instability (5/57), Haemodynamic Instability - Post Operative (9/57), PE (1/57), Post Cardiac Arrest (8), Pulmonary Oedema (1), Respiratory Failure (11), Sepsis/SIRS (10), Not documented/Unclear (12)

Documented Views Obtained:

Parasternal Long Axis 42

Parasternal Short Axis 34

Apical 4 Chamber View 34

Subcostal View 31

FICE Questions

LV Significantly or Moderately Impaired 18

LV Dilated 5

RV Impaired/Dilated 8

Hypovolaemia 17

Pericardial Effusion (trace or above) 6

Evidence of Pleural Effusion 13

Additional Findings

Valve Problem 11 patients

Regional Wall Abnormality (4)

Fluid Status Improvement (1)

Other Finding (8) - including dilated bowel loops!

Significant Conclusions

14 patients had a problem of cardiac origin

3 patients were fluid overloaded/required restriction

2 found to have pericardial effusion (not drainable)

1 Respiratory Problem

20 patients had no significant abnormality/not documented

Changed management in 44 patients, Ventilation (1), Fluid Administration 17, Inotrope Addition/Change 5, Vasopressor Addition/Change 6, Fluid Restriction 1,Fluid Removal, (Diuretic/RRT) 2

Other Investigations 4

Contributed to decision about escalation/limitation of critical care 4

Formal ECHO documented as requested following FICE: 20/53

Consultant 31/57, Registrar 11, 2 FICE trainee (1 documented as reviewed by FICE approved Consultant)

**Conclusion** It is clear that FICE echo has had a significant impact on the management strategies of a significant number of patients, however the impact of this on morbidity/mortality is impossible to assess. It is also interesting to see that a disproportionate number of scans were performed during the weekend, when formal cardiac investigations aren't always as available as during the week. Documentation was generally good but some gaps in documentation were found and require improvement. We suggest that this data, although limited in number, supports funding of expanded FICE training and availability at district general critical care units.

### A187 Bedside assessment of preload in critically ill septic patients by echocardiography and electrical cardiometry

#### D.Z. Roushdy^1^, R.A. Soliman^1^, R.A. Nahas^2^, M.Y. Arafa^2^

##### ^1^Faculty of Medicine, Cairo University, Critical Care Department, Cairo, Egypt; ^2^Cairo University, Critical Care Department, Cairo, Egypt

###### **Correspondence:** D.Z. Roushdy – Faculty of Medicine, Cairo University, Critical Care Department, Cairo, Egypt

**Introduction** Right atrial pressure is considered a surrogate for right ventricular filling pressure or cardiac preload. Evaluation of the inferior vena cava is the most common technique for the estimation of RAP.(1)

Another alternative approach has been proposed using tissue Doppler imaging of the tricuspid valve.(2)

**Objectives** investigate the reliability of using the Tricuspid E/Ea ratio, IVC collapsibility index by bedside echocardiography and Stroke volume variation (SVV) by electrical cardiometry for the assessment of right atrial pressure in critically ill septic patients as an alternative to invasive CVP.

**Methods** Thirty patients with severe sepsis and hypotension (Mean arterial pressure i.e. MAP < 65 mmHg), were enrolled in our study. Fluid resuscitation (30 ml/kg) was administered. Fluid response was defined as MAP ≥ 65 mmHg. Preload assessment was done through CVP, IVC collapsibility and tricuspid E/Ea by tissue Doppler imaging (TDI). Stroke volume variation (SVV) measured by ICON® was used to assess fluid response.

**Results** The study included 13 males (43.3 %) with age 47.8 ± 19.7. Paired comparison showed significant change in MAP readings (P value < 0.001). Right ventricular filling pressures (CVP) were correlated to tricuspid E/Ea (R 0.608, P value < 0.001), and to IVC collapsibility index (R -0.495, P value 0.005). ROC curve showed cutoff 11.5 % for SVV to predict fluid responsiveness with Area under Curve (AUC) 0.927, sensitivity 100.0 %, and specificity 70.0 %.

**Conclusions** Tricuspid excursion using TDI offers non-invasive evaluation of right sided heart pressures and SVV could be used to predict fluid response in critically ill septic patients.

**Keywords:** right atrial pressure, tricuspid excursion, stroke volume variation, fluid response

**Reference(s)**

1. Kircher BJ, Himelman RB, Schiller NB. Noninvasive estimation of right atrial pressure from the inspiratory collapse of the inferior vena cava. Am J Cardiol. 1990;66(4):493-6. Epub 1990/08/15.

2. Arbo JE, Maslove DM, Beraud AS. Bedside assessment of right atrial pressure in critically ill septic patients using tissue Doppler ultrasonography. J Crit Care. 2013;28(6):1112 e1-5. Epub 2013/10/01.

## Acute myocardial infarction

### A188 Comparison of long-term mortality in patients with acute myocardial infarction associated with or without sepsis during admission

#### W.-T. Hung^1^, C.-C. Chiang^2^, W.-C. Huang^1^, K.-C. Lin^1^, S.-C. Lin^1^, C.-C. Cheng^2^, P.-L. Kang^2^, S.-R. Wann^1^, G.-Y. Mar^2^, C.-P. Liu^2^

##### ^1^Kaohsiung Veterans General Hospital, Department of Critical Care Medicine, Kaohsiung City, Taiwan, Province of China; ^2^Kaohsiung Veterans General Hospital, Cardiovascular Division, Kaohsiung City, Taiwan, Province of China

###### **Correspondence:** W.-T. Hung – Kaohsiung Veterans General Hospital, Department of Critical Care Medicine, Kaohsiung City, Taiwan, Province of China

**Introduction** Sepsis is a clinical syndrome characterized by a systemic inflammatory response to an infectious process. Patient with acute myocardial infarction (AMI) may be predisposed to develop to sepsis during admission.

**Objectives** The aim of this study was to evaluate the rates of sepsis during admission in patients with acute myocardial infarction (AMI) and their associated factors, and the long-term mortality in patients with AMI in associated with or without sepsis.

**Methods** The data from the National Healthcare Insurance Research Database (NHIRD) in Taiwan between January of 2000 and December of 2012 was used in this study. All patients who were first admitted to AMI were enrolled. Among the 186,112 identified cases hospitalized for AMI, 13,065 cases with an alternative diagnosis of sepsis (ICD: 038) were identified. Of the remaining 173,047 cases, patients with any diagnosis of infectious disease were excluded, leaving 146,737 AMI cases for comparison. For analysis, survival was defined as the end date of National Healthcare Insurance coverage.

**Results** The overall rate of sepsis during admission in patients with AMI was 8.18 %. The rates of sepsis in patients with AMI were 11.70 % in female and 6.63 % in male (P < 0.001). The rates of sepsis in patients with AMI were 3.19 % in percutaneous coronary intervention (PCI) group and 14.06 % in non-PCI group (P < 0.001). The rates of sepsis in patients with AMI were 3.79 % in below-65-year-old group and 11.32 % in equal-to-or-more-than-65-year-old group (P < 0.001). About the comorbidities, patients with AMI and sepsis have significant higher rates of hypertension, dyslipidemia, diabetes, peripheral vascular disease, congestive heart failure, end-stage renal disease, cerebral vascular accident and chronic obstructive pulmonary disease (all P < 0.05).

After 13 years of follow up, the survival rate of AMI was significant higher in patients without sepsis during admission (1.97 % vs. 0.19 %, adjusted HR 1.97; 95 % CI 1.93 to 2.01). Subgroup analysis revealed that both male and female patients with AMI have significant higher survival rates in non-sepsis groups (2.20 % vs. 0.26 % in male, P < 0.001; 1.40 % vs. 0.11 % in female, P < 0.001). Both patients with age below 65 and equal to or more than 65 years with AMI have significant higher survival rates in non-sepsis groups (3.23 % vs. 0.63 % in below 65 years, P < 0.001; 0.99 % vs. 0.09 % in equal to or more than 65 years, P < 0.001). Both PCI or non-PCI patients with AMI have significant higher survival rates in non-sepsis groups (2.17 % vs. 0.47 % in PCI group, P < 0.001; 1.70 % vs. 0.12 % in non-PCI group, P < 0.001).

**Conclusions** This study demonstrated that female gender, no PCI, and older age groups were associated with higher rates of sepsis during admission in patients with AMI. The long-term survival rates of AMI were significant higher in patients without sepsis during admission, including subgroup analysis of genders, ages, and PCI.

**Table 60 (abstract A188).** Characteristics in patients with/without sepsis-1

Variable	Sepsis		P-value
	No	Yes	
	n (%)	n (%)	
Gender			
Female	43063(29.35%)	5706(43.67%)	<.0001
Male	103674(70.65%)	7359(56.33%)	
Age (years)			
<65	64198(43.75%)	2532(19.38%)	<.0001
≧65	82539(56.25%)	10533(80.62%)

**Table 61 (abstract A188).** Characteristics in patients with/without sepsis-2

Variable	Sepsis		P-value
Comorbidities			
Hypertension	86523(58.96%)	7889(60.38%)	0.0016
Dyslipidemia	70758(48.22%)	3732(28.56%)	<.0001
Diabete Mellitus	56215(38.31%)	6070(46.46%)	<.0001
Peripheral vascular disease	5014(3.42%)	871(6.67%)	<.0001
Congestive heart failure	34000(23.17%)	4090(31.31%)	<.0001
End-stage renal disease	5854(3.99%)	974(7.46%)	<.0001
Cerebral vascular accident	30714(20.93%)	4899(37.5%)	<.0001
Chroinc obstructive pulmonary disease	15394(10.49%)	2651(20.29%)	<.0001

**Table 62 (abstract A188).** Characteristics in patients with/without sepsis-3

Variable	Sepsis		P-value
PCI			
Non-PCI	63040(42.96%)	10311(78.92%)	<.0001
PCI	83697(57.04%)	2754(21.08%)

**Fig. 83 (abstract A188).**
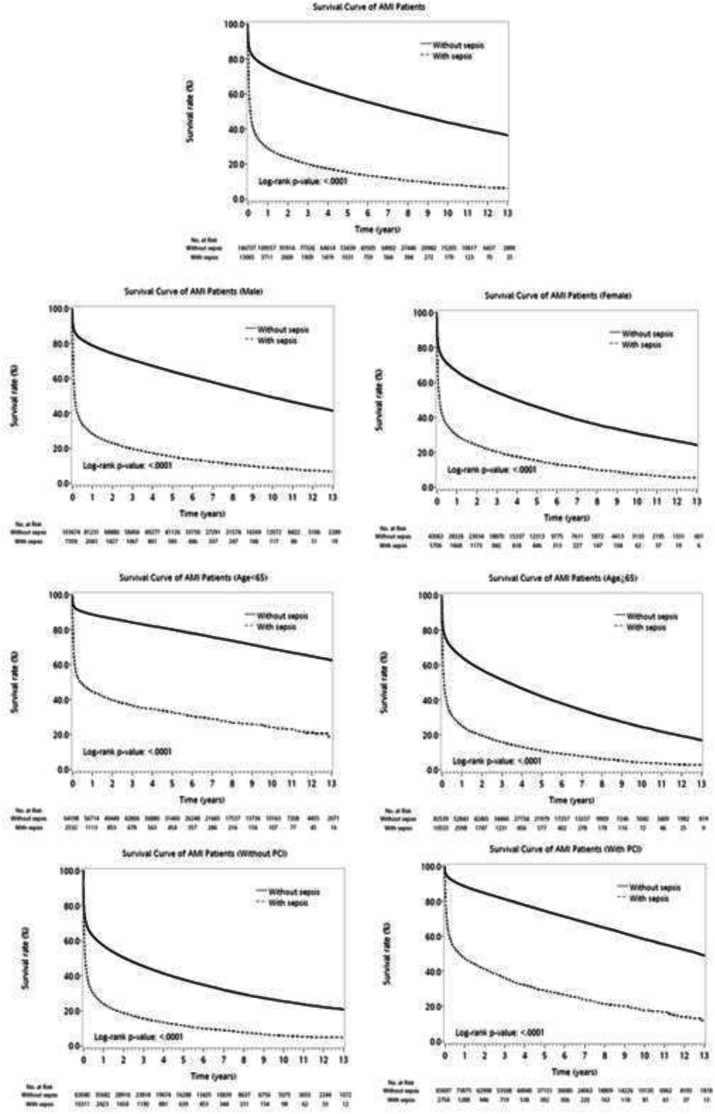
Kaplan-Meier survival curve after AMI

### A189 Cardiogenic Shock.ARIAM registry analysis ten years later

#### M. Lopez Carranza^1^, H. Sancho Fernandez^2^, J.A. Sanchez Roman^2^, F. Lucena^2^, A. Campanario Garcia^2^, A. Loza Vazquez^2^, A. Lesmes Serrano^2^, ARIAM-SEMICYUC Registry Investigators

##### ^1^Hospital Universitario V. de Valme, Medicina Intensiva, Sevilla, Spain; ^2^Hospital Universitario V. de Valme, Sevilla, Spain

###### **Correspondence:** M. Lopez Carranza – Hospital Universitario V. de Valme, Medicina Intensiva, Sevilla, Spain

**Introduction** Cardiogenic Shock (CS) is a severe complication of the Acute Myocardial Infarction (AMI). At the ESICM Congress 2004, we presented a study on the mortality of CS by gender in Spain, based on ARIAM Registry.

**Objectives** Evaluate the gender differences regarding the diagnosis, management and prognosis of patients with CS. A secondary objective was to compare these differences ten years later.

**Methods** Observational, descriptive and retrospective study. Patients included in the study are from ARIAM - SEMICYUC Registry (2012- 2015) and the ARIAM Registry (2000- 2004). All patients studied were diagnosed with CS. Variables: gender, age, pulmonary catheter and supportive measures (inotropics, mechanical ventilation, thrombolysis and Percutaneous Coronary Interventions(PCI) ). Statistical analysis: Chi- square.

**Results** In 2012-2015, there were 15,750 AMI patients with 847 (5.3 %) CS. 583 (68.83 %) men and 264 (31.17 %) women. PCI 628 (74.14 %). 372 (43.9 %) patients died in the ICU, of these 232 (39.79 %) were men and 140 (53.03 %) women. In 2000- 2004, there were 20,181 AMI patients and 555 (2.75 %) CS. PCI 126( 22.7 %). 249 (44.86 %) patients died in ICU, 160 (43.24 %) men and 89 (48 %) women.

**Conclusions** There are differences by gender in the management and outcome of the patients with CS. Supportive measures and PCI are used less frequently in women than in men. Mortality is higher in women. These latest results are very similar to those of ten years ago, even with the increase in the use of PCI.

**Grant acknowledgement**

ARIAM-SEMICYUC Registry Investigators.

**Table 63 (abstract A189).** General Data

2012-2015	Male Mortality* 232	Female Mortality* 140	p*	Male Total 583	Female Total 264	Mortality/Total
AP Catheter	25(53.2%)	6(50%)	0.0033	47(5.6%)	12(1.4%)	31/59 (52.5%)
Inotropics	208(42%)	104(50.5%)	0.001	495(58.4%)	206(24.3%)	312/701 (44.4%)
Mechanical Ventilation	142(45.9%)	64(59.3%)	0.004	309(36.5%)	108(12.8%)	206/417 (49.5%)
Thrombolysis	38(41.8%)	23(56.1%)	0.038	91(10.7%)	41(4.8%)	61/132 (46.2%)
PCI	145(32.3%)	76(42.5%)	0.001	449(53%)	179(21.1%)	221/628 (35.2%)

### A190 Is there a long term prognosis impact for different revascularization strategies in acute coronary syndromes complicated with acute heart failure at admission?

#### L. Sayagues Moreira^1^, R. Vidal-Perez^2^, U. Anido Herranz^1^, J.M. Garcia Acuna^2^, C. Pena Gil^2^, J.L. Garcia Allut^1^, P. Rascado Sedes^1^, C. Martin Lopez^1^, E. Saborido Paz^1^, C. Galban Rodriguez^1^, J.R. Gonzalez-Juanatey^2^

##### ^1^Complexo Hospitalario Universitario de Santiago de Compostela, Intensive Care, Santiago de Compostela, Spain; ^2^Complexo Hospitalario Universitario de Santiago de Compostela, Cardiology, Santiago de Compostela, Spain

###### **Correspondence:** L. Sayagues Moreira – Complexo Hospitalario Universitario de Santiago de Compostela, Intensive Care, Santiago de Compostela, Spain

**Introduction** Acute heart failure (AHF) has a strong negative prognostic impact in patients with non-ST-elevation acute coronary syndromes (ACS) and ST elevation ACS.

**Objectives** To establish the effect of coronary revascularization on the prognosis of ACS complicated with AHF at admission

**Methods** We retrospectively studied 5070 non selected patients admitted in a third level hospital from 2004 to 2009 followed up a mean of 5.4 ± 2.4 years. The effect of coronary revascularization on the outcome of patients with and without AHF was assessed using a multivariable regression model.

**Results** Acute heart failure was present in 765 patients (14.5 %) at hospital admission. During the index hospitalization 471 (61.5 %) patients received coronary revascularization by percutaneous coronary intervention (PCI) in 424 cases (55.4 %), and by coronary artery bypass grafting (CABG) in 47 patients (6.1 %). In a 5-year follow-up the mortality was 255 patients PCI group (49.7 %) , 27 (41.1 % ) in CABG group, and 289 (69.63 %) in the non revascularized group. AHF was an independent predictor of 5-year mortality (adjusted HR 4.3; 95 % CI 3.16-4.97; p < 0.001). Revascularization with PCI was protective in AHF population and non AHF population. Revascularization significantly influenced the prognosis of patients presenting AHF by CABG (adjusted HR 0.55; 95 % CI 0.28-1.09), but not of those without AHF (adjusted HR 0.51; 95 % CI 0.33-0.774; p = 0.001), data shown in figure.

**Conclusions** In patients with ACS, clinical manifestations of AHF at admission constitute a strong predictor of adverse outcome in the follow up that may be significantly modified by the coronary revascularization strategy

**Fig. 84 (abstract A190).**
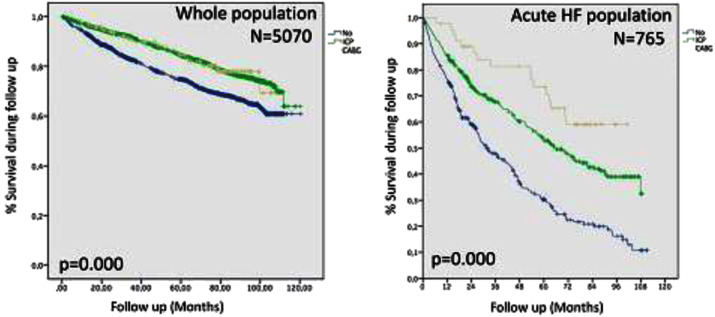
Prognosis impact of revascularization

### A191 Ten-year evolution of reperfusion strategies in patients with st-segment elevation myocardial infarction in a second level hospital and in Andalusia

#### A. Vallejo-Baez, M.V. de la Torre-Prados, P. Nuevo-Ortega, A. Fernández-Porcel, E. Cámara-Sola, T. Tsvetanova-Spasova, C. Rueda-Molina, L. Salido-Díaz, A. García-Alcántara, and ARIAM Group

##### ^1^University Hospital Virgen de la Victoria/IBIMA, Department of Intensive Care Unit, Málaga, Spain

###### **Correspondence:** P. Nuevo-Ortega – University Hospital Virgen de la Victoria/IBIMA, Department of Intensive Care Unit, Málaga, Spain

**Introduction** Early coronary reperfusion improves clinical course, prognosis and reduces mortality in patients with ST-segment elevation acute coronary syndrome

**Objectives** To analyze over a period of 10 years the evolution of reperfusion strategy and mortality in patients with ST-segment elevation myocardial infarction (STEMI), admitted in a 18 beds medical-surgical Intensive Care Unit (ICU), compared to other hospitals in the region (Andalusia).

**Methods** It is a prospective cohort study of patients with a diagnosis of STEMI admitted to a second level Universitary Hospital Virgen de la Victoria (UHVV) ICU, n = 2313 or, in other ICUs of Andalusia, n = 23167. The data were extracted from the Spanish record of Acute Myocardial Infarction Delay (ARIAM) between 01/01/2005 to 31/12/2014. The degree of coronary reperfusion, systemic fibrinolysis, primary and rescue percutaneous coronary intervention (PCI) and mortality were compared in three periods: 2005-2007, 2008-2010 and 2011-14. Descriptive and comparative statistical analysis was performed using the statistical software packages SPSS version 18.0 (SPSS Inc., Chicago, IL, USA)

**Results** We studied 2313 patients in UHVV group with a mean age of 61.8 ± 12.2 years old (61.3 ± 12.3 vs. 61.9 ± 12.5 vs. 62.2 ± 11.69, p= > 0.05) and 23 % were female (23 % vs. 22 % vs. 22 %; p= > 0.05). In Andalusia group (n = 23167) mean age was 62.3 ± 11.7 years old (62.5 ± 11.7 vs. 62.1 ± 11.8 vs. 61.9 ± 11.9; p= > 0.05) and 23 % were female (24 % vs. 23 % vs. 22 %; p= > 0.05). No significant differences between three periods in both groups The results we can see in the Table 64.

**Conclusions** During the studied period we found a higher percentage in the degree of coronary reperfusion in UHVV vs. Andalusia (84 % vs. 76 %), mainly by primary and rescue PCI (66 % vs. 48 %) and, greater decline in mortality in the first group than the regional benchmark (1.8 vs. 1.2).

**Reference(s)**

El Khoury C, et al. Five-year evolution of reperfusion strategies and early mortality in patients with ST-segment elevation myocardial infarction in France. Eur Heart J Acute Cardiovasc Care. 2015 Dec 17, 2015: 1-10

**Grant acknowledgement**

ARIAM spanish record.

**Table 64 (abstract A191).** Ten-year evolution of reperfusion strategies

Variables	UVVH: 2005-07 N=592	UVVH: 2008-10 N=718	UVVH: 2011-14 N=1003	UVVH*: 2005-14 N=2313	Andalusia: 2005-07 N=6322	Andalusia: 2008-14 N=6727	Andalusia: 2011-14 N=10118	Andalusia**: 2005-14 N=23167
Reperfusion	75%	79%	93%	84%	67%	74%	83%	76%
No reperfusion	25%	21%	7%	16%	33%	26%	17%	24%
Fibrinolysis	57%	44%	27%	40%	61%	56%	40%	50%
Primary Percutaneous Coronary Intervention	18%	34%	66%	44%	6%	18%	43%	26%
Rescue Percutaneous Coronary Intervention	11%	20%	38%	22%	7%	21%	33%	22%
Intensive Care Unit Mortality	6.8%	5.7%	5%	5.7%	5.9%	5.9%	4.7%	5.4%

*ICU* intensive care unit, *UVVH* University Virgen de la Victoria Hospital

(*)Chi Square p= 0,000

(**)Chi Square p=0,000

### A192 Levosimendan for low cardiac output state - a retrospective analysis

#### J. Aron, R. Marharaj

##### Kings College Hospital, Intensive Care Unit, London, United Kingdom

###### **Correspondence:** J. Aron – Kings College Hospital, Intensive Care Unit, London, United Kingdom

**Introduction** Levosimendan improves haemodynamic performance and may have cardio-protective effects. Small trials have demonstrated efficacy in well-defined populations (1). There is no consistent evidence of mortality reduction, however a recent meta-analysis (2) demonstrated that levosimendan reduced mortality in the overall population and subpopulations of cardiac surgery and cardiology.

**Objectives** Given the uncertain benefits, an analysis of the utilisation of levosimendan for low cardiac output states was undertaken.

**Methods** The retrospective analysis was performed at a single center. Data from 62 admissions due to low cardiac output state, treated with levosimendan, was retrospectively abstracted; demographics, illness severity, co-morbidity, haemodynamic, metabolic, biochemical, resource utilisation, organ support and hospital outcomes where analysed.

**Results** The population group had a mean age of 59.2 years (range 19-84) and a mean APACHE II score of 23.7 (range 7 - 61). Causes of low CO state included myocardial infarction (n = 14, 22.5 %), after cardiac arrest (n = 6, 9.7 %), after cardiac surgery (n = 7, 11.3 %), septic cardiac dysfunction (n = 5, 8.1 %), acute cardiomyopathies (n = 7, 11.3 %) amongst other causes (n = 23, 37 %).

Levosimendan was received at a variable point during their ICU treatment (mean day 3.7, range 0 -14 days) and was usually the 3rd inotrope (range 0-5) commenced.

Treatment with levosimendan resulted in significant improvements in CI (p = 0.013) and acidosis (p < 0.0001) . There was a trend towards improved lactate clearance and oxygenation.

Overall length of stay (LOS) in ICU was 12.7 days (range 1 - 60), 30-day mortality was 59.7 % and survival to discharge was 38.7 %.

Commencing levosimendan within 48 hours after admission resulted in decreased duration of ventilation

(p 0.013) and ICU LOS (p 0.0009), with a trend towards a reduction in length of renal replacement therapy (p 0.06) (Table 64). Similar benefits were not demonstrated if levosimendan was introduced as a first or second vasoactive agent compared to a third or fourth agent. However survival to discharge was improved (41.5 % vs 33.3 % respectively).

**Conclusions** When levosimendan was used early in the disease process ICU LOS and duration of ventilation were reduced. When used as a first or second inotrope, rather than a third or fourth, survival to discharge was higher. More research is required to determine the optimum timing of therapy.

**Reference(s)**

1. S Mathieu, G Craig; Levosimendan in the treatment of acute heart failure, cardiogenic and septic shock: a critical review. JICS 2011;12, 15-24

2. G Landoni et al; Effects of levosimendan on mortality and hospitilisation.

A meta-analysis of RCT; CCM 2012;40.2

**Table 65 (abstract A192).** Outcome measures

	< Day 2 Commenced	> Day 2 Commenced	P value
Total no.	38	24	
RRT days m (range)	5 (1 -41)	9.7 (0-50)	0.06
Vent days, m (range)	6.08 (0-32)	10.7 (1-23)	0.013*
ICU LOS, m (range)	8.44 (1 -41)	18.6 (3-60)	0.0009*
30-day mort, n (%)	24 (63.2%)	13(54.2%)	
Survival to discharge, n (%)	14 (36.8%)	10 (41.7%)	
Hosp LOS in survivors, m (range)	35.6 (14 - 121)	45.7 (7 -114)	0.45

### A193 Clinical predictors of death in patients with cardiogenic shock: an observational retrospective study

#### K. Gervasio, M. Bottiroli, M. Mondino, D. De Caria, A. Calini, E. Montrasio, F. Milazzo, M.P. Gagliardone

##### Anesthesia and Critical Care Medicine, Cardiothoracic Department 'A. De Gasperis', ASST Grande Ospedale Metropolitano Niguarda, Milan, Italy

###### **Correspondence:** M. Bottiroli – Anesthesia and Critical Care Medicine, Cardiothoracic Department 'A. De Gasperis', ASST Grande Ospedale Metropolitano Niguarda, Milan, Italy

**Introduction** Cardiogenic shock (CS) is a syndrome characterized by tissue congestion and hypo-perfusion consequently to cardiac pump failure. While many studies have evaluated the outcome of post-ischemic CS there is a paucity of data on non-ischemic CS etiology.

**Objectives** The aim of this study is to identify independent predictors of death for CS caused by different etiologies.

**Methods** We performed a retrospective observational study on patients with CS admitted in our ICU from Jan 2010 to Dec 2014. Patients with postcardiotomy and/or post-transplant CS were excluded. Demographic, clinical and biochemical variables were collected. Continuous variables are presented as median (IQR). Data were analyzed using comparative statistics and multivariate analysis was performed with Cox regression.

**Results** We recruited 80 patients. Etiologies of CS were: 33 acute decompensation of chronic cardiomyopathies, 16 acute myocarditis, 16 acute myocardial infarctions and 15 other causes. Baseline variables are reported in Table 66 (*p < 0,05).

In the first six hours after presentation all patients received inotropes, 79 % needed invasive mechanical ventilation, 75 % intra aortic ballon pump and 29 % VA ECMO for refractory CS. 27 patients died during their ICU stay while 53 survived. 39 patients were bridged to recovery, 11 patients to heart transplant and 6 patients to left ventricle assist device (LVAD). No differences for etiology at ninety days mortality were observed (Kaplan-Meyer; log-rank p = 0,41). Non-survivors were characterized by a higher need for inotropes dose and VA ECMO assistance rate in the first 6 hours (inotropic score: 14 (8-20) vs. 24 (14-30), p = 0.01; VA ECMO: 11/53 vs 12/27, p = 0,037). Multivariate analysis revealed Lactate at ICU admission as an independent risk factor for hospital mortality at ninety days: HR 1,18 (per mmol/l) IC95% 1,09-1,27 p = 0,001.

**Conclusions** Hyperlactatemia at presentation is an independent predictor of death in CS caused by different etiologies. It is likely that this is due to a more severe state of global hypoperfusion or a prolonged state of shock.

**Table 66:** (abstract A193).

	Survivors (n=53)	Non-survivors (n=27)
Age (years)*	44 (25-62)	51 (44-64)
Lactate (mmol/l)*	2,5 (1,0-5,2)	5,1 (2,8-12)
Base Excess (mmol/l)*	-2(-6-0,6)	-6(-8 - -2)
Mean Arterial Pressure (mmHg)	67 (58-73)	68 (59-73)
Heart Rate (bpm)	94 (82-120)	97 (88-120)
SOFA score*	9 (7-12)	12 (11-14)
Bilirubine (mg/dl)	0,9 (0,5-1,4)	1,3 (0,7-2,3)
Creatinine (mg/dl)	1,1 (0,7-2)	1,7 (1,3-2,3)
Platlets (10^9/l)*	251 (151-343)	170(111-274)

### A194 Thrombolyisis therapy and mortality in patients with ST elevation in myocardial infarction during 2005 to 2014 period

#### A. Vallejo-Báez, M.V. de la Torre-Prados, P. Nuevo-Ortega, A. Fernández-Porcel, E. Cámara-Sola, T. Tsvetanova-Spasova, C. Rueda-Molina, L. Salido-Díaz, A. García-Alcántara, and ARIAM group

##### University Hospital Virgen de la Victoria/IBIMA, Department of Intensive Care Unit, Málaga, Spain

###### **Correspondence:** P. Nuevo-Ortega – University Hospital Virgen de la Victoria/IBIMA, Department of Intensive Care Unit, Málaga, Spain

**Introduction** Early reperfusion may prevent damage to the myocardium, improves left ventricular function and prognosis of patients suffering a ST elevation in acute myocardial infarction (STEMI).

**Objectives** To analyze the mortality in the groups of patients with STEMI and fibrinolytic therapy in relation with the thrombolysis place and hours of symptom onset

**Methods** It is a prospective cohort study of patients diagnosed of STEMI admitted to a coronary Intensive Care Unit (ICU) at a second level hospital, University Hospital Virgen de la Victoria (UHVV) during three periods, 2005 to 2007 (n = 354), 2208-2010 (n = 323) and 2011-2014 (n = 269). The data belong to ARIAM (Delay Analysis Acute Myocardial Infarction) record. The variables studied are ICU mortality, thrombolysis place (prehospital, emergency or ICU) and time in minutes after symptom onset (>60,> 120 and > 180). All patients receive rescue or facilitated percutaneous coronary intervention (PCI). Descriptive and comparative statistical analysis was performed using the statistical software packages SPSS version 18.0 (SPSS Inc., Chicago, IL, USA).

**Results** We studied 946 patients with a mean age of 61.8 ± 12.2 years (61.3 ± 12.3 vs. 61.9 ± 12.5 vs. 62.2 ± 11.69, p= > 0.05) and 22.7 % were female (22 % vs. 23 % vs. 23 %; p= > 0.05), no significant differences between three periods. The results we can see in the Table 66.

**Conclusions** In three periods study there was significant differences between mortality and thrombolysis place in STEMI. Early thrombolysis in prehospital and emergency hospital areas showed decreased in mortality ICU.

**Reference(s)**

McCaul M, Lourens A, Kredo T. Pre-hospital versus in-hospital thrombolysis for ST-elevation myocardial infarction. Cochrane Database Syst Rev. 2014 Sep 10;9:CD010191

**Grant acknowledgement**

ARIAM spanish record.

**Table 67 (abstract A194).** Thrombolysis and Mortality in STEMI

Period Studies, n	P1, n=354	P1, n=354	P2, n=323	P2, n=323	P3, n=269	P3, n=269	Pt, 2005-2014, n=946	Pt, 2005-2014, n=946
Place and Time	Fx, n/%	M, n/%	Fx, n/%	M, n/%	Fx, n/%	M, n/%	M, n/%	Total Fx, n
Prehospital*, n/%	48/16	6/12.5	118/40	3/2.5	132/44	6/4.5	15/5	298
Emergency*, n/%	265/45	14/5.3	193/32	13/6.7	137/23	0/0	27/4.5	595
ICU, n/%	24/75	3/12.5	7/22	1/14.3	1/3	0/0	4/12.5	32
<60 minutes n/%	22/23	0/0	36/37	1/2.8	39/40	1/2.6	2/2.1	97
<120 minutes n/%	136/33	8/5.9	145/35	4/2.8	134/32	4/3	16/3.9	415
<180 minutes n/%	234/36	14/6	221/34	9/4.1	204/30	4/2	27/4	659

*P1* 2005-2007, *P2* 2008-2010, *P3* 2011-2014, *Pt* 2005-2014, *n* number, *%* percentage, *Fx* Fibrinolysis, *M* mortality

(*)Chi Square, p=<0.05

### A195 Risk heterogeneity and long term prognosis of heart failure complicating acute miocardial infarction

#### L. Sayagues Moreira^1^, R. Vidal-Perez^2^, U. Anido^1^, C. Pena Gil^2^, J.M. Garcia Acuna^2^, P. Rascado Sedes^1^, C. Martin Lopez^1^, E. Saborido Paz^1^, J.L. Garcia Allut^1^, C. Galban Rodriguez^1^, J.R. Gonzalez-Juanatey^2^

##### ^1^Complexo Hospitalario Universitario de Santiago de Compostela, Intensive Care, Santiago de Compostela, Spain; ^2^Complexo Hospitalario Universitario de Santiago de Compostela, Cardiology, Santiago de Compostela, Spain

###### **Correspondence:** L. Sayagues Moreira – Complexo Hospitalario Universitario de Santiago de Compostela, Intensive Care, Santiago de Compostela, Spain

**Introduction** The Killip class classification for heart failure it is used to predict short-term mortality in patients with acute coronary syndrome (ACS)

**Objectives** To determine the contemporary long-term prognosis of ACS with acute heart failure graded according to the Killip classification.

**Methods** Cohort study of consecutive hospitalized patients with ACS diagnosis from 2004 to 2009. Follow-up was done by clinical review or telephone contact and death or cardiovascular events were recorded, as well as the cause of death

**Results** 5070 patients were included with a complete follow up after a mean of 5.8 ± 2.6 years. The clinical characteristics were analyzed in relation with Killip class at admission (shown in Table). A stepwise gradient in the adjusted hazard ratio (HR) for mortality was observed with increasing Killip class: class > I HR 4.35 (95 % CI 3.81 to 4.97) unexpectedly, in a landmark analysis excluding deaths < 30 days after admission, patients in Killip class IV had a lower adjusted long-term mortality than those in class III (shown in Figure).

**Conclusions** The heterogeneity in early versus late risk in patients with Killip class IV heart failure it is present in our contemporary cohort highlighting the importance of an appropriate early treatment in cardiogenic shock patients.

**Table 68 (abstract A195).** Characteristics by Killip Class

	KILLIP I N:4305 (84.9%)	KILLIP II N:554 (10.9%)	KILLIP III N:146 (2.8%)	KILLIP IV N:65 (1.4%)	P-Value
Mean Age (years)	64,5 (SD 13)	72 (SD 13)	77 (SD 7.9)	66.8 (SD 12.9)	0.000
Sex (% male)	3085 (71.7%)	358 (64.6%)	93 (63.7%)	48 (73.8%)	0.001
Hypertension (%)	2373 (55.1%)	345 (62.3%)	102 (69.9%)	26 (40%)	26 (40%) 0.000
Diabetes (%)	1049 (24.4%)	218 (39.4%)	69(47.3%)	17 (26.2%)	0.170
Hyperlipidemia (%)	2054 (47.7%)	237(42.8%)	53 (36.3%)	16 (24.6%)	0.170
ACS with ST elevation(%)	1237 (28.4%)	190 (33.3%)	51 (32%)	51 (69%)	0.000
Prior PCI (%)	370 (8.6%)	40 (7.2%)	9 (6.2%)	2 (3.1%)	0.209
Prior Heart Failure (%)	115 (2.7%)	76 (13.7%)	20 (13.7%)	5 (7.7%)	0.000
Mortality at end follow up (%)	610 (14.1%)	284 (51.3%)	103 (70.5%)	45 (69.5%)	0.000

**Fig. 85 (abstract A195).**
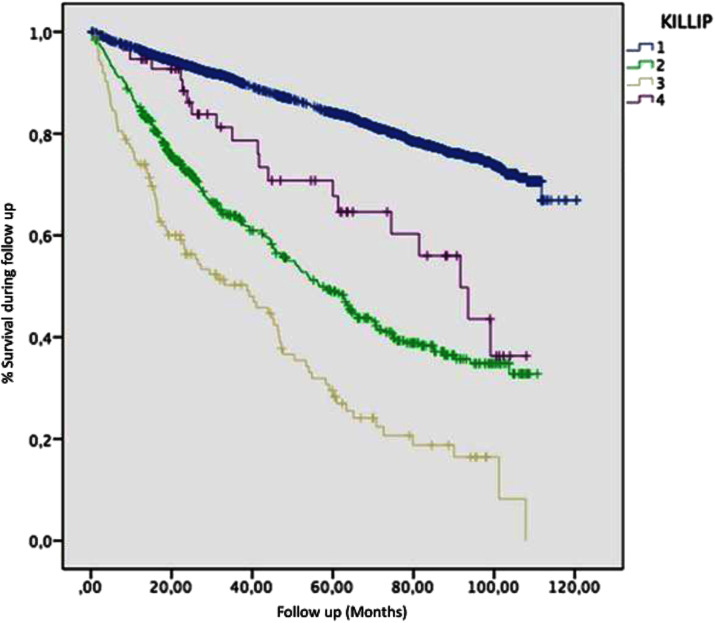
Mortality in follow up by Killip Class

### A196 Type 2 myocardial infarction in the ICU: incidence, descriptive analysis and outcome

#### Y. Hamdaoui^1^, A. Khedher^1^, M. Cheikh-Bouhlel^2^, J. Ayachi^1^, K. Meddeb^1^, N. Sma^1^, N. Fraj^1^, N. Ben Aicha^1^, S. Romdhani^1^, R. Bouneb^1^, I. Chouchene^1^, M. Boussarsar^1,3^

##### ^1^Farhat Hached University Hospital, Medical Intensive Care Unit, Sousse, Tunisia; ^2^Farhat Hached University Hospital, Cardiology Department, Sousse, Tunisia; ^3^Ibn Al Jazzar Faculty of Medicine, University of Sousse, Research Laboratory N° LR14ES05. Interactions of the Cardiopulmonary System, Sousse, Tunisia

###### **Correspondence:** Y. Hamdaoui – Farhat Hached University Hospital, Medical Intensive Care Unit, Sousse, Tunisia

**Introduction** Myocardial infarction (MI) has known several sub-classifications through the time. A consensus statement classified MI by clinical scenario into various subtypes. Type 2 MI is now defined as myocardial injury with necrosis where a condition other than CAD contributes to an imbalance between myocardial oxygen supply and/or demand (1). Several mechanisms were identified to lead to this type of MI. These conditions are under-recognized in the critically-ill.

**Objectives** To appreciate the incidence of type 2 MI in medical ICU patients.

To identify risk factors associated with type 2 MI.

**Methods** A retrospective chart review of consecutive patients discharged from a medical ICU with the final diagnosis of type 2 MI. Two chart reviewers (senior intensivists) studied all the medical records using an explicit protocol. Were analyzed, demographics, underlying conditions, clinical characteristics, therapeutic interventions and outcome.

**Results** From January 2010 to December 2015, a total of 1809 patients were hospitalized in a 7-bed medical ICU. 62(3.4 %) patients met the definition criteria of type 2 MI. Median (IQR) age was 50[15-85] years. 36(58 %) were male and 5(8 %) were obese, with diabetes mellitus in 8(13 %); arterial hypertension, 18(29 %); CHF, 8(13 %) ; chronic respiratory failure, 25(41 %). On ICU admission, 40(65 %) had acute respiratory failure; 26(42 %), shock, 7(15 %), near drowning and 3(5 %) had carbon monoxide poisoning. Median (IQR) SAPS II was 42[6-78]; PaO2/FIO2 ratio, 204[32-367]; lactate level, 4.8[0.7-8.9] mmol/l; pH, 7.15[6.80-7.51] and bicarbonates at 25[6-37] mmol/l. 55(89 %) patients underwent TTE, 30(55 %) had kinesis abnormalities. Median (IQR) cTn I level was 9[0-18] ng/ml. 13(21 %) had a coronary angiography, 100 % with no luminal irregularities. 49(78 %) had invasive mechanical ventilation and received vasopressors. 20(31 %) patients received anti-ischemic, anti-thrombotic and/or anticoagulant therapy. 3(4 %) received fibrinolytic therapy. 34(55 %) demonstrated complete regression of all abnormalities after the correction of respiratory and/or hemodynamic distresses. 30(48.4 %) died.

**Conclusions** Type 2 MI reveals rather common and frequently misdiagnosed in ICU patients. This lead to management delay and errors. Patients are rather young, without underlying cardiovascular risk factors. They exhibit a myriad of clinical conditions in which the common factor is oxygen supply-demand mismatch. They have less cTn I levels but relatively high mortality related to the associated illness severity.

**References** 1 Kristian Thygesen, Joseph S. Alpert, Allan S. Jaffe, Maarten L. Simoons, Bernard R. Chaitman and Harvey D. White: the Writing Group on behalf of the Joint ESC/ACCF/AHA/WHF Task Force for the Universal Definition of Myocardial Infarction. Third Universal Definition of Myocardial Infarction.2012. Journal of the American College of Cardiology 2012; 16:1581-98.

### A197 Effectiveness of has- bled score in assessing risk of bleeding among adult Filipino patients admitted in the medical intensive care unit in a tertiary hospital

#### M.P.R.D.L. Dela Cruz, J.M. Bernardo

##### Makati Medical Center, Internal Medicine, Makati, Philippines

###### **Correspondence:** M.P.R.D.L. Dela Cruz – Makati Medical Center, Internal Medicine, Makati, Philippines

**Background** HAS- BLED scoring in assessment of bleeding risk among patients with atrial fibrillation has been proven effective in risk stratification. This helps in anticoagulation management as well as in assessing morbidity and mortality of patients with atrial fibrillation who are on anti coagulants.

**Objective** This study aims to show if HASBLED scoring is also useful in bleeding risk assessment among patients without atrial fibrillation admitted in the medical intensive care unit.

**Study design** This is a single centered retrospective cross sectional Study.

**Methods** Descriptive statistics will be reported as mean ± SD, median (IQR) or proportion (%) as applicable and presented in tables or graphs. Cox regression analysis was used to determine association of clinical risk factors with bleeding, cardiovascular events and mortality. Hazard-ratios and predictive values were also estimated.

**Participants** Included in this study were Filipino patients ages 19 and above, admitted to the Medical ICU from the Emergency Room with outcome seen during the same admission and those patients who satisfied all criteria for the HAS BLED scoring. Excluded are patients who are known case of Atrial Fibrillation, those admitted for any episode of active bleeding. and patient admitted for major surgery.

**Results** A total of 281 patient charts of which only 231 were included in the study. The study was able to risk stratify patients who are high risk for bleeding both patients with or without atrial fibrillation. Twenty percent of the patients had atrial fibrillation. The majority of the patients were classified as high risk for bleeding (HAS-BLED) and for venous thromboembolic risk (Caprini). The most common outcome of patients was Acute Coronary Syndrome. Those with a HASBLED score of 3 or more, where in 107 (46 %) out of 231 had an acute coronary event through the course of admission. Among the 231 respondents, 69 of them had any episode of bleeding, both minor and major. AF and Non-AF on HAS-BLED score are not significant with p-value of 0.1048. However the odds ratio presents 10.8391 (0.6086 to 193.0421) which means that the AF respondents as compared to Non-AF are 10.839 times likely (more likely) to have a HAS-BLED score greater than or equal to 3 than HAS-BLED < 3.

It also showed that those with high HASBLED score are at high risk for bleeding, even among patitnes without atrial fibrillation. However, on statistical analysis, it did not show clinical significance. HAS-BLED and Caprini scores were found to be directly and moderately correlated, with Spearman's rho = 0.386 (p-value < 0.005).

**Conclusion** Patients admitted in the critical care unit, regardless if with or without history of atrial fibrillation, both HASBLED and Caprini scoring should be done to risk stratify patients for bleeding, and longer period of observation is warranted.

### A198 Protective effect of MGC-0109 following ischemia/reperfusion injury

#### F. Galfo^1,2^, A. Dyson^1,3^, M. Singer^1,3^

##### ^1^University College London, Bloomsbury Institute of Intensive Care Medicine, London, United Kingdom; ^2^University of Messina, Depart. of Clinical and Experimental Medicine, Messina, Italy, ^3^Magnus Oxygen Ltd, London, United Kingdom

###### **Correspondence:** F. Galfo – University College London, Bloomsbury Institute of Intensive Care Medicine, London, United Kingdom

**Introduction** As myocardial infarction can result in permanent ischaemic damage, early revascularization is vital to spare myocardium. However, the downside of this treatment is reperfusion injury, and this in itself will contribute to long-term ventricular dysfunction. Strategies attenuating reperfusion injury have, to date, proved unsuccessful. As reactive oxygen species (ROS) derived from the mitochondrial electron transport chain (ETC) are the main cause of reperfusion injury, modulation of oxidative phosphorylation (peri-revascularization) may confer benefit. Sulphide donors reversibly inhibit complex IV of the ETC so this approach offers putative benefit.

**Objectives** To test the slow-release sulphide donor, MGC-0109, in a cell (cardiomyocyte) model of ischaemia/reperfusion injury.

**Methods** H9C2 cells were subjected to 24 h hypoxia followed by 2 h reoxygenation (in room air). Cells received MGC-0109 (0.005-5.5 mM) or vehicle (cell medium) at the beginning of the reoxygenation period. In separate experiments, normoxic cells were treated with the ROS, hydrogen peroxide (H_2_O_2_) 500 microM for 4 h with MGC-0109 (5.5.mM) added to half the wells after 1 h. Cell viability was assessed by flow cytometry using an Annexin V/PI assay. One-way ANOVA and Tukey post-hoc testing (SPSS v20) was used to test for statistical significance.

**Fig. 86 (abstract A198).**
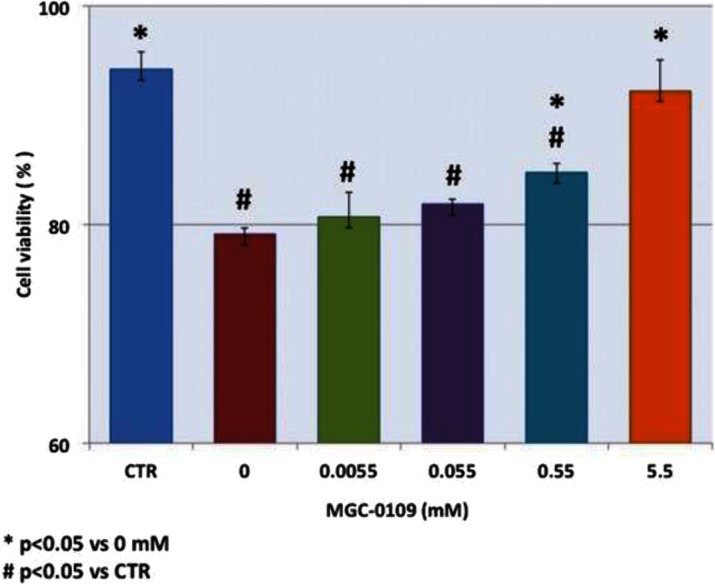
Cell viability following I/R injury. # p < 0.05 vs CTR (no I/R) and *p < 0.05 vs 0 mM (I/R, untreated)

**Results** Ischaemia/reperfusion reduced cell viability from 94 % to 79 % (Fig. 86). MGC-0109 adminstered at the onset of reperfusion increased cell survival in a dose-dependent manner. At the highest concentration survival was similar to cells that did not undergo I/R. Protective effects were also seen with addition of MGC-0109 to H_2_O_2_-treated normoxic cells (data not shown).

**Conclusions** MGC-0109 reversed cell death related to reperfusion injury. Further studies are ongoing to confirm if its mechanism of action is via mitochondrial ETC inhibition.

### A199 Haemodynamic and perfusion markers relate to insult severity in myocardial ischaemia/reperfusion injury

#### A. Marino^1,2^, A. Dyson^1,3^, M. Singer^1,3^

##### ^1^University College London, Bloomsbury Institute of Intensive Care Medicine, London, United Kingdom; ^2^Università degli Studi di Milano, Dipartimento di Fisiopatologia Medico-Chirurgica e dei Trapianti, Milan, Italy; ^3^Magnus Oxygen Ltd, London, United Kingdom

###### **Correspondence:** A. Marino – University College London, Bloomsbury Institute of Intensive Care Medicine, London, United Kingdom

**Introduction** Rapid revascularisation is the treatment of choice to minimize ischaemic injury in acute myocardial infarction. However, this procedure can itself induce reperfusion injury that may further damage the myocardium [1]. Experimental models of myocardial infarction and reperfusion involve temporary ligation of the left anterior descending coronary artery (LAD). This model, though representative of the clinical scenario, is associated with infarcts of variable size, and, consequently, insult severity [2]. However, peri-procedural haemodynamics are not well described.

**Objectives** To assess the impact of insult severity on cardiac, haemodynamic and perfusion markers.

**Methods** Eight instrumented, mechanically ventilated, thoracotomized, male Wistar rats (250-300 g) were subjected to 20 minutes of myocardial ischaemia by temporary occlusion of the LAD. Animals were monitored for up to 240 min post-reperfusion. At baseline, end-ischaemia and at hourly intervals post-reperfusion, mean arterial pressure (MAP), echocardiographic and arterial blood gas measurements were performed. Aortic peak systolic blood flow velocity (Vmax) was used as a marker of myocardial contractility. At experiment end the infarct area/left ventricle ratio (IA/LV) was assessed by histology to determine insult severity. Comparisons were drawn between mild (<19 % IA/LV, n = 4) and severe (>19 % IA/LV, n = 4) insults, with analysis by two-way ANOVA. P values < 0.05 were considered significantly different. Normality was tested by the Shapiro-Wilk test.

**Fig. 87 (abstract A199).**
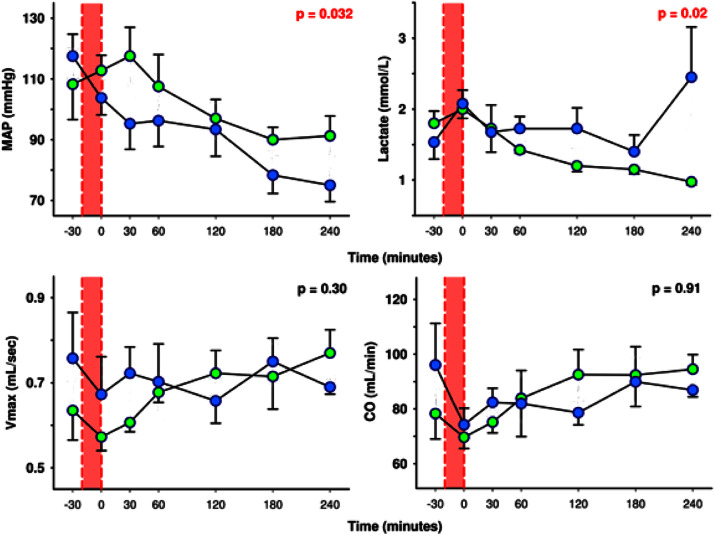
Timecourse of haemodynamic, contractility and perfusion markers in mild (IA/LV < 19 %, green dots) and severe (IA/LV > 19 %, blue dots) animals. Ischaemia time shown as red area

**Results** Insult severity (IA/LV ratio) was normally distributed with a mean ± SEM of 19 ± 10 %. The severe group (IA/LV > 19 %) had significantly lower MAP and higher lactate values than the mild severity group (Fig. 87, top panel). Temporal changes in cardiac contractility and output did not however relate to insult severity (Fig. 87, bottom panel).

## Conclusions

In an experimental rat myocardial ischaemia/reperfusion injury model, blood pressure and metabolic impairments related to insult severity. By contrast, cardiac contractility and cardiac output showed no significant temporal change.

**References**

[1] Frank A et al. *Semin Cardiothorac Vasc Anesth.* 2012; 16:123-32

[2] van den Bos EJ et al. *Am J Physiol Heart Circ Physiol*. 2005; 289:H1291-300

### A200 Using smartphone application for transmitting ECG to reduce time of percutaneous intervention

#### C.C. Chao^1,2^, P. Hou^3,4^

##### ^1^Taipei Medical University Hospital, Emergency Department, Taipei, United States; ^2^Taipei Medical University, Taipei, Taiwan, Province of China; ^3^Harvard Medical School, Boston, United States, ^4^Brigham and Women's Hospital, Boston, United States

###### **Correspondence:** C.C. Chao – Taipei Medical University Hospital, Emergency Department, Taipei, United States

**Introduction** How to reduce the time of percutaneous intervention in patients diagnosed with ST elevation myocardial infarction (STEMI) is a critical issue for care process. Many interventions such as prehospital ECG, cardiac catheterization laboratory (CCL) activated by emergency physician (EP) have been proved to improve the result(1). However discordance in diagnosis between the EP and the interventional cardiologist may result in unnecessary activation of the CCL. Communication between EP and interventional cardiologist is often verbal report via telephone. After Development of the smartphones, EPs can use application to transmit ECG images to interventional cardiologists when STEMIs were diagnosed.

**Objectives** This study aims to evaluate the use of smartphone application to facilitate communication between the EP and the interventional cardiologist in order to minimize the time to activate CCL and time of percutaneous intervention.

**Methods** We retrospectively collected time-point of every step in management and outcome of patients diagnosed with STEMI in the ED. Total 84 patients were enrolled. In group 1, patient's ECG was described by traditional verbal communication via telephone. In group 2, we use smartphone application for transmitting ECG to interventional cardiologist. Time-points of intervention were recorded for analysis.

**Results** Time of ECG done was not significantly different between the groups, 9.8 ± 3.3 and 7.8 ± 2.3 minutesThe D2B time was 119.3 ± 16.3 minutes in group 1 compared with 90.4 ± 9.8 minutes in group 2

(p = 0.13). The reduction in the D2B time was driven by a reduction in time from ECG interpretation to activate CCL (28.3 ± 4.1 in the group 1 and 17.6 ± 2.3 minutes in the group 2) (p = .001) shown in Table 69. Mortality rate in group 1 is 12.5 % compare to 2.2 % in group 2 (p = .07), shown in Table 69.

**Conclusions** Usage smartphone to transmit ECG in the ED to interventional cardiologists can facilitate communication and reduce the time of CCL activation and balloon inflation.

**Reference(s)**

1. Peterson MC, Syndergaard T, Bowler J, Doxey R. A systematic review of factors predicting door to balloon time in ST-segment elevation myocardial infarction treated with percutaneous intervention. International journal of cardiology. 2012;157(1):8-23.

**Table 69 (abstract A200).** Group 1 using telephone; group 2 using smartphone

	Group 1 (n=40) (95 % CI)	Group2 (n=44) (95 % CI)	P value
Sex, Male	34	40	0.40
Age, years	60.0±1.8 (56.2~63.7)	64.4±2.1 (60.2~68.5)	0.11
Time to ECG Time to ECG done, minutes	9.8±3.3 (3.1~16.5)	7.8±2.3 (3.6~11.5)	0.57
Time to ECG interpretation by EP, minutes	11.8±3.5 (4.6~19.0)	8.6±2.0 (4.6~12.7)	0.44
Activate CCL time, minutes	54.5±12.7 (28.8~8.02)	34.7±9.0 (16.5~52.8)	0.10
EP read ECG to CCL activation time, minutes	28.3±4.1 (19.9~36.7)	17.6±2.3 (13.1~22.1)	0.01
EP read ECG to balloon inflation time, minutes	93.1±9.5 (73.9~112.4)	73.4±3.9 (65.6~81.2)	0.025
D2B, minutes	119.3±16.3 (86.4~152.2)	90.4±9.8 (70.7~110.1)	0.13
Mortality rate	Mortality rate 12.5% (Expire 5; Survive 35)	Mortality rate 2.2% (Expire 1; Survive 43)	0.07

### A201 The application of innovative design of an electrocardiogram exam accessory device to improve ambulance prehospital electrocardiogram implantation rate in a city based multicenter trial

#### W.-C. Huang^1^, C.-C. Hung^1^, C.-H. Chiang^2^, W.-T. Hung^1^, K.-C. Lin^1^, S.-C. Lin^1^, Y.-J. Liou^3^, S.-M. Hung^3^, Y.-S. Lin^3^, C.-C. Cheng^2^, F.-Y. Kuo^2^, K.-R. Chiou^2^, C.-J. Chen^4^, L.-S. Yan^4^, C.-Y. Liu^4^, H.-H. Wang^4^, P.-L. Kang^2^, H.-L. Chen^3^, C.-K. Ho^4^, G.-Y. Mar^2^, C.-P. Liu^4^

##### ^1^Kaohsiung Veterans General Hospital, Critical Care Division, Kaohsiung City, Taiwan, Province of China; ^2^Kaohsiung Veterans General Hospital, Cardiovascular Division, Kaohsiung City, Taiwan, Province of China; ^3^Kaohsiung City Government, Fire Bureau, Kaohsiung City, Taiwan, Province of China; ^4^Kaohsiung City Government, Department of Health, Kaohsiung City, Taiwan, Province of China

###### **Correspondence:** C.-H. Chiang – Kaohsiung Veterans General Hospital, Cardiovascular Division, Kaohsiung City, Taiwan, Province of China

**Introduction**

The prehospital electrocardiogram (ECG) was identified as an critical part of treatment for patients with STEMI. However, it remained a challenging issue to set up prehospital ECG in Asia.

**Objectives**

This study is to investigate the application of innovative design of an electrocardiogram exam accessory device to improve ambulance prehospital ambulance electrocardiogram implantation rate via in a city based multicenter trail.

**Method**

This study started since Sep, 2011 via a multidisciplinary team among Kaohsiung veterans General hospital, fire bureau and department of health, Kaohsiung city government. The unique accessory device for 12 lead electrocardiography apparatus has 10 holes, which are arranged according to a standard electrode placement for the 12 lead ECG measurements. The design of inter-nipple line and mid-sternum line on the device can assist the staffs to perform ECG shortly. This breakthrough innovation designed to address the core issue of the efficiency of the ambulance pre-hospital ECG system. Therefore, the invention successfully promoted Kaohsiung city council to set up Asian first ambulance prehospital telemetry electrocardiogram system. The innovative design of a ECG exam accessory device was patented in Taiwan and won golden award in Geneva and Korean international invention. The consecutative chest pain patients received ambulance ECG exam were enrolled from Jan. 2011 to September. 2015 in 18 different fire brigades at Kaohsiung city. The ECG implementation rate is defined as chest pain patients received ambulance ECG exam divided by all patients with chest pain.

**Results**

The ECG implementation rate increased from 0 % in pre-interventional to 62.2 % in post-interventional group (p < 0.001). Total 66 patients with STEMI was detected in 1205 chest pain patients received ambulance ECG exam. In these STEMI patients, average door to balloon time was 51 minutes, average ischemia to balloon time was 125 minutes and in-hospital mortality was 0 %.

Conclusions

This study demonstrates that application of innovative design of an electrocardiogram exam accessory device can solve the main problem of system and assist to set up first ambulance prehospital telemetry electrocardiogram system in Asia and further improve ambulance prehospital electrocardiogram implantation rate in Kaohsiung city.

## QUALITY AND SAFETY I

### A202 To investigate the effect of increased ICU demand on the quality of care provided

#### S. Grewal^1^, S. Gopal^1^, C. Corbett^2^

##### ^1^New Cross Hospital, Integrated Critical Care Unit, Wolverhamptom, United Kingdom; ^2^New Cross Hospital, Critical Care Services, Research and Audit Office, Wolverhamptom, United Kingdom

###### **Correspondence:** S. Grewal – New Cross Hospital, Integrated Critical Care Unit, Wolverhamptom, United Kingdom

**Introduction**

The demand for critical care services is increasing steadily both nationally and internationally. It is important that with increasing demand the quality of care delivered is maintained. A well-recognised quality marker for intensive care units(ICU) are readmissions within 48 hours of discharge[1]. ICU readmission is associated with significantly increased morbidity, mortality, prolonged hospital admissions and increased cost[2,3]. The reported average of unplanned readmissions is 1.4 % in the UK[4]. Globally this varies between 1.3-13.7 %[2]. There have been no specific causal factors found[2].

**Objectives**

Identify the 48 hour readmission rate over two consecutive years and its impact on patient care.

**Method**

This was a retrospective study in a 16 bedded adult general medico-surgical ICU in a large acute hospital in England. The sample was collated from the ICU discharges in 2014 and 2015, identifying those patients who were readmitted within 48 hours of discharge. Specific times of discharge and readmission, underlying cause, length of readmission stay and final outcome data was collected. The results of 2014 and 2015 were compared.

**Results**

In 2014, 672 patients were discharged from ICU, with 21 readmissions and only 2 within 48 hours (0.3 %). The time of re-admission ranged from 14 to 48 hours. Following readmission, the length of stay ranged from 10 days to 17 days.

In 2015, there were 702 discharges with 30 readmissions. 14 of these were within 48 hours (2.0 %). The time of readmission varied from 9 to 48 hours (median = 25 hours). Cardio-respiratory failure was the most common cause for readmission. The length of the readmission stay on ICU ranged from 1 day to 68 days (median = 8 days).

Over the two year period the ICU mortality rate remained unchanged at 16 % however patients readmitted within 48 hours showed considerably higher hospital mortality rates of 50 % in 2014 and 42 % in 2015.

**Conclusions**

Over the two year period, there was an increase in admissions and discharges from ICU. This was associated with an increase in the midnight bed occupancy rate from 79.5 % in 2014 to 84.3 % in 2015. However, this was linked with a 7 fold rise in readmissions within 48 hours and an associated hospital mortality rate higher than both the previous year and national average.

An increasing demand for ICU services can have an adverse effect on the quality of care delivered. Further research is needed to inform clinicians and commissioners how quality can be maintained with increasing demand.

**References**

1. The Faculty of Intensive Care Medicine: Core Standards for Intensive Care, Edition1 (2013)

2. Elliott M, et al. Intensive Care Readmission: A contemporary review of literature. Intensive Crit Care (Nurse) 2013. http://dx.doi.org/10.1016/j.iccn.2013.10.005

3. Roseberg AL et al. Patients readmitted to ICUs: A systematic review of risk factors and outcomes. Chest 2000;118(2):492-502.

4. ICNARC Case Mix Programme 2012-13

### A203 'Ventsafe': a quality improvement project

#### A. Wilson, J. Capps, W. Ayoub, A. Lomas, S. Ghani, J. Moore, D. Atkinson, M. Sharman

##### Central Manchester Foundation NHS Trust, Adult Critical Care, Manchester, United Kingdom

###### **Correspondence:** A. Wilson – Central Manchester Foundation NHS Trust, Adult Critical Care, Manchester, United Kingdom

**Introduction**

There is growing evidence that a protective lung ventilation strategy offers benefits to all patients undergoing mechanical ventilation[1]. In addition, the deleterious effects of excess oxygen in critically ill patients are becoming increasingly well recognised[2].

We present the results of a 10-month-long quality improvement project that has improved adherence to a lung protective ventilation strategy on our unit. The project has also yielded a culture shift in the way that oxygen is prescribed and titrated for all our patients.

**Objectives**

1. Record the height of all critical care patients and calculate their ideal body weight (IBW).
2. Adopt a lung protective (6-8 ml/kg IBW) ventilation strategy for all patients receiving controlled ventilation.
3. Titrate oxygen delivery according to prescribed saturation targets (90 % or 95 %).

**Method**

Our project consisted of a series of tools to raise awareness, educational interventions, daily prompts and practical measures to promote adherence. In addition we adopted a pressure-control, volume-guarantee mode of ventilation as the initial standard for all patients ventilated on our unit. Our critical care technologists offered on-going practice education for members of the clinical team whilst a run-chart of week-by-week performance offered immediate feedback on the status of the project.

**Results**

**Fig. 88:**
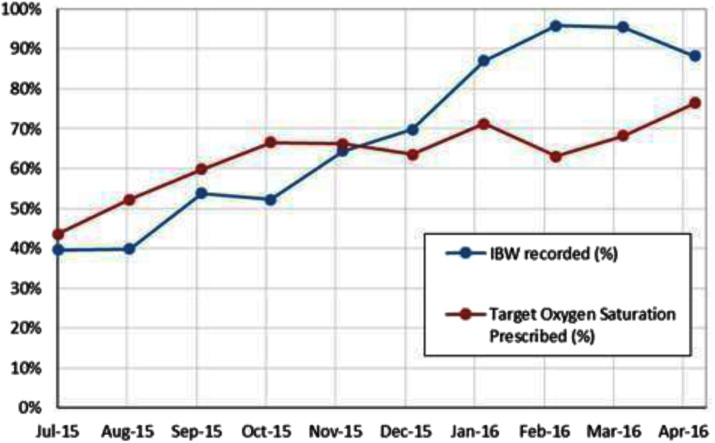
(abstract A203).

We have reviewed over 4200 patient-days of data in a rolling audit of the effectiveness of our project. Fig. 88 demonstrates the percentage of patients for whom an IBW was recorded and for whom a target oxygen saturation was prescribed each month.

Our latest results (April 2016) showed that 88 % (75/85) of patients on our unit had their IBW recorded and 100 % (14/14) of those receiving controlled ventilation had an appropriate tidal volume prescribed. Ventilation matched the prescription in 71 % (10/14) of cases. Routinely knowing the IBW of every patient on the unit has also been useful for the safe prescribing of haemofiltration and critical care drugs.

76 % (65/85) of patients had target oxygen saturations prescribed although only 38 % (25/65) were 'on target' at the time of data collection. Most importantly, prescribing an oxygen saturation target has empowered our nursing staff to wean inspired oxygen concentrations without waiting for an arterial blood gas.

**Conclusions**

Our project has demonstrated that a series of simple interventions can help to optimise mechanical ventilation and oxygen titration within critical care. There have been associated benefits in terms of safe prescribing and a reduction in our reliance on arterial blood gas analysis.

**References**

[1] Serpa Neto A. Association between use of lung-protective ventilation with lower tidal volumes and clinical outcomes among patients without acute respiratory distress syndrome. JAMA. 2012;308(16):1651-1659.

[2] Ridler N et al. Oxygen therapy in critical illness: friend or foe? JICS. 2014;15(3):190-196.

### A204 From zero to hero: a history of 10 years of quality improvement in VAP prevention

#### W. Swinnen, J. Pauwels, K. Mignolet, E. Pannier, A. Koch, T. Sarens, W. Temmerman

##### az Sint-Blasius, Dendermonde, Belgium

###### **Correspondence:** W. Swinnen – az Sint-Blasius, Dendermonde, Belgium

**Introduction**

VAP is an ICU-specific nosocomial infection, causing important additional costs in healthcare, extending ICU and hospital length of stay, with its own attributable mortality.

A sudden rise in VAP-rate in 2007 urged the ICU of the az Sint-Blasius to improve quality of care.

**Objectives**

To reduce VAP-rate by systematical Introduction of evidence based measures supported by PDCA-cycle Methodology.

**Method**

Process improvements included:

2008: tracheal suction protocol, prevention of colonization of ventilator tubing, HME change, oral care with hexetidin, cuff pressure control, 30° head elevation, ETT fixation, gastric residue control, ETT with polyurethane cuff, closed airway suctioning

2009: stop closed airway suctioning, ETT with PVC tapered shaped cuff and subglottic suctioning, digital continuous cuff pressure controller, automated subglottic suctioning pump

2011: oral care with chlorhexidine 0,2 % and oral care system permitting social control

2012: Belgian VAP-bundle, with continuous compliance measurement

2014: RASS to adjust the depth of sedation

**Results**

2008 measures were ineffective. Nurses identified ineffective tracheal suctioning by closed airway suctioning as a major problem. In 2009, stopping closed airway suctioning and the Introduction of 3 new technologies, reduced VAP-rate by half. In 2011, oral care with chlorhexidine 0,2 % and an oral care system permitting social control did not improve results. Although almost all VAP-bundle elements already were used in our ICU, the formal implementation of the Belgian VAP-bundle in 2012 caused an important additional decline in VAP-rate, emphasizing the importance of sedation control and compliance measurement.

In 2014, adjusting depth of sedation by RASS resulted in the lowest VAP-rate in 10 years.

**Conclusions**

This continuous quality improvement program shows that the systematic use of evidence based and PDCA-cycle driven process improvements may contribute to reduce VAP-rate.

**References**

1. Blot S, Poelaert J, Kollef M. How to avoid microaspiration? A key element for the prevention of ventilator-associated pneumonia in intubated ICU patients. BMC Infectious Diseases. 2014;14:119.

2. How to improve. Institute for Healthcare Improvement. http://www.ihi.org/resources/Pages/HowtoImprove/default.aspx Accessed 19/4/2016.

**Fig. 89 (abstract A204).**
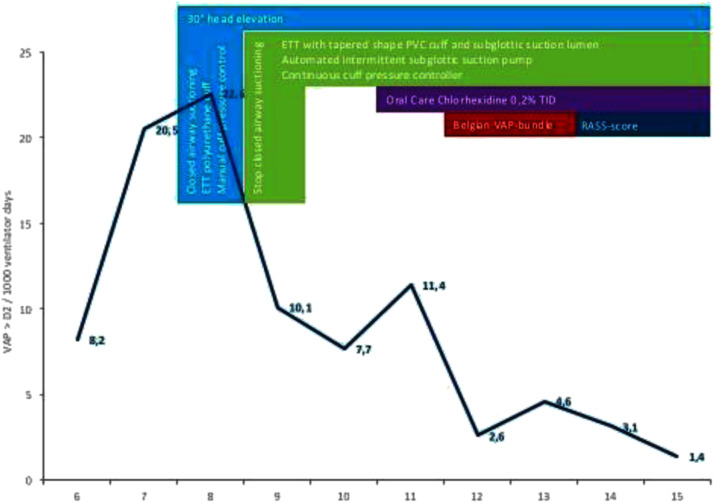
az Sint-Blasius VAP-rate

### A205 The impact of goal directed morbidity and mortality conferences on patient outcome, 3 year study

#### A.M. Elmenshawy, A.M. Fayed, M. Elboriuny, E. Hamdy, E. Zakaria

##### Alexandria University, Critical Care Medicine, Alexandria, Egypt

###### **Correspondence:** A.M. Elmenshawy – Alexandria University, Critical Care Medicine, Alexandria, Egypt

**Introduction**

Morbidity and mortality conferences (MMCs) are a traditional tool of improving local care management, and clinical management education especially in high risk specialty, but they lack a precise format for practice in intensive care units (ICU).

**Objectives**

To explore the impact of goal directed MMCs on mortality rate and adverse events.

**Method**

3 year prospective study in 2 ICUs (15 bed each) for evaluating a systematized MMCs with a clear goal of improving local care (how to avoid this) through case discussion, analysis, brainstorming and clear recommendations in a multidisciplinary meeting involving all related caregivers in a blame free environment. The present study included 4 phases; pre-intervention (Jan-12-April-13), intervention (May-July 2013), post-intervention (Aug-13-Oct-14), washout period (Nov-14-Dec-15). A period prevalence of ICU acquired adverse events were audited in March and December 2013.

**Results**

4589 patients were included in the study, from which 1456 died (31.7 %). During the intervention and post-intervention phases, 18 MMCs were held which discussed 36 died cases (28 from ICU3 and 8 from ICU1), reviewed other causes of death and unit performance indicators and made 96 recommendations (80 % accomplished in ICU3 versus 60 % in ICU1). The mortality rate decreased in post-intervention phase from 44.2 % to 25.1 % in ICU3 (p < 0.001) and from 21 % to 20.5 % in ICU1 (p = 0.748) then increased in washout period to 28.3 % in ICU3 (p = 0.189) and to 25.2 % in ICU1 (p = 0.041). Conversely, the mortality rate in washout period (in relation to pre-intervention phase) decreased in ICU3 (p < 0.001) but increased in ICU1(p = 0.036). Subanalysis in ICU1 mortality revealed significant decrease in early post-intervention (17.3 % with p = 0.031) and significant increase in late post-intervention (24.4 % with p = 0.028). Adverse events decreased insignificantly in December 2013 (as compared to April 2013); unplanned extubation from 71 to 48/1000 ventilator days ( 24 % to 15.5 %) in ICU3, unexpected cardiac arrest from 17.7 to 12.7/1000 patient days (8.6 % to 6.2 %) in ICU1, stress ulcer bleeding from 17 to 4.5/ 1000 patient days (12.6 % to 4.4 %) in ICU3 and 25 to 18/1000 patient days (12 % to 8.7 %) in ICU1, Deep vein thrombosis from 5.7 to 0/1000 patient days (4 % to 0 %) in ICU3 and from 12.6 to 5/1000 patient days (6 % to 2.5 %) in ICU1, iatrogenic pneumothorax from 5.7 to 0/1000 patient days (4 to 0 %) in ICU3 and from 5 to 7.6/1000 patient days (from 2.5 to 3.8 %) in ICU1. However, unexpected cardiac arrest (from 26.7 to 6.8/1000 patient days, p = 0.019) and VAP rate (from 10 to 2.5/1000 ventilator days, p = 0.002) in ICU3 and unplanned extubation (from 105 to 33/1000 ventilator days, p = 0.003) in ICU1 decreased significantly in Dec-13.

**Conclusions**

Although confounding factors were not controlled, goal oriented MMC reduced some adverse event and probably mortality rate especially with continuous monitoring and improvement.

**Fig. 90 (abstract A205).**
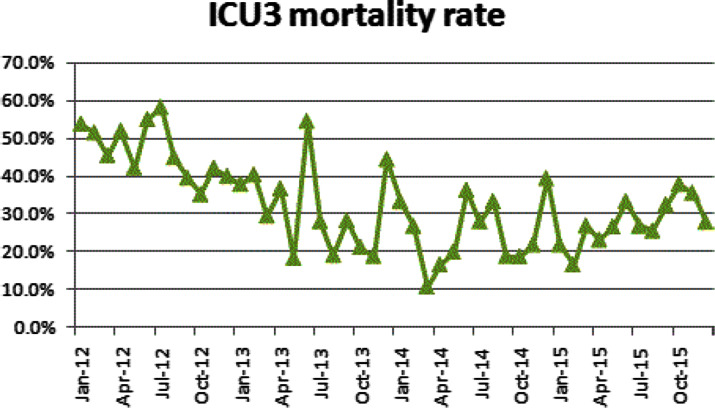
[ICU3 mortality]

**Fig. 91 (abstract A205).**
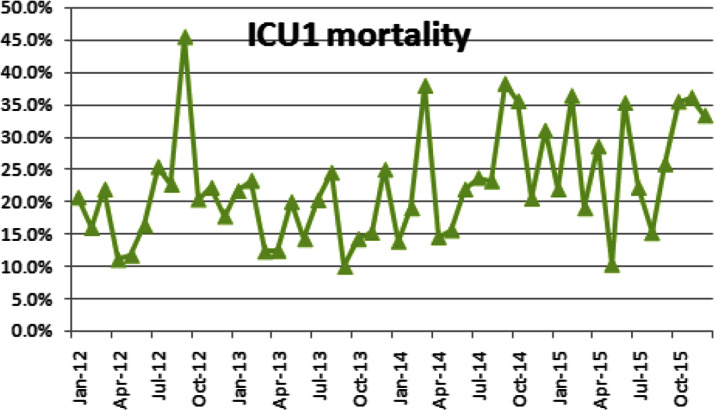
[ICU1 mortality]

### A206 Quality of nursing and care burdened measurement during intensive care in relation to patient outcome

#### A.-C. Falk

##### Karolinska University Hospital, CIVA, Stockholm, Sweden

**Introduction**

Intensive care is one of the most resource-intensive forms of medical care due to severely ill patients. During recent years the quality of intensive care has been in focus however there is still lacking result from nursing point of view.

**Objectives**

To describe nurse/patient ratio in relation to Care burdened measurement (VTS) and optimal medical and nursing-related result usually used indicators as mortality and complications during intensive care.

**Method**

This is a retrospective registry study includes a survey of critical care of registry data (all patients > 15 years) receiving care in two general Level I critical care units with similar rate of admissions during 2010-2014. Data of nurse/patient ratio is collected from each unit. The data is analyzed by descriptive and comparative statistical Method

**Results**

The result showed differences in specialized nurse/patient ratio of 0,5:1 to 1:1 ratio and Care burdened measurement (VTS) despite similarities in admission rate. Differences in cause of admission (surgicalv.s medical) and in the amount of unexpected surgery patients were found. Differences were also found in mean time on non-invasive ventilation and mean time on ventilator. Complications during critical care was measured by readmission and unplanned reintubation and showed that unplanned reintubation varied between 2.4-1.6 percent. ICU mortality showed differences with the lowest ICU mortality in the hospital with lower nurse/patient ratio. However, 30 days mortality was lower in the hospital with higher nurse/patient ratio. Further analysis is needed.

**Conclusions**

Preliminary results show differences in nurse/patient ratios and Care burdened measurement (VTS) with differences in and quality measurements in general critical care units.

**References**

1. Soini K, Stiernström H (red.Larsson & Rubertsson) (2005) *Intensivvård*. Liber. Torino, Italien

2. Institute of medicine (2001). *Crossing the quality chasm: a new health system for the 21 st century*. Washington DC: National Academy Press

3. Aiken L et al (2014). Nurse staffing and education and hospital mortality in nine European countries: a retrospective observational study. Lancet http://dx.doi.org/10.1016/s014-6736(13)62631-8

4. McGahan M, Kucharski G, Coyer F (2012). Nursing staffing levels and the incidence of mortality and morbidity in the adult intensive care unit: a literature review. *Australian critical care* 2012; 25,64-77

### A207 Adverse events measured by quality indicators in intensive care, an observational study

#### A. Petosic^1^, K. Olafsen^2^, H. Wøien^3^, H. Flaatten^4^, K. Sunde^2^

##### ^1^Oslo University Hospital, General Intensive Care Unit - Ullevål, Oslo, Norway; ^2^Oslo University Hospital, Department of Anaesthesiology, Oslo, Norway; ^3^Oslo University Hospital, General Intensive Care Unit 1 -Rikshospitalet, Oslo, Norway; ^4^Haukeland University Hospital, General Intensive Care Unit, Bergen, Norway

###### **Correspondence:** A. Petosic – Oslo University Hospital, General Intensive Care Unit - Ullevål, Oslo, Norway

**Introduction**

The use of quality indicators (QIs) for improvement of care in the intensive care unit (ICU) is increasing. Incidence of adverse events like ventilator associated pneumonia (VAP) and pressure ulcers are not routinely measured in norwegian ICUs.

**Objectives**

The aim of this pilot study was to evaluate the frequency of these two adverse events measured by specific QIs in two ICUs at the Oslo University Hospital Ullevål (OUHU).

**Method**

ICU-patients at two different ICUs at OUHU were included in the study in a predefined time period during Autumn 2015. Data for determining VAP and pressure ulcers were retrieved from the daily electronic patient record, journal and/ or bedside on a specific study sheet. Pneumonia (PN) was identified using the European Centre for Disease Prevention and Control (ECDC) protocol; -a combination of imaging, clinical and laboratory criteria. VAP was defined as a PN where the patient had been on mechanical ventilation for more than 48 hours and ventilator in place on the day of event or the day before. Pressure ulcers were identified using the European Pressure Ulcer Advisory Panel (EPUAP) classification system. To determine the severity of the pressure ulcer, four categories were used. Descriptive statistics are presented using SPSS version 21. Results are presented as mean ± standard deviation (SD).

**Results**

We included 58 adult ICU patients, all mechanically ventilated for a minimum of 48 hours, of whom 93 % were surgical patients. Among those, 69 % were trauma patients and 12 % acute surgery patients. Mean SAPSII score was 32,3 ± 14,1 and mean time on mechanical ventilation was 11,2 days ± 9,6. PN was present in 28 patients (48 %), and 19 (33 %) had per definition VAP. Per ECDC classification 19 of the 28 PNs were classified as PN1 with microbiology confirmation from Broncho-Alveolar Lavage (BAL), five PN2 with microbiology confirmation from endotracheal aspirates, and four PN5 without microbiology confirmation (clinical pneumonia). The incidence of VAP was 29/1000 ventilator days. Pressure ulcers were present in 18 (31 %) of the patients, and the majority of patients had more than one ulcer. The ulcers were classified as EPUAP category one (57 %), two (34 %), three (4 %) and four (1 %). The incidence of pressure ulcers was 70/ 1000 ICU days.

**Conclusions**

At OUHU there is a potential for reducing the incidence of VAP and pressure ulcers if we are to reach the current standard of care. Identification of specific QIs is important for future improving quality of care.

**Grant acknowledgment**

Oslo University Hospital.

### A208 Analysis of the perception and evaluation of the heathcare quality and the process of death: an empirical study from the perspective of families of deceased patients in an intensive care unit (ICU)

#### J.J. Cáceres Agra^1^, J.L. Santana Cabrera^1^, J.D. Martín Santana^2^, L. Melián Alzola^2^, H. Rodríguez Pérez^1^

##### ^1^Hospital Insular Las Palmas GC, Las Palmas de Gran Canaria, Spain; ^2^Hospital Insular Las Palmas GC, Department of Economy. University of Las Palmas de Gran Canaria, Las Palmas de Gran Canaria, Spain

###### **Correspondence:** H. Rodríguez Pérez – Hospital Insular Las Palmas GC, Las Palmas de Gran Canaria, Spain

**Introduction**

Care that takes the needs of families into account is very important, but in order to offer family-centered care, it is necessary to understand families experiences.

**Objectives**

To develop a valid and reliable tool to measure the perceived quality of care, the quality of the process of death and the satisfaction generated from the perspective of relatives of deceased patients in an ICU.

**Method**

Elaboration of an ad hoc questionnaire after literature review and validated by a multidisciplinary panel of experts. The construct of quality of service (QS) was measured by using three different constructs: quality of the communication (QS1), with two dimensions (“kindness and respect” and “sincerity and empathy”); quality of the information (QS2), with four dimensions (“welcome”, “information about disease”, “shared decission making” and “empathy with family needs”); technical and infraestructural quality (QS3), with six dimensions (“human aproach, “professionalism”, “waiting room”, “facilities”, “visiting hours” and “meals”). The construct about the process of death was measured by using three dimensions (“technical quality”, “human quality” and “quality of the information given”. Finally, the construct about overall satisfaction with the service was measured with three dimensions about satisfaction with “form and content of the information”, “technical and structural quality”, and “process of death”.

95 relatives of deceased patients participated with a post mail questionnaire (response rate: 38,9 %). After a month since the death, recruitment by telephone was carried out. Analysis of psychometric properties: convergent validity with correlation coefficient of Pearson, predictive validity with multiple regression models in order to predict the dimensions of satisfaction from the scales of quality, and reliability with α Cronbach and test of two halves.

**Results**

Nearly all of the correlation coefficients between the items of each construct were > 0,5, which meant, therefore, that all constructs had convergent validity. The three regression models performed to evaluate the predictive validity showed high determination coefficients (78,2 %. 94,9 % and 75,4 %). The evaluation of the items of all dimensions were high, with mean values around 6, except dimensions “empathy with family needs” and “waiting room”, with scores < 5.

**Conclusions**

The ad hoc tool developed was easy to use and showed adequate psychometric properties of validity and reliability which could be improved by removing some items that showed low correlations.

**References**

1. Van den Broek JM, Brunsveld-Reinders AH, Zedlitz AM, et al. Questionnaires on Family Satisfaction in the Adult ICU: A Systematic Review Including Psychometric Properties. Crit Care Med. 2015; 43(8):1731-44.

2. Wall RJ, Curtis JR, Cooke CR, et al. Family satisfaction in the between families of survivors and nonsurvivors. Chest. 2007; 132(5): 1425-33.

### A209 Reducing antibiotic resistance - watch out for protocols!

#### T. Castro Pires^1^, H. Calderón^2^, A. Pereira^3^, S. Castro^2^, C. Granja^1,2,4^

##### ^1^University of Algarve, Departmant of Biomedical Sciences and Medicine, Faro, Portugal; ^2^Centro Hospitalar do Algarve, Hospital de Faro, Emergency and Intensive Care Departmant, Faro, Portugal; ^3^Centro Hospitalar do Algarve, Hospital de Faro, Faro, Portugal; ^4^Faculty of Medicine of Porto, CINTESIS, Porto, Portugal

###### **Correspondence:** T. Castro Pires – University of Algarve, Departmant of Biomedical Sciences and Medicine, Faro, Portugal

**Objectives**

The purpose of this study was to evaluate the profile of antibiotic resistance and the effect of the Introduction of an antibiotic protocol in the ICU length of stay and mortality, costs and consumption of antibiotics.

**Method**

We conducted a retrospective study from a total of 476 patients admitted with in the ICU between January 2015 and December 2015. For the purpose of this study, and to obtain a profile of the microorganisms in our ICU, resistant microorganisms (RM) were defined as those with non-susceptibility to one (RM1) or more than one class of antibiotics (RM2).We also compared our results whit previous studies conducted in the ICU.

**Results**

Most prevalent microorganisms were*: Staphylococcus aureus*, *Escherichia coli*, *Pseudomonas aeruginosa* and *Klebsiella pneumonia*, 33 % of all microorganisms identified were resistant to more than one class of antibiotics (RM2) and 17 % of the patients had a RM1.Resistance was statistically significantly (p < 0,05) associated with:**Previous stay in wards -** in particular patients coming from surgical wards or Emergency;**Number of days from admission in hospital to ICU admission** - patients with RM2 had a mean hospital stay before ICU admission of 12 days;**Number of previous surgeries in the same hospital event** - RM2 were identified in patients with an average of two previous surgeries;**Admission type** - mainly patients coming from urgent surgery and medical causes;**Number of days in ICU** - patients with RM2 stay in average at least 5 days more in ICU than patients with microorganisms with no resistance;**Antibiotic treatment previous to ICU admission** (for at least 5 days before ICU admission), more than 30 % of patients with RM2 were submitted to antibiotic treatment before ICU admission.After the implementation of the antibiotic protocols we verified that the ICU length of stay was reduced in almost 3 days; the number of agents isolated increased 33 %; microbiological analyses increased 53 % which allowed us the practice of de-escalation consumption of antibiotics, by category, decreased by 82 % for the carbapenems, 33 % for antifungals and 35 % for antipseudomonal beta-lactams.There was a significant decrease in mortality, from 31,8 % to 26,8 %;There was a 22 % reduction in antibiotics consumption, with a total saving of 65,862€ in one year.

**Conclusions**

Resistance was associated with previous stay in wards, previous surgery and antibiotic treatment previous to surgery. The implementation of antibiotic protocols has had a positive impact as it was significantly associated with reduction on the length of ICU stay and ICU mortality, as well as consumption and costs associated with antibiotic therapy

**References**

1. Spellberg B. (2014). The future of antibiotics. Critical Care 18:228;

2. European Centre for Disease Prevention and Control. Antimicrobial resistance surveillance in Europe 2014. Annual Report of the European Antimicrobial Resistance Surveillance Network (EARS-Net). Stockholm: ECDC; 2015

### A210 Impact of preoperative health related quality of life on outcomes after cardiac surgery

#### I. Norkiene^1^, I. Urbanaviciute^2^, G. Kezyte^2^, D. Ringaitiene^1^, T. Jovaisa^3^

##### ^1^Vilnius University, Clinic of Anaesthesiology and Intensive care, Vilnius, Lithuania; ^2^Vilnius University, Faculty of Medicine, Vilnius, Lithuania; ^3^Lithuanian University of Health Sciences, Clinic of Anaesthesiology, Kaunas, Lithuania

###### **Correspondence:** I. Urbanaviciute – Vilnius University, Faculty of Medicine, Vilnius, Lithuania

**Introduction**

Advances in cardiac surgery techniques and postoperative care had led to decrease in major postoperative morbidity and mortality. Since operative risk declines, improvement of quality of life became an important patient centred outcome. It depends not only on physical but also on mental status and individual perception of health and rehabilitation.

**Objectives**

The primary aim of our study was to define the impact of preoperative patient characteristics and quality of preoperative life on Health Related Quality Of Life (HRQOL) dynamics one year after cardiac surgery. Secondary aim was to identify factors influencing long-term HRQOL postoperatively.

**Method**

A prospective cohort study in a tertiary referral university hospital. Study protocol was approved by institutional bioethics committee. The 36-item Short Form Health Survey (SF-36) was used to assess HRQOL amongst the study participants. An in-patient SF-36 questionnaire was completed a day before the elective surgery and repeated one year after surgery via telephonic interview. SF-36 is composed of 8 domains covering physical and mental aspects of health. Answers are converted to scale from 0 to 100 where higher values represent better health status. Effect Size Method was used to establish clinically significant change, therefore improvement was defined as positive change exceeding 1 Standard Deviation between baseline and follow up score in particular domain. Summary scores of Physical Component (PCS) and Mental Component (MCS) were used to identify patients with overall improvement in HRQOL. Based on these results study group was divided into improvers and non-improvers. Clinical data was collected from medical records. Independent samples and paired samples T-tests were used to compare baseline and follow-up data and differences between two groups.

**Results**

210 patients were enrolled in the study and underwent surgery in 2013-2014. After one year we were able to contact 53.8 % patients, hence final analysis included 105 patients. Overall positive significant change was identified in half of the domains, with significant improvement in PCS and MCS. Mean PCS at baseline was 42.5 (13.13-91.25) and 58.75 (23.13-92.5) at follow-up; MCS 52.75 (24.38-91) at baseline and 62 (24.5-99) at follow-up, p < 0,001 for both. There were 51 patients in non-improvers group and 54 patients in improvers group. No significant differences were identified between groups in demographic and peri-operative variables. Non-improvers had significantly higher preoperative scores in all domains including MCS (60.59 ± 14.28 vs 44.09 ± 12.09, p < 0,001) and PCS (54.36 ± 14.94 vs 37.93 ± 11.83 p < 0,001).

**Conclusions**

Our findings suggest that patients with worse HRQOL at baseline are more likely to experience long-term improvement following cardiac surgery. We did not identify any other factors influencing long-term HRQOL outcomes.

### A211 Health-related quality of life after surgical intensive care

#### G. Vogel^1^, U.-B. Johansson^1,2^, A. Sandgren^3^, C. Svensen^1^, E. Joelsson-Alm^1^

##### ^1^Karolinska Institutet, Department of Clinical Science and Education, Södersjukhuset, Stockholm, Sweden; ^2^Sophiahemmet University, Stockholm, Sweden; ^3^Linnaeus University, Kalmar/Växjö, Sweden

###### **Correspondence:** G. Vogel – Karolinska Institutet, Department of Clinical Science and Education, Södersjukhuset, Stockholm, Sweden

**Introduction**

Patients who have been treated at an ICU often have affected mental and physical health with complications such as depression, anxiety, posttraumatic stress disorder (PTSD) and, sleep disturbances[1-4]. Decreased health-related quality of life (HRQoL) after ICU stay have been shown in mixed ICU-populations but there is a lack of knowledge of HRQoL after surgical intensive care.

**Objectives**

The aim of this study was to describe HRQoL 3, 6 and 12 months after discharge from a general surgical ICU and to analyze factors associated with impaired HRQoL.

**Method**

Prospective cohort study in a general surgical ICU in Sweden. Included are patients with an ICU length of stay ≥ 96 hrs, < 18 years old, 2004-2012. HRQoL was measured with SF-36 at 3, 6 and 12 months after discharge from the ICU. Age, gender, APACHE II, LoS, mechanical ventilation, admission diagnosis, preexisting disease and marital status were recorded. Wilcoxon Signed Ranks Test was used for comparing HRQoL and changes over time. A general linear regression analysis was performed to analyze impact of background and ICU-related factors on HRQoL at 12-months, standardized to a general population in the same age in Sweden.

**Results**

A total of 276 (62 %) patients 18 - 89 yrs were included. HRQoL significantly improved between 3-12 months after ICU in six domains; PF (p = 0.00), RP (p = 0.00), GH (p = 0.03), VT (p = 0.00), SF (p = 0.00) and RE (p = 0.01), but were still lower in all eight domains compared to standard population. Age < 75 years was a risk factor.

**Conclusions**

Surgical intensive care patients have lower HRQoL compared to standard population one year after stay in the ICU. Age < 75 yrs is a risk factor for worse HRQoL. This study also contributes to the knowledge of risk factors for impaired HRQoL which can be important when considering selection of patients to the ICU follow-up clinics.

**References**

1. Jackson JC, Pandharipande PP, Girard TD, et al. Depression, post-traumatic stress disorder, and functional disability in survivors of critical illness in the BRAIN-ICU study: a longitudinal cohort study. *The Lancet Respiratory medicine* 2014; **2**(5): 369-79.

2. Deja M, Denke C, Weber-Carstens S, et al. Social support during intensive care unit stay might improve mental impairment and consequently health-related quality of life in survivors of severe acute respiratory distress syndrome. *Critical care (London, England)* 2006; **10**(5): R147.

3. Jones C, Backman C, Capuzzo M, Flaatten H, Rylander C, Griffiths RD. Precipitants of post-traumatic stress disorder following intensive care: a hypothesis generating study of diversity in care. *Intensive care medicine* 2007; **33**(6): 978-85.

4. Parsons EC, Hough CL, Vitiello MV, Zatzick D, Davydow DS. Insomnia is associated with quality of life impairment in medical-surgical intensive care unit survivors. *Heart & lung: the journal of critical care* 2015; **44**(2): 89-94.

### A212 Pulmonary function and quality of life in a Brazilian post-ICU follow-up outpatient clinic

#### M.A. Leite, L.D. Murbach, E.F. Osaku, C.R.L.M. Costa, M. Pelenz, N.M. Neitzke, M.M. Moraes, J.L. Jaskowiak, M.M.M. Silva, R.S. Zaponi, L.R.L. Abentroth, S.M. Ogasawara, A.C. Jorge, P.A.D. Duarte

##### Western Parana State University Hospital, Cascavel, Brazil

###### **Correspondence:** M.A. Leite – Western Parana State University Hospital, Cascavel, Brazil

**Introduction**

Critical illness survivors have physical impairment, reduced functional capacity and change in quality of life after discharge from the Intensive Care Unit (ICU)**.**

**Objectives**

To evaluate pulmonary function and quality of life of critically ill patients three months after discharge from the ICU.

**Method**

Cohort study conducted in the Post-ICU Follow-Up Outpatient Clinic of an University Hospital, Southern Brazil, from April 2012 to June 2013. It was performed spirometry test and the patients answered SF-36 after three months of discharge from ICU. The patients were divided into 3 groups: normal spirometry (G1), restrictive (G2) and obstructive (G3). The variables that were in accordance with the assumptions of normality and homoscedasticity were evaluated by analysis of variance (ANOVA single-factor) and those that were not in accordance with the assumptions were evaluated by Kruskal-Wallis test.

**Results**

609 patients were admitted, 440 were discharged alive from the hospital. In the Outpatient Clinic, 126 patients were evaluated, 61 % male, age 44 ± 18.19 years, 48 % were smokers and 8 % COPD. The main causes of admission were postoperative elective surgery (26 %) and trauma with head injury (20 %), mean APACHE II 19.08, ICU length of time 7.23 days, hospital length of time 17.75 days, MV > 24 hours 47.62 %, and mean MV time 86.45 hours. The groups showed significant differences in spirometric data, shown in Table 70. In the SF-36 only the areas vitality and social aspects showed statistical difference (Table 71).

**Conclusions**

Most of the patients had normal spirometry. The main pulmonary impairment was obstructive feature. Some areas of quality of life after ICU discharge present relationship with worsening of lung function.

**Table 70 (abstract A212).** Spirometry Results

Spirometric variables	Normal (n=79)	Restrictive (n=10)	Obstructive (n=35)	p
FEV1	2.98 + 0.81	2.21 + 0.67	2.24 + 1.01	0.00002
PEF	375.84 + 128.92	321.79 + 130.04	282.37 + 139.70	0.001
FVC	3.60 + 1.01	2.48 + 0.69	3.48 + 1.36	0.001

**Table 71 (abstract A212).** Results of SF-36

SF-36	Normal (n=79)	Restrictive (n=10)	Obstructive (n=35)	p
Functional Capacity	65.57 ± 110.34	47.86 ± 27.99	33.50 ± 30.32	0.09
Physical Limitations	23.73 ± 35.33	25.00 ± 34.26	22.50 ± 36.88	0.91
Pain	34.53 ± 38.81	24,17 ± 49.93	42.20 ± 33.10	0.23
General Health	68.51 ± 24.07	62.57 ± 25.76	62.40 ± 25.84	0.20
Vitality	68.10 ± 25.87	58.00 ± 27.11	57.50 ± 26.46	0.04*
Social Aspects	72.66 ± 34.12	64.40 ± 33.92	46.40 ± 35.21	0.04*
Emotional Aspects	35.43 ± 40.81	38.11 ± 47.14	33.30 ± 45.14	0.96
Mental Health	67.44 ± 26.75	65.14 ± 26.94	65.20 ± 30.49	0.80

### A213 Quality of life of critical patients three months after ICU discharge

#### L.D. Murbach, M.A. Leite, E.F. Osaku, J. Barreto, S.T. Duarte, S. Taba, D. Miglioranza, D.P. Gund, C.F. Lordani, C.R.L.M. Costa, S.M. Ogasawara, A.C. Jorge, P.A.D. Duarte

##### Western Parana State University Hospital, Cascavel, Brazil

###### **Correspondence:** L.D. Murbach – Western Parana State University Hospital, Cascavel, Brazil

**Introduction**

The evolution of care to critically ill patients has provided a considerable number of survivors after admission to the ICU, but several patients have impaired quality of life owing to changes in physical, functional, social and emotional disorders that often culminate in a prolonged recovery. In low-income countries this picture is poorly described.

**Objective**

To evaluate health-related quality of life in patients that survived to ICU stay three months after ICU discharge.

**Method**

Prospective cohort study conducted in the period 2012-2014, at the Post-ICU Multiprofessional Follow-up outpatient Clinic of a teaching hospital, in Southern Brazil. Included patients > 24 hours in the ICU, ≥ 18 years old and who attended the outpatient evaluation. To evaluate the quality of life it was applied the questionnaire SF-36.

**Results**

In the 2-year period, 275 patients were evaluated: 63.6 % male, mean age 44.0 years. Trauma was the leading cause of hospitalization (37.1 %). 45.5 % had comorbidities: hypertension (43.7 %), diabetes (32.4 %) and alcohol / tobacco (18.7 %). Mean ICU length of stay was 9.9 days; 74.9 % remained more than 48 hours with mechanical ventilation; 63.6 % were sedated and 25.8 % tracheostomy. Mean APACHE II 20.2.There was significant impairment in relation to the limitations of physical and emotional aspects. The main factors associated with change in QOL were: Physical Component: Mechanical Ventilation, Sedation and the presence of post-ICU sequelae; Mental Component: Admission by neuro-trauma and the presence of post-ICU sequelae.

**Conclusion**

There was impairment in quality of life related to the limitations of physical, social and emotional aspects, justifying the need for monitoring and rehabilitation after discharge, mainly the patients victim of neurological trauma and underwent sedation and prolonged MV.

### A214 Quality of care in the intensive care unit: more than just survival statistics. Measurement of family satisfaction using the FS(ICU) 24 questionnaire in a single UK centre

#### H. Vollmer, M. Gager, C. Waldmann

##### Royal Berkshire Hospital, Intensive Care Unit, Reading, United Kingdom

###### **Correspondence:** H. Vollmer – Royal Berkshire Hospital, Intensive Care Unit, Reading, United Kingdom

**Introduction**

Patient experience is a key marker of healthcare quality and is a focus of the Department of Health. 1 in 5 critically ill patients die, those that survive often have little memory of their intensive care stay. Family members are a valuable proxy for patient experience in ICU. The Family Satisfaction in the Intensive Care Unit FS(ICU) 24 questionnaire was developed in Canada and is widely validated. It has 2 domains: Satisfaction with Care and Satisfaction with Decision Making[1]. The UK FREE study adapted the questionnaire and found it to have high internal consistency and criterion validity, but that the free text responses demonstrated scope for improvement [2].

**Objectives**

To assess levels of satisfaction in family members of ICU patients to guide improvement in quality of care.

**Method**

213 patients were admitted between 01/10/15 and 01/01/16. 210 FS(ICU)-24 questionnaires were sent to their next of kin in the month after death or ICU discharge. 19 questions were analysed using the 5-point Likert scale.

**Results**

96 questionnaires were returned (45.7 %). 62.5 % of responders were female; the mean age was 60.4 (±15.7) yrs. 25 % of relatives had previous ICU experience and 71.6 % lived with the patient prior to their illness. 66 % were the patient's partner.

Overall satisfaction was 86.1. Satisfaction with Care score was 87.6 The highest score was in Concern and Caring (95.3) and the lowest in Waiting Room Atmosphere (67.4). Satisfaction with Decision Making scored 82.8. The highest score was in Honesty of Information (85.7); the lowest score of 76.9 was in Frequency of Communication with Doctors.

11 % of relatives felt they could have used more time when making decisions. 77 % felt they had been supported through the decision-making process; 6 % felt overwhelmed. 8 % felt out of control over the care of the patient, although this was not always negative - *'leaving it to the experts'*. The free text responses were insightful and a valuable contribution to the data.

**Conclusions**

The low response rate and the potential for response bias due to the most dissatisfied relatives declining to respond limit this study. Its strengths are that it provides individualised feedback which can be used to enhance quality of care locally. Future quality improvement projects will target improving communication and include disseminating a study summary to staff members to highlight the importance of communication, more information in the waiting room on expectations in the intensive care unit and the use of family ward rounds.

**References**

1) Wall RJ, Engelberg RA, Downey L et al. Refinement, scoring, and validation of the Family Satisfaction in the Intensive Care Unit (FS-ICU) survey. Crit Care Med 2007;35(1):271-9.

2) Wright SE, Walmsley E, Harvey SE et al. Family-Reported Experiences Evaluation study: a mixed-Method study to evaluate families' satisfaction with adult critical care services in the NHS. Health Services and Delivery Research 2015;3(45):ISSN 2050-4349

**Fig. 92 (abstract A214).**
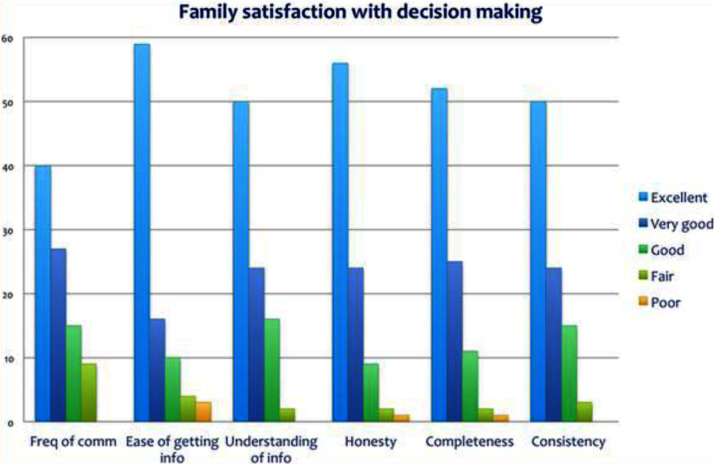
Family satisfaction with decision making

**Fig. 93 (abstract A214).**
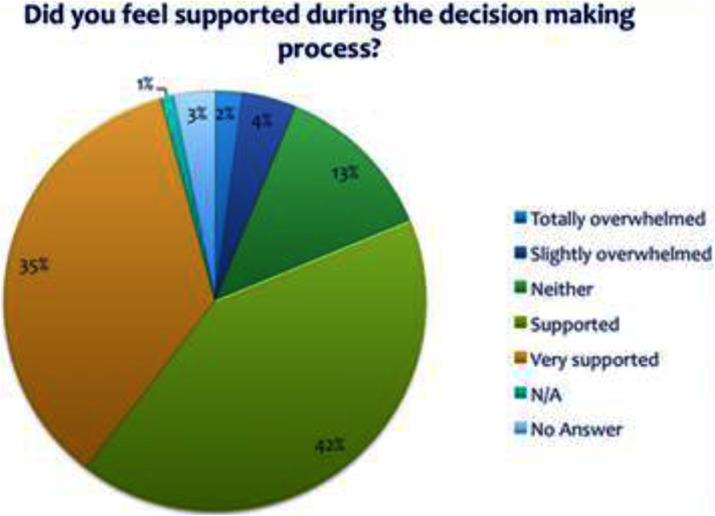
Support during the decision making process

**Fig. 94 (abstract A214).**
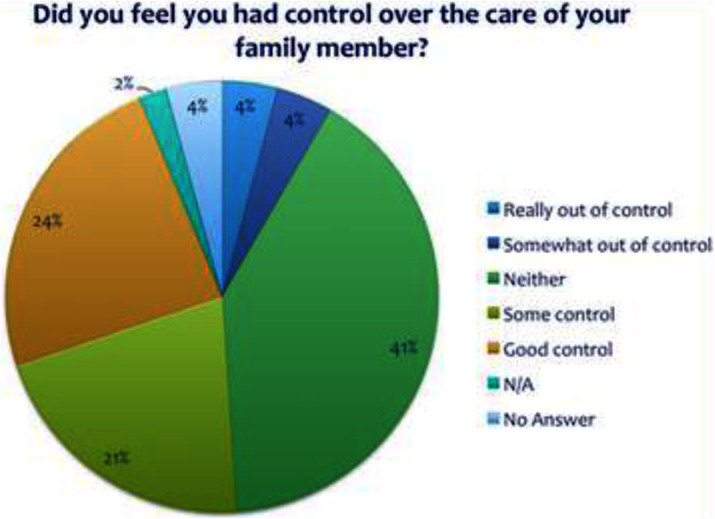
Control over the care of your family member

### A214 A multivariate projection method to investigate inflammatory mediator profiles in the early phase of critical illness

#### A.T. Mazzeo^1^, R. Tesio^1^, C. Filippini^1^, M.E. Vallero^1^, C. Giolitti^1^, S. Caccia^1^, M. Medugno^1^, T. Tenaglia^1^, R. Rosato^2^, I. Mastromauro^1^, L. Brazzi^1^, P.P. Terragni^1^, R. Urbino^1^, V. Fanelli^1^, V.M. Ranieri^3^, L. Mascia^4^

##### ^1^University of Turin, Anesthesia and Intensive Care, Turin, Italy; ^2^University of Turin, Psychology, Torino, Italy; ^3^Sapienza University of Rome, Anesthesia and Intensive Care, Rome, Italy; ^4^Sapienza University of Rome, Scienze e Biotecnologie Medico Chirurgiche, Rome, Italy

###### **Correspondence:** A.T. Mazzeo – University of Turin, Anesthesia and Intensive Care, Turin, Italy

**Introduction**

Critical illness of different etiology triggers an inflammatory cascade associated with organ dysfunction. However it is not known if the inflammatory pattern is disease specific or related to the severity of injury. We hypothesized that in critical ill patients of different etiology a specific pattern of cytokines measured in the early phase after ICU admission may predict mortality.

**Objectives**

Aim of this study was to investigate the early inflammatory profile in critical ill patients and its association with ICU mortality.

**Method**

Local Ethic Committee approved the study protocol. Critical ill patients admitted to ICU for sepsis-associated acute respiratory distress syndrome (ARDS), severe traumatic brain injury (TBI) and subarachnoid hemorrhage (SAH) were studied. Demographic data, severity indexes at admission and physiological variables were recorded. Blood samples for cytokines analysis were collected at days 1, 2 and 4 after admission. The cytokine analysis was performed with Bioplex technology and 27 cytokines were analysed. Statistical analysis: Multivariate projection technique was applied to analyse variation and collinearity within the cytokines dataset without a priori selecting potential relevant molecules. Principal component analysis (PCA) was used to identify principal components (PC) which account for the majority of the variation within the dataset.

**Results**

Eighty-six critical ill patients admitted for sepsis-associated ARDS (n = 36), severe TBI (n = 29), SAH (n = 21) were studied. Mean age was 52.8 ± 18, APACHE II 17.6 ± 2.9, SAPS II 44.9 ± 11.7, mean SOFA in the first week 8.3 ± 2.9; median ICU-length of stay was 19 days (range 14-31), median Hospital-LOS was 34 days (range 17-50), ICU mortality 31.7 %. Using PCA, the first five PCs generated by the model explained 65 % of the variation within the dataset. The first component is strongly correlated with the following cytokines - IL4, IL12, IL10, IL-1ra, FGF basic, IL13, GCSF, IL-1β, IL7, TNFα, IL5, MIP-1α, INFγ, IL8, IL2, IL17, IL6. Moreover the second component is strongly correlated with VEGF, IP-10, IL9. After correcting for age, ApacheII, SAPS and SOFA, the two principal components PC1 and PC2 were evaluated as predictor of ICU mortality with OR 1.0737 (C.I. 0.8897; 1.2956) and OR = 1.5204 (C.I. 1.0962; 2.1086), respectively.

**Conclusions**

In critical ill patients of different etiology the multivariate projection Method represents a valuable technique to identify inflammatory patterns as predictor of outcome.

**References**

Mickiewicz B et al:Integration of metabolic and inflammatory mediator profiles as a potential prognostic approach for septic shock in the intensive care unit. Critical Care 2015;19:11

Helmy A et al:Recombinant human IL-I receptor antagonist promotes MI microglia biased cytokines and chemokines following human traumatic brain injury.JCBFM 2015;Dec1

**Grant acknowledgment**

University of Torino MAZA_RILO_1601

## CONTEMPORARY ISSUES IN ARF

### 0215 Inappropriate coding of chronic severe respiratory disease in a general adult intensive care unit

#### J. Ballantyne, L. Paton, A. Mackay

##### Queen Elizabeth University Hospital, NHS GG&C, Intensive Care Medicine, Glasgow, United Kingdom

###### **Correspondence:** J. Ballantyne – Queen Elizabeth University Hospital, NHS GG&C, Intensive Care Medicine, Glasgow, United Kingdom

**Introduction**

High quality clinical databases facilitate repeated evaluation of clinical practice and comparative audit. [1] However, inaccurate data entry can lead to inaccurate conclusions.

**Objectives**

We sought to determine whether patients in our intensive care unit (ICU) were appropriately coded as having chronic severe respiratory disease.

**Method**

We conducted a retrospective case note review of patients admitted to a general adult ICU between January *2009* and December *2014* and labelled with chronic severe respiratory disease. Severe respiratory disease was defined as “chronic restrictive, obstructive or vascular disease resulting in severe exercise restriction or documented chronic hypoxia, hypercapnia, secondary polycythaemia, severe pulmonary hypertension or respirator dependence”. Patients were identified from the WardWatcher™ database, electronic records interrogated and statistical significance assessed using the Paired T-test and the Chi Squared test.

**Results**

There were 112 ICU patients (48 men, 43 %) labelled with chronic severe respiratory disease over this six year period. These patients had a median age of 62 years (IQR 54-70 years) and median APACHE II score of 20 (IQR 16-28). Electronic records were insufficient to confirm or refute the label of chronic severe respiratory disease in 13 cases and these patients were excluded from analyses. Forty-two of the remaining 99 patients (42 %) did not have chronic severe respiratory disease by the standardised definition. There was no significant difference in APACHE-II, ICU mortality, ICU length of stay, hospital mortality or hospital stay. The standardised mortality ratio was 0.752 (95 % CI 0.748-0.756) for appropriately coded patients and 0.855 (95 % CI 0.848-0.862) for inappropriately coded patients.

**Conclusions**

Almost half of patients are wrongly coded as having chronic severe respiratory disease. Moreover, this clearly impacts the SMR for this group of patients. Such errors in data entry compromise conclusions drawn from this cohort and comparisons made with prior cohorts and other ICUs. In order to improve this resource, stringent documentation by clinicians and education of those inputting the data is required.

**References**

1. Harrision D, Brady A, Rowan K. Case mix, outcomes and length of stay for admissions to adult, general critical care units in England, Wales and Northern Ireland. *Critical care*. 2004; 8:R99-R111

### A216 Prospective validation of right ventricular role in primary graft dysfunction after lung transplantation

#### P. Perez-Teran^1^, O. Roca^2^, J.C. Ruiz-Rodriguez^2^, A. Zapatero^1^, J. Serra^2^, J.R. Masclans^1^

##### ^1^Hospital del Mar/CIBERES/UPF, Intensive Care, Barcelona, Spain; ^2^Vall d´Hebrón University Hospital, Intensive Care, Barcelona, Spain

###### **Correspondence:** P. Perez-Teran – Hospital del Mar/CIBERES/UPF, Intensive Care, Barcelona, Spain

**Introduction**

Primary graft dysfunction is a significant cause of lung transplant morbidity and mortality, but its underlying mechanisms are not completely understood.

**Objectives**

Aims of the study: 1) to confirm that right ventricular function is a risk factor for severe primary graft dysfunction; 2) to propose a clinical model for predicting the development of severe primary graft dysfunction.

**Method**

A prospective cohort study was performed over 14 months. The primary outcome was development of primary graft dysfunction grade 3. An echocardiogram was performed immediately before transplantation, measuring conventional and speckle-tracking parameters. Pulmonary artery catheter data were also measured. A classification and a regression tree were made to identify prognostic models for the development of severe graft dysfunction.

**Results**

Seventy lung transplant recipients were included. Patients who developed severe primary graft dysfunction had better right ventricular function, as estimated by cardiac index (3.5 ± 0.8 vs. 2.6 ± 0.7 l/min*m^2^; p < 0.01) and basal longitudinal strain (-25.7 ± 7.3 vs. -19.5 ± 6.6 %; p < 0.01). Regression tree analysis provided an algorithm based on the combined use of three variables (Basal longitudinal strain, pulmonary fibrosis disease and ischemia time), allowing accurate preoperative discrimination of three distinct subgroups with low (11 to 20 %), intermediate (54 %) and high (75 %) risk of severe primary graft dysfunction (AUROC 0.81).

**Conclusions**

Better right ventricular function is a risk factor for the development of severe primary graft dysfunction. Preoperative estimation of right ventricular function could allow early identification of recipients at increased risk, who would benefit the most from careful perioperative management in order to limit pulmonary overflow.

**References**

1. Pérez-Terán P, et al. Influence of right ventricular function on the development of primary graft dysfunction after lung transplantation. *J Hear Lung Transplant* 2015.

2. Liu Y, et al. Recipient-related clinical risk factors for primary graft dysfunction after lung transplantation: a systematic review and meta-analysis. *PLoS One* 2014;9(3).

3. Champion HC, et al. Comprehensive invasive and noninvasive approach to the right ventricle-pulmonary circulation unit: state of the art and clinical and research implications. *Circulation* 2009;120(11):992-1007.

4. Wrobel JP, et al. Preoperative echocardiographic-defined moderate-severe pulmonary hypertension predicts prolonged duration of mechanical ventilation following lung transplantation for patients with COPD. *Lung* 2012;190(6):635-643.

**Grant acknowledgment**

Dr. Pérez-Terán is the recipient of a *“Rio Hortega”* grant (Ref. CM12/00216) from the Instituto de Salud Carlos III, Ministerio de Ciencia e Innovación.

### A217 Gas exchanges during whole lung lavage

#### S. Bianzina, P. Cornara, G. Rodi, G. Tavazzi, M. Pozzi, G.A. Iotti, F. Mojoli, A. Braschi

##### Anesthesia and Intensive Care, Fondazione IRCCS Policlinico S. Matteo, University of Pavia, Pavia, Italy

###### **Correspondence:** S. Bianzina – Anesthesia and Intensive Care, Fondazione IRCCS Policlinico S. Matteo, University of Pavia, Pavia, Italy

**Introduction**

Pulmonary alveolar proteinosis (PAP) is a rare disorder characterized by a perturbation in surfactant homeostasis, resulting in its accumulation within alveolar spaces, with a consequent development of severe hypoxemia.

Whole lung lavage (WLL) is a complex procedure, dedicated to those patients affected by a severe condition not responsive to medical treatment[1].

**Objective**

To analyze the evolution of gas exchanges during WLL, evaluating PaO_2_ variations during the different phases of the procedure.

**Patients and method**

We enrolled 27 patients with PAP (16 males, age 15-64 years), who underwent WLL in our ICU between 2010 and 2015.

WLL was conducted in general anesthesia, using selective endotracheal tubes. It consisted of different phases, for each lung: 1) bipulmonary mechanical ventilation in supine position at FiO_2_ 1 and ZEEP; 2) bipulmonary ventilation in supine position with PEEP; 3) monopulmonary ventilation in supine position, in order to achieve complete atelectasis of the contralateral lung; 4) monopulmonary ventilation in lateral position, with the ventilated lung downward; 5) lavage of the whole atelectatic lung with liquid tidal ventilation at different levels of hydrostatic lavage pressure.

We collected data regarding patient gas exchanges by performing several blood gas analysis during the different steps of WLL. We expressed data as mean ± standard deviation (SD).

**Results**

**Fig. 95:**
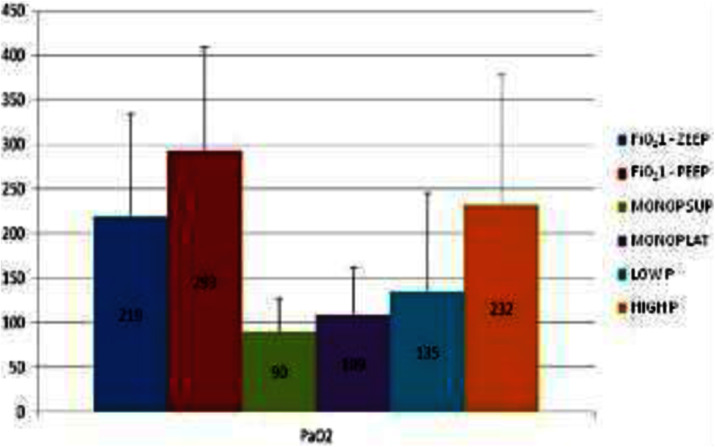
(abstract A217).

Figure 95 shows the mean values of PaO_2_ during the different phases of WLL. During bipulmonary ventilation gas exchanges improved in response to FiO_2_ 1 and PEEP. Monopulmonary ventilation, instead, induced a clear reduction of PaO_2_, which increased in lateral position and during liquid tidal ventilation, with a substantial effect of elevated hydrostatic lavage pressures. The wide SD indicates an uneven response of gas exchanges in the studied population.

**Conclusions**

The pathophysiology of PAP is characterized by altered alveolo-capillary diffusion and intrapulmonary shunt, thus significantly responsive to FiO_2_ and PEEP, respectively. During monopulmonary ventilation, shunt is reduced by the lateral position, which provides a better perfusion of the ventilated lung, and the elevated hydrostatic lavage pressures, which are able to limit perfusion in the contralateral lung under WLL.

**Reference**

1. Pulmonary alveolar proteinosis: diagnostic and therapeutic challenges. Campo et al. Multidisciplinary Respiratory Medicine 2012, 7:4.

### A218 Study of clinico-epidemiological profile of patients during swine flu epidemic in 2015 at a tertiary care hospital in new Delhi

#### A. Vishnu, D. Buche, R. Pande

##### Blkapoor Super Speciality Hospital, BLK Centre for Critical Care, New Delhi, India

###### **Correspondence:** A. Vishnu – Blkapoor Super Speciality Hospital, BLK Centre for Critical Care, New Delhi, India

**Introduction** WHO reported that influenza A(H1N1) pandemic in 2009 caused 4100 deaths globally[1]. New Delhi reported 2241 cases in 2015 with 8 confirmed deaths till March 2015 [2]. The mortality in severe refractory ARDS related to H1N1 influenza A pneumonia is high [3]. During 2015 swine flu epidemic our hospital admitted 162 adult patients who were either suspected or confirmed cases.

**Objectives** To study the epidemiological and clinical profile of patients admitted to BLK Superspeciality Hospital, New Delhi during 2015 H1N1 influenza epidemic.

**Method** Data was collected retrospectively from hospital records after IRB approval, for patients admitted either as a suspected or proven case (outside) of Influenza A (H1N1) pneumonia. Demographical details, category of presentation, hospital and ICU course and ventilation strategies applied were collected.18 patients were excluded as significant data was missing from records.

**Results** We analysed 144 patients out of which, 66 % (n = 96) were from an urban background and 33 % (n = 48) were from a rural background. 56.9 % (n = 82) patients were H1N1 positive by RT-PCR technique. 24.4 % (n = 20) had a positive history of contact with established H1N1 cases, whereas history of travel to affected areas was present in 54.6 % (n = 45) patients. Maximum admissions were reported in the month of February (n = 47) and March( n = 28). 21.8 % patients ( n = 21 ) were belonging to age group 30 - 35 years and > 60 years age group.15.85 % (n = 13) of all the positive patients were of Category C, who were admitted to ICU directly.

71.6 % (n = 58) were managed with non invasive ventilatory support only, and 14.6 % (n = 12) were intubated and mechanically ventilated. 10 out of 12 patients requiring intubation and mechanical ventilation belonged to Category C. 75 % (n = 9) of intubated patients had refractory hypoxemia (mean PaO2/FIO2 ratio 61.33 ± 4.05), and 6 patients required early prone ventilation. 8.5 % (n = 7) patients died and all of them were mechanically ventilated. 89 % (n = 73) were successfully discharged from the hospital. Mean ICU and hospital LOS was 3.83 ± 4.06 days and 7.53 ± 4.2 days respectively. Higher mortality rates was also seen in those patients who had more than 3 days of time lag for initiation of oseltamivir after the symptom onset (n = 5).

**Conclusions** Influenza A H1N1 pneumonia is a significant burden during epidemics associated with a high morbidity. The mortality among severe refractory ARDS patients who were invasively ventilated is very high.

**References**

1. MOHFW, Press information Bureau Release, March 2015

2. CDC guidelines, 2009

3. Domínguez-Cherit G1, Lapinsky SE, Macias A et al. Critically Ill patients with 2009 influenza A(H1N1) in Mexico. JAMA. 2009 Nov 4;302(17):1880-7.

### A219 Structural and organizational factors influencing mortality of severe community-acquired pneumonia patients in the intensive care

#### D.L.J. Moolenaar^1^, F. Bakhshi-Raiez^2,3^, D.A. Dongelmans^2,3^, N.F. de Keizer^2,3^, D.W. de Lange^1,3^

##### ^1^University Medical Center Utrecht, Intensive Care, Utrecht, Netherlands; ^2^Academic Medical Center, University of Amsterdam, Intensive Care, Amsterdam, Netherlands; ^3^Dutch National Intensive Care Evaluation Foundation, Amsterdam, Netherlands

###### **Correspondence:** D.L.J. Moolenaar – University Medical Center Utrecht, Intensive Care, Utrecht, Netherlands

**Introduction**

Severe community-acquired pneumonia patients bear the highest morbidity, in-hospital mortality, and cost of all patients with community acquired pneumonia[1]. The appropriate management of these patients has received close attention in the current era of rising health care costs. Nevertheless, the outcome of these patients remains poor[2] and it is unknown which patient characteristics or treatment modalities are associated with a better outcome.

By combining a national registry with a specifically designed questionnaire we were able to examine the factors related to organizational characteristics and treatment policies that might explain the variation in mortality outcomes in sCAP patients in the ICU.

**Method**

This study used a dataset from a national registry containing data on patient and ICU level combined with a web-based survey on treatment policies. The relationship between in-hospital mortality and determinants was analyzed using multivariable logistic regression analysis.

**Results**

From January 1st , 2008 to January 1st 2013, 79 Dutch ICUs provided data to the registry, from which 62 (78.5 %) returned the questionnaire. The responding ICUs admitted 11,245 patients with severe community-acquired pneumonia. These patients had a higher severity of illness than the general medical ICU population and their mortality was also higher (ICU mortality 17.8 %; hospital mortality 25.1 % versus ICU mortality 15.8 %; hospital mortality 22.5 %, respectively). Severe community-acquired pneumonia patients had more co-morbidities and stayed longer at the ICU. Rare use of tracheostomies for the weaning process (OR:0.784(0.662-0.928)) was associated with better outcomes in these patients. The need for mechanical ventilation (OR:1.385(1.194-1.607)) was associated with poor outcomes as was the case for the mean number of ICU admissions per year (OR: 1.006 ( 1.001-1.010).

**Conclusions**

CAP is a disease with a high in-hospital mortality. We have shown that, after correction for confounding factors some treatment and organizational factors are related with outcome in patients with sCAP. The need for mechanical ventilation remained an independent risk factor for mortality, and liberal use of tracheostomies for the weaning process was also associated with higher mortality. The mean number of ICU admissions with sCAP per year was inversely correlated with hospital mortality. Many other parameters that are often claimed to be associated with better or worse outcome (like differences in antibiotic therapy) did not correlate with outcome.

**References**

1) Mandell, L. A. et al. 2007. Infectious Diseases Society of America/American Thoracic Society consensus guidelines on the management of community-acquired pneumonia in adults. Clin.Infect.Dis. 44 Suppl 2:S27-S72.

2) Brinkman, S. et al. Mortality after hospital discharge in ICU patients. Crit Care Med. 41[5], 1229-1236. 2013.

**Grant acknowledgment**

None.

### A220 Status asthma in intensive care unit. Experience of a third level center

#### I. Fuentes Fernández, D. Martínez Baño, J.L. Buendía Moreno, R. Jara Rubio

##### Hospital Virgen de la Arrixaca, ICU, El Palmar, Spain

###### **Correspondence:** I. Fuentes Fernández – Hospital Virgen de la Arrixaca, ICU, El Palmar, Spain

**Background and objectives**

Severe asthmatic patient who requires the use of invasive mechanical ventilation in an intensive care unit is, despite its low incidence, a potentially very serious case, which requires early and very specific respiratory care. This care will largely determine the average ICU stay, occurrence of secondary injuries or even death. Our objective is to describe the characteristics of severe asthmatic patient admitted to ICU and to analyze the initial treatment.

**Method**

Descriptive study of patients admitted to the ICU of the Clinical Hospital Virgin of Arrixaca in the period between January 2011 and September 2015. We analyzed different variables, age, sex, APACHE II, SAPS II, parameters of mechanical ventilation, ICU stay and laboratory parameters.

**Results**

18 patients (6 women) admitted to the ICU with a diagnosis of severe asthma. The average age was 35.06. The average stay in the ICU was 14.39 ± 25.55 days, with 16.89 ± 6.67 APACHE II and SAPS II 33.72 ± 13.45 points. Eight patients needed non invasive mechanical ventilation (44.4 %) with a failure rate of 50 % (4 patients). The use of invasive mechanical ventilation was required in 10 patients, 3 of them needed tracheostomy for prolonged mechanical ventilation and sevoflurane Anaconda® device was used in 2 of them. Data respirator parameters were collected during the first 48 hours, highlighting VT (ml / kg) 6.46 ± 1.23, 2.25 ± 2.26 initial PEEP, Ppeak 37.0 ± 13.32 and 12.57 ± 4.68 AutoPEEP cm H2O. The average pH at admission was 7.21 ± 0.14 with a range of [6.99 to 7.40], PCO2 of 64.5 ± 24.22 mm Hg and a lactate concentration of 2.91 ± 2.11 mMol/L. All patients were administered inhaled beta-agonists (two patients salbutamol endovenous) and corticosteroids. 5 patients required magnesium sulfate, 11 patients required sedation; 6 of these patients required relaxation with atracurium or cisatracurium. One patient presented barotrauma while using noninvasive mechanical ventilation prior to endotracheal intubation.

**Conclusions**

The clinical profile of patients admitted to the ICU with severe/status asthmaticus is a young male, with requirements of invasive mechanical ventilation after the failure in using medical gases such as helium and noninvasive mechanical ventilation. The strategy of ventilation is protective with permissive hypercapnia.

**Table 72 (abstract A220).** Characteristics (n = 18)

Average age	35,06
Woman	6 (33,3%)
Average stay (days)	14,39±25,55
APACHE II	16,89±6,67
INVASIVE MECHANICAL VENTILATION	10 patients
Tidal Volume (ml/kg)	6,46±1,23
AutoPEEP (cm H2O)	12,57±4,68)
pH at admission	7,21±0,14. Range [6,99-7,40]
pCO2 (mm Hg)	64,5±24,22
Ion lactate at admission	2,91±2,11

### A221 2015/16 influenza pneumonitis: mater misericordiae university hospital intensive care unit

#### J. Scott^1^, D. Phelan^2^, D. Morely^2^, J. O'Flynn^2^, P. Stapleton^3^, M. Lynch^3^, B. Marsh^1^, E. Carton^1^, C. O'Loughlin^1^

##### ^1^Mater Misericordiae University Hospital, ICU, Dublin, Ireland; ^2^Mater Misericordiae University Hospital, Dublin, Ireland, ^3^Mater Misericordiae University Hospital, Microbiology, Dublin, Ireland

###### **Correspondence:** J. Scott – Mater Misericordiae University Hospital, ICU, Dublin, Ireland

**Introduction**

Viral influenza, especially influenza A (H1N1) pandemic influenza, is associated with a significant increase in morbidity and Intensive Care Unit (ICU) admissions.

**Objectives**

The aim of this study is to characterise the burden of illness and secondary infection among critically unwell patients admitted to our ICU this year.

**Method**

A retrospective, observational, cohort study of critically ill adult patients with influenza admitted to a Level 3 ICU Dublin, between Decemeber 2015 and March 2016. IntelliVue Clinical Information Portfolio (ICIP) was used to obtain data.

**Results**

Critical illness occurred in twenty patients with confirmed influenza; 12 A(H1N1) (60 %), 1 A(H3N2) (5 %), 4 A(non-subtyped) (20 %) and 3 B (15 %). The median age was 50 years (42.5-66.5); 14 patients (70 %) were under 65; 11 (55 %) were male. Thirteen (65 %) had comorbidities, including respiratory disease 5 (25 %), morbid obesity 3 (15 %) and malignancy or immunosuppression 3 (15 %). One was pregnant (5 %) and 8 (40 %) had a smoking history. Nine (45 %) were retrieved from outside the Ireland East Hospital Group. The mean APACHE (Acute Physiology and Chronic Health Evaluation) II Score and the mean SOFA (Sequential Organ Failure Assessment) score, on day one, were 17.85 ± 5.78 and 9.85 ± 3.33 respectively. Twenty (100 %) were mechanically ventilated, for a median of 17.5 days (9.5-32). Eighteen (90 %) satisfied the criteria for Acute Respiratory Distress Syndrome, with a mean PaO2/FIO2 ratio on day one of 13.35 ± 6.46. Fifteen (83.3 %) required rescue therapies for severe hypoxaemia, including extracorporeal life support (ECLS) in five (25 %), prone ventilation and inhaled nitric oxide. Sixteen (80 %) received vasopressors, and 12 (60 %) required renal replacement therapy. The median ICU length of stay was 18.5 days (11-42) and as of 31st March 2016, three (15 %) had died. Four (20 %) had documented early secondary infection, at less than 48 hours; *streptococcus pneumoniae* was the sole isolate (100 %). Seven (35 %) were treated for presumed late (greater than 48 hours) secondary infection; *aspergillus fumigatus* (28.6 %) and pansensitive *staphylococcus aureus* (42.9 %) were the most prevalent. All patients received a neuraminidase inhibitor: oseltamivir was prescribed in 19 (95 %), for a median of 7 days (7-9.75), with 3 (20 %) patients receiving the higher, 150 mg twice daily, dosing regime.

**Conclusion**

Seasonal influenza is a major public health concern. It is associated with severe morbidity, resulting in significant economic consequences, as well as a substantial burden on tertiary ICUs. In keeping with national trends, the predominant circulating virus was influenza A(H1N1) and secondary coinfection was common. Although traditional teaching emphasises *S.aureus* as a common coinfection in viral illness, our results highlight the importance of considering a broad spectrum of bacterial, viral and fungal microorganisms when prescribing empirically in the critically ill patient.

### A222 Predictors of successful endotracheal extubation

#### K.-C. Cheng, M.-I. Sung

##### Chi Mei Medical Center, Internal Medicine, Tainan, Taiwan, Province of China

###### **Correspondence:** K.-C. Cheng – Chi Mei Medical Center, Internal Medicine, Tainan, Taiwan, Province of China

**Introduction**

About 10-16 % patients are reintubated after planned endotracheal intubation for 48-72 hours. Reintubation will increase the risk of pneumonia, ventilator and ICU days, and resulting in 25-50 % mortality. Many studies had explored the risk factors of failed extubation, but the integrated indexes for successfully planned endotracheal extubation are inadequate.

**Objectives**

To establish useful predictors for successfully-planned extubation which can be followed by medical personnel.

**Method**

The patients admitted to the adult ICUs of a tertiary hospital in southern Taiwan, who met the criteria of intubated over 48 hours and prepared for extubation were collected retrospectively between January 2005 to December 2015. Patient's characteristics, disease severity, rapid shallow breath index (RSBI), maximal inspiratory pressure (MIP), maximal expiratory pressure (MEP), cuff leak test (CLT) before extubation, ICU days and outcomes were recorded retrospectively. CLT was classified as 2+ with audible flow without stethoscope, 1+ with audible flow by stethoscope and negative (N) with no audible flow even by stethoscope. Failure of extubation was defined as reintubated within 48 hours.

**Results**

Totally 7460 patients were enrolled with 436 (5.8 %) patients failure for extubation. The extubation failure rate of female was higher than male (7.1 % vs 5.1 %, P < 0.001). Failure group patients were noted to have older age (66.6 ± 14.5 vs 64.4 ± 16.4, P = 0.002), higher APACHE II score (17.0 ± 7.5 vs 16.2 ± 7.9, P = 0.044), lower coma scale (10.1 ± 3.8 vs 10.6 ± 3.8, P = 0.019), higher RSBI (69.2 ± 38.0 vs 58.6 ± 30.2, P < 0.001), lower MIP and MEP (-35.5 ± 15.1 vs -38.0 ± 14.7, P = 0.0001; 49.5 ± 28.2 vs 58.9 ± 30.5,P < 0.001 respectively), and higher mortality rate (24.6 % vs 10.7 %, P < 0.001) as compared with successful group. After multivariate logistic regression, cuff leak test 2 + (OR 1.85, P = 0.002), MEP≧55 cmH_2_O(OR 1.48, P = 0.001), RSBI < 68 (OR 1.47, P < 0.001) and MIP < -40cmH_2_O(OR 1.26, P = 0.048) were found as predictors for successful extubation.

**Conclusions**

Cuff leak test, RSBI < 58, MEP≧60 cmH_2_O and MIP < -40cmH_2_O are identified as effective predictors for successfully planned endotracheal extubation.

**References**

1. Penuelas O, Frutos-Vivar F, Fernandez C, et al. Characteristics and outcomes of ventilated patients according to time to liberation from mechanical ventilation. *American journal of respiratory and critical care medicine* 2011;**184**(4): 430-7. 2. Thille AW, Harrois A, Schortgen F, Brun-Buisson C, Brochard L. Outcomes of extubation failure in medical intensive care unit patients. *Critical care medicine* 2011; **39**(12): 2612-8.

3. Thille AW, Boissier F, Ben Ghezala H, Razazi K, Mekontso-Dessap A, Brun-Buisson C. Risk factors for and prediction by caregivers of extubation failure in ICU patients: a prospective study. *Critical care medicine* 2015; **43**(3): 613-20

**Grant acknowledgment**

Financial support by Chi Mei Medical Center

### A223 Measuring intra-abdominal pressure during spontaneous breathing trial, does it help?

#### M.O. Elghonemi, M.H. Saleh

##### Kasr Alainy Medical School, Cairo University, Critical Care Medicine Department, Cairo, Egypt

###### **Correspondence:** M.O. Elghonemi – Kasr Alainy Medical School, Cairo University, Critical Care Medicine Department, Cairo, Egypt

**Background**

Respiratory system impairment may be caused by an increase of the intra-abdominal pressure.

Aim of work: To assess the role of measuring intra-abdominal pressure in predicting successful weaning from mechanical ventilation.

**Method**

124 patients with ARF fulfilling the criteria for weaning were included. Each underwent a 1-hour SBT. All clinical, respiratory parameters and mechanics were recorded. IAP was measured using Kron`s technique at the beginning and every 15 minutes till the end of SBT. The mean of IAP during SBT was calculated.

**Results**

Of 124 patients included in the study, 94 patients achieved successful SBT and extubation, while 31 patients needed re-intubation within 48 hours. Mean IAP was lower in patients achieved successful SBT compared to patients who didn`t, 7.25 ± 2.28 vs 9.96 ± 2.6, p value < 0.001. Moreover, patients who needed re-intubation within 48 hours had higher mean IAP compared to patients who didn`t, 9.96 ± 1.4 vs 5.92 ± 1.17, P value < 0.001. Using Spearman´s rank correlation coefficient, it was found that mean IAP was positively correlated with auto PEEP that measured at the beginning and at the end of SBT, and admission APACHE II score, with correlation coefficient measuring 0.515, 0.595, and 0.4 respectively.

**Conclusion**

High IAP predicts failure of SBT and need for re-intubation within 48 hours.

**References**

1 -Torquato Jamili Anbar, Lucato Jeanette Janaina Jaber, Antunes Telma, Barbas Carmen Valente. Interaction between intra-abdominal pressure and positive-end expiratory pressure. Clinics. 2009 Feb; 64(2): 105-112.

2- Malbrain ML. Different Techniques to measure intra-abdominal pressure (IAP): time for a critical re-appraisal. Int Care Med. 2004;30:357-71.

### A224 The association between the use of life support and 90-day mortality in an international cohort of adult ICU patients

#### T.S. Meyhoff, M. Krag, P.B. Hjortrup, A. Perner, M.H. Møller

##### Copenhagen University Hospital, Department of Intensive Care 4131, Copenhagen, Denmark

###### **Correspondence:** T.S. Meyhoff – Copenhagen University Hospital, Department of Intensive Care 4131, Copenhagen, Denmark

**Introduction**

The use of life support in intensive care units (ICUs) is common and associated with a high risk of poor outcome. However, the prognostic importance of the duration of life support is less studied.

**Objectives**

We aimed to assess the use of life support and the association between its duration and 90-day mortality in adult ICU patients.

**Method**

We performed a post-hoc analysis of the SUP-ICU 7-day inception cohort study (1) conducted from Dec 2013 till April 2014 in 97 ICUs in 11 countries. From this cohort (n = 1034), we included adult general ICU patients with an ICU stay of ≥3 days. We assessed the use of life support day 1-3 in the ICU and the crude and adjusted association between its duration and 90-day mortality using logistic regression analysis.

**Results**

**Fig. 96:**
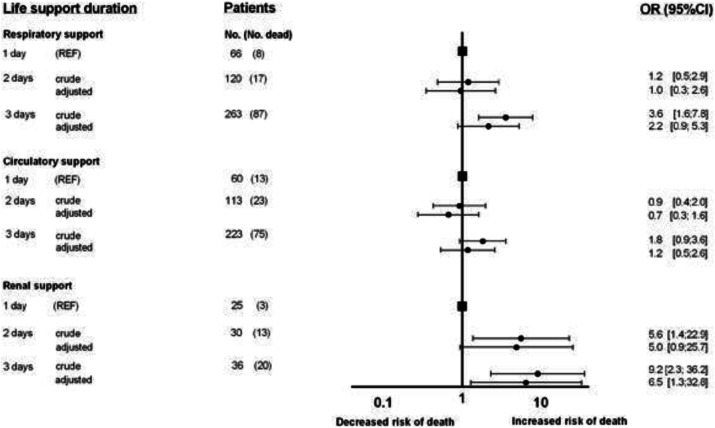
(abstract A224).

We included 690 patients with a 90-day mortality rate of 23 %. During the first 3 days in ICU, 65 % of the patients received respiratory support, 57 % circulatory support and 13 % renal replacement therapy (RRT). Patients receiving 3 days of RRT had the worst outcome (OR 6.5 [95 % CI 1.3 - 32.8]) as compared to patients receiving 1 day. For respiratory- and circulatory support the odds ratios were 2.2 [0.9 - 5.3] and 1.2 [0.5 - 2.6], respectively (Fig. 96).

**Conclusions**

The outcome of adult ICU patients was associated with both type and duration of life support. RRT seemed to be associated with worst outcome, potentially because kidney failure often occurs concomitantly to respiratory and circulatory failure.

**References**

(1) Krag M, Perner A, Wetterslev J, et al. Prevalence and outcome of gastrointestinal bleeding and use of acid suppressants in acutely ill adult intensive care patients. *Intensive Care Med* 2015; 41:833-45.

**Grant acknowledgment**

We received support from The Novo Nordisk Foundation (NNF15OC0017574). The funding source had no influence on the design of the study.

### A225 Evaluation of a capnodynamic method for assessment of effective lung volume in intubated pigs during hypercapnia

#### T. Öhman^1^, T. Sigmundsson^1^, E. Redondo^2^, M. Hallbäck^3^, F. Suarez-Sipmann^4^, H. Björne^1^, C. Hällsjö Sander^1^, KARISMA

##### ^1^Karolinska University Hospital, Department of Anaesthesiology, Surgical Services and Intensive Care Medicine, Stockholm, Sweden; ^2^Hospital de Navarra, Department of Intensive Care Medicine, Pamplona, Spain; ^3^Maquet Critical Care AB, Solna, Sweden; ^4^Uppsala University, Hedenstierna Laboratory, Department of Surgical Sciences, Uppsala, Sweden

###### **Correspondence:** T. Öhman – Karolinska University Hospital, Department of Anaesthesiology, Surgical Services and Intensive Care Medicine, Stockholm, Sweden

**Introduction**

Effective lung volume (ELV) can be calculated continuously using a capnodynamic equation, and correlates well with functional residual capacity (FRC) in healthy lungs in a porcine model [1]. Hypercapnia is common in the ICU during lung protective mechanical ventilation, and may affect the capnodynamic method.

**Objectives**

The aim of this study was to evaluate ELV during induced hypercapnia in pigs, and to confirm its stability during hemodynamic challenges.

**Method**

A cyclic sequence altering breaths with expiratory holds with normal breaths induces periodic changes in alveolar concentration of carbon dioxide. By integrating these variations into the capnodynamic equation ELV can be calculated. Hypercapnia was induced by increasing instrumental dead space in eight anaesthetized, relaxed and mechanically ventilated pigs. FRC was measured with a Sulfur-hexa-fluoride wash out technique. Cardiac output (CO) was measured using an ultrasonic flow probe placed around the pulmonary artery trunk. Hemodynamic measurements and blood gas analysis were obtained during normocapnia and during hypercapnia at baseline, preload reduction (cava balloon inflation) and dobutamine stimulation.

**Results**

**Fig. 97 (abstract A225).**
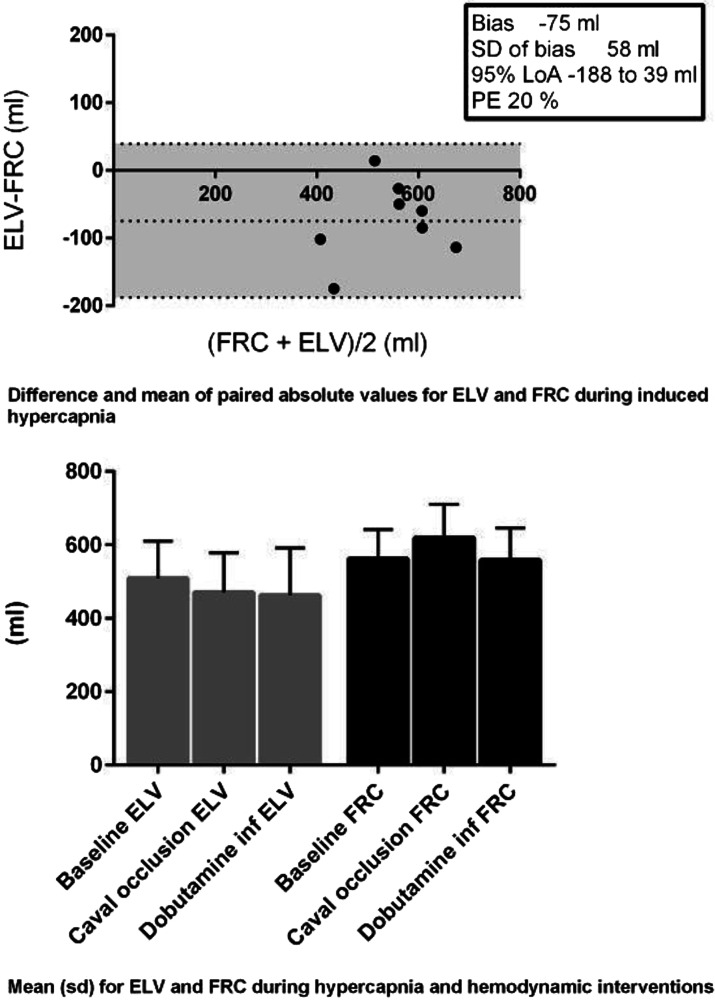
ELV during induced hypercapnia

Carbon dioxide levels raised from (mean (SD)) 5.6 kPa (0.40) to 9.2 kPa (0.47) during hypercapnia. The bias (limits of agreement, LoA) for ELV at normocapnia was 303 (131 to 476) ml, and percentage error (PE) was 31 %. During hypercapnia, bias (LoA) decreased to -75 (-188 to 39) ml, and PE to 20 %. The hemodynamic interventions resulted in significant changes in CO, i.e. a decrease by 41 % (caval occlusion) followed by a 59 % increase (dobutamine inf.). ELV and FRC remained stable throughout these changes (Fig. 97).

**Conclusions**

ELV showed good performance during hypercapnia. The Method shows good stability during severe changes in cardiac output. This indicates that it would be interesting to further evaluate if the Method could be suitable for monitoring lung function in the ICU for instance during protective lung ventilation with permissive hypercapnia or in septic patient with hyper dynamic hemodynamics.

**References**

1. Capnodynamic assessment of effective lung volume during cardiac output manipulations in a porcine model. Hällsjö Sander C, Lönnqvist PA, Hallbäck M, Sipmann FS, Wallin M, Oldner A, Björne H. J Clin monit Comput 2015 Sept: 1-9

**Grant acknowledgment**

Håkan Björne recives grants from Maquet critical care

### A226 Collapse and decollapse in acute respiratory distress syndrome

#### M. Cressoni^1^, D. Chiumello^2,3^, C. Chiurazzi^4^, M. Brioni^1^, I. Algieri^1^, M. Guanziroli^1^, G. Vergani^1^, T. Tonetti^5^, I. Tomic^6^, A. Colombo^1^, F. Crimella^1^, E. Carlesso^1^, A. Colombo^2^, V. Gasparovic^6^, L. Gattinoni^5^

##### ^1^Università degli Studi di Milano, Dipartimento di Fisiopatologia Medico Chirurgica e dei Trapianti, Milano, Italy; ^2^Policlinico Di Milano, Dipartimento di Anestesia, Rianimazione, Urgenza ed Emergenza, Milano, Italy; ^3^Plug Working Group, Milan, Italy; ^4^Università degli Studi di Milano, Dipartimento di Fisiopatologia Medico-Chirurgica e dei Trapianti, Milano, Italy; ^5^Georg-August-University Goettingen, Anesthesiology and Intensive Care Medicine, Goettingen, Germany; ^6^University of Zagreb, Department of Intensive Care Medicine, Rebro, Croatia

###### **Correspondence:** C. Chiurazzi – Università degli Studi di Milano, Dipartimento di Fisiopatologia Medico-Chirurgica e dei Trapianti, Milano, Italy

**Introduction**

In ARDS, independent of severity, lung protective strategy implies a high-PEEP ventilation setting to prevent end-expiratory collapse and to prevent cyclic alveolar opening and closing. [1] [2]

**Objectives**

Measure the intratidal collapse and decollapse at similar tidal volumes at positive end-expiratory pressure (PEEP) of 5 and 15 cmH_2_O.

**Method**

ARDS patients [3] underwent expiratory and inspiratory CT scans at 5-15 cmH_2_O PEEP during inspiratory and expiratory pause keeping constant the tidal volume (6-8 ml/kg IBW). In each of the CT slices, lung profiles were manually delineated, excluding hilar structures. Thereafter, quantitative analysis of CT scan images was performed with a dedicated software package (Soft-E-Film, www.elekton.it). Lung tissue was classified, according to its gas/tissue content, as not inflated when CT number between +100 and -100. [4] Lung collapse-decollapse was estimated as:

1. Collapse-decollapse PEEP 5 cmH_2_O = not inflated 5 insp (g) - not inflated 5 esp (g) Collapse-decollapse PEEP 15 cmH_2_O = not inflated 15 insp (g) - not inflated 15 esp (g)

**Results**

**Fig. 98 (abstract A226).**
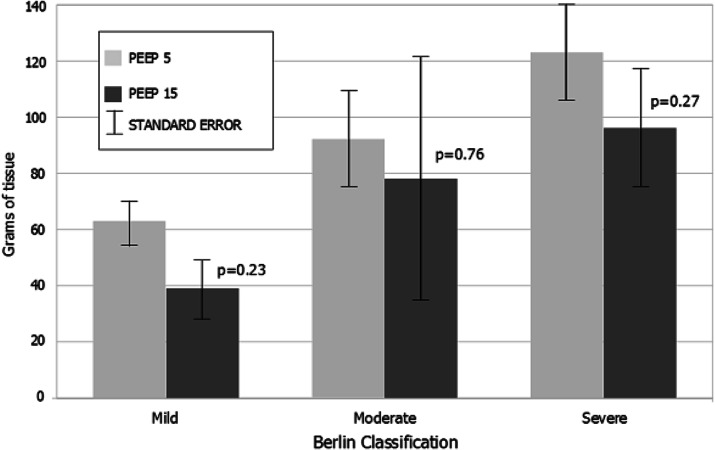
Intratidal collapse and decollapse

Thirty-three ARDS patients were enrolled (5 mild, 10 moderate and 18 severe). As shown in Fig. 98, within a given class of severity, the grams of tissue undergoing the intratidal collapse did not change significantly between PEEP 5 or 15 cmH_2_O (63 ± 26 vs 39 ± 32, 92 ± 53 vs 78 ± 142 and 123 ± 94 vs 96 ± 84 in mild, moderate and severe ARDS respectively). We observed a clear tendency to decrease from PEEP 5 to 15 cmH_2_O, though it was not statistically significant (p = 0.23, 0.76 and 0.27 respectively in mild, moderate and severe ARDS - paired t-test).

**Conclusions**

A consistent intratidal collapse and decollapse is still present at 15 cmH_2_O PEEP. We observed a clear tendency to decrease at 15 cmH_2_O PEEP, though it was not significant.

**References**

[1] Muscedere JG, *Am J Respir Crit Care Med* 1994. [2] Caironi P, *Am J Respir Crit Care Med* 2010 [3] ARDS Definition Task Force,*JAMA J Am Med Assoc* 2012. [4] Gattinoni L, *Am Rev Respir Dis* 1987

### A227 Low level laser therapy in chronic obstructive pulmonary disease

#### R. El-Sherif^1^, M. Abd Al-Basser^1^, A. Raafat^2^, A. El-Sherif^1^

##### ^1^Kasr Al-Aini Hospitals, Cairo University, Critical Care Department, Cairo, Egypt; ^2^Cairo University, Physiotherapy Department, Cairo, Egypt

###### **Correspondence:** A. Raafat – Cairo University, Physiotherapy Department, Cairo, Egypt

**Introduction**

Chronic obstructive pulmonary disease (COPD) is a common preventable and treatable disease. Low level laser (LLL) therapy appears to be a promising noninvasive modality in COPD management.

**Objectives**

Study the short-term effects of LLL therapy on clinical and cardiac status in stable COPD patients.

**Method**

After exclusion of patients with Impaired left ventricular systolic function, atrial fibrillation, pulmonary hypertension due to causes other than COPD, and those who had any contraindication to exercise test, thirty stable COPD patients were divided into laser and control groups (15 pts each). Medical treatment was optimized in each group with the addition of LLL in the laser group.

In addition to history and physical examination, MMRC scale, 6 MWT, echocardiography with measurements of RV dimensions, TAPSE, and lateral tricuspid annulus tissue Doppler velocities were assessed in each patient before and after LLL. The LLL was done using the following parameters: Wave length: 905 nm, Output 5-20 mw & Frequency 500 HZ. Laser probe was placed on intercostal space corresponding to the site of lesion both anteriorly and posteriorly on chest wall and arm with standardized laser acupuncture points of application with a frequency of 5 sessions/week for 2 successive weeks.

**Results**

No significant differences in both groups regarding demographic data. The laser group had higher PASP, lower E´, and higher A´ velocities versus control with p 0.009, 0.03, and < 0.0001 respectively. The laser group showed more improvement in MMRC scale and 6 MWT versus control. 100 % of laser patients showed improvement in MMRC scale by at least one grade versus 46 % in the control. In laser group, 6 MWT was 24.4 ± 10.4 before the study versus 52.9 ± 14.7 meters at the end of the study, p 0.001. In control, 6 MWT was 32.4 ± 14.9 versus 40.1 ± 19.2, p 0.003. No significant changes between any of the echocardiographic criteria before and after the study.

**Conclusions**

The use of LLL was associated with more clinical improvement. No echocardiographic changes were noticed after LLL.

### A228 Physiotherapy assessment of extubation suitability

#### L.E. Brock^1^, L. Osman^2^, G. Cork^3^

##### ^1^Guys and St Thomas' NHS Foundation Trust, Physiotherapy, London, United Kingdom; ^2^Guys and St Thomas' NHS Foundation Trust, London, United Kingdom; ^3^Guy's and St Thomas' NHS Foundation Trust, London, United Kingdom

###### **Correspondence:** L.E. Brock – Guys and St Thomas' NHS Foundation Trust, Physiotherapy, London, United Kingdom

**Introduction**

Timing of extubation is of clinical importance as extended periods of intubation and premature extubation resulting in reintubation are both associated with negative patient outcomes[1]. Adult intensive care unit (AICU) extubation failure rates of 10-20 % have been reported[2].

Within a large, UK teaching hospital it was identified that extubation assessment was a multidisciplinary decision but different clinicians applied varying criteria and assessment was not standardised. Following a review of extubation failure rates, a quality improvement initiative was instigated including the development of local ventilator weaning guidelines and physiotherapy (PT) led assessment of extubation suitability.

**Objectives**

The aims of this evaluation were to describe the PT assessment of extubation suitability in the AICU and to report extubation failure rates.

**Method**

All patients under consideration for extubation by the AICU physicians, who underwent a PT assessment of extubation were included. This assessment was documented within the patient's electronic casenotes.

Data was collected by retrospective casenote review during a 3 week period in August 2015. The project was registered as a service evaluation and therefore ethics requirements were waived. Demographics, details of the PT assessment ,and outcomes following extubation were collected (Table 72). Extubation failure was defined as reintubation up to one week following extubation.

**Results**

Data was collected from 45 PT assessments. These assessments most frequently included neurological status, Rapid Shallow Breathing index (RSBi), occlusion pressure during initial 100 ms of inspiration (P0.1), Negative Inspiratory Force (NIF), peak cough flow (PCF) and secretion load.

Range of values for the assessed parameters are shown below.

When providing opinion regarding suitability for extubation, PTs gave more weight to neurological status, PCF and secretion load than other weaning parameters (see image below). They were more likely to recommend against extubation in the presence of low PCF, inappropriate neurology and large secretion load.

**Conclusions**

Physiotherapists frequently include neurological status, work of breathing, PCF, secretion load and NIF in their assessment for extubation. They predominantly use neurological status, cough strength and secretion load to inform recommendations regarding extubation. The extubation failure rate of 15 % is in keeping with current literature although the studied sample size was small.

**References**

1. Menon N, Joffe AM, Deem S, Yanez ND, Grabinsky A, Dagal AH, Daniel S, Treggiari MM. Occurrence and complications of tracheal reintubation in critically ill adults. Respir Care. 2012 Oct;57(10):1555-63.

2. Thille AW, Richard JC, Brochard L. The decision to extubate in the intensive care unit. Am J Respir Crit Care Med. 2013 Jun 15;187(12):1294-302.

**Table 73 (abstract A228).** Demographics

Demographic	
Age (median)	56.5
% male	68
Number of patients	33
Number of assessments	45
Days ET ventilated (median)	3
Extubationn events	33
Failure rate	15%

**Table 74 (abstract A228).** Parameters assessed

Parameter	Frequency of assessment (%, n=45)
Neurology	88
RSBi	90
P01	95
NIF	90
PCF	100
Secretion load	90

**Fig. 99 (abstract A228).**
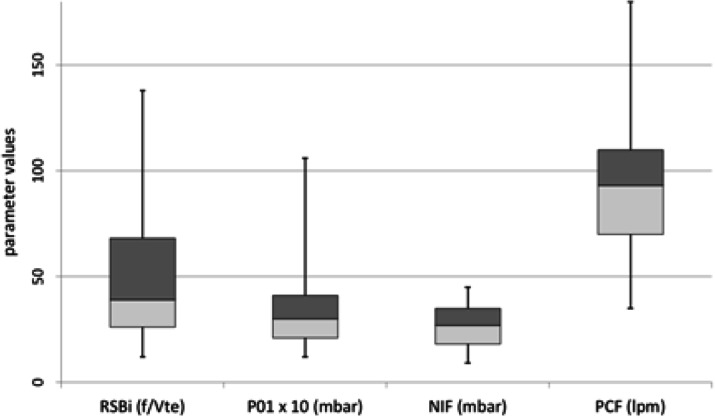
Ranges of parameters assessed

**Fig. 100 (abstract A228).**
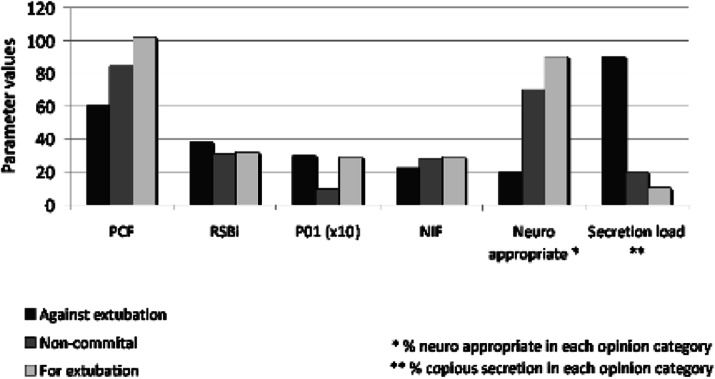
Parameters in relation to PT advice given

## CLINICAL STUDIES ON ARDS AND MECHANICAL VENTILATION

### A229 Improved prognostication of patients with mild ARDS based on P/F and PEEP thresholds 24 hours after presentation

#### F.D. Simonis^1^, L.R.A. Schouten^1^, O.L. Cremer^2^, D.S.Y. Ong^2^, G. Amoruso^3^, G. Cinnella^3^, M.J. Schultz^1^, L.D.J. Bos^1^

##### ^1^Academic Medical Center, Amsterdam, Netherlands; ^2^University Medical Center Utrecht, Utrecht, Netherlands, ^3^University of Foggia, Foggia, Italy

###### **Correspondence:** F.D. Simonis – Academic Medical Center, Amsterdam, Netherlands

**Introduction**

Reclassification after 24 hours using PaO_2_/FiO_2_ and PEEP thresholds improves the prognostication of patients with moderate/severe ARDS [1,2] but it is uncertain if this also holds true for patients with mild ARDS.

**Objectives**

The aim of this investigation was to determine if reclassification after 24 hours using PaO2/FiO2 and PEEP thresholds improves prognostication of mortality in a cohort of prospectively identified patients with mild ARDS in two intensive care units in the Netherlands.

**Method**

Patients with mild ARDS, according to the Berlin definition [3], were categorized into 4 groups based on measurements obtained at presentation of ARDS or 24 hours after: PaO2/FiO2 ≥ 250 mm Hg and PEEP = 5 cm H2O (group I); PaO2/FiO2 ≥ 250 mm Hg and PEEP > 5 cm H2O (group II); PaO2/FiO2 < 250 mm Hg and PEEP = 5 cm H2O (group III); PaO2/FiO2 < 250 mm Hg and PEEP > 5 cm H2O (group IV). Patients no longer receiving mechanical ventilation after 24 hours were classified as 'extubated' (group 0). No patients died within 24 hours. The primary outcome was all-cause in hospital mortality. Secondary outcomes were , ICU- and 90-day mortality and the number of ventilator-free days and alive at day 28.

**Results**

Of 7,784 patients, 693 patients had ARDS of which 164 patients with mild ARDS and on invasive ventilation were included in the analysis. Table 75 shows outcomes per group at the moment mild ARDS was diagnosed, and after 24 hours. Reclassification after 24 hours showed an improved prognostication with regard to hospital mortality, ICU- and 90-day mortality and the number of ventilator-free days and alive at day 28.

**Conclusions**

Reclassification after 24 hours using two simple cutoffs improves prognostication in mild ARDS patients.

**References**

1. Villar J, Fernández RL, Ambrós A, et al. Crit Care Med 2015; 43: 346

2. Bos LD, Cremer OL, Ong DSY, et al. Intensive Care Med 2015; 41:2004

3. The ARDS Definition Task Force. JAMA 2012; 307:2526

**Grant acknowledgment**

This research was performed within the framework of CTMM, the Center for Translational Molecular Medicine (www.ctmm.nl) project MARS (grant 04I-201).

**Table 75 (abstract A229).** Distribution of outcome in patients with mild ARDS

at onset		Group 0 (n=0)	Group I (n=28)	Group II (n=29)	Group III (n=38)	Group IV (n=69)	*P-value*
	ICU mortality	-	7%	17%	18%	26%	0.03
	All-cause hospital mortality	-	39%	38%	29%	36%	0.73
	90-day mortality	-	39%	38%	42%	40.5%	0.83
	VFD-28	-	22 [15-25]	23 [7-25]	22 [8-25]	17 [0-24]	0.09
after 24 hours		Group 0 (n=20)	Group I (n=27)	Group II (n=15)	Group III (n=42)	Group IV (n=60)	*P-value*
	ICU mortality	0%	7%	20%	12%	37%	<0.0001
	All-cause hospital mortality	10%	33%	27%	38%	45%	0.0073
	90-day mortality	20%	30%	33%	45%	50%	0.0057
	VFD-28	27 [27-27]	21 [11-25]	20 [2-24]	21 [13-24]	11 [0-21]	<0.0001

Data are medians [IQR] or percentages. P-value is p-for trend or p for linear regression

Abbreviations: *ARDS* acute respiratory distress syndrome, *VFD-28* ventilator free days and alive at day 28

### A230 Time-dependent predictive capabilities of ARDS-scores regarding mortality: a prospective observational study in patients screened for the exodus-study

#### W. Huber^1^, P. Schmidle^1^, M. Findeisen^2^, P. Hoppmann^3^, J. Jaitner^3^, F. Brettner^4^, R.M. Schmid^1^, T. Lahmer^1^, EXODUS-investigators

##### ^1^Technische Universität München, II. Medizinische Klinik, Munich, Germany; ^2^Städtisches Klinikum München Harlaching, Munich, Germany; ^3^Technische Universität München, I. Medizinische Klinik, Munich, Germany; ^4^Krankenhaus Barmherzige Brüder, Munich, Germany

###### **Correspondence:** W. Huber – Technische Universität München, II. Medizinische Klinik, Munich, Germany

**Introduction** ARDS still carries a high mortality. Therefore, extracorporeal lung membrane oxygenation (ECMO) should be considered in these patients. However, indication and timing of ECMO are still controversial. Several recent ECMO registries suggest that early initiation of ECMO might be more beneficial than its use as a rescue therapy. However, there is no consensus, according to which criteria ECMO should be initiated. Most of the recent multi-centric studies such as CESAR, EOLIA and EXODUS (NCT02550600) include(d) patients based on ARDS definitions and on scores like the Murray-score. However, the predictive capabilities of these scores depending on the time after intubation are poorly investigated.

**Objectives**

To compare the predictive capabilities of pO2/FiO2, oxygenation-index OI (mean airway pressure*FiO2/pO2), AECC- and Berlin-definition of ARDS, single components of the Murray-score and its total score without radiological points (Murray-WRP) regarding ICU-, 28d- and hospital mortality in 43 patients screened for the EXODUS-study.

**Method**

28 male, 15 female patients with ARDS according to Berlin-definition. PiCCO-monitoring (Pulsion, Germany) in 25 patients. Statistics: SPSS 23.

**Results**

Primary ARDS in 17 of 43 cases (39 %), secondary ARDS in 26/43 (61 %). ALI in 22 (51 %) and ARDS according to AECC in 21 (49 %) out of 43 cases. Mild, moderate and severe ARDS according to the Berlin definition in 20 (46.5 %), 20 (46.5 %) and 3 (7 %) of the patients. Due to prolonged hospitalization the final outcome was available in only 40 of 43 patients. 28-day-, ICU- and hospital-mortality rates were 13/40 (33 %), 19/40 (48 %) and 20/40 (50 %). On the day of intubation, 28-day mortality (primary endpoint) was best predicted by OI (ROC-AUC 0.725; p = 0.026), pO2/FiO2 (ROC-AUC 0.725; p = 0.026) and Murray-WRP (AUC 0.702; p = 0.046). By contrast, AECC (AUC 0.648), Berlin (AUC 0.667) and APACHE-II (AUC 0.639) were not predictive. While Murray-WRP was predictive on day-1, it was not predictive on day-2 (AUC 0.688; p = 0.057), day-3 (AUC 0.604; p = 0.338) and day-4 (AUC 0.598; p = 0.390).

Pulmonary vascular permeability index PVPI on day-2 (AUC 0.825; p = 0.013; largest of all AUCs), on day-3 ( AUC 0.810; p = 0.018) and on day-4 (AUC 0.800; p = 0.048) as well as extravascular lung water index EVLWI on day-2 (AUC 0.782; p = 0.032) significantly predicted mortality. PVPI > 2 on day-2 predicted 28d-mortality with a specificity of 94 %, a sensitivity of 57 % and an accuracy of 80 %.

**Conclusions**

OI, pO2/FiO2 and Murray-WRP predict mortality of patients with ARDS on the day of intubation. By contrast, AECC- and Berlin-definition were not predictive. Since Murray-WRP on the 2^nd^, 3^rd^ and 4th day after intubation were less predictive than on the day of intubation, earlier consideration of ECMO might be preferable. High values of PVPI and EVLWI on day-2 were the best predictors of 28-d mortality in the subgroup with PiCCO.

**Grant acknowledgment**

EXODUS is supported by Xenios, Germany

### A231 The effect of budesonide/formoterol on biomarkers of acute lung injury in the LIPS-B trial

#### E. Festic^1^, G. Rajagopalan^2^, V. Bansal^1^, R. Frank^1^, R. Hinds^2^, J. Levitt^3^, United States Critical Illness and Injury Trials Group/LIPS-B investigators

##### ^1^Mayo Clinic, Jacksonville, FL, United States; ^2^Mayo Clinic, Rochester, NY, United States; ^3^Stanford University, Stanford, CA, United States

###### **Correspondence:** E. Festic – Mayo Clinic, Jacksonville, FL, United States

**Introduction** In the Lung Injury Prevention Study with Budesonide and formoterol (LIPS-B), we studied feasibility of nebulized budesonide and formoterol to prevent or alleviate lung injury in patients at risk for ARDS. All available blood samples obtained prior to study drug and on hospital day 2 were analyzed for biomarkers associated with progression or severity of ARDS.

**Objectives**

To determine if early administration of inhaled budesonide and formoterol modulates ARDS-associated plasma biomarkers.

**Method**

Serum samples were analyzed in duplicates with Luminex Plate in a 1:2 dilution and per manufacturer´s directions. Analytes were IL-6, vWF-A2, IL-8, IL-10, RAGE, ICAM-1, and SP-D. We analyzed biomarker concentrations in specimens from the treatment versus placebo arms at baseline (before the study drug delivery) and day 2 (after at least one dose of the study drug). The observed concentrations that were above or below calibrated expected range were replaced with highest and lowest observed within the range concentration values, respectively. Subsequently, the values were log-transformed and differences between treatment arms were compared by type 3 folded F test.

**Results**

Of 59 enrolled patients, 39 had samples available for baseline and day 2. The log-transformed values of IL-6, vWF-A2, IL-8, RAGE, and ICAM-1 decreased on day 2 with the observed decreases being greater in the treatment arm. However, the differences were not statistically significant: IL-6 p = 0.16, vWF-A2 p = 0.9, IL-8 p = 0.68, RAGE p = 0.27, ICAM-1 p = 0.1. Interestingly, IL-10 also decreased in the treatment arm while slightly increasing in the placebo arm, although differences were not significant (p = 0.28). The concentration of SP-D increased on day 2 in both arms with a non-significant greater increase in the placebo arm (p = 0.5).

**Conclusions**

Early treatment with nebulized budesonide and formoterol in patients at risk for ARDS in LIPS-B trial did not significantly alter serum levels of biomarkers associated with acute lung injury on day 2 of hospital admission. However, IL-6 and ICAM-1 showed trends toward greater reduction in treated patients and our small sample size likely lacked power to detect smaller differences between two arms.

### A232 National survey of outcomes and practices in acute respiratory distress syndrome (SOAP-ARDS)

#### S. Siddiqui, SICM NICER Group

##### Khoo Teck Puat Hospital, Singapore, Singapore

###### **Correspondence:** S. Siddiqui – Khoo Teck Puat Hospital, Singapore, Singapore

**Introduction**

In the past 20 years, management of the acute respiratory distress syndrome (ARDS) has been revolutionised by the application of lower tidal volume, lung protective ventilation strategies. Whilst our understanding of ARDS management has improved, the worldwide incidence and outcomes are unclear, with several studies reporting highly variable regional incidence rates, and no studies characterising ARDS epidemiology in Asia.

**Objectives**

The goal of this observation study was to determine the incidence, mortality and management practices of ARDS in a developed South East Asian country.

**Method**

We conducted a prospective, population based observational study in 6 public hospitals. During a one month period from May to June 2015, we identified all patients admitted to any Singapore public hospital intensive care unit (ICU) who met ARDS criteria. Demographic information, clinical management data and ICU outcomes data were collected. Concurrently, a survey was conducted to determine ARDS management preferences of physicians at the study centres involved.

**Results**

A total of 904 adult patients were admitted to the ICUs during the study period and 16 patients met ARDS criteria, using the Berlin definition. Based on this, the unadjusted incidence of ARDS in Singapore is 5.49 cases per 100,000 population and 1.76 % of all ICU patients. Most patients belonged to Medical ICUs (56 %), were male (75 %), Chinese (62 %) and had pneumonia (73 %). Management strategies varied across all ICUs. Our survey results showed that the majority of physicians thought that it was useful to study ARDS prevalence but that they believed overall mortality and prevalence was decreasing. Our 28 day in hospital mortality was 15 % and median length of stay was 7 ± 3 days.

**Conclusions**

The incidence of ARDS in a developed S.E Asia country is comparable to European reported rates. The proportion of ventilated patients developing ARDS is lower than international studies published in different regions. However, management strategies varied considerably.

**References**

[1] Rubenfeld GD, Caldwell E, Granton J, Hudson LD, Matthay MA. Interobserver variability in applying a radiographic definition for ARDS. Chest. 1999;116(5):1347-1353.

[2] Villar J, Perez-Mendez L, Lopez J, et al. An early PEEP/FIO2 trial identifies different degrees of lung injury in patients with acute respiratory distress syndrome. Am J Respir Crit Care Med. 2007;176(8):795-804.

[3] The ARDS Definition Task Force. Acute respiratory distress syndrome: the Berlin definition. JAMA. Epub May 21, 2012.

[4] Bellingan GJ. The pulmonary physician in critical care * 6: the pathogenesis of ALI/ARDS. Thorax. 2002;57(6):540-546.

[5] Tomashefski JF., Jr Pulmonary pathology of the adult respiratory distress syndrome. Clin Chest Med. 1990;11(4):593-619.

**Grant acknowledgment**

We acknowledge receiving a NICER grant from Singapore Intensive care Medicine society.

### A233 Fluid balance in ARDS

#### J.P. Gilbert, K. Sim

##### Whiston Hospital, Department of Critical Care, Liverpool, United Kingdom

###### **Correspondence:** J.P. Gilbert – Whiston Hospital, Department of Critical Care, Liverpool, United Kingdom

**Introduction**

A restrictive, post resuscitation, fluid balance strategy for patients with Acute Respiratory Distress Syndrome (ARDS) has been suggested by the fluid and catheter treatment trial (FACTT) study in 2006. This showed increased ventilator free days and trends towards reduced mortality with no change in other organ dysfunction with restrictive fluid balance compared to a liberal fluid balance.

**Objectives**

Quantify fluid balance, severity of illness and mortality of ARDS patients. Aim to reduce post resuscitation fluid balance in our population.

**Method**

Retrospective review of all patients coded as ARDS for Intensive Care National Audit and Research Centre (ICNARC) from 1/1/12 to 31/12/15. A review of the electronic record and comparison of APACHE II score, ventilator free days, mortality and fluid balance. Data was compared to the FACTT study.

**Results**

86 patients were included for analysis, mean age was 53 with a predominance of females (54 %). 74 (86 %) were ventilated, with a mean ventilator free days of 9.7. Mean post resuscitation fluid balance was 4950mls. 51 % of patients had an underlying diagnosis of pneumonia. Mean APACHE II score was 17.5 and unit stay was 10.9 days. Overall mortality was 46 % compared to 25.5-28.4 % in the FACTT trial group. The mean fluid balance was 3 litres less in the survivors compared with those who died, however, APACHE II score was 14.8 in the survivors and 20.4 in the group that died (p value 0.03). Mortality was 37.0 % in patients who received a cumulative post resuscitation fluid balance of less than

1 litre and 49.1 % in patients with fluid balance greater than 1 litre (p value 0.35).

**Conclusions**

A trend towards improved mortality with less fluid was observed but not statistically significant. Higher than expected mortality could be explained by the exclusion criteria for the FACTT population in particular chronic lung disease. Further education of junior medical and nursing staff, a daily review of fluid balance has been emphasised and re-audit in 18 months to keep awareness high were recommended.

**Reference**

1. The National Heart, Lung, and Blood Institute Acute Respiratory Distress Syndrome (ARDS) Clinical Trials Network. Comparison of Two Fluid-Management Strategies in Acute Lung Injury. N Engl J Med 2006; 354:2564-2575.

### A234 Relationship between age and mortality of acute respiratory distress syndrome (ARDS)

#### C.-H. Wang^1^, H.-C. Hu^2^, I.-J. Li^3^, W.-R. Tang^1^, K.-C. Kao^4^

##### ^1^Chang Gung University, School of Nursing, College of Medicine, Taoyuan, Taiwan, Province of China; ^2^Chang Gung Memorial Hospital, Division of Thoracic Medicine, Taoyuan, Taiwan, Province of China; ^3^Taipei Tzu Chi Hospital, Buddhist Tzu Chi Medical Foundation, Department of Nursing, New Taipei City, Taiwan, Province of China; ^4^Chang Gung Memorial Hospital, Department of Respiratory Therapy, Taoyuan, Taiwan, Province of China

###### **Correspondence:** H.-C. Hu – Chang Gung Memorial Hospital, Division of Thoracic Medicine, Taoyuan, Taiwan, Province of China

**Introduction**

Old patients have become an increasingly prevalent proportion of the critically ill population. The outcomes of patients with acute respiratory distress syndrome (ARDS) were improving in recent years. However, limited information existed on the studies for elderly of ARDS patients.

**Objectives**

To evaluate the factors associated with mortality of ARDS patients and investigate the relationship between age and mortality in ARDS patients.

**Method**

We performed a prospective observational study in adult ICUs at the Chang Gung Memorial Hospital from October 2012 to May 2015. Patients were included if they met the Berlin definition of ARDS. Data collected included patients' demographic, severity of illness, management and clinical outcomes. All the studied patients were followed until discharged from the hospital.

**Results**

During the study period, 22470 receiving invasive mechanical ventilation patients admitted to adult ICUs with PaO2/FiO2 < 300 mmHg were screened. Totally, 945 ARDS patients were included for analysis. The mean age and hospital mortality were 63.2 ± 16.1 years and 56.9 %. Of these 945 ARDS patients, 463 (49 %) patients were older than 65 years. By multivariate logistic regression analysis, factor associated with hospital mortality included body mass index (odds ratio 1.07, 95 % confidence interval 1.04-1.11; *p* < 0.001), Sequential Organ Failure Assessment score (odds ratio 0.88, 95 % confidence interval 0.81-0.95; *p* = 0.002), Lung Injury Score (odds ratio 0.68, 95 % confidence interval 0.46-1.00; *p* = 0.05) and PaO2/FiO2 (odds ration 0.995, 95 % confidence interval 0.991-1.00; *p* = 0.04). The hospital mortality in ARDS patients younger than 65 years was significantly lower than ARDS patients older than 65 years patients (50.2 % vs. 63.9 %, *p* < 0.001). For ARDS patients older than 65 years, we classified the patients as the young-old (65 ~ 74 years, n = 194, 41.9 %), middle-old (75 ~ 84 years, n = 189, 40.8 %) and old-old (≥85 years, n = 80, 17.3 %). The hospital mortality were not significantly different between these three group (63.9 % vs. 65.6 % vs.60 %, *p* = 0.682).

**Conclusions**

For ARDS patients, younger patients had lower mortality than older patients. For ARDS patients older than 65 years, the age did not influence the hospital mortality.

**Keywords**

Elderly, Outcomes, Berlin definition, Mechanical ventilation

### A235 Prone position and lung ultrasound (PROPLUS) in ARDS

#### P. Persona^1^, A. De Cassai^2^, M. Franco^2^, F. Facchin^2^, C. Ori^2^, S. Rossi^1^, A. Goffi^3^

##### ^1^Azienda Ospedaliera di Padova, Emergency Department, Padova, Italy; ^2^University of Padova, Medicine Department, Padova, Italy; ^3^Toronto Western Hospital MSNICU, Toronto, Canada

###### **Correspondence:** P. Persona – Azienda Ospedaliera di Padova, Emergency Department, Padova, Italy

**Introduction**

ARDS is a life-threatening condition characterized by increased lung weight and loss of lung aeration. Recently, prone position as adjunct to lung protective ventilation demonstrated significant mortality reduction in ARDS patients[1]. Lung Ultrasound (LUS) has emerged as a powerful diagnostic tool that could help in diagnosis and guide management at the bedside. Performance of serial CT scans, the gold standard for lung recruitment assessment, can be challenging and not feasible outside research protocols.

**Objectives**

We hypothesized that, in ARDS patients, LUS could detect changes in regional inflation during prone position compared to supine position and over time. We also hypothesized that such changes correlate with commonly monitored parameters of aeration, oxygenation and ventilation, as measured by arterial blood gas analysis and respiratory mechanics. Finally, we hypothesized that specific LUS aeration patterns, identified immediately before and after prone position initiation, are predictive of clinical response to this adjunctive treatment.

**Method**

In this observational prospective study on ARDS patients, we performed LUS on the first day of prone position treatment at different time points: before (supine - S0), immediately after (P0) and 1 hour after (P1) initiation of prone position, immediately before (Pfin) and after returning the patient supine (Sfin). For the LUS protocol, we used a 2-4 MHz curvilinear transducer and we divided each hemithorax in 2 anterior, 2 lateral and 3 posterior zones. The worst LUS pattern detected in each zone was considered as characterizing the examined region. Off-line image review by two independent physicians, unaware of timing, position and patient's characteristics, was used to calculate a modified LUS aeration score, as previously described[2]. Spearman's rank correlation coefficient was used to correlate changes in LUS score (V-LUSS) with changes in compliance and PaO2/FiO2 (P/F).

**Results**

We enrolled 13 ARDS patients, admitted to the Padova University Hospital ICU [median age 58 years (IQR 53-61); median SAPSII 45.5 (IQR 33-52)]. V-LUSS between P0 and P1 correlated with changes in compliance (r = 0.690; p < 0.05) and P/F (r = 0.70; p < 0.02) between S0 and Sfin. V-LUSS P0-Pfin also correlated with changes in P/F (r = 0.61; p < 0.05) and compliance (r = 0.60; p < 0.05) between S0 and SFin.

**Conclusions**

LUS is feasible in ARDS patients in prone position and V-LUSS correlates with changes in compliance and P/F. Moreover, the V-LUSS at 1 hour after initiation of prone position may predict the change in compliance and P/F at the end of a pronation cycle, after returning the patient in supine position.

**References**

1. Guérin C, et al. Prone positioning in severe acute respiratory distress syndrome. *N Engl J Med*. 2013;368(23):2159-2168.

2. Soummer A, et al. Ultrasound assessment of lung aeration loss during a successful weaning trial predicts postextubation distress*. *Crit Care Med*. 2012;40(7):2064-2072.

### A236 High incidence of acute respiratory distress syndrome (ARDS) but same mortality in H1N1 pneumonia with respiratory failure

#### S.-H. Li, H.-C. Hu, L.-C. Chiu, C.-Y. Hung, C.-H. Chang, K.-C. Kao

##### Chang Gung Memorial Hospital, Division of Thoracic Medicine, Taoyuan, Taiwan, Province of China

###### **Correspondence:** S.-H. Li – Chang Gung Memorial Hospital, Division of Thoracic Medicine, Taoyuan, Taiwan, Province of China

**Introduction**

Influenza A (H1N1) pneumonia could cause severe hypoxemia and need mechanical ventilation support. The incidence of development to ARDS and the related mortality rate in H1N1 pneumonia with respiratory failure were not clear.

**Objectives**

Our purpose is to analyze the outcome of patients with ARDS caused by influenza A (H1N1) in intensive care unit (ICU) requiring mechanical ventilation.

**Method**

This was a retrospective study including adult patients with ARDS due to confirmed influenza A(H1N1) pneumonia needed mechanical ventilation in a medical center between July 2009 and May 2014 in Chang Gung Memorial Hospital in Taiwan. We investigated the patients' characteristics, clinical presentation, severity of illness and outcomes.

**Results**

There were 199 influenza-infected patients hospitalized in ICU during the study period. Seventy-three patients were confirmed influenza A (H1N1), and 54 (82 %) patients developed to ARDS, including 36 (67 %) with severe ARDS and 14 (26 %) with moderate ARDS by Berlin definition. Compared with non-ARDS influenza A (H1N1) patients, age was younger (50 ± 14 vs. 62 ± 16 years, *p* = 0.016) and body mass index (BMI) was higher (26.7 ± 4.7 vs. 23.7 ± 4.0 kg/m^2^, *p* = 0.045) in ARDS patients. The duration of oseltamivir therapy in ARDS patients was longer (7.2 ± 2.6 vs. 4.9 ± 0.7 days, *p* = 0.010) than no ARDS patients. Despite significantly prolonged mechanical ventilation support (20.7 ± 18.5 vs. 9.6 ± 9.8 days, *p* = 0.013) and intensive care unit stay (21.7 ± 16.0 vs. 8.7 ± 8.2 days, *p* = 0.003) in ARDS patients, there were no significant difference in the mortality rate of ICU (18.5 % vs. 33.3 %, *p* = 0.256) and hospital (37 % vs. 33.3 %, *p* = 0.809) between ARDS and non-ARDS patients.

**Conclusions**

For patients with influenza A (H1N1) pneumonia complicated respiratory failure, high incidence developed to severe ARDS but not correspond to high mortality rate.

**References**

• Estenssoro E, R ´ıos FG, Apeztegu ´ıa C, Reina R, Neira J, Ceraso DH, Orlandi C, Valentini R, Tiribelli N, Brizuela M, Balasini C, Mare S, Domeniconi G, Ilutovich S, Gomez A, Giuliani J, Barrios C, Valdez P (2010) Pandemic 2009 Influenza A(H1N1) in Argentina: a study of 337 patients on mechanical ventilation. Am J Respir Crit Care Med.

• Rello J, Rodr ´ıguez A, Iban ~ez P, Socias L, Cebrian J, Marques A, Guerrero J, Ruiz-Santana S, Marquez E, Del Nogal- SaezF,Alvarez-LermaF,Mart ´ınezS, Ferrer M, Avellanas M, Granada R, Marav ´ı-PomaE,AlbertP,SierraR, Vidaur L, Ortiz P, Prieto del Portillo I, Galva ´ n B, Leo ´ n-Gil C, H1N1 SEMICYUC Working Group (2009) Intensive care adult patients with severe respiratory failure caused by influenza A(H1N1)v in Spain. Crit Care 13:R148.

### A237 Quality of life in long-term survivors of the acute respiratory distress syndrome

#### B. Llorente Ruiz^1^, J. Lujan Varas^1^, R. Molina Montero^1^, C. Pintado Delgado^1^, O. Navarrete^2^, M. Vazquez Mezquita^2^, E. Alonso Peces^2^

##### ^1^Hospital Universitario Príncipe de Asturias, Intensive Care Unit, Madrid, Spain, ^2^Hospital Universitario Príncipe de Asturias, Pneumology, Madrid, Spain

###### **Correspondence:** B. Llorente Ruiz – Hospital Universitario Príncipe de Asturias, Intensive Care Unit, Madrid, Spain

**Introduction**

In the last years, there has been an enormous research effort into the knowledge of physiopathology of acute respiratory distress syndrome (ARDS) which, together with the advances in critical care, has led to a reduction in mortality. With declining fatality rates, the interest of researchers has shifted from mortality statistics to other outcome parameters such as health-related quality of life (HRQL) after ARDS.

**Objective**

The aim of our study was to investigate HRQL in long-term survivors (longer than 2 years) after ARDS, all of them ventilated with the low-tidal volume protocol.

**Method**

A total of 54 patients more than 2-year survival after an ADRS episode were identified between June 2008 and May 2009. 8 patients were excluded because of pulmonary o cardiovascular previous disease. 15 patients refused to participate. Finally, 31 previously healthy patients participated in this study. The medical records were searched for demographic data, smoke status, ventilator data, length of ICU and hospital stays and measures of severity of illness such APACHEII, LIS and SOFA. HRQL was evaluated using the St George's Respiratory Questionnaire (SGRQ).

SGRQ is a self-administered questionnaire that measures perceived quality of life and impairments in health due to respiratory disease. It contains 76 items in three domains (symptoms, activity, and impacts on daily life) and a summary or total score. Scores range from 0 (good health) to 100 (poor health). The questionnaire has been validated in a Spanish population.

**Results**

The results of the demographic and clinical variables are shown in Table 76.

Regarding the evaluation of HRQL by SGRQ, the mean scores were slightly higher in all domains with regard to the reference values from Spanish population by age and sex, indicating subjective respiratory problems with an impact on daily life. Only 7 of our 31 patients (22.6 %) had scores normal in all domains and in the total scores. 18 patients (58.1 %) had higher scores in all domains. The other 6 patients (19.3 %) had a higher score in some of the domains. Table 77

**Conclusions**

· ARDS has a negative impact on the quality of life of survivors detectable even beyond two years after the acute episode.

· Domains of activity and symptoms are the most affected in patients who survive ADRS.

**References**

1. Herridge MS. Long-term outcomes after critical illness. Curr Opin Crit Care 2002 Aug; 8(4):331-336.

2. Hopkins RO,et al. Two-year cognitive, emotional, and quality-of-life outcomes in acute respiratory distress syndrome. Am J Respir Crit Care Med 2005 Feb 15;171(4):340-347.

3. Ferrer M, et al. Interpretation of quality of life scores from the St George´s Respiratory Questionnaire. Eur Respir J 2002 Mar;19(3):405-413.

4. Ferrer M, et al. Validity and reliability of the St George´s Respiratory Questionnaire after adaptation to a different language and culture: the Spanish example. Eur Respir J 1996 Jun;9(6):1160-1166.

**Table 76 (abstract A237).** Demographics and clinical data

Age	53.9 ± 16.3
Male	22 (73.3%)
Ever smoke	21 (67.7%)
Days of machanical ventilation	20 ± 18.9
Days in ICU	24 ± 19.3
Days in hospital	60.38 ± 44.04
Follow-up period (months)	65.8 ± 21.1
APACHE II	20,38 ± 7,42
SOFA	10 ± 3,44
LIS at admission	3,06 ± 0,63

**Table 77 (abstract A237).** HRQL by SGRQ

Total	24.1 ± 19.6
Symptoms	29.1 ± 26.5
Activity	32 ± 25.3
Impacts on Daily Life	17.6 ± 17.4

### A238 Positive end expiratory pressure titration at bedside using electrical impedance tomography

#### M.A.M. Nakamura^1^, L.A. Hajjar^2^, F.R.B.G. Galas^3^, T.A. Ortiz^1^, M.B.P. Amato^1^

##### ^1^Hospital das Clínicas da Faculdade de Medicina da Universidade de São Paulo, Pulmonology Division, Heart Institute, São Paulo, Brazil; ^2^Hospital das Clínicas da Faculdade de Medicina da Universidade de São Paulo, Cardiology Division, Heart Institute, São Paulo, Brazil; ^3^Hospital das Clínicas da Faculdade de Medicina da Universidade de São Paulo, Anesthesiology Division, Heart Institute, São Paulo, Brazil

###### **Correspondence:** M.A.M. Nakamura – Hospital das Clínicas da Faculdade de Medicina da Universidade de São Paulo, Pulmonology Division, Heart Institute, São Paulo, Brazil

**Introduction**

Appropriated level of positive end expiratory pressure (PEEP) can improve oxygenation and avoid ventilator induced lung injury. Many Method for setting PEEP have been proposed, but they are longstanding and difficult to perform in clinical practice, or based in oxygenation table, ignoring individual mechanical characteristics. We propose a Method to titrate PEEP at bedside, that considers regional respiratory mechanical differences, in a fast and practical way.

**Objectives**

Compare agreement between a PEEP titration Method performed fast (about 7 minutes) and slow (about 45 minutes).

**Method**

Eleven mechanically ventilated patients after elective cardiac surgery with moderated ARDS according to the Berlin criteria, but using a P/F ≤ 250 mmHg, received descending PEEP titration in steps of 2cmH_2_O from 23 to 5cmH_2_O performed in two randomly ways. One of them was performed with steps of 40 seconds (total time less than 7 minutes), and the other, in steps of 4 minutes (total time of 45 minutes). Each PEEP trials were preceded by standard (or maximal) alveolar recruitment manouver. PEEP trials were performed with PEEP titration tool available in Electrical Impedance Tomography (EIT) device (ENLIGHT 1800, TIMPEL). This tool [1] provides a report with a functional map ventilation, demonstrating the amount of collapsed and hyperdistension tissue for each step of PEEP. The minor PEEP with less than 5 % of collapse was chosen as the optimal PEEP. This is part of a translational research, in the experimental setting, 3 pigs with establish lung injury received a descendent PEEP titration using EIT monitoring and dynamic Computed Tomography (CT). The CT was performed to evaluate the correlation of collapsed tissue amount in each step of PEEP between CT and EIT.

**Results**

There were no differences in optimal PEEP titrated with fast and slow titration Method (13,20 ± 3,58cmH_2_O *versus* 13,40 ± 3,37cmH_2_O, p =0,727). The mean difference between two Method was 0,20 ± 1,75cmH_2_O with limits of agreement of -3,23 to 3,63cmH_2_O. Analysing the amount of collapse during entire titration, there was no significant differences between the fast and the slow titration (p = 0,401), however there were differences between each PEEP steps (p < 0,001) and there was no interaction between the fast and the slow titration groups and PEEP steps (p = 0,997). There was a good correlation between collapsed tissue provided by the EIT and CT (R^2^ = 0,97).

**Conclusions**

There is agreement between the fast and the slow titration Method and the PEEP titration can be performed easily in less than 7 minutes with EIT monitoring, at bedside.

**References**

1. Costa, E.L., et al., *Bedside estimation of recruitable alveolar collapse and hyperdistension by electrical impedance tomography.* Intensive Care Med, 2009. **35**(6): p. 1132-7.

**Grant acknowledgment**

Financial support by grants from “Fundação de Amparo à Pesquisa do Estado de São Paulo (FAPESP)”.

### A239 Functional imaging of lung macrophage inflammation during high volume ventilation using PET-CT

#### L. Bitker^1,2^, N. Costes^3^, D. Le Bars^3^, F. Lavenne^3^, D. Mojgan^4,5^, J.-C. Richard^1,2,5^

##### ^1^Hospices Civils de Lyon, Hôpital de la Croix-Rousse - Service de réanimation médicale, Lyon, France; ^2^CNRS UMR 5220 - INSERM U1206, CREATIS, Lyon, France; ^3^CERMEP, Bron, France; ^4^Hospices Civils de Lyon, Hôpital de la Croix-Rousse - Service d'anatomopathologie, Lyon, France; ^5^Université de Lyon, Université Lyon I, Lyon, France

###### **Correspondence:** L. Bitker – Hospices Civils de Lyon, Hôpital de la Croix-Rousse - Service de réanimation médicale, Lyon, France

**Introduction**

Ventilation Induced Lung Injury (VILI) is associated with an increased mortality in ARDS [1]. Through mechanical stress and strain of the lung, VILI triggers an inflammatory response, which may be spatially characterized by imaging techniques such as Positron Emission Tomography combined with computed tomography (PET/CT). ^11^C-PK11195 is a PET radiotracer with short half-life and potential to repeatedly quantify macrophage inflammation.

**Objectives**

To evaluate ^11^C-PK11195 lung uptake and its association with mechanical strain assessed with CT, as well as its relation with both macrophage lung recruitment and histologic injury.

**Method**

VILI was performed in 5 anesthetized pigs by increasing the tidal volume (VT) to obtain a transpulmonary pressure (TPP) between 35 and 40 cmH_2_O under zero end-expiratory pressure. CT and PET acquisitions, were performed before (T1) and after 4 hours of high volume ventilation (T2), and measurements were performed globally on the whole lungs, and regionally by partitioning the lung in 4 regions defined by the cephalo-caudal and the antero-posterior planes. ^11^C-PK11195 uptake was quantified using the Standardized Uptake Value (SUV), corrected for the fraction of tissue in each lung region (as assessed in CT). Regional strains (dynamic and static) were estimated by CT analysis. After euthanasia, a semi-quantitative lung injury score and macrophages recruitment were quantified in lung samples.

**Results**

Between T1 and T2, VT and TPP increased from 6.0 ± 0.1 to 49.4 ± 2.9 ml/kg and from 9 ± 2 to 38 ± 4 cmH_2_O, respectively. Between T1 and T2, global ^11^C-PK11195 SUV and global dynamic strain increased significantly from 1.83 ± 0.58 to 2.97 ± 0.53, and from 0.36 ± 0.03 to 2.06 ± 0.23, respectively, whereas static strain did not change significantly. Regional ^11^C-PK11195 SUV significantly increased between T1 and T2, without significant inter-regional differences, while regional dynamic strain increased after VILI, with significant inter-regional differences between antero-caudal and postero-caudal regions. Regional static strain differed neither between T1 and T2, nor between regions. In multivariate analysis, regional dynamic strain was independently associated with regional SUV (p = 0.04). Histologic analysis showed greater alveolar damage in the caudal regions (p < 0.01). SUV was positively correlated with macrophages recruitment (p = 0.03).

**Conclusions**

^11^C-PK11195 is a macrophage-specific PET radiotracer whose lung uptake is independently associated with dynamic strain and macrophage lung recruitment in a high-volume VILI model.

**References**

1. ARDS Network: Ventilation with lower tidal volumes as compared with traditional tidal volumes for acute lung injury and the acute respiratory distress syndrome. *N Engl J Med* 2000, 342:1301-1308.

**Grant acknowledgment**

Research founded by the French society of intensive care medicine and *La Fondation pour la Recherche Médicale* (DEA20140630499).

### A240 Determinants of energy load in acute respiratory distress syndrome patients

#### C. Chiurazzi^1^, M. Cressoni^2^, D. Massari^2^, M. Guanziroli^2^, G. Vergani^2^, M. Gotti^2^, M. Brioni^2^, I. Algieri^2^, P. Cadringher^2^, T. Tonetti^3^, D. Chiumello^4,5^, L. Gattinoni^3^

##### ^1^Università degli Studi di Milano, Dipartimento di Fisiopatologia Medico-Chirurgica e dei Trapianti, Milano, Italy; ^2^Università degli Studi di Milano, Dipartimento di Fisiopatologia Medico Chirurgica e dei Trapianti, Milano, Italy; ^3^Georg-August-University Goettingen, Anesthesiology and Intensive Care Medicine, Goettingen, Germany; ^4^Policlinico Di Milano, Dipartimento di Anestesia, Rianimazione, Urgenza ed Emergenza, Milano, Italy; ^5^Plug Working Group, Milan, Italy

###### **Correspondence:** C. Chiurazzi – Università degli Studi di Milano, Dipartimento di Fisiopatologia Medico-Chirurgica e dei Trapianti, Milano, Italy

**Introduction**

Experimental evidence suggest that ventilator induced lung injury (VILI) depends on the energy load applied to the respiratory system, which in healthy lungs encompasses tidal volume, respiratory rate and flow. [1]

**Objectives**

To investigate if putative mechanisms of VILI (lung strain [2], lung inhomogeneities [3], collapse and decollapse [4]) are associated with an increased energy load per breath.

**Method**

Patients underwent a CT scan at PEEP 5 cmH_2_O end-expiration and a second CT scan at end-inspiration. Airway and esophageal pressure were recorded during tidal ventilation. Energy load per breath (Joule) was defined as the area between the inspiratory limb of the delta-transpulmonary pressure (x-axis)-volume curve and the volume axis (y). Tidal strain was defined as tidal volume (ml)/gas volume at PEEP 5 cmH_2_O (ml); lung inhomogeneities were computed as previoulsy described [2]. Intratidal collapse and decollapse was defined as the difference in not inflated tissue between end-inspiration and end-expiration expressed as fraction of total lung weight.

**Results**

**Fig. 101:**
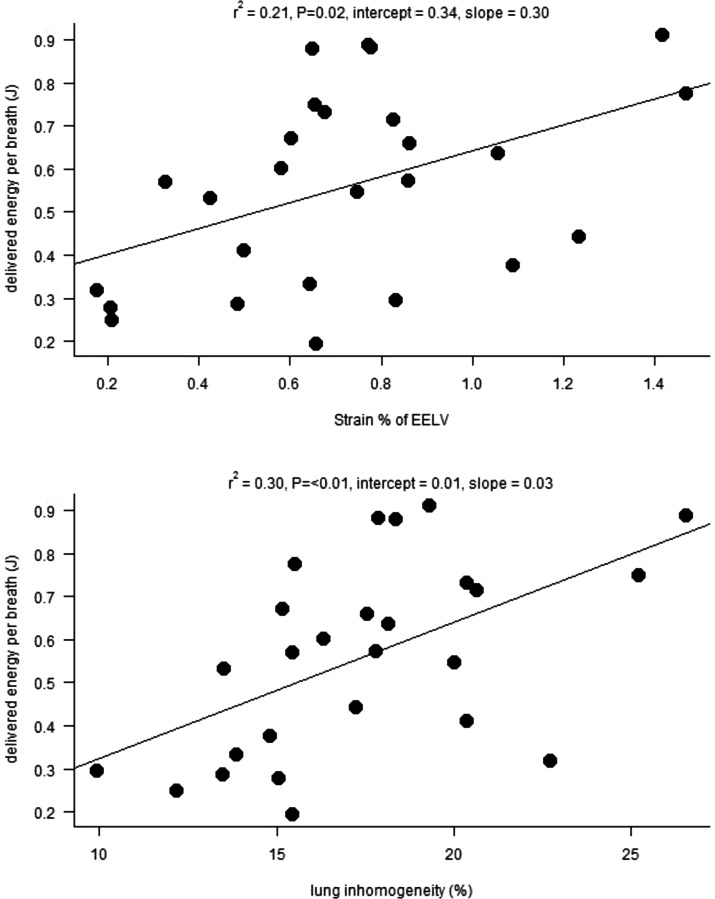
(abstract A240).

Twenty-seven ARDS patients were studied at PEEP 5 cmH_2_O. Age 58 [44-72] years (median [IQ range]), BMI 25 [22-29] kg/m^2^, PaO_2_/FiO_2_ 105 [83-168], PaCO_2_ 44 [40-51] mmHg, tidal volume 7 [5-8] ml/Kg IBW. Energy delivered per breath (J) was significantly related to lung strain (Fig. 101, upper panel) and inhomogeneity (Fig. 101, lower panel); the relationship between delivered energy and intratidal collapse-decollapse did not reach statististical significance (r^2^ = 0.10, p = 0.11).

**Conclusions**

Greater lung strain and lung inhomogeneities increase the energy delivered to the respiratory system by the tidal volume.

**References**

[1] Cressoni M, Anesthesiology, 2016

[2] Chiumello, Am J Respir Crit Care Med, 2008

[3] Cressoni M, Am J Respir Crit Care Med, 2014

[4] Caironi, Am J Respir Crit Care Med, 2011

**Grant acknowledgment**

ESICM Clinical Science Award 2014 to MC

### A241 The impact of frailty on weaning status and outcomes of critically ill patients receiving invasive mechanical ventilation

#### A. Zerman^1^, M. Türkoğlu^1^, G. Arık^2^, F. Yıldırım^1^, Z. Güllü^1^, I. Kara^1^, N. Boyacı^1^, B. Basarık Aydoğan^1^, Ü. Gaygısız^1^, K. Gönderen^1^, G. Aygencel^1^, M. Aydoğdu^1^, Z. Ülger^2^, G. Gürsel^1^

##### ^1^Gazi University Faculty of Medicine, Department of Critical Care Medicine, Ankara, Turkey; ^2^Gazi University Faculty of Medicine, Department of Geriatrics, Ankara, Turkey

###### **Correspondence:** A. Zerman – Gazi University Faculty of Medicine, Department of Critical Care Medicine, Ankara, Turkey

**Introduction**

Invasive mechanical ventilation (IMV) is frequently applied in critically ill elderly patients. Age, comprehensive geriatric assessment (CGA) parameters and recently frailty were reported as general prognostic factors in these patients [1, 2]. However, there aren't enough studies about the prognostic factors specifically in patients with IMV.

**Objectives**

To investigate the clinical characteristics of elderly patients receiving IMV and to show the impact of age, CGA parameters and particularly frailty on weaning success and outcomes of these patients.

**Method**

The patients, >50 years old, admitted to the medical and pulmonary intensive care units (ICU) in Gazi University Hospital were prospectively included. Patients without consent and stayed < 24 hours in the ICU were excluded. Frieds, Clinical and Edmonton frailty scores (FS) were calculated and CGA parameters were assessed by the geriatrician. The length and the result of weaning were recorded. The weaning was classified as simple, difficult and prolonged weaning.

**Results**

IMV was applied in %49 of 180 patients. While the age had no impact on the need of IMV(p = 0.407), frailty defined by Edmonton FS was an independent factor for IMV; increasing the need approximately 3 times (p = 0.023). The presence and the degree of frailty according to all FSs and CGA parameters had no impact on weaning and mortality in patients with IMV(p > 0.05). In the whole study group, when the patients with and without fraility according to the Edmonton FS were compared, disease severity scores (APACHE and SOFA scores), duration of weaning were similar in both groups (p > 0.05), but the comorbidities in the ICU such as development of infection and septic shock, frequency of renal replacement therapy and and mortality were higher in the frail patients (p = 0.03, p = 0.04, p = 0.02 and p = < 0.01, respectively).

**Conclusions**

The presence of frailty in critically ill elderly patients increased the need for IMV. After initiation of IMV, frailty had no impact on weaning and mortality in these patients. On the other hand, as the need for IMV, comorbidities in the ICU and therefore overall mortality are found to be incresead with fraility.

**References**

1. Avelino-Silva TJ, Farfel JM, Curiati JA, Amaral JR, Campora F, Jacob-Filho F. Comprehensive geriatric assessment predicts mortality and adverse outcomes in hospitalized older adults BMC Geriatrics 2014; 14: 129.

2. Bagshaw SM, Stelfox HT, McDermid RC, Rolfson DB, Tsuyuki RT, Baig N, ArtiuchB, Ibrahim Q,Stollery DE, Rokosh E, Majumdar SR. Association between frailty and short- and long- term outcomes among critically ill patients: a multicentre prospective cohort study. *CMAJ* 2014. 2014186:E95-102.

**Grant acknowledgment**

We didn't receive any grants and we don't have any acknowledgment.

### A242 Prone positioning as a bridge to recovery from refractory hypoxemia following lung transplantation

#### J. Riera^1,2,3^, C. Maldonado Toral^1^, C. Mazo^1^, M. Martínez^1^, J. Baldirà^1^, L. Lagunes^1^, A. Roman^3,4^, M. Deu^5^, J. Rello^1,2,3^, D.J. Levine^6^

##### ^1^Vall d'Hebron University Hospital, Critical Care Department, Barcelona, Spain; ^2^Vall d'Hebron Research Institut, Barcelona, Spain; ^3^CIBERES, Instituto de Salud Carlos III, Madrid, Spain; ^4^Vall d´Hebrón University Hospital, Pneumology, Barcelona, Spain; ^5^Vall d´Hebrón University Hospital, Thoracic Surgery, Barcelona, Spain; ^6^University of Texas Health Science Center at San Antonio, Department of Medicine, Division of Pulmonary and Critical Care Medicine, San Antonio, TX, USA

###### **Correspondence:** C. Maldonado Toral – Vall d'Hebron University Hospital, Critical Care Department, Barcelona, Spain

**Introduction**

Refractory hypoxemia is the leading cause of early mortality following lung transplantation (LT). Rescue therapies, such as extracorporeal membrane oxygenation, have been shown to be useful for supporting LT recipients with refractory hypoxemia. Prone position (PP) is a low cost measure that has been shown to improve gas interchange in patients with severe acute respiratory distress syndrome. Major thoracic surgery has been considered a contraindication for its implementation. Thus, to date there is no published evidence of the beneficial effects of PP as a bridge to recovery for patients with refractory hypoxemia after LT.

**Objectives**

The primary objective was to assess the gas interchange improvement with PP. The secondary objective was to compare the outcomes of these patients with those of the general population of our LT recipients.

**Method**

Prospectively collected data from 131 consecutive adult patients undergoing LT between January 2013 and December 2014 were evaluated. Indications, associated complications, time to initiation and duration of the maneuver were analyzed and the effects of PP on gas interchange were evaluated. Finally, outcomes in this cohort were compared against the rest of LT recipients. Continuous data are reported as median and interquartile range(IQR) and categorical data as numbers and percentages. Differences between categorical variables were assessed with the Chi-square test and continuous variables with the Mann-Whitney test. The Student “t” test was used to evaluate the effects of PP on gas interchange.

**Results**

Twenty-two patients received PP. The maneuver was more frequently implemented within the first 72 hours (68.2 %) and its main indication was primary graft dysfunction. The maneuver was maintained during a median of 21 hours. After PP, the PaO_2_/F_I_O_2_ ratio significantly increased from 81.0 mmHg (IQR 71.5-104.0) to 220.0 (IQR 160.0-288.0) (P < 0.001). No complications related with the technique were reported. Patients who underwent the maneuver had longer hospital stay (50.0 days [IQR 36.0-67.0] vs 30.0 [IQR 23.0-56.0],P = 0.006) than the rest of the population. No differences were found comparing either one-year mortality (9.1 % vs 15.6 %; P = 0.740) or one-year graft function (FEV_1_ of 70.0 [IQR 53.0-83.0] vs 68.0 [IQR 53.5-80.5], P = 0.469).

**Conclusions**

PP was found to be a safe and successful therapy for refractory hypoxemia after LT and should be considered as an option in treating these patients.

**References**

1. -Fuehner T, Greer M, Welte T, Gottlieb J. The lung transplant patient in the ICU. Curr Opin Crit Care 2012; 18:472-478.

2. -Bermudez CA, Adusumilli PS, McCurry KR et al. Extracorporeal membrane oxygenation for primary graft dysfunction after lung transplantation: long-term survival. Ann Thorac Surg 2009; 87(3):854-60.

3. -Guérin C, Reignier J, Richard JC et al; PROSEVA Study Group. Prone positioning in severe acute respiratory distress syndrome. N Engl J Med 2013; 368(23):2159-68.

## NUTRITION MODALITIES AND MONITORING

### 0243 Association of iron status with the risk of bloodstream infection in a general Norwegian population. The HUNT Study

#### R.M. Mohus^1^, Å. Askim^2^, J. Paulsen^3^, A. Mehl^4^, A.T. Dewan^5^, J.K. Damås^2^, E. Solligård^2^, B.O. Åsvold^6^, Mid-Norway Sepsis Research Center

##### ^1^Norwegian University of Science and Technology, Department of Circulation and Medical Imaging, Trondheim, Norway; ^2^Norwegian University of Science and Technology, Department of circulation and medical imaging, Trondheim, Norway; ^3^Norwegian University of Science and Technology, Centre of Molecular Inflammation Research, Department of Cancer Research and Molecular Medicine, Trondheim, Norway; ^4^Norwegian University of Science and Technology, Department of Cancer Research and Molecular Medicine, Trondheim, Norway; ^5^Yale University School of Public Health, Department of Chronic Disease Epidemiology, New Haven, CT, USA; ^6^Norwegian University of Science and Technology, Department of Public Health and General Practice, Trondheim, Norway

###### **Correspondence:** R.M. Mohus – Norwegian University of Science and Technology, Department of Circulation and Medical Imaging, Trondheim, Norway

**Background**

Iron is an essential element for both proper immune function and for the growth of most human pathogens. Alterations in iron status may affect the immune system and the risk of infections. No population-based studies have investigated the association between markers of iron status and the risk of bloodstream infection (BSI).

**Objectives**

We assessed the associations of serum iron concentration (SI), total iron binding capacity (TIBC) and transferrin saturation percent (TS) with the risk of BSI and fatal BSI (death within 30 days after detection of a BSI).

**Method**

We studied 64033 participants with baseline measurements of SI, TIBC and TS in the second survey of the population-based HUNT Study in Nord-Trøndelag County, Norway (HUNT2, 1995-97). SI, TIBC and TS were categorized as low (<2.5-percentile), high (>97.5 percentile) and quintiles of values between the 2.5 and 97.5 percentiles. Incident BSIs through 2011 were identified though linkage to prospectively recorded information at the local and regional hospitals. For each measure of iron status, we assessed the risk of a first-time BSI and fatal BSI using Cox proportional hazards regression, with the middle quintile as the reference. The primary analyses were adjusted for age and sex. Additionally, we adjusted for self-reported comorbid conditions; lung disease, cardiovascular disease, cancer, kidney failure, diabetes and body mass index at baseline.

**Results**

During a median follow-up of 15 years, 1840 persons had at least one episode of BSI, and 396 experienced a fatal BSI. In age- and sex-adjusted analyses, BSI risk was increased among participants with indices of iron deficiency, either low SI (HR 1.77, 95 % CI 1.39-2.26), low TS (HR 1.62, CI 1.24-2.11) or high TIBC (HR 1.42, CI 1.03-1.95). After adjustment for comorbidities, the associations remained similar for low SI (HR 1.79, CI 1.38-2.31) and low TS (HR 1.59, CI 1.19-2.12), but attenuated for high TIBC (HR 1.22, CI 0.85-1.75). The corresponding HRs of fatal BSI were 1.60 (CI 0.90-2.83) for low SI, 1.43 (CI 0.65-3.11) for low TS and 2.21 (CI 1.15 - 4.25) for high TIBC. We found no increase in BSI risk related to indices of high iron status.

**Conclusions**

In this large population-based cohort study, indices of low iron status were associated with increased risk of BSI. Iron is a crucial element in our immune response and these findings suggest that alterations in this fine tuned system could influence the risk of BSI.

**Grant Acknowledgement**

The study was supported by a grant from the Liasion Committee between the Central Norway Regional Health Authority and the Norwegian University of Science and Technology.

### A244 Associations of obesity and lifestyle with the risk and mortality of bloodstream infection in a general population: a 15-year follow-up study of 64,000 individuals in the HUNT Study

#### J. Paulsen^1^, Å. Askim^2^, R.M. Mohus^2,3^, A. Mehl^4,5^, A. DeWan^6^, E. Solligård^2,3^, J.K. Damås^1,7^, B.O. Åsvold^8,9^

##### ^1^Norwegian University of Science and Technology, Centre of Molecular Inflammation Research, Trondheim, Norway; ^2^Norwegian University of Science and Technology, Department of Circulation and Medical Imaging, Trondheim, Norway; ^3^St Olavs University Hospital, Clinic of Anaesthesia and Intensive Care, Trondheim, Norway; ^4^Norwegian University of Science and Technology, Institute of Cancer Research and Molecular Medicine, Trondheim, Norway; ^5^Nord Trøndelag Hospital Trust, Levanger Hospital, Department of Medicine, Levanger, Norway; ^6^Yale University School of Public Health, New Haven, CT, USA; ^7^St Olavs University Hospital, Department of Infectious Diseases, Trondheim, Norway; ^8^Norwegian University of Science and Technology, Department of Public Health and General Practice, Trondheim, Norway; ^9^St Olavs University Hospital, Department of Endocrinology, Trondheim, Norway

###### **Correspondence:** J. Paulsen – Norwegian University of Science and Technology, Centre of Molecular Inflammation Research, Trondheim, Norway

**Introduction**

Bloodstream infection (BSI) causes considerable morbidity and mortality. As emerging antibiotic resistance seriously threatens global public health, primary prevention of bacterial infections should be a priority. Lifestyle factors are of particular interest since they are modifiable, and optimization could reduce the population-level burden of BSI.

**Objectives**

To assess the associations of smoking, obesity, alcohol intake and physical inactivity with the risk of incident and fatal BSI.

**Method**

In a prospective population-based cohort study, 65236 participants of the HUNT2 Survey (1995-97) in Norway were followed up through 2011 by linkage to prospectively recorded information on BSI at the local and regional hospitals. Using Cox regression, we estimated age- and sex- adjusted hazard ratios (HR) of a first-time BSI and fatal BSI (death within 30 days after BSI) by baseline body mass index (BMI) measurements and self-reported smoking habits, leisure time physical activity and alcohol intake. In additional analysis we also adjusted for education and lifestyle factors.

**Results**

During 14.8 years of follow-up, 1844 (2.9 %) participants experienced at least one episode of BSI, and 396 (0.62 %) experienced a fatal BSI. Obesity was dose-dependently associated with increased risk of BSI and fatal BSI. Compared with normal weight participants (BMI 18.5-24.9 kg/m^2^), the age- and sex-adjusted risk of a first-time BSI was 28 % (95 % confidence interval (CI) 11-47 %) higher at BMI 30.0-34.9 kg/m^2^, 83 % (95 % CI 46-129 %) higher at BMI 35.0-39.9 kg/m^2^ and 200 % (95 % CI 111-328 %) higher at BMI ≥ 40.0 kg/m^2^. Correspondingly, the risk of fatal BSI was 34 % (95 % CI -1-81 %) higher at BMI 30.0-34.9 kg/m^2^, 144 % (95 % CI 55-286 %) higher at BMI 35.0-39.9 kg/m^2^, and 300 % (95 % CI 94-727 %) higher at BMI ≥ 40.0 kg/m^2^, compared with normal weight participants. Current smokers had a 55 % (95 % CI 37-75 %) higher risk of BSI and a 77 % (95 % CI 36-130 %) higher risk of fatal BSI compared with never-smokers. Inactive participants had a 65 % (95 % CI 37-101 %) higher risk of BSI and a 105 % (95 % CI 35-212 %) higher risk of fatal BSI compared with the most physically active participants. The findings were essentially unchanged after adjustment for education and lifestyle factors.

**Conclusions**

This study underscores that maintaining a healthy lifestyle with normal weight, non-smoking and physical activity may contribute to the prevention of invasive bacterial infections.

**Grant acknowledgment**

This work was supported by a grant from the Liaison Committee between the Central Norway Regional Health Authority (RHA) and the Norwegian University of Science and Technology (NTNU).

### A245 Is obesity paradox real or not? The role of leptin in septic shock patients

#### O. Aktepe, A. Kara, H. Yeter, A. Topeli

##### Hacettepe University, Ankara, Turkey

###### **Correspondence:** A. Topeli – Hacettepe University, Ankara, Turkey

**Objective**

Recent observational studies showed that obese critically-ill patients have an unexpectedly reduced risk of death, having low mortality rate in the course of septic shock, as well. This situation is described as “obesity paradox”. Leptin, a hormone made by white adipose cells regulating energy balance by inhibiting hunger and which rises proportionally to body weight has also been shown to have a role in immune modulation. Therefore, leptin could play the pivot role in case of obesity paradox. The aim of this study was to investigate the relationship between mortality and body mass index (BMI) in patients with septic shock. In addition, we tested association of leptin levels with BMI and mortality.

**Method**

Between September 2014 and January 2016, 52 patients with septic shock were included in the study. As a control group, 27 healthy people with BMI (kg/m^2^) similar to patients' BMI values were included. Patients who had uncontrolled diabetes mellitus, malignancy and who received corticosteroids chronically prior to ICU admission were excluded. Patients were categorized into five groups according to BMI (group 1 (n = 6) ≤18; group 2 (n = 14) 18.1-24.9; group 3 (n = 19) 25-29.9; group 4 (n = 10) 30-39.9; grup 5 (n = 3) ≥40 kg/m^2^). We measured leptin levels at 9:00 a.m. after patient has been diagnosed with septic shock.

**Results**

The median (min-max) age of 52 patients was 68 (19-88). 25 of these patients were male. The median APACHE II score was 28.5 (11-45). Median leptin level was 0.2 (0.1-54.5) ng/ml. Control patients' median age, BMI and leptin levels were [62 (24-82); 26.8 (18.8-44.8) and 4 (0.1-22.8)], respectively. 27 of the 52 septic shock patients died within 28 days. The APACHE II scores were similar between the survivor and non-survivor groups (28 (11-40), 29 (11-45), p = 0.37). The surviving patients had lower but statistically insignificant BMI values compared to non-survivors (24.9 (17.8-35.1), 27.2 (16.5-49.0), p = 0.12). 28th day mortality rate in 5 groups stratified according to BMI was similar (p = 0.50). Leptin levels were similar in surviving and non-surviving patients (0.13 (0.1-11.0), 0.25 (0.1-54.5), p = 0.23). However, there was a difference among 5 BMI groups in terms of leptin levels (median leptin: group 1 0.28, group 2 0.16, group 3 0.22, group 4 0.37, group 5 4.80; p = 0.046), such that group 5 (BMI ≥ 40) had the highest leptin level compared to other 4 groups (p = 0.047). Control patients have higher leptin levels than patients with septic shock (p < 0.001). There was a statistically significant correlation between BMI and leptin level in all patients (n = 79, r = 0.49, p < 0.001).

**Conclusion**

Our study showed that increased BMI was not related to increased survival. Leptin levels were lower in patients with septic shock compared to control patients. Although leptin levels correlated with BMI, there was no difference in leptin levels between surviving and non-surviving patients.

### A246 Relationship between muscle mass and force in ICU long-stayers

#### M. Norrenberg^1^, M. Devroey^2^, H. Khader^2^, J.-C. Preiser^2^

##### ^1^Erasme, Brussels, Belgium, ^2^Erasme Hospital, ICU, Brussels, Belgium

###### **Correspondence:** M. Norrenberg – Erasme, Brussels, Belgium

**Introduction**

ICU-acquired weakness is related to loss of muscle mass and function, especially in long-stayers. Both factors can be assessed at the bedside using independent techniques, but the correlation between mass and function is unknown

**Objectives**

To evaluate any correlation between muscle mass and force in long-stay ICU patients.

**Method**

Muscle mass and force were assessed on admission and at the end of the ICU stay in patients with an expected length of stay (LOS) of at least 5 days. Mass was measured from anthropometric variables (skinfolds of calf and arm) using the Lee formula (1) and bioelectric impedance (phase angle (PhA)) at 50 Hz. Force was measured using the Medical Research Council (MRC) score (2) and dynamometry (handgrip test). Effect of time over the ICU stay was assessed using a Student's t test. Correlations between mass and force, and between these variables and the APACHE II score, the duration of ICU stay and of mechanical ventilation were investigated.

**Results**

Eighteen patients (age 56 ± 16 years, 12 male, APACHE II 20 ± 5) were included. During the ICU stay (17 ± 12 days), muscle mass assessed by skin folds and by PhA at 50 Hz decreased from 26.8 ± 5.4 kg to 25.6 ± 5.4 kg (p < 0.003) and from 4.0 ± 1.6 to 3.4 ± 1.4 (NS), respectively. Likewise, force evaluated with MRC score decreased from 52 ± 2 to 42 ± 5 (NS). There was a correlation between the percentage change in mass and in force (r =0.57, p < 0.01), between the percentage change in mass and length of ICU stay (r = - 0.79; p < 0.0001) and between the durations of mechanical ventilation and ICU stay (r = 0.89; p < 0.0001). The APACHE II score was negatively correlated with force measured using the handgrip test (r = -0.67; p = 0.002), with the MRC score (r = -0.765; p < 0.0001) and with muscle mass evaluated by PhA (r = -0.665; p = 0.004).

**Conclusions**

Muscle mass and function decrease during a long ICU stay, and these changes are correlated. Use of these bedside Method to guide therapeutic management needs to be assessed.

**References**

1 Lee R, Wang Z, Heo M, Ross R, Janssen I, and Heymsfield S.B, Total-body skeletal muscle mass: development and cross-validation of anthropometric prediction models, Clin Nutr, 72:796-803, 2000.

2 De Jonghe B, Bastuji-Garin S,Sharshar Tarek, Outin H, Brochard L Does ICU-acquired paresis lengthen weaning from mechanical ventilation? Intensive Care Medicine 2004;30:1117-1121

### A247 Application of prone position combined with post- pylorus feeding for acute respiratory distress syndrome caused by severe pneumonia after renal transplantation

#### Z. Tang, C. Qiu, L. Tong, C. Cai

##### The First Affiliated Hospital of Sun Yat-Sen University, Guangzhou, China

###### **Correspondence:** Z. Tang – The First Affiliated Hospital of Sun Yat-Sen University, Guangzhou, China

**Introduction**

The mortality of severe acute respiratory distress syndrome(ARDS) caused by pulmonary infection after renal transplantation is high. Prone position ventilation can effectively improve the prognosis of patients with ARDS. Post- pylorus feeding can significantly improve the nutritional intake and reduce the risk of aspiration.

**Objectives**

To evaluate the value of prone position combined with post- pylorus feeding on severe ARDS caused by severe pneumonia after renal transplantation.

**Method**

Prospective observational study in a surgical intensive care unit (10 beds) of a university hospital. Patients met the berlin criteria of severe ARDS after renal transplantation and needed invasive mechanically ventilation were included.12 consective hours of prone position ventilation and post-pylorus feeding tube placement were applied to all included patients

**Results**

In total, 8 patients were included, average 38 ± 10 years, 6 (75 %) were men. The 28-day mortality was 12.5 %(1/8). The ratio of the partial pressure of arterial oxygen(Pao2) to the fraction of inspired oxygen (Fio2)on 1 h,6 h and 12 h after prone positon were improved significantly than before(P < 0.05). The time to reach target feeds was 73 ± 15 hours through Post-pyloric feeding, the rate of aspiration is 0, Nutritional status of all patiens was gradually improved.

**Conclusions**

Prone position combined with post- pylorus feeding can improve the prognosis of severe ARDS cuased by pulmonary infection after renal transplantation.

**Reference**

1. Sun Q, Liu ZH, Chen J, et al. An aggressive systematic strategy for acute respiratory distress syndrome caused by severe pneumonia after renal transplantation. Transpl Int, 2006,19(2):110-6.

2. Tu G, Ju M, Zheng Y, et al. Early- and late-onset severe pneumonia after renal transplantation. Int J Clin Exp Med,2015 ,15;8(1):1324-32.

3. Guérin C, Reignier J, Richard JC, et al.Prone positioning in severe acute respiratory distress syndrome,N Engl J Med, 2013 ,368(23):2159-68.

### A248 Permissive underfeeding of mechanically ventilated septic ICU Patients

#### M. Theodorakopoulou, A. Diamantakis, M. Kontogiorgi, E. Chrysanthopoulou, T. Christodoulopoulou, F. Frantzeskaki, M. Lygnos, O. Apostolopoulou, A. Armaganidis

##### Attikon University Hospital, ICU, Athens, Greece

###### **Correspondence:** M. Theodorakopoulou – Attikon University Hospital, ICU, Athens, Greece

**Introduction**

Nutritional support is an essential part of intensive care unit management However, the appropriate caloric intake for critically ill patients still remains unclear. Permissive underfeeding is based on the fact that provision of 100 % of estimated nutrient requirements may be metabolically and functionally detrimental to some patient populations. It has also been shown that meeting a patient's estimated needs may result in inflammation, cytokine release, and oxidant production while short term restriction of nutrient intake may limit the pathologic processes that occur in critical illness and reduce organ dysfunction.

**Objectives**

To evaluate the effect of permissive underfeeding, as compared with standard protocol enteral feeding, on 28-day mortality among septic mechanically ventilated critically ill patients

**Method**

A single centre study of patients admitted to a 25 bed University Hospital ICU over a period of one year. Demographics, severity of illness scores (APACHE and SOFA), BMI and “Malnutrition Universal Screening Tool” (MUST) were measured upon admission. Daily nutrition requirements were calculated for each patient. Patients were randomly assigned to permissive underfeeding group with caloric goal: 50-70 % of the calculated requirement or standard protocol feeding groups with caloric goal: 80-100 % of calculated requirement. Each patient was monitored for 14 days. The protein intake (1.5 g/kg/day) was maintained the same in both groups. The primary outcome was 28-day mortality. Results are expressed as mean ± SD. P < 0.05 was considered significant.

**Results**

A total of 74 patients (38 men) mechanically ventilated septic patients having a mean(±SD) age of 68.4 ± 18.4 years were studied. All patients met the consensus criteria for sepsis. Baseline characteristics were similar in the two groups. APACHE II and SOFA at study entry were 22 ± 4 and 8 ± 4respectively.The mean height of the patients was 164.0 cm. The mean(±SD) BMI was ≈ 21.5 ± 3.4 kg/m^2^. Obese patients were excluded from the study. During the study period, the permissive-underfeeding group received fewer calories than did the standard-protocol feeding group (962 ± 314 kcal per day vs. 1308 ± 513 kcal per day) achieving caloric requirements of 51 ± 14 % vs. 82 ± 11 %, respectively (P < 0.001). Protein intake was similar in both groups (57 ± 24 g per day and 59 ± 25 g per day, respectively). The 28-day mortality was lower in the permissive-underfeeding group (18.4 %) than in the standard-feeding group (28.9 %). There were no significant differences among the groups with respect to feeding intolerance, diarrhoea or serious adverse events.

**Conclusions**

In critically ill patients, permissive underfeeding may be associated with lower mortality rates than standard-protocol feeding. It is evident that permissive underfeeding is a strategy that should be considered in the ICU septic patient. Employing such an approach may offer clinical benefit.

### A249 Prognostic implication of lower serum total cholesterol level in critically ill medical patients

#### J.Y. Moon^1^, M.R. Park^1^, I.S. Kwon^2^, G.R. Chon^3^, J.Y. Ahn^4^, S.J. Kwon^5^, Y.J. Chang^4^, J.Y. Lee^6^, S.Y. Yoon^6^, J.W. Lee^1^, The Korean Chungcheong Critical Care Research Group

##### ^1^Chungnam National University Hospital, Department of Internal Medicine, Daejeon, Republic of Korea; ^2^Chungnam National University Hospital, Clinical Trials Center, Daejeon, Republic of Korea; ^3^Cheongju St. Mary's Hospital, Department of Internal Medicine, Cheongju, Republic of Korea; ^4^Chungbuk National University Hospital, Department of Internal Medicine, Cheongju, Republic of Korea; ^5^Konyang University Hospital, Department of Internal Medicine, Daejeon, Republic of Korea; ^6^Konkuk University Chungju Hospital, Department of Internal Medicine, Chungju, Republic of Korea

###### **Correspondence:** J.Y. Moon – Chungnam National University Hospital, Department of Internal Medicine, Daejeon, Republic of Korea

**Introduction**

The serum total cholesterol (TC) level is known to be associated with survival of critical illness, especially in sepsis and surgical patients. However, there are only a few studies evaluating TC as a prognostic factor in medical patients.

**Objectives**

The aim of this study was to determine the relationship between TC level and survival in medical patients admitted in intensive care unit (ICU).

**Method**

From September 2013 to February 2014, the data was acquired at the nine intensive care units in the four provincial academic medical centers retrospectively. Statistical analysis was conducted to confirm risk factors with using correlation analysis and logistic regression.

**Results**

In this study, a total of 503 patients were enrolled. The hospital mortality which is based on 28 days after admission was 28.2 %. TC levels derived at specific point of time showed negative correlation with APACHE IV score (initial TC, r = -0.231, p < 0.001; second week TC, r = -0.361, p < 0.001; third week TC, r = -0.327, p < 0.001)

TC level of the second week, C-reactive protein level and status of ARDS were the independent risk factor for mortality in multivariate analysis (hazard ratio (HR) =0.98, p < 0.001; HR = 1.05, p = 0.02, HR = 8.24, p = 0.001, respectively)

**Conclusions**

Lower TC level is a prognostic indicator of hospital mortality in medical patients admitted in ICU. Following up TC level regularly has the advantage of managing critically ill patients.

**References**

1. Chenaud C, Merlani PG, Roux-Lombard P, et al (2004) Low apolipoprotein A-I level at intensive care unit admission and systemic inflammatory response syndrome exacerbation. Crit Care Med 32:632-637.

2. Giovannini I, Boldrini G, Chiarla C, et al (1999) Pathophysiologic correlates of hypocholesterolemia in critically ill surgical patients. Intensive care medicine 25:748-751.

3. Gui D, Spada PL, De Gaetano A, Pacelli F (1996) Hypocholesterolemia and risk of death in the critically ill surgical patient. Intensive care medicine 22:790-794.

4. Gordon BR, Parker TS, Levine DM, et al (2001) Relationship of hypolipidemia to cytokine concentrations and outcomes in critically ill surgical patients. Critical care medicine 29:1563-1568.

5. Chien J-Y, Jerng J-S, Yu C-J, Yang P-C (2005) Low serum level of high-density lipoprotein cholesterol is a poor prognostic factor for severe sepsis*: Critical Care Medicine 33:1688-1693. doi: 10.1097/01.CCM.0000171183.79525.6B

**Table 78 (abstract A249).** Correlation analysis with APACHE IV score

	r	P-value
Initial TC	-0.231	<0.001
Second week TC	-0.361	<0.001
Third week TC	-0.327	<0.001
C-reactive protein	0.277	<0.001
Pro-BNP	0.139	0.037

**Fig. 102 (abstract A249).**
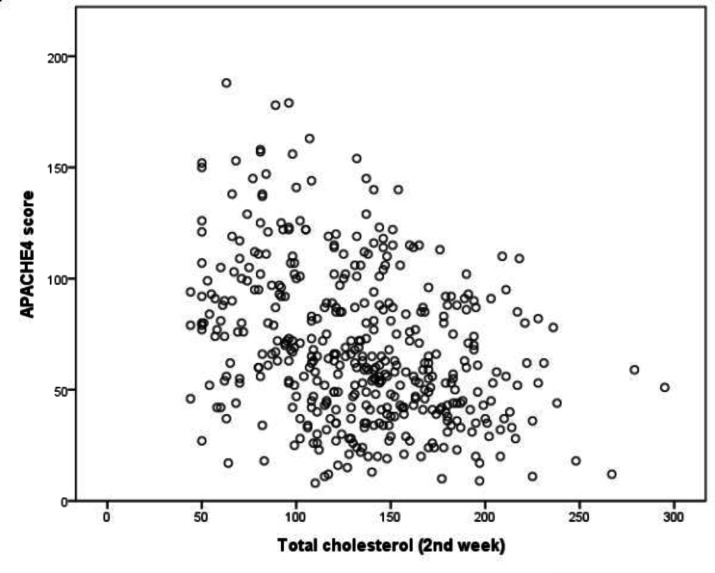
The second week TC and APACHE4 score

**Table 79 (abstract A249).** Risk factors for hospital mortality

	P-value	Hazard ratio	95% Confidential Interval
Second week TC	<0.001	0.98	0.97-0.99
C-reactive protein	0.02	1.05	1.01-1.09
ARDS	8.24	8.24	2.49-27.29
CRRT	0.34	0.53	0.15-1.94

### A250 Acute muscle wasting (myopenia) in ICU patients with complex pancreatitis, quantified from routinely acquired CT imaging

#### M. Kostalas^1,2^, J. Mckinlay^1^, G. Kooner^1^, G. Dudas^1,3^, A. Horton^4^, C. Kerr^4^, N. Karanjia^2,4^, B. Creagh-Brown^1,2^

##### ^1^Royal Surrey County Hospital, ICU and SPACeR research group, Guildford, United Kingdom; ^2^University of Surrey, Guildford, United Kingdom; ^3^University of Semmelweis, Budapest, Hungary; ^4^Royal Surrey County Hospital, Guildford, United Kingdom

###### **Correspondence:** J. Mckinlay – Royal Surrey County Hospital, ICU and SPACeR research group, Guildford, United Kingdom

**Introduction**

Acute pancreatitis accompanied by organ dysfunction is termed severe acute pancreatitis (SAP) and if local complications (such as infected pancreatic pseudocysts) develop it may be described as complex SAP. As these patients often require care by specialist hepatopancreaticobiliary (HPB) surgeons and interventional radiologists, they may be transferred to specialist HPB centres.

ICU-acquired weakness affects up to 40 % of patients on the ICU and is greatest in those with multiple organ systems requiring support. It is associated with prolonged need for mechanical ventilation and ICU stay, increased risk of death following hospital discharge, and long-term complications including impaired physical function [1,2]. One Method of quantifying muscle wasting is the measurement of the cross sectional area (CSA) of para-spinal muscles at the level of the third lumbar vertebra from CT imaging [3].

The ICU at RSCH receives patients transferred from other ICUs for specialist care relating to their complex SAP. For clinical reasons these patients often have repeated CT imaging of their abdomens.

**Objectives**

To characterise patients requiring critical care who have been transferred for specialist care of their complex pancreatitis between 2008 and 2014; and to measure their L3 para-spinal muscle CSA (L3MCSA) from each CT scan to ascertain their rate of muscle wasting.

**Method**

Patients were identified from our ICU patient database (WardWatcher) and additional clinical details acquired from electronic databases. CT Images were exported as DICOM files from our PACS system. The cross-sectional area of the paraspinal muscles were measured using ImageJ software in duplicate by two independent users and the average values were used. Analysis used Excel (MS) and GraphPad (PRISM).

**Results**

45 patients met inclusion criteria and of these, 21 patients had ≥ 2 CT scans in ICU, enabling serial estimation of L3 paraspinal muscle CSA (L3MCSA). The average age was 53 (16.6) years. The median APACHE II score was 17. Patients underwent a median of 2 interventional/surgical procedures. The mean ICU LOS was 48 (26.9) days and ICU mortality was 19.1 %.

95 % of patients had a decrease in their L3MCSA during their ICU stay. See Table 80.

The median % L3MCSA change / day was -0.56 %. Higher pancreatitis severity score (Glasgow score at admission to our ICU) was associated with greater loss of muscle (p = 0.03). Increased systemic inflammation (% of time with CRP > 200 mg/l) was associated with increased rate of loss of muscle (r = -0.45 and p = 0.03). L3MCSA was not demonstrated to relate to duration of ventilation or ICU mortality.

**Conclusions**

This cohort of ICU patients with complex SAP showed profound muscle wasting, greatest in those with persistent severe inflammation. Identification of preventative strategies is a research priority.

**References**

1) NEJM 2014;370:1626

2) JAMA 2013;310(15):1591

3) Appl. Physiol. Nutr. Metab. 2008;33:997-1006

**Table 80 (abstract A250).** Acute muscle wasting

	ICU admission	ICU discharge
L3MCSA/cm2 Median (IQR)	82.4 (65.9 - 90)	69.0 (62.6 - 75.3) p<0.001
% change Mean (95% CI)		-12.4% (-7.1 to -17.6)

### A251 The effect of the parenteral nutrition solution on the oxidant state of the critically ill patients

#### N.D. Altintas^1^, S. Izdes^2^, O. Keremoglu^2^, A. Alkan^3^, S. Neselioglu^4^, O. Erel^4^

##### ^1^Ankara University Faculty of Medicine, Department of Internal Medicine, Intensive Care, Ankara, Turkey; ^2^Yıldırım Beyazıt University, Medical Faculty, Department of Anesthesiology and Reanimation, Ankara, Turkey; ^3^Yıldırım Beyazıt University, Department of Biostatistics and Medical Informatics, Ankara, Turkey; ^4^Yıldırım Beyazıt University, Medical Faculty, Department of Biochemistry, Ankara, Turkey

###### **Correspondence:** N.D. Altintas – Ankara University Faculty of Medicine, Department of Internal Medicine, Intensive Care, Ankara, Turkey

**Introduction**

Oxidative stress is known to adversely affect a variety of cellular functions, promote inflammation and take part in development of multi-organ dysfunction. Oxidant status can be evaluated by thiol/disulphide homeostasis. Previous studies have indicated that certain lipid formulations may contribute to decrease the oxidative stress. Potentiation of PON1 activity is one of the suggested mechanisms [1].

**Objectives**

Our aim was to evaluate basal paraoxonase1 activity(PON1), salt stimulated paraoxonase(stPON1), arylesterase(ARE), plasma native thiol, total thiol and disulphide levels in critically ill patients on parenteral nutrition(PN) containing different lipid formulations.

**Method**

Critically ill patients for whom PN was planned, and who did not have a contraindication to PN were included in the study. Patients already on PN or who did not give consent were excluded. Three different PN solutions were used: a soya oil containing solution [long chain fatty acids(LCT)], an olive oil containing solution, and a solution containing middle and long chain fatty acids(MCT/LCT). Nutritional support was planned to provide 25-30 kcal/kg/day. Age, sex, admission diagnosis, smoking status, APACHEII and SOFA scores, invasive procedures performed, FiO_2_ levels, administered drugs, transfusion were recorded for 3 days(0, 24 and 48th hour). Lipid profiles(HDL, LDL, total cholesterol, triglyceride), liver enzyme levels(AST, ALT, GGT, ALP, total/direct bilirubin), CRP, prealbumin values were recorded. As well, nosocomial infections that developed during admission, ICU and hospital length of stays and outcomes were recorded. Serum samples were collected before start of PN, at 24th and 48th hours of PN for PON-1, stPON1, ARE and total thiol levels and stored at -20 °C. Biochemical studies were conducted after collection of all samples. PON1, stPON1 and ARE were measured spectrophotometrically. Plasma native thiol, total thiol and disulphide levels were measured as previously defined.

**Results**

A total of 36 patients, 12 in each group, were included in the study. Age, sex, day of admission to hospital, APACHEII scores, smoking status were similar across the groups. Lipid profiles, liver enzyme levels, CRP levels were also similar. Although initial (prenutrition) plasma native thiol, total thiol and disulfite levels were different across groups; on follow-up no difference was observed between the groups on the basis of PON1, stPON1, ARE, plasma thiol, total thiol and disulphide levels.

**Conclusions**

Based on the results of this study, different PN solutions with different lipid compositions do not seem to alter the oxidant status as evaluated by the thiol/disulphide homeostasis. Although olive oil is expected to promote PON1 activity, this could not be demonstrated in this study. Prospective, randomized, dose dependent studies may be planned for further evaluation.

**References**

1. Lou-Bonafonte JM et al. *Nutrients.* 2015;7(6):4068.

### A252 Autophagy flux in critically illness

#### N. Tardif, T. Gustafsson, O. Rooyackers

##### Karolinska University Hospital / Karolinska Institutet, Stockholm, Sweden

###### **Correspondence:** N. Tardif – Karolinska University Hospital/Karolinska Institutet, Stockholm, Sweden

**Introduction**

Autophagy is a survival process involved in the removal of protein aggregates and organelles that cannot be processed by the proteasome. Recently an impairment of this system has been hypothesized in the development of organ failure of the ICU patients.

**Objectives**

Autophagy is a highly dynamic process that cannot be studied in human *in vivo*. The aim of this study was to develop an experimental model to study the autophagy flux in ICU patients and to investigate which step of the autophagic pathway is impaired. We have developed an in vitro screening Method to measure autophagic flux in human primary myotubes incubated with serum from ICU patients.

**Method**

Human primary myotubes were incubated with serum from consecutive ICU patients taken in the first 24 h of admission (n = 95) and healthy volunteers (n = 10) with a similar age range (40 - 60 years old). Myotubes were cultured in 96-well plates, at 7 days of differentiation they were incubated with 10 % of human serum for 24 hours in the presence or absence of chloroquine (50 μM, 6 hr), an inhibitor of autophagy. p62 expression, a marker of autophagic vacuole accumulation, was measured by in-cell western, with an Odyssey scanner. The autophagic flux was calculated as followed: autophagic flux = p62 expression (+CQ) - p62 expression (-CQ). The results were normalized against cells number and expressed as a % of the autophagic flux in control condition. The screening on all serum samples have been repeated in 5 independent experiments. Results were analyzed by ANOVA with a Fisher post-hoc test.

## Results

We observe a larger variation in the expression of p62 induced by the patients' serum compared to the variation induce by the serum from healthy volunteers. p62 expression in myotubes incubated with the serum from 66 patients (ICU group) was similar to the healthy group (131 ± 4 vs. 122 ± 9 % of control; ICU vs. healthy). Interestingly, the serum from 29 ICU patients (ICU+ and ICU- groups) induced an expression of p62 superior of 2 SD in comparison with the values observed in myotubes incubated with the serum from healthy volunteers. Within this 29 samples, 14 were inducing an increase in the autophagic flux (p < 0.05, ICU+ vs. Healthy) and 15 were inducing a block of the autophagic flux (p < 0.05, ICU- vs. Healthy). Interestingly, the length of hospital stay was significantly greater in the ICU- (8.7 days, 95 % CI [6.0, 11.4]) compared to the ICU (4.0 days, 95 % CI [2.8, 5.3]) and the ICU+ (4.3 days, 95 % CI [1.7, 7.0]).

**Conclusion**

Serum from ICU patients was able to activate or block the flux of autophagy in human primary myotubes. Interestingly, even if the serum was collected during the first 24 h of admission we observed differences in the length of stay between our groups. The group inducing a block in autophagy corresponded to the long stayers patients.

### A253 Feeding tube monitoring - a quality improvement project

#### K.N. MacEachern, M. Traille, I. Bromberg, S.E. Lapinsky

##### Mount Sinai Hospital, Toronto, Canada

###### **Correspondence:** K.N. MacEachern – Mount Sinai Hospital, Toronto, Canada

**Introduction**

Nasal or oro-gastric nutrition tube placement is verified radiologically. Daily placement verification is typically by air insufflation into the tube and auscultation, which has been shown to be unreliable. Tubes may move with patient movement, patient pulling, or coughing; necessitating ongoing placement verification. Other verification Method are testing gastric aspirate pH or pepsin, tube CO_2_ output, visualization of gastric aspirate, and record of tube insertion length. Our intensive care unit (ICU) at Mount Sinai Hospital initiated a quality improvement (QI) project to change from auscultation to the gastric pH, with record of tube insertion length and visualization of gastric aspirate.

**Objective**

To adopt an evidenced based practice change and complete plan, do, see, act (PDSA) cycles, then make further changes based on our results.

**Method**

Gastric aspirate samples were sent to the laboratory for testing by pH meter. Nursing education was done to introduce the test and procedure. A pH ≤ 5.5 was considered confirmation of gastric placement. Data collected included aspirate pH, use of acid inhibiting drugs, calories from EN and ordered and delivered, time EN held before pH testing, tube insertion marking length, and aspirate appearance. Ongoing education and changes to nursing and nutrition practice were made as results were evaluated as part of our PDSA cycle. Statistical analysis included mean pH, stratified by acid inhibitor, mean calorie deficit from holding EN for the test, and the 90^th^ percentile laboratory turnaround time.

**Results**

**Fig. 103 (abstract A253).**
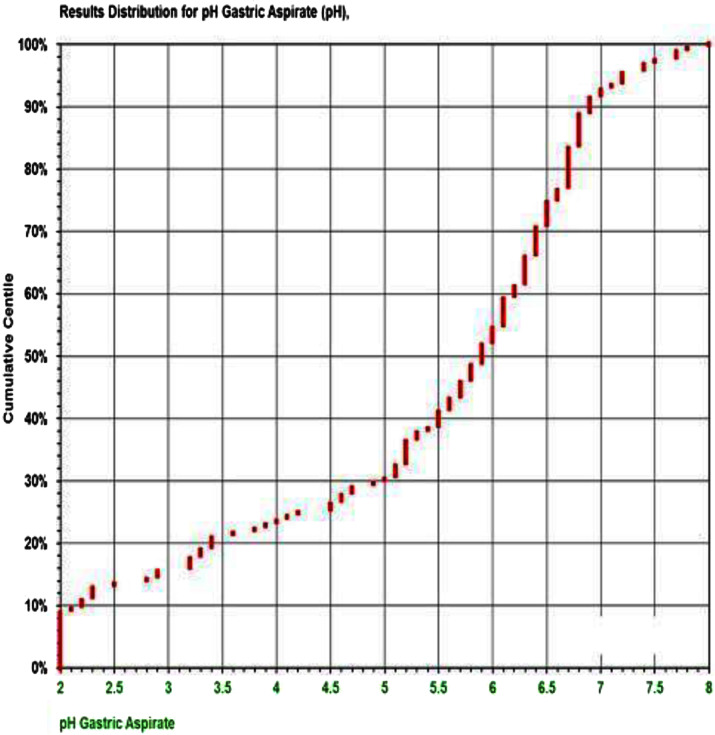
Results distribution for gastric aspirate (pH)

Ninety pH tests were done and 21 tests missed, for 12 patients. The overall mean pH was 5.2 and median pH 5.7. Stratification by drug therapy results can be found in Table 81. A cumulative percentile chart of pH with acid inhibitor Method is reported in Fig. 103. All patients received EN. The mean calorie deficit observed was 1513 calories, for all reasons, not the gastric pH test alone. EN was held for 1 h before the test. The lab reported results usually in 30 minutes. Length of the feeding tube to insertion point was recorded for 1 patient.

**Conclusion**

Nurses were accepting of this process as evidenced by the few missed tests. Tests weren't done due to confusion over tube types requiring the test, inability to aspirate fluid from the tube, and forgetting. This created an opportunity for staff education to improve our process. Our data suggest that gastric aspirate pH ≤ 5.5 alone is not a reliable marker of gastric feeding tube placement when patients receive acid inhibiting drugs. There is opportunity to develop a confirmation bundle including a description of the aspirate, recording of length of the tube at insertion, and use of acid suppressing therapy. The possibility of underfeeding is an opportunity to implement a volume based EN protocol to allow the nurse to adjust the hourly volume of EN to compensate for time EN is held. This ICU QI project implemented a best practice following a PDSA cycle.

**Table 81 (abstract A253).** Gastric pH with acid suppressing therapy, mean (n)

Medication	No acid suppressing therapy	Histamine 2 blocking agent	Proton pump inhibitor
pH	3 (12)	5.2 (41)	6.2 (37)

### A254 Testing gastric pH using CE marked colour change indicator strips to ascertain gastric placment of oro/naso enteral feeding tubes: is it safe and reliable?

#### M.J. Moore

##### St Georges NHS Foundation Trust, Critical Care Directorate, London, United Kingdom

**Introduction**

Following insertion of a naso/oro gastric enteral tube and periodically afterwards in the United Kingdom it is essential to ensure that the distal portion of the tube is in the lower protion of the GI tract (the stomach). This is achieved principally through measuring the pH of gastric aspirate (1st line test) =/- x-ray (2nd line test) (NPSA, 2011). This small pilot study illuminates the accuracy (and potential for errors) with using pH indicator strips to confirm gastric placment.

**Objectives**

To establish the accuracy of Nurses and Doctors interpretation of a range of known pH solutions using CE marked pH indicator strips with visual interpretation of pH results

**Method**

50 nursing and medical staff were asked to identify the pH of clear/colourless solutions with pH values ranging between 1 and 7. They were blinded to the pH value of the soultion until after they had completed the test. The pH indicator strips used had a possible range of 0-6.0.

**Results**

All staff agreed to participate in this small study with the assurance of anonymity. Staff were observed to perform the test correctly if they

- waited 10-60 seconds prior to reading the strip;
- did not wipe the strip after immersion in the test solution. 100 % acheived accurate testing procedures.

The majority of staff (approximately 80 %) correctly interpreted the pH of solutions between 1 and 4 but demonstrated slightly less accuracy with pH of 5.8 (75 % accurate), erring on the lower value on the pH indicator strip of 5.5

When challenged with a pH solution which was know to be 7, only 1 out of ten staff they could not identify the pH value since the colour change was out of range of the pH indicator strip. The remaing staff (9-90 %) incorrectly interpreted the solution as having a pH of 6).

**Conclusions**

Although this was a small study, there is clearly a danger in relying on visual interpretation of colour change of CE marked pH indicator strips to confirm correct placement of naso/orogastric feeding tubes. Future/further studies intend to compare alternative Method of pH quantification, incuding electronic pH testing devices.

**References**

1. National Patient Safety Agency (2011). Reducing the harm caused by misplaced nasogastric feeding tubes in adults, children and infants. NPSA: London

## TISSUE PERFUSION, DIAGNOSTIC AND THERAPEUTIC ASPECTS

### A255 Transpulmonary thermodilution versus transthoracic echocardiography for cardiac output measurements in severe sepsis patients

#### Z. Tang, C. Cai, L. Tong

##### The First Affiliated Hospital of Sun Yat-Sen University, Guangzhou, China

###### **Correspondence:** Z. Tang – The First Affiliated Hospital of Sun Yat-Sen University, Guangzhou, China

**Introduction**

Measurements of cardiac output (CO) are frequentlydone in surgical and critically ill patients as part of optimizationstrategies Transthoracic echocardiography (TTE) Method of CO measurement is more popular in critically ill patients.

**Objectives**

Whether TTE Method of CO measurement is accurate as transpulmonary thermodilution (TPTD).

**Method**

We compared near-simultaneously performed CO measurements in severe sepsis patients using TPTD with the PiCCO (Pulse index Continuous Cardiac Output) system or TTE. Outcomes were compared using t-tests, linear regression

**Results**

Forty severe sepsis patients were studied. An analysis of 120 data pairs revealed that PiCCO yielded similar CO measurements to TTE (p  > 0.05). PiCCO- derived CO measurements highly related to and TTE- derived CO measurements (R  =  0.75, p  <  0.05).

**Conclusions**

TTE can used for objective cardiovascular monitoring and to guide goal-directed fluid resuscitation in severe sepsis patients instead of PiCCO, and is noninvasive and convenient.

**References**

1. Morgaz J, Granados Mdel M, Muñoz-Rascón P, et al. Comparison of thermodilution, lithium dilution, and pulse contour analysis for the measurement of cardiac output in 3 different hemodynamic states in dogs.J Vet Emerg Crit Care (San Antonio), 2014,24(5):562-70.

2. Itami T, Endo Y, Hanazono K, Ishizuka T, et al.Comparison of cardiac output measurements using transpulmonary thermodilution and conventional thermodilution techniques in anaesthetized dogs with fluid overload. Vet Anaesth Analg, 2015,doi: 10.1111/vaa.12331.

### A256 Prognostic value of maximum dose of norepinephrine in septic shock

#### J.-L. García-Garmendia, F. Villarrasa-Clemente, F. Maroto-Monserrat, O. Rufo-Tejeiro, V. Jorge-Amigo, M. Sánchez-Santamaría, C. Colón-Pallarés, A. Barrero-Almodóvar, S. Gallego-Lara

##### Hospital San Juan de Dios del Aljarafe, Intensive Care Unit, Bormujos, Sevilla, Spain

###### **Correspondence:** J.-L. García-Garmendia – Hospital San Juan de Dios del Aljarafe, Intensive Care Unit, Bormujos, Sevilla, Spain

**Introduction**

Norepinephrine (NE) is the most common vasoconstrictor drug used in the treatment of septic shock. High doses of NE are associated to bad prognosis, but the limits of refractory septic shock have not been well established.

**Objective**

To analyze the prognostic value of the maximum dose of norepinephrine on the mortality of patients admitted to ICU with septic shock.

**Method**

A two-years retrospective study in a single ICU on patients admitted with septic shock treated with NE. The NE maximum dose was analyzed, the time to reach this dose, the days of treatment, the origin and focus of sepsis, the use of other amines, the APACHE II and SOFA score and ICU and hospital mortality. Prognostic value of maximum dose of NE was analyzed using multivariate logistic regression and a ROC curve analysis was carried out to assess its discriminating ability.

**Results**

We analyzed 128 consecutive patients, with an average age of 64 years (SD 15,6), a mean APACHE II 20 (SD 8) and a mean SOFA score of 8,3 (SD 3,4). The average value of the maximum dose of NE was 0,94 μg/Kg/min (SD 0,84) and the median time to achieve it was 1 day (range 0-22). ICU mortality was 30 % and hospital mortality was 34 %. In the multivariate analysis, the maximum dose of NE was independently associated with ICU mortality (14,6 95 % CI: 6,1-34, 8; p < 0,001) and hospital mortality (9,2 95 % CI: 4,0-19,3; p < 0,001). ROC curve of the maximum dose of NE showed a high discriminative ability for ICU mortality with a r = 0, 93 (95 % CI 0,88-0,97; p < 0,001) and for hospital mortality with r = 0,87 (95 % CI 0,81-0,94, p < 0,001), with a cut-off point of maximum sensitivity (0,82) and specificity (0,83) on 1 μg/Kg/min. In-hospital mortality of patients with dose maximum of NE > 1 μg/Kg/min was 72 % versus 10,3 % in patients with lower doses and it was 100 % in the patients with septic shock with doses greater than 2 μg/Kg/min (20 cases).

## Conclusions

The maximum dose of norepinephrine had an independent prognostic value in septic shock. Doses higher than 1 μg/Kg/min are associated with a clear worse prognosis, so it could be considered the limit of refractory septic shock.

**References**

1. Kastrup M, Braun J, Kaffarnik M, von Dossow-Hanfstingl V, Ahlborn R, Wernecke KD, et al. Catecholamine dosing and survival in adult intensive care unit patients. World J Surg. 2013 Apr;37(4):766-73

2. Brown SM, Lanspa MJ, Jones JP, Kuttler KG, Li Y, Carlson R, et al. Survival after shock requiring high-dose vasopressor therapy. Chest 2013 Mar; 143(3):664-71.

3. Martin C, Medam S, Antonini F, Alingrin J, Haddam M, Hammad E, et al. Norepinephrine: not too much, not too long. Shock 2015 Oct; 44(4):305-9

### A257 The effects of hydroxyethyl starch 130/0.42 vs. Ringer’s acetate on cytokine levels in patients with severe sepsis

#### C.T. Anthon^1^, R.B. Müller^1^, N. Haase^1^, K. Møller^2^, P.B. Hjortrup^1^, J. Wetterslev^3^, A. Perner^1^

##### ^1^Copenhagen University Hospital - Rigshospitalet, Department of Intensive Care, Copenhagen, Denmark; ^2^Copenhagen University Hospital - Rigshospitalet, Center of Inflammation and Metabolism, Copenhagen, Denmark; ^3^Copenhagen University Hospital - Rigshospitalet, Centre for Clinical Intervention Research - Copenhagen Trial Unit, Copenhagen, Denmark

###### **Correspondence:** C.T. Anthon – Copenhagen University Hospital - Rigshospitalet, Department of Intensive Care, Copenhagen, Denmark

**Introduction**

The 6S trial showed increased 90-day mortality with hydroxyethyl starch (HES) 130/0.42 vs. Ringer's acetate in patients with severe sepsis [1], but the pathophysiology driving this has not been fully elucidated.

**Objectives**

To compare changes in cytokine plasma concentrations in the days after randomisation into the 6S trial.

**Method**

In a subgroup of 226 patients from the 6S trial we analysed differences between the HES- and Ringer's groups in delta plasma concentrations of TNF-α, IL-6 and IL-10 from baseline to day 2 after randomisation using multiple linear regression analysis. Additionally, associations between the changes in cytokines and 90-day mortality were investigated using multiple logistic regression analysis. We imputed values missing due to discharge or errors using multiple imputation.

**Results**

Baseline characteristics were similar in the HES- and the Ringer's groups. By day 2, 13 (11 %) patients in the HES group had died vs. 11 (10 %) in the Ringer's group (P = 0.91). Plasma concentrations of TNF-α, IL-6 and IL-10 decreased from baseline to day 2 in the HES- and the Ringer's groups, but mean delta cytokine concentrations did not differ between the groups (Table 82). Also, no associations were observed between changes in the cytokine plasma concentrations and 90-day mortality (TNF-α: odds ratio for 1-unit increase, 1.000 (95 % Confidence Interval, 0.998 - 1.003), P = 0.87; IL-6: 1.001 (0.999 - 1.002), P = 0.32; IL-10: 1.000 (0.999 - 1.001), P = 0.89).

**Conclusions**

Resuscitation with HES 130/0.42 vs Ringer's acetate did not affect TNF-α, IL-6 and IL-10 plasma concentrations and changes in these cytokines were not associated with 90-day mortality in patients with severe sepsis. Consequently, our results suggest that the increased mortality seen with HES in the 6S trial was not mediated by early changes in systemic inflammation.

**References**

1. Perner A, Haase N, Guttormsen AB, et al. Hydroxyethyl starch 130/0.42 versus Ringer´s acetate in severe sepsis. N Engl J Med. 2012;367:124-34.

**Grant acknowledgment**

The 6S trial was funded by Rigshospitalet and the Danish Research Councils and supported by the ACTA foundation and B. Braun Medical.

**Table 82 (abstract A257).** Mean differences in delta cytokine concentrations from baseline to day 2 in patients with severe sepsis resuscitated with either HES or Ringer's acetate

Cytokine	Estimate, HES vs. Ringer	95% CI	P-value
TNF-alfa (pg/mL)	-23.9	-58.7 to 10.8	0.18
IL-6 (pg/mL)	17.3	-69.0 to 103.6	0.69
IL-10 (pg/mL)	-66.7	-164.9 to 31.5	0.18

The imputed dataset from all the 202 day 2 survivors were analysed and adjusted for SAPS II and the stratification variables: university hospital, hematologic malignancy and shock at randomization

### A258 Does B-type natriuretic peptide estimate the re-filling phase in sepsis patients?

#### M. Nakanishi^1^, A. Kuriyama^2^, T. Fukuoka^2^

##### ^1^Kurashiki Central Hospital, Department of Emergency Medicine, Kurashiki, Japan; ^2^Kurashiki Central Hospital, Department of General Medicine, Kurashiki, Japan

###### **Correspondence:** M. Nakanishi – Kurashiki Central Hospital, Department of Emergency Medicine, Kurashiki, Japan

**Introduction**

Fluid overload is associated with poor outcomes in septic patients. B-type natriuretic peptide (BNP) concentrations are useful tools to guide the therapy of patients with heart failure. However, BNP concentrations in the patients after septic shock resucitation are poorly studied. If BNP concentrations can predict the day of the peak fluid balance, BNP guided therapy enables us to restrict the fluid volume and start the diuresis appropriately.

**Objectives**

We investigated daily BNP concentrations and fluid balance in septic patients. We hypothesized that the day of the peak BNP concentration predict the day of maximum fluid balance. The patient age, sources of infection are included in the analysis as confounders.

**Method**

Our inclusion criteria was as follows; 1) septic patients of any sources of infection (urinary tract infection, pneumonia, soft tissue infections, upper and lower GI tract perforation, and bacteremia), 2) those admitted to our ICU, and 3) the daily BNP concentrations were measured during the ICU stay. We excluded patients who had been already on hemodialysis. We first described the time lag between the day of the peak BNP concentration and that of maximum daily fluid balance. We conducted multivariate regression analysis on this time lag with patient age, sources of infection, and maximum daily fluid balance as covariates.

**Results**

We included 48 patients, aged 75 in average. The peak BNP concentration followed the maximum daily fluid balance by 0.57 day (SD, 2.36). In multivariate regression analysis, the delay of maximum BNP concentration after the maximum daily fluid balance was associated with older age (β 0.08, p = 0.03), but was not associated with net daily fluid balance or sites of infections.

**Conclusions**

Our study suggested that the timing of maximum BNP concentrations and the maximum daily fluid balance might parallel in septic patients. However, the peak BNP concentrations tended to follow the maximum daily fluid balance as the patients get older. Thus, BNP cannot be used to estimate the refilling phase.

**Grant acknowledgement**

None.

### A259 Impact of gelatins on perfusion of microcirculatory blood flow in patient with septic shock

#### M.A. Abd el Halim^1^, M.H. Elsaid hafez^1^, A.M. Moktar^1^, A. Eladawy^1^, H.M. Elazizy^1^

##### ^1^Cairo University/Kasr Alainy Medical School, Anethesia, Cairo, Egypt

###### **Correspondence:** M.A. Abd el Halim – Cairo University/Kasr Alainy Medical School, Anethesia, Cairo, Egypt

**Introduction**

Microcirculatory alterations in sepsis plays a vital role in development of multi organ system failure, and this is clear from the observation that despite correction of systemic hemodynamic parameters still usually the course of multi organ system failure continue[1]. Effective fluid resuscitation is a corner stone in the effective management of patients with septic shock with the goal to improve tissue perfusion at the micro circulatory level[2].

**Objectives**

The aim of this work is to compare and evaluate the influence of gelatin and saline on sublingual microcirculation in patients with severe sepsis and septic shock. Another objective is to assess its effects on the incidence of renal dysfunction and acid base disturbance.

**Method**

Prospective, randomized, controlled study was conducted on 42 patients with severe sepsis and septic shock, Patients meeting inclusion criteria was randomly assigned to receive either gelatin 4 % (21 pts) or saline 0.9 % (21 pts). Patients received 500 ml of either solution every 30 minutes till reaching the goals of initial resuscitation, using a Sidestream Dark Field device mean flow index was determined before and 6 hours after resuscitation. Arterial blood pressure, heart rate, and vasopressor therapy was recorded every 30 min during the first 6 h. Arterial blood samples were collected at three specific times: on randomization before fluid administration, 6 and 24 hours after initial resuscitation. Urea, creatinine, urine output and SOFA score was measured daily for the first 5 days after resuscitation.

**Results**

Among forty two patients enrolled in the study, demographic data and patients´ characteristics were comparable among both groups. After 6 hours from resuscitation, no difference arose in the Sublingual microcirculation parameters between both groups, mean flow index after resuscitation in gelatin group (1.65 ± 0.65) which was not superior to saline group (1.74 ± 0.65). No difference was noticed in acute kidney injury nor acid base disturbance, but there was significantly greater net cumulative fluid balance in saline group 2726 (1429) ml compared to gelatin group 930 (2450) ml (P = 0.006) which indicates more fluid need for the first 24 hours in the saline group.

**Conclusions**

The main finding in this prospective randomized study was that the use of gelatin in resuscitation of patients with septic shock does not confer any advantage over saline in recruitment of sublingual microcirculation.

**References**

1. Shapiro NI, Arnold R, Sherwin R,et al. The association of nearinfrared spectroscopy-derived tissue oxygenation measurements with sepsis syndromes, organ dysfunction and mortality in emergency department patients with sepsis. Crit Care 2011; 15:R223.

2. Zanotti-Cavazzoni SL, Guglielmi M, Parrillo JE, et al. Fluid resuscitation influences cardiovascular performance and mortality in a murine model of sepsis. Intensive Care Med 2009;35:748-754.

### A260 Feasibility of using antecubital peripheral venous oxygen saturation for the management of severe sepsis and septic shock

#### K. Abdel Hakim, A. Chaari, M. Elbahr, M. Ismail, T. Mahmoud, V. Kauts, K. Bousselmi, E. Khalil, W. Casey

##### King Hamad University Hospital, Intensive Care Department, Muharraq, Bahrain

###### **Correspondence:** K. Abdel Hakim – King Hamad University Hospital, Intensive Care Department, Muharraq, Bahrain

**Introduction**

Central venous oxygen saturation (ScvO2) is an indirect indicator of the adequacy of oxygen delivery and cardiac output. A reduction in the level of ScvO2 below normal levels indicates an increase in oxygen extraction by the tissues in response to a decrease in the arterial oxygen content or cardiac output[1]. The ScvO2 has been widely utilized for hemodynamic optimization in severe sepsis/septic shock[2]. A time lag usually occurs between admission of a septic patient to the emergency department and the insertion of a central venous catheter (CVC). Thus, the need for a surrogate to the ScvO2 which is easily and rapidly obtainable.

To date peripheral venous oxygen saturation (SpvO2), which involves an antecubital blood sample has not been validated to replace the ScvO2 for the purpose of hemodynamic optimization in severe sepsis/septic shock.

**Objectives**

The objective of this study was to assess the feasibility of replacing the ScvO2 with the antecubital SpvO2 for the management of severe sepsis/septic shock.

**Method**

Thirty five successive patients with severe sepsis/septic shock in whom a CVC was inserted were the subject of this study. Simultaneous central venous and antecubital peripheral venous samples were withdrawn and tested for oxygen saturations. We compared 35 pairs of simultaneous ScvO2 and SpvO2 samples.

**Results**

Mean age was 65.3 ± 16 years. Sex-ratio (M/F) was 1.7. Eleven patients (31.4 %) had severe sepsis and 24 patients (68.6 %) had septic shock.

Spearman's correlation coefficient between ScvO2 and SpvO2 was 0.497 (p = 0.002). Subgroup analysis of patients with severe sepsis and patients with septic shock showed a Spearman's rho coefficient of 0.727 (p = 0.011) and 0.422 (p = 0.065) respectively.

**Conclusions**

SpvO2 showed good correlation with ScvO2 in patients with severe sepsis. Therefore, it may be a useful tool for early implication of hemodynamic optimization in patients with severe sepsis.

**References**

1. Walley KR. Use of central venous oxygen saturation to guide therapy. American journal of respiratory and critical care medicine. 2011;184(5):514-20.

2. Dellinger RP, Levy MM, Rhodes A, Annane D, Gerlach H, Opal SM, et al. Surviving sepsis campaign: international guidelines for management of severe sepsis and septic shock: 2012. Crit Care Med. 2013;41(2):580-637.

### A261 The prognostic value of red blood cell distribution width (RDW) in patients with sepsis and its relations with shock index and haemodynamic parameters

#### S.H. Zaky, A. Rizk, M.O. Elghonemi, R. Ahmed

##### Cairo University, Critical Care, Cairo, Egypt

###### **Correspondence:** M.O. Elghonemi – Cairo University, Critical Care, Cairo, Egypt

**Introduction**

For several decades, RDW has been typically used in combination with the MCV to differentiate the cause of underlying anaemia in clinical practice. Recently, high RDW has been associated with increased mortality in patients with severe sepsis and Septic Shock [2]. The pathophysiologic mechanisms underlying the association between RDW and mortality are unclear; however, it is possible that its relationship with inflammation and oxidative states plays role in this[3]. RDW has been linked with inflammation in critically ill patients and with oxidative stress in animal models.

**Objectives**

To assess the prognostic value of red cell distribution in patients with sepsis and its relation with hemodynamic parameters assessed non-invasively.

**Method**

Prospective observational study that included thirty patients admitted to the MICU sepsis. RDW, shock Index & Hemodynamic Parameters by Cardiac Bio impedance measurement were assessed during hospital stay in all Patients[4,5].

**Results**

RDW was higher in Non-survivors than survivors (22.46 % ±3.2 Vs 15.97 % ± 1.32 ), (P value < 0.001) with cutoff value of mortality 18.5 % with sensitivity 96 %, specificity 100 % and AUC 0.99 . Higher Shock Index in Non-survivors than survivors (1.26 ± 0.39 Vs 0.78 ± 0.13), (P value < 0.001) with cutoff value of Mortality 0.87 with sensitivity 85 %, specificity 89 % and AUC 0.86. There was a Significant correlation between RDW & lactate and between RDW & shock index with P < 0.001 in Both respectively . -Cardiac output & cardiac Index were higher in Non-survivors than survivors (8.59 ± 2.4 vs 6.6 ± 1.25) & (4.76 ± 1.47 vs 3.46 ± 0.72) , (P value = 0.031 & 0.003) respectively but without significant correlation with RDW.

**Conclusions**

High RDW is associated with higher mortality in sepsis patients and is higher in patients who developed respiratory failure during ICU stay. RDW showed significant correlations with shock index and lactate levels.

**References**

1. *"Red Cell Distribution Width"*. Family Practice Notebook. Retrieved 18 October 2013

2. *Jo, You Hwan, et al.*"Red cell distribution width is a prognostic factor in severe sepsis and septic shock." The American journal of emergency medicine 31.3 (2013): 545-548.

*3. Ghaffari S (2008) Oxidative stress in the regulation of normal and neoplastic hematopoiesis. Antioxid Redox Signal*

4. *Allgöwer M, Buri C.Schockindex. Deutsche* Medizinische Wodenschrift. 1967

5. *Lorente, Leonardo, et al.* "Red Blood Cell Distribution Width during the First Week Is Associated with Severity and Mortality in Septic Patients." (2014): e10

### A262 Sublingual versus gut: comparative evaluation of microcirculatory dysfunction in the acute phase of sepsis

#### J.C.F. Vieira^1^, R.B. Souza^2^, A.M.A. Liberatore^1^, I.H.J. Koh^1^

##### ^1^Federal University of São Paulo, Surgery, São Paulo, Brazil; ^2^Federal University of São Paulo, Morphology and Genetics, São Paulo, Brazil

###### **Correspondence:** J.C.F. Vieira – Federal University of São Paulo, Surgery, São Paulo, Brazil

The installation of progressive microcirculatory dysfunction has been identified as a crucial factor in the development of MODS in sepsis, which is why the identification, prevention or recovery of microcirculatory damage has been a major focus of research. In clinical practice, the sublingual region has been the site of choice for evaluation of dysfunction in sepsis, mainly for ease of access. However, there are doubts as to its validity in relation to microcirculation of other organs commonly committed in sepsis. This study aimed to compare the sublingual microcirculation with intestinal, during the initial phase of sepsis in order to assess whether the sites with the same embryological origin have similarities in microcirculatory dynamics.

**Method**

Wistar rats were subjected to sepsis (iv. E. coli 2x10^9^ CFU/mL, DL80 in 26 hours, n = 10) and microcirculation of sublingual regions and jejunum were captured by Sidestream Darkfield images (SDF). The total vessel density (TVD) was analyzed by software AVA-3.0 at 0, 1, 2, 3, 4, 5 and 6 consecutive hours post sepsis. The sham group was injected with saline only. (n = 5). In all periods of the study the animals were kept under general anesthesia with mechanical ventilation, receiving hydration (7 ml saline/kg/hr, iv).

**Result**

**Fig. 104 (abstract A262).**
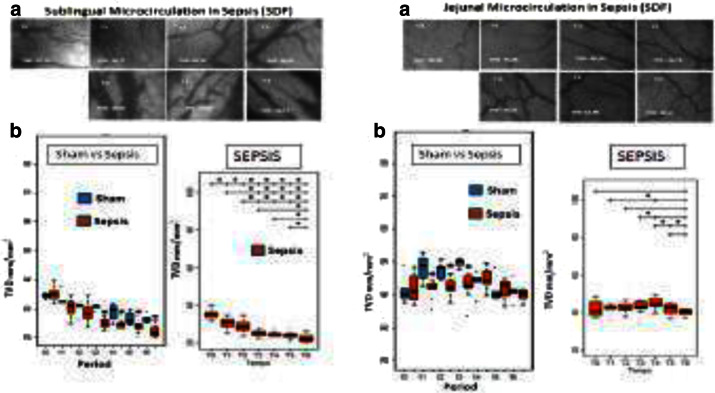
Sublingual and Jejunal Microcirculation in Sepsis

In sublingual, there was a significant reduction in vascular density of animals with sepsis only after 3 hours of sepsis compared to the sham group. (Fig. 104). This suggests that reducing the density in sepsis is only noticeable during periods of increased severity of sepsis. The comparison between the periods of sepsis showed that only the first two hours had a higher density compared to other periods of sepsis. These results have shown that in sepsis, the density of the microcirculation decreases with the severity of sepsis, however, AVA-3.0 Method was able to show differences only between extreme stages (almost normal with the stages of extreme severity), indicating a low sensitivity of the Method to differentiate small changes in density. In the gut, no changes were detected between groups and between periods, showing that the jejunum density is not variable in sepsis. These data showed that the dynamic microcirculatory is organ-specific and independent of embryological origin. In short, the evaluation of microcirculatory dysfunction is site-dependent and appears to require assessment Method also organ-specific to the kinetic measurement of microcirculatory dysfunction in sepsis.

**Grant acknowledgment**

FAPESP 2011/20401-4.

### A263 Dobutamine improves intestinal microvascular blood flow heterogeneity and oxygen extraction in a hypodynamic septic shock model

#### G.A. Ospina-Tascón^1^, A.F. Garcia Marin^1^, G.J. Echeverry^1^, W.F. Bermudez^1^, H.J. Madriñan-Navia^1^, J.D. Valencia^1^, E. Quiñonez^1^, A. Marulanda^1^, C.A. Arango-Dávila^1^, A. Bruhn^2^, G. Hernandez^2^, D. De Backer^3^

##### ^1^Fundación Valle del Lili - Universidad ICESI, Department of Intensive Care Medicine, Cali, Colombia; ^2^Pontificia Universidad Católica de Chile, Facultad de Medicina, Departamento de Medicina Intensiva, Santiago, Chile; ^3^CHIREC Hospitals, Université Libre de Bruxelles (ULB), Department of Intensive Care Medicine, Brussels, Belgium

###### **Correspondence:** G.A. Ospina-Tascón – Fundación Valle del Lili - Universidad ICESI, Department of Intensive Care Medicine, Cali, Colombia

**Introduction**

Derangements on microvascular blood flow distribution have been implied on impairment of oxygen extraction by peripheral tissues.

**Objectives**

To evaluate the effects of dobutamine on the heterogeneity of microvascular blood flow at intestinal and sublingual territories and its relationship with mesenteric and systemic oxygen extraction in a severe hypodynamic septic shock model.

**Method**

Fifteen landrace pigs were anesthetized and mechanically ventilated. Catheters were inserted into carotid artery, pulmonary artery and portal vein for blood sampling, and for invasive pressures and cardiac output monitoring. A gas-tonometer was placed into jejunum for mucosal-CO_2_ determination while a jejunostomy was prepared to visualize gut-mucosal microcirculation. At each measurement time-point, arterial, mesenteric-venous and mixed-venous blood samples were obtained to blood gases analysis. Images of intestinal microcirculation were acquired using the Side-stream dark Field technique. These video-sequences were stored under a random number for ulterior blinded analysis. After baseline measurements (BL), fecal peritonitis was induced. When hypotension was not responsive to fluid resuscitation, norepinephrine was started and shock was declared (TS). Then, pigs were randomized to a fixed-dose of dobutamine at 5 μg/kg/min (n = 6) or placebo (n = 6). New measurements were performed 2 (T2H) and 6 hours (T6H) after. Three sham animals subjected to identical monitoring served as time-matched controls

**Results**

We observed a significant decrease in the proportion of intestinal-villi perfused (%villi-PPV) at TS in both dobutamine and placebo groups. Cardiac output and systemic DO_2_ evolved similarly in both experimental groups. After starting resuscitation, %villi-PPV significantly increased in dobutamine group (Friedman repeated-measures analysis of variance, p < 0.05 for time and time/group interactions). Variations of %villi-PPV between baseline measurements and each measurement time-point (BL-to-TS; BL-to-T2H; BL-to-T6H) exhibited a good agreement with variations in mesenteric VO_2_ and oxygen extraction ratio (R^2^ = 0.57 and 0.71 respectively, p < 0.001). Variations of small-vessels perfused (PPV) at sublingual mucosa (SL-PPV) tracked %villi-PPV throughout the experiment and were well correlated with mesenteric VO_2_ (R^2^ = 0.70)

**Conclusions**

Dobutamine improved heterogeneity of gut microvascular blood flow during severe hypodynamic septic shock. Decreasing heterogeneity of flow was associated with an improvement in regional oxygen extraction, suggesting the crucial role of microvascular blood flow distribution on oxygen consumption in sepsis

**Grant acknowledgment**

Tecnoquimicas S.A. (Colombia) - Centro Investigaciones Clinicas, Fundacion Valle del Lili (CO) (CIC 001). Universidad ICESI (CO) (IP-FO-01)

**Fig. 105:**
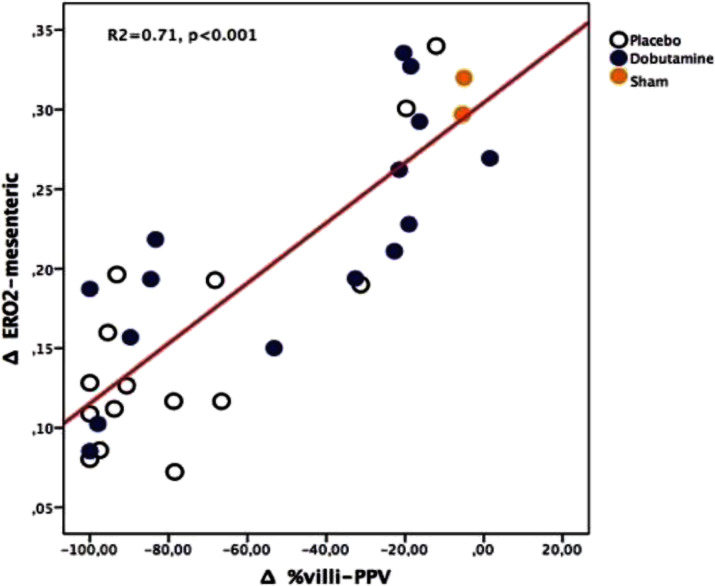
(abstract A263).

.

### A264 Detection of tissue hypoperfusion in an experimental model of septic shock

#### D. Orbegozo Cortes, F. Su, J.-L. Vincent, J. Creteur

##### Erasme University Hospital, Université Libre de Bruxelles, Intensive Care, Brussels, Belgium

###### **Correspondence:** D. Orbegozo Cortes – Erasme University Hospital, Université Libre de Bruxelles, Intensive Care, Brussels, Belgium

**Introduction**

Septic shock is characterized by altered perfusion, potentially leading to tissue hypoxia. The central-peripheral difference of some metabolites (lactate, oxygen and CO_2_) and their ratios may be good markers of peripheral hypoperfusion.

**Objectives**

To characterize the evolution of some peripheral indexes of perfusion and metabolism in a model of sepsis due to peritonitis.

**Method**

We studied 18 anesthetized, mechanical ventilated and invasively monitored sheep, in which autologous feces were injected into the abdomen. Fluid administration was titrated to maintain initial pulmonary artery occlusion pressure. Vasopressors were not used. Arterial, mixed venous (v), and right femoral venous (fv) blood samples were collected at baseline and every 4 hours to perform blood gas analysis and lactate (LACT) measurements. A microdialysis catheter was placed in the posterior leg muscle to measure the lactate/pyruvate (LP) ratio every hour. We calculated the **fCO**_**2**_ **gap** as PfvCO_2_-PaCO_2_, **fLACT gap** as fvLACT - art LACT, **fO**_**2**_ **content gap** as [(SaO_2_ × 1.34 × Hb) + (0.031 × PaO_2_)]-[(SfvO_2_ × 1.34 × Hb) + (0.031 × PfvO_2_)], **fO**_**2**_ **Sat gap** as SaO_2_-SfvO_2_, **fLACT/O**_**2**_ **index** as fLACTgap/fO_2_content gap, **fCO**_**2**_ **/O**_**2**_ **index** as fCO_2_gap/fO_2_content gap and the **modified (m) fCO**_**2**_ **/O**_**2**_ **index** as fCO_2_gap/fO_2_Sat gap. These variables were investigated at baseline (T1), before hypotension (MAP < 60 mmHg) (T3), before death (T5) and at midpoints between these times (T2 and T4). Results are presented as median (p25-75) values. Statistical analyses were performed using SPSS 23.0.

**Results**

All animals died during the 30-hour observation period. Results at each time point are presented in Tables 83 and 84, and all data before and after the development of hypotension are presented in Table 85. († = only 12 animals; * = p < 0.05 vs T1).

**Conclusions**

All peripheral indexes were influenced by the decreased blood flow, but the fCO_2_ gap and the fLACT gap were altered earlier; these indexes could potentially be useful to detect tissue hypoperfusion during sepsis.

**Table 83 (abstract A264).** Global hemodynamic variables

	T1	T2	T3	T4	T5
MAP (mmHg)	106(97-110)	86(79-93)*	64(61-83)*	51(45-54)*	44(37-49)*
Cardiac index (L/min/m^2^)	4.9(4.0-5.2)	4.0(3.6-5.1)*	4.3(3.7-4.8)*	3.2(2.9-3.9)*	2.7(2.3-3.3)*
Art LACT (mmol/L)	1.2(0.9-1.5)	1.2(0.8-1.3)	1.3(1.1-1.7)	2.2(1.7-3.0)*	5.7(4.8-10.7)*
SvO2 (%)	74(70-79)	72(68-80)	70(61-79)*	61(57-69)*	44(39-54)*
CO2 gap (mmHg)	6(4-7)	6(4-7)	6(5-8)	9(6-10)*	14(12-23)*

**Table 84 (abstract A264).** Peripheral perfusion variables

	T1	T2	T3	T4	T5
Femoral blood flow (mL/min) †	50(30-100)	32(29-40) *	14(10-31)*	6(4-10)*	5(3-12)*
LP ratio	35(25-49)	29(15-47)	35(21-200)	77(42-357)*	84(28-658)*
fLACT gap (mmol/L)	0.18(0.03-0.23)	0.34(0.15-0.53)*	0.65(0.56-1.04)*	1.68(1.06-2.08)*	1.80(1.20-2.31)*
fLACT/O2 index	0.05(0.01-0.10)	0.09(0.06-0.12)	0.14(0.09-0.17)*	0.22(0.13-0.30)*	0.25(0.20-0.27)*
fCO2 gap (mmHg)	4(4-8)	8(6-9)*	11(8-13)*	18(15-19)*	23(18-33)*
fCO2/O2 index	1.89(1.68-2.14)	1.64(1.53-1.90)	1.70(1.58-2.15)	2.23(1.86-3.00)	3.15(2.97-3.70)*
m fCO2/O2 index	28(26-33)	27(25-34)	29(25-40)	41(32-51)	59(52-66)*

**Table 85 (abstract A264).** Median values before and after hypotension

	MAP>60mmHg	MAP<60mmHg	p
MAP (mmHg)	84 (72-93)	48 (40-54)	<0.01
Art LACT (mmol/L)	1.1 (0.8-1.4)	3.6 (2.0-6.4)	<0.01
Femoral blood flow (mL/min)	32 (18-48)	6 (4-11)	<0.01
L/P ratio	31 (19-47)	66 (30-408)	<0.01
fLACT gap (mmol/L)	0.33 (0.15-0.60)	1.75 (1.12-2.08)	<0.01
fLACT/O2 index	0.09 (0.04-0.13)	0.22 (0.16-0.29)	<0.01
fCO2 gap (mmHg)	8 (6-10)	17 (14-23)	<0.01
fCO2/O2 index	1.68 (1.48-1.92)	2.93 (2.00-3.53)	<0.01
m fCO2/O2 index	27 (25-33)	51 (37-61)	<0.01

### A265 Ventriculoarterial coupling monitoring in the treatment of septic shock patients: preliminary data

#### L. Tullo, L. Mirabella, P. Di Molfetta, G. Cinnella, M. Dambrosio

##### University of Foggia, Anaesthesia and Intensive Care, Foggia, Italy

###### **Correspondence:** L. Mirabella – University of Foggia, Anaesthesia and Intensive Care, Foggia, Italy

**Introduction**

Shock is best defined as a life-threatening, generalized form of acute circulatory failure associated with inadequate oxygen utilization by the cell. Importantly, peripheral vasodilation (relative hypovolemia and low systemic vascular resistance) coexist with cardiac dysfunction (left ventricular (LV) diastolic and systolic dysfunction secondary to primary myocardial injury or right ventricular dysfunction due to pulmonary hypertension) resulting in ventriculoarterial decoupling. Ventriculoarterial coupling is defined by the ratio of arterial elastance (Ea) to left ventricular end-systolic elastance (Ees).

**Objectives**

In this study, we analyzed the variability of ventriculoarterial coupling in ICU patients with septic shock and the effect of therapeutic interventions such as vasopressor and inotropic agent to improve perfusion.

**Method**

In this prospective study, we measured routine hemodynamics using indwelling arterial catheters and transthoracic echocardiograms in septic patients upon ICU admission. Hemodynamic variables included cardiac index (CI), heart rate (HR), mean arterial pressure (MAP). Ees was measured by echocardiography using a single-beat (EesSB) Method Ea was calculated as 0.9 systolic arterial pressure/stroke volume, and then the Ea/EesSB ratio was calculated (normal value < 1.36).The measure were repeated every day for a maximum of seven days.

**Results**

We analyzed a series of measured parameters in six patients. At the time of diagnosis of septic shock patients presented in a hyperdynamic state (median CI = 2.96 ± 1.10 L/min/m2 and HR = 84 ± 24 beats/min, hypotensive (MAP 56.8 ± 17,6 ) and higher Ea/EesSB ratio (1.41 ± 0.08). After starting treatment with norepinephrine and levosimendan and as it improved the state of septic patients , there was a parallel normalization of haemodynamic parameters and especially of ventriculoarterial coupling, the Ea/EesSB ratio was respectively 1.21 ± 0.1, 1.01 ± 0.10, 0.93 ± 0.11 on day 2, 3 and 4.

**Conclusions**

In septic shock patients, there is a higher percentage of ventriculoarterial decoupling.. Because ventriculoarterial decoupling is an index of cardiovascular inefficiency and a determinant of cardiac energetics, we speculate that such “uncoupled” patients may benefit from therapies aimed at normalizing the Ea/Ees ratio

**References**

1. Guarracino F, Ferro B, Morelli A et al Ventriculoarterial decoupling in human septic shock. Critical Care 2014, 18:R80

### A266 Fluid therapy in septic shock. Beyond the resuscitation fluids; are hidden fluids administered?

#### C. Villavicencio Lujan, J. Leache irigoyen, M. Cartanya ferré, R. Carbonell García

##### Joan XXIII University Hospital, Tarragona, Spain

###### **Correspondence:** C. Villavicencio Lujan – Joan XXIII University Hospital, Tarragona, Spain

**Objectives**

To evaluate the characteristics of fluid therapy during the first week of septic shock and its possible impact in 28-day mortality.

**Method**

Prospective, observational study, conducted in a polyvalent ICU (intensive care unit). 35 patients with diagnosis of septic shock were included over a period of 5 months. Demographic data, comorbidities, APACHE II, SOFA, clinical and hemodynamics parameters, as well as fluid therapy characteristics were analysed during the first week of hospitalization. The impact in 28-day mortality was evaluated. We defined “hidden volume” as the volume delivered beyond the resuscitation fluid therapy.

**Results**

During the first 72 hours of treatment in ICU, the patients received an average total volume of 12.8 ± 4.0 litters. The amount of resuscitation fluids decreased progressively from 48 % (mean 1313.1 ± 664.7 ml) to 4 % (mean 520 ± 382.4 ml) within the first 72 hours. The opposite was found regarding maintenance and drug-associated volume, with an increase from 27 % (mean 383.5 ± 189.2 ml) to 42 % (mean 1493.6 ± 649.7 ml) and from 30 % (mean 421.7 ± 297.3 ml) to 37 % (mean 1304.1 ± 906.8 ml) respectively, within the first 72 hours. A tendency towards hemodynamic stabilization was observed within the first 72 hours, with a mean arterial pressure of 70 ± 13 mmHg to 86 ± 14 mmHg, diuresis of 1188 ± 1119 ml to 1782 ± 1404 ml and lactate of 3.68 ± 2.38 mmol/l to 2.19 ± 1.58 mmol/L. It was found that 28-day mortality was significantly lower when patients received more resuscitation fluids during the first 6 hours of hospitalisation (1087.6 ± 1102.8 ml vs. 492.4 ± 416 ml, p = 0.024). In contrast, those patients with a higher 7-day cumulative hydric balance (321 ± 7702.4 ml vs 2346.6 ± 2444.2 ml, p = 0,018) showed a higher mortality. Clinical and hemodynamic characteristics in admission to the ICU were compared between 28-day survivors and non-survivors without significant differences, except for more left ventricular systolic dysfunction within the non-survivors (33,3 % vs. 18,7 %; p > 0,05).

**Conclusions**

During the first week of ICU admission, the excess of fluids seems to be caused by hidden fluids (maintenance and drug-related fluids). Furthermore, these appear to be related with a higher mortality, as does an insufficient resuscitation within the first 6 hours of ICU admission.

**References**

1. Surviving sepsis campaign: international guidelines for management of severe sepsis and septic shock, 2012. Intensive Care Med 2013; 39: 165-228

2. Cordemans C, et al.: Fluid management in critically ill patients: the role of extravascular lung water, abdominal hypertension, capillary leak and fluid balance. Ann Intensive Care 2012; 2

3. Mikkelsen ME, et al. Serum lactate is associated with mortality in severe sepsis independent of organ failure and shock. Crit. Care Med 2009. 37:1670-1677.

### A267 Real time heart rate variability is highly predictive of mortality in pediatric septic patients

#### A. Mukhtar^1^, M. Ahmed^1^, M. El Ayashi^2^, A. Hasanin^1^, E. Ayman^1^, M. Salem^1^, A. Eladawy^1^, S. Fathy^1^, H. Nassar^1^, A. Zaghlol^1^

##### ^1^Cairo University, Cairo, Egypt, ^2^Cairo University Medical School, Cairo, Egypt

###### **Correspondence:** M. Ahmed – Cairo University, Cairo, Egypt

**Introduction**

Heart rate variability (HRV), has shown promise in predicting pediatric patients at high risk for sepsis and death [1]. However, its use in clinical practice has been precluded by the absence of real-time data.

**Objective**

This study was conducted to evaluate the utility of electrical cardiometry for real-time determination of HRV and using these data in predicting the outcome in pediatric patients presented with septic shock.

**Method**

Prospective enrolment of pediatric patients who met the criteria of septic shock. Electric cardiometry (ICON) was used to measure time-domain HRV continuously in real time for 2 hours after admission to intensive care unit. Electric impedance cardiography was used to determine cardiac output. Hemodynamic parameters which discriminate survivors from non-survivors were evaluated.

**Results**

We enrolled 15 pediatric patients with septic shock of whom 5 (33 %) patients were discharged and 10 (66 %) died. Heart rate was similar between survivor and non-survivor. The median (interquartile range [IQR]) of HRV of enrolled patients was significantly lower among the non- survivors (4.3 {3.8-7.3}) than survivors (19.5 {13.5-34.7}) (*P* = 0.005).The cut-off of the HRV value for predicting mortality was ≤ 12.5. This cut-off value had a sensitivity of 100 % and a specificity of 80 %; the area under the curve was 0.96 (95 % confidence interval 0.7-1.0, P < 0.0001.

**Conclusion**

We concluded that decreased HRV is good predictor of mortality in pediatric patient with septic shock. Real-time HRV may be a useful adjunct to standard vital signs monitoring in this population.

**References**

1. Griffin MP, Lake DE, Bissonette EA, et al. Heart rate characteristics: novel physiomarkers to predict neonatal infection and death. Pediatrics 2005;116:1070-4.

### A268 Effectiveness of fluid thoracic content measurement by bioimpedance guiding intravascular volume optimization in patients with septic shock

#### M.F. Aguilar Arzapalo

##### SSA UADY, Mérida, Mexico

**Introduction**

Appropriate resuscitation and intravascular volume optimization are treatment cornerstone in septic shock. Optimization requires invasive monitoring and static measurements, due to it, is necessary a non invasive, fast and dynamic Method Thoracic fluid content (TFC) by thoracic bioimpedance surge as a monitoring system that fulfills the requirements: non invasive, easy placement and dynamic

**Objectives**

Evaluate the effectiveness of fluid thoracic content measurement by bioimpedance guiding intravascular volume optimization in patients with septic shock

**Method**

Sixty patients were enrolled, divided in two groups with 30 subjects each, admitted at ICU with septic shock diagnosis. F Group was formed by patients in whom TFC was measured by thoracic bioimpedance, in order to guide intravascular volume optimization; C Group patients were optimized following “Surviving Sepsis Campaign Guidelines”, recording mortality at ICU discharge and at 28 days, mechanical ventilation length and ICU length of stay

**Results**

Based on TFC F Group obtained a faster and higher Mean Arterial Pressure (MAP) increasing up to 42.5 % at 6 hours, compared to C Group where MAP arise only 19.5 % at 6 hours (p:< 0.05). Due to lactate clearance F Group, obtained a total clearance of 54.4 % and 66.6 % at 6 and 12 hours, on C Group clearance just came up to 25.3 % and 35.8 % at 6 and 12 hours (p: 0.001). A higher mortality at ICU discharge was reach on C Group with 33.4 % vs 26.7 % (p: 0.001) from F Group, 28 day mortality was 36.5 % and 27.8 % (p:0.003) on C and F Group respectively.

**Note:** This abstract has been previously published and is available at [1]. It is included here as a complete record of the abstracts from the conference.

**References**

1. Aguilar Arzapalo M, Barradas L, Lopez V, Cetina M (2016) Effectiveness of fluid thoracic content measurement by bioimpedance guiding intravascular volume optimization in patients with septic shock. Critical Care 20(Suppl 2):94.

## Conclusions

Measurement of thoracic fluid content by thoracic bioimpedance is effective while optimizing intravascular volume in septic shock

**References**

1. Facchini C, Malfatto G, et al. Lung ultrasound and transthoracic impedance for noninvasive evaluation of pulmonary congestion in heart failure. JCM 2015 Ene;(10);2459

**Grant acknowledgment**

Hospital O´Horán

**Fig. 106 (abstract A268).**
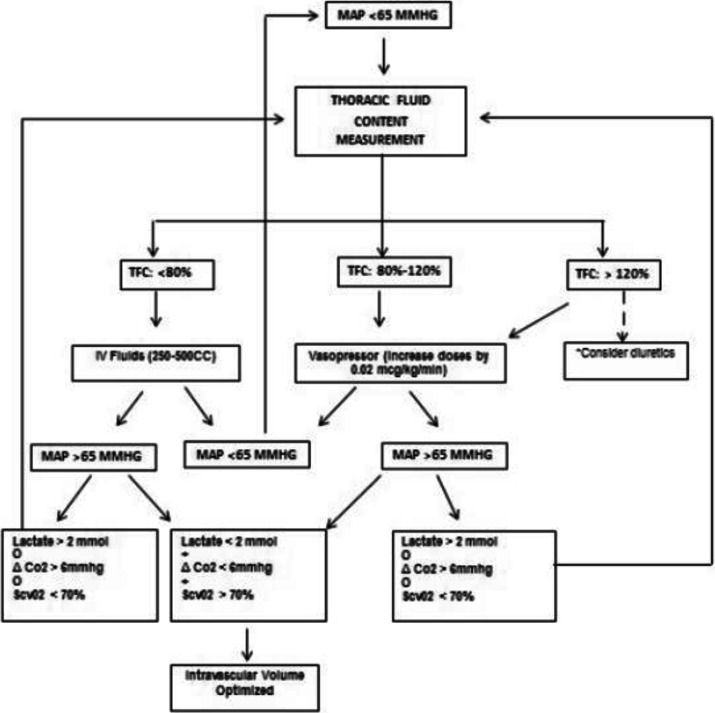
Intravascular Optimization Algorithim

## STAFF & PATIENT CHALLENGES IN ICU

### A269 Normal 0 21 association between post traumatic stress symptoms and sense of coherence in intensive care unit patients

#### Å. Valsø^1^, K. Sunde^1^, T. Rustøen^1^, I. Schou-Bredal^2^, L. Skogstad^3^, K. Tøien^1^

##### ^1^Oslo University Hospital, Division on Emergencies and Critical Care, Oslo, Norway; ^2^Oslo University Hospital, Divison of Cancer, Oslo, Norway; ^3^Oslo University Hospital, Division of Medicine, Oslo, Norway

###### **Correspondence:** Å. Valsø – Oslo University Hospital, Division on Emergencies and Critical Care, Oslo, Norway

**Introduction**

Posttraumatic stress symptoms (PTSS) following intensive care treatment are frequent. In accident victims strong sense of coherence (SOC) has previously been associated with less PTSS. To our knowledge no study has investigated the association between PTSS and SOC in a mixed intensive care unit (ICU) population.

**Objectives**

To examine the prevalence of PTSS after ICU stay, and the relationship between PTSS, SOC and demographic and clinical characteristics in ICU patients.

**Method**

In a cross sectional study patients ^3^18 years treated in two ICUs at Oslo University Hospital Ullevål for ^3^ 24 hours were consecutively included during one year (2014-2015). Demographic and clinical data were collected. PTS symptoms and SOC were measured with Post Traumatic Stress Scale 10 Intensive care screen (PTSS10-I) and Sense of Coherence Scale 12 (SOC-12) at the ward after ICU discharge. PTSS-I0-I scores ^3^35 was used as cut off value indicating clinical significant PTSS. Descriptive and multivariate logistic regressions analysis was performed with IBM SPSS statistics 21.

**Results**

We included 133 patients with mean age 54 ( ±17 SD) and 56 % being male, of whom 37 % were trauma patients and 54 % receiving ventilator treatment. Prevalence rate of defined PTSS was 25 %. Demographic and clinical variables were investigated for association with PTSS and variables with a p value < 0,1 were included in the multivariate logistic regression analysis. Adjusting for gender and age, only SOC (OR 0.91, [95%CI: 0.87-0.95]) and ventilator treatment (OR 4.98, [95%CI: 1.67-14.83]) were significantly associated with PTSS symptoms.

**Conclusions**

In our study population low SOC and ventilator treatment was associated with clinical significant PTSS in patients after discharge from the ICU.

### A270 Effort reward imbalance and burnout in an ICU nursing team: comparison between registered nurses and nurse aides

#### C. Padilla^1^, Y. Palmeiro^2^

##### ^1^Hospital Clinico UC - CHRISTUS, Unidad de Paciente Critico, Santiago, Chile; ^2^Universidad de los Andes, Santiago, Chile

###### **Correspondence:** C. Padilla – Hospital Clinico UC - CHRISTUS, Unidad de Paciente Critico, Santiago, Chile

**Introduction**

Intensive Care Units (ICU) are characterized not only for caring the sickest patients but also due to its high levels of anxiety and stress in health workers, which takes its maximum expression in the burnout syndrome. The cause of this burden is frequently related to specific ICU work factors such as physical and emotional workload. There are several theoretical approaches to these problems, for example the imbalance effort - reward (ERI) model, which has been poorly tested in the ICUs. In chilean ICUs, the multidisciplinary team is mostly composed by registered nurses and nurse aides whom must work hand in hand to ensure the quality and safety of care. The aim of this study was to investigate the prevalence of effort-reward imbalance and burnout among ICU registered nurses and nurse aides in a teaching hospital.

**Methodology**

Comparative, cross-sectional study. A convenience sample of ICU registered nurses (RN) and nurse aides (NA) from a teaching hospital in Santiago, Chile was obtained. Three questionnaires were used for data collection:

i.) A sociodemographic questionnaire,
ii.) Spanish short version of Effort-Reward Imbalance questionnaire by Siegrist, an ERI > 1 indicated an imbalance between efforts and rewards , and
iii.) Maslach Burnout Inventory, including their 3 subscales: emotional exhaustion (EE), personal accomplishment (PA) and depersonalization (DP). A p-value < 0.1 was considered.

**Results**

The response rate was 87.2 % (n = 82). A 56.1 % of the sample was NA and 43.9 % was RN. An 89.1 % of NA reported an ERI > 1, while registered nurses reached 75 % (p < 0.1). Compared to RN, NA had higher ERI (1.58 v/s 1.23, p < 0.05) and lower rewards (13.9 v/s 17.5, p < 0.01). Only one case of burnout was observed among NA, but no cases were observed among RN. A higher proportion of RN was observed with medium levels of DP (47.2 % v/s 32.6 %, p < 0.1) and PA (44.4 % v/s 30.4 %, p < 0.1) compared to NA, however NA had a higher proportion of low levels of PA compared to RN (56.5 % v/s 30.6 % p < 0.05).

**Conclusions**

Most of the ICU nursing team has an effort-reward imbalance, which is higher in nurse aides due to a lower perception of rewards. Burnout seems not to be a problem, but low degree of personal accomplishment suggests that is necessary to perform early interventions based on the recognition and opportunities for personal development, specifically on nurse aides, this interventions could help to maintain low levels of burnout and improve effort reward imbalance.

**Grant acknowledgment**

None of the authors of this work received any grant or financial aid.

### A271 Nurses´ perceptions of patient safety climate in intensive care units

#### W. Egbaria^1^, R. Kigli^2^

##### ^1^Tal Hashomer Hospital, General ICU, Umm el Fahem, Israel; ^2^Tel Aviv University, Ramat Gan, Israel

###### **Correspondence:** W. Egbaria – Tal Hashomer Hospital, General ICU, Umm el Fahem, Israel

**Introduction**

Climate safety is a design factor guiding the judgment and behavior of health care professionals, and highlights the issue of patient safety at the top of their priorities. At safety climate, employees are directed to act in accordance with company-wide commitment to safety, so that each state maintain personal safety norms and those of colleagues. This formed a consensus on the relative importance of safe behavior in situations that present conflicting requirements in safety competitors such as meeting deadlines.

**Objectives**

Checking the safety climate concept among intensive care nurses while making a comparison between ICU nurses and CCU nurses. And check whether the theoretical model fails to predict a safe culture among intensive care nurses?

**Method**

The study population consisted of 81 nurses who work in intensive care units in hospitals at tel aviv district. The subjects were asked to complete a structured questionnaire containing 69 items. Which examined the perception of nurses´ patient safety climate in workplace, communication between employees, frequency of reported incidents, organizational and departmental policies and procedures and practices. Data were collected in April-May 2015.

**Results**

This study increased moderately positive correlation exists between the perception of workers ICU patient safety, and the total score of the atmosphere of safety (r = 0.623, p < 0.000). There is a positive medium correlation between the perception of icu workers of corporate policies and departmental and between process and Methodology (r = 0.568, p < 0.01), while in the ICU General (r = 0.626, p < 0.01), compared to intensive care Cardiology (r = 0.508, P < 0.01).

**Conclusions**

The findings support the theory of ZOHAR (2000, 2003) about the safety climate that is meaningful for intensive care nurses. Which refers to a common perception among members in all aspects of the organizational environment, that dictates the behavior at work and the extent to which certain aspects are rewarded and supported in the organization. There are differences between the general intensive care and cardiac intensive care in evaluating patient safety and an atmosphere of safety, particularly in assessing and evaluating the work area corporate policy.

**References**

1. Colla, J.B., Bracken, A.C., Kinney, L.M. & Weeks, W.B. (2005). Measuring patient safety climate: a review of survey. *Quality safe health care*, 14, 364-366.

2. Dov, Z. & Gil, L. (2005). A multilevel model of safety climate: cross level relationships between organization and group level climates. *Journal of applied psychology*. 90 (4), 616-628.

**Grant acknowledgment**

The current research haven’t published yet.

### A272 Continuous endotracheal cuff-pressure (P_cuff_) control in the prevention of ventilator-associated respiratory infections (VARI): a systematic review and meta-analysis

#### B. Maertens^1^, K. Blot^1^, S. Blot^2^

##### ^1^Ghent University, Faculty of Medicine and Health Sciences, Ghent, Belgium; ^2^Ghent University, Dept. of Internal Medicine, Ghent, Belgium

###### **Correspondence:** B. Maertens – Ghent University, Faculty of Medicine and Health Sciences, Ghent, Belgium

**Introduction**

Ventilator-associated respiratory infections (VARI) are an important source of morbidity in ICU patients. Continuous endotracheal P_cuff_-control is a way to minimize micro-aspiration of subglottic secretions, which is considered to be a major pathogenic mechanism of VARI.

**Objectives**

To perform a systematic review and meta-analysis to assess the efficacy of continuous P_cuff_-control in the prevention of VARI.

**Method**

MEDLINE, EMBASE, CENTRAL/CCTR, Clinicaltrials.gov and ICTRP were systematically searched. Eligible trials were randomized controlled clinical trials (RCTs) and quasi-RCTs comparing continuous P_cuff_-control with manual, intermittent P_cuff_-control in intubated patients. All studies reporting VARI incidence were included. VARI includes ventilator-associated pneumonia and ventilator-associated tracheobronchitis. Inclusion of trials was irrespective of publication status, date of publication or language.

**Results**

Two RCTs *[1,2]* and one quasi-RCT *[3]* meeting the inclusion criteria were identified (one using a pneumatic device *[1]* and two using an electronic device for continuous P_cuff_-control *[2,3]*). All studies were single-center; none of the trials was blinded for the intervention. 465 patients were allocated to the intervention arm and 455 to the control arm. 50 VARI episodes occurred in the intervention group and 91 in the control group. The pooled Odds Ratio for the incidence of VARI was 0.45 (random effects model, 95 % confidence interval, 0.30-0.69; z = 3.68 p < 0.001).

**Conclusions**

This meta-analysis shows that continuous P_cuff_-control appears to reduce the incidence of VARI. However, the number of included studies is small, and there is an inherent risk of bias due to the unblinded designs.

**References**

1. Valencia M, et al. Crit Care Med 2007

2. Nseir S, et al. Am J Respir Crit Care Med 2011

3. Lorente L, et al. Am J Infect Control 2014

**Grant acknowledgment**

We received no grants for this study.

**Fig. 107 (abstract A272).**
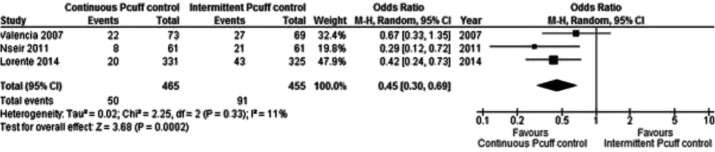
Meta-analysis Forest plot; incidence of VARI

### A273 Acute kidney injury in adult patients undergoing heart transplantation: incidence and outcomes according KDIGO criteria

#### E. Santana-Santos^1^, E.R. dos Santos^2^, R.E.D.L. Ferretti-Rebustini^3^, R.D.C.C.D.O. dos Santos^1^, R.G.S. Verardino^1^, L.A. Bortolotto^4^

##### ^1^Heart Institute (InCor) do Hospital das Clínicas da Faculdades de Medicina da Universidade de São Paulo, Nursing Coordination, São Paulo, Brazil; ^2^Instituto Israelita de Ensino e Pesquisa Albert Einstein, School of Nursing, São Paulo, Brazil; ^3^Nursing School of University of São Paulo, São Paulo, Brazil; ^4^Heart Institute (InCor) do Hospital das Clínicas da Faculdades de Medicina da Universidade de São Paulo, Hypertension Department, São Paulo, Brazil

###### **Correspondence:** E. Santana-Santos – Heart Institute (InCor) do Hospital das Clínicas da Faculdades de Medicina da Universidade de São Paulo, Nursing Coordination, São Paulo, Brazil

**Introduction** acute kidney injury (AKI) is a commom complication after heart transplantation and it is associated with high morbidity and mortality.

**Objective**

To evaluate the incidence of acute kidney injury (AKI) and outcomes in adult patients undergoing heart transplantation according KDIGO criteria.

**Method**

We performed a retrospective cohort study in a tertiary hospital specialized in cardiac surgery and reference in Latin America. Patients older than 18 years old who had undergone heart transplantation between January 2011 and January 2015 were included. Patients with congenital heart disease were excluded.

**Results**

Patients were divided into two groups (those who did not develop AKI and those who did develop AKI). The incidence of acute kidney injury was 86 %. We did not observe diference between the groups in relation to males (70.0 % vs. 66.7 %, p = 0.834), age (43 ± 13 years vs. 46 ± 12 years, p = 0.493) and EuroSCORE (8.2 ± 1.6 vs. 7.4 ± 2.2, p = 0.289), respectively in no AKI and AKI group. When we compared clinical characteristics before the transplantation, we found significant differences in relation to in inotropic dosis (18.6 ± 5.1 mcg/kg/min vs. 14.2 ± 5.9 mcg/kg/min, p = 0.037) and the use of intra-aortic balloon pump (88.9 vs. 46.7, p = 0.012), respectively in no AKI and AKI group. The outcomes length of stay and mortality were greater in the postoperative period in the group of patients who did develop AKI when compared with the group who did not develop AKI.

**Conclusion**

The incidence of acute kidney injury in patients undergoing heart transplantation was 86 %. The mortality was higher with the presence of AKI.

**References**

1. Hobson CE, Yavas S, Segal MS, Schold JD, Tribble CG, Layon AJ, Bihorac A. Acute kidney injury is associated with increased long-term mortality after cardiothoracic surgery. Circulation 2009; 119: 2444-53.

2. Sampaio MC, Máximo CA, Montenegro CM, Mota DM, Fernandes TR, Bianco AC, Amodeo C, Cordeiro AC. Comparison of diagnostic criteria for acute kidney injury in cardiac surgery. Arq Bras Cardiol 2013; 101: 18-25.

3. Thakar CV, Liangos O, Yared JP, Nelson D, Piedmonte MR, Hariachar S, Paganini EP. ARF after open-heart surgery: Influence of gender and race. Am J Kidney Dis 2003; 41: 742-51.

### A274 High performance teams in ICU: engaging critical care nurses as key stakeholders

#### A.-M. Doyle, I. Naldrett, J. Tillman, S. Price

##### The Royal Brompton and Harefield NHS Foundation Trust, Critical Care, London, United Kingdom

###### **Correspondence:** A.-M. Doyle – The Royal Brompton and Harefield NHS Foundation Trust, Critical Care, London, United Kingdom

**Introduction**

Team leadership & staff engagement are key influences on performance, clinical outcomes & patient satisfaction[1,2]. There is increasing recognition of stress experienced by critical care nurses[3,4,5]. These factors, alongside a shortage of critical care nurses, indicate that recruitment, retention & professional support for nurses are key issues in the delivery of safe & effective healthcare.

**Objective**

The study aimed to positively engage nurses, as key stakeholders in critical care, to identify the gold standard framework for a high performance team, from a nursing perspective. The study uses qualitative Methodology, which is recognised to be under-utilised as a research tool in critical care[6].

**Method**

Critical care nurses (n = 102) participated in focus group discussions addressing four key themes: 1. Identification of the gold standard high performance team in critical care; 2. Identification of current team performance; 3. Identification of current team strengths; 4. Identification of key priorities for quality improvement.

**Results**

Results were analysed using inductive content analysis & revealed the outline of a gold standard high performance critical care team from a nursing perspective. Key factors included: high morale, positive team climate, effective/open communication, role clarity, good clinical outcomes, patient satisfaction, professional development opportunities, job satisfaction, strong leadership & supportive management. The results also indicated the nursing team's perception of current team strengths & key priority for change.

**Conclusion**

This is the first study outlining the gold standard high performance team in critical care from a nursing perspective using qualitative research Methodology. The work forms part of a wider quality improvement strategy engaging multi-disciplinary staff in critical care team performance work with the aim of improving the quality and safety of patient healthcare.

**References**

^*1*^ *Factors supporting high performance in health care organisations: a review of the literature.* La Trobe University: Health Management Group.

^2^ Reader, T. W., Flin, R., Mearns, K., & Cuthbertson, B. H. (2009). Developing a team performance framework for the intensive care unit. *Crit Care Med*, 37(5), 1787-1793. doi: 10.1097/CCM.0b013e31819f0451

^3^ Epp, K. (2012). Burnout in critical care nurses: a literature review. *Dynamics*, 23(4), 25-31.

^4^ Bakker, A. B., Le Blanc, P. M., & Schaufeli, W. B. (2005). Burnout contagion among intensive care nurses. *J Adv Nurs*, 51(3), 276-287. doi: 10.1111/j.1365-2648.2005.03494.

^5^ Spence Laschinger, H.K. and Leiter, M.P. (2006) The impact of nursing work environments on patient safety outcomes: the mediating role of burnout/engagement. *Journal of Nursing Administration,* 36(5): 259-267 Leggatt, S. and Dwyer, J. (2003)

^6^ Charlesworth, M., & Foëx, B. A. (2015). Qualitative research in critical care: Has its time finally come? *Journal of the Intensive Care Society*, 1751143715609955.

### A275 Measuring nursing workloads and activity in critical care: a review of evidence

#### S. Shrestha^1,2^, P. Pearson^3^, J. Greaves^3^, D. Goodall^3^, A. Berry^4^, A. Richardson^5^

##### ^1^Frimley Health NHS Foundation Trust, ICU, Frimley, United Kingdom; ^2^The Royal College of Nursing Critical Care and Inflight Nursing Forum, London, United Kingdom; ^3^Northumbria University, Newcastle, United Kingdom; ^4^UKCCNA, Manchester, United Kingdom; ^5^BACCN, Newcastle, United Kingdom

###### **Correspondence:** S. Shrestha – Frimley Health NHS Foundation Trust, ICU, Frimley, United Kingdom

**Introduction**

Nurse Staffing guidelines in Critical Care generally suggest a nurse/patient ratio of 1:2 for High Dependency Unit (HDU) patients and 1:1 for Intensive Care Units (ICU) (FICM, 2015). Current recommendations depend mainly on organ failure as the evidence available on which to judge adequate levels of staffing is relatively limited and based on the opinions of expert group.

**Objective**

The United Kingdom Critical Care Nursing Alliance (UKCCNA), an alliance of all the leading critical care nursing organisations in the United Kingdom, have developed a proposal, commissioned and directed a literature review of critical care nursing activity tools that currently exists and utilised around the world.

**Method**

A rapid review Methodology was employed by a team of critical care experts, an information specialist and two nursing academics. The scope of the search included research studies, guidelines and surveys within and outside United Kingdom in the last 20 years. The search encompassed subject specific electronic databases. The SPICE standardised framework was used (Booth 2004).

**Results**

117 abstracts were considered, 81 abstracts excluded and a total of 36 further considered. Studies were drawn from around the globe, with considerable work undertaken in UK (9 studies) and Brazil (10 studies). The empirical studies ranged from retrospective analyses, staff diaries and surveys, observational studies, prospective studies of the use of specific tools and staffing models and instrument development. The tools examined fell into two main groups: those related to the condition and needs of the patient, and those related to nursing activities and interventions.

**Conclusions**

Understanding nursing workload and activity is complex and there are numerous tools available to measure this. Nursing Activities Score (NAS) was the most extensively examined tool, with generally reliable results focusing on the whole of the ICU nurse's workload so most suitable for evaluating overall staffing levels. The risk based model provides a means of determining nurse allocation around risk as opposed to Workload or patient dependency. However, we concluded that there is a scope to develop a new tool using the elements from the tools examined in this review which then needs to be tested in clinical practice.

**References**

1. FICM (2015). Guidelines for the Provision of Intensive Care Services, Faculty of Intensive Care Medicine & Intensive Care Society.

2. Booth, A (2004) Formulating answerable questions. In Booth, A & Brice, A (Eds) Evidence Based Practice for Information Professionals: A handbook. (pp. 61-70) London: Facet Publishing.

### A276 Efforts towards building the capacity of the paediatric critical care nurse task force in East Africa

#### G.O. Odundo, P. Omengo, P. Obonyo, N.M. Chanzu

##### Gertrude's Children's Hospital, Nairobi, Kenya

###### **Correspondence:** G.O. Odundo – Gertrude's Children's Hospital, Nairobi, Kenya

**Introduction**

There is a direct link between Intensive Care Unit (ICU) staff numbers and clinical outcomes; an increase in the ICU staff capacity translates to improved clinical outcomes, which results in a decrease in ICU mortality rates. This effect is more pronounced when the staff are dedicated and specifically trained to care for critically ill patients. Furthermore, an increase in ICU staff numbers has a cost saving benefit to both the patient and the healthcare institution. Increased staff numbers result in a decline in patients' lengths of stay hence there is a drop in the risk of hospital related complications including hospital-acquired infections and also the cost of laboratory tests, hospital procedures and specialized medication. It is against this background that the Paediatric Critical Care Nursing program (PCCN) was launched in Nairobi, Kenya.

**Objectives**

The program aims to train 30 nurses per year (two intakes per year of 15 nurses each) as per the Nursing Council of Kenya guidelines so as to build the capacity of critical care nurses in East Africa.

**Method**

The PCCN training program is tailored for state registered nurses or Bachelor of Science in Nursing graduates who wish to specialize in critical care environments; critical care of neonates, infants, children and adolescents along the critical care continuum across a variety of health services, from resuscitation, critical stabilization, transfer and vigilant and responsive care through the often turbulent and dynamic period of critical illness in the Paediatric Intensive Care Unit (PICU), Neonatal Intensive Care Unit (NICU) and High Dependency Unit (HDU). Training is over a 52-week period and includes theory (16 weeks) and critical nursing experience (36 weeks). Additional courses include EPLS (European Advanced Pediatric Life Support), ETAT+ (Emergency Treatment and Triage) and PEWS chart (Pediatric early warning score).

**Results**

A total of 45 nurses from 22 healthcare institutions across East Africa have benefitted from the training since the launch of the program in 2013 to-date; four (4) nurses from Rwanda, one (1) from Uganda and forty (40) from Kenya. Of the nurses trained nine (9) were male and thirty six (36), female with a mean age of thirty seven (37) years. The graduates have taken up leadership positions and are now using the skills acquired to improve paediatric critical care through evidence based clinical practices, besides the management of critical care units and translation of strategic health care policies to tactical and operational plans for effective implementation for positive outcomes in national healthcare systems.

**Conclusions**

These efforts are in line with the United Nations Convention on the Rights of a Child, which is in place to ensure quality healthcare for all children globally.

### A277 Assessing nursing and healthcare professional's perceptions of a stress reduction massage intervention

#### R. Kleinpell, S.J. Sarris, P. Nedved, M. Heitschmidt

##### Rush University Medical Center, Chicago, United States

###### **Correspondence:** R. Kleinpell – Rush University Medical Center, Chicago, United States

**Introduction**

It is well acknowledged that the intensive care unit (ICU) environment can create a stressful work atmosphere for clinicians. Promoting a healthy work environment for ICU settings has been identified as a way to reduce work-related stress in the ICU. As part of an overall initiative to enhance the work environment in the ICU at a 620 bed university affiliated medical center, an employee appreciation day was designated and 10 minute massages were provided to clinical staff.

**Objective**

The objective of this study was to assess the impact of a massage intervention on self-reported stress ratings.

**Method**

The Numeric Stress Scale, an established Likert scale ranging from “0” or “No Stress” to “10” or “Unbearable Stress” was used to collect self-assessed perceptions of stress ratings. A 10 minute massage was provided by certified massage therapists using portable massage chairs set up in a conference room.

**Results**

A total of 182 nurses and other clinicians completed a pre-post assessment of self- reported levels of stress. Before the massage, clinicians rated their own level of stress as being, on average, 6 out of ten (moderate). After the 10 minute massage, stress ratings were found to decrease, with over one third of respondents reporting low levels of stress [0 or 1], and 30 % of respondents reporting “no stress”. The number of clinicians reporting high levels of stress [9 or 10] decreased from 11 to 3 after the massage intervention. An independent samples T-test was used to compare the pre (mean = 6.15, standard deviation 2.02) and post (mean = 2.25, standard deviation 2.14) stress ratings. The change in self-reported levels of stress was found to be a statistically significant decrease (t-statistic = 12.5, df 181, p < .000).

**Conclusion**

A 10 minute massage intervention was found to result in decreased levels of self-reported stress levels in ICU clinicians in this single center study. Exploring the impact of stress reducing interventions for ICU clinicians requires further investigation as part of an overall focus in promoting a healthy work environment in the ICU.

### A278 Management and outcome of diabetic ketoacidosis in a Tunisian intensive care unit: where are we?

#### H. Ben-Ghezala, S. Snouda, S. Djobbi

##### University of Tunis El Manar, Teaching Department of Emergency and Critical Care Medicine, Zaghouan, Tunisia

###### **Correspondence:** H. Ben-Ghezala – University of Tunis El Manar, Teaching Department of Emergency and Critical Care Medicine, Zaghouan, Tunisia

**Introduction**

Diabetic ketoacidosis (DKA) is a major complication of diabetes mellitus. It is more and more frequent in Tunisia with an incidence ranging from 4 to 8 per 1000 diabetic patients. There are many insulin administration protocols and many unresolved questions : the place of alkalinization and the signification of hyperchloremic acidosis.

**Objectives**

The aim of this work was to evaluate the management of DKA in a Tunisian medical intensive care unit and to determine the prognosis factors in our diabetic patients.

**Method**

We conducted a retrospective analysis over two years (January 2013 - December 2015), of records of patients who were admitted to our intensive care unit (ICU). Diabetic ketoacidosis was diagnosed by the biochemical triad: ketonaemia, hyperglycemia and acidaemia. We picked up all the clinical and laboratory data, fluid replacement, complications and disease progression.

**Results**

52 patients were enrolled. The prevalence of ketoacidosis was 3 % and nearly 0.02 % of all patients who attended our emergency department. The mean age was 36 ± 21 years with a sex ratio of 0.4. 70 % of our patients had type 1 juvenile diabetes and 15 % had type 2 diabetes. The other 15 % had inaugural ketoacidosis. The mean SAPS II score was 24 ± 15. Asthenia was the most frequent clinical sign (71 %). 61 % of patients expressed digestive symptoms. Nine patients had altered vital signs with mainly a shock and nine other patients had altered mental status. 40 patients had hyper leukocytosis. All patients had both venous and capillary glycaemia. The mean capillary glycaemia was 5, 16 ± 1, 40 g/l. Twenty two patients (40 %) had pseud hyponatremia. Seven patients (13 %) presented hyperkaliemia at admission. The mean c-reactive protein level was 15,28 ± 23,81 mg/l. The mean level of bicarbonate value was 10,68 ± 5 mEq/l at admission. The mean overall osmolarity was 318 ± 16 mEq/l. 28 patients were infected (54 %). All patients had a close bedside monitoring of blood glucose, blood gas and blood electrolyte measurement according to our local protocol management which will be detailed in this work. The fluid replacement therapy was based on crystalloids. Three patients received alkalinization. Four patients presented a coronary syndrome. Five patients developed hyperchloremic acidosis. The overall mortality rate was 13.5 % (7 patients). The mean hospital stay in our ICU was 37,8 ± 22,7 hours. Factors associated with mortality were type 1 of diabetes (p = 0.004), SAPS 2 (p < 10^-6^), shock ((p < 10^-6^), and acute renal failure (ARF)(p < 10^-6^) in univariate analysis. Only SAPS 2 and ARF were independently associated with a poor outcome in multivariate analysis.

**Conclusions**

The clinical assessment and aims of treatment in the management of DKA are similar to literature. But, many controversial points about the optimum treatment regimen, the fluid to use, alkalinization and the management of hyperchloremic acidosis are discussed in this work.

### A279 NT-proBNP levels and echocardiography in the etiologic diagnosis of acute dyspnea: a prospective study

#### H. Ben-Ghezala, S. Snouda

##### University of Tunis El Manar, Teaching Department of Emergency and Critical Care Medicine, Zaghouan, Tunisia

###### **Correspondence:** H. Ben-Ghezala – University of Tunis El Manar, Teaching Department of Emergency and Critical Care Medicine, Zaghouan, Tunisia

**Introduction**

Both NT- proBNP and Doppler echocardiography have been approved in the diagnosis of heart failure. In our Study, we compared the contribution of the NT-proBNP levels with the Doppler echocardiography findings in the diagnosis of decompensated congestive left-heart failure (CHF) in patients with acute dyspnea.

**Objectives**

To compare NT-proBNP levels and echocardiography finding in the determination of the etiology of difficult-to-diagnose severe dyspnea in the emergency department.

**Method**

It was a prospective, observational study at the teaching department of emergency and intensive care in the regional hospital of Zaghouan, including patients with severe dyspnea over six months. All patients underwent physical examination, 12-lead ECG, RX Thorax, NT-ProBNP essay and echocardiography by an attending cardiologist on admission. The accuracy of the two Method for etiologic diagnosis was compared on the basis of the final diagnoses established by the medical staff.

**Results**

65 patients were enrolled, including 45 (69 %) with CHF. Diagnosis of CHF was due to coronary artery disease, hypertension, valve disease, arrhythmia and dilated cardiomyopathy. Non-CHF was due to decompensated chronic obstructive pulmonary disease, pneumonia, and severe asthma. Fifteen patients (23 %) were misdiagnosed at admission. The mean NT-proBNP concentration was 8989 [769 to 18945] pg/ml in the CHF subgroup and 462 [22 to 1589] pg/ml in the other patients (p < 0.01). Systolic LV dysfunction (LVEF < 0.45) was found in 31 patients with CHF (60 %) and in 7 patients with other causes of dyspnea (15 %) (p < 0.01). The E/A ratio and the deceleration time of E-wave (DT) were respectively 1,85 ± 0,77 and 12o ± 23 in the group CHF; 0,81 ± 0,44 and 208 ± 47 in the group Non-CHF (p < 0.01). Impaired relaxation and Restrictive mitral pattern were observed respectively in 28 % and 30 % of the patients with CHF and in only two patients and th + ree patients in the other group.

**Conclusions**

Both NT-proBNP assay and echocardiography can be used for the diagnosis of CHF inacutely dyspneic patients. However, the echocardiography is more accurate in patients with intermediate BNP levels.

**Note:** This abstract has been previously published and is available at [1]. It is included here as a complete record of the abstracts from the conference.

**References**

1. Snouda S, Ben Ghezala H, Abbes MF, Daoudi R, Kaddour M, Benchiekh I (2016). NT-proBNP levels and echocardiography findings in the etiologic diagnosis of acute dyspnea. Annals of Intensive Care 6(Suppl 1): P221.

### A280 Cough augmentation techniques for extubation and weaning critically ill patients from mechanical ventilation

#### L. Rose^1^, N.K.J. Adhikari^2^, D. Leasa^3^, D. Fergusson^4^, D.A. Mckim^5^

##### ^1^University of Toronto, Toronto, Canada; ^2^Sunnybrook Health Sciences Centre, Toronto, Canada; ^3^London Health Sciences Centre, London, Canada; ^4^Ottawa Hospital Research Institute, Ottawa, Canada; ^5^Ottawa Hospital, Ottawa, Canada

###### **Correspondence:** L. Rose – University of Toronto, Toronto, Canada

**Introduction**

Despite decades of research, predictors of weaning and extubation failure remain unclear, although ineffective cough and secretion retention likely play a role. Cough augmentation techniques (lung volume recruitment [LVR], manually assisted cough [MAC], and mechanical insufflation-exsufflation [MI-E]) are used to prevent and manage respiratory complications of chronic disease and may improve short- and long-term outcomes for patients experiencing acute respiratory failure. The role of cough augmentation to enable extubation and prevent post-extubation respiratory failure for critically ill patients with acute respiratory failure is unclear.

**Objectives**

To determine extubation success, duration of ventilation, ICU stay, mortality, and harm using cough augmentation techniques compared to no cough augmentation for critically ill adult and paediatric patients with acute respiratory failure.

**Method**

Systematic review of trials evaluating LVR, MAC, or MI-E compared to a control group without this intervention. We considered non-randomized studies [NRS] for harm evaluation only. Two authors independently screened electronic databases to April 2016 and abstracted data on a standardized form with accuracy verified by a 3rd author.

**Results**

We screened 2686 references, identified 23 for full text review and excluded 15. Included studies comprised 2 RCTs (112 participants); 1 evaluated MI-E + MAC, the other LVR + MAC. NRS included 1 case-control study and 5 large case series. One trial reported greater extubation success (no need for reintubation for 48 hours) in the MI-E + MAC group (82.9 % vs 52.5 %, relative risk [RR] 1.58, 95 % CI 1.13 to 2.20). One trial reported a statistically significant reduction in mechanical ventilation favouring MI-E + MAC (mean difference -6.1 days, 95 % CI -8.4 to -3.8). Two trials reporting on ICU stay and mortality demonstrated no difference. Harm reported in the 2 RCTs comprised 2 intervention patients experiencing hemodynamic compromise compared to no control arm patient (RR 3.2, 95 % CI 0.35 to 29.2). In the trial of MI-E + MAC, 9 (23 %) control group patients compared to 2 (6 %) MI-E + MAC patients experienced secretion retention and severe hypoxemia warranting reintubation (RR 0.25, 95 % CI 0.06 to 1.10). No patient in either trial experienced new onset arrhythmia or pneumothorax. Of the 5 case series, 2 studies (33 patients) evaluating MAC during invasive ventilation reported no complications (hemodynamic compromise, pneumothorax, hypoxemia, mucus plugging). In the 3 case series that evaluated MI-E + MAC (292 patients considered non-weanable), no complications were reported; however, 14/37 (38 %), 8/157 (5 %) and 8/98 (8 %) required reintubation due to plug related desaturation after extubation (harm not reported in case control study).

**Conclusions**

Data on the efficacy of LVR, MAC, and MI-E to promote extubation success are limited though complications appear to be uncommon.

**Grant acknowledgment**

CIHR.

### A281 A pilot study of prehabilitation and enhanced post-operative physiotherapy for patients undergoing oesophagectomy and total gastrectomy (TG)

#### J. Weblin^1^, O. Tucker^2^, D. McWilliams^1^

##### ^1^University Hospitals Birmingham NHS Foundation Trust, Physiotherapy, Birmingham, United Kingdom; ^2^University Hospitals Birmingham NHS Foundation Trust, Birmingham, United Kingdom

###### **Correspondence:** J. Weblin – University Hospitals Birmingham NHS Foundation Trust, Physiotherapy, Birmingham, United Kingdom

**Introduction**

Major oesophagogastric surgery is associated with significant postoperative morbidity, reduced functional capacity and quality of life[1]. Evidence from colorectal surgery populations demonstrates significant benefits of prehabilitation[2]. There is no evidence to support this approach for patients undergoing oesophagectomy and TG.

**Objective**

To assess the impact of implementing prehabilitation and enhanced post-operative physiotherapy in patients undergoing oesophagectomy and TG.

**Method**

Consecutive patients from November 2014 to March 2015 were entered into a standardised ERAS programme incorporating twice daily physiotherapy for five days postoperatively with pre-specified mobility milestones. Of these, 17 were eligible and offered prehabilitation, incorporating twice-weekly cardiovascular and strengthening classes for 4 weeks, of which 13 attended. Outcomes measured were functional capacity by incremental shuttle walk test (ISWT), anxiety and depression by HADS score, and perceived health status by EQ5D. We also compared data for those who attended prehabilitation to those who received ERAS only post operatively. Post-operative outcomes were time to mobilise 30 m, mobility level at critical care discharge by MMS and total hospital length of stay (LOS).

**Results**

Thirteen patients attended prehabilitation, with improvements seen for all outcomes on completion (see Table 86).

Post operatively, patients who attended prehabilitation were more mobile at ICU discharge (MMS 7 vs 6) and quicker to mobilise 30 m (3.2 vs 4.7 days). A reduction in LOS was also seen for prehabilitation patients in comparison to those receiving ERAS alone (14.7 days vs 17.5 days).

**Conclusion**

Implementation of a programme of prehabilitation is feasible in patients undergoing major oesophagogastric surgery and was associated with improved outcomes preoperatively. These improvements were associated with a higher level of mobility at the point of critical care discharge and shorter hospital length of stay. Future appropriately powered research is needed to confirm these findings.

**References**

1. McCulloch P, Ward J, Tekkis PP, et al. Mortality and morbidity in gastrooesophageal cancer surgery: initial results of ASCOT multicentre prospective cohort study. BMJ. 2003;327:1192-1197

2. Mayo NE et al 2011. Impact of preoperative change in physical function on post-operative recovery: argument supporting prehabilitation for colorectal surgery. Surgery 2011 Sep; 150(3) 505

**Table 86 (abstract A281).** Prehabilitation outcomes

	Initial Assessment	Post Prehab	Difference
ISWT (m)	401	515	114 (28%)
Anxiety	5.1	4.2	-0.9
Depression	4.2	2.5	-1.7
EQ5D	70	82	+12%

### A282 Improved usability of a multi-infusion setup using a central control display

#### F. Doesburg^1^, F. Cnossen^2^, W. Dieperink^1^, W. Bult^3^, M.W.N. Nijsten^1^

##### ^1^University Medical Center Groningen, Department of Critical Care, Groningen, Netherlands; ^2^University of Groningen, Artificial Intelligence, Groningen, Netherlands; ^3^University Medical Center Groningen, Hospital Pharmacy, Groningen, Netherlands

###### **Correspondence:** F. Doesburg – University Medical Center Groningen, Department of Critical Care, Groningen, Netherlands

**Introduction**

Infusion pumps are often associated with poor usability and an increased likelihood of medication errors [1]. Critically ill patients in the intensive care unit (ICU) usually receive multiple infusions simultaneously, which increases the likelihood of pump-related errors. Furthermore, the ICU is frequently a hectic environment, compounding the likelihood of human error. Improving the usability of infusion pumps could help prevent injury or death in this vulnerable population. Where previous usability studies focused on individual pumps, this study focuses on the pump system as a whole, a situation more representative of the ICU.

**Objectives**

The aim of this study is to develop and test a pump display that facilitates centralized monitoring and control of multiple infusion pumps to improve the safety and efficiency in the interaction with infusion technology.

**Method**

A central pump display was developed according to the latest guidelines in user-centered design [2]. The usability of this display was compared to that of a conventional pump setup in a simulation study where 18 ICU nurses performed common pump-related tasks with either the central or conventional interface. Participants had a mean of 12 (±12) years of ICU work experience. Task execution times and key presses were recorded and logs were scanned for errors after the experiment. A usability questionnaire with a 5-point Likert scale was administered at the end of each experimental run to assess end-user satisfaction.

**Results**

Overall there was no difference in total task execution time between the central (M = 421 ± 107 seconds) and conventional system (M = 405 ± 119), p = 0.78. Fewer clicks were needed to execute the tasks with the centralized system compared to the conventional system (40 ± 3 and 73 ± 20 clicks respectively), p = 0.001. Fewer errors were made with the centralized system compared to the conventional system (0.9 ± 1 and 2.9 ± 2 errors respectively), p = 0.031. Questionnaire results indicated an overall preference towards the centralized system (4.6 ± 0.3 vs. 4.1 ± 0.5), p = 0.033.

**Conclusions**

Expert users with significant experience using the conventional pump interface demonstrated a more efficient and safer performance using the central control display. The central control display had a better overall usability than the conventional pump control interface, despite participants never having used it before. The results of this study underline the importance of usability testing in the improvement of high-risk medical systems.

**References**

1. Husch M, Sullivan C, Rooney D, et al (2005) Insights from the sharp end of intravenous medication errors: implications for infusion pump technology. Qual Saf Health Care 14:80-6.

2. Zhang J, Johnson TR, Patel VL, et al (2003) Using usability heuristics to evaluate patient safety of medical devices. J Biomed Inform 36:23-30.

**Grant acknowledgment**

This study was supported by a UMCG Healthy Ageing grant.

## INFECTIONS AND PERIOPERATIVE CARE

### 0283 Renal angina as a predictor of renal failure in adult patients admitted in a mixed ICU

#### G.A. Galvez-Blanco, E. Monares Zepeda, C.I. Olvera Guzman, J.S. Aguirre Sánchez, J. Franco Granillo

##### ABC Medical Center, Intensive Care Unit, Mexico City, Mexico

###### **Correspondence:** G.A. Galvez-Blanco – ABC Medical Center, Intensive Care Unit, Mexico City, Mexico

**Introduction**

Renal failure in a patient, whether it develops during hospitalization or admission, is an independent predictor of mortality so efforts to detect it earlier in an ICU setting should be performed in all patients in order to set up preventive and therapeutic maneuvers. Despite well established diagnostic criteria for acute kidney injury (AKI), the detection is often delayed in the clinical practice. When making the analogy with cardiac angina, even though several biomarkers have been developed for an early detection of AKI none of them have the sensitivity and specificity of troponin levels in a cardiac setting. Therefore, the concept of renal angina was developed considering risk factors and clinical signs of renal failure, scoring patients in 4 groups according to the need of Continuous Renal Replacement Therapy (CRRT): very high, high, moderate and low risk. In pediatric ICU populations this concept has been widespread, not as well in adult ICU patients.

**Objectives**

To present a pilot study where we describe 8 patients who required CRRT in the last month and determine the risk for the presence of renal angina at admission.

**Method**

Descriptive pilot study. We retrospectively analyzed all adults patients who required CRRT during March 2016 in which all data needed to evaluate the risk for renal angina was completed. We obtained and interpreted their demographics and laboratory results as well as their pre-medical history.

**Results**

All patients were male, with a mean age of 74 ± 12 (55-89). 3 patients died. The most common diagnosis was community acquired pneumonia (50 %), and second was acute on chronic cardiac failure (25 %). At admission they were all classified in the very high risk group. Also, the 8 patients had one or more mayor chronic criteria. The most frequent risk factors were hypertension history or hypotension at admission (75 % respectively), age and sepsis (62.5 % respectively). All patients had early elevation of serum creatinine (sCr) of at least 0.1 mg/dl. Average days between admission to the ICU and beginning of CRRT was 3.

**Conclusion**

Determining renal angina score in patients admitted to the ICU might be a cornerstone for treatment and prevention of renal failure, especially in patients within the very high and high risk groups. This will allow clinicians to be very suspicious of the slightest sCr elevation (as low as 0.1 mg/dl), as it can predict the need for CRRT, and thus establish preventive maneuvers or initiate replacement therapy early.

**References**

1.- Chawla et al. Renal angina: concept and development of pretest probability assessment in acute kidney injury. Critical Care (2015) 19:93.

2.- Cruz DN, Ferrer-Nadal A, Piccinni P, Goldstein SL, Chawla LS, Alessandri E, et al. Utilization of small changes in serum creatinine with clinical risk factors to assess the risk of AKI in critically ill adults. Clin J Am Soc Nephrol. 2014; 9:663-72.

**Fig. 108 (abstract 0283).**
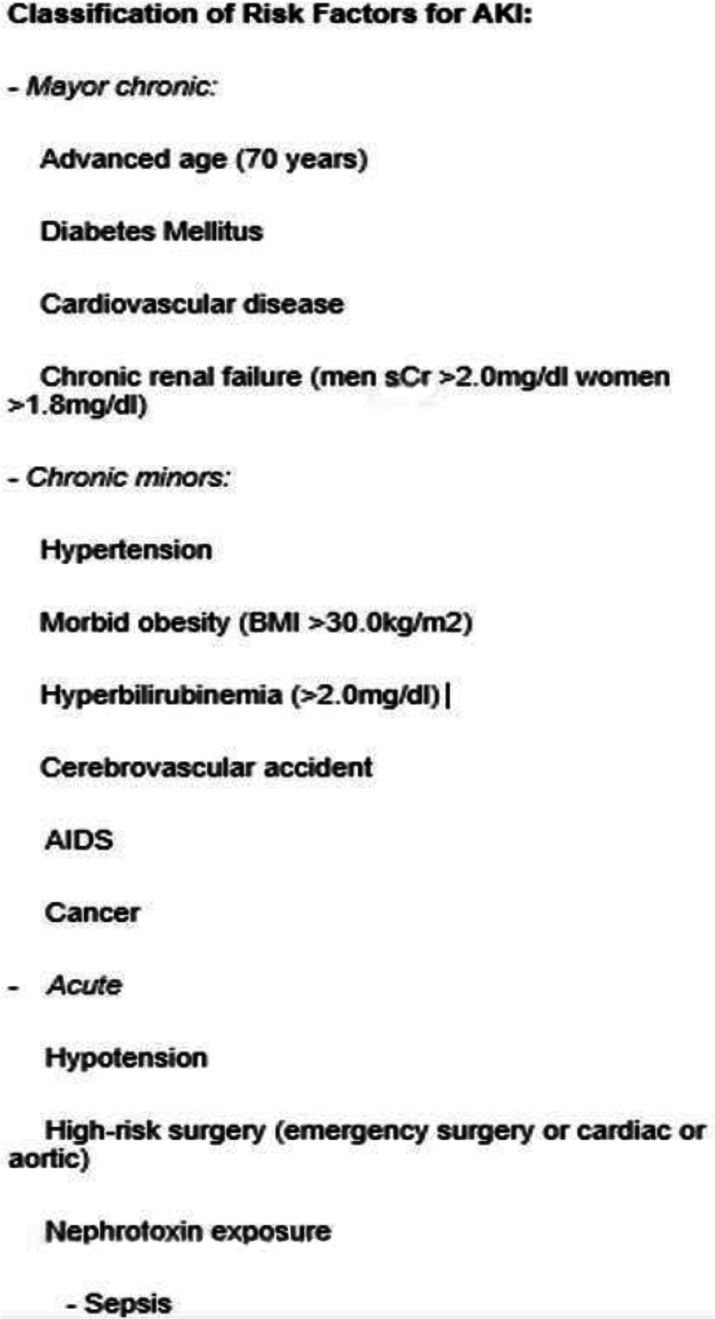
Renal Angina Criteria

**Fig. 109 (abstract 0283).**
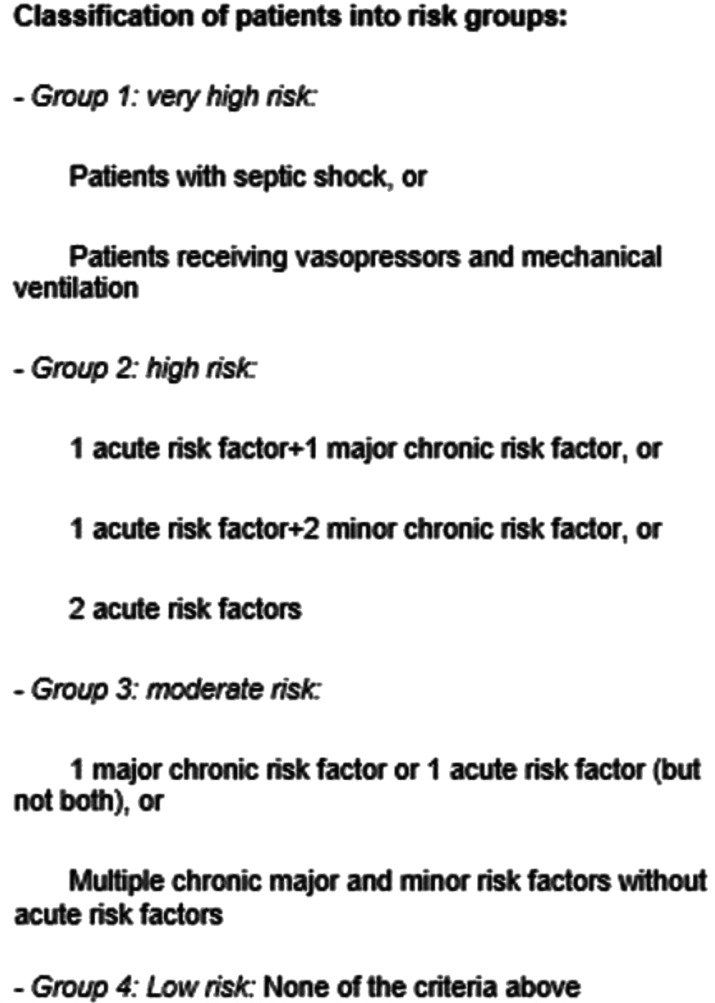
Classification of risk groups

### A284 Incidence and recovery of acute kidney injury after cardiac surgery

#### J. Santos Stroud^1^, R. Thomson^2^

##### ^1^St Georges NHS Foundation Trust, Cardiothoracic Intensive Care, London, United Kingdom; ^2^St Georges NHS Trust, London, United Kingdom

###### **Correspondence:** J. Santos Stroud – St Georges NHS Foundation Trust, Cardiothoracic Intensive Care, London, United Kingdom

**Introduction**

Acute kidney after cardiac surgery is well described [1]. AKI is associated with an increase in morbidity and mortality both in the short and long term [2]. The severity of AKI is directly proportional to outcome [3] and is independently associated with mortality, specifically if sustained injury occurs [4]. In 2012 we implemented a protocol post cardiac surgery to ensure adequate fluid resuscitation as a strategy to reduce complications, namely AKI. The incidence of AKI was reduced from 19.9 % to 6.5 %.

**Objectives**

To ensure our efforts to reduce the incidence of AKI were still effective.

**Method**

Pre-operative baseline and peak creatinine data is prospectively recorded by our audit team to monitor the incidence of AKI. Baseline characteristics were collected along with the discharge creatinine, incidence of CVVH, length of ICU and hospital stay, ICU readmissions and mortality.

**Results**

Over 6 month period, 431 cardiac surgical admissions occurred in CTICU of those 9.28 % (n = 40) sustained AKI using the KDIGO classification. Of those 4.87 % (n = 20) sustained stage I, 1.62 % (n = 7) sustained stage II, and 2.78 % (n = 12) sustained stage III. 12.5 % (n = 5) died. 10 % (n = 4) were re-admitted to ICU and 44 % (n = 15) of those surviving, the AKI had resolved prior to hospital discharge.

**Conclusions**

The incidence of AKI remained below the baseline from 2012. Though resolution of AKI did not occur in most.

Further follow up is required by GP's to ensure further injury doesn't occur and to highlight caution. There was no evidence of AKI within the surgical discharge summary. Our recommendation is to write ICU discharge letters including such events. Although the incidence of AKI has remained lower, AKI still occurs. Identifying patients at risk for AKI during pre-op assessment is recommended. Modifiable risk factors should be optimized and high risk patients should be identified prior to cardiac surgery as recommended in the literature to reduce this complication further.

**References**

1. Rosner MH. Okusa MD. Acute Kidney Injury Associated with Cardiac Surgery. Clin J Am Soc Nephrol 1: 19-32, 2006. doi: 10.2215/CJN.00240605

2. Corredor C. Thomson R. Al-Subaie N. Long-Term Consequences of Acute Kidney Injury After Cardiac Surgery: A Systematic Review and Meta-Analysis. J Cardiothorac and Vasc Anaesth.

3. Vanderberghe W. Gevaert S. Kellum JA, Bagshaw SM. Peperstraete h. Herck I. Decruyenaere J. Hoste EA. Acute Kidney Injury in Cardiorenal Syndrome Type 1 Patients: A A Systematic Review and Meta-Analysis. NCBI Cardiorenal Med 2016 Feb; 6(2):116-28.doi:10.1159/000442300.

4. Hoste EA. De Corte w. Epidemiology of AKI in the ICU. NCBI Acta Clinica Belgica 2007; 62 Suppl 2:314-7.

5. Kidney Disease: Improving Global Outcome (KDIGO) Acute Kidney Injury Work Group. KDIGO Clinical Practice Guideline for Acute Kidney Injury. Kidney inter., Suppl. 2012; 2: 1-138.

### A285 Risk factors and head-of-bed elevation on pressure ulcers related to patient positioning: results from CAPCRI study

#### M. Llaurado-Serra^1,2^, A. Lobo-Civico^3^, M. Pi-Guerrero^4^, I. Blanco-Sanchez^5^, A. Piñol-Tena^6^, C. Paños-Espinosa^7^, Y. Alabart-Segura^8^, B. Coloma-Gomez^8^, A. Fernandez-Blanco^3^, F. Braga-Dias^5^, M. Treso-Geira^9^, A. Valeiras-Valero^4^, L. Martinez-Reyes^7^, A. Sandiumenge^10^, M.F. Jimenez-Herrera^1^, CAPCRI Study

##### ^1^Rovira i Virgili University, Nursing Department, Tarragona, Spain; ^2^Joan XXIII University Hospital, Tarragona, Spain; ^3^Dr Josep Trueta University Hospital, Intensive Care Unit, Girona, Spain; ^4^Hospital de Sant Joan Despí Moissès Broggi, Intensive Care Unit, Barcelona, Spain; ^5^Quiron Salud-Hospital General de Catalunya, Intensive Care Unit, Barcelona, Spain; ^6^Verge de la Cinta University Hospital, Intensive Care Unit, Tortosa, Spain; ^7^Sant Pau I Santa Tecla University Hospital, Intensive Care Unit, Tarragona, Spain; ^8^Joan XXIII University Hospital, Intensive Care Unit, Tarragona, Spain; ^9^Verge de la Cinta University Hospital, Intensive Care Unit, Tarragona, Spain; ^10^Vall d´Hebrón University Hospital, Barcelona, Spain

###### **Correspondence:** M. Llaurado-Serra – Rovira i Virgili University, Nursing Department, Tarragona, Spain

**Background**

Pressure ulcers (PU) are a frequent adverse event in intensive care unit (ICU). The main risk factor for their development is patient's immobility. This analysis is part of a study named CAPCRI which had the objective to determine the factors related to the head-of-bed elevation (HOBE) in patients with mechanical ventilation.

**Objective**

To investigate the risk factors for the development of the PU related to the patient positioning in critically ill patients with mechanical ventilation and the effect of the presence of a pressure ulcer in HOBE.

**Method**

Descriptive, longitudinal and multicentre study in 6 Spanish ICUs from March to December 2013. Inclusion criteria were: patients >18 years old, mechanical ventilation >48 h and being able to measure HOBE during the first 24 hours of mechanical ventilation. Patients with contraindications for HOBE 30-45° were excluded. Sample size calculation resulted in 50 patients/center. PUs were diagnosed according to NPUAP/EPUAP guidelines. Descriptive, bivariate and multivariate analyses were performed. Significance level was set at p < 0.05.

**Results**

25 patients (9 %) out of the 276 included developed PU representing 34 PU. The most frequent diagnose was surgical (52 %). Patients were admitted to the ICU for a median of 22 (11-35) days and 16 (9-26) days under mechanical ventilation. 68 % of the patients developed only 1 PU. The mean admission time until PU diagnosis was 10.6 (10.3) days. The majority of PU were Stage I and Stage II (35.3 % and 44.1 % respectively) and diagnosed in sacrum and heel (52.9 % and 41.2 %).

In multivariate logistic regression analysis the independent risk factors for PU were the sedation time [OR 1.018 (CI95% 1.009-1.027)], vasoactive drugs duration [OR 1.023 (CI95% 1.015-1.031)] and time with a mean arterial pressure < 70 mmHg [OR 1.016 (CI95% 1.005-1.026)]. On the contrary, the factors preventing PU were a higher APACHE II Score [OR 0.876 (CI95% 0.833-0.920)] and higher values of albumin and pre-albumin [OR 0.617 (CI95% 0.388-0.982); OR 0.873 (CI95% 0.822-0.927)] respectively. The degree of HOBE was not statistically significant [OR 0.96 (CI95%0.90-1.02)].

When analyzing the impact of the presence of a PU, it did not affect HOBE as it remained similar to previous (before diagnosis mean 29.6 (4.6)° vs after diagnosis mean 29.9 (4.0)°; p = 0.677).

**Conclusions**

9 % of the patients analysed developed PU related with body positioning. The degree of HOBE was not evidenced as a risk factor for PU. The hypotension, the duration of the sedation and the vasoactive drugs are important risk factors for the development of PU. Besides other preventive measures, maintaining adequate values of albumin and pre-albumin is important to prevent PU. The presence of PU did not affect the attitude of the nurses towards the degree of HOBE.

**Grant acknowledgment**

The project was funded by the 14° National Award of Nursing Research from Marques de Valdecilla Hospital (Spain).

### A286 Urinary vs. plasmatic NGAL as a predictor of acute renal failure in patients undergoing cardiac surgery

#### R. Prada^1^, P. Juárez^1^, R. Argandoña^1^, J.J. Díaz^1^, C. Sánchez Ramirez^1^, P. Saavedra^2^, S. Ruiz Santana^1^

##### ^1^Hospital Universitario de Gran Canaria Dr Negrin, Las Palmas de Gran Canaria, Spain; ^2^Universidad de Las Palmas de Gran Canaria, Las Palmas de Gran Canaria, Spain

###### **Correspondence:** R. Prada – Hospital Universitario de Gran Canaria Dr Negrin, Las Palmas de Gran Canaria, Spain

**Objectives**

To assess NGAL as a predictor of acute kidney injury in patients undergoing cardiac surgery.

**Method**

Prospective, longitudinal study involving ICU patients undergoing cardiac surgery without renal disease. Creatinine and plasma and urinary NGAL level determinations were performed at 0, 2, 6 and 12 hours post ICU admission. NGAL cut-off was considered to predict acute renal failure when above 137 ng/mL and 131.7 ng/mL in plasma and urine, respectively. Statistical analysis: Patients were classified according to presence or absence of renal failure. Categorical variables were summarized in frequencies and percentages and numerical in means and standard deviations (SD) or medians and interquartile ranges (IQR). The percentages were compared, as appropriate, with the X2 test or Fisher´s exact test, mean the t-test and medians with the Wilcoxon test for independent groups. In order to evaluate the diagnostic potential of NGAL (in urine and plasma) for renal failure ROC (Receiver Operating Characteristics) analysis for each of the markers was performed. The predictive ability of the markers was assessed through areas under the corresponding ROC curves, which were estimated by 95 % confidence intervals. Optimal cutoff for each of the markers was considered those ones that corresponded to the sensitivity and specificity values that minimized the following expression: . For these cut-off sensitivity, specificity, positive predictive value, negative predictive value, positive likelihood ratio and negative likelihood ratio were obtained. These parameters were estimated by 95 % confidence. A hypothesis test was considered statistically significant when the corresponding p-value was less than .05 and the obtained area under the two ROC curves were also compared.

**Results**

**Fig. 110:**
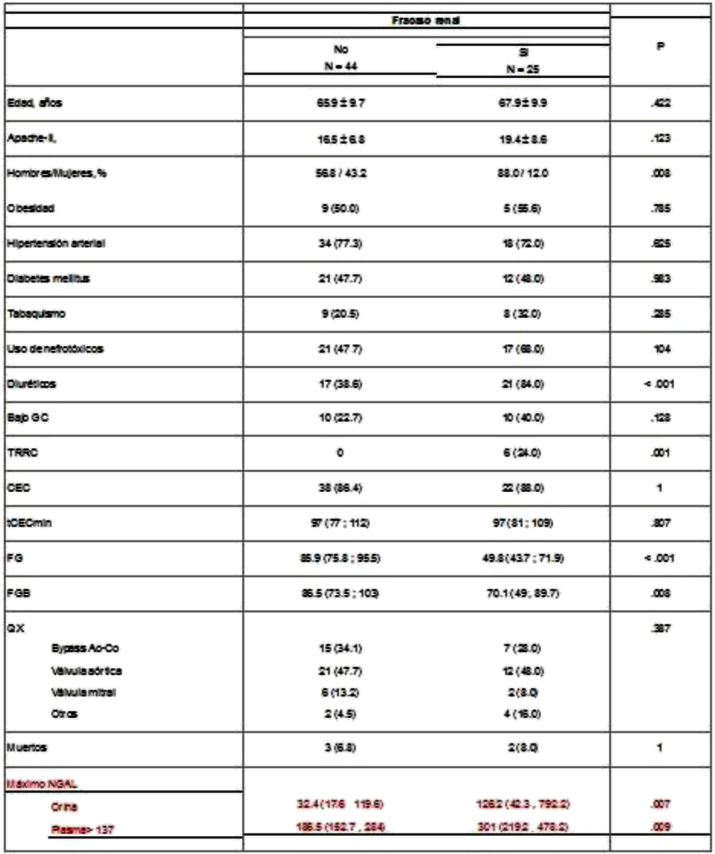
(abstract A286).

Figure 110 shows the variables of the study in the groups defined as presence or absence of renal failure. The behavior of the variables analyzed is similar in both study groups.

**Conclusion**

NGAL is a good marker of early acute renal injury in patients after cardiac surgery and it did not show statistically significant differences when comparing urine vs. plasma levels.

**Fig. 111:**
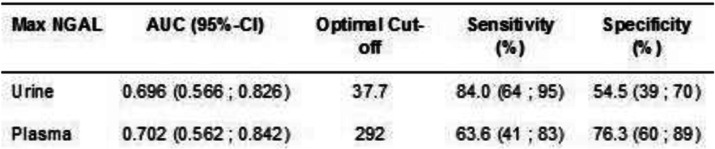
(abstract A286).

**Fig. 112 (abstract A286).**
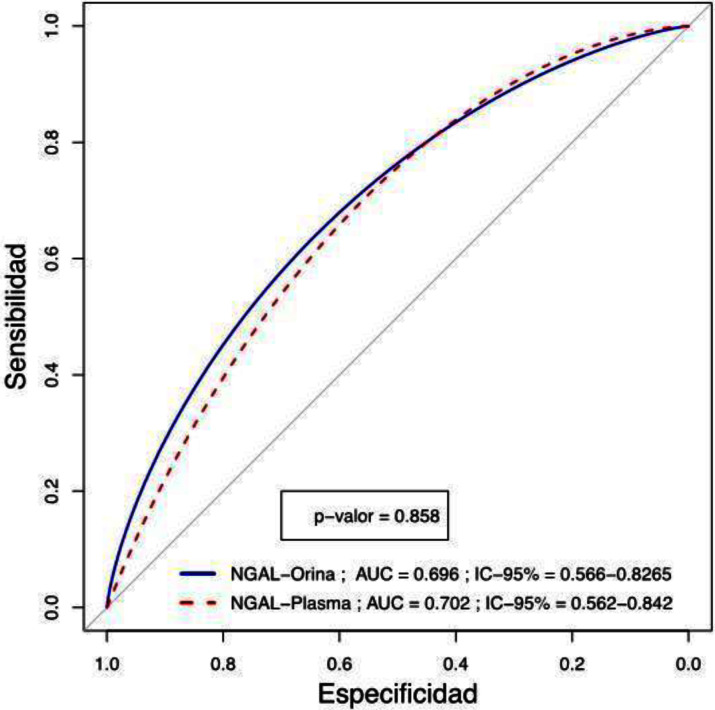
ROC`s comparative

### A287 Sensitivity and specificity of new clinical criteria of sepsis (QSOFA) in patients receiving anticancer therapy

#### O. Obukhova, S. Kashiya, I.A. Kurmukov, A.M. Pronina

##### N.N. Blokhin Russian Cancer Research Center, Medical ICU, Moscow, Russian Federation

###### **Correspondence:** O. Obukhova – N.N. Blokhin Russian Cancer Research Center, Medical ICU, Moscow, Russian Federation

**Introduction**

Immediate diagnosis of sepsis in critical conditions of cancer patients can be complicated due to the similarity between the manifestations of drug toxicity and systemic infectious complications. Generally this category of patients have overdiagnosis of sepsis in case of the SIRS criteria use [1]. New clinical criteria for the sepsis diagnosis were developed (S-3) [2], but the rationale of their use in patients receiving anticancer therapy is not yet determined.

**Objectives**

To determine the sensitivity and specificity of the new sepsis clinical criteria in patients who received anti-cancer therapy

**Method**

A total of 48 records of patients having 2 or more SIRS criteria (S-2) that admitted to therapeutic ICU in 2014-2015 were subjected to a retrospective analysis. All patients had anticancer drug-related adverse events of various grade. The diagnosis of sepsis (criteria SIRS + defined infectious lesion ± defined infectious agent) was determined in 35 (72.9 %) of cases. Retrospectively, all patients met the S-3 criteria. The sensitivity, specificity and accuracy of S-3 criteria were calculated. The calculation of 95 % confidence interval (CI) was carried out by means of the Wilson Method

**Results**

At the time of the ICU admission 34 patients met the S-3 criteria. Sepsis was further confirmed in 29 (85.3 %) patients. S-3 sensitivity was 0.83 (95%CI 0.67-0.92), S-3 specificity was 0.62 (95%CI 0.36-0.82) and S-3 accuracy - 0.77 (95%CI 0.53-0.97). S-2 sensitivity was 0.73 (95%CI 0.59-0.83).

**Conclusions**

New clinical criteria of sepsis have sufficient specificity and high sensitivity in patients receiving anticancer therapy.

**References**

1. Levy MM, Fink MP, Marshall JC, et al. Intesive Care Med. 2003;29(4):530-538.

2. Singer M., Deutschman CS, Seymour ChW, et al. JAMA. 2016;315(8):801-810.

**Grant acknowledgment**

None.

### A288 A five years longitudinal magnetic resonance imaging study of severe traumatic brain injury patients. Correlation with functional outcome

#### P. Simeone^1^, L. Puybasset^2^, G. Auzias^3,4^, O. Coulon^3,4^, B. Lesimple^2^, G. Torkomian^2^, L. Velly^1,3^

##### ^1^Aix Marseille University, CHU Timone, Department of Anaesthesiology and Critical Care Medicine, Marseille, France; ^2^CHU Pitié-Salpêtrière, Neurosurgical Intensive Care Unit, Paris, France; ^3^Aix-Marseille University / CNRS, Institut des Neurosciences de la Timone, UMR7289, Marseille, France; ^4^Aix-Marseille University / CNRS, Laboratoire des Sciences de l'Information et des Systèmes, UMR7296, Marseille, France

###### **Correspondence:** P. Simeone – Aix Marseille University, CHU Timone, Department of Anaesthesiology and Critical Care Medicine, Marseille, France

**Introduction**

Traumatic brain injury (TBI) doesn't seem to be a single insult with a monophasic resolution. Recently, degenerative mechanisms have been suggested to occur in the chronic phase and could constituted “tertiary” lesions [1]. These degenerative phenomena can potentially have a worsening impact on the long-term functional prognosis.

**Objectives**

The objective of this prospective study was to longitudinally evaluate (1) white and grey matter structures volumes measured from T1 three-dimensional (3D) and (2) white matter integrity assessed from diffusion tensor imaging (DTI) in severe TBI.

**Method**

20 severe TBI (37 ± 16 yrs) and 12 healthy volunteers (HV; 42 ± 6 yrs) underwent multimodal magnetic resonance imaging in the sub-acute phase (within 21 ± 8 days after injury). A longitudinal follow-up was obtained for all of them at the chronic phase of injury (median 64 ± 16 months after injury) together with neuropsychological assessments. Longitudinal imaging changes were assessed using cortical volumetric reconstruction and segmentation of white and deep grey matter structures with Freesurfer [2]; cortical sulci were automatically reconstructed and identified with Brainvisa software, and a voxel-based DTI analysis was performed with Comasoft. The Extended GOS (GOSE) was used to classify at 5 years the TBI subjects into " good" (GOSE 6-7; *n* = 11) and “intermediate” (GOSE 3-5; *n* = 9) recovery. Cortical morphometry and fractional anisotropy (FA) derived from DTI were used with linear mixed effects models to link changes to behaviour status.

**Results**

At baseline, there were no volumetric differences between the 3 groups (GOSE 3-5; GOSE 6-7; HV). At 5 years, patients with TBI demonstrated a significant volumetric reduction of the whole white matter (-10 ± 4 %; *P* < 0.01), and of the deep grey matter structures (-13 ± 10 %; *P* < 0.03). In contrast, HV did not present any significant change over the same period. Specifically, direct comparisons between patient groups revealed that over time GOSE 3-5 showed greater atrophy than GOSE 6-7 in the parietal lobe (-5 ± 2 *vs*. -3 ± 5 % respectively; *P* < 0.001), brain stem (-12 ± 6 *vs*. -6 ± 7 %; *P* < 0.006), corpus callosum (-19 ± 19 *vs*. -10 ± 15 %; *P* < 0.01), and cingulate (-7 ± 5 *vs*. -2 ± 6 %; *P* < 0.023). This was associated with higher depth mean on sulci data. Furthermore, FA was lower at the first MRI in GOSE 3-5 group in the same regions.Finally, neuropsychological score (Z-score) correlated significantly with the volume loss in these anatomical regions.

**Conclusions**

We observed a strong correlation between neuropsychological scores and morphometric changes over time suggesting (1) occurrence of tertiary lesions and (2) that lesions location influence functional outcome. These data provide further insight into early and late pathophysiology of cognitive dysfunctions after TBI.

**References**

[1] Newcombe *et al*., NNR 2016; 30:49-62

[2] Reuter *et al.*, NeuroImage 2012; 61:1402-18

**Grant acknowledgment**

Fondation "gueules cassées".

### A289 The pharmacokinetics of dexmedetomidine during long-term infusion in critically ill pediatric patients

#### A. Bienert^1^, A. Bartkowska-Sniatkowska^1^, P. Wiczling^2^, O. Szerkus^2^, D. Siluk^2^, J. Bartkowiak-Wieczorek^1^, J. Rosada-Kurasinska^1^, J. Warzybok^1^, A. Borsuk^2^, R. Kaliszan^2^, E. Grzeskowiak^1^

##### ^1^Poznan Unversity of Medical Sciences, Poznan, Poland; ^2^Medical University of Gdansk, Gdansk, Poland

###### **Correspondence:** A. Bienert – Poznan Unversity of Medical Sciences, Poznan, Poland

**Introduction**

Different degree of maturation of various systems and organs in children and neonates might disturb desired therapeutic effects. Therefore, the identification of inter-individual differences affecting pharmacokinetics (PK) of drugs, is important for the dose individualization, especially in children in severe conditions. It especially applies to new drugs such as dexmedetomidine (DEX), for which there is a relatively small number of studies performed. Pediatric Intensive Care Units (PICU).

**Objectives**

The goal of this study was to assess the pharmacokinetics of dexmedetomidine in the PICU settings during the prolonged infusion to assess the influence of routinely collected covariates on underlying PK parameters.

**Method**

Thirty eight patients were enrolled into the study. Continuous intravenous infusion of DEX was initiated at the rate of 0.8 μg/kg/h following by gradual increase or decrease to keep the level of sedation between 7 and 14 points in the Cook Scale. Blood samples for PK assessment (2.0 mL) were collected from the arterial catheter. The analysis was made for the concentrations obtained at two occasions: first from 0 to 24 hr after infusion initiation and second from 0 to 8 hr after infusion end. Data analysis was conducted using WINBUGS software With informative literature priors.

**Results**

The incorporation of time-depended (different between two occasions) PK parameters improved the model. The typical value of the volume of the central compartment (*V*_*1*_) scaled to 70 kg was 52 L, whereas the volume of the peripheral compartment was slightly higher (*V*_*2*_ = 70 L). The typical systemic clearance (*CL*) of DEX and the distribution clearance (*Q*) were 41.6 L/h and 56.8 L/h for a patient with a weight of 70 kg. The IIV estimated for the *CL, Q*, and *V*_*1*_ and *V*_*2*_, were 56 %, 83 %, 152 %, 68 % with a strong correlation (0.7) between *Q* and *V*_*1*_. Those values are consistent with literature parameters in children and adults and are very close to the priors used. It was observed that volume of distribution and clearance is 1.5-fold and 1.3-fold, respectively higher during the second occasion.

**Conclusions**

Population PK model was successfully developed to describe the time course and variability of dexmedetomidine in PICU patients using allometric principles and clearance maturation model. More data is needed to fully confirm clinical significance of the phenomenon of an increase in the volume of distribution and clearance after infusion cessation. The disease status described by PRISM score, the duration of infusion, gender, body weight and age were not found to be independent significant covariates in this study.

**References**

1. Harnisch L, Shepard T, Pons G, Della Pasqua O. (2013) Modeling and simulation as a tool to bridge efficacy and safety data in special populations. CPT Pharmacometrics Syst Pharmacol. 2, e28.

**Grant acknowledgment**

This project was supported by the grant 2015/17/B/NZ7/03032 founded by Polish National Science Centre.

### A290 Early initiation of continuous renal replacement therapies (CRRT) for refractory metabolic acidosis in a cardiothoracic intensive care unit: incidence, timing of CRRT and results

#### C. Hernandez Caballero, S. Roberts, G. Isgro, D. Hall

##### Royal Brompton and Harefield NHS Foundation Trust, London, United Kingdom

###### **Correspondence:** C. Hernandez Caballero – Royal Brompton and Harefield NHS Foundation Trust, London, United Kingdom

**Introduction**

CRRT are being used increasingly in the intensive care unit, not only for renal indications but also other organ-supportive strategies. Although early initiation of RRT is not clearly associated with benefit, avoiding or delaying RRT is associated with higher mortality and increased hospital/ICU lengths of stay

**Objectives**

To describe the use of CRRT for the treatment of non-renal metabolic acidosis in a cardiothoracic intensive care unit.

**Method**

We performed a retrospective observational study including all patients admitted to level 3 areas requiring CRRT for refractory metabolic acidosis at Harefield Hospital during 2015, i.e. pH less than 7.15 and base deficit less than or equal to -8 mmol/L. This population included patients admitted after cardiac and thoracic surgery, heart or lung transplantation, mechanical circulatory devices, out of hospital cardiac arrests (OOHCA) and medical admissions from the cardiology or cardio-thoracic surgical wards. Demographic variables were collected, along with the timing and duration of CRRT.

**Results**

**Fig. 113 (abstract A290).**
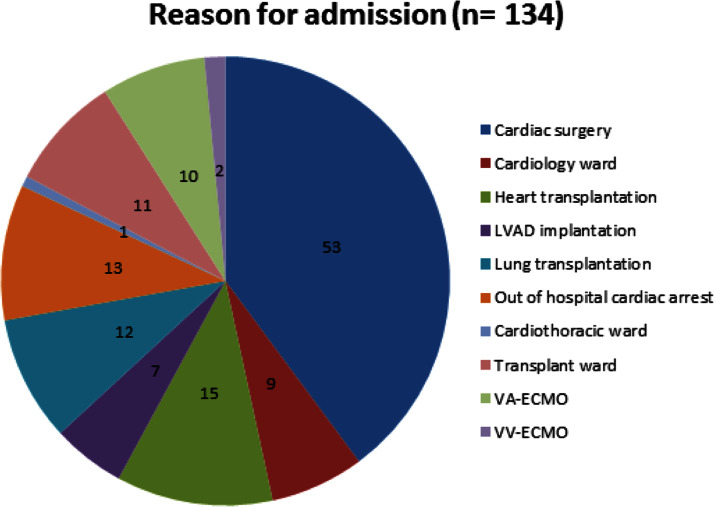
Reason for admission to ITU

Of the 1829 patients admitted to level 3 areas at Harefield Hospital during 2015, 229 (12.5 %), required CRRT during their admission. 134 (58.5 %) of those patients were initiated on CRRT due to refractory metabolic acidosis. The median age of this group was 61 years: 70.1 % were male, 47.8 % hypertensive and 19.4 % diabetic. Most patients (113 patients, 84.3 %) were haemodynamically unstable, defined as the need for one inotropic drug at a high dose or the combination of two or more vasoactive drugs. The reason for admission also differed from the general CRRT group, with a higher frequency of heart transplantations and OOHCA, as shown in Table 87 and Fig. 113. Time from admission to ITU to initiation of CRRT was shorter at 1 day in the refractory metabolic acidosis CRRT group vs. 4 days in the general CRRT group. The ITU mortality in the study group was 42.5 % vs. 36.2 % in the general CRRT group.

**Conclusions**

CRRT for refractory metabolic acidosis in the context of cardiac instability is used in the critically ill patient as an adjunct strategy and is applied earlier than CRRT initiated for other indications.

**References**

1. Karvellas CJ, Farhat MR, Sajjad I, Mogensen SS, Leung AA, Wald R, Bagshaw SM. A comparison of early versus late initiation of renal replacement therapy in critically ill patients with acute kidney injury: a systematic review and meta-analysis. Crit Care. 2011;15:R72

2. Clec'h C, Darmon M, Lautrette A, Chemouni F, Azoulay E, Schwebel C, Dumenil AS, Garrouste-Org, Goldgran-Toledano D, Cohen Y, Timsit JF. Efficacy of renal replacement therapy in critically ill patients: a propensity analysis. Crit Care. 2012;16:R236

**Table 87 (abstract A290).** Groups comparison

	CRRT group (n= 239)	Metabolic acidosis group (n=134)
Median age in years	58.32	60.63
Haemodynamic instability	160 ( 69.95%)	113 (84.8%)
Reason for admission	Cardiac surgery 36.2% Cardiology ward 10% Transplant ward 10% VA-ECMO 10% Lung transplantation 9.2% Heart transplantation 7.9% OOHCA 7.4% LVAD implantation 3.4% Thoracic surgery 1.7% VV-ECMO 1.7% Cardiothoracic ward 1.3%	Cardiac surgery 39.6% Heart transplantation 11.2% OOHCA 9.7% Lung transplantation 8.2% Transplant ward 8.2% VA ECMO 7.5% Cardiology ward 6.7% LVAD implantation 5.2% VV-ECMO 1.7% Cardiothoracic ward 0.7%
Mortality in ITU	83(36.2%)	57 ( 42.5%)
Mean ITU LOS in days	18	17.6
Time to CRRt in days	1	4

### A291 Incidence and characteristics of sudden unexplained cardiac death: insights from the Parisian registry

#### G. Guillaume^1,2,3^, O. Passouant^1^, F. Dumas^2,3,4^, W. Bougouin^1,2,3^, B. Champigneulle^1^, M. Arnaout^1,2^, J. Chelly^5^, J.-D. Chiche^1,2^, O. Varenne^2,6^, J.-P. Mira^1,2^, E. Marijon^2,3,7^, A. Cariou^1,2,3^

##### ^1^Cochin Hospital, Medical Intensive Care Unit, Paris, France; ^2^Paris Descartes University, Paris, France; ^3^Sudden Death Expertise Center, INSERM UMR1018, Paris, France; ^4^Emergency Department, Cochin Hospital, Paris, France; ^5^Intensive Care Unit of Melun Hospital, Melun, France; ^6^Cardiology Department, Cochin Hospital, Paris, France; ^7^Georges Pompidou European Hospital, Cardiology Department, Paris, France

###### **Correspondence:** G. Guillaume – Cochin Hospital, Medical Intensive Care Unit, Paris, France

**Introduction**

Respective proportions of final etiologies are disparate in cohorts of cardiac arrest patients, depending on examined population and diagnostic algorithms. In particular, incidence and characteristics of sudden unexplained death (SUD) are debated. We aimed at describing etiologies in a large cohort of out-of-hospital cardiac arrest (OHCA) patients, in order to characterize victims without identified diagnosis.

**Patients and method**

We analyzed data from our prospective registry of OHCA patients admitted between January 2000 and December 2014. Initial diagnostic strategy included coronary angiography, brain and chest CT scan. This was completed by an extensive diagnostic strategy, encompassing biological and toxicological tests, repeated electrocardiograms and echocardiography, MRI, Holter monitoring and endocavitary explorations. Two independent investigators reviewed each final diagnosis. Baseline characteristics were compared between subgroups of patients with chi-square test and Mann-Whitney test, as appropriate. One-year mortality was compared between subgroups using univariate Kaplan-Meier curves.

**Results**

Over the study period, 1,857 patients were admitted in our unit after a resuscitated OHCA. The event was related to a non-cardiac and a cardiac cause in 526 (30.7 %) and 1,057 (61.8 %) patients, respectively. The main cause of cardiac related OHCA was ischemic heart disease (78,4 %) while non-structural cardiomyopathies accounted for only 2,2 %. No diagnosis was evidenced in 127 (7.4 %) patients. In these SUD patients, baseline characteristics and one-year survival of patients under 35y were similar to those with identified non-structural cardiomyopathy.

Conclusion

We observed that ischemic heart disease was by far the most common cause of cardiac arrest, while non-structural cardiomyopathies accounted for a very low part of diagnosis. Young patients victims of a sudden unexplained death shared similar baseline characteristics and outcome with patients with non-structural cardiomyopathies. Focusing on this subgroup of patients for further investigations and follow-up may help in managing themselves and their relatives.

**Note:** This abstract has been previously published and is available at [1]. It is included here as a complete record of the abstracts from the conference.

**Reference**

1. Passouant O, Geri G, Dumas F, Bougouin W, Champigneulle B, Arnaout M, Chelly JD, Varenne O, Mira JP, Cariou A (2016) Incidence and characteristics of sudden unexplained cardiac death: insights from the Parisian registry. Annals of Intensive Care 6(Suppl 1): S43.

### A292 Lung ultrasound detects more pulmonary complications than chest X-ray in patients after cardiac-surgery: a prospective observational study

#### M. Beerepoot^1^, H.R. Touw^2^, K. Parlevliet^2^, C. Boer^2^, P.W. Elbers^2^, P.R. Tuinman^2^

##### ^1^VU University Medical Center Amsterdam, Intensive Care, Amsterdam, Netherlands; ^2^VU University Medical Center Amsterdam, Amsterdam, Netherlands

###### **Correspondence:** M. Beerepoot – VU University Medical Center Amsterdam, Intensive Care, Amsterdam, Netherlands

**Introduction**

Patients after cardiac surgery are prone to develop postoperative pulmonary complication (PPCs), which are currently diagnosed by chest X-ray (CXR). LUS may provide accurate diagnostic information in patients with acute respiratory distress [1]. The role of LUS in detecting PPCs in patients after cardiac surgery has yet to be determined.

**Objectives**

To compare the rates of PPCs detected by LUS - and CXR in patients after cardiac surgery admitted to ICU. Additionally, we assessed the inter-observer agreement for LUS and compared the time it takes to perform LUS and CXR.

**Method**

This is a prospective observational single centre study. The study was approved by the institutional review board. On admission to the ICU, rates of PPCs detected by LUS according to the BLUE-protocol [2] and by CXR were compared. In addition, accuracy of LUS and CXR for detecting clinical relevant (cr-PPCs), PPCs for which the attending physician, blinded for the LUS data, initiated therapy was evaluated. Cr-PPCs were considered the composite reference standard. 70 blue points were assessed to determine inter-observer agreement. In 15 patients time to perform both techniques was compared.

**Results**

70 % Of the 134 enrolled patients was male with a mean age of 68 ± 9,5. LUS identified 62 PPCs (46 %), compared to 29 (22 %) by CXR (P = 0,011). PPCs identified were: positive Postero-Lateral Alveolar and/or Pleural Syndrome (PLAPS) 40 % vs 13 %, pulmonary edema (5 % vs 9 %) and pneumothorax (1 % vs 0 %). Incidence of cr-PPCs was 16 (12 %). Table 88 summarizes the diagnostic accuracy of LUS and CXR for the cr-PPCs identified. LUS can be performed within 15 ± 5 minutes compared to 42 ± 16 minutes for CXR,(p < 0.01). Overall inter-observer agreement for LUS showed a near to perfect agreement (k = 0.907, p ≤ 0.000).

**Conclusions**

LUS detects more PPCs than CXR in patients after cardiac surgery and with good inter-observer agreement. In addition, time to perform LUS is significantly shorter. However, most PPCs detected by LUS come with little clinical consequence. Therefore, we suggest in further studies to quantify the extent of the PPC detected by LUS to improve its value in clinical decision making.

**References**

1. Touw HR, Tuinman PR, Gelissen HP, Lust E, Elbers PW. Lung ultrasound: routine practice for the next generation of internists. Neth J Med 2015;73(3):100-107

2. Lichtenstein D, van HS, Elbers P, Malbrain ML. Ten good reasons to practice ultrasound in critical care. Anaesthesiol Intensive Ther 2014;46(5):323-335

**Grant acknowledgment**

This study is not supported by any grant.

**Table 88 (abstract A292).** PPC's identified by LUS and CXR related to clinical relevant PPC's for which attending physician initiated therapy

Findings (N=134)	LUS/CXR	SN%	SP%	NPV%	PPV%
PLAPS positive	LUS 53(40%) CXR 17(13%)	100% vs 73%	65% vs 91%	100% vs 97%	19% vs 47%
Pulmonary edema	LUS 7 (5%) CXR 12(9%)	100% vs 75%	97% vs 93%	100% vs 99%	43% vs 25%
Total PPCs detected	LUS 62(64%) CXR 29(22%)	100% vs 81%	62% vs 86%	100% vs 66%	26% vs 45%

### A293 Empirical antibiotic therapy after cardiac arrest

#### Á.J. Roldán Reina, Y. Corcia Palomo, R. Martín Bermúdez, L. Martín Villén, I. Palacios García, J.R. Naranjo Izurieta, J.B. Pérez Bernal, F.J. Jiménez Jiménez, Cardiac Arrest Group HUVR

##### Hospital Universitario Virgen del Rocío, Sevilla, Spain

###### **Correspondence:** Á.J. Roldán Reina – Hospital Universitario Virgen del Rocío, Sevilla, Spain

**Introduction**

Respiratory infection is a common complication in ICU patients admitted after a cardiac arrest. Amoxicillin-clavulanate is used as empirical antibiotic therapy in these patients to prevent aspiration pneumonia. However, recent studies have shown a resistance rate up to 15 %.

**Objectives**

To describe the resistance pattern of microorganisms isolated from bronchial sample of patients admitted to the ICU after a resuscitated cardiac arrest. To assess the appropriate empiric antibiotic therapy.

**Method**

Prospective, observational study from 2013 to 2015. We included all patients admitted in the ICU after a recovered out-of-hospital cardiac arrest and all patients with an in-of-hospital cardiac arrest admitted in the hospital less than 48 hours and without previous administration of antibiotics.

We took samples of tracheobronchial secretions through the endotracheal tube in all patients within the first three days of admission. Demographic information, comorbidities, performing of therapeutic hypothermia, empirical antibiotic therapy administered and the result of bronchial aspirates culture were collected. Empiric antibiotic therapy was considered adequate when at least one effective antibiotic was administered within the first 24 hours of ICU admission.

**Results**

60 patients were included. The average age was 59 and 83,9 % were male. 87,1 % were out-of-hospital cardiac arrests. 50 % of them received therapeutic hypothermia. The most used antibiotic was amoxicillin-clavulanate (54.8 %), followed by piperacillin-tazobactam (6.5 %) and levofloxacin (4.8 %). In 32,3 % of cases no antibiotics were prescribed. Positive microbiological results were obtained in 38.7 % of bronchial aspirates. The most common microorganisms isolated were S. aureus (22,2 %), E. coli (14,8 %), E. cloacae (14,8 %), K. pneumoniae (11.1 %), S. marcescens (11.1 %) and H. Influenzae (7,4 %).

48.1 % of the isolates microorganisms were resistant to amoxicillin-clavulanate, 18.5 % to piperacillin-tazobactam and 14.8 % to third-generation cephalosporin. No organism was resistant to quinolones nor carbapenems.

According to this pattern of sensitivity, 38.1 % of patients with microbiological isolation in the bronchial sample received inappropriate empirical antibiotic therapy.

**Conclusions**

Our results suggest that there is a high resistance rate to amoxicillin-clavulanate of germs isolated in respiratory samples of patients after a resuscitated cardiac arrest.

The most common microorganisms isolated in these samples were S. Aureus, E. coli and E. cloacae, resulting in a high rate of inappropriate empirical antibiotic therapy.

**References**

1. Perbet et al. Early-onset pneumonia after cardiac arrest: characteristics, risk factors and influence on prognosis. *Am J Respir Crit Care Med* 2011

2. Mongardon N et al. Infectious complications in out-of- hospital cardiac arrest patients in the therapeutic hypothermia era. Crit Care Med 2011

**Table 89:** (abstract A293).

Smoking	39(62,9%)
Hypertension	33(50,0%)
Previous isquemic heart disease	13(12,0%)
Microbiologic isolation	24(38,7%)
Empirical Amoxicilin-Clavulanate	34(54,8%)
Empirical Piperacilin-Tazobactam	4(6,5%)
Empirical Quinolones	3(4,8%)
Other empirical antibiotic therapy	1(1,5%)
No antibiotic	20(32,3%)
Inappropriate empirical antibiotic therapy	8(38,1%)

**Table 90 (abstract A293).** Isolations and resistances

S. aureus	6 (22,2%)
E. coli	4 (14,8%)
E. cloacae	4 (14,8%)
K. pneumoniae	3 (11,1%)
S .marscescens	3 (11,1%)
H. influenzae	2 (7,4%)
P. aeruginosa	1 (3,7%)
Amoxicilin-Clavulanate resistance	13 (48,1%)
Piperacilin-Tazobactam resistance	5 (18,5%)
3rd generation cephalosporin resistance	4 (14,8%)

### A294 Hemofiltration veno-venous continouos high and very high volume, hemodynamic answer and mortality in refractory septic shock patients

#### F. Cota-Delgado, M.V. de la Torre-Prados, A. Fernández-Porcel, P. Nuevo-Ortega, E. Cámara-Sola, T. Tsvetanova-Spasova, C. Rueda-Molina, L. Salido-Díaz, A. García-Alcántara

##### University Hospital Virgen de la Victoria / IBIMA, Department of Intensive Care Unit, Málaga, Spain

###### **Correspondence:** F. Cota-Delgado – University Hospital Virgen de la Victoria / IBIMA, Department of Intensive Care Unit, Málaga, Spain

**Introduction**

Refractory septic shock mortality is about 50 %. In the early phase of septic shock, hemofiltration can attenuate the inflammatory cascade, thus alleviating cell and tissue damage and reducing the mortality due to multiple-organ failure syndrome.

**Objectives**

In patients with refractory septic shock Hemofiltration Veno-Venous Continuous High Volume (HFVVCHV, 35 ml / kgr / h) or Hemofiltration Veno-Venous Continuous Very High Volume (HFVVCVHV, 55 ml / kgr / h) decreases vasoactive support and mortality

**Method**

102 refractory septic shock patients, with or without renal dysfunction and consecutively admitted to the ICU, were treated with early resuscitation and support bundles, according Surviving Sepsis Campaign (SSC) 2012, and 36 hours septic shock onset randomly assigned to group 1 (35 ml / kg / h) or group 2 (55 ml / kg / h). The variables studied were hemodynamic parameters (arterial pressure, heart rate, cardiac output, systemic vascular resistance, and central venous pressure), vasoactive drug parameters (dose and time of norepinephrine and hourly fluid intake), pulmonary (paO2/FiO2), and renal function.

**Results**

In both groups the percentage of the initial dose of norepinephrine was reduced 96 hours septic shock onset, higher in group 2 (61 %) than group 1 (54 %).

ICU mortality in the series of patients globally was 19 %, higher percentage in group 1 (23 %) than in group 2 (16 %). Hospital mortality showed an increase of 8 points in group 1 and 10 points in group 2; the global hospital mortality was 29 %.

**Conclusions**

In refractory septic shock patients, after the SSC measures correctly applied in time, an early horizontal treatment with HFVVCHV and HFVVCVHV could improve the prognosis.

**References**

1. Joannes-Boyau O, Honoré PM, Perez P, Bagshaw SM, Grand H, Canivet JL. High-volume versus standard-volume haemofiltration for septic shock patients with acute kidney injury (IVOIRE study): a multicentre randomized controlled trial Intensive Care Med 2013; 39:1535-1546

**Table 91 (abstract A294).** Noradrenaline reduction at 96 h septic shock onset

	Group 1 (35ml/Kg/h) n=52	Group 5 (55ml/Kg/h) n=50	Total n=102
Hemodynamic Disfunction	Media/DS/IC95%	Media/DS/IC95%	Media/DS/IC95%
T-0h: NA mc/Kg/min*	1.42/0.84/1-18-1.65	1.80/0.95/1.53-2.07	1.6/0.91/1.41-1.7
T-96h: NA mcg/kgr/min	0.66/1.10/0.36-0.97	0.72/0.87/0.47-0.96	0.69/0.99/0.49-0.8
%NA Reduction: T-96h and T-0h*	54%/65/36-72	61%/39/50-72	57%/54/42-68

*p* ns, *NA* Noradrenaline

(*) T Student

**Table 92 (abstract A294).** Hospital Mortality

Hospital Mortality	Group 1 (35ml/kg/h) n=52	Group 1 (55 ml/kg/h) n=50	Total n=102
	Número / % Group	Número / % Group	Número / % Group
No	36 / 69%	37 / 74%	73 / 72%
Si	16 / 31%	13 / 26%	29 / 28%
Total	52 / 100%	50 / 100%	102 / 100%

(*) Chi Square, p=ns

## TRAUMA AND EMERGENCY CARE

### A295 Increased serum neutrophil gelatinase-associated lipocalin reflected kidney function and systemic inflammation in emergency department

#### T. Kaneko, H. Tanaka, M. Kamikawa, R. Karashima, S. Iwashita, H. Irie, S. Kasaoka

##### Emergency and General Medicine, Kumamoto University Hospital, Kumamoto, Japan

###### **Correspondence:** T. Kaneko – Emergency and General Medicine, Kumamoto University Hospital, Kumamoto, Japan

**Introduction**

Neutrophil gelatinase-associated lipocalin (NGAL) is used as a biomarker of acute kidney injury (AKI), measured in serum and urine. Recently, serum NGAL has been reported as biomarker of mortality and organ dysfunction in sepsis and post cardiac arrest patients, which is related to systemic inflammation. Therefore, we measured serum NGAL in emergency department patients, and tried to assess which type of pathophysiology was related to increased serum NGAL.

**Objectives**

To assess serum NGAL could be a biomarker of systemic inflammation in emergency department.

**Method**

Forty-four cases admitted emergency department were retrospectively analyzed. Serum NGAL, C-reactive protein (CRP), and white blood cell count (WBC) were compared between 2 groups (estimated glomerular filtration rate (eGFR) >40 mL/min/1.73 m^2^ or not). Correlation analysis was also performed between serum NGAL and others.

**Results**

Median age was 64 y.o., 61 % of patients were male. Median value of serum NGAL was 83 ng/mL. The value of serum NGAL was significant difference between the groups of eGFR > 40 ng/mL and eGFR < 40 ng/mL (80 ng/mL v.s. 163 ng/mL, P = 0.045). Serum NGAL and CRP were significantly correlated in the group of eGFR > 40 ng/mL (R = 0.650, P < 0.001).

**Conclusions**

Serum NGAL might reflects systemic inflammation with patient in emergency department.

**References**

1. Wang B, Chen G, Zhang J, et al. Increased neutrophil gelatinase-associated lipocalin is associated with mortality and multiple organ dysfunction syndrome in severe sepsis and septic shock. Shock 2015;44:234-8.

**Grant acknowledgment**

None.

### A296 Noble gas xenon lessens myocardial injury after out-of-hospital cardiac arrest

#### O. Arola^1^, R. Laitio^1^, A. Saraste^2^, J. Airaksinen^2^, M. Pietilä^2^, M. Hynninen^3^, J. Wennervirta^3^, M. Bäcklund^3^, E. Ylikoski^3^, P. Silvasti^3^, E. Nukarinen^3^, J. Grönlund^1^, V.-P. Harjola^4^, J. Niiranen^5^, K. Korpi^5^, M. Varpula^5^, R.O. Roine^6^, T. Laitio^1^, for the Xe-HYPOTHECA study group

##### ^1^Turku University, Turku University Hospital, Division of Perioperative Services, Intensive Care Medicine and Pain Management, Turku, Finland; ^2^Turku University, Turku University Hospital, Heart Center, Turku, Finland; ^3^Helsinki University, Helsinki University Hospital, Division of Intensive Care Medicine, Department of Anaesthesiology, Intensive Care and Pain Medicine, Helsinki, Finland; ^4^Helsinki University, Helsinki University Hospital, Department of Emergency Medicine and Services, Helsinki, Finland; ^5^Helsinki University, Helsinki University Hospital, Department of Cardiology, Helsinki, Finland; ^6^Turku University, Turku University Hospital, Division of Clinical Neurosciences, Turku, Finland

###### **Correspondence:** O. Arola – Turku University, Turku University Hospital, Division of Perioperative Services, Intensive Care Medicine and Pain Management, Turku, Finland

**Introduction**

In comatose cardiac arrest survivors, the extent of myocardial damage and cardiovascular instability have an important role in the course of developing post cardiac arrest syndrome and predicting long-term outcome. Recent clinical studies have revealed that inhaled xenon provides beneficial cardiovascular effect and mitigate ischemic brain injury in out-of-hospital cardiac arrest (OHCA) patients [1,2].

**Objectives**

The purpose of this study was to assess the effect of xenon inhalation on myocardial ischemic damage and left ventricular function after OHCA.

**Method**

A total of 110 comatose patients who had experienced OHCA were randomized to receive either inhaled xenon combined with hypothermia (33 °C) for 24 hours (n = 55 in the xenon group) or hypothermia treatment alone (n = 55 in the control group). Whenever indicated, coronary angiography and percutaneous interventions were performed before intensive care unit admission or later during hospital stay. Xenon was administered with at least 40 % end-tidal concentration and completed at start of rewarming. Troponin-T (TnT) was measured at hospital admission, and at 24 h, 48 h and 72 h post cardiac arrest. Left ventricular function was assessed with echocardiography by cardiologist at intensive care arrival and at 24 hours after completing rewarming.

**Results**

Among the 110 patients comprehensive TnT measurements were available from 51 xenon patients (median age 63) and 53 control patients (median age 60). Complete echocardiographic data was available on 18 xenon and 20 control patients. The number of ST-elevation myocardial infarction and primary coronary intervention, time for return of spontaneous circulation, cardiovascular medication among other baseline characteristics did not differ significantly between the groups. A median (interquartile range, IQR) post-arrival incremental change in TnT at 72 hours was 0.05 μg/l (-0.03 μg/l -0.61 μg/l) in the xenon group and 0.28 μg/l (0.04 μg/l -1.48 μg/l) in the control group (P = 0.014 for the difference between the groups). A mean (95 % confidence interval) absolute post-arrival incremental change in left ventricular ejection fraction at 24 hours after rewarming was 10.4 % (5.6 %-15.3 %) in the xenon group and 4.3 % (1.4 %-7.2 %) in the control group (P = 0.028 for the difference between the groups).

**Conclusions**

Inhaled xenon in combination with mild therapeutic hypothermia may protect against ischemic myocardial injury as demonstrated by significantly attenuated short-term TnT release and better left ventricular function when compared with hypothermia treatment alone. These observations suggest that inhaled xenon may provide cardioprotective effect in OHCA patients.

**References**

1. Arola O et al. Crit Care Med 2013;41:2116-24;

2. Laitio R et al. JAMA 2016;315:1120-28

**Grant acknowledgment**

This study was funded by Academy of Finland and the Clinical Research Funding (EVO) of Hospital District of South-West Finland.

### A297 NT-proBNP levels and echocardiography findings in the etiologic diagnosis of acute dyspnea

#### S. Salah, B.G. Hassen, A. Mohamed Fehmi

##### Regional Hospital of Zaghouan, Zaghouan, Tunisia

###### **Correspondence:** S. Salah – Regional Hospital of Zaghouan, Zaghouan, Tunisia

**Introduction**

Both NT- proBNP and Doppler echocardiography have been approved in the diagnosis of heart failure. In our Study, we compared the contribution of the NT-proBNP levels with the Doppler echocardiography findings in the diagnosis of decompensated congestive left-heart failure (CHF) in patients with acute dyspnea.

**Patients and method**

It was a prospective, observation al study at the teaching department of emergency and intensive care in the regional hospital of Zaghouan, including patients with severe dyspnea over six months. All patients underwent physical examination, 12-lead ECG, RX Thorax, NT-ProBNP essay and echocardiography by an attending cardiologist on admission. The accuracy of the two Method for etiologic diagnosis was compared on the basis of the final diagnoses established by the medical staff.

**Results**

65 patients were enrolled, including 45 (69 %) with CHF. Diagnosis of CHF was due to coronary artery disease, hypertension, valve disease, arrhythmia and dilated cardiomyopathy. Non-CHF was due to decompensated chronic obstructive pulmonary disease, pneumonia, and severe asthma. Fifteen patients (23 %) were misdiagnosed at admission. The mean NT-proBNP concentration was 8989 [769 to 18945] pg/ml in the CHF subgroup and 462 [22 to 1589] pg/ml in the other patients (p < 0.01). Systolic LV dysfunction (LVEF < 0.45) was found in 31 patients with CHF (60 %) and in 7 patients with other causes of dyspnea (15 %) (p < 0.01). The E/A ratio and the deceleration time of E-wave (DT) were respectively 1,85 ± 0,77 and 12o ± 23 in the group CHF; 0,81 ± 0,44 and 208 ± 47 in the group Non-CHF (p < 0.01). Impaired relaxation and Restrictive mitral pattern were observed respectively in 28 % and 30 % of the patients with CHF and in only two patients and tree patients in the other group.

**Conclusion**

Both NT-proBNP assay and echocardiography can be used for the diagnosis of CHF in acutely dyspneic patients. However, the echocardiography is more accurate in patients with intermediate BNP levels.

**Note:** This abstract has been previously published and is available at [1]. It is included here as a complete record of the abstracts from the conference.

**References**

1. Snouda S, Ben Ghezala H, Abbes MF, Daoudi R, Kaddour M, Benchiekh I (2016). NT-proBNP levels and echocardiography findings in the etiologic diagnosis of acute dyspnea. Annals of Intensive Care 6(Suppl 1): P221.

### A298 Role of in-house trauma surgeon in the initial resuscitation of severe major trauma patients in the South Korea

#### S. Kim

##### Catholic University of Korea, Seoul, Republic of Korea

**Introduction**

The predictive mortality of major trauma patients in the South Korea is 35-40 %. High mortality late of major trauma in the South Korea is due to underdevelopment of prehospital management and transport system, hospital care system, and insufficient supporting system of government policy. Establishment and operation of level I trauma center and management of major trauma patients in the South Korea is at an early stage. In house trauma surgeon´s role in the initial resuscitation of patient with major trauma is very important.

**Objectives**

This study will evaluate the effect of in house trauma surgeon (IHTS) on time for decision making about major procedure (TD), time for hospitalization (TH) and time to operation (TO) in patients with major trauma.

**Method**

This is a retrospective cohort study using trauma database in Uijeonbu St. Mary´s hospital. According to the hospital system, IHTS takes on trauma patients at emergency room for three days on a week. On the other four days, trauma patients were managed by on-call surgeon. Between January, 2013 and December, 2013, 372 of major trauma patients were consecutively enrolled in the study. TD, TH, TO, trauma team activation (TTA). Data were analyzed with presence of IHTS.

**Results**

In patients who were admitted to the department of trauma surgery of general surgery, TD and TH with IHTS took significantly less than those without IHTS (TD: 137 minutes vs. 283 minutes, p = 0.002; TH:302 minutes vs. 635 minutes, p < 0.001). Also TO with IHTS took shorter than that without IHTS (200 minutes vs. 256 minutes), although it shows no statistical significance (p = 0.202). In patients who were admitted to the department of orthopedic surgery, neurosurgery, thoracic surgery or plastic surgery, TD and TH with IHTS took 173,331 minutes. They were lower than those without IHTS (289,642 minutes) and showed statistical significance (p = 0.001, 0.001), Likewise TO with IHTS took shorter than that without IHTS (238 minutes vs. 283 minutes) and showed no statistical difference (p = 0.497). In patients with TTA, TD and TH took statistically less time than those without TTA (TD: 168 minutes vs. 320 minutes, p < 0.001; TH: 329 minutes vs. 773 minutes, P < 0.001). However, TO showed no statistical difference patient with an without TTA (2016 minutes vs. 271 minutes, p = 0.200)

**Conclusions**

IHTS proved it important role by deducing TD and TH in initial trauma resuscitation.

**References**

1. Luchette F, Kelly B, Davis K, Johanningman J, et al. Impact of the in-house trauma surgeon on initial patient care, outcome, and cost. J Trauma. 1997 Mar;42(3):490-5.

2. Helling TS, Nelson PW, Shook JW, Lainhart K, Kintigh D. The presence of in-house attending trauma surgeons does not improve management or outcome of critically injured patients.J Trauma. 2003 Jul;55(1):20-5.

### A299 Early tapering down of resuscitation fluids shortens the periods of mechanical ventilation in major burn patients

#### Y.-C. Hsu

##### National Taiwan University Hospital, Anesthesiology, Taipei, Taiwan, Province of China

**Introduction**

Fluid resuscitation is a crucial component of initial resuscitation in major burn patients to avoid early mortality. Large amount of intravenous fluids are given during this period of time no matter Parkland formula or other formulae are used as the fluid resuscitation guidelines. Furthermore, it is a trend to administrate even larger amount of intravenous fluids in these years, which is known as “fluid creep” phenomenon [1]. However, excessive intravenous fluids would produce generalized and lung edema, which may preclude wound healing and prolong intubation. Besides, no guideline tells us how to adjust intravenous fluids after the first 48 hours.

**Objectives**

We investigate the impact of total intravenous fluids given in the first week to prognosis.

**Method**

This is a retrospective cohort study. Patients who injured in Taiwan Formosa Fun Coast explosion event and admitted to the intensive care units (ICUs) in our hospital in 7 days were included. Group A were patients who admitted to the ICU where a more restrictive fluid management Method was conducted; Group B were patients who admitted to other ICUs where more liberal fluid management approach was applied. We compare the outcome such as sepsis insults, organ dysfunctions, intubation days, ICU days, and hospital days between these 2 groups of patient of different fluid management strategies in major burn patients.

**Results**

18 patients were included. The mean age and total body surface area of burn (TBSA burn) were 21.0 ± 3.0 years old and 56.2 ± 15.0 %, respectively [Table 93].

The total intravenous fluids in the first week in Group A patients (57,363.1 ml) were much less than in Group B patients (68,437.9 ml), and the daily fluids of Group A patients were significantly less at day 4 and day 5 [Table 94] comparing to Group B patients.

**Fig. 114 (abstract A299).**
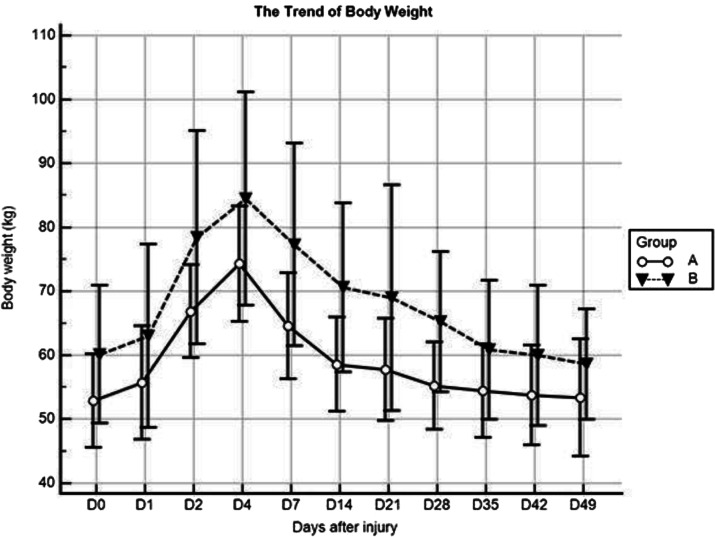
The trend of body weight

Group A patients also had less body weight gain than Group B patients [Fig. 114].

As the results, Group A patients had significantly less days of mechanical ventilation than Group B patients, while length of ICU stay, length of hospitalization, sepsis insults, and organ dysfunctions were all similar in 2 groups [Table 95].

**Conclusions**

We concluded that a more restrictive fluid resuscitation strategy might shorten the period of mechanical ventilation in major burn patients. The aggressive resuscitation fluids should be tapered down quickly after 72 hours.

**References**

1. Saffle JI: The phenomenon of "fluid creep" in acute burn resuscitation. J Burn Care Res 2007, 28(3):382-395

**Grant acknowledgment**

The emergent burn team in Taiwan Fun Coast explosion event, 3A1, 3A2, and 4FI ICUs in National Taiwan University Hospital

**Table 93 (abstract A299).** Patient demographic data

	Group A	Group B	p-value
Patients	10	8	
Gender (male/female)	2/8	5/3	0.145
Age (years old)	21.2±2.6	20.7±3.7	0.756
TBSA (%)	55.4±9.4	57.1±20.6	0.832
Inhalation injury	6	5	1.000
Intubation	7	5	1.000
Body Weight (kg)	52.9±7.3	60.1±10.7	0.109

**Table 94 (abstract A299).** Daily intravenous fluids in the 1st week

Day	Group A	Group B	p-value
1	12501±4509	15069±5647	0.369
2	13183±3446	13437±2904	0.890
3	10074±2338	10720±4340	0.702
4	5870±1518	8702±2288	0.010*
5	5465±1017	9013±2765	0.023*
6	5049±834	6260±2235	0.214
7	5521±863	5237±1328	0.975

**Table 95 (abstract A299).** Patient Outcomes

	Group A	Group B	p-value
Days with mechanical ventilation (all patients)	8.7±9.0	18.4±16.2	0.127
Days with mechanical ventilation (patients with mechanical ventilation)	12.4±8.2	29.4±7.5	0.004*
Length of ICU stay	42.7±12.5	42.9±15.9	0.979
Length of hospitalization	82.7±36.5	82.4±28.8	0.984
ARDS	3/10	4/8	0.630
Sepsis	7/10	6/8	1.000
Acute kidney injury	1/10	1/8	1.000
Mortality	0/10	0/8	

### A300 Time to computer tomography for trauma patients: uncentric prospective study

#### J. Barea-Mendoza^1^, C. García-Fuentes^1^, M. Castillo-Jaramillo^1^, H. Dominguez-Aguado^1^, R. Viejo-Moreno^1^, L. Terceros-Almanza^1^, S. Bermejo Aznárez^1^, C. Mudarra-Reche^1^, W. Xu^2^, M. Chico-Fernández^1^, J.C. Montejo-González^1^

##### ^1^Hospital 12 de Octubre, Trauma ICU, Critical Care Department, Madrid, Spain; ^2^Maryland, Bethesda, United States

###### **Correspondence:** J. Barea-Mendoza – Hospital 12 de Octubre, Trauma ICU, Critical Care Department, Madrid, Spain

**Introduction**

Time is critical in trauma patient care. In other critical diseases (sepsis or IM), decreasing time to definitive treatment have improved patient outcome. Computed Tomography (CT) help us to detect life-threatening lesions. The time until definitive treatment is highly related with CT acquisition times. Due to variations in current literature, there is no consistent arrival to CT times established.

**Objectives**

To describe the time related with Computer Tomography for trauma patients as well as determining associated factors.

**Method**

The design was a prospective and unicentric with a previous protocol. The inclusion criteria were trauma patients accepted in ICU from February to December 2015. Exclusion criteria were previous admission in other hospital. We collected information about demographic characteristics, severity trauma scores and treatment in the Trauma Room (TR). A form was used to collect the time-points (in minutes). Five time-period were defined: T1 from admission (TR) until the start of transferring to CT; T2 from the start of transferring until first image; T3 between the first and the last CT image; T4 from admission (TR) until first oral report by radiologist, T5 from admission (TR) until definitive destination ( ICU, operating room or interventional radiology). Continuous data are presented as medians (interquartile range; IQR) and categorical data in percents. Analyses were performed with Wilcoxon or Fisher´s test depending on data. We perform a multivariate logistic analysis to asses the factors implicated in the times. Using STATA 12, we calculated 2 tailed P-values setting significance at 0.05.

**Results**

113 patients were admitted during study period with a median age of 39 years; 82.2 % were men. The injury severity score (ISS) was > 16 in 53.9 % of patients and urgent surgery was required in 38 % of them. The most frequent mechanism of injury was precipitation (23.3 %). For the described time periods in minutes (T1-T5) median were T1: 30 (25-41), T2: 8 (6-11), T3: 15 (9-20), T4: 50 (30-65), T5: 65 (52-87). Total body strategy was achieved in 78.9 %. In this subgroup, times were longer at T3 (15 vs 9 ) and at T5 (65 vs 50 ) ; p < 0.05.

In the multivariate analysis the factors included were: sex, age, GCS and the related with the resuscitation. At T1 the factors associated with an increased time to definitive treatment were shock and thoracic drainage in the TR (p < 0.05). For T5 the factors were shock , thoracic drainage and whole body strategy (p < 0.05).

**Conclusions**

The CT acquisition times in our center were similar to other centers. Prolonged times were associated with shock patients who needed more aggressive therapies. Awareness about time-wasting activities in trauma patients could help us to detect correctable delays.

**References**

1. Huber-Wagner S et al. Effect of the localisation of the CT scanner during trauma resuscitation on survival. Injury . 2014 Oct.

**Fig. 115 (abstract A300).**
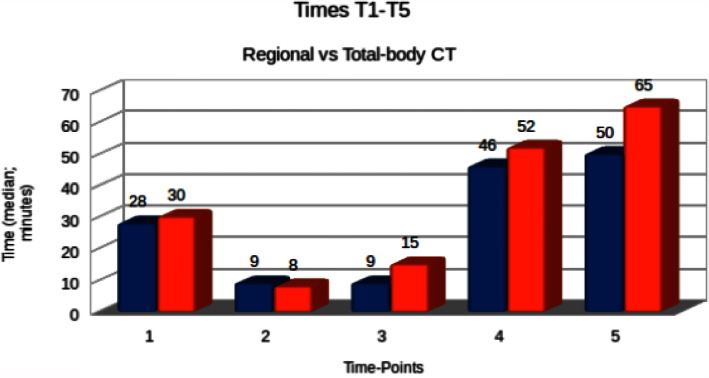
Times vs CT strategy

### A301 Apnoeic preoxygenation for emergency rapid sequence induction of anaesthesia of critically ill patients

#### K. Crewdson, M. Thomas, M. Merghani, L. Fenner, P. Morgan, D. Lockey

##### North Bristol NHS Trust, Anaesthetics and Intensive Care Medicine, Bristol, United Kingdom

###### **Correspondence:** K. Crewdson – North Bristol NHS Trust, Anaesthetics and Intensive Care Medicine, Bristol, United Kingdom

**Introduction** Tracheal intubation is associated with significant complications including death and neurological injury. Patients who require rapid sequence induction (RSI) for emergency tracheal intubation are often critically ill or injured with little physiological reserve. Desaturation is a common problem and studies have demonstrated severe hypoxaemia (arterial oxygen saturation, SaO_2_ < 80 %), in up to 26 % of patients during emergency RSI performed on Intensive Care Units (ICU). Episodes of hypoxaemia during RSI are associated with worse morbidity and increased mortality.

Preoxygenation is intended to reduce desaturation and hypoxaemia in the drug-induced apnoeic phase of RSI. Application of 100 % oxygen via nasal prongs promotes movement of oxygen-enriched air into the alveoli during apnoea, which can help sustain SaO_2_. The majority of studies, though of variable methodology suggest a benefit with apnoeic oxygenation with improvement in SaO_2_ during the apnoeic phase or difficult laryngoscopy. One RCT comparing apnoeic oxygenation to standard facemask oxygen prior to laryngoscopy failed to show any benefit of apnoeic oxygenation.

**Objectives** This is a clinical effectiveness study of apnoeic oxygenation as a preoxygenation strategy to reduce episodes of hypoxaemia for patients undergoing emergency RSI. The primary endpoint was the number of patients who desaturate to less than 90 % in the peri-RSI period.

**Methods** Patients undergoing emergency RSI in the Emergency Department (ED) and ICU at Southmead Hospital, Bristol were included in the study. Southmead Hospital is the regional major trauma, neuroscience and vascular centre and has an additional large unselected medical and surgical take. Data collected included patient demographics, observations before, during and after RSI, and indication for RSI. Data from current standard practice (preoxygenation via a face-mask) was collected over a 3-month period to establish current rates of hypoxaemia. The same data were collected for a subsequent 3 months using apneic oxygenation.

**Results** In total, 71 patients underwent emergency RSI in the study period. Common reasons for intubation were clinical course (39 %), failure to oxygenate (34 %) and failure to ventilate (14 %). Twenty-one complications were experienced during RSI in 20 patients; hypoxia was the most common complication, occurring in 23 % of patients. In the study period, 24 patients received apnoeic oxygenation. Nine of 47 patients who did not receive apnoeic oxygenation experienced hypoxia (19 %), compared with 7 of 24 patients (24 %) did, p = 0.377.

Summary: The incidence of hypoxia associated with emergency RSI in unselected patients is high. The use of apnoeic oxygenation to reduce hypoxia during the drug-induced apnoeic phase of RSI remains a much debated intervention. Having established feasibility, we now intend to investigate the utility of apnoeic oxygenation with larger patient numbers.

### A302 Performance of new generation transport ventilators in simulated critical care conditions

#### E.J. van Lieshout^1^, B. Oomen^1^, J.M. Binnekade^1^, D.A. Dongelmans^1^, R.J. de Haan^2^, N.P. Juffermans^1^, M.B. Vroom^1^

##### ^1^Academic Medical Center, University of Amsterdam, Intensive Care, Amsterdam, Netherlands; ^2^Academic Medical Center, University of Amsterdam, Clinical Research Unit, Amsterdam, Netherlands

###### **Correspondence:** E.J. van Lieshout – Academic Medical Center, University of Amsterdam, Intensive Care, Amsterdam, Netherlands

**Introduction** Given the increase in intra- and inter-hospital transport of critically ill patients it is of vital importance that transport ventilators perform at the same level as ICU ventilators, despite their compact design and challenging conditions during transport ^1^.

**Objectives** To determine accuracy of tidal volume (V_T_), plateau pressure (Pplat) and positive expiratory pressure (PEEP) delivery by gas-driven and turbine equipped transports ventilators under different simulated pulmonary conditions, ventilator settings and oxygen supply modes.

**Methods** Six transport ventilators (gas-driven Hamilton Raphael, Oxylog 3000, Medumat Transport and turbine-equipped Hamilton C1, C2, Elisée 350) and two ICU ventilators (Servo-I , Hamilton G5) were tested under pulmonary conditions simulating healthy lungs, Acute Respiratory Distress Syndrome (ARDS) and Chronic Obstructive Pulmonary Disease (COPD). Accuracy of V_**T**_, Pplat and PEEP were measured by a calibrated pneumotachograph. A percentage difference between actual and displayed values of more than ±10 % was defined as inaccurate.

**Results** Inaccuracy in V_**T**_ delivery was demonstrated in gas-driven transport ventilators Medumat Transport 66010 (in 8 of 10 experiments), Oxylog 3000 (in 7 of 10 experiments), Hamilton Raphael 350 (in 4 of 10 experiments) as well in the turbine-equipped ventilator Elisée 350 (in 6 of 10 experiments). Inaccuracy in PEEP was present mainly in Medumat Transport 66010 (in 5 of 10 experiments) and turbine-equipped Hamilton C2 (in 4 of 10 experiments). No Pplat inaccuracies were detected in any ventilator. Pulmonary conditions as ARDS or COPD or ventilation settings with high PEEP and respiratory rate did not consistently influence inaccuracy in V_T_. The influence of delivery of oxygen from a cylinder on V_T_ inaccuracy was present only in gas-driven ventilators Oxylog 3000 and Medumat Transport 66010 under ARDS-conditions.

**Conclusions** Transport ventilators differ in accuracy of delivering tidal volume demonstrating better performance in turbine equipped models. Oxygen supply by cylinder was of limited influence in two gas-driven ventilators only. Two of three turbine equipped transport ventilators (Hamilton C1 & C2) tested showed accuracy comparable to ICU ventilators and therefore are suitable for critical care transport. The use of many gas-driven ventilators in critical care transport should be questioned considering their inaccurate performance.

**References**

1. Boussen S, Gainnier M, Michelet P. Evaluation of ventilators used during transport of critically ill patients: a bench study. *Respiratory care.* 2013;58(11):1911-1922.

### A303 Clinical warning capability prior to in-hospital cardiac arrest

#### R. Algarte^1^, L. Martínez^1^, B. Sánchez^1^, I. Romero^2^, F. Martínez^1^, S. Quintana^1^, J. Trenado^1^

##### ^1^Hospital Universitari Mutua Terrassa, Critical Care Department, Terrassa, Spain; ^2^Hospital Universitari Mutua Terrassa, Terrassa, Spain

###### **Correspondence:** L. Martínez – Hospital Universitari Mutua Terrassa, Critical Care Department, Terrassa, Spain

**Fig. 116 (abstract A303).**
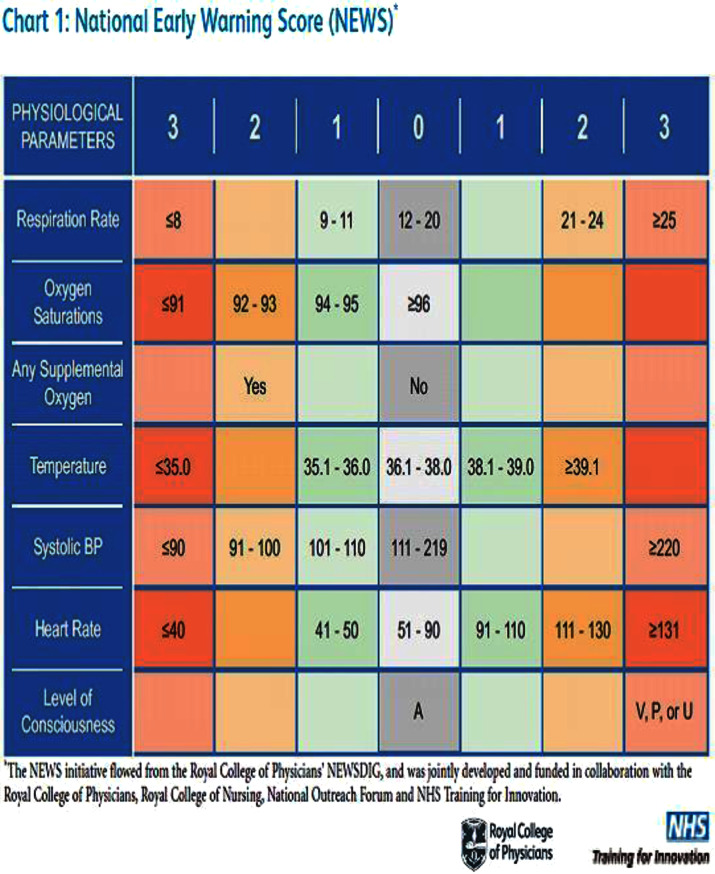
National Early Warning Score (NEWS)

**Introduction** Early detection of clinical response is determinants of clinical outcome in people with acute illness. IHCA (intrahospital cardiac arrest) often is preceded by a clinical deterioration that could be identified and treated by trained personnel. The NEWS (National Early Warning Score) from NHS (National Health System)^1^, is based on a simple scoring system in which a score is allocated to six simple vital signs parameters (respiratory rate, oxygen saturations, temperature, systolic blood pressure, pulse rate, level of consciousness. Figure 116

**Objectives** To describe the patient clinical state prior to IHCA and the relationship with prognosis, in a University Hospital.

**Methods** A retrospective, single-center and descriptive study was conducted during 2014 and 2015. We analyzed all patients admitted on hospital ward that were assisted by the IHCA team. Patients admitted less than 24 hours on Ward and patients not eligible for resuscitation were excluded. Demographic data (age and gender) were collected. We described the type of patient (medical or surgical), features of IHCA attention. The NEWS has 3 levels of scoring, low (1-4), medium (5 and 6) and high (>6) that are related to clinical risk. It was performed by evaluating the data from the vital signs registered on the Ward in the 24 hours prior IHCA.

Statistics.Qualitative variables are expressed as percentages and compared using the X2-test; quantitative ones are expressed as means and standard deviations (± S.D), and analyzed using Student´s t-test. The level of significance was placed at p < 0.05. The statistical analysis was performed using specific software (IBM SPSS Statistics for Windows, Version 19.0. Armonk, NY: IBM Corp).

**Fig. 117 (abstract A303).**
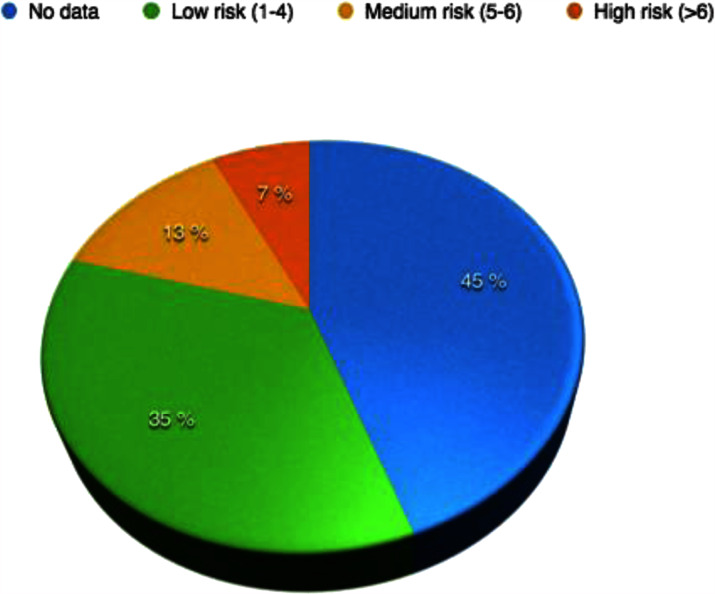
Distribution of NEWS in the total IHCA a

**Results** 85 patients were included. In Table 96 we described the characteristics of the study population. The beginning of cardiopulmonary resuscitation (CPR) maneuvers were immediate on ward, according to IHCA protocol. The arrival of IHCA team was less than 5 minutes in all cases. Figure 117 shows the distribution of NEWS in the IHCA analyzed.

**Fig. 118 (abstract A303).**
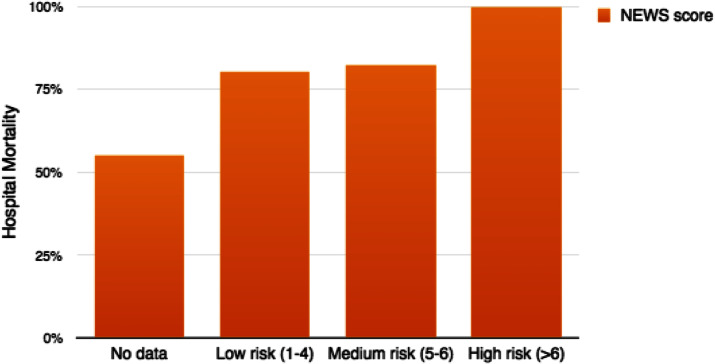
Analysis between hospital mortality

In 43.5 % of cases there are not enough information to make a NEWS. In the remaining patients it was able to perform the NEWS although we could only obtain all data in 6.5 % of patients, in the remaining 50 % of IHCA in which was calculated the NEWS some data (vital sings) was missing. Respiratory rate and oxygen saturations were the most frequent missed data. It could be probably underestimating the NEWS performed. Figure 118 shows the relationship between a greater hospital mortality and a higher NEWS.

**Conclusions** Abnormal vital signs are common within 24 hours before IHCA events on hospital wards. A suitable recording vital signs could be useful to alert patients at risk and anticipate in the detection of IHCA.

**References**

1- Royal College of Physicians. *National Early Warning Score (NEWS): Standardising the assessment of acuteillness severity in the NHS*. Report of a working party. London: RCP, 2012.

**Table 96 (abstract A303).** Characteristics of the study population

Characteristics of the study population	All patients (n=85)
Age. Years mean (SD)	75.1 (SD 11.7).
Male %	65.9
Pre-cardiac arrest situation at IHCA team´s arrival %	37.8
ROSC %	41.5
Hospital mortality %	63.5
IHCA witnessed %	60
IHCA in holiday schedule %	61.5
Recording frequency of vital signs at least every 8 hours %	85
Medical patient %	71.8

### A304 Audit of OOHVF post resuscitation care in Portsmouth post TTM

#### O. Sheikh, D. Pogson, R. Clinton, F. Riccio

##### Queen Alexandra Hospital, Critical Care, Portsmouth, United Kingdom

###### **Correspondence:** O. Sheikh – Queen Alexandra Hospital, Critical Care, Portsmouth, United Kingdom

**Introduction** We conducted a retrospective review of all our ICU admissions post cardiac arrest to identify OOHVF survivors and compare our own care in the post TTM era with ILCOR standards of care and unit guidelines.

**Objectives** To analyse the electronic record of all OOHVF patients admitted to our unit between October 2014 and July 2015 and identify our performance in achieving key components of current standard post-resuscitation care having adopted 36C as our target for 30H post ROSC. Unit LOS and outcomes were also collected.

**Methods** We identified all admissions to our unit post cardiac arrest using the ICNARC database. Our electronic record was then analysed to identify all those patients admitted post OOHVF.

Electronic records were used to identify age, sex, unit LOS(d), estimated ROSC duration, cooling method, time to achieve TTM target 36C, max temp recorded 30H post ROSC, presence of rebound hyperthermia >38C within 48H post TTM, incidence of seizures, use of anticonvulsants and NMB drugs, SSEP N20 and EEG, GCS on unit discharge and unit outcome.

**Results** 66 OOHVF survivors were admitted to our ICU in the audit period. Median age was 62.7 (17-85) M 54, F 12. Median unit LOS was 5.7 days. 56 % of admissions were discharged alive from ICU. 25.7 % patients had seizures on EEG and SSEP was used to prognosticate in 19.7 %. 38 % patients required atracurium to prevent shivering during the TTM period. TTM target 36C was achieved within 4 h post ROSC in 87 %. 78 % patients received cooling via the Icycath and the remainder via surface methods. 37 % of patients had rebound hyperthermia over 38C within 48 h post TTM.

One patient was discharged with GCS9. All other survivors had a GCS of 14 or 15 at unit discharge.

**Conclusions** Our unit adopted controlled temperature management to 36C for 30 h post ROSC after publication of the TTM trial. Our admission numbers and demographics have remained unchanged and our adherence to our guideline is good, though only 78 % received IV cooling catheters as stipulated. Our unit survival of 56 % is identical to audits previously performed and is in line with published outcomes. Despite using cooling catheters to control temperature after the TTM period, 37 % of patients suffered rebound hyperthermia in that 48H window. This does not seem to have adversely affected mortality. Use of EEG and SSEP to assist prognostication common on our unit in prolonged coma. The incidence of seizures is common in the post TTM phase.

**References**

Nolan J *et al.* ILCOR consensus statement. Resuscitation (2008) **79;** 350-379

Nielsen *et al.*TTM at 33c vs. 35c after cardiac arrest. NEJM (2013) **369;** 2197-2206

**GRANT ACKNOWLEDGMENT**

The help of Mr M Lympany, IT support in our Dept.

### A305 A review of outcome after out of hospital cardiac arrest admitted to a tertiary centre

#### L. Gemmell^1^, A. MacKay^1^, A. Arthur^2^, L. Young^1^, A. Sinclair^2^

##### ^1^Queen Elizabeth University hospital, Anaesthetics and Intensive Care, Glasgow, United Kingdom; ^2^Golden Jubilee National Hospital, Anaesthetics and Intensive Care, Glasgow, United Kingdom

###### **Correspondence:** L. Gemmell – Queen Elizabeth University hospital, Anaesthetics and Intensive Care, Glasgow, United Kingdom

**Introduction** Sudden cardiac death represents a major health problem. In adults, the prevalence of out of hospital cardiac arrests (OHCA) attended by the emergency medical services is 75 per 100,000. Mortality remains high, and exceeds 90 %. It is well documented that patients with a shockable rhythm have a consistently higher survival than those whose initial cardiac rhythm is non-shockable (1).

**Objectives** The aim of this study was to look at outcome from cardiac arrests admitted to a tertiary referral service and assess whether the presentation of a shockable rhythm was associated with a better prognosis.

**Methods** A retrospective case note review over a one year period of all patients admitted to the Golden Jubilee National hospital with an OHCA with initial rhythm being ventricular tachycardia (VT) or ventricular fibrillation (VF) were included. We looked at cause of cardiac arrest, ICU survival and dependance upon survival, age and length of stay.

**Results** Sixty three patients were identified, and a full data set obtained for 59. Median age of admission was 59 years, with age range from 26 years to 83 years. All presenting rhythms were either VF or VT. 58 % of this cohort of patients survived post cardiac arrest. Median length of stay was 5 days (IQ range 1-14days). Of the 34patients that survived, 32 of these patients went on to live an independant life. Of the 25patients that died, all of these patients died during their admission to Intensive Care and did not die post ICU discharge. Interestingly, patients whose cause of cardiac arrest was purely arrhythmogenic all survived to hospital discharge, although these numbers are small (n = 11).

**Conclusions** Mortality from cardiac arrest is high and places a huge burden on ICU services. The median length of stay for OHCA patients is 5 days, and with patient numbers in this study is attributable to one ICU bed per day. Their length of stay is thought to quantify the difficulties in prognostication of survival in this cohort of patients, particularly when it comes to the secondary brain injury. Although our sample size is small, it is demonstrated that survival after OHCA where a shockable rhythm is the presenting rhythm, is higher than quoted in literature. Interestingly, patients that survive OHCA are likely to lead an independant life post discharge. If the cardiac arrest cause is arrhythmogenic, although the numbers are small, the survival for this cohort is 100 %.

**References**

1. Temple et al. Predicting neurological outcome and survival after cardiac arrest. Anaesthesia 2012.

### A306 Increase of lipopolysaccharide binding protein is associated with reduction of circulating endotoxin with the onset of fever in trauma patients hospitalized in intensive care unit. Preliminary results

#### D. Markopoulou, K. Venetsanou, L. Filippou, E. Salla, S. Stratouli, I. Alamanos

##### KAT Hospital, B ICU and Research Center, Kifisia, Greece

###### **Correspondence:** D. Markopoulou – KAT Hospital, B ICU and Research Center, Kifisia, Greece

The occurrence of septic shock and sepsis in critically ill patients, hospitalized in ICU, continues to be among the most serious complications, despite the new methods of diagnosis and treatment. The aim of the study is to investigate the effect of fever onset on endotoxin markers.

**Materials & methods** Eighteen polytrauma patients admitted in ICU and 18 healthy volunteers enrolled in the study. 10 ml of blood collected from i) each patient within 24 h of admission (A) and the onset of fever >38° (F) and ii) from healthy individuals (H). Clinical and demographic data recorded on admission. Serum/plasma samples were isolated with centrifugation and stored at -70 ° C. Lipopolysacharide binding protein (LBP), measured with ELISA and circulating endotoxin (LAL) with chromatometric assay.

**Results** On admission, the levels of LBP and endotoxin had no significant differences between patients (A) and healthy (H), P > 0.05. The onset of fever (F) was accompanied by abundant significant LBP release (P_FA_ >0.001 and P_FH_ >0.001) parallel reduction of circulating endotoxin (P_FA_ >0.001 and P_FH_ >0.001)

**Conclusions** Increased LBP release at the onset of fever could account for marker of circulating endotoxin and the following inflammatory implications, in trauma patients hospitalized in ICU.

**Table 97 (abstract A306).** Data are presented as Median± IQR, LBP and endotox

	A (n=18)	F (n=18)	H (n=18)	p A~H	p F~H	p F~A
ENDOTOXIN (EU/ml)	0.32(0.257, 0,422)	0,14±0,03	0,3±0,01	NS	<0.001	<0.001
LBP (μg/ml)	4,4 ± 5,8	94,7± 90,6	6,9 ± 9,5	NS	<0.001	<0.001

### A307 The timing of tracheotomy in cervical spinal cord injury patients: a retrospective study

#### A.H. Guirgis

##### Khoula Hospital, ICU, Muscat, Oman

**Introduction** In Intensive Care Unit (ICU) per cutaneous tracheotomy plays a vital role in airway management of patients with Cervical Spinal Cord Injury (CSCI). This retrospective study evaluated the favorable effect of early tracheotomy in patients CSCI.

OBJETICVES. To evaluate the timing of tracheotomy in patients with cervical injury and its effect on ICU stay

**Methods** Retrospective data analysis of 72 patients with CSCI who underwent tracheotomy. The primary objective was to evaluate the impact of early tracheotomy(performed within 1 week of CSCI) on better outcome, in terms of days on mechanical ventilation and ICU stay in patients with high (C1, C2) and low (C3 to C7) CSCI. Outcome measures were also compared between the high and low CSCI patients when early tracheotomy was performed in both. In addition the impact of early tracheotomy in terms of survival benefit, ventilator dependence, requirement of inotropic medications, and whether surgical intervention resulted in better outcome in terms of days in mechanical ventilation and ICU stay were also analysed.

**Results** Patients with high CSCI patients with early tracheotomy had significantly less days on mechanical ventilation and inotropic support compared to those with late tracheostomy (9.3+/-7.2 and 13.7+/-3.2; p = 0.04 and 0.3+/-1.8 and 4.7+/-11.4; p = 0.035 respectively). Low CSCI patients with early tracheotomy also had significantly less days on mechanical ventilation compared to those with late tracheotomy (12.1+/-10.4 and 25.2+/-17.7; p = 0.034). Early tracheotomy resulted in significantly less days of inotropic support in high CSCI patients compared to low CSCI (0.3+/-1.8 and 2.7+/-5.6; p = 0.022). A trend was observed towards lesser ICU stay and days on mechanical ventilation in patients of high CSCI with early tracheotomy. There was no difference in terms of survival benefits, ventilator dependence, and impact of surgery on outcome measures

**Conclusions** Our retrospective analysis supports the hypothesis that early tracheotomy is beneficial in reducing the period on mechanical ventilation and inotropic support in patients with CSCI, irrespective of the anatomical level of injury

**References**

1. Herkowitz HN, Grafin SR, Eismont FJ, Bell GR, Balderston RA. The Spine; Expert Consultant: 6^th^ Edition 2011.

2. Thesleff T, Niskakangas T, Luoto TM, Ohman J, Ronkainen A. Fatal cervical spine inuries: a Finnish nationwide register based epidemiological study on data from 1987 to 2010. Spine J 2015; Epub Dec 7.

3. Tanaka J, Yugue I, Shiba K, Maeyama A, Naito M. A study of risk factors for tracheostomy in patients with a cervical spinal cord injury. Spine (Phila Pa 1976) 2015; Nov 30: [Epub ahead of print]

**Grant acknowledgment**

To all the staff of Khoula ICU who shared me in this article.

### A308 Emergency trauma and surgery patients: negative pressure therapy vs temporary abdominal closure

#### R. Gutiérrez Rodriguez^1^, M.J. Furones Lorente^2^, I. Macias Guarasa^1^

##### ^1^Carlos Haya Hospital, ICU, Malága, Spain; ^2^Carlos Haya Hospital, Málaga, Spain

###### **Correspondence:** M.J. Furones Lorente – Carlos Haya Hospital, Málaga, Spain

**Introduction** Severe abdominal sepsis is one of the more important risk factor for ICU and Hospital Mortality in the abdominal post-operative period.

**Objectives** The objective was to analyze ICU, and Hospital mortality, Days of Mechanical ventilation, kidney failure, pneumonia, adult respiratory distress syndrome, and vasoactive drugs used. Also we study the differences between temporary abdominal closure and negative pressure therapy (ABThera,kCI).

**Methods** We analyze all patients admitted in our ICU from emergency abdominal Trauma and surgery areas, between January 2009 to January 2015 . It was a retrospective study. We compered different variables as age, sex, manheim peritonitis index, CHARLSON INDEX, APACHE II, and SOFA on first day admission ,ICU, Hospital Mortality, pneumonia, days of mechanical ventilation in two groups of patients; patients with primary temporary abdominal closure, and patients with previous negative pressure assistance. Significant statically results when P level was < 0.05.

**Results.** 93 patients were admitted in our ICU between 2009-2015, Mean APACHE II 22.3,Mean SOFA 3,8.

Group 1: patients with primary abdominal closure: Hospital mortality was lower in patients after prymary abdominal closure 32 % vs 37 % ( ns P > 0.05) in patients with previous negative abdominal pressure assistance, longer stay 22.6 days vs 15.6 days in patients without primary abdominal closure ( P ns), less days of mechanical ventilation 8 days vs 11 days in patients without abdominal closure, P > 0.05, pneumonia 12.9 % vs 18 %, ns, nosocomial infections, 36 % vs 58 %, p ns, kidney failure by RIFLE score 11 % vs 21 %, ns.

**Conclusions** In our study the primary abdominal closure showed less ICU and Hospital mortality also less ICU complications as pneumonia or Kidney failure although the differences were not significant statically. Probably we will need it a bigger sample to observe significant differences.

## CARDIOVASCULAR MONITORING 1

### A309 The influence of peep and positioning on central venous pressure and venous hepatic hemodynamics in patients undergoing liver resection

#### A. Ukere^1^, S. Meisner^2^, G. Greiwe^1^, B. Opitz^1^, D. Benten^2^, B. Nashan^3^, L. Fischer^3^, C.J.C. Trepte^1^, D.A. Reuter^1^, S.A. Haas^1^, C.R. Behem^1^

##### ^1^University Medical Center Hamburg-Eppendorf, Anesthesiology, Hamburg, Germany; ^2^University Medical Center Hamburg-Eppendorf, Internal Medicine, Hamburg, Germany; ^3^University Medical Center Hamburg-Eppendorf, Hepatobiliary Surgery and Visceral Transplantation, Hamburg, Germany

###### **Correspondence:** C.R. Behem – University Medical Center Hamburg-Eppendorf, Anesthesiology, Hamburg, Germany

**Introduction** Quantity of blood loss during liver resection is known to be a predictor for poor clinical outcome (1). One generally used approach for minimization of blood loss is to decrease central venous pressure (CVP) (2). The rationale for this concept is that low CVP is supposed to reduce hepatic blood congestion.

**Objectives** In order to assess blood congestion of the liver we aimed to evaluate the influence of a positive-end-expiratory-pressure (PEEP) and positioning of the patient on CVP and venous hepatic blood flow in patients undergoing liver resection. Further, we analyzed correlation between CVP and venous hepatic blood flow parameters.

**Methods** We analyzed 20 patients scheduled for elective liver resection in this study. We measured CVP and quantified venous hepatic hemodynamics by ultrasound assessment of flow velocity and diameter of the right hepatic vein and the portal vein during the following maneuvers: M1: 0° supine position, PEEP 0cmH_2_O; M2: 0° supine position, PEEP 10cmH_2_O; M3: 20° reverse-trendelenburg position; PEEP 10cmH_2_O; M4: 20° reverse-trendelenburg position, PEEP 0cmH_2_O.

**Results** Changing from supine to reverse-trendelenburg position was accompanied by a significant decrease in CVP (M3 5.95 ± 2.06 mmHg vs. M1 7.35 ± 2.18 mmHg, p = 0.031 and M2 8.55 ± 1.79 mmHg, p < 0.01 respectively). The reduction of PEEP in reverse-trendelenburg position further decreased CVP (M4 2.9 ± 2.17 mmHg vs. M1 7.35 ± 2.18 mmHg, p < 0.01, M2 8.55 ± 1.79 mmHg, p < 0.01 and M3 5.95 ± 2.06 mmHg, p < 0.01 respectively). PEEP and positioning induced no significant changes in the diameters of the right hepatic or portal vein. The combination of PEEP 10cmH_2_O and reverse-trendelenburg position led to significant reduction of systolic (Vs_HV_) and diastolic (Vd_HV_) flow velocities of the right hepatic vein (Vs_HV_ M3 19.96 ± 6.47 cm s^-1^ vs. M1 27.81 ± 11.03 cm s^-1^, p < 0,01; Vd_HV_ M3 14.94 ± 6.22 cm s^-1^ vs. M1 20.15 ± 10.34 cm s^-1^, p = 0,01 and M2 20.19 ± 13.19 cm s^-1^, p = 0,021 respectively) whereas no significant changes of flow velocity of the portal vein occurred in any of the maneuvers. No correlations between CVP and diameters or flow velocities of the right hepatic and the portal vein were found.

**Conclusions** Changes of central venous pressure due to changes of PEEP and positioning were not correlated with changes of venous liver blood flow. Therefore, those strategies aiming for low central venous pressure maneuvers in liver resection are not supported by these results.

**References**

1. Katz SC, Shia J, Liau KH, Gonen M, Ruo L, Jarnagin WR, Fong Y, D´Angelica MI, Blumgart LH, Dematteo RP. Operative blood loss independently predicts recurrence and survival after resection of hepatocellular carcinoma. Annals of surgery 2009;249:617-23.

2. J Wang WD, Liang LJ, Huang XQ, Yin XY. Low central venous pressure reduces blood loss in hepatectomy. World journal of gastroenterology: WJG 2006;12:935-9.

### A310 Echocardiographic variables and outcome in cardiac and respiratory patients: the vital role of cardiac electromechanics

#### G. Tavazzi^1,2^, B. Ana^3^, A. Vazir^4^, D. Gibson^4^, S. Price^5^

##### ^1^University of Pavia, Anaesthesia, Intensive Care and Pain Therapy, Pavia, Italy; ^2^Fondazione Policlinico San Matteo, IRCCS, Anaesthesia, Intensive Care and Emergency Department, Pavia, Italy; ^3^Universitat Autònoma de Barcelona, Cardiology Department, Barcelona, Spain; ^4^Royal Brompton and Harefield NHS Foundation Trust, Cardiology Department, London, United Kingdom; ^5^Royal Brompton and Harefield NHS Foundation Trust, London, United Kingdom

###### **Correspondence:** G. Tavazzi – University of Pavia, Anaesthesia, Intensive Care and Pain Therapy, Pavia, Italy

**Introduction** There are few data reporting which hemodynamic variables are associated with outcome^1^ Despite the widespread of echocardiography, indices to assess the cardiac function in ICU are the same used in the cardiology ward although they have not been validated. Systolic longitudinal function (MAPSE) and total isovolumic time (t-IVT), although under recognized, are sensitive and early marker of myocardial perfusion mismatch and, the latter, of cardiac output(CO) and VO2 changes in patients with coronary artery disease and chronic heart failure1.

**Objectives** We sought to determine the correlation between echocardiographic systo-diastolic indices, including t-IVT and MAPSE, and hemodynamic parameters and their outcome value on 30-days mortality in the cardiothoracic ICU.

**Methods** The local ethical committee approved the study. We retrospectively analyzed data for patients (n = 131; age 59 ± 18.3; 61.3 % male) admitted to ICU requiring an echocardiography from January to August 2012.

Patients were divided in 3 groups: 55 patients had severe respiratory failure; 40 patients after cardiac surgery and 36 had primary cardio-circulatory failure.

In addition to demographic and hemodynamic parameters, echocardiographic indices included: MAPSE (M-mode on the four portion of the mitral annulus), ejection fraction(EF), fractional shortening(FS), E/A, E/E', Doppler assessment ejection time (ET-aortic forward flow)& filling time(FT-mitral inflow), t-IVT (calculated as [60-(total ET + total FT)]) and SV&CO derived from aortic VTI.

Continuous variables were compared using the Wilcoxon rank-sum test, categorical variables were compared using the Pearson χ2 test. The significance of differences in mortality between groups was assessed using the log-rank test.

**Results** In univariate analysis a strong inverse correlation was found between t-IVT and MAPSE and SV&CO.

In the univariate logistic analysis, SV&CO, MAPSE and t-IVT were all good predictors of the 30 days outcome. In the multivariate model, along with SAPS II (HR 1.07, IC 95 %1.05-1.098), t-IVT (HR 5.1, IC 95 % 2.2-11,8) and MAPSE (HR 0.17, IC95%0.02 -0.97)showed to be independent predictors of mortality.

EF and FS do not correlate with other echocardiographic and hemodynamic parameters and no relation with outcome was found.

**Conclusions** This is the first study investigating the role of those echocardiographic indices in the ICU cohort. EF, a validated index to stratify patients according to the systolic function, failed to correlate with hemodynamic parameters and outcome. t-IVT, index of systo-diastolic interaction correlating with the consequences, in term of cardiac output, of those variations, and MAPSE resulted the most sensitive parameters reflecting cardiac efficiency and outcome.

**References**

1. Torgersen C.Crit Care. 2009;13(5):R157.

2. Duncan A. JACC Vol.41, No. 1, 2003.

**Table 98 (abstract A310).** t-IVT correlation with echo&hemodynamic indice

	Whole population	Respiratory	Cardiac post-surgery	Cardiac primary
SV	0.006 [0.75-0.94]	<0.0001 [0.85-0.95]	<0.0001 [0.8 - 0.96]	<0.0001 [0.87-0.96]
CO	0.0001 [0.43-0.65]	<0.0001 [0.04-0.38]	<0.0001 [0.04 - 0.62]	<0.0001[0.18- 0.58]
MAPSE lateral	<0.0001 [0.68-0.89]	<0.0001[0.65-0.91]	0.008 [0.03-0-58]	<0.0001[0.25-0.53]
MAPSE septal	<0.0001 [0.45-0.72]	<0.0001[0.68-0.89]	0.01 [0.05-0.56]	<0.0001[0.42-0.82]
E/A	0.13 [0.05-1.47]	0.48 [0.13-2.22]	0.54 [0.12-1.89]	0.015 [-7.5—0.85]
E/E´	0.23 [0.04-1.34]	0.32 [0.12-1.76]	0.57[0.1-1.5]	0.08[-6.5-0.67]

**Table 99 (abstract A310).** Outcome predictor

	Coeff	Std Err	t	P	CI 95%
MAPSE	-23.29	5.44	-4.28	<0.0001	-34.08 - -12.52
SV	0.16	0.41	4.06	<0.0001	0.08 -0.25
CO	-1.39	0.4	-3.47	0.001	-2.2 - -0.59
SAPSII	0.16	0.02	5.65	<0.001	0.1 - 0.21

**Fig. 119 (abstract A310).**
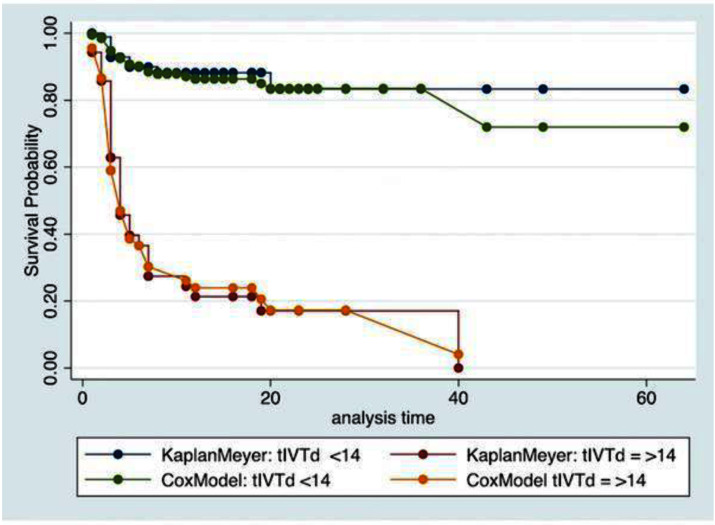
t-IVT outcome

### A311 The effect of passive leg raising maneuver on right internal jugular vein diameter in icu patients under mechanical ventilation

#### M. Masjedi^1,2^, M.R. Hadavi^1^, M. Riahi alam^3^, M.R. Sasani^4^

##### ^1^Shiraz University of Medical Sciences, Anesthesia and Intensive Care Department, Shiraz, Islamic Republic of Iran; ^2^Anesthesiology and Critical Care Research Center, Anesthesia and Intensive Care Department, Shiraz, Islamic Republic of Iran; ^3^Shiraz University of Medical Sciences, Shiraz, Islamic Republic of Iran; ^4^Shiraz University of Medical Sciences, Radiology, Shiraz, Islamic Republic of Iran

###### **Correspondence:** M. Masjedi – Shiraz University of Medical Sciences, Anesthesia and Intensive Care Department, Shiraz, Islamic Republic of Iran

**Introduction** To improve accessibility, central vein catheterization in upper body region classically done in trendelenburg position but it may impose potential disadvantages to respiratory system and disturb physiologic status of many other organs. Passive leg raising (PLR), a simple maneuver , widely used to improve cardiac preload and to predict patients' volume responsiveness could be an alternative.

**Objectives** In this study, we evaluated the effect of PLR maneuver on right internal jugular vein (RIJV) diameter in intensive care unit patients under mechanical ventilation.

**Methods** As a prospective study , twenty patients under synchronized intermittent mandatory ventilation (SIMV) without valvular heart problem or heart failure and acute respiratory distress syndrome were studied. RIJV diameter was measured with bedside color Doppler sonography of neck , first in supine position and then for second and third measurement, after 30° PLR for 1 and 10 minutes. Measurements were at the end of inspiratory cycle with positive end expiratory pressure of 5. We chose 30° PLR to keep bedridden patients away from possible damage that may be induced with higher upward slope.

**Results** RIJV diameter increased with 30° PLR maneuver , more prominent after 1 minute in comparison to 10^th^ minute time point . Mean RIJV diameter was 11.66 mm in supine, 13.37 mm (P = 0.001) and 12.95 mm (P = 0.005), 1 and 10 minutes after 30° PLR maneuver respectively . Increments in diameter were slightly lower than that associated with trendelenburg position reported in other studies . No complication was noted.

**Conclusions** PLR maneuver can be safely considered as an alternative to trendelenberg position to increase internal jugular vein diameter in mechanically ventilated patients.

**References**

1- Monnet x , Teboul JL. Passive leg raising , Intensive care med. 2008 April;34(4):659-63.

2- Lee JG, Park HB, Shin HY et al . Effect of trendelenburg position on right and left internal jugular vein cross-sectional area. Korean J Anesthesiol. 2014 Nov;67(5):305-9.

3- Schummer W, Schummer C, Nieen WD, Gerstenberg H. Doppler-guided cannulation of internal jugular vein, subclavian vein and innominate ( brachiocephalic) vein -a case control comparison in patients with reduced and normal intracranial compliance. Intensive Care Med. 2003 Sep;29(9):1535-40

**Grant acknowledgment**

This study received confimation of Ethics Committee of the University.written consent was obtained from patients themselves or their first relatives.

The authors would like to thank Deputy of Research of Shiraz University of Medical

Sciences for its kindly cooperation. We are also thankful to the nursing staff of General and Central ICUs of Nemazee hospital for their kind help.

### A312 Inferior vena cava ultrasound assessment of central venous pressure in critical spontaneously breathing patients: a systematic review

#### N. Parenti^1^, F. Agrusta^2^, C. Palazzi^2^, B. Pifferi^2^, R. Sganzerla^2^, F. Tagliazucchi^2^, A. Luciani^2^

##### ^1^University of Modena, Bologna, Italy; ^2^University of Modena, Modena, Italy

###### **Correspondence:** Riccardo Sganzerla – University of Modena, Modena, Italy

**Introduction** The assessment of Central Venous Pressure (CVP) is fundamental in critical patients particularly in shock.

The inferior vena cava (IVC) ultrasound exam has been suggested to predict the CVP but there are few and divergent conclusions on the its effectiveness in spontaneously breathing patients.

**Objectives** To check the level of reliability, validity and correlation with CVP of the IVC ultrasound measures and the quality of reporting of literature on this topic.

**Methods** This review was based on the PRISMA guideline. The systematic search of the literature published from 1941 through 30 June 2015 explored the PubMed, Cochrane Library, Web of Knowledge and Scopus databases. Inclusion criteria were studies who investigated the reliability, the validity in predicting CVP and the correlation with CVP of the IVC ultrasound measures in adult (>18 yrs) spontaneously breathing patients. Two researchers selected studies using inclusion criteria and then assessed their quality using the STARD and QUADAS guidelines. The key words for literature search were: inferior vena cava, ultrasonography, volume status, central venous pressure.

**Results** We collected 593 studies: 148 excluded with reasons, 433 because duplicates. 12 studies were included for the final analysis with 7 reports which included spontaneously breathing patients and 5 a mixed population (spontaneous and ventilated): 3 on reliability, 10 on correlation with CVP, 5 on validity. The IVC ultrasound measures (IVC ratio, IVC MAX diameter, IVC MIN diameter , Caval index [IVC-CI]) showed an inter-rater agreement range from moderate to very good. The IVC MAX diameter had a significant high correlation with CVP; there were divergent conclusions on IVC-CI and poorly correlation for IVC ratio. The IVC-MAX and IVC-CI showed a good validity in predicting low CVP; the IVC Max and IVC-CI showed good validity in prediction high CVP in one study. Eight studies respected more than 60 % of the STARD items and five more than 80 % of QUADAS items.

**Conclusions** Because few reports have been published on the reliability and validity of IVC ultrasound measures the conclusions of this review should be confirmed. Anyway the quality of reporting and methodology of the studies collected were good. The IVC max and min diameter seem to correlate with CVP. All IVC measures show a good accuracy in predicting low or high CVP.

### A313 Transesophageal echocardiography in sedated patients requiring noninvasive ventilation by face mask

#### M. Möller, J. Müller- Engelmann, G. Montag, P. Adams, C. Lange, J. Neuzner, R. Gradaus

##### Klinikum Kassel, Cardiology and Intensive Care, Kassel, Germany

###### **Correspondence:** M. Möller – Klinikum Kassel, Cardiology and Intensive Care, Kassel, Germany

**Introduction** Transesophageal Echocardiography (TEE) is a standard procedure in Intensive Care. In patients with respiratory insufficiency, TEE with often necessary sedation may lead to further deterioration. We performed TEE in pts. already under NIV via a full face mask.

**Methods** Consecutive pts. already under NIV because of respiratory failure (15 pts, 8 m/7f , age 72 ± 11,2 years, SAPS II score 44 ± 12, PaO2/ FiO2 ≤ 250) with a clinically given indication for immediate TEE were equipped with a full face mask (“endoscopy mask“, VBM Medizintechnik GmbH, Sulz a.N.,Germany).

Ventilation was uniformely set (BiPAP f = 20/ min, 30-10 cmH2O, I:E 1:1, FiO2 1,0). For sedation, disoprivan was given as needed.

Heart rate, arterial blood pressures and transcutaneous SaO2 were monitored continuously. Blood gases were taken 15 min before, on start and then every five min troughout TEE and 15 and 60 min thereafter. Indications for TEE included endocarditis/ sepsis- focus, n = 8; intracavitary thrombus in tachyarrhythmia, n = 5; or quantification of vituim cordis, n = 2.

**Results** TEE was completed in all pts. Mean duration of the procedure was 11,6 ± 2,9 min, mean ejectionfraction was 45 ± 15 %. Percutaneous SaO2 never fell below 92 %. No pt. had to be intubated within 24 h. Mean HR (99,5 ± 13,7 / min), MAP (74,3 ± 3,2 mmHg), pH (7,41 ± 0,01), PaO2/ FiO2 - ratio (212 ± 21) und pCO2 (42,9 ± 1,8 mmHg) did not change significantly (p < 0,5, ANOVA).

In 11 pts. TEE lead to definite diagnosis. In 5 pts. immediate therapy (i.e. electrical cardioversion) was be performed

**Conclusions** Performed by experienced physicians, TEE at the bedside during NIV in sedated pts. was feasible and save.

### A314 Identification of the aorta by electrical impedance tomography

#### K.H. Wodack^1^, F. Thürk^2^, A.D. Waldmann^3^, M.F. Grässler^1^, S. Nishimoto^1^, S.H. Böhm^3^, E. Kaniusas^2^, D.A. Reuter^1^, C.J. Trepte^1^

##### ^1^University Medical Center Hamburg-Eppendorf, Department of Anesthesiology, Hamburg, Germany; ^2^Vienna University of Technology, Institute of Electrodynamics, Microwave and Circuit Engineering, Vienna, Austria; ^3^Swisstom AG, Landquart, Switzerland

###### **Correspondence:** K.H. Wodack – University Medical Center Hamburg-Eppendorf, Department of Anesthesiology, Hamburg, Germany

**Introduction** Electrical impedance tomography (EIT) is a noninvasive and radiation free bedside monitoring technology, primarily used to detect ventilation disorders. First experimental data in animals suggests that measurement of central hemodynamics within the descending aorta might become possible with EIT.^1^ To achieve this goal, it is first necessary to determine within the EIT images the exact location of an individual´s aorta.

**Objectives** The aim of this study was to improve and validate an algorithm to automatically detect the aorta by EIT using a hypertonic saline bolus.^2^

**Fig. 120 (abstract A314).**
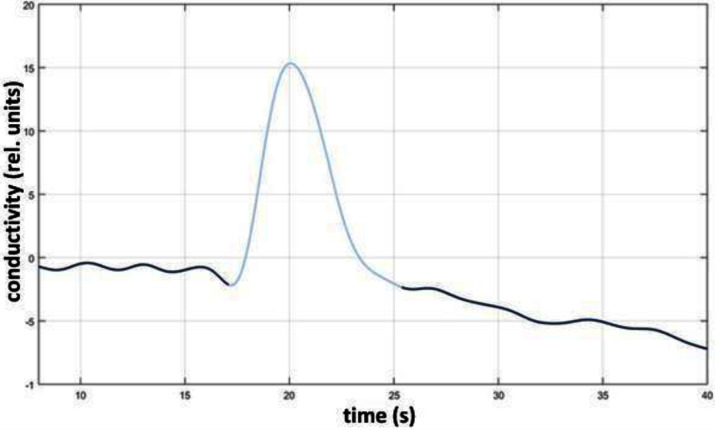
Example of peak in conductivity caused by the hypertonic saline bolus within the pixel representing the aorta; the dark blue line shows the filtered conductivity signal, while the light blue line marks the bolus event

**Methods** Ten domestic pigs were anesthetized and mechanically ventilated. A bolus of hypertonic saline (10 mL, 20 %), with a higher conductivity than blood was administered into the ascending aorta while EIT data were recorded. The resulting EIT images were analyzed pixel by pixel to identify the aortic pixel (p_A_), in which the bolus caused the highest transient impedance peak in time (Fig. 120). After completion of the EIT measurements a thoracic computed tomography scan (CT) was performed for each pig. The CT-images were segmented individually for the relevant anatomical structures. EIT images were reconstructed using the GREIT model, based on the individual´s thoracic contours derived from the segmented CT-images.^3^The resulting spatial resolution of EIT images was 3 mm / pixel.

**Fig. 121 (abstract A314).**
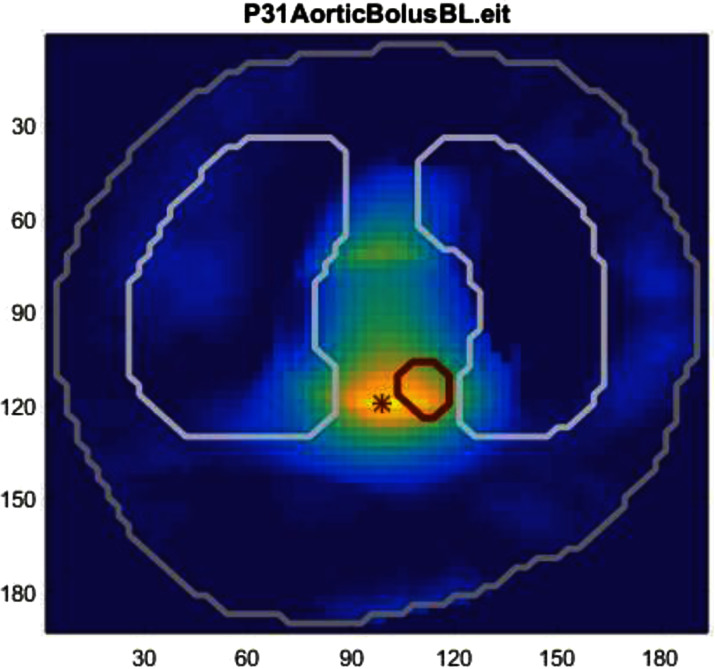
Examplary EIT image with superimposed CT-derived outer contours of thorax and lungs. The red circle marks the location of the aorta according to CT. The red asterisk indicates the location of aorta as detected by EIT using a bolus of hypertonic saline as contrast agent

**Results** The location of the aorta could be detected by EIT in all animals, showing a mean offset of 15 ± 7.5 mm when compared to the center of the true anatomical location identified by CT (Fig. 121).

**Conclusions** It is possible to detect the aorta by EIT using an intraaortic bolus of hypertonic saline. There is a misalignment between the location of the aorta identified by EIT and CT. The significance of this offset for an accurate measurement of fluid responsiveness needs to be determined in further studies.

**References**

1. Maisch S, Bohm SH, Solà J et al: Heart-lung interactions measured by electrical impedance tomography*.Crit Care Med* 2011; 39: 2173-6

2. Thürk F, Waldmann A et al: Hypertonic saline injection to detect aorta in porcine EIT. Accepted for 17^th^ conference on EIT 2016

3. Adler A, Arnold JH, Bayford R et al.: GREIT: a unified approach to 2D linear EIT reconstruction of lung images.*Physiol Meas* 2; 30: 35-55

**Grant acknowledgement**

The study was supported by departmental funds of the Department of Anesthesiology, University Medical Center Hamburg-Eppendorf, Germany.

### A315 A capnodynamic method for monitoring effective pulmonary blood flow - evaluation during hypercapnia

#### T. Sigmundsson^1,2^, T. Öhman^1,2^, E. Redondo^3^, M. Hallbäck^4^, M. Wallin^4^, F. Suarez Sipman^5^, A. Oldner^1,2^, C. Hällsjö Sander^1,2^, H. Björne^1,2^

##### ^1^Karolinska University Hospital, Department of Anaesthesiology, Surgical Services and Intensive Care Medicine, Stockholm, Sweden; ^2^Karolinska Institute, Department of Physiology and Pharmacology, Stockholm, Sweden; ^3^Hospital de Navarra, Department of Intensive Care Medicine, Pamplona, Spain; ^4^Maquet Critical Care, Stockholm, Sweden; ^5^Hedenstierna Laboratory, Department of Surgical Sciences, Uppsala, Sweden

###### **Correspondence:** T. Sigmundsson – Karolinska University Hospital, Department of Anaesthesiology, Surgical Services and Intensive Care Medicine, Stockholm, Sweden

**Introduction** A capnodynamic equation can be used to continuously calculate non shunted pulmonary blood flow (CO_EPBF_) during severe hemodynamic changes ADDIN EN.CITE ADDIN EN.CITE.DATA (1). Hypercapnia is a common clinical state both in the perioperative period and the ICU. Theoretically, elevated carbon dioxide levels could affect the performance of the capnodynamic method.

**Objectives** The aim of the current study was to evaluate the performance of CO_EPBF_ during elevated PvCO_2_ in a porcine model.

**Methods** The required alterations of alveolar concentration of carbon dioxide were created by a ventilatory pattern containing cyclic reoccurring expiratory holds during controlled ventilation. The mathematical model used to calculate CO_EPBF_ assumes a steady state in PvCO_2_ levels.

Hypercapnia was induced by three means; decreasing minute ventilation by either lowering respiratory rate or tidal volume, and finally, increasing dead space with preserved minute ventilation. CO_EPBF_ was compared to a reference method for CO, an ultrasonic flow probe around the pulmonary trunk. Hemodynamic measurements and blood gas analysis were obtained at baseline before hypercapnia and during the three different types of hypercapnia. Preload reduction (inflated cava balloon) and dobutamine stimulation was performed during low respiratory rate and increased dead space.

**Fig. 122:**
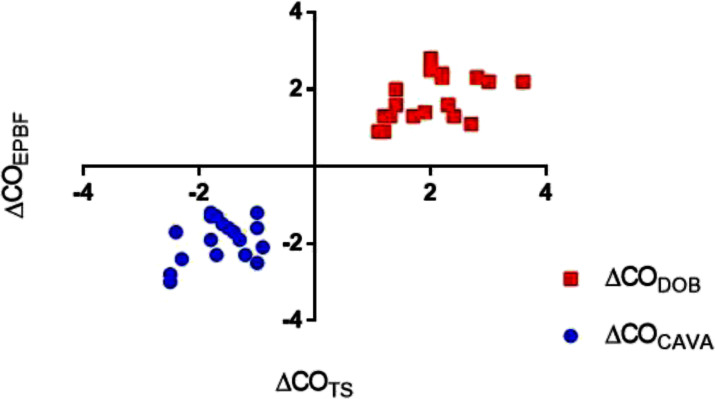
(abstract A315).

**Results** During hypercapnia the PaCO_2_ and PvCO_2_ levels were raised on average 58 % and 40 % (+/- 17 and 16 %) from within normal limits, respectively. Bias (LoA) att baseline before induction of hypercapnia was 0.5 L/min (-0.5 to 1.5) and percentage error (PE) 28 %. During hypercapnia, bias (LoA) was 1.4 L/min (0.2 to 2.7), PE 26 % following lower respiratory rate, 0.7 L/min (-0.7 to 2.2), PE 30 % when tidal volumes were decreased, and 0.5 L/min (-0.4 to 1.4), PE 19 % when dead space was increased. During hemodynamic changes the PE was slightly increased (see Table 100 for all values) and the concordance rate was 100 % (see Fig. 122).

**Conclusions** CO_EPBF_ performed well during hypercapnia, both during different types of low minute ventilation and increased dead space. The performance was maintained during major changes in cardiac output and trending was excellent. These results indicate that the capnodynamic method should be tested during lung protective ventilation with permissive hypercapnia and even in laparoscopic surgery.

**References**

1. Hallsjo Sander C, Hallback M, Wallin M, Emtell P, Oldner A, Bjorne H. Novel continuous capnodynamic method for cardiac output assessment during mechanical ventilation. British journal of anaesthesia. 2014;112(5):824-31.

**Grant acknowledgement**

Håkan Björne received research grant from Maquet Critical Care.

**Table 100:** (abstract A315).

Intervention	COEPBF (L/min)	COTS (L/min)	Shunt (%)	Dead space (%)	PaCO2 (kPa)	PvCO2 (kPa)	Bias (L/min)	LoA (L/min)	PE (%)
Normocapnia	3.8 (0.5)	3.3 (0.5)	8 (3)	55 (3)	5.56 (0.42)	7.41 (0.69)	0.5	-0.5 to 1.5	28
Hypercapnia low RR	5.5 (0.9)	4.1 (0.8)	13 (4)	47 (9)	8.52 (0.79)	9.83 (1.22)	1.4	0.2 to 2.7	26
*>Caval occlusion*	3.1 (0.5)	2.3 (0.4)	7 (2)	53 (4)	8.05 (0.84)	10.46 (1.00)	0.8	0.1 to 1.4	24
*>Dobutamine*	7.0 (0.7)	5.5 (0.7)	19 (5)	45 (10)	9.40 (0.74)	10.65 (1.02)	1.6	-0.1 to 3.2	27
Hypercapnia low TV	5.6 (1.0)	4.9 (0.6)	13 (4)	66 (4)	9.23 (0.32)	10.94 (0.46)	0.7	-0.8 to 2.2	30
Hypercapnia dead space	5.0 (0.7)	4.5 (0.5)	13 (4)	78 (2)	9.06 (0.49)	10.50 (0.91)	0.5	-0.4 to 1.4	19
*>Caval occlusion*	3.1 (0.5)	2.7 (0.2)	7 (3)	80 (2)	8.34 (0.42)	10.33 (0.95)	0.4	-0.5 to 1.3	31
*>Dobutamine*	5.0 (0.7)	4.4 (0.5)	14 (4)	78 (3)	9.91 (0.43)	10.46 (1.32)	-0.04	-2.3 to 2.2	33

### A316 Ecocardiographyc measurement of cardiac output through modified subcostal window: consistency analysis with conventional methods in critically-ill patients

#### L. Colinas^1^, G. Hernandez^1^, R. Vicho^2^, M. Serna^3^, R. Cuena^4^, A. Canabal^1^, ECOCRITIC group

##### ^1^Hospital Virgen de la Salud, SESCAM, Intensive Care Medicine, Toledo, Spain; ^2^Hospital Quironsalud Palmaplanas, Intensive Care Medicine, Palma de Mallorca, Spain; ^3^Hospital Marina Salud Denia, Intensive Care Medicine, Murcia, Spain; ^4^Hospital Virgen de la Salud, SESCAM, Research Unit, Medical Council, Toledo, Spain

###### **Correspondence:** L. Colinas – Hospital Virgen de la Salud, SESCAM, Intensive Care Medicine, Toledo, Spain

**Introduction** Echocardiography in the setting of the critically ill patient might be hindered due to poor transthoracic acoustic window. The modified subcostal window, which is obtained from an extrathoracic region, gives us a short-axis parasternal-like view, at the level of the great vessels.

**Objectives** To address the consistency of cardiac output values, measured from the modified subcostal view as compared with those obtained from a transthoracic approach.

**Methods** In 54 consecutive critically-ill patients undergoing transthoracic ecocardiography (Philips Sparq) for both initial diagnosis of shock or subsequent hemodynamic monitoring, velocity time integral of pulsed wave Doppler of the left ventricular outflow tract (VTI_LVOT_) and the pulmonary flow (VTI_Pu-Ps_ and VTI_Pu-Ms_) were measured from the apical four-chamber view, the short-axis paraesternal and modified subcostal views respectively. Results were analyzed with predictive performance test and the consistency analysis. Interobserver reproducibility analysis was assessed in 20 patients with repeated measurements by two experienced physicians using the intraclass correlation coefficient (ICC).

**Fig. 123:**
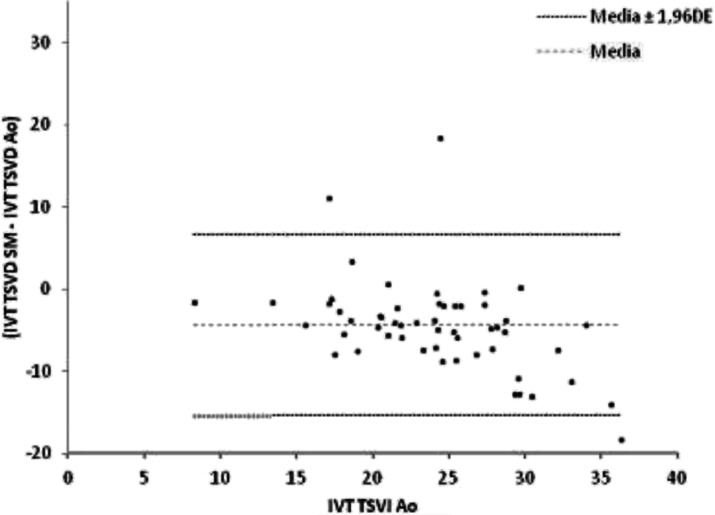
(abstract A316).

**Fig. 124:**
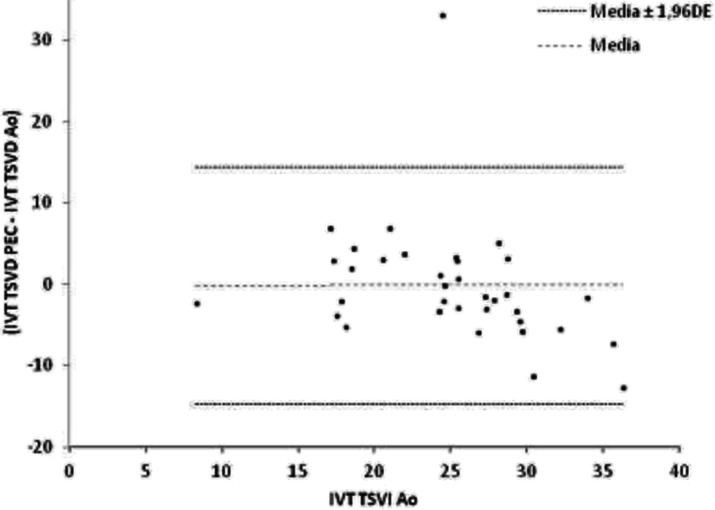
(abstract A316).

**Results** Baseline characteristics included: mean heart rate 79 beats per minute (15.7), age 59.2 years (15.6), male 29 (53.7 %), previus cardiac disease 9 (16.7 %), medical diagnosis at admission in 40 patients (74.1 %, with primary cardiac diagnosis in 8 [14.8 %]); 19 mechanically ventilated patients (35.2 %, mean PEEP 6.8 [2.8]), with vasoactive drugs in 13 patients (24.1 %, mean norepinephrine dose .38 [.29] mcg/Kg/min). Median VTI_LVOT_ was 24.3 cm (interquartile range [IQR] 8-36), VTI_Pu-Ps_ 24.5 cm (IQR: 6-57) and VTI_Pu-Ms_ 18.9 cm (IQR: 7-43). The comparison between VTI_LVOT_ and VTI_Pu-Ms_ revealed a consistency of .52 (95%CI .29 to .69, p < .001), see Fig. 123; and between VTI_LVOT_ and VTI_Pu-Ps_ .44 (95%CI .13 to .67, p = .004), see Fig. 124. The ICC observed was .93.

**Conclusions** The consistencies between VTI_LVOT_ and VTI_Pu-Ms_ and VTI_LVOT_ and VTI_Pu-Ps_ were moderate but VTI_LVOT_ and VTI_Pu-Ms_ consistency improved after excluding patients with VTI_LVOT_ >29 cm. VTI_Pu-Ms_ underestimate VTI_LVOT_ a mean 4.45 cm. Further research is required in order to assess it.

### A317 Usefulness of cardiac function index as a surrogate of left ventricle ejection fraction in patients with acute circulatory failure

#### A. Chaari^1^, K. Abdel Hakim^2^, M. Etman^2^, M. El Bahr^2^, A. El Sakka^2^, K. Bousselmi^2^, A. Arali^2^, V. Kauts^2^, W.F. Casey^2^

##### ^1^King Hamad University Hospital, Intensive Care, Muharaq, Bahrain; ^2^King Hamad University Hospital, Muharaq, Bahrain

###### **Correspondence:** A. Chaari – King Hamad University Hospital, Intensive Care, Muharaq, Bahrain

**Introduction** The assessment of the left ventricle function is of paramount importance for the management of patients with acute circulatory failure. In this regard, the measurement of the left ventricle ejection fraction (LVEF) by echocardiography is considered to be the gold standard. Only few studies investigated the correlation between the LVEF and the Cardiac Function Index (CFI) calculated through transpulmonary thermodilution [1, 2].

**Objective** To assess the usefulness of cardiac function index (CFI) as a surrogate of left ventricle ejection fraction (LVEF) in patients with acute circulatory failure.

**Methods** We conducted a prospective study in a 12-bed medical surgical intensive care unit. All patients admitted with acute circulatory failure and monitored with a transpulmonary thermodilution device were included in our study. We excluded the patients with acute core pulmonale and those with poor echogenicity. A bedside trans-thoracic echocardiography was performed simultaneously with transpulmonary thermodilution. Cardiac function index and left ventricle ejection fraction were recorded simultaneously.

**Results** Twenty-one patients were included in our study. Median [quartile] age was 66 [57-81] years. Sex-ratio (M/F) was 12/9. Fourteen patients (66.7 %) were admitted with septic shock whereas 7 patients (33.3 %) had cardiogenic shock. Mechanical ventilation was required for 13 patients (61.9 %). Median [quartile] CFI was 4.5 [2.75 - 6] min^-1^. Median [quartile] LVEF was 60 [45.75 - 66] %. Spearman coefficient was 0.844 (p < 0.001). Moreover, a CFI > 4.5 min^-1^ predicted a LVEF > 60 % with a sensitivity of 100 % and a specificity of 82 %.

**Conclusion** CFI can be used as a reliable tool to predict LVEF in patients with acute circulatory failure.

References

1. Jabot J, Monnet X, Bouchra L, Chemla D, Richard C, Teboul JL. Cardiac function index provided by transpulmonary thermodilution behaves as an indicator of left ventricular systolic function. Crit Care Med. 2009; 37(11):2913-8.

2. Combes A, Berneau JB, Luyt CE, Trouillet JL. Estimation of left ventricular systolic function by single transpulmonary thermodilution. Intensive Care Med. 2004;30(7):1377-83.

**Grant acknowledgment**

None.

### A318 Correlation between microcirculatory perfusion and arterial elastance

#### O. Bond^1^, P. De Santis^1^, E. Iesu^1^, F. Franchi^2^, J.-L. Vincent^1^, J. Creteur^1^, S. Scolletta^2^, F.S. Taccone^1^

##### ^1^Hopital Erasme, Université Libre de Bruxelles, Department of Intensive Care, Brussels, Belgium; ^2^University of Siena, Anesthesia and Intensive Care Unit, Department of Medical Biotechnologies, Siena, Italy

###### **Correspondence:** O. Bond – Hopital Erasme, Université Libre de Bruxelles, Department of Intensive Care, Brussels, Belgium

**Objectives** Hypotension is a common problem in critically ill patients. Arterial blood pressure (ABP) is influenced by changes in intravascular volume, cardiac output and vascular tone. In particular, systemic vascular resistance (SVR) is a main determinant of arterial elastance (Ea); SVR is also influenced by peripheral endothelial function (e.g. microcirculatory flow). However, few data are available on the correlation of these parameters in this setting.

**Methods** Prospective study conducted in a 35-bed medico-surgical ICU since January 2016. Patients with an invasive ABP monitoring and requiring a cardiac output (CO) monitoring during a fluid challenge (FC) were simultaneously assessed with a pulse wave analysis (PWA) system (MostCare, Vygon, France) to estimate Ea and with an Incident Dark Field (IDF) handheld device (Braedius Medical BV, The Netherlands) to evaluate sublingual microcirculation. Microvascular perfusion was assessed using the proportion of small-perfused vessels (PPV). SVR was calculated according to standard formulas. Relative changes in each variable were calculated before and after FC; fluid responders had a CO increase of at least 10 % from baseline.

**Results** We included 13 patients (age 64 [54-73] years; 7 male) requiring a fluid challenge (n = 6 for hypotension; n = 5 for oliguria; n = 2 for hypovolemia). At baseline, mean arterial pressure was 72 [66-84] mmHg, heart rate (HR) 83 [80-106] bpm and CO 4.2 [3.5-4.4] L/min; after fluid challenge, MAP was 73 [71-74] mmHg, HR 85 [77-103] bpm and CO 4.8 [3.9-5.4] L/min. Seven patients were fluid responders. There was no correlation between PPV and SVR (ρ = 0.12; p = 0.23) or Ea (ρ = 0.03; p = 0.56). Likewise changes in SVR and Ea during fluid challenge were not correlated with changes of PPV (-ρ = 0.01; p = 0.91).

**Conclusions** No correlation was found between either SVR or elastance and indexes of microvascular perfusion in the sublingual region. The impact of microcirculatory perfusion on the arterial load should be further defined.

### A319 Comparison of the transthoracic doppler sonography and echocardiography in cardiac output measurement in severe trauma patients

#### Z. Marutyan, L. Hamidova, A. Shakotko, V. Movsisyan, I. Uysupova, A. Evdokimov, S. Petrikov

##### N.V. Sklifosovsky Research Institute of Emergency Medicine of the Moscow Healthcare Department, Moscow, Russian Federation

###### **Correspondence:** Z. Marutyan – N.V. Sklifosovsky Research Institute of Emergency Medicine of the Moscow Healthcare Department, Moscow, Russian Federation

**Introduction** Monitoring of a systemic hemodynamic at victims with the severe combined injury allows to outline the volume and structure of an infusion therapy. At this moment invasive methods are typically used for monitoring of a systemic hemodynamic. However, invasive monitoring is impossible or extremely limited in some patients because of a mode of failure (for example, pelvic fracture) and a consumable cost. In such cases non-invasive monitoring of a systemic hemodynamic can be used as an optional method.

**Objective** to compare cardiac output measurements between transthoracic Doppler sonography and echocardiography (EchoKG).

**Material and methods** Twenty four patients at the age of 41 ± 14 enrolled in the study (women/men - 3/19, traumatic brain injury - 3, a spinal trauma - 2, a concomitant injury- 19). We determined a stroke volume (SV) and a cardiac output (CO) with the use of the transthoracic Doppler sonography ("USCOM", Australia) and the transthoracic EchoKG (GE Vivid Q, USA).

Transthoracic doppler sonography was provided by doctors of the intensive care unit, EchoKG was performed by doctors of the functional diagnostics.

**Results** We didn't find differences between the average values SV and CO evaluated by DS and EchoKG: 67,2 ± 21,4 ml vs 64,2 ± 18 ml, and 6,5 ± 2,4 l/min vs 6,4 ± 2 l/min.

**Conclusion** Usage of transthoracic doppler sonography for determining systemic hemodynamic values at victims with a severe injury allows to receive the measured data of the systolic discharge and cardiac output comparable to data of transthoracic EchoKG.

TDS provided a comparable results of SV and CO measurements with EchoKG and could be used in trauma care practice.

### A320 Cerebral oximetry assessed by near -infrared spectrometry in patients with postresuscitation syndrome

#### C. Gonen^1^, E. Haftacı^2^, C. Balci^1,2^

##### ^1^Kocaeli Derince Training Hospital, Intensive Care, Kocaeli, Turkey; ^2^Kocaeli Derince Education and Research Hospital, Kocaeli, Turkey

###### **Correspondence:** C. Balci – Kocaeli Derince Training Hospital, Intensive Care, Kocaeli, Turkey

**Introduction** Transcranial cerebral oximetry is a non-invasive method to monitor the changes in the cerebral oxygen metabolism. It is a near-infrared spectroscopy method that uses multi-wavelength radiation between 690-1100 nm spectrum. This photons can pass skin, bone, brain and cerebrospinal fluid.

**Objectives** The aim of the study is to analyze the association of monitoring transcranial cerebral oximertry with morbidity and mortality in patients with postresuscitation syndrome.

**Methods** In this study we retrospectively analyzed the data of 23 patients with postresuscitation syndrome. The data included age, sex, arrest time, location of arrest, light reflex, Glascow coma scale and cerebral oximetry values compared with mean arterial pressures, SpO2 values and survival.

**Results** Glascow coma scale and survival are well correlated with higher values of cerebral oximetry (p < 0.05).

**Conclusions** This study suggests that, high cerebral oxygen saturation values in patients with postresuscitation syndrome are associated with lower morbidity and mortality.

**References**

Association between hemoglobin, cerebral oxygenation and neurologic outcome in postcardiac arrest patients I Meex, K Ameloot, C Genbrugge, M Dupont, B Ferdinande, J Dens, C Dedeyne Crit Care. 2015; 19(Suppl 1): P430. Published online 2015 March 16. doi: 10.1186/cc14510.

### A321 Goal-directed therapy guiaded by dynamics preload variables (SVV and VVP) after major hepatic resection can help intraoperative fluid optimization and reduce post-surgical complications

#### F.J. Redondo Calvo^1^, N. Bejarano^2^, V. Baladron^3^, R. Villazala^3^, J. Redondo^3^, D. Padilla^4^, P. Villarejo^4^

##### ^1^Facultad de Medicina Ciudad Real, Hospital General Universitario de Ciudad Real, Anestesiologia y Reanimacion, Ciudad Real, Spain; ^2^Facultad de Medicina Ciudad Real, Hospital General Universitario Ciudad Real, Cuidados Criticos Pediatricos, Ciudad Real, Spain; ^3^Hospital General Universitario Ciudad Real, Anestesiologia y Reanimacion, Ciudad Real, Spain; ^4^Facultad de Medicina Ciudad Real, Hospital General Universitario Ciudad Real, Cirugía Hepatobiliar, Ciudad Real, Spain

###### **Correspondence:** F.J. Redondo Calvo – Facultad de Medicina Ciudad Real, Hospital General Universitario de Ciudad Real, Anestesiologia y Reanimacion, Ciudad Real, Spain

**Introduction** Classically, central venous pressure (CVP) and pulmonary artery occlusion pressures (PAOPs) have been used as surrogates for volume measurements.However dynamic preload variables like as pulse pressure variations (PPV) and stroke volumen variations (SVV) could be very useful in hepatic postresection phase to optimize the volumen needed for the patients.

**Objectives** The aim of this study is to assess if is better to reduce postoperative complications, optimizing hemodynamic situation after hepatic resection guided by dynamic variables preload (PPV and SVV) versus using liberal fluid management.

**Methods** Experimental clinical trial, controlled, randomized, single blind, in patients undergoing hepatic resection. In both groups perioperative fluid restriction was done (5 ml/kg/hour of Ringer Lactate) until removal of the surgical specimen was performed. Affer that, two randomized groups were established. In the control group fluids (colloids) were administered until hemodiámica stability was achiveved and standard pressures were got (MAP > 65 mmHg CVP 8-14, urine output > 0.5 ml / kg / h). In the other group volume was administered until a SVV < 12 and a PPV < 14 and MAP > 65 mmHg were achieved.

In both groups the volume administered to achieve quantified objectives and postoperative complications (nausea and vomiting, respiratory and infectious complications) was registred.

**Results** 9 patients were enrolled in the GDT group and 10 patients in the control group. There were no statistically significant differences in preoperative variables. A statistically significant difference in the volume administered after resection in both groups (1290 +/- 375 vs 128 +/- 485.55, p < 0.01) was found. In the surgical time we found no correlation between VPP and CVP (r = 0,172, p = 0.656) and between the VSS and CVP (r = 0,243, p =0.492). We found very good ability to predict the response to volume with both the VPP (ROC curve: 0.96) as the VSS (ROC curve: 0.92), defined as the 20 % improvement in cardiac output. We found a decrease in complications but no statistically significant differences in respiratory complications (4 vs 2, p = 0.62), infectious complications (5 vs 2, p = 0.35) and nausea and vomiting (6 vs 1, p = 0.057).

**Conclusions** Guided therapy goals (GTD) is able to decrease the volume of liquid provided after hepatic resection. This fact optimize hemodynamics values in these patients (cardiac output, mean arterial pressure) and therefore, reduce postoperative complications due to excessive intake volumen.

**References**

1. De Wolf Am, Aggarwal S. Monitoring preload during liver transplantation. Liver Traspl 2008; 14: 268-269

**Grant acknowledgement**

We express our gratitudes to Mutua Madrileña Fundation (Madrid, Spain) for its grant collaboration by without which this work could not have been completes.

## RRT NEW DEVELOPMENTS FOR AKI

### A322 Impact of cumulative nephrotoxin exposure on acute kidney injury in the pediatric critically ill patients

#### A. Akcan-Arikan^1,2^, C.E. Kennedy^1^

##### ^1^Baylor College of Medicine, Pediatric Critical Care, Houston, TX, United States; ^2^Baylor College of Medicine, Pediatric Nephrology, Houston, TX, United States

###### **Correspondence:** A. Akcan-Arikan – Baylor College of Medicine, Pediatric Critical Care, Houston, TX, United States

**Introduction** Critically ill children are exposed to multiple nephrotoxic medications due to the nature of the underlying disease process as well as comorbid conditions. Acute kidney injury (AKI) is prevalent in the pediatric critically ill. Nephrotoxin exposure is a potentially modifiable risk factor for AKI in non-critically ill children, with exposure to three or more discrete nephrotoxic agents significantly increasing odds of AKI. Despite higher inherent risk, data on AKI and nephrotoxin use in the pediatric intensive care unit (PICU) setting is scarce. Fluid Overload Kidney Injury Score (FOKIS) is a decision support tool piloted in our PICU as a daily score incorporating subscores for AKI (pRIFLE creatinine and urine output), fluid overload (total fluid (in- out)/ICU admission weight), and exposure to nephrotoxic medications (a priori determined list of medications).

**Objectives** We aimed to investigate AKI prevalence and frequency of nephrotoxin exposure using FOKIS subscores.

**Methods** Retrospective analysis of daily FOKIS subscores in PICU patients (pts) over 18 months. AKI was defined and staged using pRIFLE creatinine criteria. Nephrotoxin exposure (Exp) was defined as exposure to three or more nephrotoxic agents in 24 hours. Each additional medication exposure was also recorded.

**Results** 2830 pts (median age 5.5 years (IQR 1.3-12.9 years), 55 % male) were included over 18 months. 246 pts (8.7 %) had Exp during PICU stay (120 (49 %) to 3, 73 (30 %) to 4, 30 (12 %) to 5, 14 (5.7 %) to 6, 9 (3.7 %) to 7 different nephrotoxins). Fifty eight percent (142/246) of pts with Exp had AKI (41 (16.7 %) R, 29 (11.8 %) I, 72 (29.3 %) F) compared to 16 % (413/2584) without (201 (7.8 %) R, 90 (3.5 %) I, 122 (4.7 %) F) (p < 0.001).

**Conclusions** A majority of PICU pts with AKI had exposure to nephrotoxins. Patients who had exposure to three or more nephrotoxins had increased AKI compared to patients who did not. This association needs to be further studied prospectively to determine causality, specifically exploring timing of AKI onset in relation to nephrotoxin exposure in critically ill pediatric patients. Cumulative nephrotoxin exposure is a possible modifiable AKI risk factor in critically ill children.

**Table 101 (abstract A322).** Cumulative nephrotoxin exposure and AKI

Exp	No AKI	Risk	Injury	Failure
No exposure	2171, (84%)	201, (7.8%)	90, (3.5%)	122, (4.7%)
3 meds	59, (49.2%)	22, (18.3%)	12, (10%)	27, (22.5%)
4 meds	28, (38.4%)	11, (15.1%)	11, (15.1%)	23, (31.5%)
5 meds	12, (40%)	6, (20%)	2, (6.7%)	10, (33.3%)
6 meds	5, (35.7%)	1, (7.1%)	2, (14.3%)	6, (42.9%)
7 meds	0, (0%)	1, (11.1%)	2, (22.2%)	6, (66.7%)

### A323 Effectiveness of renal angina index score predicting acute kidney injury on critically ill patients

#### M.F. Aguilar Arzapalo

##### SSA UADY, Mérida, Mexico

**Introduction** Until two thirds of critically ill patients develop Acute Kidney Injury (AKI) and it is associated with an increased risk of death. Renal Angina Index is a score that evaluates the risk of presenting AKI.

**Objectives** This study was used to determine the effectiveness of the Renal Angina Index as a developing predictor of AKI in 3 days.

**Methods** The study was based on a prospective cohort of critically ill patients in whom Renal Angina Index score was completed with a 72 hour follow up of serum creatinine levels, water balance and urinary output, establishing after patients that develop AKI. Afterwards a relationship was established between Renal Angina Index score and its predictive capacity of developing AKI, determining sensibility, specificity, positive predictive value and negative predictive value for the score.

**Results** A final sample of 206 patients was obtained at the end of the study. The incidence of AKI in the studied population was 27.2 % (n = 56), and the average scores of Renal Angina Index were 20.52 in those who develop AKI and 4.35 in those who didn't develop AKI. This score offers a 90.7 % sensibility, 95.4 % specificity with an area under de curve of 0.963 (0.934-0.991). A positive predictive value of 0.88 was obtained and a negative predictive value 0.97

**Conclusions** Renal Angina Index score is effective predicting AKI in critically ill adult patients.

**Note:** This abstract has been previously published and is available at [4]. It is included here as a complete record of the abstracts from the conference.

**References**

1. Chawla L, Goldstein S, Kellum J, Ronco C. Renal Agina: concept and development pretest probability assessment in acute kidney injury. Critical Care. 2015; 19: 93.

2. Basu R, Zappitelli M, Brunner L, Wang Y, Wong H et al. Kidney International. 2013; 85, 659-667.

3. Basu R, Wang Y, Wong H, Chawla L, Wheeler D, Goldstein S, Incorporation of biomarkers with the renal angina index for prediction of severe AKI in critically ill children. Clin J Am Soc Nephrol. 2014; 9: 654-662.

4. Aguilar Arzapalo M, Barradas L, Lopez V, Escalante A, Jimmy G, Cetina M (2016) Effectiveness of renal angina index score predicting acute kidney injury on critically ill patients. Critical Care 20(Suppl 2): 94.

**Grant acknowledgement**

To Hospital O´Horán.

**Fig. 125 (abstract A323).**
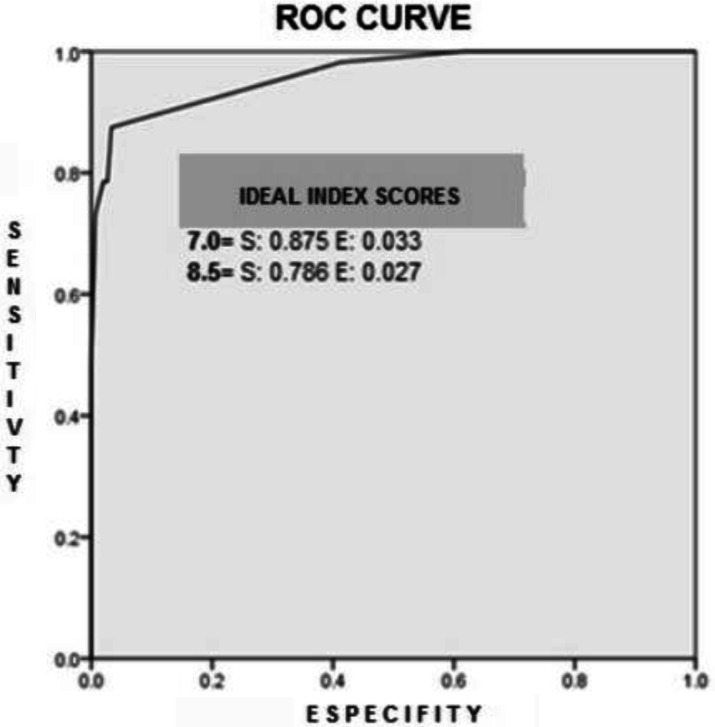
ROC Curve ''Angina Renal Index Score''

### A324 Predictive model for contrast-associated acute kidney injury in critical patients

#### C. Gomez-Gonzalez^1^, S. Mas-Font^2^, A. Puppo-Moreno^1^, M. Herrera-Gutierrez^3^, M. Garcia-Garcia^4^, S. Aldunate-Calvo^5^, NEFROCON Investigators

##### ^1^Hospital U.V. Rocío, Seville, Spain; ^2^Hospital General Universitario de Castellon, Castellon, Spain; ^3^H Carlos Haya, Malaga, Spain; ^4^Hospital de Sagunto, Valencia, Spain; ^5^Complejo Hospitalario de Navarra, Pamplona, Spain

###### **Correspondence:** C. Gomez-Gonzalez – Hospital U.V. Rocío, Seville, Spain

**Introduction** Incidence of acute kidney injury in critically ill patients increases with chronic diseases, nephrotoxic drugs, and the use of contrast in diagnostic/therapeutic techniques. Contrast-associated acute kidney injury (CA-AKI) is strongly predictive of adverse outcomes.

**Objectives** To build a statistical predictive model to evaluate the probability of developing contrast-associated acute kidney injury in critical patients.

**Fig. 126 (abstract A324).**
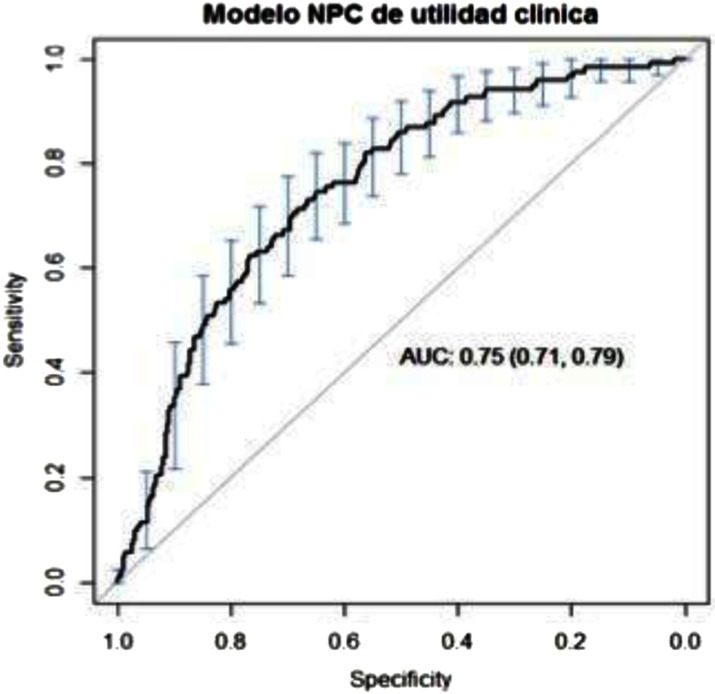
ROC curve of the model

**Fig. 127 (abstract A324).**
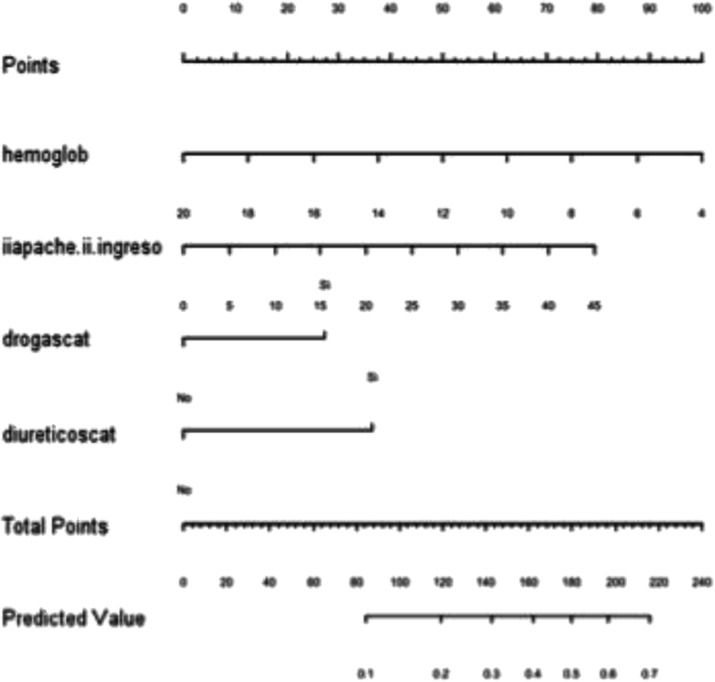
Nomogram of the predictive model

**Methods** This study has been endorsed by the Spanish Society of Intensive Critical and Emergency Care Medicine (SEMICYUC). Data were obtained in a prospective multicenter study in 33 Spanish Intensive Care Units, with a total of 1009 patients. The criteria used to define CA-AKI was the AKIN criteria: a rise of serum creatinine of ≥0.5 mg/dl or a 50 % relative rise in creatinine at 48-72 hours after contrast exposure. The predictive model has been developed employing a binary logistic regression using the software R. The ROC curve was obtained (Fig. 126) and the model was calibrated using this graph. From the model, we have generated a graphical nomogram (Fig. 127) to facilitate its use in a clinical environment. The nomogram includes the 4 variables shown to have prognostic value.

**Results** 12 % of the patients developed CA-AKI. Predicting factors were elevated APACHE II test score, hemoglobin and baseline serum creatinine, shock or acute myocardium infarct at admission, vasoactive drugs and diuretics at the moment of the contrast administration, and the following comorbidities: chronic heart failure and chronic kidney failure. Significant risk factors in the univariate analysis were selected for the predictive model (Table 102). A bootstrap method was used to select the best subset of risk factors to avoid overfitting the data. The corresponding ROC curve of the model (Fig. 126) has an area under the curve of AUC = 0.75 (range 0.71-0-79).

**Conclusions** A predictive model of CA-AKI has been developed. Predicting variables with prognostic value are the hemoglobin content, the APACHE II test score on admission and the use of vasoactive drugs and of diuretics. The corresponding nomogram allows for easy evaluation of the probability of developing CA-AKI in critical patients.

**References**

1. Hoste EA, Doom S, De Waele J et al. Epidemiology of contrast-associated acute kidney injury in patients: a retrospective cohorte analisis. *Intensive Care Med* 2011; 37(12):1921-31.

2. McDonald J, MacDonald R, Comin J et al. Frequency of Acute Kidney Injury Following Intravenous Contrast Medium Administration: A Systematic Review and Meta-Analysis. *Radiology* 2013; 267(1):119-128.

**Table 102 (abstract A324).** Risk factors selected for the predictive model

	Significance (p)	Odds Ratio	Confidence Interval 95%
APACHE II	<0.01	1.04	1.069-1.014
Hemoglobin	<0.01	0.86	0.942-0.793
Diuretics	<0.01	2.31	3.515-1.513
Vasoactive drugs	<0.01	1.87	2.960-1.174

### A325 Impact of continuous veno-venous hemodyalisis with an increased adsorption membrane in septic acute kidney injury

#### E.P. Plata-Menchaca^1^, X.L. Pérez-Fernández^2,3^, M. Estruch^4^, A. Betbese-Roig^5^, P. Cárdenas Campos^2^, M. Rojas Lora^2^, N.D. Toapanta Gaibor^2^, R.S. Contreras Medina^2^, V.D. Gumucio Sanguino^2^, E.J. Casanova^2^, J. Sabater Riera^2,3^, SIRAKI group

##### ^1^Instituto Nacional de Ciencias Médicas y Nutrición Salvador Zubirán, Critical Care, México, Mexico; ^2^Hospital Universitari de Bellvitge, Critical Care, L´Hospitalet, Spain; ^3^Instituto de Investigacion Biomédica de Bellvitge, L´Hospitalet, Spain; ^4^Hospital de la Santa Creu i Sant Pau, Institut de Recerca Cardiovascular, Barcelona, Spain; ^5^Hospital de la Santa Creu i Sant Pau, Critical Care, Barcelona, Spain

###### **Correspondence:** E.P. Plata-Menchaca – Instituto Nacional de Ciencias Médicas y Nutrición Salvador Zubirán, Critical Care, México, Mexico

**Introduction** It is postulated that the effects of continuous renal replacement therapy (CRRT) delivered as hemofiltration (CVVH-ST150) might benefit critically ill patients with sepsis-associated acute kidney injury (AKI), by better clearing large toxic inflammatory cytokines.

**Objectives** To compare CRRT delivered as hemodialysis associated with an adsorptive membrane (CVVHD-ST150), with CRRT delivered as CVVH-ST150 in 81 septic patients with AKI.

**Methods** In this randomized non-blinded control trial, patients were recruited from 2 ICUs in Barcelona (Spain), and were assigned to one of two arms of intervention: CVVH-ST150 or CVVHD-ST150. The primary outcome was 90-day mortality. Secondary outcomes were inflammatory cytokine clearance, consumption of extracorporeal circuits, ICU and hospital length-of-stay (LOS), and presence of CRRT associated complications (*dialytrauma)*.

**Results** The median duration of follow up was 130 days. Mean age was 63.5 ± 13.3 years and distribution of baseline characteristics were similar between groups. Overall 90-day mortality was 56.1 % in the CVVH-ST150 group and 50 % in the CVVHD-ST 150 group (p = 0.94). Number of extracorporeal circuits employed after 72 hours of CRRT initiation were 4.08 ± 1.8 in the CVVH-ST150 group and 3.31 ± 1.28 in the CVVHD-ST150 group (p = 0.038). Median extracorporeal circuit lifespan was 18,91 ± 6 and 20,63 ± 4.3 hours in the CVVH-ST150 group and the CVVHD-ST150 group (p = 0.019), respectively. Heparin was employed in 26.8 % in the CVVH-ST150 group and 15 % in the CVVHD-ST150 group (p = 0.27). Mean ICU stay was 17.3 ± 14.3 days and 12.4 ± 11.9 days in CVVH-ST150 group and CVVHD-ST150 group (P = 0.098), respectively. Hospital length-of stay (LOS) was 33.5 ± 25.7 in the CVVH-ST150 group, as compared with 21.2 ± 18.5 in CVVHD-ST150 group (P = 0.016). Serum levels of inflammatory cytokines were similar between groups.

**Conclusions** There were no differences on survival, dialytrauma and inflammatory cytokine clearance between CVVH-ST150 and CVVHD-ST150 groups. However, the use of CVVHD-ST150 was associated with a lower hospital LOS and less consumption of extracorporeal circuits during the first 72 hours of CRRT.

**Grant acknowledgments**

FEDER & ISCIII.

### A326 The impact of macro- and micronutrients on predicting outcomes of critically ill patients requiring continuous renal replacement therapy

#### K. Kritmetapak^1^, S. Peerapornratana^1^, P. Kittiskulnam^1^, T. Dissayabutra^2^, K. Tiranathanagul^1^, P. Susantithapong^1^, K. Praditpornsilpa^1^, K. Tungsanga^1^, S. Eiam-Ong^1^, N. Srisawat^1^

##### ^1^Division of Nephrology, Chulalongkorn University, Department of Medicine, Bangkok, Thailand; ^2^Chulalongkorn University, Department of Biochemistry, Bangkok, Thailand

###### **Correspondence:** S. Peerapornratana – Division of Nephrology, Chulalongkorn University, Department of Medicine, Bangkok, Thailand

**Introduction** Critically ill patients with acute kidney injury (AKI) who receive renal replacement therapy (RRT) have very high mortality rate. During RRT, there are markedly changes in the metabolism of macro- and micronutrients which may cause malnutrition and result in impaired renal recovery and patient survival.

**Objectives** We aimed to examine the predictive role of macro- and micronutrients on survival and renal outcomes in critically ill patients undergoing continuous RRT (CRRT).

**Methods** This prospective observational study enrolled critically ill patients requiring CRRT at Intensive Care Unit of King Chulalongkorn Memorial Hospital from November 2012 until November 2013. The serum, urine, and effluent fluid were serially collected on the first three days to calculate protein metabolism including dietary protein intake (DPI), nitrogen balance, and normalized protein catabolic rate (nPCR). Serum zinc, selenium, and copper were measured for micronutrients analysis on the first three days of CRRT. Survivor was defined as being alive on day 28 after initiation of CRRT. Dialysis status on day 28 was also determined.

**Results** Of the 70 critically ill patients requiring CRRT, 27 patients (37.5 %) survived on day 28. The DPI and serum albumin of survivors were significantly higher than non-survivors (0.8 ± 0.2 vs 0.5 ± 0.3 g/kg/day, p = 0.001, and 3.2 ± 0.5 vs 2.9 ± 0.5 g/dl, p = 0.03, respectively) while other markers were comparable. The DPI alone predicted patient survival with area under the curve (AUC) of 0.69. A combined clinical model predicted survival with AUC of 0.78. When adjusted for differences in albumin level, clinical severity score (APACHEII and SOFA score), and serum creatinine at initiation of CRRT, DPI still independently predicted survival (odds ratio 395.8, p = 0.024). The serum levels of micronutrients in both groups were comparable and unaltered following CRRT. Regarding renal outcome, patients in the dialysis-independent group had higher serum albumin level than the dialysis-dependent group, p = 0.01.

**Conclusions** In critically ill patients requiring CRRT, DPI is a good predictor of patient survival while serum albumin is a good prognosticator of renal outcome.

**Grant acknowledgement**

Funding from the Ratchadapisek Sompoch Research Grant, Chulalongkorn University.

**Note:** This abstract has been previously published and is available at [1]. It is included here as a complete record of the abstracts from the conference.

**References**

1. Kriemetapak K, Peerepornratana S, Srisaway N, et al. (2016). The impact of macro-and micronutrients on predicting outcomes of critically ill patients requiring continuous renal replacement therapy. PloS ONE: http://dx.doi.org/10.1371/journal.pone.0156634.

### A327 Hydroxyethyl starch for volume expansion after subarachnoid haemorrhage does not impair renal function

#### T. Winkelmann^1^, T. Busch^1^, J. Meixensberger^2^, S. Bercker^1^

##### ^1^University Hospital Leipzig, Anesthesia and Intensive Care, Leipzig, Germany; ^2^University Hospital Leipzig, Neurosurgery, Leipzig, Germany

###### **Correspondence:** T. Winkelmann – University Hospital Leipzig, Anesthesia and Intensive Care, Leipzig, Germany

**Introduction** Hydroxyethylstarch (HES) was part of triple H-therapy for prophylaxis and therapy of vasospasm in patients suffering from subarachnoid haemorrhage (SAH). [1, 2] The EMA restricted the use of HES 2013 based on studies showing significant increase of renal failure in septic or critical ill patients receiving HES compared to crystalloids as fluid therapy. [3] There's still a lack of data examining the occurrence of renal insufficiency in non-septic patient groups.

**Objectives** The purpose of our study was to evaluate the effect of HES on renal function in SAH patients, who received large amounts of HES in the context of triple H therapy.

**Methods** Medical records of all patients presenting between January 2009 and December 2013 with non-traumatic SAH were analysed. Patients were divided in two groups based on the administration of HES 6 % and/or 10 % (n = 183) vs. controls receiving crystalloids for fluid therapy (n = 93).

Primary outcome was acute kidney injury (AKI) assessed using the RIFLE criteria referring to increase of serum creatinine and/or oliguria. [4]

**Results** The study groups had similar baseline characteristics except for SAPS scores, incidence of vasospasm and ICU LOS. Patients receiving HES fulfilled significantly more often sepsis criteria.

24.6 % (62/183) of patients in the HES group had AKI at any time during their ICU stay compared to 23.6 % (25/93) in controls (p = 0.679). Only few patients needed renal replacement therapy with no significant difference between groups (4.3 % control group vs. 2.2 % in HES group, p = 0.322).

**Conclusions** In non-traumatic SAH there was no association between AKI and HES therapy. The use of HES as fluid replacement therapy might be of less risk in non-septic critically ill patients.

**References**

1 Muench E, Horn P, Bauhuf C, Roth H, Philipps M, Hermann P, Quintel M, Schmiedek P, Vajkoczy P: Effects of hypervolemia and hypertension on regional cerebral blood flow, intracranial pressure, and brain tissue oxygenation after subarachnoid hemorrhage. Crit Care Med 2007.

2 Sen, J., Belli, A., Albon, H., Morgan, L., Petzold, A., & Kitchen, N: Triple-H therapy in the management of aneurysmal subarachnoid haemorrhage. Lancet Neurology 2003.

3 Mayor, S.: EMA confirms that hydroxyethyl starch solutions should not be used in critically ill, sepsis, or burns patients: BMJ (Clinical Research Ed.). 2013.

4 Bellomo R, Ronco C, Kellum JA, Mehta RL, Palevsky P, Acute Dialysis Quality Initiative: Acute renal failure - definition, outcome measures, animal models, fluid therapy and information technology needs: the Second International Consensus Conference of the Acute Dialysis Quality Initiative (ADQI) Group. Crit Care 2004.

### A328 Early and late acute kidney injury in burn patients

#### E.M. Flores Cabeza, M. Sánchez Sánchez, N. Cáceres Giménez, C. Gutierrez Melón, E. Herrero de Lucas, P. Millán Estañ, M. Hernández Bernal, A. Garcia de Lorenzo y Mateos

##### Hospital Universitario La Paz, Medicina Intensiva, Madrid, Spain

###### **Correspondence:** E.M. Flores Cabeza – Hospital Universitario La Paz, Medicina Intensiva, Madrid, Spain

**Objectives** To study the prevalence and factors related with the development of early AKI (before day 3) and late AKI (after day 3)

**Methods** 165 critical burn patients were studied over a period of 3 years. All of them were resuscitated by a resuscitation protocol guided by transpulmonary thermodilution and monitoring lactic acid levels. In resuscitation phase artificial colloids were used at low doses.The incidence of AKI (according to AKIN criteria), early AKI (defined as before day 3), recovery or progression and late AKI, and related factors with their development: comorbidities, severity scores, need for mechanical ventilation, development of shock and mortality were studied. Descriptive analysis and Kruskall-Wallis test was performed

**Results** The average total body surface area (TBSA) burned was 30 ± 15 %, mean age was 43 ± 16 years, mean ABSI score was 7.1 ± 2.1and the median of the total volume needed in the first 24 hours was 4.01 ml/kg/% .

42 patients developed AKI (stage I: 20 , II : 5, III : 17). Of these, 12 patients (28 %) developed early AKI (stage I : 7, II 1 and III : 4). There were no differences in comorbidities en no AKI, early AKI, and late AKI patients. There were differences between late AKI and no AKI patients; the late AKI patients were older (55 vs 40 p < 0.001), had more TBSA burned (27 vs 37 p < 0.001), presented worse severity scores (ABSI 6.7 vs 8.7 p < 0.001; SOFA day 0:2.1 vs 3.9 p < 0.001; SOFA day 3: 3.5 vs 6.5 p < 0.001), presented greater need for mechanical ventilation (43 % vs 80 % p < 0.001), had more prevalence of shock ( 37 vs 73 p < 0.0001), and more mortality (4.9 % vs 39.2 % p < 0.0001) than the no AKI patients. But there were no differences in the other study factors. There were no differences between early and late AKI patients nor between no AKI and early AKI patients (except for mortality 4.9 vs 25 % p < 0.008). 4 patients with early AKI returned to basal values at 7^th^ day and the other 8: 3 continued in AKIN I at 7^th^ day, 1 increased to AKIN II level and 4 to AKIN III): Only two patients (15 % of them),one alive and another dead, did not recover basal values.

**Conclusions** The patients without AKI had similar characteristics to the patients who developed early AKI but the patients who developed late AKI were older, had more TBSA burned, , worse severity scores, greater need for mechanical ventilation, presented more prevalence of shock and higher mortality. One third of the early AKI patients recovered normal values before the 7^th^ day, and most of them recover this normal values before discharge.

### 0329 HES (130/0.4) has no negative effect on kidney function and microvascular perfusion in severe acute normovolemic hemodilution in pigs

#### B. Ergin^1^, P. Guerci^1^, P.A.C. Specht^2^, Y. Ince^1^, C. Ince^1^

##### ^1^Academic Medical Center, University of Amsterdam, Translational Physiology, Amsterdam, Netherlands; ^2^Erasmus Medical Center, Experimental Anesthesiology, Roterdam, Netherlands

###### **Correspondence:** B. Ergin – Academic Medical Center, University of Amsterdam, Translational Physiology, Amsterdam, Netherlands

**Introduction** Acute normovolemic hemodilution (ANH) using artificial colloid such as starch solution is a common technique to reduce the requirements for allogeneic blood transfusion but is controversial especially concerning renal function. Not much data exists about the influence of starches in setting of hemodilution on renal function and microvascular perfusion.

**Objectives** The present study was designed to investigate the impact of ANH on renal cortex microcirculatory flow distribution and its short-term functional consequences on the systemic, renal hemodynamics and oxygenation.

**Methods** Fully instrumented 11 female Yorkshire pigs (27.5 ± 1,5 kg) were divided into two group; Hemodilution (n = 8)(ANH) and a control (Ctr) group (n = 3). Hemodilution was performed by replacing blood with HES solution (6 % Hydroxyethyl Starch 130/0.4, Voluven, Fresenius Kabi) till hematocrit levels reached 20 % (T1), 15 % (T2) and 10 % (T3). Cardiac output (CO), mean arterial pressure (MAP), central venous blood pressure (CVP), pulmonary arterial pressure (PAP), systemic vascular resistance (SVR) and heart rate (HR) were monitored.The right kidney was exposed and an flow probe (Transonic Systems Inc.) was placed around the renal artery to measure renal blood flow (RBF). Renal cortex microcirculatory flow distribution was measured by Laser Speckle Imaging (Moor Instruments, UK).)

**Fig. 128:**
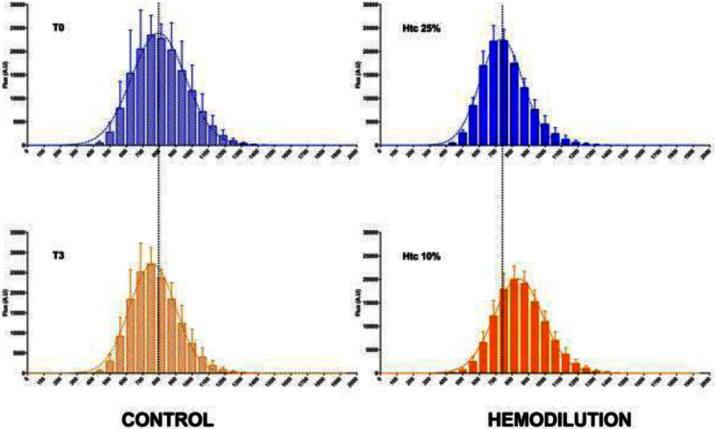
(abstract 0329).

**Fig. 129:**
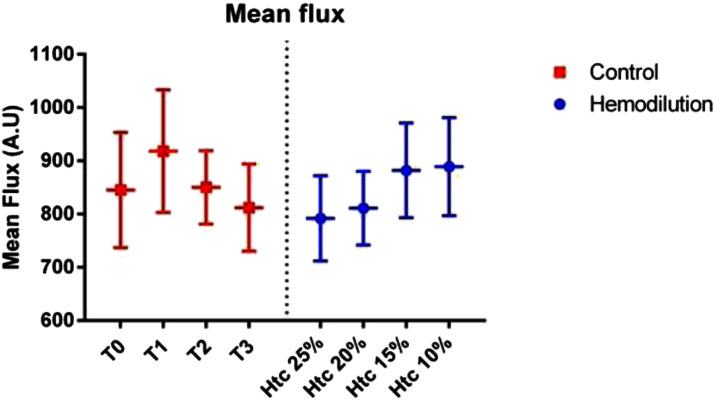
(abstract 0329).

**Results** Despite MAP and CVP being stable during the entire study, we found that while HR (106+/-18 in ANH group vs. 76+/-7 in Ctr group, p = 0.025) and CO (6.7+/-1.1 in ANH group vs. 3.9+/-0.1 in Ctr group, p = 0.002) increased, PAP (17.6+/-2.3 in ANH group vs. 22+/-4.3 in Ctr, p = 0.05) decreased significantly at Hct 10 % compared to the Ctr. Oxygen delivery (DO_2_) did not change but SVR (704+/-128 in ANH group vs. 1259+/-128 in Ctr group, p = 0.001 at T3) and oxygen consumption (VO_2_) (798+/-137 in ANH group vs. 1046+/-113 in Ctr group, p = 0.02 at T2; 842+/-218 in ANH group vs. 1142+/-78 in Ctr group, p = 0.05 at T3) were found to be lower than the Control. RBF increased at T3 ( 255+/- 55 ml/min. in ANH group and 218+/-42 ml/min. in the Ctrl group, p = 0.046) and was accompanied with slightly right shifted histograms but no significant change in the mean flux values of kidney was found (Figs. 128, 129). No differences was found in creatinine clearance, tubular sodium reabsorption and fractional sodium excretion of kidney between 10 % Hct of ANH and Ctr group at T3.

**Conclusions** This study showed that ANH using HES can cause a reduction of systemic oxygen consumption with sustained oxygen delivery being maintained by an increase CO. ANH with HES also caused an increase in RBF but did not change the microvascular perfusion and flow heterogeneity in kidney. In the present study, we demonstrated that there is no deleterious effect of HES on renal function and microvascular perfusion in the setting of severe ANH.

**Grant acknowledgment**

This study was supported by the Dutch Kidney Foundation (Innovation grant 14OIP11).

### A330 The effects of high level magnesium dialysis/substitution fluid on magnesium homeostasis under regional citrate anticoagulation in critically ill

#### M. Balik, M. Zakharchenko, F. Los, H. Brodska

##### 1st Medical Faculty, Charles University, Prague, Czech Republic

###### **Correspondence:** M. Balik – 1st Medical Faculty, Charles University, Prague, Czech Republic

**Introduction** The requirements for magnesium (Mg) supplementation increase under regional citrate anticoagulation (RCA) because citrate acts by chelation of bivalent cations within the blood circuit. The level of magnesium in commercially available fluids for continuous renal replacement therapy (CRRT) may not be sufficient to prevent hypomagnesemia.

**Objectives** To test dialysis/haemofiltration fluid with Mg concentration 1.50 mmol/l under various common settings of CRRT with RCA.

**Methods** Patients (n = 23 CVVH, n = 22 CVVHDF) on RRT dosage 2000 ml/h, blood flow (Qb) 100 ml/min, with RCA modality (4 % trisodium citrate) using calcium free fluid with 0.75 mmol/l of Mg and magnesium substitution were observed after switch to the calcium-free fluid with magnesium level of 1.50 mmol/l (n = 42) and no extra magnesium replenishment. All patients had renal indication to CRRT, were treated with the same devices, filters and the same postfilter ionized calcium endpoint (<0.4 mmol/l) of prefilter citrate dosage. Under the high level Mg fluid the Qb, dosages of citrate and CRRT were consequently escalated in 9 h steps to test various settings.

**Results** Median balance of Mg was -0.91 (-1.18 to -0.53) mmol/h with Mg 0.75 mmol/l and 0.2 (0.06-0.35) mmol/h when fluid with Mg 1.50 mmol/l was used. It was close to zero (0.02 (-0.12-0.18) mmol/h) with higher blood flow and dosage of citrate, increased again to 0.15 (-0.11-0.25) mmol/h with 3000 ml/h of high magnesium containing fluid (p < 0.001). The arterial levels of Mg were mildly increased after the change for high level magnesium containing fluid(p < 0.01).

**Conclusions** Compared to ordinary dialysis fluid the mildly hypermagnesemic fluid provided even balances and adequate levels within ordinary configurations of CRRT with RCA and without a need for extra magnesium replenishment.

**Note:** This abstract has been previously published and is available at [2]. It is included here as a complete record of the abstracts from the conference.

**References**

1. Zakharchenko M, Leden P, Rulisek J, Los F, Brodska H, Balik M: Ionized Magnesium and Regional Citrate Anticoagulation for Continuous Renal Replacement Therapy. Blood Purif 2016;41:41-47

2. Zakharchenko M, Los F, Brodska H, Balik M 92016) The effects of high level magnesium dialysis/substitution fluid on magnesium homeostasis under regional citrate anticoagulation in critically ill. PloS ONE: http://dx.doi.org/10.1371/journal.pone.0158179

**Grant acknowledgement**

Supported in part from ESICM Stoutenbeek Award 2012.

### A331 Withdrawn

### A332 Circuit lifespan of continuous veno-venous haemofiltration directly connected to extracorporeal membrane oxygenation circuit compared to central venous catheter

#### C. de Tymowski^1^, P. Augustin^1^, M. Desmard^2^, P. Montravers^1^

##### ^1^Hopital Bichat Claude Bernard, Anaesthesiology and Surgical Critical Care, Paris, France; ^2^Centre Hospitalier Sud Francilien, Critical Care Medecine, Corbeil-Essonnes, France

###### **Correspondence:** C. de Tymowski – Hopital Bichat Claude Bernard, Anaesthesiology and Surgical Critical Care, Paris, France

**Introduction** Undetected decrease of blood flow is a known factor for decreasing circuit lifespan of continuous veno-venous haemofiltration^1^ (CVVH).The connection of CVVH lines to a circuit of extracorporeal membrane oxygenation (ECMO) may allow for a more stable blood flow and a possible increase of circuit lifespan compared to a conventional CVVH realized using a central venous catheter (CVC)^2^.

**Objectives** To assess lifespan of CVVH connected to ECMO circuit compared to conventional CVVH using central venous catheter.

**Methods** In this single-center prospective study, all patients requiring CVVH for acute kidney injury were eligible for inclusion. A group of patients without ECMO receiving CVVH through CVC (C group) was compared to patients receiving ECMO (E group) in whom CVVH lines were connected to the ECMO circuit. The circuit lifespan was measured from its connection to the session discontinuation. Reasons for ending CVVH were classified as follows: effective session; primary clotting of the circuit; unsuitable inflow or outflow line pressure; not CVVH-related end of session (NRCES). All sessions for each patient were recorded and included. Data related to CVVH were recorded at the time of initiation of CVVH (H0), then after 6(H6), 12 (H12), 24 (H24), 48 (H48), and 72 hours (H72). Primary endpoint was lifespan of the circuit. Results are presented as median and interquartile range (IQR) [25-75], and absolute numbers or proportions. Statistical analysis used Mann-Whitney U test and two way independent ANOVA for comparison of multiple measures at different times. The time-to-event of both groups were estimated using Kaplan Meier analysis and log-rank test.

**Fig. 131 (abstract A332).**
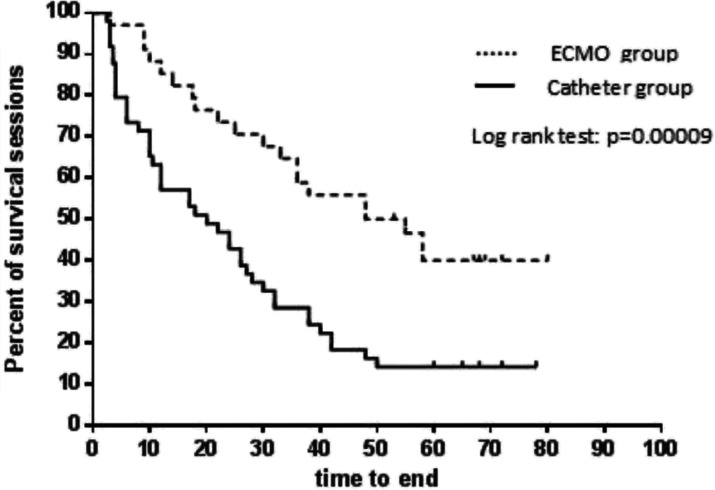
Kaplan-Meier of CVVH circuit survival

**Fig. 132 (abstract A332).**
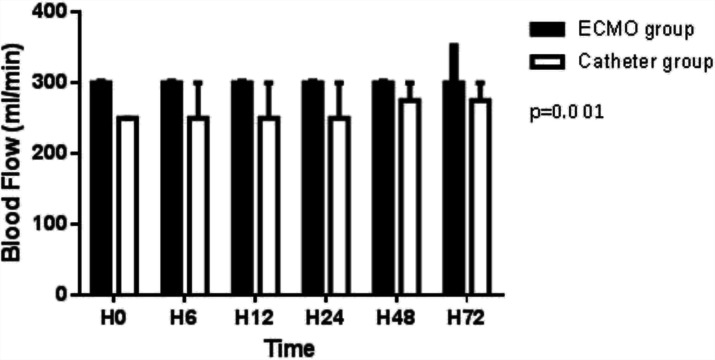
Effective blood flow during CVVH (median with IQ)

**Results** Between January 2014 and May 2015, 17 patients were included in each group, 43 sessions of CVVH were recorded in the E group and 56 in the C group. 16 sessions were interrupted prematurely for NRCES leaving 34 and 49 episodes (respectively E and C group) for analyzing circuit lifespan. Circuit lifespan was statistically increased in the E group compared to the C group. Median lifespans of CVVH circuit were respectively 48 H [21-72] vs 20 H [6-39] in E and C groups (relative risk of session ending: 2.4, 95 % CI [1.41-3.9], log rank p = 0.0009 (Fig. 130). CVVH blood flow was higher and more stable throughout sessions in the E group (p < 0.001) (Fig. 131). Median heparin dose used for anticoagulation was higher in the C group (p < 0.001) (Fig. 132) but without effect on the activated partial thromboplastin time (aPTT) all over the session.

**Conclusions** In our experience, connection of CVVH lines to ECMO is associated with an increased lifespan of the CVVH circuit, the direct connection of CVVH circuit to ECMO allowing higher blood flow.

**References**

*1 ICM* 2004;**30:** 2074-2079

*2 CC & Resuscitation* 2014;16: 127-130

**Fig. 133 (abstract A332).**
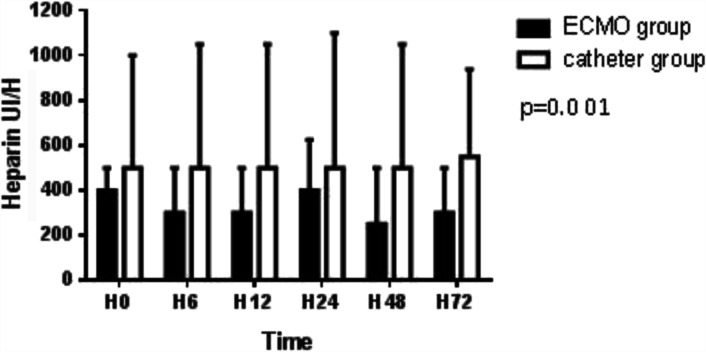
Heparin doses during CVVH (median with IQ)

### A333 Amino acid loss during CVVH in critically ill patients

#### S.N. Stapel, R. de Boer, H.M. Oudemans

##### VU University Medical Center, Intensive Care, Amsterdam, Netherlands

###### **Correspondence:** S.N. Stapel – VU University Medical Center, Intensive Care, Amsterdam, Netherlands

**Introduction** Continuous venovenous hemofiltration (CVVH) is used for renal replacement in critically ill patients with acute kidney injury (AKI). Unfortunately, substrate removal is aselective. Previous studies have shown a substantial loss of amino acids (AA) in the ultrafiltrate. However, none of the studies investigated the loss of amino acids due to adsorption to the filter membrane.

**Objectives** To quantify the total loss of amino acids during predilution CVVH in critically ill patients, including the loss by ultrafiltration (UF) and adsorption (Ads); and secondly, to determine the sieving coefficients of the different amino acids.

**Methods** Prospective observational study in 8 critically ill patients with AKI receiving predilution CVVH, blood flow180 ml/min. Predilution flow of 2400 ml/h, Fresenius polysulfone membrane (Ultraflux AV). Amino acids were measured in arterial plasma, post-filter plasma and ultrafiltrate at baseline, 1-hour, 8-hours and 24-hours after start of a new CVVH session, and were measured by high performance liquid chromatography (HPLC). Calculations:

- Total AA loss: AA _filter in_ - AA _filter out_ (g/hour)
- Loss by ultrafiltration (UF): [AA]_UF_ (g/L) * Q_UF_ (L/h)
- Loss by adsorption (Ads): Total loss - Loss by UF
- Sieving coefficient: [AA]_UF_/{0.5 ([AA]_pre-filter_ + [AA]_post-filter_

**Results** Mean age was 57 (±18) years, APACHE II score 27 (±6); Nutrition was enteral in 6 patients, parenteral in 1; 1 patient did not receive nutrition (shock). Total AA loss (mg/h), AA loss by ultrafiltration (mg/h), and AA loss by adsorption (mg/h) at 1-h, 8-h, 24-h after start of CVVH, are shown in Fig. 133. Patient nr 8, with acute ischemic liver failure, had the greatest loss of amino acids. The total amino acid loss (p = 0.276), loss by ultrafiltration (p = 0.876) and loss by adsorption (p = 0.368) did not change during the course of CVVH. The estimated median amino acid losses (g/day) are shown in Table 103.

The sieving coefficients of most amino acids were near 1. Sieving coefficients of glutamic acid (0.60), taurine (0.83), tryptophan (0.84) and ornithine (0.85) were lower.

**Conclusion** During CVVH with a modern polysulfone membrane, the estimated amino acid loss is 22.0 g/day. Total loss consists of loss by ultrafiltration for 56 % and of loss by adsorption for 44 %. Loss by UF and adsorption is stable during the first 24-h. Amino Acid loss is higher than reported previously, because loss by adsorption was not measured before.

**Fig. 134 (abstract A333).**
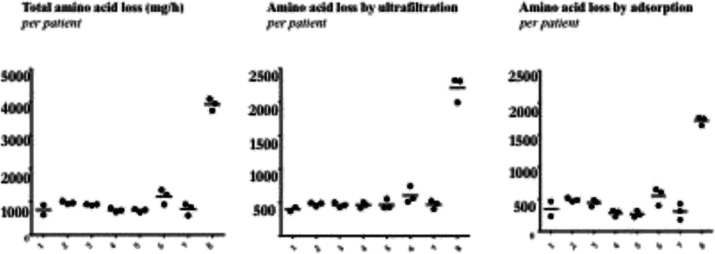
amino acid losses per patient (mg/h)]

**Table 103 (abstract A333).** Estimated total amino acid loss (g/day)]

Amino acid loss	Median (IQR)
Total	22 (18.3 - 23.8)
by Ultrafiltration	11.8 (10.3 - 12.9)
by Adsorption	10.3 (7.4 - 12.2)

### A334 Impact of type and amount of fluid for circulatory resuscitation on patients requiring renal replacement therapy: a single-centred retrospective 5-year observation

#### A. Hollinger^1^, T. Schweingruber^1^, F. Jockers^1^, M. Dickenmann^2^, M. Siegemund^1,3^, Clinical Intensive Care Research Basel

##### ^1^University Hospital Basel, Anaesthesia & Intensive Care: Surgical Intensive Care, Basel, Switzerland; ^2^University Hospital Basel, Nephrology & Transplant Immunology, Basel, Switzerland; ^3^University Hospital Basel, Department of Clinical Research, Basel, Switzerland

###### **Correspondence:** M. Siegemund – University Hospital Basel, Anaesthesia & Intensive Care: Surgical Intensive Care, Basel, Switzerland

**Introduction** Volume resuscitation is the mainstay of treatment in most types of shock, especially in hypovolemic, hemorrhagic and septic shock [1, 2]. Although there has been an increasing amount of research within the last decade in order to evaluate the optimal amount and composition of fluids for volume resuscitation (e.g. colloids, crystalloids, red blood cell transfusion, albumin, fresh frozen plasma), results remain inconclusive and to some extent contradictive.

**Objectives** To evaluate the impact of amount and type of fluids administered on the use of renal replacement therapy (RRT) and outcome.

**Methods** Single-centred retrospective observational study analysing patient charts on the medical and surgical ICU of the University Hospital Basel from 2011 until 2015.

**Results** We retrospectively analysed type and amount of fluid administered within the first 72 hours after ICU admission to patients receiving continuous veno-venous hemodiafiltration (RRT) or hemodialysis in the surgical or medical ICU at Basel University hospital during a 5-year period. A total of 343 ICU patients underwent RRT from 1st January 2011 to 31st December 2015 (median duration of 3 days, interquartile range (IQR) 1-7 days).

We found a significant difference comparing patient survival and patient death among age (p < 0.001), SAPS II score, total volume within the first 72 hours on the ICU, fluid balance in the second and third 24 h (p = 0.001 and p = 0.006) and also in balance at discharge (p < 0.001).

Out of 343 patients, 189 (55.1 %) died. The annual mortality varies from 65.6 % in 2011, 44.8 % in 2013 to 54.9 % in 2015 (average 55.1 %, p(2011/2013) = 0.169). The number of RRT in the ICU did not change during the study period. HES was administered to 75 patients (21.9 %), the number of HES-receiving patients decreased from 64.1 % in 2011 to 6 % in 2013 and finally to 0 patients in 2015. Patients who were given HES received more intravenous fluids within the first 72 hours (p < 0.001), more noradrenaline (p = 0.033) and had significant higher fluid balances at all measured time-points. The mortality of patients without HES administration was lower than those who received HES (51.2 % vs. 69.3 %, p = 0.005, Phi/Cramer V = 0.151).

**Conclusions** Contrary to our expectations, the rate of renal replacement therapy did not decrease over the last 5 years despite abandoning administration of HES solution. Nevertheless, the administration of HES was associated with a higher mortality. Additional high fluid load and a positive balance are highly associated with mortality. Further research is needed to evaluate if the relationship between high mortality and higher amount of administered fluids is based on correlation or causation.

**References**

1. Rochwerg, B. et al. Intensive Care Med. 2015;41(9):1561-71

2. Haase N, et al. BMJ. 2013 15;346:f839.

**Table 104 (abstract A334).** Patient characteristics per Year

	2011	2012	2013	2014	2015
Number	64 (48 - 16)	72 (43 - 29)	67 (43 - 24)	69 (48 - 21)	71 (41 - 30)
SAPS-II	71 (62 - 78)	69 (59 - 82.8)	73 (58 - 86)	81 (65 - 87)	73 (56 - 86)
HES used n(%)	41 (64)	29 (40)	4 (6)	1 (1.5)	0 (0)
Mortality n (%)	42 (66)	41 (57)	30 (45)	37 (54)	39 (55)
ICU LOS [d]	6 (3 - 14.8)	12 (3 - 21)	7 (3 - 13)	9 (3 - 19.5)	7 (3 - 14)
RRT length [d]	2.5 (1 - 7.8)	1 (3.5 - 8.8	3 (1 - 6)	4 (2 - 7.5)	3 (1 - 8)
Total fluids in 72h [L]	14.2 (7.2 - 22)	13.1 (5.5 - 24.5)	13.9 (5.2 - 26.2)	14.7 (6.1 - 23.4)	8.8 (3.3 - 16)
∑ Noradrenaline in 72h [mg]	27.5 (6.3 - 79.3)	26.3 (7.8 - 61.4)	12.0 (1.0 - 61.2)	12.1 (1.9 - 45.2)	9.0 (0.07 - 29.9)
Dialysis on discharge n (%)	3 (4.7)	10 (4.2)	5 (4.5)	11 (4.4)	4 (4.2)

**Table 105 (abstract A334).** Impact on survival

	Survivours	Dead	p-Value
	Median (IQR)	Median (IQR)	Unpaired t-Test & Pearson-Chi^2^
Number (m/f)	154 (99/55)	189 (124/65)	
SAPS-II Score	69 (55.75 - 79)	77 (64 - 88)	<0.001
Length of RRT [d]	4 (2 - 8)	2 (1 - 6)	
∑ Volume in 72h [L]	10.4 (4.4 - 19.8)	14.6 (8.1 - 24.6)	<0.001
HES received n (%)	23 (14.9)	52 (27.5)	0.005
∑ Ec, FFP, Tc in 72h [L]	0.6 (0 - 1.8)	1.2 (0.3 - 4.6)	<0.001
∑ Noradrenaline in 72h [mg]	7.4 (0.05 - 38.4)	26.4 (6.6 - 55.4)	0.036
Balance first 24h [L]	2.6 (0.9 - 5.8)	4.4 (2.0 - 8.6)	0.001
Balance at 72h [L]	9.7 (4.2 - 17.0)	12.5 (6.9 - 21.1)	<0.001
Exclusion LOS <72h	(13 survival)	(56 death)	

### A335 Continuous renal replacement therapy prescription and dosing in intensive care patients at the Queen Elizabeth University Hospital Glasgow

#### N. Runciman, M. Ralston, R. Appleton

##### Queen Elizabeth University Hospital, NHS GG&C, Glasgow, United Kingdom

###### **Correspondence:** N. Runciman – Queen Elizabeth University Hospital, NHS GG&C, Glasgow, United Kingdom

**Introduction** Current continuous renal replacement therapy (CRRT) guidelines recommend delivery of a dose of 20-25 ml/kg/hr ^1^. Previous studies have shown a tendency to higher intensity dosing than required ^2^. The new Queen Elizabeth University Hospital Glasgow (QEUHG) merged three intensive care units (ICUs), and practice at the new unit had not yet been evaluated.

**Objectives** To determine the delivered dose of CRRT to patients in the ICU, the number and length of breaks in delivery, and the choice of filtration fluid.

**Method** 79 patients received CRRT in the ICU at the QEUHG between May and December 2015. A sample of 29 patients was examined. Effluent doses were collected from the electronic patient record (EPR), normalised by the patients' weights and by time on CRRT. Prescriptions, interruptions in delivery of CRRT (including noted reasons for the break) and the choice of filtration fluid were also retrieved.

**Result** Patients received 3333 hours of CRRT, or 158 24-hour periods (8 am-7 am). Daily prescriptions were completed in 42 % of cases. Prescriptions were completed in 56 % of cases in which CRRT was required prior to review on a ward round. The median prescribed dose was 25.5 ml/kg/hr. 29 % of days saw the recommended dose delivered for patients. On 32 % of days studied, patients received a dose lower than 20 ml/kg/hr, while on another 39 % of days, patients received greater than 25 ml/kg/hr. The median delivered dose was 24.3 ml/kg/hr. Greater variation in prescription and delivery were noted in the first 3 days of CRRT. Breaks in CRRT constituted 15 % of total hours examined. The most frequent reason was for a clotted filter. The average filter lifespan was 18.6 hours, with 2.7 % lasting the maximum 72 hours. Prismasol 4 % filtration fluid was used for 94.3 % of the hours spent on CRRT.

**Conclusions** Rates of daily prescription are poor, at 42 %. Completed prescriptions show reasonable adherence to recommended dosages. This suggests there may be a role for prompting to complete/reassess prescriptions within the EPR. Only 29 % of days saw the correct dose of CRRT delivered. It may be that the breaks in CRRT are not adjusted for in the prescribed dose, leading to under-dosing. Higher prescription, in the context of anticipated disruption, may reduce under-dosing. However, this would worsen cases of higher-intensity delivery. Further work is required to understand the reasons for high-intensity prescription and delivery. Improved anticoagulation may reduce breaks in CRRT delivery, and improve filter lifespan. This could reduce workload, cut costs, and potentially improve patient outcomes.

**References**

1. Ostermann M, Forni L. Acute Renal Replacement Therapy. In: Guidelines for Provision of Intensive Care Services FICM/ICS; 2015. p. 81-4.

2. Black E, Chalmers J, Wallis C, Cole S, on behalf of the SICS Trainees Audit Share Group. Renal replacement therapy in Scottish critical care units: A national audit of practices. J Intensive Care Soc. 2015 Feb 1;16(1):45-51.

**Table 106 (abstract A335).** CRRT Prescription in First Week

Day	Median Dose (ml/kg/hr)	Range [IQR]
1	25	25-60 [25-25]
2	25	25-67 [23-30]
3	30	25-40 [25-36]
4	25	25-35 [25-25]
5	25	25-25 [25-25]
6	25	25-25 [25-25]
7	25	25-25 [25-25]

## NONINVASIVE VENTILATION

### A336 Correlation between improvement of physiological variables and increasing flow rates during High Flow Nasal Cannula (HFNC) therapy

#### T. Mauri^1,2^, L. Alban^1,3^, C. Turrini^1,3^, T. Sasso^1^, T. Langer^1^, M. Panigada^1^, P. Taccone^1^, E. Carlesso^1^, C. Marenghi^1^, G. Grasselli^1,2^, A. Pesenti^1,2,4^

##### ^1^Fondazione IRCCS Ca' Granda Ospedale Maggiore Policlinico, Milano, Italy; ^2^Plug Working Group, ESICM, Milano, Italy; ^3^Sant'Anna Hospital, University of Ferrara, Ferrara, Italy; ^4^University of Milan, Milano, Italy

###### **Correspondence:** L. Alban – Fondazione IRCCS Ca' Granda Ospedale Maggiore Policlinico, Milano, Italy

**Introduction** High Flow Nasal Cannula (HFNC) is a non-invasive respiratory support that positively modifies clinical outcomes of hypoxemic Acute Respiratory Failure (ARF) patients [1]. Available data suggest that HFNC support induces a number of physiological benefits over conventional oxygen therapy, however little is known about the correlation between physiological effects of HFNC and flow rates.

**Objective** Aim of this study was to disclose whether physiological effects of HFNC linearly or non-linearly follow increased flow rates.

**Methods** We performed a prospective randomized cross-over study on 10 hypoxemic ARF patients with PaO_2_/FiO_2_ ≤ 300 mmHg while on non-invasive oxygen support. FiO_2_ was set to obtain SpO_2_ of 90-95 % by facial mask and left unchanged throughout the study. Patients underwent four randomized steps, lasting 20 minutes each: facial mask (gas flow 12 L/min) vs. HFNC at 30, 45 and 60 L/min. During all phases we assessed gas exchange, lung volumes by electrical impedance tomography and inspiratory effort by esophageal pressure. Data measured during the 4 steps were compared using one-way ANOVA while best fitting of linear vs. non-linear correlations was assessed by lower Akaike´s information criterion (AIC) value.

**Results** Patients were 61 ± 10 year-old, five were female and PaO_2_/FiO_2_ at enrollment was 171 ± 44 mmHg. At higher flow rates (Table 107): PaO_2_ improved (p < 0.001) while pH and PaCO_2_ didn't vary; end-expiratory lung volume (ΔEELV) increased (p < 0.05) and peak expiratory flow (PEF) decreased (<0.05) suggesting positive expiratory pressure effect by increased expiratory resistance and/or improved respiratory system compliance; respiratory rate (RR) decreased (p < 0.001) and tidal volume (Vt) didn't change (p > 0.05), anyway yielding decreased minute ventilation (MV) (p < 0.01) and corrected minute ventilation (MV_corr_ = MV*PaCO_2_/40 mmHg) (p < 0.01), indicating enhanced CO_2_ removal; finally, esophageal pressure swing (ΔPes) and pressure-time product (PTPes) decreased (p < 0.01 for both). AIC for linear correlation with flow rates was lower for PaO_2_, RR, ΔEELV and PEF, as if improved aeration and its effects on oxygenation constantly increase with flow; while non-linear AIC was lower for MV, MV_corr_, ΔPes and PTPes, possibly suggesting that most of the improvement in CO_2_ wash-out from upper airway dead space and its consequences on patient's effort is already obtained at 30 L/min.

**Conclusions** HFNC induces multiple beneficial physiologic effects that might delay respiratory decompensation and improve clinical outcomes. However, not all effects linearly increase with HFNC flow so that its selection might be personalized to target the most clinically relevant physiologic derangement.

**References**

1. Papazian et al., Intensive Care Med, 2016.

**Grant acknowledgment**

Departmental.

**Table 107 (abstract A336).** Effects of HFNC on gas exchange

	Facial mask (12 L/min)	HFNC (30 L/min)	HFNC (45 L/min)	HFNC (60 L/min)	P-value (ANOVA)	Fitting (AIC) Linear	Fitting (AIC) Non-linear
PaO2 (mmHg)	72.8±13.5	86.7±14.4*	92.4±11.3*	95.9±13.9*	<0.001	228.1	231.2
PaCO2 (mmHg)	38.9±5.6	39.2±6.4	39.1±6.6	38.9±6.3	n.s.	-	-
pH	7.47±0.1	7.47±0.1	7.47±0.1	7.48±0.1	n.s.	-	-

* p<0.05 vs. facial mask by post-hoc Bonferroni test; no other between-phases post-hoc comparison was significant

**Table 108 (abstract A336).** Effects of HFNC on ventilation pattern

	Facial mask (12 L/min)	HFNC (30 L/min)	HFNC (45 L/min	HFNC (60 L/min)	P-value (ANOVA)	Fitting (AIC) Linear	Fitting (AIC) Non-linear
RR (bpm)	21±5	19±4	17±5*	16±5*	<0.001	204.7	206.9
Vt (mL)	499±371	481±376	487±377	482±368	n.s.	-	-
MV (L/min)	9.5±4.1	7.0±2.5*	7.5±3.2*	7.2±2.1*	<0.01	228.2	225.7
MV corr (L/min)	9.2±4.5	6.8±2.7*	7.2±3.4	6.9±2.4*	<0.01	235.9	234.5
ΔEELV (mL)	baseline	81±228	119±178	212±284*	<0.05	425.2	428.1
PEF (L/min)	40.7±23.9	34.8±21.1*	36.7±21.3	36.6±17.6	<0.05	225.1	226.4
ΔPes (cmH2O)	10.0±4.1	8.0±2.9*	7.7±2.9*	7.2±2.3*	<0.01	203.9	202.8
PTPes (cmH2O*s/min)	228±74	178±55*	152±41*	143±44*	<0.001	235.9	202.3

* p<0.05 vs. facial mask by post-hoc Bonferroni test; no other between-phases post-hoc comparison was significant

### A337 Contribution of mechanical in-exsufflation (MI-E) device in preventing post-extubation respiratory failure in patient with critical illness polyneuromyopathy (CIP) – neuromie

#### P. Wibart^1^, T. Reginault^1^, M. Garcia^1^, B. Barbrel^1^, A. Benard^2^, C. Bader^2^, F. Vargas^1^, H.N. Bui^1^, G. Hilbert^1^

##### ^1^CHU Bordeaux, Réanimation Médicale, Bordeaux, France; ^2^CHU Bordeaux, Unité d'Épidemiologie Clinique, Bordeaux, France

###### **Correspondence:** P. Wibart – CHU Bordeaux, Réanimation Médicale, Bordeaux, France

**Introduction** CIP appears frequently in ICU and can occur in up to 25 % of patients. After extubation CIP causes poor airway clearance due to respiratory muscle weakness and can lead to respiratory failure and reintubation. Reintubation, which increases severity of illness, is an independent risk of nosocomial pneumonia, increased hospital stay and mortality. Currently, standard treatment includes respiratory physiotherapy with manual assisted cough. However respiratory failures still occurs in 30 % of patients within 48 hours after planned extubation. Though we conducted a study evaluating, in patients with CIP, the efficiency of MI-E device in the prevention of respiratory failure during 48 hours after extubation. MI-E is a non-invasive technic to assist respiratory physiotherapy which aims to suction tracheal mucus. MI-E has been evaluated for neuromuscular disease patients, and it has been shown to increase peak expiratory flow and to improve airway clearance.

**Objectives**

**Primary outcome:** Incidence of respiratory failure after extubation

**Secondary outcomes:**

- reintubation rate
- mean length of stay in ICU
- mortality at day 28

**Methods** In a medical ICU of a university hospital we conducted a prospective randomized open study in two parallel groups. All intubated patients were screened with a Medical Research Council (MRC) muscular test. Patients were diagnosed CIP if the MRC score was equal or under 48/60. All patients with CIP and without exclusion criteria were randomized in the study. During the treatment period, patients received two daily session of MI-E plus manually assisted coughing or standard treatment (manually assisted coughing).

**Results** 123 patients were included in three years, 62 in the standard group, and 61 in the MI-E group. There was no difference between the two groups at baseline. The results show no difference for the primary outcome (p 0,6022): 7/62 respiratory failures in the standard group (11,5 %), 10/61 in the MI-E group (16,4 %). For the secondary outcomes, we found no statistical difference concerning reintubation (3.6 % vs 7 %), mean length of stay in ICU (6,5 vs 7,9 days) and mortality at day 28 (14.8 % vs 18.3 %)

**Conclusions** The study demonstrated no superiority of the MI-E device in the prevention of post-extubation respiratory failure for ICU patients with CIP.

**References**

1. Bach JR, Smith WH, Michaels J et al. Airwaiy secretion clearance by mechanical exsufflation for post poliomyelistis ventilator assisted individuals.Arch Phys Med Rehabil 1993; 74: 170-6

2. De Jonghe B, Sharshar T, Lefaucheur JP, et al. Paresis acquired in the intensive care unit: a prospective multicenter study. 2002;288: 2859-67.

### A338 Spectral analysis of noise intensity from various devices and assistance for non-invasive respiratory support

#### J.M. Serrano Simón^1^, P. Carmona Sánchez^1^, F. Ruiz Ferrón^2^

##### ^1^Hospital Universitario Reina Sofia, Intensive Care Unit, Córdoba, Spain; ^2^Complejo Hospitalario de Jaén, Intensive Care Unit, Jaén, Spain

###### **Correspondence:** J.M. Serrano Simón – Hospital Universitario Reina Sofia, Intensive Care Unit, Córdoba, Spain

**Introduction** The noise is associated with morbidity and mortality in critically ill patients, related to sleep fragmentation and delirium primarily (1-3). The ventilation devices can generate noise, with further impact on the effectiveness of the technique. An example is the non-invasive CPAP devices and high nasal flow, widely used in ICU (4).

**Objectives** To evaluate the intensity of the noise level as perceived by the patient and to compare the difference in noise intensity between several different devices for non-invasive respiratory support at different levels of assistance.

**Methods** Made in patients with acute respiratory failure, and pulmonary dummy-simulator with spontaneous breathing, were connected to differents device CPAP with 5, 10, 12.5, and 15cmH2O, as high flow system 35-110 L/min. We studied 3 models of noninvasive CPAP (Wisperflow/Vital Signs; Ventumask® HF, Starmed, Intersurgical; valve-Boussignac®, Vigon); device of high flow: Optiflow®-MR850 with Whisperflow high flow generator, and Airvo2® Fisher & Paykel. The flow is measured with Ohmeda 5410 Volume Monitor. The intensity of sound is measured at ear level using Speedlink SL-8691-SBK-01 microphones. The audio signal is recorded for 60 seconds in wav format (22,050 KHz). Three measurements were performed for each level of assistance and devices. The noise intensity was obtained from the spectral power (PSD) of sound pressure waves, hanning windows and fast Fourier transform was used. PSD is reported in decibels sound pressure level (dB SPL)/KHz, where: dB SPL = 20 log(P/P0), P is intensity of the stimulus, and P0 the reference's intensity at the threshold of human hearing at the frequency of 1 kHz. Analysis was perfomed with software Matlab R2008a of the maximum and mean (SD) spectral power/frequency of each signal. The comparisons are performed by analysis of variance for multiple samples.

**Results** The table below shows the measured noise intensity levels from the devices and support leves studied. The figure shows the power spectral (dB/KHz) of signals at level representative, from devices CPAP with 12.5-15 cmH2O and high nasal flow. Boussignac CPAP and HF Ventumask produced the highest levels of noise: 2.3 to -3.8 dB/KHz at the high CPAP levels. The lowest noise levels are produced by high flow nasal Airvo2 35 L/min (dB/KHz max: -48). The noise intensity increasing in proportion to the flow.

**Conclusions** Our results suggest that the devices studied are the noisiest valve-Boussignac and Ventumask. The noise intensity increases in proportion to the flow, regardless of the level of applied pressure. The less noisy device: Airvo2 35 L/min. The noise Optiflow-MR850 can be relative to the flow generator used.

**References**

1) Figueroa-Ramos MI, ICM 2009; 35(5):781.

2) Milbrandt EB, CCM 2004; 32(4):955.

3) E. Wesley Ely, JAMA 2004; 291(14):1753.

4) König K, Neonatology 2013;103:264.

**Table 109:**
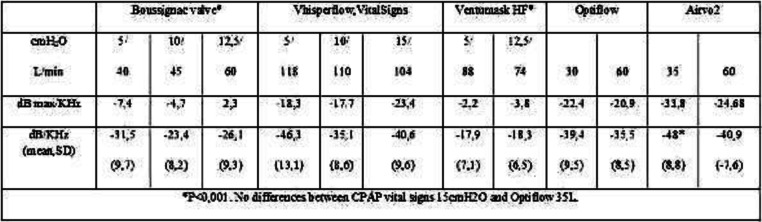
(abstract A338).

**Fig. 135:**
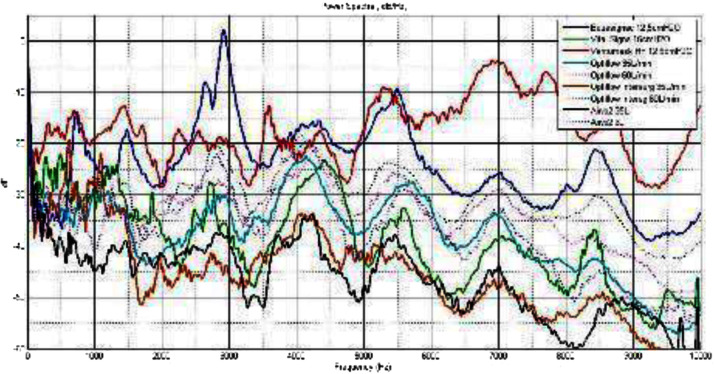
(abstract A338).

### A339 Can hypoxemic patients with bilateral infiltrates treated with high flow nasal cannula be considered as acute respiratory distress syndrome? An inflammation study

#### M. García de Acilu^1,2^, J. Marin^3^, V. Antonia^3^, L. Ruano^1^, M. Monica^4^, R. Ferrer^1,5^, J.R. Masclans^3,5^, O. Roca^1,5^

##### ^1^Vall d'Hebron University Hospital, Critical Care Department, Barcelona, Spain; ^2^Universitat Autònoma de Barcelona, Medicine, Barcelona, Spain; ^3^Parc de Salut Mar. IMIM (Mar Medical Research Institut), Critical Care Department, Barcelona, Spain; ^4^Joan XXIII University Hospital, Critical Care Department, Tarragona, Spain; ^5^Instituto de Salud Carlos III, Ciber Enfermedades Respiratorias (Ciberes), Madrid, Spain

###### **Correspondence:** M. García de Acilu – Vall d'Hebron University Hospital, Critical Care Department, Barcelona, Spain

**Introduction** High flow nasal cannula (HFNC) is emerging as a supportive therapy for patients with acute respiratory failure. However, according to the Berlin definition, it is unclear whether patients with acute hypoxemia (PaO2/FIO2 ≤ 300) and bilateral opacities not fully explained by cardiac failure and who are treated with HFNC should be considered as acute respiratory distress syndrome (ARDS) patients.

**Objectives** To examine whether HFNC patients with acute hypoxemia and bilateral opacities treated with HFNC and ARDS patients who were directly mechanically ventilated are similar in terms of lung epithelial, endothelial and inflammatory biomarkers.

**Methods** Prospective multicenter study at three university tertiary hospitals enrolling intubated and non-intubated patients admitted to the ICU with acute hypoxemia (PaO_2_/F_I_O_2_ ≤ 300) and bilateral opacities. HFNC or mechanical ventilation was initiated at the discretion of the attending physician. We measured plasma biomarkers of lung epithelial (receptor for advanced glycation end-products [RAGE] and surfactant protein D [SP-D]) and endothelial (Angiopoietin-2 [ANG-2]) injury and inflammation (interleukin [IL] 6, IL8, IL33 and soluble suppression of tumorigenicity-2 [sST2]) within the first 24 hours of ARDS onset. A propensity score matching was performed using 5 different variables (APACHE II, SOFA, PaO2/FIO2, origin of ARDS and need for vasopressors). Non-hypoxemic mechanically ventilated critically ill patients and healthy volunteers served as the controls.

**Results** Of the 102 enrolled patients, 71 (69.6 %) were intubated and 31 (30.4 %) were treated with HFNC at ARDS onset. Patients initially treated with HFNC had lower APACHE II (20 [14-23] vs 21 [19-24]; p = < 0.01) and SOFA score (6 [4-8] vs 7 [5-10]; p = < 0.01) and were less likely to develop shock during their ICU stay (48.4 % vs 83.1 %; p = < 0.01). After propensity score matching (23 HNFC patients vs 23 MV patients), no significant differences were observed in RAGE, SP-D, ANG-2, IL6, IL8, IL33 and ST2 between matched patients who were treated with HFNC at ARDS onset and those who were intubated.

After matching, no differences in mortality or length of stay were observed. All biomarkers with the exception of IL33 were higher in both groups of matched ARDS patients than in both control groups.

**Conclusions** Acute hypoxemic patients with bilateral infiltrates treated with HFNC present a similar pattern of biomarkers of inflammation and injury compared with those ARDS patients who were directly mechanically ventilated. The results suggest that these HFNC patients may be considered as ARDS patients.

**Grant acknowledgment**

This study was supported by grants from Instituto de Salud Carlos III-FEDER, (PI14/01420) and Spanish Foundation of Critical Patient (feec 2015). Fisher & Paykel support a post-doctoral fellow in Medical Research at the Hospital del Mar Institute. OR and JRM have received travel expenses from Fisher & Paykel.

**Table 110 (abstract A339).** Biomarkers at day 1 (median [IQR])

	HFNC (n=23)	MV (n=23)	P value
RAGE (ρg/ml)	2164.57 (1000.46-3394.40)	1386.35 (972.75-2180.29)	0.18
SP-D (ηg/ml)	13.70 (5.85-18.95)	12.25 (4.11-19.19)	0.51
ANG-2 (ρg/ml)	7215.00 (3712.92-10875.42)	10243.75 (5978.54-15510.83)	0.17
sST2 (ρg/ml)	1506.87 (1130.77-3691.53)	1399.89 (1075.22-1837.99)	0.61
IL33 (ηg/ml)	0.87 (0.77-1.59)	0.94 (0.85-1.38)	0.45
IL-8 (ρg/ml)	37.29 (22.46-143.39)	83.68 (33.48-145.02)	0.28
IL-6 (ηg/l)	111.63 (84.29-135.73)	74.41 (52.61-113.93)	0.05

### A340 Usefulness of high-flow nasal cannula oxygen therapy in patients with acute respiratory failure undergoing bronchoalveloar lavage

#### G. Hong, D.H. Kim, Y.S. Kim, J.S. Park, Y.K. Jee

##### Dankook University Hospital, Dankook University College of Medicine, Department of Internal Medicine, Cheonan, Republic of Korea

###### **Correspondence:** G. Hong – Dankook University Hospital, Dankook University College of Medicine, Department of Internal Medicine, Cheonan, Republic of Korea

**Introduction** Critically ill patients with acute respiratory failure (ARF) undergoing bronchoscopy have an increased risk of hypoxemia-related complications. Previous studies have shown that in awake, hypoxemic patients non-invasive ventilation is helpful in preventing hypoxemia during bronchoscopic procedure. Recently, high-flow nasal cannula (HFNC) therapy has been used to improve oxygenation in patients with ARF. However, there are minimal data evaluating the use of high flow nasal cannula undergoing bronchoscopy with bronchoalveolar lavage (BAL).

**Objectives** This study investigated the feasibility and safety of high-flow nasal cannula (HFNC) oxygen therapy for ARF in adult patients undergoing bronchoscopy with BAL.

**Methods** We identified 19 patients with suspected pneumonia and hypoxemic ARF (PaO2/FiO2 ≤ 300) who were admitted to a medical intensive care unit and who received HFNC therapy undergoing bronchoscopy with BAL between May 2014 and March 2016 at Dankook University Hospital. Their medical records were reviewed retrospectively.

**Results** The subjects included 13 men and 6 women, mean age 61 years (range 23-86 y).

Of the 19 patients, 18 (94.7 %) successfully complete BAL on HFNC. Only 1 patient required endotracheal intubation during BAL. Two patients required endotracheal intubation with mechanical ventilation 3 and 5 hours after BAL, respectively.

The etiologies of acute respiratory failure were bacterial pneumonia (8, 42.1 %), Influenza pneumonia (2, 10.5 %), pneumonia due to undefined pathogen (7, 36.8 %), and diffuse alveolar hemorrhage (1, 5.3 %), acute exacerbation of idiopathic pulmonary fibrosis (1, 5.3 %). HFNC was initiated at a mean FiO2 of 0.56 (range 0.30 - 0.9) and flow of 44.3 L/min(range 30 - 50 L/min). Mean PaO2/FiO2 at baseline was 168 (range 77 - 260) mm Hg and at the end of procedure was 157.8 (range 54.5 - 266).

Changes in mean blood pressure, heart rate, respiratory rate and PaO2/FiO2 values induced by the procedure did not reach significance (P > 0.05).

**Conclusions** Application of the HFNC to be a safe and effective alternative to intubation for accomplishing bronchoscopy with BAL in patients with hypoxemic ARF.

**References**

1. Antonelli M, et al: Noninvasive positive-pressureventilation via face mask during bronchoscopy with BAL in high-risk hypoxemic patients. Chest 1996, 110:724-728.

2. Ricard JD: High flow nasal oxygen in acute respiratory failure. Minerva Anestesiol 2012, 78:836-841.

### A341 Effect of high-flow nasal cannula therapy in ARDS

#### Z. Yu xiang, W. Jia-xing, W. Xiao dan, N. Wen long, W. Yu, Z. Yan, X. Cheng

##### 309th Hospital PLA, Beijing, China

###### **Correspondence:** Z. Yu xiang – 309th Hospital PLA, Beijing, China

**Introduction** Although several approaches for providing supplemental oxygen have been suggested, the best option for patients with acute respiratory failure remains unclear.

**Objectives** To study the effect of the HFNC with ARDS.

**Methods** We chose the 12 patients with ARDS in the study, average 67 years. The patients were all treated with the HFNC in the PLA 309 ICU. According the Berlin's standard , the patients were divided into the mild to moderate ARDS group(7 cases)and the severe ARDS group(5 cases).In order to detect the effect of the HFNC, we recorded the respiratory frequency , Pa0_2_/FiO_2_ ,PaCO_2_ in the 0 h,24 h,48 h,72 h after the treatment of HFNC as well as the failure rate of each group.

**Results** The failure rate in the mild to moderate ARDS group is 14 percent,the HFNC don't cause the significant change in the PaCO_2_ of the group(*P* > 0.05); The respiratory frequency of the patients in the group has been ameliorated in the 48 h due to the HFNC treatment(20.57 ± 2.37vs.23.87 ± 2.64,*P* < 0.05)and the result can be better in the 72 h.As long as the treatment of the HFNC, Pa0_2_/FiO_2_ was significantly higher at 72 h(276.00 ± 108.15vs.177.75 ± 64.23, *P* < 0.05). In the severe ARDS group, the failure rate is 40 percent and there is no statistics significance about Pa0_2_/FiO_2_ before and after the treatment of the HFNC.The respiratory frequency could be ameliorated in the proceeding of HFNC treatment(48 h:19.25 ± 3.77*vs*.25.60 ± 2.50, *P* < 0.05;72 h 20.75 ± 3.59vs.25.60 ± 2.50,*P* < 0.05),but the PaCO_2_ has achieved to the normal level(39.75 ± 7.13vs.30.40 ± 6.98,*P* < 0.05).

**Conclusions** HFNC can ameliorate the symptom of the mild to moderate ARDS, but the effect in the severe ARDS isn't certain. So, the symptom of patients must have been monitored frequently in order to avoid missing the chance of intubation.

**References**

1) Nishimura M. High-Flow Nasal Cannula Oxygen Therapy in Adults: Physiological Benefits, Indication, Clinical Benefits, and Adverse Effects. Respir Care, 2016, 61(4):529-541.

2) Masaji Nishimura. High-flow nasal cannula oxygen therapy in adults [J]. Nishimura Journal of Intensive Care, 2015, 3:15.

3) D. Chiumello , M. Gotti, C. Chiurazzi. High-Flow Nasal Cannula Oxygen Therapy: Physiological Effects and Clinical Data. J.-L. Vincent (ed.), Annual Update in Intensive Care and Emergency Medicine 2016,DOI 10.1007/978-3-319-27349-5_21

4) Laurent Papazian, Amanda Corley, Dean Hess, et al.Use of high-flow nasal cannula oxygenation in ICU adults: a narrative review[J]. Intensive Care Med DOI 10.1007/s00134-016-4277-8.

5) Lemiale V, Mokart D, Resche-Rigon M, et al. The effect of noninvasive ventilation vs oxygen therapy on mortality among immunocompromised patients with acute respiratory failure: a randomized clinical trial[J]. JAMA, 2015, 314:1711-1719.

### A342 Comparison of an ordinary mask and an open face mask: is there a better way to provide oxygen?

#### T. Kobayashi, Y. Onodera, R. Akimoto, A. Sugiura, H. Suzuki, M. Iwabuchi, M. Nakane, K. Kawamae

##### Yamagata University Faculty of Medicine, Anaesthesiology and Critical Care Medicine, Yamagata, Japan

###### **Correspondence:** T. Kobayashi – Yamagata University Faculty of Medicine, Anaesthesiology and Critical Care Medicine, Yamagata, Japan

**Introduction** Oxygen therapy is one of the most popular treatments in the ICU. We typically use oxygen masks to give oxygen to patients who breathe spontaneously. However, ordinary oxygen masks have a risk of CO_2_ rebreathing—especially at lower flow rates (less than 5 l/min).

For our bench study, we investigated the use of an open face mask (Atom Medical, Japan) in alleviating the risk of CO_2_ rebreathing while still delivering a relatively high F_I_O_2_. The result showed that the open face mask mitigated this safety concern. It provided a 10 % higher of inspired oxygen (F_I_O_2_) and a lower inspired carbon dioxide (F_I_CO_2_) as 60-100 % reduction than an ordinary mask (IMJ, Japan). That result was presented in the congress of WFSICCM.

**Objective** We hypothesize that the use of an open face mask is better for our spontaneous breathing patients who need oxygen than an ordinary mask because it can give patients better oxygenation and CO_2_ clearance.

**Methods** This study was performed among 22 ICU patients who suffered from mild respiratory failure. We applied both masks (ordinary and open face mask) with various flow rates: 5, 4, 3, 2, 1 and 0.5 l/min for three minutes, respectively. First, we tried ordinary masks with 5 l/min for 15 minutes and decreased the flow to 0.5 l/min every three minutes. The patients whose SpO_2_ achieved 100 % with 5 l/min of oxygen were excluded. Respiratory parameters (SpO_2_, respiratory rate: RR; F_I_CO_2_, end-tidal CO_2_: EtCO_2_) were recorded at the end of each flow rate.

F_I_CO_2_ and EtCO_2_ were measured via Capnostream^TM^ (Medtronic, US). Next, we investigated an open face mask in the same manner: applying 5 l/min of oxygen for 15 minutes. Finally, we checked patient satisfaction with each mask using a visual analogue scale (VAS: 0-best, 5-worst) at the end of each trial. We used the Wilcoxon matched-pairs signed-ranks test to detect significant differences in the two groups.

**Results** Data from 12 patients were analysed. The median age was 66 (range: 50-87). Most of the patents (92 %) underwent an operation (cardiovascular surgery: 67 %, orthopaedic surgery: 17 %, abdominal surgery: 8.3 %). The patient who did not receive an operation had congestive heart failure.

The group using open face masks showed higher SpO_2_ (Mean: 97.8 ± 0.47 % vs 97.5 ± 0.71 %, p < 0.01) and lower F_I_CO_2_ (0.76 ± 0.16 % vs 2.00 ± 0.77 %, p < 0.01) and EtCO_2_ (35.5 ± 0.36 % vs 37.0 ± 0.78 %, p < 0.01). The respiratory rate did not reveal any significant difference (20.19 ± 0.75/min vs 20.85 ± 0.68/min, p = 0.15). Patient satisfaction with the open face mask was superior to an ordinary oxygen mask (VAS 2.42 ± 1.08 vs 3.25 ± 1.14, p < 0.01).

**Conclusion** Open face masks can provide better oxygenation and reduce CO_2_ rebreathing for the ICU patients in comparison with ordinary masks.

**Reference**

Lamb K et al. Can J Respir Ther. 2016; 52: 13-5.

### A343 Prophylactic assistance for low cough peak expiratory flow at extubation

#### P. Carmona Sanchez, M.D. Bautista Rodriguez, M. Rodriguez Delgado, V. Martínez de Pinillos Sánchez, A. Mula Gómez, J.M. Serrano Simón

##### Hospital Universitario Reina Sofia, Intensive Care Unit, Córdoba, Spain

###### **Correspondence:** P. Carmona Sanchez – Hospital Universitario Reina Sofia, Intensive Care Unit, Córdoba, Spain

**Introduction** The peak expiratory flow (PEF) is a strong predictor of success or failure of extubation. The risk of extubation failure for PEF < 60 L/min is to 6.3 times higher than PEF > 60 L/min (1,2).

**Objectives** To evaluate the impact on extubation outcome of prophylactic noninvasive assistance at extubation of patients with weak cough, and to identify optimal device for assistance.

**Methods** Prospective collected database was conducted of from December 2014 to April 2016. Weak cough was defined by PEF < 60 L/min. The PEF was measured with Cosmed Pony Graphic® spirometer v.4.0 S-CZ before extubation, for the patients mechanically ventilated > 24 h, and without tracheostomy, who passed successfully a sponyaneus breathing trial (SBT) at least of 30 min of pressure support at 5-8 cmH2O, CPAP, or T-T. The patients were then extubated regardless the PEF. The patients with PEF > 60 L/min conventional oxygen therapy was applied, and groups at risk of extubation failure (PEF < 60 L/min) was applied randomly prophylactic CPAP, noninvasive ventilation (VNI BiLevel), or humidified high flow nasal cannula (HFNC). Extubation failure was defined by the need of reintubation within 48 h following extubation. We compared both groups of patients according to the PEF, and prophylactic assistance on groups patients with weak cough, on outcome extubation. Continuous variables were expressed as mean ± SD or median (IRQ) and categorical variables as absolute value and percentage. The comparison of continuous variables was performed by Student t test and Mann-Whitney test and comparison between categorical variables was performed by Fisher's exact test and Chi-square test.

**Results** 137 patients were studied, 89 males (65 %). The two groups of patients according to the PEF, were similar regarding age, APACHE II, underlying chronic disease and duration of mechanical ventilation before extubation. Prophylatic assistance was effectively applied to 72,1 % of patients with PEF < 60 L/min and 87,2 % for PEF > 60 L/min. VNI was applied at 51,2 % (CPAP 18,6 % and BiLevel 32,6 %), Optiflow® 48,8 %. No significant differences were found between the prophylactic devices of assistance at risk group of extubation failure. In the patients with PEF > 60 L/min, extubation failure rate was 12,8 %. There were not differences between diagnosis and extubation failure. Not differences in total stay in ICU between both groups.

**Conclusions** Prophylactic non invasive assistance at extubation could reduce the risk of extubation failure in patients with a weak cough strength without increasing length of stay. It's could be applied to any mode and device (HFNC, CPAP or VNI).

**References**

1. Smina M. Cough peak flows and extubation outcomes. Chest. 2003 Jul;124:262-8. 2. Beuret P. Interest of an objective evaluation of cough during weaning from mechanical ventilation. Intensive Care Med 2009; 35:1090-1093.

### A344 Measure of cough strength at extubation on the screen of the ventilator

#### P. Beuret, C. Fortes, M. Lauer, M. Reboul, J.-C. Chakarian, X. Fabre, B. Philippon-Jouve, S. Devillez, M. Clerc

##### Centre Hospitalier, Intensive Care Unit, Roanne, France

###### **Correspondence:** P. Beuret – Centre Hospitalier, Intensive Care Unit, Roanne, France

**Introduction** Numerous studies have shown that a weak cough strength, if evaluated objectively before extubation by the measure of peak cough expiratory flow (PCEF), is a strong predictor of extubation failure. However, reported cut-off values depend on the device used for the measure. Measuring PCEF on the screen of the ventilator by its flow sensor seems easy to perform, without disconnecting the patient from the ventilator, and could provide a method available everywhere at the bedside.

**Objectives** This prospective study aimed to compare the measure of PCEF on the screen of the ventilator Servo i (Maquet, Solna, Sweden) to the measure obtained with an electronic flowmeter, the Piko-1 (Ferraris Respiratory, Hertford, UK), whose accuracy to predict extubation outcome has been previously reported, with an optimal cut-off value of 35 l/min [1].

**Methods** The PCEF was measured by the respiratory therapist just before extubation for the patients mechanically ventilated for more than 24 hours and who passed successfully a spontaneous breathing trial of 30 minutes of pressure support at 8 cm H2O. The order of the measures with the two devices was changed after inclusion of half of the patients. The best value obtained from two measures was kept for the analysis.

**Results** Over one year 87 patients were eligible for the study; the measure of PCEF was impossible to achieve because of lack of understanding in 14 patients (16 %). Among the 73 patients included for the analysis, there was a significant correlation between the measures obtained with the Piko-1 and the Servo i (r_s_ = 0.831; p < 0.01) (Fig. 135). The linear regression line obtained predicts a cut-off value of PCEF on the Servo i at 60 l/min, corresponding to the value at 35 l/min previously determined with the Piko-1 [1].

**Conclusions** The measure of PCEF just before extubation on the screen of the ventilator Servo i is easy to perform and well correlated with the measure performed by an electronic flowmeter Piko-1. This allows to propose a cut-off value of 60 l/min with the ventilator, below which the cough strength may be judged as weak.

**References**

1. Beuret P. Interest of an objective evaluation of cough during weaning from mechanical ventilation. Intensive Care Med 2009; 35: 1090-1093.

**Fig. 136:**
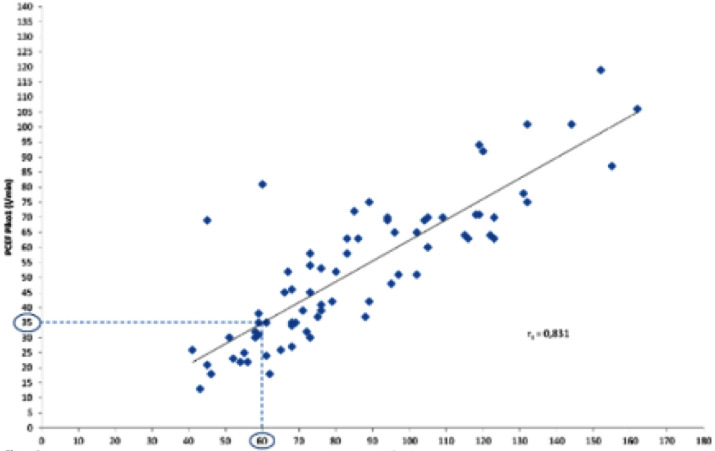
(abstract A344).

### A345 A randomized cross-over physiological study of high flow nasal oxygen cannula versus non-invasive ventilation in adult patients with cystic fibrosis: the HIFEN study

#### N. Rittayamai^1,2,3^, M. Sklar^1,2^, M. Dres^1,2,4^, M. Rauseo^1,2^, C. Campbell^1^, B. West^5^, D.E. Tullis^1,5^, L. Brochard^1,2^

##### ^1^Keenan Research Centre, Li Ka Shing Knowledge Institute, St. Michael's Hospital, Toronto, Canada; ^2^Interdepartmental Division of Critical Care Medicine, University of Toronto, Toronto, Canada; ^3^Division of Respiratory Diseases and Tuberculosis, Department of Medicine, Faculty of Medicine Siriraj Hospital, Bangkok, Thailand; ^4^Neurophysiologie Respiratoire Expérimentale et Clinique, Sorbonne Universités, Paris, France; ^5^Division of Respirology, St. Michael's Hospital, Toronto, Canada

###### **Correspondence:** N. Rittayamai – Keenan Research Centre, Li Ka Shing Knowledge Institute, St. Michael's Hospital, Toronto, Canada

**Introduction** Non-invasive ventilation (NIV) is the first option for the treatment of cystic fibrosis (CF) patients with acute exacerbation. High flow nasal oxygen cannula (HFNC) is a heated humidified, high flow oxygen delivery system that has demonstrated benefits in terms of survival in patients with acute hypoxemic respiratory failure and in preventing postextubation failure. This device may also have benefits in patients with hypercapneic respiratory failure including CF patients. We hypothesize that HFNC would not be inferior to NIV in terms of reducing work of breathing and improving breathing pattern in CF patients requiring ventilator support.

**Objectives** To compare HFNC vs. NIV induced changes in inspiratory work of breathing assessed by the thickening fraction of the diaphragm (TFdi), and breathing pattern, hemodynamics, dyspnea and comfort.

**Methods** CF patients with acute exacerbation requiring ventilator support were ventilated with HFNC and NIV for 30 minutes in random order. TFdi was measured using ultrasound at baseline and at 25 minutes with each device. Pulse oximetry (SpO_2_), transcutaneous CO_2_ (PtcCO_2_) were continuously recorded and respiratory rate, tidal volume (V_T_) and minute ventilation (MV) measured by bio-impedance techniques; hemodynamics, dyspnea and comfort assessed by visual analog scales were also recorded. Results were compared using a Mann Whitney, 2 tailed test, and are expressed as mean (SD) with each intervention compared to baseline conditions.

**Results** 12 patients were enrolled (mean age 31.3 years, mean FEV_1_/FVC 49.9 %, mean FEV_1_ 28.4 % predicted). TFdi was similar with the two techniques, but HFNC, compared to NIV, resulted in a significant decrease in respiratory rate (-20.2 % (18.0) vs -0.2 % (18.7), p = 0.024) and a lower mean arterial pressure (0.3 % (5.6) vs 5.8 % (4.9), p = 0.017). No significant differences were found in heart rate, SpO_2_, PtcCO_2_, V_T_, MV, comfort and dyspnea (Table 111).

**Conclusions** HFNC was not inferior to NIV with respect to diaphragmatic work in CF patients who had an indication for ventilator support. These preliminary data suggest that HFNC may confer physiological benefits by decreasing respiratory rate, and constitute an interesting alternative to NIV.

**References**

1. Madden BP, Kariyawasam H, Siddiqi AJ, Machin A, Pryor JA, Hodson ME. Noninvasive ventilation in cystic fibrosis patients with acute or chronic respiratory failure. Eur Respir J 2002;19(2):310-3.

2. Vivier E, Mekontso Dessap A, Dimassi S, Vargas F, Lyazidi A, Thille AW, et al. Diaphragm ultrasonography to estimate the work of breathing during non-invasive ventilation. Intensive Care Med 2012;38(5):796-803.

**Grant acknowledgement**

This study was supported by Cystic Fibrosis Canada and by a grant from Siriraj Hospital in Bangkok. LB's laboratory received a grant and equipment from Fisher Paykel for the study. LB holds the Keenan Chair in Critical Care and Acute Respiratory Failure.

**Table 111 (abstract A345).** Percentage of change from baseline and 25 minutes

	HFNC; Mean (SD)	NIV; Mean (SD)	p-value
Thickening fraction of the diaphragm	-1.8 (32.7)	-2.0 (32.8)	0.880
Respiratory rate	-20.2 (18.0)	-0.2 (18.7)	0.024
SpO2	1.5 (3.2)	0.3 (2.9)	0.450
PtcCO2	1.2 (4.7)	0.8 (4.0)	0.667
Minute ventilation	-17.7 (33.2)	-11.3 (23.1)	0.627
Mean arterial pressure	0.3 (5.6)	5.8 (4.9)	0.019
Heart rate	-3.8 (5.7)	-3.7 (4.4)	0.793
Dyspnea	11.1 (63.0)	-11.1 (42.8)	0.338
Comfort	-22.9 (13.9)	-15.8 (27.6)	0.310

### A346 High-flow nasal cannula effectively washes out CO2 from anatomical dead space in a sophisticated respiratory model made by 3D printer

#### Y. Onodera, R. Akimoto, H. Suzuki, M. Okada, M. Nakane, K. Kawamae

##### Yamagata University, Department of Anesthesiology, Faculty of Medicine, Yamagata, Japan

###### **Correspondence:** Y. Onodera – Yamagata University, Department of Anesthesiology, Faculty of Medicine, Yamagata, Japan

**Introduction** The washout effect of HFNC has not been well evaluated. Our prior study, presented at the ESICM congress 2014, evaluated the reduction of P_ET_CO_2_ with HFNC using a tracheal intubation trainer and a test lung. Although we concluded that the washout effect is most effective with a relatively low flow, the model had a large upper airway dead space of 200 mL, which was thought to have influenced P_ET_CO_2_. We also were not able to measure PEEP, which is also thought to have interaction between HFNC flows. Therefore, we developed a more sophisticated artificial respiratory model using a 3D printer, and used a lung model equipped with a pressure sensor to quantitatively evaluate the washout effect of HFNC and the interaction with PEEP.

**Objectives** To quantitatively evaluate the washout effect and interaction with PEEP using different levels of HFNC flow.

**Methods** The airway model was made by a 3D printer using the craniocervical CT data of a healthy 32-year-old male. The total anatomical dead space was adjusted to 180 mL (3 mL/kg). The model lung (LUNGOO : Air water safety service Inc., Kobe, Japan) had the following settings: normal (Compliance (C) 50 ml/cmH_2_O, resistance (R) 5 cmH_2_O/L/s, tidal volume (Vt) of 500 mL, respiratory rate (RR) 16 /min), obstructive (C 70, R 20, Vt 700, RR 10), restrictive (C 30, R 5, Vt 300, RR 30) with inspiratory time at 1 second, and residual volume of 1000 mL. CO_2_ was infused into respiratory lung models to reach P_ET_CO_2_ of 40 mmHg without HFNC. After setting P_ET_CO_2_ with each lung model, HFNC with flows of 10 to 60 L/min were applied and the change in P_ET_CO_2_ in the subglottic area and the inlet of the lung model was measured. PEEP inside the model was also recorded.

**Results** With the normal lung open-mouth model, 10 L/min of HFNC flow decreased the P_ET_CO_2_ of the subglottic area and the inlet in a lung model to 30 mmHg. Increasing HFNC flow did not decrease P_ET_CO_2_ in either area. With the normal lung closed-mouth model, P_ET_CO_2_ of all sites required a HFNC flow of 40 L/min to decrease, and reached 30 mmHg with HFNC flow of 60 L/min. With the obstructive lung open mouth model, P_ET_CO_2_ of all sites had the same trends as the normal lung open-mouth model. With the restrictive lung open mouth model, 20 L/min of HFNC flow decreased the P_ET_CO_2_ of the subglottic area and the inlet to 25 mmHg, and did not decrease thereafter. As HFNC flow was increased, PEEP gradually generated up to around 8 to 5 cm H_2_O with open mouth models and up to 17 cmH_2_O with the normal lung closed mouth model.

**Conclusions** The washout effect of HFNC is thought to reduce the P_ET_CO_2_ enough to have a clinical effect. Contrary to the relation of HFNC flow and generated PEEP, the HFNC reduced P_ET_CO_2_ with a relatively low flow in open mouth models. HFNC required more flow to reduce P_ET_CO_2_ with a closed mouth model, which is thought to be due to a less efficient washing out of the dead space than in the open model.

**Grant acknowledgment** Nothing to Declare.

**Fig. 137 (abstract A346).**
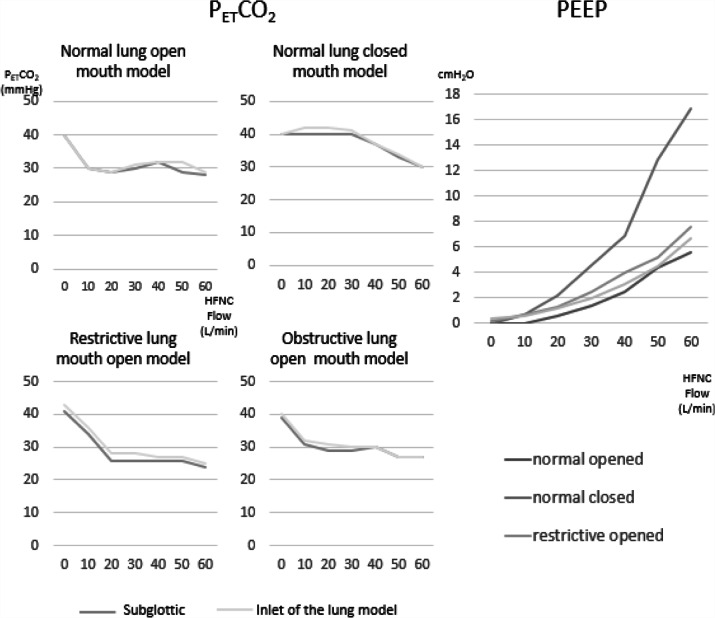
Relation of PETCO2, PEEP and HFNC flows

### A347 Impact of frailty score on survival rate in patients admitted to critical care with acute respiratory failure treated with bilevel noninvasive ventilation (NIV) in a large teaching hospital critical care department

#### N. Ahmad, M. Wood, A. Glossop

##### Northern General Hospital, Sheffield Teaching Hospital NHS Trust, Anaesthesia and Critical Care, Sheffield, United Kingdom

###### **Correspondence:** N. Ahmad – Northern General Hospital, Sheffield Teaching Hospital NHS Trust, Anaesthesia and Critical Care, Sheffield, United Kingdom

**Introduction** Clinical frailty is increasingly used and recognised to predict mortality and functional dependence following critical admission^1^ although less is known regarding its impact on specific patient groups. Bilevel noninvasive ventilation (NIV) is frequently used to treat acute respiratory failure (ARF) of various aetiologies. Whilst NIV is known to be beneficial in exacerbations of COPD^2^, acute cardiogenic pulmonary oedema^3^ and other presentations of ARF, there is scarce data available on the impact frailty may have on the success of NIV in this patient group.

**Objective** We examined the impact of frailty score on outcomes in patients receiving NIV for mixed aetiology of ARF within our critical care (CC) unit.

**Methods** Case notes and electronic patient data records (Metavision,iMDsoft,MA,USA) were retrospectively reviewed for adult patients with ARF who received NIV between December 2011 and April 2013. Patients who received continuous positive airway pressure and those with a primary surgical problem were excluded. Patient frailty score was recorded on admission using the Rockwood frailty index in on 55 consecutive patients. Demographic data, body mass index (BMI), primary cause of ARF and hospital mortality were recorded.Patients were divided into two groups according to their frailty score: below 4 (very fit to vulnerable) and 5 and above (frail to severely frail).

**Results** 55 patients were identified. 44 % were male, mean age 63 years. Presenting diagnosis was pneumonia in 32 patients (58 %) and acute exacerbation of COPD in 13 patients (23 %). The overall CC survival rate was 84 % with 73 % surviving to hospital discharge. 31 patients had a frailty score of 5 or above (77 % male, mean age 66 years) and 24 patients had a frailty score of 4 or less (50 % male, mean age of 62 years). Patients with a lower frailty score had a higher overall survival rate (91 % on CC and 83 % to hospital discharge) compared to those with a higher frailty score (70 % on CC and 51 % to hospital discharge). The mean BMI for both groups was identical (30.5 kg/m^2^). There were no differences in the mean length of stay in CC or in hospital or duration of NIV use between the two groups.

**Conclusion** Our data suggests that a Rockwood frailty score of 5 or above is associated with reduced CC and hospital survival in patients with ARF requiring NIV. Use of a frailty index may be a useful predictive marker in patients admitted to critical care and further work is warranted to define the prognostic value of frailty scoring.

**References**

1. Le Maguet, P.et al. Prevalence and impact of frailty on mortality in elderly ICU patients: a prospective, multicenter, observational study. *Intensive Care Med, 2014.4(5):p.674-82.*

2. Roberts, C.M.et al. Acidosis, non-invasive ventilation and mortality in hospitalised COPD exacerbations. *Thorax, 2011.66(1):p.43-8.*

3. Masip, J.et al. Noninvasive ventilation in acute cardiogenic pulmonary edema: systematic review and meta-analysis. *JAMA, 2005.294(24):p.3124-30.*

### A348 Has the high flow therapy changed our approach to the acute respiratory failure?

#### J. Higuera Lucas, A. Blandino Ortiz, D. Cabestrero Alonso, R. De Pablo Sánchez, L. Rey González

##### Hospital Universitario Ramón y Cajal, Unidad de Cuidados Intensivos, Madrid, Spain

###### **Correspondence:** A. Blandino Ortiz – Hospital Universitario Ramón y Cajal, Unidad de Cuidados Intensivos, Madrid, Spain

**Introduction** The use of high flow therapy in the critical care units for adults is becoming more common from few years on. This therapy approaches the patient with acute respiratory failure from a new way of treatment.

**Objectives** Our goal is to analyze the impact and the results of the application of high flow therapy in an intensive care unit of a tertiary hospital. Evaluate if the use of such a therapy has avoided the use of mechanical ventilation in the patients.

**Materials and methods** The study has summarized all the patients that have been treated with the oxygen therapy treatment between May 2013-October 2015. The reviewed data has been: SOFA, APACHE II, SAPS II, tabulated diagnosis, etiology of the respiratory failure, length of stay, use of high flow before or after the intubation, evaluate if the intubation has been avoided, mortality and mortality in 30 days.

**Results** The study includes 113 patients, medium average age 58, men 56.5 %,and women 43.5 %. The severity indexes of the study are: SOFA 8,37 ± 4,44(1-19), APACHE II 19,68 ± 8,75(3-44), SAPS2 49,31 ± 20,59(11-95). The average stay of the patients is 14,70 days.

The origin of the disease is respiratory in the 55 % of the causes, hematological 22 %, septic 14,5 %, cardiologic 3,8 % and miscellaneous in all the rest.

The cause of the ventilation decompensate has been:respiratory 77,1 %, septic 16,8 %, and miscellaneous in all the other cases. The causes of the respiratory failure have been hypoxemic in the 75,6 % of the cases, hipercapnic in the 1,5 % and mixed causes in the rest.

42,7 % of the times high flow therapy is provided before the mechanical ventilation. 19,8 % of the times after the mechanical ventilation.

It is considered that avoids mechanical ventilation in 43,5 % of the cases.

Applying non parametrical test the group of patients that needs mechanical ventilation has a higher mortality (p < 0, 0001)

**Conclusions** The use of high flow therapy has changed the attitude towards the patients with insufficiency respiratory diseases avoiding in many cases the use of mechanical ventilation.

More controlled studies are needed showing which other patients can be beneficed by the high flow therapy to prevent the delays in the start of mechanical ventilation.

**References**

[1] Williams R, Rankin N, Smith T, Galler D, Seakins P. Relationes-hip between the humidity and temperature of inspired gas and the function of the airway mucosa. Crit Care Med.1996; 24:1920-9

[2] Williams AB, Ritchie JE, Gerard C. Evaluation of a high-flownasal oxygen delivery system: Gas analysis and pharyngeal presure. Intensive Care Med. 2006;32 (S1):S219

[3] Groves N, Tobin A. High flow nasal oxygen generates positive air-way pressure in adult volunteers. Aus Crit. Care2007;20:126-31

[4] Sztrymf B, Messika J, Mayot T, Lenglet H, Dreyfuss D, Ricard JD. Impact of high flow nasal cannula oxygen therapy on intensive care unit pacients with acute respiratory failure: A prospective observacional study. J Crit. Care.2012;27:324,e9-13

### A349 Comparative evaluation of three full face masks for delivering NIV

#### R. Costa, G. Spinazzola, A. Pizza, G. Ferrone, M. Rossi, M. Antonelli, G. Conti

##### Catholic University of Rome, Department of Anesthesia and Intensive Care, Rome, Italy

###### **Correspondence:** R. Costa – Catholic University of Rome, Department of Anesthesia and Intensive Care, Rome, Italy

**Introduction** Full face masks have been proposed to improve patient comfort during NIV and thus increase NIV success.

**Objectives** To compare three full Face masks (FF) (Dimax, Dimar, Italy; Performax, Respironics,US and BiTrac, Pulmodyne, US).

**Methods** An adult mannequin, connected to an active lung simulator (Compliance : 60 ml/cmH_2_O; Respiratory Resistances : 4 cmH_2_O/L/sec) breathing at 20 and 30 breaths/minute was ventilated with the three FF in Pressure Support (PS 10 and 15 cmH_2_O, PEEP 8 cmH_2_O) with a fast pressurization ramp (100 %) and 2 expiratory triggers (25 %, 50 %). The data analysis evaluated Patient-ventilator interaction (**Delay**_**trinsp,**_ **Delay**_**trexp,**_ **Time**_**sync**_ **)** and masks performance (**Swing**_**trigger**_, **PTP**_**t**_, **PTP**_**300**_ **, PTP**_**500**_ **Index)**.

**Results** At RR 20, Timesync was longer with FF-Pulmo and FF-Dimar, while, at RR 30, no significant difference was found between the masks. FF-Dimar and FF-Respir significantly increased Swing_trigger_ and PTP_t_ at RR 20, but both FF showed a better performance in terms of PTP_300._ No significant difference was found in terms of PTP_500_ Index.

**Conclusions** Independently from the setting, all FF guaranteed a Time_sync_ above 50 % of the neural inspiratory time. The different shape and material of the FF significantly influenced our results: softer flange and the need for less head-strips tightness required by FF-Dimar caused an initial pressure dissipation with longer Delay_trinsp_, Swing_trigger_ and PTP_t._ Conversely, the need to strongly pull the head-strips with FF-Pulmo to reduce leaks, explained its superior performance and interaction.

**Grant acknowledgment**

None.

## OUTCOME ANALYSIS I

### A350 Three years of experience in pulmonary embolism approach in an intermediate care unit

#### H. Ribeiro, J. Alves, M. Sousa, P. Reis

##### Centro Hospitalar de Entre o Douro e Vouga, Serviço de Medicina Intensiva Polivalente, Santa Maria da Feira, Portugal

###### **Correspondence:** H. Ribeiro – Centro Hospitalar de Entre o Douro e Vouga, Serviço de Medicina Intensiva Polivalente, Santa Maria da Feira, Portugal

**Introduction** Venous Thromboembolism encompasses deep venous thrombosis (DVT) and pulmonary embolism (PE) and has a significant impact on morbidity and mortality, being responsible for more than 370000 deaths every year in 6 European countries. Nowadays, clinical scores and diagnostic non-invasive techniques have improved diagnosis and early treatment: anticoagulant and thrombolytic therapy.

**Objectives** To characterize patients admitted to Intermediate Care Unit (ICU) due to PE and submitted to thrombolytic therapy.

**Methods** Retrospective observational study of patients admitted due to PE in the ICU between January 2013 and December 2015. Data was collected and analysed with SPSS and descriptive and analytic statistic were performed. A value of p ≤ 0,05 was considered statistically significant.

**Results** During 3 years, 82 patients (51 female, median age 69,5, ranging from 24 to 89, 48 with more than 65 years) were admitted due to PE, more frequently from the Emergency Service (86,6 %).The most common symptoms were dyspnea (61 %), chest pain (50 %) and syncope (24,4 %) and 54 patients were classified as low probability PE using Wells Criteria. Computed Tomography was positive in 97,6 % of patients. Using Pulmonary Embolism Severity Index (PESI) Score, 9 patients had very low risk score (mean age 40,4 ± 7,1 years), 13 low risk (mean age 50 ± 16,9 years), 26 moderate risk (mean age 70,4 ± 10,3 years), 14 high risk (mean age 71,4 ± 12,6 years)and 18 very high risk score (mean age 72,6 ± 14,2 years). Age was different between PESI Score classes (p < 0,001). The most common DVT risk factors were age, peripheral venous insufficiency (24,4 %), obesity (23,2 %), previous immobilization (17,1 %). In the ICU, 10 patients were admitted due to massive PE, 23 submassive PE, 11 due to hypoxemia and 23 for other reasons, more frequently due to the need constant monitoring. DVT was confirmed in 17 patients. Thrombolytic therapy was administered to 29 patients (13 submassive PE; 13 massive PE), 3 with registered complications (haemoptysis, haematuria and multiple hematomas and skin haemorrhage). Ventilation and vasopressor support were needed in 7,3 %. One patient was transferred to Intensive Care Unit; 1 died in ICU, 2 after discharge of ICU and 2 in the next 6 months.

**Conclusions** Age is an important risk factor in patients admitted to ICU. Although 21 patients were classified as low/very low risk patients they were clinically unstable, had cardiac repercussion or needed constant monitoring. These patients were younger, with less comorbidities and with better physiological condition that may have underestimated PESI Score. This study highlights the importance of individualized and clinical evaluation, early therapy and the low rate of complications in patients submitted to thrombolytic therapy.

### A351 Socioeconomic status in ICU survivors and post-hospital outcomes

#### C.S. Socolovsky^1^, R.P. Cauley^2^, J.E. Frankel^3^, A.L. Beam^4^, K.O. Olaniran^5^, F.K. Gibbons^6^, K.B. Christopher^7,8^

##### ^1^Massachusetts General Hospital, Department of Medicine, Boston, MA, United States; ^2^Massachusetts General Hospital, Department of Surgery, Boston, MA, United States; ^3^Spaulding Rehabilitation Hospital, Physical Medicine & Rehabilitation, Boston, MA, United States; ^4^Harvard Medical School, Biomedical Informatics, Boston, MA, United States; ^5^Massachusetts General Hospital, Renal Division, Boston, MA, United States; ^6^Massachusetts General Hospital, Pulmonary and Critical Care Medicine, Boston, MA, United States; ^7^Brigham and Women's Hospital, Renal Division, Boston, MA, United States; ^8^Brigham and Women's Hospital, Channing Division of Network Medicine, Boston, MA, United States

###### **Correspondence:** C.S. Socolovsky – Massachusetts General Hospital, Department of Medicine, Boston, MA, United States

**Introduction** Complex factors in ICU survivors such as health literacy, socioeconomic status and social environment may be predictive of adverse outcomes following hospital discharge but are not well studied.

**Objectives** We hypothesized that Medicaid Insurance, a proxy for individual low socioeconomic status, would be associated with increased hospital readmission rates following hospital discharge.

**Methods** We performed a two center observational study of patients treated in medical and surgical intensive care units in Boston, Massachusetts. We studied 82,583 patients, age ≥ 18 years, who received critical care between 1998 and 2012 and survived hospitalization. The exposure of interest was Medicaid Insurance status. The primary outcome was unplanned 30-day hospital readmission. Adjusted odds ratios were estimated by multivariable logistic regression models with inclusion of covariate terms for gender, race, Deyo-Charlson index, type (surgical vs. medical), sepsis, acute organ failure, area deprivation index (proxy for area socioeconomic status and social environment) and age as a restricted cubic spline function. In a subset admitted to Spaulding Rehabilitation Hospital following hospital discharge, (n = 4,232) we evaluated the association of Medicaid Insurance status and rehabilitation hospital length of stay utilizing a negative binomial regression model.

**Results** The cohort patients were 58 % male, 20 % nonwhite and 51 % surgical. 10 % of the cohort had sepsis and the mean age was 61.2 years. Medicaid Insurance was present in 10 %. Those with Medicaid Insurance were significantly younger, more frequently non-white, with higher sepsis and acute lung injury rates. Unplanned 30-day readmission rate was 14.1 %. 33.1 % were discharged to a care facility. 90-day post-discharge mortality was 6.5 % in patients with Medicaid Insurance and 7.3 % in patients without. Medicaid Insurance was a robust predictor of 30-day hospital readmission and remained so following multivariable adjustment. Patients with Medicaid Insurance have an adjusted OR of 30-day readmission of 1.31 (95%CI, 1.22-1.41; P < 0.001) relative to patients without Medicaid Insurance. Further, patients with Medicaid Insurance have an adjusted OR for discharge to a care facility of 1.34 (95%CI, 1.26-1.42; P < 0.001) relative to patients without Medicaid Insurance. Finally, patients with Medicaid Insurance compared to those without Medicaid Insurance, are expected to have a 1.2 fold times greater rehabilitation hospital length of stay [adjusted IRR = 1.21 (95%CI 1.11, 1.32) P < 0.001].

**Conclusions** In critical illness survivors, individual socioeconomic status as reflected by Medicaid Insurance is a robust predictor of hospital readmission, placement in a care facility and rehabilitation length of stay. Disparities in ICU survivor outcomes are likely multifactorial involving individual, acute illness, hospital, post-hospital, and neighborhood-level factors that alter optimum post ICU recovery.

### A352 Outcome after critical care predicted by preceding ward length of stay

#### J. Pennington, P. Zolfaghari

##### Barts Health NHS Trust, Adult Critical Care Unit, London, United Kingdom

###### **Correspondence:** J. Pennington – Barts Health NHS Trust, Adult Critical Care Unit, London, United Kingdom

**Introduction** Prolonged hospital stay prior to admission to the intensive care unit has been shown to be independently associated with poorer outcome (1,2). Even a few hour delay in transfer from emergency department to intensive care worsens outcome (3). This may relate to an ongoing deterioration of physiological function while in hospital, potentially influenced by the process or disease state that culminates in admission to critical care.

We investigated whether common illness severity scores e.g. Acute Physiology and Chronic Health Evaluation II (APACHE II) or Intensive Care National Audit & Research Centre (ICNARC), are significantly different in patients admitted after a prolonged ward stay. We describe mortality and ICU / hospital length of stay in said patients, in an order to elucidate predictors for outcome.

**Objectives** Show higher length of stay before ICU admission as an independent predictor of outcome

· Identify whether ICU scoring systems predict outcome in our population

· Identify whether serum albumin or other surrogate marker of frailty (e.g. Creatinine) can help predict outcome?

**Methods** Retrospective analysis of prospectively collected data of all admissions in ICNARC database to 44 bed adult critical care unit in University Associated London Major Trauma Centre over two-year period (1^st^ January 2013 - 31^st^ December 2014).

Demographic data, APACHE II, ICNARC score, ICU mortality, and length of stay on ward preceding ICU, within ICU and overall hospital length of stay were collected. Data was analyzed using ANOVA tests, according to preceding ward length of stay.

**RESULTS.** n = 4340

Mean APACHE 2 score for all admissions was 14.88 (sd 7.0)

P < 0.01 ANOVA comparing Length of ICU stay between 0-7 and >28 days.

P < 0.01 ANOVA comparing APACHE2 across all ward LOS groups.

P < 0.01 ANOVA comparing serum albumin across ward LOS 0-7 days vs >28 days, and non significant across other groups.

**Conclusions** Prolonged pre-ICU hospital admission is associated with longer ICU and hospital admission and generally higher ICU mortality. APACHE2 scoring and serum albumin predict outcome.

**References**

1. Goldhill DR, McNarry AF, Hadjianastassiou VG, Tekkis PP. The longer patients are in hospital before Intensive Care admission the higher their mortality. Intensive Care Med. 2004 Oct;30(10):1908-13.

2. Higgins TL, McGee WT, Steingrub JS, Rapoport J, Lemeshow S, Teres D. Early indicators of prolonged intensive care unit stay: impact of illness severity, physician staffing, and pre-intensive care unit length of stay. Crit Care Med. 2003 Jan;31(1):45-51.

3. Chalfin DB, Trzeciak S, Likourezos A, Baumann BM, Dellinger RP, DELAY-ED study group. Impact of delayed transfer of critically ill patients from the emergency department to the intensive care unit. Crit Care Med. 2007 Jun;35(6):1477-83.

**Table 112 (abstract A352).** Demographics & scores

	LOS prior to ICU admission (days)				
	0–7	8–14	15–21	21–28	>28
Number of patients	3872	216	93	55	104
Age (years)	56.0 (sd 19.1)	62.8 (sd 15.5)	61.1 (sd 15.1)	60.5 (sd 16.0)	63.2 (sd 14.3)
APACHE II score	14.5 (sd 6.9)	17.5 (sd 7.4)	17.7 (sd 7.0)	18.1 (sd 7.7)	18.4 (sd 6.9)
APACHE II probability of death (%)	18.0 (sd 20.9)	24.1 (sd 22.3)	26.0 (sd 21.7)	24.2 (sd 24.1)	26.2 (sd 22.8)
ICNARC Score	13.9 (sd 8.7)	15.4 (sd 8.6)	15.4 (sd 9.1)	16.9 (sd 9.5)	15.5 (sd 8.5)
ICNARC mortality probability (%)	15.6%	20.9%	20.3%	24.3%	21.4%
Outreach involvement (%)	7.6%	26.4%	29.0%	43.6%	45.2%
LOS on ICU (days)	6.5 (sd 14.3)	8.2 (sd 16.0)	7.3 (sd 11.1)	10.8 (sd 22.3)	11.5 (sd 24.3)
LOS hospital (days)	20.9 (sd 27.1)	43.8 (sd 40.2)	51.3 (sd 41.6)	54.8 (sd 27.5)	108.7 (sd 70.3)

**Fig. 138 (abstract A352).**
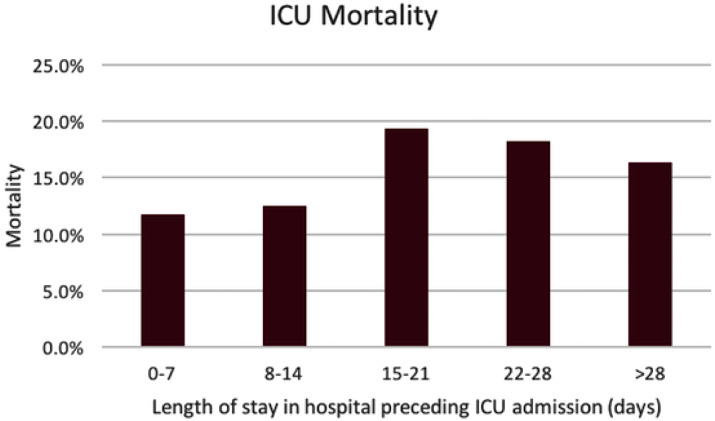
ICU Mortality vs Preceding ward Length of stay

**Fig. 139 (abstract A352).**
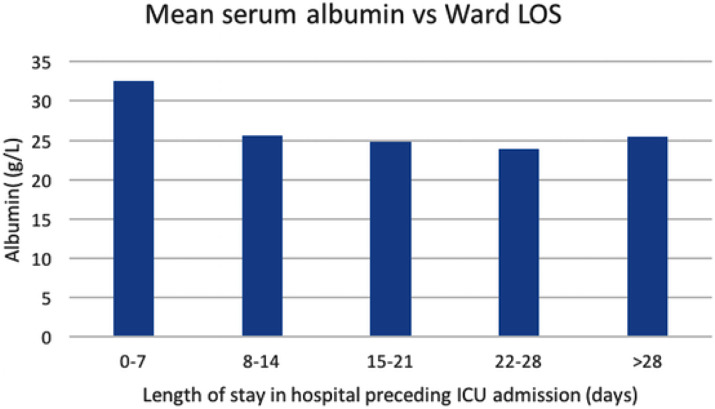
Albumin vs Ward LOS

### A353 A retrospective cohort study evaluating clinical outcomes of inter-hospital retrieval ECMO patients

#### H.S. King, H.H.Y. Kong, H.P. Shum, W.W. Yan

##### Pamela Youde Nethersole Eastern Hospital, Department of Intensive Care, Hong Kong, Hong Kong, China

###### **Correspondence:** H.S. King – Pamela Youde Nethersole Eastern Hospital, Department of Intensive Care, Hong Kong, Hong Kong, China

**Introduction** A historical cohort study of patients who received Extra-corporeal membrane oxygenation (ECMO) in ICU of a regional hospital.

**Objectives** To compare the clinical outcomes between in-house ECMO patients and inter-hospital retrieval ECMO patients.

**Methods** Primary outcome was the ICU mortality rate between in-house and inter-hospital retrieved ECMO patients. Mann-Whitney U tests and Fisher's exact tests were used for comparisons of continuous and categorical variables respectively. 2-tailed p-values < 0.05 represented statistical significance.

**Results** Between 2009 to 2015, 112 patients received ECMO. Among them, 101 patients (77 VV-ECMO, 24 VA-ECMO) with complete data for further analysis. Overall mortality rate was 40/101 (39.6 %). Mean *Acute Physiology and Chronic Health Evaluation* (APACHE) II scores were 30.03 for in-house ECMO vs. 30.80 for inter-hospital retrieval ECMO (p = 0.774).

For those patients who received ECMO for respiratory support (VV-ECMO, n = 77), their mean Respiratory Extracorporeal Membrane Oxygenation Survival Prediction (RESP) score (3.64 vs. 3.03, p = 0.060) and ICU mortality (34.8 % vs. 19.4 %, p = 0.199) were not significantly different between in-house and retrieved cases. Predicted hospital survival for RESP score risk classe II (score 3 to 5) was 76 %.

For those patients who received ECMO for circulatory support (VA-ECMO, n = 24), their mean Survival After Veno-arterial-ECMO (SAVE) score (-7.2 vs. -2.75, p = 0.304) and ICU mortality (86.7 % vs. 55.6 %, p = 0.150) were not significantly different between in-house and retrieved cases. Predicted hospital survival for SAVE score risk class IV (score -9 to -5) and class III (score -4 to 0) were 30 % and 18 % respectively.

For in-house ECMO patients, higher proportions of non-infective aetiologies for VV-ECMO (RESP score) and post-MI cariogenic shock for VA-ECMO were observed. These conditions have poorer prognosis and lower reversibility, which may account for the poorer RESP/SAVE scores (though statistically insignificant) and higher predicted and observed mortality.

**Conclusions** Our findings did not show a statistically significant difference in ICU mortality rate between in-house and retrieval ECMO groups. Inter-hospital ECMO retrieval is feasible for further development. The RESP score and SAVE score systems provides a tool for monitoring of our centre´s performance in comparison to ECMO centres worldwide.

**References**

1. Schmidt M, *et al.*; Survival after Extracorporeal Membrane Oxygenation for Severe Acute Respiratory Failure - The Respiratory Extracorporeal Membrane Oxygenation Survival Prediction (RESP) Score. *Am J Respir Crit Care Med* Vol 189, Iss 11, pp 1374-1382

2. Schmidt M, *et al*.; Predicting survival after ECMO for refractory cardiogenic shock: the survival after veno-arterial-ECMO (SAVE) score. *European Heart Journal* First published online: 1 June 2015

**Grant acknowledgment**

None.

### A354 Evaluation of outcome from intensive care units in turkey: a prevalence study

#### C. Kaymak^1^, N. Okumus^2^, A. Sari^2^, B. Erdogdu^2^, S. Aksun^2^, H. Basar^1^, A. Ozcan^1^, N. Ozcan^1^, D. Oztuna^3^

##### ^1^Ministry of Health, Ankara Training and Research Hospital, Anesthesiology and Reanimation Department, Intensive Care Unit, Ankara, Turkey; ^2^Ministry of Health, Departmant of Health Services, Ankara, Turkey; ^3^University of Ankara, Faculty of Medicine, Medical Biostatistics Department, Ankara, Turkey

##### **Correspondence:** C. Kaymak – Ministry of Health, Ankara Training and Research Hospital, Anesthesiology and Reanimation Department, Intensive Care Unit, Ankara, Turkey

**Introduction** The outcome in critically ill patients regarded with prognosis has many background effects of risk factors. An aging population and chronic diseases may also result in an increased number of patients in intensive care unit (ICU). Clinical results have revealed the need for outcome examination and guidance on the effective use of ICU.

**Objectives** The aim of this study was to evaluate mortality among patients in Turkish ICUs. Regarding this, the present study analyzed APACHE II databases in critically ill patients at secondary and tertiary referral hospital ICUs in Turkey.

**Methods** During the study period, clinical data that were collected concurrently for each patient contained demographic details, diagnostic category leading to ICU admission and APACHE II scores following ICU admission. Patients were followed up during ICU stay. The equation coefficients for APACHE II were supplied by APACHE Medical Systems. The mortality in intensive care units was analyzed according to APACHE II scores. The other attempts performed during ICU stay were also recorded.

**Results** 13.313 patients were enrolled in this study. The 69.9 % of patients were > 60 years old and 53.8 % of them were male. The mean APACHE II score was 21.49. The ICU's mortality rate was 44.5 %. The mechanical ventilation was determined as the most performed attempt in ICU's with a ratio of 55.9 %. The ratio of central venous catheterization was found 53.5 %. The ratios of systemic infection and antibiotic administration were 63.6 % and 80.7 %, respectively.

**Conclusion** In the present study, the patients hospitalized in ICU's of ministry, university, and private hospitals were analyzed all over Turkey. Early identification of patients at risk, both before admission and after discharge from ICU, may allow to prevent some of the physiologic abnormalities contributing to the APACHE II score. There was a wide difference in outcome for patients admitted to different ICU's by using risk adjustment methods.

### A355 Development of an ICU-specific questionnaire for patient-reported outcome measures

#### J.A. Malmgren^1^, S. Lundin^1^, K. Torén^2^, M. Eckerström^2^, A. Wallin^2^, A.-C. Waldenström^2^, for the Section on Ethics of the ESICM

##### ^1^Sahlgrenska University Hospital, Dep. of Anaesthesiology and Intensive Care, Gothenburg, Sweden; ^2^University of Gothenburg, Gothenburg, Sweden

###### **Correspondence:** J.A. Malmgren – Sahlgrenska University Hospital, Dep. of Anaesthesiology and Intensive Care, Gothenburg, Sweden

**Introduction** Growing interest in the long-term effects after critical care has formed investigator-led clinical research groups around the world. Work aiming at standardizing core outcome measures and instruments for randomized clinical trials is ongoing. Whether these outcome measures reflect the domains most valued by patients or if important issues are missing from the existing scales is unclear. Commonly used tools like SF-36 and EQ-5D are too unspecific.

**Objectives** To develop and validate a questionnaire for patient-reported outcome measures (PROM) after critical care.

**Methods** During a 24-months qualitative phase, 35 former ICU-patients were interviewed in a semi-structured way, providing detailed information on symptoms and difficulties in all areas of everyday life. Patients were recruited from the post-ICU clinic at Sahlgrenska University Hospital, covering both urban and rural areas. The interviews were recorded, transcribed, and issues were categorized into 13 hypothesized domains: cognitive, executive/fatigue, physical health, pain, mental health, daily activities, sleep, food/drink/smoking, sexuality, hearing/visual/dysphagia, intestinal and urinary problems, and return to work/financial situation. After searching the literature and commonly used assessment tools, additional issues were included. All issues were then rephrased into questions, with care taken to maintain only one conceptual entity per question, and with the recall period usually being the last month. Adequate scales for frequency, intensity and duration were used. All questions were validated face-to-face with another set of former ICU-patients and with non-ICU-treated controls to make sure the wording was easily understood and neither confusing nor upsetting.

**Results** The questionnaire contains 271 questions. It is currently being tested in a pilot study with 650 patients, recruited six months to three years after discharge from the ICU, and 200 controls, matched for age and gender. The questionnaire is sent by mail after an invitation letter followed by a phone call, and returned in a pre-stamped envelope. Returned questionnaires are being scanned and data digitally imported into SPSS, where additional clinical data will be added. After comparison with controls, item reduction will follow, resulting in an ICU-specific PROM questionnaire.

**Conclusions** A patient-centred, ICU-specific questionnaire will be available for long-term follow-up in the post-ICU clinic. Being a postal document, the patients do not have to return to the hospital to provide their information, making it and suitable for large-scale studies.

**References**

2002 Brussels Roundtable Participants; Intensive Care Med; 2003; 29

Needham D, Pronovost et al.; Intensive Care Med; 2005; 31

Lind H, Waldenström AC, Dunberger G et al.; Br J Cancer; 2011; 105(6)

**Grant acknowledgment**

Göteborgs Läkaresällskap.

### A356 One year review of oesophagogastrectomies in Queen Alexandra Hospital Portsmouth

#### F.C. Riccio, D. Pogson

##### Queen Alexandra Hospital, Critical Care, Portsmouth, United Kingdom

###### **Correspondence:** F.C. Riccio – Queen Alexandra Hospital, Critical Care, Portsmouth, United Kingdom

**Introduction** Approximately 2000 oesophagogasterectomies are performed each year, with a 5 year survival of 25 % and 30 day mortality of 10 %^1^. Secondary analysis of ICNARC data has shown the median length of stay to be 2.8 days, and a readmission rate of 12.2 %^2^. We conducted a retrospective review of all post Oesophagogastrectomies ICU admissions in our hospital between Jan 2014-15, as part of a quality improvement project to reduce morbidity and length of stay (LOS).

**Objective** To analyse the electronic record and chest Xray of every oesophagogastrectomy in order to compare our own LOS, patient characteristics and identify factors affecting LOS and unit morbidity.

**Method** We analysed the electronic case record for each open or minimal access oesophagogastrectomy patient. Data was gathered on age, gender, analgesic method, pain scores, analgesia failure, vasopressor use, highest arterial lactate within 24 h, unit LOS and readmission rates.

**Results** 42 patients were identified; 77 % male and median age 64.4. 25/42 (58 %) of patients had a combination of epidural and paravertebral analgesia, 17/42 (40 %) epidural alone and 1/42 (2 %) paravertebral alone. Median pain score on D1 was 1.4 (0-3). There was a 13/42 (30 %) epidural failure rate, 10 disconnections and 3 never effective.

Vasopressors were used on D1 in 28/42 (67 %) patients. Median base excess on D1 was -1 (0-5.1) and median lactate on D1 was 1.93 (0.1-5).

Unit LOS had a range of 3-52 days (median 6 days).

Combinations of collapse and atelectasis were identified in 26/42 (62 %) patients on review of CXR, with one apical pneumothorax.

**Conclusion** Epidural disconnection rate was very high, contributing to a longer unit LOS than national average and higher pain scores. We have introduced a training package on epidural care for our nurses and encouraged the use of tunnelled epidurals in combination with paravertebral catheters.

The incidence of CXR changes after surgery in this cohort has not been studied previously. A large proportion of our patients had radiologically apparent collapse and even consolidation on arrival on ICU. This may contribute to the development of pneumonia in this setting if analgesia is imperfect as CPAP is relatively contraindicated due to the oesophageal anastomosis. We now encourage a longer period in recovery, with lung toilet and recruitment manoeuvres at the end of one-lung ventilation.

**References**

1. Bartels H, Stein HJ, Siewert JR. Preoperative risk analysis and postoperative mortality of oesophagectomy for resectable oesophageal cancer. *Br J Surg* 1998; 85: 840-4

2. Park DP, Welch CA, Harrison DA, et al. Outcomes following oesophagectomy in patients with oesophageal cancer: a secondary analysis of the ICNARC Case Mix Programme Database. *Critical Care* 2009; 13(2):S1 (doi:10.1186/cc7868)

**Grant acknowledgment**

The help of Mr M Lympany, IT support in our department.

### A357 Characteristics and outcomes of critically ill patients undergoing tracheostomy and transferred to the ward in a Brazilian public hospital

#### A.C.P. Antonio^1,2^, A.F. Leivas^1^, F. Kenji^1^

##### ^1^Hospital de Clínicas de Porto Alegre, Porto Alegre, Brazil; ^2^Hospital Moinhos de Vento, Porto Alegre, Brazil

###### **Correspondence:** A.C.P. Antonio – Hospital de Clínicas de Porto Alegre, Porto Alegre, Brazil

**Introduction** Placement of tracheostomy is commonly thought to allow a more secure and manageable airway and to facilitate weaning from mechanical ventilation. However, current literature suggests that tracheostomy has no impact on survival in unselected ICU patients, and it only transfers the mortality from the ICU to the ward. Moreover, in many circumstances tracheostomies are placed in patients who are at the end of their lives with little hope of meaningful recovery.

**Objectives** To describe main characteristics and outcomes of tracheostomized patients discharged from ICU to ward in a public hospital.

**Methods** A retrospective descriptive study was conducted to analyze data from the electronic medical record. The setting was four adult medical-surgical ICUs (44 beds) in a tertiary care public hospital in southern Brazil. Data from 71 adult subjects who underwent a tracheostomy as part of their ICU management and were subsequently transferred to ward were obtained. Individuals who died at ICU during first admission were excluded. Demographic data, diagnoses on admission, comorbidities, duration of mechanical ventilation, ICU length of stay, end-of-life decisions and mortality were recorded.

**Results** From January to December 2015, 104 subjects received tracheostomy. Thirty-two died during their initial ICU admission (30.4 %) and therefore were excluded of analysis. Of the remaining 73 individuals, twenty-eight died (38.3 %), and only four of whom were readmitted to the ICU within 48 hours of discharge. Mean age was 56.9 ± 17.7 years, 52.1 % were male and mean APACHE II score was 21.6 ± 6.8 points. Chronic neurologic disorders and cancer were main comorbidities (21.1 % and 14 %, respectively). Most common diagnosis were sepsis (33.8 %) and neurological emergencies (stroke, intracerebral hemorrhage, meningitis) [23.9 %]. Length of ICU stay was 30.7 ± 17 days and duration of mechanical ventilation was 23 (13 - 29) days. Life-sustaining treatments were withheld or withdrawn in twenty-five decedents. Seven subjects died in posterior hospitalizations at our institution over the period recorded.

**Conclusions** Tracheostomy may represent a burden after ICU discharge, requiring high resource use and low survival rate. Indication for tracheostomy should be cautious, and efforts should be made to recognize patients who might clearly benefit from this technique to avoid unnecessary and unwanted prolonged mechanical ventilation. All the decisions we make in the ICU do have an important impact on future care needs. Knowledge of characteristics and outcomes may assist in identifying interventions to reduce the need for tracheostomy or improve outcomes.

### A358 ICU readmissions and subsequent outcomes: a ten-year retrospective analysis

#### E. James, P. Morgan

##### East Surrey Hospital, Intensive Care, Redhill, United Kingdom

###### **Correspondence:** E. James – East Surrey Hospital, Intensive Care, Redhill, United Kingdom

**Introduction** Readmission to Intensive Care during the same hospital admission has been shown to be associated with a higher risk of mortality (1,2).

**Objectives** To determine the readmission rate over a ten year period and subsequently analyse any effect this may have on mortality, length of ICU stay and whether readmission to ICU requires higher levels of care.

**Methods** Retrospective analysis of our electronic patient information system for admissions from 2005 to 2016. Patients under 18 years old were excluded. Data collected included: mean age, mean stay, proportions of ventilated patients, patients commenced on RRT, unit mortality, hospital mortality and MRSA status. The two patient groups were compared: patients readmitted to ICU within their hospital admission (Group 1), and those patients who were not readmitted (Group 2).

**Results** 7422 patients were identified in the 10-year period; 277 in Group 1 (3.7 %) and 7145 in Group 2 (96.3 %). Mean age in Group 1 was 64 compared to 61 in Group 2. Mean stay was 7.1 days and 5.3 days respectively.

**Conclusions** Patients readmitted to ICU during their hospital stay were at significantly increased risk of dying in hospital, but not ICU itself. MRSA is more likely to be detected in patients who are readmitted to ICU. Further work should be carried out to investigate patients readmitted more thoroughly, with an aim of identifying patients most at risk of readmission and strategies to prevent readmission and improve discharge planning.

**References**

1. Patients readmitted to the intensive care unit during the same hospitalization: Clinical features and outcomes. Chen LM et al. Critical Care Medicine: Nov 1998. Volume 26(11). pp1834-1841

2. Critically ill patients readmitted to intensive care units—lessons to learn? Metnitz PGH et al. Intensive Care Medicine. Feb 2003, Volume 29(2). pp 241-248

**Table 113 (abstract A358).** Results

	Readmitted (Group 1)	Not readmitted (Group 2)	p value (NS = Not significant)
Hospital mortality	100 (36.1)	1889 (26.4%)	0.008
Unit mortality	60 (21.6%)	1351 (18.9%)	NS
Mech. Vent	144 (51.9%)	3433 (48.1%)	NS
RRT	39 (14.1%)	1107 (15.5%)	NS
MRSA	20 (7.2%)	243 (3.4%)	0.002

### A359 Pancreatitis in the west of Scotland intensive care population over a 20 year period

#### G. Carroll, L. Gemmell, A. MacKay, C. Wright, J. Ballantyne

##### Queen Elizabeth University hospital, Anaesthetics and Intensive Care, Glasgow, United Kingdom

###### **Correspondence:** G. Carroll – Queen Elizabeth University hospital, Anaesthetics and Intensive Care, Glasgow, United Kingdom

**Introduction** Pancreatitis is a common precipitant of critical illness and intensive care admission. Mortality from pancreatitis overall should be under 10 % and in severe pancreatitis under 30 % (1). Mortality risk is mulit-factorial but those at high risk are co-morbid, elderly, develop SIRS or progress to pancreatic necrosis. We sought to look at all of our pancreatitis admissions to ICU over a 21 year period and identify the average demographics and difference between survivors and non survivors.

**Objectives** To identify patients admitted to ICU with a primary diagnosis of acute pancreatitis, and to compare predicted demographics and features of systemic inflammatory response between survivors and non survivors. We hypothesised that if you required Intensive Care for the management of severe pancreatitis, death is likely to occur at the beginning of your ICU stay due to overwhelming organ failure. If you were to survive the initial insult, it was hypothesised that you may survive to hospital discharge, although the length of hospital stay would be prolonged. We sought to test this theory with our patient group.

**Methods** A retrospective audit of patients admitted to ICU in the Glasgow Victoria Infirmary, Southern General and Queen Elizabeth University hospital from 1994 to 2015. Patients were identified on Wardwatcher via a search of APACHE II diagnosis including pancreatitis. Data was collected from patient profiles on the Wardwatcher and TrakCare CIS.

**Results** 182 patients were identified with an admission diagnosis of pancreatitis from 12704 patients admitted giving an incidence of 1.4 % of all ICU admissions. Other results are as demonstrated below with with all data being presented as mean and 95 % confidence intervals with p-values from Student´s unpaired t-test where applicable.

**Conclusions** As could be predicted, pancreatitis is a diagnosis of the older male population in ICU, likely as a result of the concomitant problem of alcohol abuse in the West of Scotland. These patients have a higher than normal APACHE-II score and predicted mortality compared with unit averages. All SIRS criteria were met when looking at average data, hence why their likely admission to ICU.

When comparing survivors to non-survivors, survivors were significantly more likely to be younger, with lower APACHE-II scores and predicted mortality. There was no difference in length of stay between groups nor degree of derangement of any of the SIRS criteria.

**References**

1. Young, Thompson. Severe acute pancreatitis. *Cont Ed Anaesthesia Crit Care.* 2008: 8(4): 125-8

**Table 114:** (abstract A359).

	All patients (n= 182)	Survivors (n=95)	Non-survivors (n=87)	p-value
Age (years)	57.4 +/- 2.2	53.8 +/- 3	61.2 +/- 3.1	<0.001
Male gender	58.8%	65.3%	51.7%	0.064
APACHE-II	21.8 +/- 1.1	18.4 +/- 1.2	25.6 +/- 1.7	<0.001
Predicted mortality	51.8 +/- 3.5	41.5 +/- 3.9	63.4 +/- 4.8	<0.001
Length of stay (days)	9.0 +/- 1.6	8.4 +/- 1.6	9.7 +/- 3.0	0.415
WCC in 1st 24hrs	16.5 +/- 1.8	17.4 +/- 3.0	15.5 +/- 1.8	0.298
HR in 1st 24hours (bpm)	130 +/- 3	129 +/- 4	131 +/- 6	0.483
RR in 1st 24hrs	29 +/- 1	29 +/- 2	29 +/- 2	0.943
Temp in 1st 24hrs (oC)	38.1 +/- 0.2	38.2 +/- 0.3	38.0 +/- 0.2	0.391

### A360 The association of intravenous fluid administration on patient outcomes in critical care

#### S. Jonnada^1^, C.S. Gerrard^2^, N. Jones^2^

##### ^1^University of Cambridge, Gonville & Caius College, Cambridge, United Kingdom; ^2^Papworth Hospital, Papworth Everard, United Kingdom

###### **Correspondence:** S. Jonnada – University of Cambridge, Gonville & Caius College, Cambridge, United Kingdom

**Introduction** Fluid resuscitation is a key part of patient management after cardiac surgery. Intravenous (IV) fluids are used to replace lost circulating volume and to maintain adequate perfusion to vital organs, but the volume given to patients varies greatly. In the past, the accepted practice was that the greater the volume of fluids a patient was given, the better their chance of survival. However, recent literature shows that fluid overload could possibly lead to worse outcomes for patients^1,2^ and suggests that clinicians should be cautious when prescribing fluids.

**Objectives** To determine the association between IV fluid administration (FA) and patient outcomes in patients in a cardiothoracic intensive care unit.

**Methods** A retrospective observational analysis was conducted of all of the patients admitted to the cardiothoracic critical care unit (CCU) at Papworth Hospital between 01/01/2014 and 31/12/15. Patient data was extracted from the CCU electronic database and patients with missing data were excluded, leading to the final patient cohort used for the study. Parameters including IV FA, survival, need for a prolonged CCU stay (defined as >72 h), need for prolonged ventilation (PV) (defined as >168 h), need for parenteral nutrition (PN) and AKI score were collected. Patients were split evenly into four quartiles, determined by daily IV FA. Relative risk (RR)and 95 % confidence intervals (CI) were calculated for each adverse patient outcome by comparing each of Quartile 2 (Q2), Quartile 3 (Q3) and Quartile 4 (Q4) to Quartile 1 (Q1).

**Results** 5519 patients were identified initially, but after excluding those with incomplete data (n = 945), a total of 4574 patients were found to be eligible for the study. Patient quartiles contained 1143 or 1144 individuals. A statistically significant association was found between high FA and adverse patient outcomes in patients in Q4 compared to patients in Q1 for mortality (RR = 13.2 (CI 5.3-32.6)), need for prolonged stay (RR = 8.8 (CI 7.1-10.8)), need for PV (RR = 43.0 (CI 16.0-115.4)), need for PN (RR = 16.0 (CI 5.0-51.2)), AKI score (RR = 3.6 (CI 2.9-4.3)) and a maximum AKI score of 3 (RR = 41.2 (CI 5.8-304.4)). These results are in keeping with the findings in other studies^1,2^.

**Conclusions** High FA is associated with increased mortality and morbidity, with the poorest patient outcomes consistently observed in Q4 compared to Q1 across all complications. Being cautious when prescribing IV fluids may help improve patient outcomes in critical care.

**References**

^1^ Pradeep, A., et al. "High volumes of intravenous fluid during cardiac surgery are associated with increased mortality." *HSR Proceedings in Intensive Care and Cardiovascular Anesthesia* 2 (2010): 287-296. 

^2^ Lee, J., et al. "Association between fluid balance and survival in critically ill patients." *Journal of internal medicine* 277.4 (2015): 468-477.

**GRANT ACKNOWLEDGMENT**

N/A.

**Table 115 (abstract A360).** Patients with Adverse Outcomes by Quartile

	Q1	Q2	Q3	Q4
No of patients per group	1143	1144	1143	1144
Median fluid administration per day (ml)	87.9	122.8	157.5	331.1
No of deaths	5	0	9	66
No staying over 72 hours	83	76	136	729
No requiring PV	4	0	0	172
No requiring PN	3	2	5	48
No with AKI (any)	120	68	100	426
No with AKI (stage III)	1	3	1	42

### A361 Early detection of intensive care unit mortality with secondary hyperlactataemia

#### J.D. Salciccioli, D.C. Marshall, M. Komorowski, A. Hartley, M.C. Sykes, R. Goodson, J. Shalhoub

##### Imperial College London, London, United Kingdom

###### **Correspondence:** J.D. Salciccioli – Imperial College London, London, United Kingdom

**Introduction** Lactate is a prognostic biomarker used commonly in intensive care to detect tissue hypoxia. Lactate clearance has been shown to be associated with improved outcomes in patients with sepsis (1) and cardiac arrest (2). Whether elevations in lactate after primary clearance (ie. secondary hyperlactataemia, SH) is associated with outcomes in critically ill patients remains unknown.

**Objectives** We hypothesized that SH is an early marker for ICU mortality and would be independently associated with mortality. In order to test this hypothesis, we performed a retrospective observational study to assess the relationship between SH and mortality.

**Methods** Retrospective analysis of the Multiparameter Intelligent Monitoring Intensive Care III (MIMIC-III) database was performed. MIMIC-III is an open access anonymised database comprising over 58,000 intensive care admissions from 2001-2012. All adult patients demonstrating an initial lactate clearance (LC) to < 2 mmol/L from > 4 mmol/L were included. SH was defined by a rise in lactate > 4 mmol/L following an initial LC. Univariate and multivariable logistic regression analysis was used to test the association between SH and 28-day mortality.

**Results** A total of 3390 patients were included, of which 341 (10 %) showed a SH, the remainder belonging to the LC group. The average age at admission was 63 (+/- 18) years, 58 % males and frequent comorbidities included hypertension, diabetes mellitus and congestive cardiac failure. The median time of initial LC was 15 hours and the delay between SH and death was 83 hours. The 28-day mortality rate in the SH group was 42 % versus 12 % in the LC group. SH was associated with 28-day mortality in unadjusted analysis (OR 5.41, 95 % CI 4.25 - 6.89; p < 0.001). After multivariable adjustments for patient demographics, co-morbid disease, new organ dysfunction and laboratory data, SH remained independently associated with 28-day mortality (OR 4.87, 95 % CI 3.70 - 6.41; p < 0.001).

**Conclusions** In this preliminary study, secondary hyperlactataemia was associated with mortality in critically ill patients. The association remained robust after multivariable adjustments. Secondary hyperlactataemia may serve as an early signal for deterioration in critically ill patients. Future studies should assess additional therapeutic options for patients with secondary hyperlactataemia in the ICU.

**References**

1. Nguyen HB, et al. Early lactate clearance is associated with improved outcome in severe sepsis and septic shock. Critical care medicine. 2004 Aug 1;32(8):1637-42.

2. Donnino MW, et al. Initial lactate and lactate change in post-cardiac arrest: a multi-center validation study. Critical care medicine. 2014 Aug;42(8):1804.

### A362 Cumulative survival rates based on liver transplant indication over 20 years experience in Santiago de Compostela (Spain)

#### J.R. Fernández Villanueva^1^, R. Fernández Garda^1^, A.M. López Lago^1^, E. Rodríguez Ruiz^1^, R. Hernández Vaquero^1^, C. Galbán Rodríguez^1^, E. Varo Pérez^2^

##### ^1^Hospital Clínico Universitario de Santiago de Compostela, Critical Care Unit, Santiago de Compostela, Spain; ^2^Hospital Clínico Universitario de Santiago de Compostela, Abdominal Transplant Unit, Santiago de Compostela, Spain

###### **Correspondence:** J.R. Fernández Villanueva – Hospital Clínico Universitario de Santiago de Compostela, Critical Care Unit, Santiago de Compostela, Spain

809 Liver Transplants (LT) made at Hospital Clínico Universitario de Santiago de Compostela (Spain) in period 1994-2014, 4.25 % of all LT performed in Spain (19005 cases, according to the Spanish Liver Transplant Registry- RETH). Review and comparison of Cumulative Survival Rates based on LT Indication over 20 Years Experience.

**Materials and methods** Retrospective and descriptive study of 809 LT cases performed from 1994 to 2014 at Hospital Clínico Universitario de Santiago de Compostela (Spain) based in our local LT Registration.

**Results** 809 LT cases,12 of which were Hepatorenal Transplantations,35 required a Re-Transplantation. Media of LT:36 LT/year. Gender:79.35 % Male,20.64 % Female,Mean Age of 51 years old.Blood Group:A positive (49 %) and 0 positive (39 %).Most frequent LT Indication Groups:Liver Cirrhosis (LC): 541 cases,Tumors (T):186 cases,Fulminant Liver Failure (FLF):50 cases and Re-Transplant (RLT):35 cases.In the LC Group the most frequent: Alcohol-Related (ARC): 64 %, Hepatitis Virus C Cirrhosis (HVC): 22 %, Primary Biliary Cirrhosis (PBC): 5 % with Cumulative Survival Rate at 20 Years: ARC: 50 %, HCV: 35 % and PBC: 48 %. In T Group the most frequent indication: Hepatocarcinoma (HC): 90 %, Neuroendocrine Tumor (NET): 4 %, Klatskin Tumor (KT): 3 % with Cumulative Survival Rate at 20 Years: HC: 59 %, NET: 50 % and KT: 30 %. In FLF Group: Autoimmune/ Toxic/ Idiopatic (ATI): 72 %, Hepatitis B Virus (HBV): 10 % and Post-traumatic (PT): 6 % with a Global Cumulative Survival Rate at 20 years: 70 % in the total group. In RLT Group: Hepatic Artery Thrombosis (HAT): 35 %, Primary Allograft Dysfunction (PAD): 29 % and Recurrence of Underlying Disease (RUD): 21 % with a Global Cumulative Survival Rate at 5 years (2008-2013): 23 %.

**Conclusions** Liver Chirrosis is the most frequent indication towards LT, followed by Tumors, with Hepatocarcinoma predominance and Fulminant Liver Failure. Cumulative Survival Rate globally is similar to the ones found out in other Transplantation Programs. In Liver Cirrhosis Group, Alcohol-Related Cirrhosis is the most frequent and with better Outcome as well as Hepatocarcinoma int the Tumor Group. Autoimmune/Toxic/Idiopatic is the most frequent in Fulminant Liver Failure Group and has the best Outcome of all LT Indications though, poor Outcome at Retransplant Group leads to a more careful selection of cases to get better Survival Ratios.

### A363 Length of stay of Spinal Cord Injury (SCI) patients and their outcome while in Neuro ICU

#### C. Hilasque

##### St. George's Healthcare Trust, Neuro ICU, London, United Kingdom

**Introduction** Why do patients with Spinal Cord Injury (SCI) stay in Neuro ICU for a long time?

**Objectives** A survey about the reasons why SCI patients stayed longer in NICU between 2013 and 2015 and their outcome

**Methods** A local database (Wardwatcher) was used to search and collection of data for patients admitted with SCI between 2013 and 2015.

**Results** Out of 3,539 admissions in NICU, 326 cases were related to spinal injury and 137 were considered as spinal cord injury and 93 % of admissions were related to trauma and 6 patients were readmitted while in the hospital between 2013 and 2015. There was an increase trend of admissions in NICU between 2013 and 2015 and the data showed that the average length number of days of patient stay was 3.6 days while spinal patients were 8.6 days.

The Guidelines for the Provision of Intensive Care Services (2015) recommends a discharge from critical care to ward must be within 4 hours from the decision of the consultant, the data showed that 71 % of between 9 to 14 hours delayed discharges from NICU to ward are mainly caused by shortage of ward beds followed by delay on ward.

60-66 % of SCI patients required advance cardiac and respiratory organ support and 36 % were on neurological support. 86 % of the patients had tracheostomy.

Only 19 patients were qualified for SCI study called Injured Spinal Cord Pressure Evaluation (ISCoPE) where the intraspinal pressure was measured in relation to traumatic spinal cord injury and this requires the patient to be in the spinal monitoring pressure for at least 7 days.

On patients outcome, 75 % had improved, 13 % died and 12 % of the patients remain unchanged. While the hospital patient outcome on discharge, 83 % lived and 17 % died.

**Conclusions** Though there were not many SCI patients admitted in the unit compared to other patients. SCI patients stayed longer by 5 days on average. Three main reasons were identified why they stayed longer; one was because of ISCoPE research, the other one was their dependency on advance organ support and lastly, the delayed discharges because of shortage of ward beds. The delayed discharges could indicate that the hospital needs to increase the bed capacity.

The patients' outcome appears encouraging with their improved outcome.

A further audit is necessary to see any changes.

**References**

Guidelines for the Provision of Intensive Care Services (2015). http://www.ics.ac.uk/ics-homepage/latest-news/guidelines-for-the-provision-of-intensive-care-services/ (Accessed: 12 April 2015)

Phang, I. and Papadopoulos, M., Intraspinal Pressure Monitoring in a Patient with Spinal Cord Injury Reveals Different Intradural Compartments: Injured Spinal Cord Pressure Evaluation (ISCoPE) Study. Neurocrit Care. 2015 Dec;23(3):414-8. doi: 10.1007/s12028-015-0153-6

## QUALITY AND SAFETY II

### A364 Impact of real time random safety analysis in structure, process and outcome indicators: a multicenter study

#### I. Oliva^1^, G. Sirgo^1^, M.C. Martin^2^, M. Olona^3^, M.C. Gilavert^1^, M. Bodí^1^

##### ^1^Hospital Universitari Joan XXIII, Intensive Care Unit, Tarragona, Spain; ^2^Hospital Universitario de Torrejón, Intensive Care Unit, Madrid, Spain; ^3^Hospital Universitari Joan XXIII, Department of Preventive Medicine, Tarragona, Spain

###### **Correspondence:** I. Oliva – Hospital Universitari Joan XXIII, Intensive Care Unit, Tarragona, Spain

**Introduction** The risk of medical errors is high in intensive care medicine. Errors in healthcare may occur due to an unintended act or by omission. Errors of omission are more insidious and more difficult to identify. Our group previously developed and validated a new tool: the real time random safety audits (in Spanish: Análisis Aleatorios de Seguridad en Tiempo Real, AASTRE). It was effective in detecting and remedying errors of omission in real time.

**Objective** The purpose of this study was to investigate the AASTRE impact in structure, process and outcome indicators through a multicenter study.

**Methods** A prospective study was conducted over a period of 12 months in two adult patient ICUs. Safety rounds were conducted three days a week ascertaining the 37 safety measures (grouped into 10 blocks). In each round, 50 % of the patients and 50 % of the measures were randomized. The impact of this safety tool was analysed in indicators of structure (culture of safety, healthcare protocols), process (improvement proportion related to tool application, IPR-AASTRE) and outcome (mortality, average stay, rate of catheter-related bacteremias and rate of ventilator-associated pneumonia, VAP).

**Results** 1241 patients-day were analyzed. Structure indicators: AASTRE was associated with an increased climate of security and creation / modification of protocols (sedation/analgesia and weaning). Process indicators: 12 of the 37 measures had an IPR-AASTRE > 10 %. Seven mesures had an IPR-AASTRE > 10 % in the three quarters analyzed. Six mesures showed a progressive decrease of the IPR over the study period. Nursing workloads and patient severity on the day of analysis were independently associated with a higher IPR-AASTRE in half of the blocks of variables. Outcome indicators: AASTRE was associated with a significant decrease in the rate of NAV.

**Conclusions** AASTRE was associated with improvement in structure, process and outcome indicators. This tool also improved the care process and adherence to the clinical practice guidelines and proved to be most useful in situations of high care load and in patients with more severe disease.

**References**

Bodí M, Olona M, Martín MC, Alceaga R, Rodríguez JC, Corral E, Pérez Villares JM, Sirgo G. Feasibility and utility of the use of real time random safety audits in adult ICU patients: a multicentre study. Intensive Care Med. 2015 Jun;41:1089-98.

**Grant acknowledgement**

supported by grants from the Fondo de Investigación Sanitaria (Institute of Health Carlos III from Spain, FIS grants, project PI11/02311).

### A365 Cost-effectiveness analysis of a quality improvement bundle to reduce mortality after emergency laparotomy

#### C. Ebm^1^, G. Aggarwal^2^, S. Huddart^2^, N. Quiney^2^, M. Cecconi^3^

##### ^1^WPK Hopsital, Vienna, Austria; ^2^Royal Surrey County Hospital, Guildford, United Kingdom; ^3^St. George's Healthcare Trust, London, United Kingdom

###### **Correspondence:** C. Ebm – WPK Hopsital, Vienna, Austria

**Introduction** Emergency laparotomy is associated with a high risk of mortality and morbidity, which leads to significant financial expenditures for the NHS. In a recent study, the Emergency Laparotomy Pathway Quality Improvement Care (ELPQUIC) program has shown that a set of sequential interventions have the potential to improve clinical outcome; however related costs or savings remain unknown.

**Objective** This economic evaluation aims to evaluate costs and the cost-effectiveness of a clinical pathway for patients undergoing emergency laparotomy, compared to a historical cohort receiving standard care.

**Methods** 299 consecutive patients in the control group were compared with 427 patients directed into a predefined pathway. To assess costs and cost-effectiveness, two decision models were constructed; the first model took hospitals management perspective, the second model took a societal perspective and evaluated lifetime costs and quality adjusted life years.

**Results** One time implementation costs of £23,406.7/hospital (£1,399.0-£31,793.0) for training, supervision, purchase of equipment and pharmaceuticals can be expected. However, these costs were offset after treating 26 patients, mainly due to reduced LOS and lower complication rates. The long-term model showed that the intervention is both more effective (2.4 month) and leads to lower costs to society (cost savings of £899.6/patient). The incremental cost-effectiveness ratio is £-4,015.9, meaning the new pathway is the dominant strategy and should be recommended to decision makers.

**Conclusion** A bundled pathway to improve clinical care for patients undergoing emergency laparotomy has shown to reduce mortality, seems cost-effective and has the potential to improve clinical outcome and lower costs for society. Decision makers need to adopt a long-term vision and be prepared to make one off investments to lower future costs.

**References**

Huddart S, Peden CJ, Swart M, et al. Use of a pathway quality improvement care bundle to reduce mortality after emergency laparotomy. *Br J Surg.* 2015; 102(1): 57-66.

### A366 Delay in the emergency department: does it matter?

#### S.M. Fernandes^1,2^, J. Santos Silva^1^, J. Gouveia^1^, D. Silva^1^, R. Marques^1^, H. Bento^3^, A. Alvarez^1^, Z. Costa Silva^1^

##### ^1^Hospital de Santa Maria/CHLN, Serviço de Medicina Intensiva, Lisboa, Portugal; ^2^Faculdade de Medicina, Universidade de Lisboa, Lisbon, Portugal; ^3^Hospital de Santa Maria/CHLN, Lisboa, Portugal

###### **Correspondence:** J. Gouveia – Hospital de Santa Maria/CHLN, Serviço de Medicina Intensiva, Lisboa, Portugal

**Introduction** Delayed admission from the emergency department (ED) to intensive care unit (ICU) has been associated with increased ICU/hospital mortality, length of stay and total cost of care^1,2^. However, there is little data to support the use of any particular time frame as an indicator of quality of care.

**Objectives** We aimed to investigate the relationship between time in ED and ICU mortality in a tertiary university affiliated hospital.

**Methods** We conducted a retrospective study of patients admitted from 2011 to 2015. We included patients admitted directly from the ED based upon administrative registries Transfers from other hospitals including administrative passages through the ED and admissions after emergency surgery were excluded. Primary outcome was ICU mortality. Secondary outcomes included nosocomial infection, ventilation time and hospital mortality. Statistical analysis included univariate and multivariate logistic regression.

**Results** We included 555 admissions (548 patients) with a mean SAPSII level of 43.0 ± 18.8 and ICU mortality of 19.8 % (n = 110). Mean age at the time of admission was 57.8 ± 18.2y, and 60.0 % were male. Patients were admitted due to medical conditions (78.2 %) and trauma with no need for emergent surgery (21.8 %). Mean time in the ED was 8.4 hours (±5.7), with a skewed distribution to the left. Significantly, there was a significant decrease of ED time during this five year period (-1.9 (-3.5- -0.5, p = 0.009) hours in 2015 compared to 2011). In the univariate analysis, we found no association of time in the ED with ICU mortality (OR = 1.01; p = 0.73). This result was not altered after adjusting for SAPSII, age, gender, trauma, acute kidney injury or year of admission. Of relevance, there was also no association with ICU mortality and an excess of 12 hours in the emergency department. Finally, there was no association between time spent in the ED and hospital mortality (OR = 1.2; p = 0.13), ventilation time (p = 0.6) or nosocomial infection (p = 0.8).

**Conclusions** Unnecessary time spent in the ED has the potential to adversely affect the care and to overwhelm crucial ED resources. In our single-center retrospective study we did not find any association between the absolute time spent in the ED and ICU mortality, and we cannot recommend any clinically relevant specific time frame. Meanwhile, patients should be transferred to the ICU from the emergency department as soon as possible and further research is needed to develop potential quality indicators.

**References**

1. Chalfin DB, et al. Impact of delayed transfer of critically ill patients from the emergency department to the intensive care unit. Crit Care Med. 2007.

2. Cardoso LT, et al. Impact of delayed admission to intensive care units on mortality of critically ill patients: a cohort study. Critical Care 2011.

**Grant acknowledgment**

No grant support.

### A367 The utility of a daily checklist in patients under mechanical ventilation to achieve low tidal volumes

#### D. Díaz Diaz, M. Villanova Martínez, E. Palencia Herrejon, A. Martinez de la Gandara, G. Gonzalo, M.A. Lopez

##### Hospital Universitario Infanta Leonor, Intensive Care Unit, Madrid, Spain

###### **Correspondence:** D. Díaz Diaz – Hospital Universitario Infanta Leonor, Intensive Care Unit, Madrid, Spain

**Objectives** Several studies have demonstrated a decrease of mortality under mechanical protective ventilation with low tidal volumes (6-8 ml per kilogram of predicted body weight) not only in patients with acute respiratory distress syndrome (ARDS), but also in ICU patients without criteria of pulmonary injury, The aim of this study is to evaluate the utility of a daily checklist applied to all the patients under mechanical ventilation (MV) admitted to an ICU as well as to identify the factors influencing the achievement of the goal to keep the tidal volume at 6-8 ml per kilogram of predicted body weight

**Methods** A prospective study including all the patients admitted to an ICU during a four-months study period. A daily-based register was implemented for all the ICU patients under MV during the morning, the afternoon and the night shifts. We analyse the degree of compliance with the checklist, as well as the influence on the achievement of the goal for tidal volume of the following factors: height, sex, type of MV (Continuous Mandatory Ventilation-CMV, Pressure Control Ventilation-PCV, Bi-level positive airway pressure-BiPAP, Synchronized Intermittent-mandatory Ventilation-SIMV, Pressure Support Ventilation-PSV), gaseous exchange (relation paO2/FiO2), pulmonary mechanics (plateau pressure).

**Results** We registered 883 measurements (36 % in the morning shifts, 31 % in the afternoon and 33 % in the night shift) for all the patients with MV (either with ARDS or without criteria of pulmonary injury). No patient developed ventilator induced lung injury (VILI). The percentage of measurements outside the established goal of tidal volume was 28,7 % (CI 95 % 23.9-33.9 %) without differences between the shifts. The average tidal volume was of 443.9 ml (range 431-457) that corresponds to 7.5 ml per kilogram of predicted body weight (IC 95 %: 7,35-7,66). In univariate analysis the factors associated to being outside of the goal for tidal volume were: female sex, height and the Pressure Support Ventilation-PSV as opposite to mandatory modes with no influence of the shifts of work, the relation paO2/FiO2 and the plateau pressure. The multivariate logistic regression analysis showed that the factors independently associated with being outside of the goal for tidal volume were female sex and the Pressure Support Ventilation-PSV.

**Conclusions** In almost 30 % of the ICU patients under MV the goal of low tidal volumes is not achieved, especially in female patients and in those under Pressure Support Ventilation. The establishment of a daily checklist for patients under VM is feasible and could influence in a low frequency of VILI in patients with ARDS and also in those without criteria of pulmonary injury.

### A368 Analysis of the serious adverse events reported at the heart surgery intensive care unit

#### P. Ruíz de Gopegui Miguelena, C.I. Bernal Matilla, P. Sánchez Chueca, M.D.C. Rodríguez Longares, R. Ramos Abril, A.L. Ruíz Aguilar, R. Garrido López de Murillas

##### Hospital Universitario Miguel Servet, Medicina Intensiva, Zaragoza, Spain

###### **Correspondence:** P. Ruíz de Gopegui Miguelena – Hospital Universitario Miguel Servet, Medicina Intensiva, Zaragoza, Spain

**Introduction** Fostering a safety culture is one of the most important landmarks when it comes to provide quality assistance in an Intensive Care Unit (ICU). The detection and analysis of the incidents and adverse events that affect our patients are the cornerstone for the development of programs and protocols leading to safe health assistance with high standards of quality.

**Objectives** Knowing the nature, casuistry and the underlying elements of adverse events (AE), rated as serious, according to the Protocol on Adverse Events Reporting established in our Unit.

**Methods** 266 notified EA, reaching 201 patients, were analysed through the Adverse Events Reporting System (AERS) established in the Heart Surgery Intensive Care Unit (HSICU) from January 2014 to January 2016. Among these, we rated as serious those that caused to the patients a temporary or permanent damage, extending their hospitalization, compromising their lives or needing surgery in order to save their lives and contributing or causing their demises. In our AERS, those rated as F-I according to the catalogue of the National Coordinating Council for Medication Error Reporting and Prevention (NCCMERP).

**Results** 21 AE rated as serious were notified. Among these, 13 were rated H category (incident that compromised the patient's life and needed care to keep him alive) and 2 were rated I category (the incident contributed or caused the patient's demise). The last were related to surgery complications. All of them needed medical attention, being the majority of them discharged to ordinary ward hospitalization. There were not relevant differences as far as the urgency of the hospitalization, the clinical profile and the assistance needed in the moment of the AE. In 30 % of the AE, the equipment and available resources were pointed out as contributing factors; in 35 % of cases it was related to formation and training; in 40 % of cases it was due to elements related to the patient and in 45 % of the cases it was elements related to the performance. The most frequent serious safety issues were surgery complications or damages related to invasive procedures, three of them related to the handling of breathing devices and two of them with reaction after blood transfusions. In 75 % , according to professional advice, the AE was deemed avoidable.

**Conclusions** The AE rated as serious were 7.7 %. The most common feature ,was their avoidability and their relation with formation and training in the techniques used during the assistance. Beyond the cases of death, the serious AE involved an extension in the stay at the hospital and a rise in the morbidity and mortality rate. According to this, the intervention over factors capable of improvement, such as formation and training of all the professionals involved in the healing process, is compulsory.

**References**

Merino P, et al. Adverse events in Spanish intensive care units:

the SYREC study. Int Qual Health Care. 2012 Apr; 24(2):105-113.

**Grant acknowledgment**

None.

### A369 Evaluation of the influence of an adequate briefing on reducing adverse effects in an intensive care unit

#### R. Fernández Fernández^1^, P. Morales Laborías^2^, M.A. Díaz Castellanos^2^, M.E. Morales Laborías^2^

##### ^1^Hospital Comarcal Santa Ana, UCI, Motril, Spain; ^2^Hospital Comarcal Santa Ana, Motril, Spain

###### **Correspondence:** R. Fernández Fernández – Hospital Comarcal Santa Ana, UCI, Motril, Spain

**Introduction** A well-defined briefing can help to improve the exchange of information among professionals in the shift change and strengthens the safety culture.

**Objectives** General: To promote safety culture in the ICU of the HGB of Motril.

Specific: Encourage communication between the interprofessional team. Diminish the appearance of adverse effects (EAs). Implement use of safety briefings to increase patient safety. Since 2008 daily meetings are held in the morning shift among medical and nursing professionals, and among nurses on each shift, for exchange of information and communication regarding patients admitted, assessing the risk of occurrence of AEs each patient.

**Methods** Description of briefing protocol designed in 2015 in our ICU wich has 8 beds with a total income of 582 in 2015. The personnel that make the interprofessional team are 7 doctors, 14 nurses and 10 assistants.In 2014 a total of 18 EA were reported 33.33 % being derived from a care or clinical procedure, 22.22 % resulting from an accident patien, 16.67 % of medical equipment and 11.11 % patients´falls. The rest was due to other causes.A working group was created to develop and promote safety culture. In November 2015 implementing security briefing was performed after consensus of all nursing professionals.

The variables were entered in the checklist (yes/no) three times a day (in the morning, in the afternoon and at night) and were: mechanical ventilation, vasoactive drugs, fasteners in the weaning, pressure ulcers, drains, revision infusion rate, review alarms, repositioning, mobilization endotracheal tube, nasogastric tube and urine probe, wristband. Mandatory measures of pneumonia Zero program were included: Ambu review and aspirator, strict hand hygiene before handling air, headboard position 30°-40°, check neumotaponamiento pressure, chlorhexidine mouthwash.

**Results**

- Regulated exchange of information among professionals.
- The number of EA reported has decreased by 60 % since the introduction of the use of the new briefing protocol.
- Briefing accepted by 100 % of the professionals.
- 97 % of staff completed the checklist successfully.

**Conclusions** It has reduced the number of adverse effects in more than a half, thus we have demonstrated the effectiveness of the protocol, it is also very important that it has been accepted by almost all of the staff, this data shows that it is simple and easy to carry out.

With these data we have sensitized the staff of the importance of information for patient safety. We think that with this work we can help other units.

**Grant acknowledgment**

To ICU stafffor making this work possible.

### A370 Daytime activation of rapid response team might improve patient safety

#### J. Cho^1,2^, J. Kim^1^, J. Park^1^, S. Woo^2,3^

##### ^1^Inha University Hospital, Pulmonary and Critical Care, Incheon, Republic of Korea; ^2^Inha University, Internal Medicine, Incheon, Republic of Korea; ^3^Inha University Hospital, Cardiovascular Medicine, Incheon, Republic of Korea

###### **Correspondence:** J. Cho – Inha University Hospital, Pulmonary and Critical Care, Incheon, Republic of Korea

**Introduction** Rapid response systems (RRS) may slightly reduce cardiac arrest in the hospital. RRS has an efferent limb such as rapid response team (RRT) and an afferent limb that has a process of timely detection and activation. RRS activates mostly 24 hours/day and 7 days/week by a multidisciplinary team. In middle-income country, unlike EU and USA the personal resources are limited. RRT call were most common during working hours in Australian study^1^.

**Objectives** Daytime activation of RRT may reduce cardiac arrests rates.

**Methods** From October 2015, Inha university hospital rapid response team (INHART) had organized and activated. The INHART consisted with three doctors (two were pulmonologist, one was cardiologist) and one nurse who has experienced in intensive care unit. The activation time was from 9 AM to 5 PM. We collected the numbers of hospital discharge, cardiac arrest events (CAE) retrospectively for 2 years (before INHART) and prospectively from October 2015 to February 2016 (after INHART). CAE per 1,000 discharge were compared between before and after INHART. Nonparametric test was performed.

**Results** Mean CAE per 1,000 discharge were 4.03 before INHART and 2.93 after INHART (p = 0.041). CAE was reduced by 27 % after INHART.

**Conclusions** Despite of short-term operation, daytime only activation of RRT reduced cardiac arrests events. After long-term application, RRT would ameliorate cardiac arrests and improve patient safety in a general hospital.

**References**

1. Jones D. The epidemiology of adult Rapid Response Team patients in Australia. Anesth Intensive Care 2014; 42:213-219

**Grant acknowledgment**

Inha University Research Grant.

### A371 A survey of patient safety culture across two critical care units

#### T. West^1^, E. Powell^2^, A. Rimmer^2^, C. Orford^2^, N. Jones^2^, J. Williams^2^

##### ^1^University Hospital of Wales, Anaesthetics, Cardiff, United Kingdom; ^2^Royal Gwent Hospital, Newport, United Kingdom

###### **Correspondence:** T. West – University Hospital of Wales, Anaesthetics, Cardiff, United Kingdom

**Introduction** Patient safety in the ICU is the prevention of injury or harm arising as a result of the process of care, rather than as a result of the underlying disease process. The incidence of medical errors and adverse events in ICU is surprisingly high^1^.

Systems to improve patient safety, such as checklists, are only effective when implemented within a receptive and positive safety climate. A positive safety climate is associated with a measurable decrease in error rates^2^.

**Objectives** To evaluate the safety climate across both critical care units within Aneurin Bevan University Health Board (ABUHB).

**Methods** We conducted an anonymous online survey across all staff in critical care within ABUHB. The survey consisted of questions validated by the Agency for Healthcare Research and Quality, part of the United States Department of Health and our own questions regarding staff's incident reporting behaviour. We ran the survey for a month and aimed to get a 50 % return rate from the 180 staff working across both sites.

**Results** We received 114 responses and 101 completed surveys from across the multi-disciplinary team.

Eighty eight percent rated overall patient safety as very good or excellent.

The survey reported a supportive environment (83 %) where people worked well together as a team (97 %), and treated each other respectfully (90 %).

Generally patient safety was not sacrificed to get more work done (66 %), and there was no pressure to take shortcuts to speed up work rate (85 %).

"Crisis mode" was precipitated by an inability to discharge wardable patients (52 %), rather than lack of staff (27 %), or the unit being too full (33 %).

Staff would generally discuss adverse incidents amongst themselves informally (85 %), and in many cases (63 %) implement a solution following this discussion.

Feedback as a result of adverse event reports was highlighted as a deficiency with only 33 (31 %) staff feeling they received this, similarly 39 (37 %) of staff did not feel they were adequately informed about any errors that happened on the units.

**Conclusions** Whilst our results indicate a positive work environment, there are areas for improvement, specifically feedback following adverse events.

Designated nurses to attend morbidity and mortality meetings and subsequent publication of patient safety bulletins will improve feedback to nursing staff.

We are going to instigate a designated patient safety board to publicise patient safety events and resultant changes in practice.

Little is currently published regarding UK ICU safety surveys to allow benchmarking of our results. The authors feel UK ICUs should consider collection and publication of this data.

**References**

1 ICS, FICM. Guidelines for Provision of Intensive Care Services. 2015. 1^st^Edition. 167-171.

2 Valentin A, Schiffinger M, Steyrer J et al. Safety climate reduces medication and dislodgement errors in routine intensive care practice. *Intensive Care Medicine* 2013;**39**: 391-398.

### A372 Experience of a report protocole on incidents and adverse events in a heart surgery intensive care unit

#### C.I. Bernal Matilla, P. Ruiz de Gopegui Miguelena, P. Sánchez Chueca, R. Ramos Abril, M.D.C. Rodríguez Longares, A.L. Ruíz Aguilar, R. Garrido López de Murillas

##### Hospital Universitario Miguel Servet, Medicina Intensiva, Zaragoza, Spain

###### **Correspondence:** C.I. Bernal Matilla – Hospital Universitario Miguel Servet, Medicina Intensiva, Zaragoza, Spain

**Introduction** Clinical safety has become on one side a quality standard and on the other side an ethical obligation in the daily work of an Intensive Care Unit (ICU). The establishment of standardized protocols and appropriate training in the area of safety have become a must when it comes to its development. Also all professional categories have to be involved in this process.

**Objectives** Analysis of the incidents and adverse events (I/AE) reported after the establishment of a report protocol (RPIAE) in a Heart Surgery Intensive Care Unit (HSICU).

**Methods** The Clinical Safety Group of the Intensive Care Service, responsible for the design of the RPIAE, was created in January 2014. Thus, the RPIAE was introduced in the HSICU in March 2014. This methodology reviews the adverse events (AE) detected in every single patient that was looked after in this unit between March 2014 and October 2015.**AE**: non-intentional damage caused during or as a consequence of the medical attention received and non-related to the evolution or eventual complications of the initial illness. The information was gathered in an Excel data base and the statistical analysis was performed through the SPSS and Epidat programs.

**Results** 254 AE's were reported on 189 patients, 67 % of which were males. 2.7 % of them were admitted to hospital as a result of an AE. 90.9 % were discharged and 23 patients passed away. 50 % of them were reported by the nursing staff; 39.8 % by doctors. 88.9 % took place in the Intensive Care Unit., 44.9 % of which during the morning shift. 79.9 % while the Unit was holding 100 % occupancy. 24 % of the AE affected the patient in a way that they needed further monitoring and/or intervention in order to check that no damage had been caused. 7.1 % compromised the patient's life and 1.6 % contributed or caused the demise of the patient. 57.9 % of them were doubtlessly avoidable. 22.8 % had their origin in the unplugging/removal of accesses/probes/catheters/sensors/tubes and 7.9 % had their origin in the handling of breathing devices and mechanical ventilation. In 43.75 % of the cases the lack of training and formation had direct effect on the I/AE. The mean duration of hospitalization stay before the AE was 7.68 days (statistic standard deviation, SSD, 30.1); the mean duration of hospitalisation was 16.45 days (SSD 25.26) and the mean stay in the Intensive Care Unit 8.69 days (SSD 13.48), (p < 0.05).

**Conclusions** The majority of the AE were deemed evitable and neither had they important effects on the evolution of the patient nor extended the patient's stay in the ICU. A longer stay in the ICU was related to a bigger incidence of AE. (p < 0.05). Formation and training are key elements in order to avoid AE and they are part in the measures currently established in our ICU in order to avoid these I/AE.

**References**

Merino P, et al. Adverse events in Spanish intensive care units: the SYREC study. Int Qual Health Care. 2012 Apr; 24(2):105-113.

**Grant acknowledgment**

None.

### A373 Reliability of clinical impact grading by health professionals of common prescribing errors and optimisations in critical care patients

#### R.S. Bourne^1^, R. Shulman^2^, M. Tomlin^3^, G.H. Mills^1^, M. Borthwick^4^, W. Berry^5^

##### ^1^Sheffield Teaching Hospitals NHS Foundation Trust, Sheffield, United Kingdom; ^2^University College London Hospitals NHS Foundation Trust, London, United Kingdom; ^3^Southampton University Hospitals NHS Trust, Southampton, United Kingdom; ^4^Oxford University Hospitals NHS Foundation Trust, Oxford, United Kingdom, ^5^Guy's and St Thomas' NHS Foundation Trust, London, United Kingdom

###### **Correspondence:** W. Berry – Guy's and St Thomas' NHS Foundation Trust, London, United Kingdom

**Introduction** Medication errors are common in critically ill patients, [1] and have the potential to adversely affect patient safety [2]. Whilst adverse events, including those caused by medications, have a direct relationship with patient outcomes, [3] the potential clinical impact of individual medication errors remain less clear. Moreover, the complexity of critically ill patient care means frequent optimisation of medications is required to ensure effective therapy. Currently the clinical impact grading of medication errors and optimisations remains subjective in clinical practice and research methodologies.

**Objectives** To identify the reliability of clinical impact grading by health professionals

(i) between professions (inter-rater) and
(ii) within professions (intra-rater)) of common prescribing errors and optimisations in critical care patients; and
(iii) identify representative clinical impact grades for each example.

**Methods** 50 representative medication error and 55 optimisation cases from the PROTECTED UK study, [4] were sent electronically to a purposive sample of 30 healthcare professionals (10 ICU consultants, specialist pharmacists and specialist nurses) in 5 UK sites. Each rater graded the error or optimisation case for severity of clinical impact using a 5-point categorical scale (*no*, *minor*, *moderate*, *severe* and *life threatening/saving* clinical impact). Inter-rater reliability was tested using a linear mixed model. Intra-rater reliability was tested using the intra-class correlation (ICC) with a two-way random effects model using absolute agreement.

**Results** The majority of errors and optimisations (both 76 %) had a modal clinical severity grade of *moderate* or higher. Error cases: doctors graded clinical impact significantly lower than pharmacists (-0.25; p < 0.001) and nurses (-0.53; p < 0.001), with nurses significantly higher than pharmacists (0.28; p < 0.001). Optimisation cases: doctors graded clinical impact significantly lower than nurses and pharmacists (-0.39 and -0.5; p < 0.001 respectively). There was excellent intra-reliability (ICC) grading for pharmacists (0.882 & 0.887; p < 0.001) and doctors (0.794 & 0.829; p < 0.001)) but only fair to good for nurses (0.429 & 0.739; p < 0.001), for optimisations and errors respectively.

**Conclusions** Over one hundred common prescribing errors and optimisations had modal clinical impact grades recorded for potential application in clinical practice and research. The inter-professional variability highlights the importance of multidisciplinary perspectives in assessment of medication errors and optimisations in clinical practice and research.

**References**

1. Rothschild JM, et al. Crit Care Med 2005; 33:1694-700.

2. Manias E, et al. Br J Clin Pharmacol 2012; 74:411-23.

3. Garrouste-Orgeas M, et al. Am J Resp Crit Care Med 2010; 181:134-42.

4. Shulman R, et al. J Crit Care 2015; 30:808-13.

**Grant acknowledgment**

Supported by Sheffield Hospitals Charity (Registered Charity Number 1059043).

### A374 Incidence of adverse events in adult intensive care unit patients

#### D. García Huertas, F. Manzano, F. Villagrán-Ramírez, A. Ruiz-Perea, C. Rodríguez-Mejías, F. Santiago-Ruiz, M. Colmenero-Ruiz

##### Complejo Hospitalario de Granada, Granada, Spain

###### **Correspondence:** D. García Huertas – Complejo Hospitalario de Granada, Granada, Spain

**Introduction** Adverse events (AE) are frequent in patients admitted in intensive care unit (ICU) for different causes.

**Objective** To determine the incidence, characteristics and results of adverse events in critically ill patients requiring mechanical ventilation (MV).

**Methods** An observational, prospective and single-center study, conducted in a medical-surgical ICU over a period of 12 months (2015), in patients who require MV > 24 hours. The variables studied were: demographic data, extubation unscheduled, severe obstructions airway, cardiac arrest, atelectasis, loss of medical devices, septic shock, duration MV, ICU and hospital stay, and ICU and hospital mortality. Statistical analysis: descriptive, bivariate (chi ^2^ and t-Student) and multivariate logistic regression analysis.

**Results** 330 patients were included, with APACHE II score 23.5 ± 7, age 61 ± 15 years, body mass index 28 ± 5, total SOFA on day 1, 8.9 ± 3.5 pts, and 67 % were males. Renal replacement therapies were 14.6 %, duration of ICU stay 17 ± 18, hospital stay 26 ± 22 days, duration of MV 10.8 ± 11 days, cardiac arrest 4.9 %, septic shock 39.8 %, airway obstructions 33.4 %, unscheduled extubation 9.1 %, loss of medical devices 8.25 %. ICU mortality was 33.7 % and hospital mortality 38.6 %. Total incidence of AE was 42.2 %. Predictive factors associated with hospital mortality were APACHE II score (OR 1.048, 95%CI 1.01 to 1.08), septic shock (OR 1.27, 95%CI 0.76 to 2.11), airway obstruction (OR 0.81, 95%CI 0.49 to 1.35), unscheduled extubation (OR 1.80, 95%CI 0.73 to 4.45), and loss of medical devices (OR 1.60, 95%CI 0.65 to 3.9).

**Conclusion** The occurrence of AE in critically ill patients is high. Strategies to reduce the incidence of adverse events may eventually improve outcomes in these patients.

### A375 Development of patient-reported domains describing quality of life after sepsis

#### C. König^1,2^, B. Matt^1^, A. Kortgen^1^, C.S. Hartog^1,2^

##### ^1^Jena University Hospital, Department of Anesthesiology and Intensive Care Medicine, Jena, Germany; ^2^Jena University Hospital, Integrated Research and Treatment Center for Sepsis Control and Care (CSCC), Jena, Germany

###### **Correspondence:** C. König – Jena University Hospital, Department of Anesthesiology and Intensive Care Medicine, Jena, Germany

**Introduction** Sepsis survival rates have improved in recent years and it becomes more and more important to asses long-term outcomes in order to align treatment with what is important for patients. Quality of life (QoL) is an important patient-reported outcome (PRO) that is commonly used as an endpoint in clinical studies. However, the most frequently used instruments, eg. SF-36 and EuroQoL-5D, were developed without input from intensive care survivors and may thus lack validity in these populations. Quality of life domains which matter to sepsis survivors have not yet been elicited.

**Objectives** Identification of the domains of quality of life of sepsis survivors according to their own perception and priorities.

**Methods** Open-ended face-to-face or telephone interviews were conducted with sepsis survivors from interdisciplinary ICUs of a German university hospital. Interviews were transcribed verbatim with a fixed set of transcription rules and analyzed qualitatively with the interpretative phenomenological analysis (IPA) approach. Codes and subcodes were applied to the data and a codebook was developed. A second experienced researcher unfamiliar with the data evaluated the codebook; differences were solved by discussion. Codes were clustered into domains and evaluated for their importance by stakeholders (survivors, relatives of survivors, intensive care physicians, nurses, psychologists, rehabilitation physicians) through a Delphi process.

**Results** Fifteen participants (7 female, 8 male) had a mean age of 62 years (range: 27 - 87 years) with mean time after onset of sepsis of 11 months (range: 5 - 40 months). Mean interview duration was 68 minutes (range: 34 - 95 minutes). The final codebook comprised 16 codes and 99 subcodes; data saturation was already 95 % after 7 interviews. Initial agreement between the two researchers about the codebook was 83 % and could be improved to 100 % after minor changes. 10 core domains of quality of life could be identified. The most relevant domain for sepsis survivors' quality of life is reintegration to normal living. Survivors reported that their quality of life depended more on how much they could participate in everyday life and less on each single persisting physical and psychological deficit. Other domains include: ability to walk, fatigue, family and partnership, self-perception and control over one's life.

**Conclusions** This study highlights the domains that are important for the quality of life of sepsis survivors as assessed by the survivors' own perception. Identified domains are only partly assessed by commonly used quality of life instruments. Reintegration as the most important domain from a patient' perspective is a novel concept in the context of quality of life after sepsis. These domains will help to improve quality of life questionnaires which provide patient-relevant outcomes measures.

**Grant acknowledgment**

Federal Ministry of Education and Research (BMBF), Germany, grant number 01EO1502.

### A376 Chest drains: is the triangle of safety really safe? An ultrasonographic study of enshrined practice to improve patient outcome and safety

#### A. Wong, C. Balan, G. Barker

##### Oxford University Hospitals NHS Trust, OCCULAR Group, Adult Intensive Care Unit, Oxford, United Kingdom

###### **Correspondence:** A. Wong – Oxford University Hospitals NHS Trust, OCCULAR Group, Adult Intensive Care Unit, Oxford, United Kingdom

**Introduction** Intercostal chest drain (ICD) insertion is a relatively common procedure but can be associated with significant complications including bleeding and organ perforation. Indications for insertion include management of hemothorax, pneumothorax and pleural effusions. The "triangle of safety (TOS)" is an often quoted site as being appropriate for ICD insertion; including in trauma patients by ATLS. The space is delineated by lateral border of the pectoralis major, lateral border of the latismus dorsi, line of the 5th intercostal space and the base of the axillaintercostal space. Although ultrasound (US) guidance has been recommended to aid insertion, its use is far from established practice and landmark techniques and the TOS is still widely practiced.

**Objectives** To establish the safety profile of the TOS through the use of US to delineate underlying anatomy including the position of intercostal vessels.

**Methods** 50 consecutive patients on a general ICU underwent bilateral US examination of their TOS. The position of liver, spleen and heart in the respective TOS was noted. Doppler ultrasound was used to identify intercostal vessels within the TOS.

**Results** Overall, the heart, liver and spleen were visible in 60 % of patients within the TOS. This percentage increased in the intubated and ventilated patient population. Intercostal vessels where visible in the minority of patients.

**Conclusions** The British Thoracic Society and the National Patient Safety Agency UK has recommended ultrasound before inserting a drain for fluid. Our study found that the TOS is a misnomer exposing the patient to the risk of underlying organ perforation especially in the patient who is intubated and ventilated. It is therefore not safe and the practice should be abandoned. The routine use of real-time US to guide ICD insertion should be recommended to improve procedural safety.

**References**

1. Havelock T, Teoh R, Laws , et al. Pleural procedures and thoracic ultrasound: BTS pleural disease guideline 2010. Thorax 2010;65(Suppl 2):61-76.

2. National Patient Safety Agency. Rapid response report, chest drains: Risks associated with insertion of chest drains (2008) http://www.nrls.npsa.nhs.uk/resources/patient-safety-topics/medical-device-equipment/?entryid45=59887&p=2

## SEPSIS THERAPEUTICS

### A377 The immunomodulation effect of Polymyxin-B Hemoperfusion in severe sepsis/septic shock: a randomized controlled trial

#### N. Srisawat, S. Peerapornratana, P. Laoveeravat, S. Tachaboon, S. Eiam-ong

##### Chulalongkorn university, Medicine, Bangkok, Thailand

###### **Correspondence:** N. Srisawat – Chulalongkorn university, Medicine, Bangkok, Thailand

**Introduction** Severe sepsis and septic shock are still the common cause of death in ICU. The extracorporeal blood purification therapy could provide a benefit in reduction the inflammatory mediators and bacterial endotoxins.

**Objectives** To study the immunomoudation effect of Polymyxin-B Hemoperfusion (PMX) in severe sepsis/septic shock

**Methods** The adult patients with severe sepsis/septic shock in intensive care units (ICUs) of King Chulalongkorn Memorial Hospital were tested with endotoxin assay. The patients with blood endotoxin level ≥0.6 were randomized into two groups, PMX hemoperfusion group and standard treatment group. The 2-hour PMX hemoperfusion were done in two consecutive days in PMX hemoperfusion group. Data for hemodynamic parameters, Sequential Organ Failure Assessment (SOFA) score, and blood sample were collected on first three day after enrollment The primary outcomes were leukocyte expression of CD11b, HLA-DR, chemotaxis function and blood endotoxin level on day 3 after enrollment. The secondary outcomes were 28-day survival, APACHE II score, change in SOFA score, CVS SOFA score.

**Results** We have enrolled 36 participants into the study, 18 patients for each group. The age, APACHE II score, endotoxin level were comparable between two groups. The percent change of EAA was -19.8 % vs -10.6 % in PMX group and control group, respectively. The APACHE II and total SOFA score was -14.8 % vs +1.5 % and -12.8 % vs +6.1 %, in PMX group and control group, respectively. The CVS SOFA score was -31.4 % vs +24.9 %, in PMX group and control group, respectively. There was the trend increase in HLA-DR expression in PMX group and no change in control group.HLA-DR expression on day 3 in PMX group was significantly higher than control group, 34.5 % vs 24.2 %, p = 0.03. 28-day survival rate was comparable between two groups.

**Conclusions** The PMX hemoperfusion seemed to improve hemodynamic instability and improved immunoparalysis status in severe sepsis/septic shock patients.

### A378 Early exercise in patients with sepsis syndromes improves tissue oxygenation

#### J. Paratz^1,2^, G. Kayambu^1^, R. Boots^1^

##### ^1^University of Queensland, Brisbane, Australia; ^2^Griffith University, Brisbane, Australia

###### **Correspondence:** J. Paratz – University of Queensland, Brisbane, Australia

**Introduction** Sepsis can induce rapid proteolysis and early exercise is desirable to prevent loss of muscle mass. It is not known whether early intervention is safe in critically ill patients with sepsis. Exercise may also act to recruit the microcirculation.

**Objectives** To investigate the effect of 30 minutes of electrical muscle stimulation and passive movements to major muscle groups on the microcirculation in patients in the first 48 hours of sepsis.

**Methods** Changes in tissue oxygenation were assessed at baseline and immediately post exercise using a] a near infrared oxygenation device on the thenar eminence to assess percentage of oxygen in the muscle (StO_2_) and percentage of muscle oxygen extraction rate (MOER%) under both baseline and hyperaemic conditions. B] an orthogonal polarization spectral imaging device was placed sublingually to measure the microvascular flow index and capillary density of the microcirculation.

**Results** All 20 patients completed the trial. There were significant effects post exercise on StO_2_ with hyperaemia [63.0 (±3.3)% to 66.2 (±5.3)%, p = 0.02], and an improvement in capillary density [2.45 (±1.01)% to 3.85 (±1.02)%, p = 0.04]. There were no adverse effects.

**Conclusions** Early exercise appears to have a beneficial effect on critically ill patients as tissue oxygenation improved. There was evidence of recruitment of the microcirculation.

**References**

Kayambu G, Boots R and Paratz J A Prospective Randomised Controlled Trial investigating Functional and Physiological Outcomes following Early Rehabilitation in Sepsis: The i-PERFORM Trial - Protocol Article, *BMC Anesthesiology* in press 2011 Oct 31;11:21

**Grant acknowledgement**

Intensive Care Foundation, Australia.

### A379 Methylene blue effectiveness as contributory treatment in patients with septic shock

#### M.F. Aguilar Arzapalo

##### SSA UADY, Mérida, Mexico

**Introduction** Generalized vasodilation with nonresponding hypotension is present in half death cases due to septicaemia. Methylene blue could be used as a valuable complement in refractory hypotension treatment. with septic shock.

**Objectives** The aim of this study was to determine the effectiveness of methylene blue as contributory treatment in patients.

**Methods** A controlled, randomized, double blinded, clinical trial was performed. 60 patients were divided in two groups. A Group received a single dose of methylene blue calculated 2 mg/kg per body weight diluted in 100 cc of 5 % dextrose infused in 60 min. and C Group, (control) received 100 cc of 5 % dextrose infused in 60 min. Basal measurements of study variables were taken (MBP, lactate, base deficit, central venous saturation and CO2 delta) prior blue methylene administration and every hour afterwards, until MBP >65 mmHg without vasopressor or 72 hours passed after shock began. Data about total noradrenaline dose in mg, length of stay, mechanical ventilation length and mortality was recorded.

**Results** MBP increased progressively first 6 hours after methylene blue infusion in A Group 22 % and C Group 9.2 % (p:< 0.05), steadily until 72 hour follow up. Noradrenaline dose decreased in the first 6 hours, on A Group an 86 %, C Group was 56 % (p:< 0.05). Lactate clearance first 6 hours was 62 % in A Group, in contrast with C Group with 33 % clearance (p:< 0.05). Mortality at ICU discharge on A Group was 20.0 % and C Group was 36.6 % (p: < 0.05) without variation at 21 days.

**Conclusions** Methylene blue is effective as contributory in septic shock treatment.

**Note:** This abstract has been previously published and is available at [2]. It is included here as a complete record of the abstracts from the conference.

**References**

1. Edmund S, Kwok M, Daniel H. Use of Methylene Blue in Sepsis: A Systematic Review. Journal of Intensive Care Medicine 2006; 21(6):359-363. 2. Alderton W, Cooper C, Knowles G. Nitric Oxide synthases: structure, function and inhibition. Biochem. J. 2001; 357:593-615.

2. Lopez V, Aguilar Arzapalo M, Barradas L, Escalante A, Gongoro J, Cetina M (2016) Methylene blue effectiveness as contributory treatment in patients with septic shock. Critical Care 20(Suppl 2): 94.

**Grant acknowledgment**

Hospital "Agustin O´Horan"

**Fig. 140 (abstract A379).**
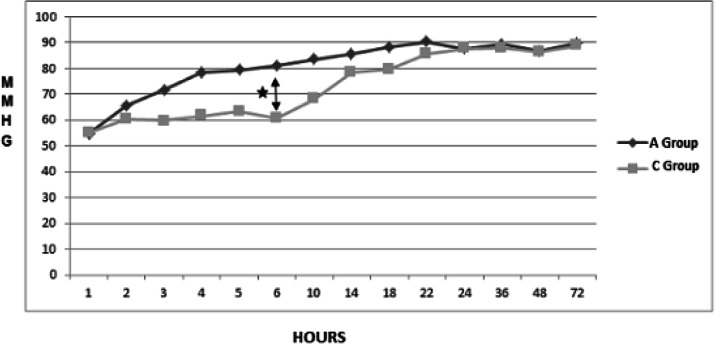
Mean pressure in the two groups

.

### A380 Elimination of cytokines from blood of septic patients with lps adsorber

#### R. Vlasenko^1^, E. Gromova^1^, S. Loginov^2^, M. Kiselevskiy^1^, Y. Dolgikova^1^

##### ^1^NN Blokhin Russisn Cancer Research Center, Moscow, Russian Federation; ^2^Moscow Botkin Clinic, Moscow, Russian Federation

###### **Correspondence:** R. Vlasenko – NN Blokhin Russisn Cancer Research Center, Moscow, Russian Federation

**Introduction** In clinical practice LPS adsorbers are used on the method of extracorporal detoxification to remove the excess of LPS from the blood of the patients with sepsis. In recent years, there is evidence of the ability of the hemosorption columnsto eliminate cytokines and inflammatory mediators that trigger a cascade reaction of irreversible pathological changes in organs and tissues of the patient [ 1].

**Objectives** Study of elimination of proinflammatory cytokines from the blood of septic patients during the hemosorption procedureby using the ***LPS*** adsorber Alteco

**Methods** The study included 20 patients with confirmed Gram-negative sepsis. Sorption procedure was implemented on Alteco columns (Sweden). Blood serum sampleswere taken before and immediately after the procedure, column lavages were taken as well. Samples were stored at - 70 ° C. The values of IL- 4 , IL- 6 , IL- 8 , IL- 10 , IL- 18 in serum and lavage was measured by ELISA.

**Results** Baseline level of serum proinflammatory cytokines was significantly increased and the median was 380 pg/ml for IL-6; 122 pg/ml for IL-8; 350 pg/ml for IL-18; IL-10 was in the range of 50-400 pg/ml. The level of IL-4 was not increased (2.6 pg/ml) and had no significant difference. Levels of cytokines changed individually during hemosorption procedure and had no unambiguous correlation and did not show a clear dependence on the baseline level or the number of the procedures performed. The concentration median of interleukins from blood serum obtained after hemosorption was 380 pg/mlfor IL-6; 165 pg/ml for IL-8; 400 pg/ml for IL-18; 70 pg/ml for IL-10.During the analysis of the results, patients were divided into two groups: those with a moderately high value of IL-6 (250 pg/ml), and with ultra-high value (from 250 pg/ml) before hemosorption procedure. In the first group 70 % of patients after hemosorption procedure showed no significant changes in the concentration of IL6. 30 % of the second group showed a tendency to decrease the level of serum IL-6. In the studied ofAlteco LPS-adsorbers eluates sorbed cytokines were found, but significantly high level was only for IL-8. There was an increase in serum IL-10 (33 %) and IL-18 (35 %) after extracorporal detoxification procedure.

**Conclusions** These results indicate that the application of LPS-absorber Alteco in patients with sepsis is not significantly affect of free cytokines level in the systemic circulation. Reduction of IL- 8 and IL-6 observed only in patients with moderate increase of these cytokines concentration (not higher than 250 pg / ml). The presence of significant amounts of IL- 6 and IL-8 in Alteco LPS-adsorbers eluates with may indicate the presence in the blood of patients with sepsis related forms of cytokines capable of dissociation and adsorption on adsorber filter LPS-Alteco.

**References**

^1^ Kulabukhov V. V. Acta Anaesthesiol. Scand. 2008.;52:P. 1024-5.

### A381 Effect of oXiris haemodiafiltration in shock reversal for intra-abdominal sepsis and septic shock: a case control series

#### K.B. Tang, C.M. Chau, K.N. Lam

##### North District Hospital, Hong Kong, Hong Kong, China

###### **Correspondence:** K.B. Tang – North District Hospital, Hong Kong, Hong Kong, China

**Introduction** Septic shock induced by gram-negative bacteria infection is a serious condition in intensive care unit (ICU), and endotoxin plays an important role in their pathogenesis. oXiris filter is a polyacrylonitrile haemofiltration membrane treated with polyethyleneimine, which provide a positive charged surface to enhance the absorption of endotoxins and cytokines.

**Objectives** case series to determine oXiris haemodiafiltration could improve the haemodynamic status in patients with gram-negative bacteria related septic shock.

**Methods** Patients admitted to regional ICU with intra-abdominal sepsis that required intervention, who developed severe septic shock required inotropic support and acute renal failure required renal support, was assigned to receive oXiris haemodiafiltration for 72 hours. Matched patients with similar pathology and severity, who received ordinary haemodiafiltration for acute renal failure, were identified for comparison. Primary outcome was the percentage of reduction of inotrope dose within 96 hours after the start of renal replacement therapy, which was calculated according to equivalent Noradrenaline dose (ug/hr). Second outcomes including duration of MV, duration of CRRT, ICU length of stay, and ICU mortality.

**Results** Three patients were assigned to receive oXiris haemodiafiltration, and five matched patients were identified for comparison. ICU mortality for patient receiving oXiris haemodiafiltration was 33 %, compare to 60 % for patient with ordinary haemodiafiltration. Patient received oXiris haemodiafiltration shown a rapid reversal of inotrope dose compare to the control, and the effect became obvious after 24 hours of treatment (24 hr: 46.21 +/- 8.23 % drop for oXiris group, 24.93 +/- 9.22 % drop for control group, 48 hr: 81.95 +/- 12.34 % drop for oXiris group, 54.85 +/- 15.24 % drop for control group, 72 hr: 93.98 +/- 2.05 % drop for oXiris group, 65.61 +/- 11.47 % drop for control group, 96 hr: 97.28 +/- 1.85 % drop for oXiris group, 42.29 +/- 36.27 % drop for control group). There was no different between groups in terms duration of MV (10.28 +/- 2.67 days in oXiris group vs 11.67 +/- 3.06 days in control group), duration of CRRT (4.55 +/- 1.7 days in oXiris group vs 5.72 +/- 2.55 days in control group), and ICU length of stay (15.17 +/- 6.96 days in oXiris group vs 14.49 +/- 2.33 days in control group).

**Conclusions** oXiris haemodiafiltration shown a rapid reversal of shock and lower mortality in patient with intra-abdominal sepsis and septic shock, while there was no difference in terms of duration of MV, duration of CRRT, and ICU length of stay.

**References**

1. T. Rimmelé, A. et al; High volume haemofiltration with a new haemofiltration membrane having enhanced adsorption properties in septic pigs. *Nephrol Dial Transplant,* 2008; pp. 1-7

**Grant acknowledgment**

nil

### A382 Clinical experience of Polymyxin B hemoperfusion in septic shock: 47 cases in a single center

#### E. Gil^1^, G.Y. Suh^1,2^, C.-M. Park^1,3^, J. Park^1^, C.R. Chung^1^

##### ^1^Samsung Medical Center, Critical Care Medicine, Seoul, Republic of Korea; ^2^Samung Medical Center, Medicine, Seoul, Republic of Korea; ^3^Samsung Medical Center, Surgery, Seoul, Republic of Korea

###### **Correspondence:** E. Gil – Samsung Medical Center, Critical Care Medicine, Seoul, Republic of Korea

**Introduction** Direct hemoperfusion with Polymyxin-B-immobilized fiber column (PMX-DHP) has been successfully used to treat patients with septic shock

**Objectives** The aim of this study was to report clinical experience of PMX-DHP in septic shock patients in a single center, and identifying subgroup of patients who may benefit from this treatment.

**Methods** From July 2014 to March 2016, we performed 56 cases of PMX-DHP in septic shock and severe acute respiratory distress syndrome (ARDS) patients. Among these, septic shock patients who required vasopressor and lactic acid level was more than 2 mg/dl after fluid resuscitation were analyzed.

**Results** We performed 47 cases of PMX-DPH treatment in 31 septic shock patients. Two session of PMX-DPH were performed in 16 patients (51.6 %). Median age was 64 years old (IQR 51-69) and 54.8 % (17/31) was male. When PMX-DHP treatment started, median SOFA score was 17 (IQR 14-20), median lactic acid level was 9.72 mg/dl (IQR 6.47-20.42), and median inotropic score (IS) was 105 (IQR 65-300). Major infection focus was intra-abdominal (22/31, 71.0 %) and 5 patients (16.1 %) treated due to respiratory tract infection. The 28-day mortality was 48.4 % (15/31), and 90-day mortality was 54.8 % (17/31). SOFA score at PMX-DHP treatment day, infection site, source control, and lactic acid level were associated with 28-day mortality. Cox-regression analysis shows a significant association of SOFA score (OR, 1.33; 95 % CI, 1.095-1.616, p = 0.004) and infection site (OR 0.264; 95 % CI, 0.083-0.845, p = 0.025) in overall survival.

**Conclusions** PMX-DHP application for septic shock patients could be considered as an adjunctive therapy.

### A383 Heparin dosing score protocol for anticoagulation during hemoperfusion with a polymyxin B-immobilized cartridge

#### C.T. Lee^1^, A. Chao^1^, P.-Y. Shih^1^, Y.-F. Chang^2^, C.-H. Lai^3^, Y.-C. Hsu^1^, Y.-C. Yeh^1^, Y.-J. Cheng^1^

##### ^1^National Taiwan University Hospital, Department of Anesthesiology, Taipei, Taiwan, Province of China; ^2^National Taiwan University Hospital, Department of Nursing, Taipei, Taiwan, Province of China; ^3^National Taiwan University Hospital, Department of Surgery, Taipei, Taiwan, Province of China

###### **Correspondence:** C.T. Lee – National Taiwan University Hospital, Department of Anesthesiology, Taipei, Taiwan, Province of China

**Introduction** In recent decades, more and more studies have reported that treatment with polymyxin B-immobilized hemoperfusion cartridge may have beneficial effects on hemodynamics and mortality in patients with severe sepsis or septic shock.(1, 2) Premature cartridge clotting is a common problem during polymyxin B hemoperfusion, and it may decrease therapeutic efficacy and increase cost of therapy. However, excessive anticoagulation in patients with severe disseminated intravascular coagulation or in intra-abdominal infection patients after operation may increase the risk of bleeding. Currently, nafamostat mesilate is used dominantly in Japan,(2) but the experience of using heparin for anticoagulation for polymyxin B hemoperfusion therapy in other countries is less and need further investigation.

**Objectives** We aimed to investigate the effectiveness and safety of a heparin dosing score protocol for anticoagulation during polymyxin B-immobilized cartridge hemoperfusion.

**Methods** This was a retrospective study in 6 ICUs in National Taiwan University Hospital. The medical records of polymyxin B hemoperfusion in 23 septic shock patients from October, 2013 to February, 2016 were reviewed. The heparin dosing score was modified from the instruction of heparin dosing from Toray Industries, Inc.

The primary aim was to investigate the completion rate of 2-hour session of hemoperfusion without premature cartridge clotting or any event of significant bleeding. The secondary aim was to investigate the effect of polymyxin B hemoperfusion on hemodynamics and disease severity scores.

**Results** Among the 23 enrolled patients, 34 sessions of polymyxin B-immobilized hemoperfusion were administered.

The completion rate of 2-hour session of hemoperfusion was 97 %. In the only one case of premature cartridge clotting, the hemoperfusion time was 94 minutes. There was no any documented significant bleeding.

For surgical patients, the mean time to initiate hemoperfusion was 16.3 ± 15.2 hours after operation. The 28-day mortality of these enrolled patients was 24 %. Significant hemodynamic improvement and reduction of disease severity scores were noted after polymyxin B hemoperfusion.

**Conclusions** Our results support that our heparin dosing score protocol provides effective and safe anticoagulation during polymyxin B hemoperfusion in septic shock patients.

**References**

1. Mitaka C, Tomita M. Polymyxin B-immobilized fiber column hemoperfusion therapy for septic shock. Shock. 2011;36:332-338

2. Cruz DN, Antonelli M, Fumagalli R, Foltran F, Brienza N, Donati A, Malcangi V, Petrini F, Volta G, Pallavicini FMB, et al.: Early use of polymyxin B hemoperfusion in abdominal septic shock: the EUPHAS randomized controlled trial. JAMA. 2009;301(23):2445Y2452

**Grant acknowledgment**

Supported, in part, by research grant NTUH.105-A125 from the National Taiwan University Hospital.

**Table 116 (abstract A383).** Heparin Dosing Score

Score	0	1	2	6
(A) Platelet count (K/μL) (1000/mL)	>80	50-80	20-49	<20
(B) INR	<1.2	1.20-1.49	1.50-1.80	>1.8
(C) PTT(sec)	<30	30-49	50-80	>80
Total score = A + B + C

**Table 117 (abstract A383).** Heparin Dosing Score

Score	0	1	2	6
(A) Platelet count (K/μL) (1000/mL)	>80	50-80	20-49	<20
(B) INR	<1.2	1.20-1.49	1.50-1.80	>1.8
(C) PTT(sec)	<30	30-49	50-80	>80
Total score = A + B + C

**Table 118 (abstract A383).** Patient Characteristics and Outcomes

	23 patients (34 sessions of hemoperfusion)
Age (years, mean(SD))	64.6(14.1)
Gender (male/female)	12/11
Body weight (kg, mean(SD))	65.6(18.3)
Management prior to hemoperfusion (operation/intervention /medical treatment only)	16 /2 /5
Mortality (%)	24
SOFA score (baseline/24 hours after hemoperfusion, mean(SD))	12.4(3.9)/11.9(5.3)
APACHE II score (beseline/24 hours after hemoperfusion, mean(SD))	24.4(6.8)/21.4(9.0)*
Inotropic equivalent score (baseline/6 hours after hemoperfusion/24 hours after hemoperfusion, mean(SD))	48(62)/34(48)*/23(40)*
Hemoperfusion initiation time(hours after operation, mean(SD))	16.3(15.2)

**Table 119 (abstract A383).** Heparin Dosing Protocol and Session Distribution

Heparin dosing score	0-1	2-3	4-5	≥6
Heparin Loading	3000 IU	1500 IU	1000 IU	0 IU
Heparin maintenance	20 IU/kg/h, MAX=2000 IU	10 IU/kg/h, MAX=1000 IU	0 IU	0 IU
Session Number	5	15	9	5
Platelet count (K/μL, mean(SD))	240(106)	217(161)	60(39)	123(64)
INR (mean(SD))	1.07(0.03)	1.36(0.15)	1.42(0.20)	2.26(0.61)
PTT (sec, mean(SD))	26.7(10.3)	45.8(12.5)	53.2(9.1)	74.2(12.9)
Duration (minutes, mean(SD))	120(0)	128(16)	125(25)	121(2)
Event of premature cartridge clotting	Nil	Nil	1	Nil
Event of significant bleeding	Nil	Nil	Nil	Nil

### A384 Argatroban anticoagulation during polimixina-B (PMB) hemoperfusion

#### V. Colella^1^, N. Zarrillo^1^, M. D'Amico^2^, F. Forfori^3^, B. Pezza^1^

##### ^1^Ospedale Sant'Anna e San Sebastiano, U.O. di Anestesia e Rianimazione, Caserta, Italy; ^2^Ospedale S. Maria delle Grazie - ASL Napoli 2 Nord, U.O. di Anestesia e Rianimazione, Pozzuoli, Italy; ^3^AOUP Università degli Studi di Pisa, IV U.O. di Anestesia e Rianimazione, Pisa, Italy

###### **Correspondence:** V. Colella – Ospedale Sant'Anna e San Sebastiano, U.O. di Anestesia e Rianimazione, Caserta, Italy

**Introduction** Argatroban (ARG) is a synthetic direct thrombin inhibitor, effective against free, fibrin and clot bound thrombin. It has been approved for HIT. ARG is hepatically metabolized and has a half-life elimination of 45'. Pharmacokinetic of ARG does not depend on renal function. It's anticoagulant effect declines within 2-4 hours.

**Objectives** The aim of our study is to investigate ARG as anticoagulant during PMB hemoperfusion in septic patients with thrombocytopenia possibly related to HIT, to prevent the circuit clotting.

**Methods** From September 2014 to October 2015 five septic patients with thrombocytopenia needed hemoperfusion with PMB were enrolled and nine hemoperfusion sessions were performed. The entry criteria were: severe sepsis or septic shock requiring PMB hemoperfusion, thrombocitopenia (<100.000). The exclusion criteria were: serious liver dysfunction, recent surgical operation (<12 h), recent head trauma or cerebral bleeding (<10 days), hemorrhagic diseases.

Patients requiring PMB hemoperfusion followed the standard protocol recommended by Estor.

The patients received ARG at a dose of 250 mcg/kg as single bolus injection, 30 min before the beginning of the treatment.

The monitoring of anticoagulation was achieved by systemic aPTT and thromboelastography assessment (TEG), before the beginning of hemoperfusion session (T0), at 1 h (T1), 2 h (T2) and at 4 h (T3) after the start of the treatment. The TEG was performed to identify R values that can be used for a routine evaluation of anticoagulant regimen in clinical setting, compared with aPTT values. The anticoagulation target was to achieve aPTT values between 1,5 and 2 times the reference values.

**Results** All patients received two hemoperfusion sessions except one patient that died prematurely. The media aPTT were at T0 37 s (±5,8), at T1 60,8 s (±14,9), at T2 61,4 s (±22,9) and at T3 58,3 s (±14,6).

The media TEG-R time were at T0 8,2 m (±2,1), at T1 19,5 m (±6,2) at T2 17,5 m (±5,1) and at T3 19,1 m (±5,8).

**Conclusions** The ARG loading dose of 250 mcg/kg has demonstrated to be effective to maintain cartridge patency during PMB hemoperfusion. No cartridge clotting events occurred. The aPTT test demonstrated an adequate anticoagulation. In contrast the TEG-R time shows a greater variability of their values and a more severe patient anticoagulation than reported by aPTT sampling. This suggests that lower ARG doses could be sufficient to ensure an adequate anticoagulation. Nevertheless no adverse events were recorded. The duration of ARG anticoagulation effect appears to be longer than described by pharmaceutical company, always exceeding 4 hours. Further investigations are necessary to introduce ARG in clinical practice as anticoagulant during PMB hemoperfusion.

**References**

• P.T. Murray et al. Kidney International 2004;66: 2446-53

• Link et al. Crit Care Med 2009;37:105-10

### A385 Hemodynamic improvement in patients with medical and post-surgical sepsis treated with new cytokines adsorber

#### T. Laddomada, V. Beltramelli, M.L. Pizzaballa, A. Doronzio, B. Balicco

##### San Marco Hospital, Anesthesia and Intensive Care, Zingonia, Italy

###### **Correspondence:** T. Laddomada – San Marco Hospital, Anesthesia and Intensive Care, Zingonia, Italy

**Introduction** Recent clinical studies have shown that the reduction of toxic levels of cytokines from blood with the use of a new extracorporeal sorbent, Cytosorb (Cytosorbents), could be useful to regain control during a complicated inflammatory condition in patients with sepsis and septic shock [1].

**Objectives** The aim of this observational study was to evaluate the course of patients with septic shock admitted to our ICU and treated with the new sorbent, Cytosorb. Primary outcomes were the influence of this new sorbent in hemodynamics, evaluating mean arterial pressure (MAP) and vasopressors need, whereas secondary outcomes were the improvement in inflammatory condition and renal function, studying procalcitonin (PCT) and creatinine.

**Methods** We enrolled 8 patients until now (4 f, 4 m): 2 severe sepsis and 6 septic shock. Patients data are reported in the table (median, lower and upper quartile). All patients were non-responding to the Standard of Care for the treatment of sepsis/septic shock. Therefore, Cytosorb was used as adjunctive therapy in combination with continuous renal replace therapy (CRRT), in order to control the cytokines storm and improve the hemodynamic stability of patients, and it was installed in series connection after the dialyser in the CRRT circuit for 24 h. Clinical parameters were collected before, during and at the end of Cytosorb treatment.

**Results** 6 treated patients survived and during the treatment there was an overall improvement of MAP from 83 (73,5-89) to 88 (82-89,5) mmHg, with a rapid reduction in inotropes need: noradrenaline decreased from 0,33 (0,15-0,46) to 0,13 (0,10-0,18) while dopamine from 7,5 (6-8) to 3 (1,5-5) Y/kg/min. Moreover, there was a markedly decrease of PCT levels from 14,53 (7,64-67,5) to 3,90 (1,62-23,05) ng/dl and an improvement in renal function, thanks to the combination of CytoSorb with CRRT; in fact creatinine decreased from 3.3 (1.05-4.42) to 1.15 (0.5-1.57) mg%. In 2 non-survivors, MAP was hard to stabilize and decreased from 89,5 (77,75-101,25) to 69,5 (63,25-73,75) mmHg and there was an aggravation in overall patients' conditions.

**Conclusions** These are preliminary data, indicating that a timely use of Cytosorb, in combination with the standard therapy, could have benefits in improving hemodynamics and helping a more rapid stabilization of patients. These results are promising, showing how Cytosorb might help in many conditions of organs dysfunction. However, more in vivo studies are needed to confirm these results.

**Note:** This abstract has been previously published and is available at [2]. It is included here as a complete record of the abstracts from the conference.

**References**

[1] Schadler et al. Critical Care 2013 17 (Suppl 2):P62

[2] Laddomada T, Doronzio A, Balicco B (2016) Case series of patients with severe sepsis and septic shock treated with a new extracorporeal sorbent. Critical Care 20(Suppl 2): 94.

**Table 120 (abstract A385).** Characteristics of Patients

Age, years	65,5 (52,25-67,25)
Type of patient	6 Surgical/2 Medical
ICU stay, days	17 (9,5- 27,5)
Vasopressors need, days	5 (3,25-10,5)

### A386 A randomized trial on the effect of anti-platelet therapy on the systemic inflammatory response in human endotoxemia

#### D. Kiers^1,2^, W. van der Heijden^3^, J. Gerretsen^1^, Q. de Mast^3^, S. el Messaoudi^4^, G. Rongen^4^, M. Gomes^5^, M. Kox^1^, P. Pickkers^1^, N.P. Riksen^3^

##### ^1^Radboud University Nijmegen Medical Centre, Department of Intensive Care Medicine, Nijmegen, Netherlands; ^2^Radboud University Nijmegen Medical Centre, Department of Anesthesiology, Nijmegen, Netherlands; ^3^Radboud University Nijmegen Medical Centre, Department of Internal Medicine, Nijmegen, Netherlands; ^4^Radboud University Nijmegen Medical Centre, Department of Pharmacology and Toxicology, Nijmegen, Netherlands, ^5^Canisius Wilhelmina Ziekenhuis, Department of Cardiology, Nijmegen, Netherlands

###### **Correspondence:** D. Kiers – Radboud University Nijmegen Medical Centre, Department of Intensive Care Medicine, Nijmegen, Netherlands

**Introduction** Platelets play a pivotal role in the host immune response, and antiplatelet therapy is associated with a beneficial outcome in sepsis patients^1^. Antiplatelet therapy may therefore, besides prevention of cardiovascular disease, also affect inflammatory processes and outcome in infections. Theoretically, modulation of prostaglandin production, adenosine metabolism, and attenuation of platelet reactivity may account for these effects^2^.

**Objectives** To evaluate the effects of clinically relevant doses and combinations of antiplatelet therapy on the innate immune response in a human model of systemic inflammation and to evaluate putative mechanisms of immunomodulation by these antiplatelet agents.

**Methods** We performed a parallel randomized controlled study in 40 healthy male volunteers, who were randomized to a seven day treatment course with either placebo (P), placebo with acetylsalicylic acid (ASA) (PA), ticagrelor and ASA (TA), or clopidogrel and ASA (CA), n = 10 per group. On the seventh day, a systemic inflammatory response was elicited by intravenous administration of a bolus of 1 ng/kg purified *E. Coli* endotoxin, followed by 1 ng/kg/h for 3 hours in all subjects. We evaluated plasma levels of cytokines, prostaglandins, and adenosine, and measured platelet reactivity during endotoxemia.

**Results** Treatment with ASA resulted in a profound augmentation of plasma levels of the pro-inflammatory cytokines TNFα, IL6, and IL8 during endotoxemia (Fig. 140), but did not affect anti-inflammatory cytokines IL-10 and IL-1RA. Although the addition of ticagrelor, but not clopidogrel, attenuated the ASA-induced increase in TNFα, these P2Y12 antagonists did not affect the concentration of other pro-inflammatory cytokines (Fig. 140). There was no difference in cytokine response between ticagrelor- and clopidogrel-treated subjects. Treatment with ASA lowered plasma levels of thromboxane B2. Plasma adenosine increased during endotoxemia, without differences between groups. Platelet reactivity was reduced in ticagrelor and clopidogrel treated subjects, without any correlation with cytokine responses.

**Conclusions** A seven day course with low dose ASA resulted in a profoundly enhanced pro-inflammatory immune response in an *in vivo* model of systemic inflammation in humans. Although addition of ticagrelor but not clopidogrel significantly attenuated the TNFα response, ticagrelor or clopidogrel did not affect IL6, IL8, IL10, and IL1RA responses.

**References**

1. De Stoppelaar SF, van 't Veer C, van der Poll T.The role of platelets in sepsis. *Thromb Haemost* 2014;112:666-677

2. Thomas MR, Outteridge SN, Ajjan R a, Phoenix F, Sangha GK, Faulkner RE, Ecob R, Judge HM, Khan H, West LE, Dockrell DH, Sabroe I, Storey RF. Platelet P2Y12 Inhibitors Reduce Systemic Inflammation and Its Prothrombotic Effects in an Experimental Human Model. *Arterioscler Thromb Vasc Biol* 2015;35:2562-2570.

**Grant acknowledgment**

This work was supported by an unrestricted grant from AstraZeneca.

**Fig. 141 (abstract A386).**
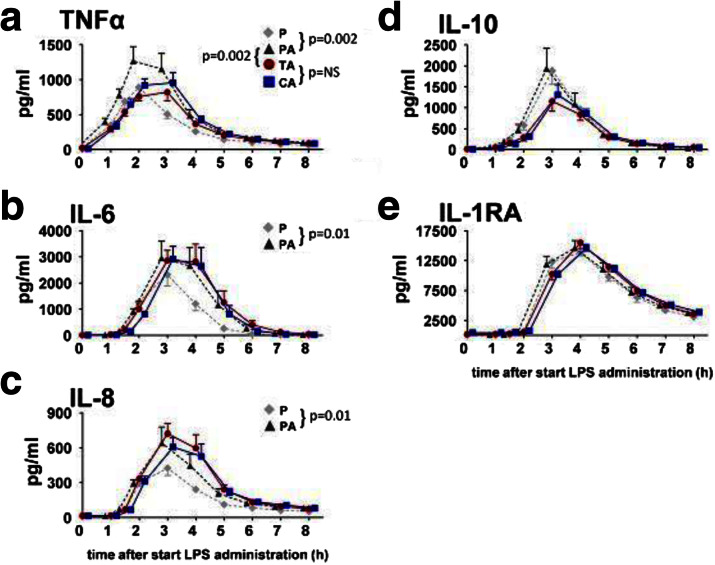
Plasma concentrations of (A) tumor necrosis factor (TNF)ɑ, (B) interleukin (IL)-6, (C) IL-8, (D) IL-10 and (E) IL-1 receptor antagonist (IL-1RA) during endotoxin-elicited systemic infalmmation. Data are expressed as means with SEM. Effects of study treatment on the cytokine response were evaluated by comparing 2 groups with a 2-ways ANOVA. Significant differences between groups are displayed in the figure with a p-value. P: placebo, PA: placebo and acetylsalicylic acid, TA: ticagrelor and acetylsalicylic acid, CA: clopidogrel and acetylsalicylic acid

### A387 Carvedilol improves septic cardiomyopathy by modulating telomere length and energy metabolism in mice

#### Y. Kashiwagi^1^, M. Okada^2^, K. Hayashi^2^, Y. Inagaki^2^, S. Fujita^2^

##### ^1^Asahikawa Medical University, Department of Anesthesiology, Asahikawa City, Hokkaido, Japan; ^2^Asahikawa Medical University, Department of Emergency Medicine, Asahikawa City, Hokkaido, Japan

###### **Correspondence:** Y. Kashiwagi – Asahikawa Medical University, Department of Anesthesiology, Asahikawa City, Hokkaido, Japan

**Introduction** Sepsis induced cardiomyopathy is a complication of severe sepsis. It is characterized by left ventricular dilatation and depressed ejection fraction. Recent meta-analysis suggested that the mortality depends on the heart hyperkinetic. However, the crucial mechanism of sepsis induced cardiomyopathy and the treatment are still unknown. It has been reported that telomere length has inverse correlation to severity of heart failure and infectious disease. In addition, telomere dysfunction decreases mitochondrial function by affecting downstream gene expression of cell energy regulators such as p53 and peroxisome proliferator-activated receptor gamma coactivator 1-alpha (PGC-1α). It is suggested that dysfunction of “telomere-p53-PGC axis” reduces a mitochondrial function, and as a result, it develops to heart failure. Beta-blockers have a possibility to improve the morbidity and mortality of sepsis by regulating sympathetic nerve system and metabolism. Carvedilol has antioxidant properties and widely used for patients with heart failure.

The present study was conducted to investigate the therapeutic potential of carvedilol which improves prognosis in preclinical models of severe infection, the murine cecal ligation and puncture (CLP) model to induce peritonitis.

**Methods** CLP procedure was performed using 10 weeks male C57BL/6 mice. After operation, mice were divided into beta blocker group (BB) and control group (CT). 1 mg/kg carvedilol or normal saline was administered every day for each group. We isolated left ventricle of the hearts and obtained blood sample from survived individuals on postoperative day 14. We estimated survival rates, cardiac function by echocardiography, blood pressure measured by indirect tail-cuff method, telomere length in heart tissue and white blood cell were evaluated by quantitative fluorescence in situ hybridization (Q-FISH) analysis.

**Results** Kaplan-Meier analysis showed that the 14 days mortality was reduced in BB group. 14 days after surgery, BB group reduced heart rate (440 ± 100 BB vs. 580 ± 41 bpm CT, p = 0.029) while there was no significant difference in mean arterial pressure (68.5 ± 17.4 vs. 66.7 ± 15.3 mmHg, p = 0.34). BB group also improved cardiac function such as E/e'(18.0 ± 7.3 vs. 27.0 ± 4.0, p = 0.027), LVEF(57.0 ± 4.8 vs. 46 ± 5.2 %, p < 0.01) and LVEDV(56.9 ± 13.4 vs. 46.3 ± 10.5ul, p = 0.02) as compared to CT group. Q-FISH analysis revealed that telomere length of white blood cells was significantly longer in BB group (68.9 ± 45.0 vs. 48.0 ± 29.6, p < 0.01; Telomere Fluorescence Units; TFU = 1 kb). Quantitative PCR analysis revealed that BB showed higher expression of telomerase reverse transcriptase (tert), PGC-α which promote mitochondrial biogenesis and lower expression of p53 in heart tissue as compared to CT group.

**Conclusions** Carvedilol improved sepsis-induced cardiomyopathy through the attenuation of telomere shortening by regulating gene expression of energy metabolism and reducing mortality resulting from sepsis.

### A388 The combined therapeutic effect of hydrocortisone with hyper-fluid reposition at the very early phase of sepsis on the microcirculation hemodynamic

#### M.N. Nakamae^1^, Y.R. Kang^1^, R.B. Souza^2^, A.M.A. Liberatore^1^, I.H.J. Koh^1^

##### ^1^Federal University of São Paulo, Surgery, São Paulo, Brazil; ^2^Federal University of São Paulo, Morphology and Genetics, São Paulo, Brazil

###### **Correspondence:** M.N. Nakamae – Federal University of São Paulo, Surgery, São Paulo, Brazil

In CORTICUS trial, hydrocortisone therapy did not improve outcomes among patients with septic shock (onset within 72 hours), although it did shorten the duration of vasopressor dependence. In this study we investigated the role of the hydrocortisone therapy, at the very early phase of sepsis, when combined to hyper-fluid therapy. The purpose was to examine the protective role of corticoid and of hyper-fluid therapy on microcirculatory dysfunction progression during the early phase of sepsis.

**Methods** Adult Wistar rats (200-300 g), under general anesthesia, were submitted to sepsis {iv. 2 mL *E. coli* 10^8^ (S8), DL60 in 26 hours}. The intervention started 30 minutes after sepsis induction. The SBH group (n = 2) was treated with basal-fluid reposition (iv. 8 ml saline/kg/h). The SHH group (n = 2) was treated with hyper-fluid therapy (i.v. 20 ml saline/kg/during 1 h and maintained with basal fluid further). The SBH + Cort group (n = 2) was treated similar to SBH, however treating with the Hydrocortisone (i.v. 100 mg/kg, diluted in 0,5 ml Milli-Q water before the fluid therapy). The SHH + Cort group (n = 2) was treated with Hydrocortisone and hyper-fluid therapy. The microcirculation of mesentery was monitored by Intravital microscopy during the first 3 hours of sepsis. The microcirculation of renal cortex area and the liver were monitored by Sidestream Dark Field Imaging (SDF) video-microscopy, at 3 hours after sepsis.

**Results** The intravital monitoring showed that, in sepsis treated with basal-fluid therapy (SBH), there was an important microcirculatory dysfunction, including an intense leucocyte adhesion and transmigration, leading to vessels' congestion and focal hemorrhages. These alterations were decreased in all the other groups, even though the ones treated with hyper-fluid (SHH) or hydrocortisone associated (SHH + Cort) resulted in lesser leucocyte adhesion and transmigration than the group treated with hydrocortisone with basal-fluid (SBH + Cort). Interestingly, the combined HH + Cort therapy determined the lowest leucocyte adhesion whereas the HH therapy alone lowered the leucocyte transmigration. The SDF monitoring revealed that, in sepsis treated with basal-fluid therapy (SBH), there was a greater microcirculatory dysfunction at both liver and kidney, presenting numerous areas of heterogeneity. The HH or corticoid alone or in combination seems to minimize the sepsis's effects on the microcirculation, however, further histological and biochemical studies are needed to better elucidate these findings. The overall data demonstrated that hyper fluid therapy and corticoid therapy, alone or combined, given in the very early phase of sepsis might bring potential benefits in the control of the exacerbated inflammatory response of sepsis.

**Grant acknowledgment**

FAPESP 2011/20401-4.

**Fig. 142 (abstract A388).**
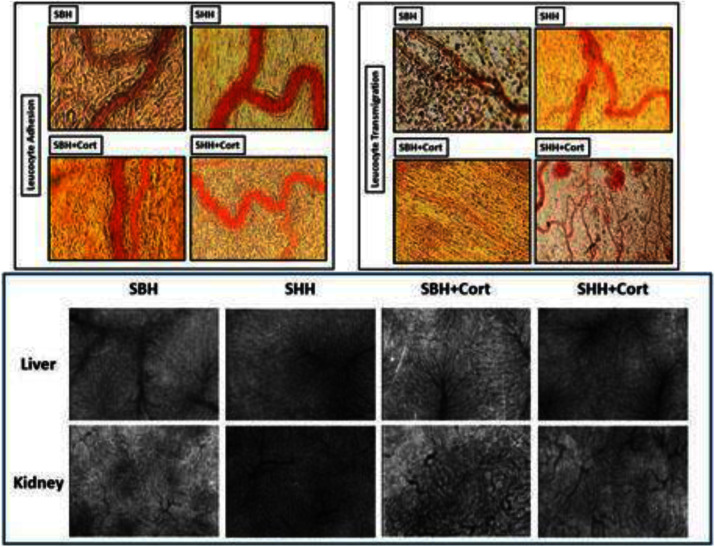
Mesenterial, liver and Kidney microcirculation

### A389 Erythropoietin prevents organ inflammatory responses and oxidative stress in septic rats

#### A. Blet^1,2,3^, M. Sadoune^1^, J. Lemarié^4^, N. Bihry^5^, R. Bern^1^, E. Polidano^1^, R. Merval^1^, J.-M. Launay^6^, B. Lévy^7^, J.-L. Samuel^1^, A. Mebazaa^1,2,3^

##### ^1^Inserm, UMR-S 942, Paris, France; ^2^AP-HP, Department of Anesthesia and Critical Care, Hôpitaux Universitaires Saint Louis-Lariboisière, Paris, France; ^3^Sorbonne Paris Cité, University Paris Diderot, Paris, France; ^4^CHU Nancy, Hôpital Central, Medical Intensive Care Unit, Nancy, France; ^5^AP-HP, Cardiology, Paris, France; ^6^AP-HP, Biochemistry and Molecular Biology, Paris, France; ^7^CHU Nancy, Hôpital Brabois, Medical Intensive Care Unit, Nancy, France

###### **Correspondence:** A. Blet – Inserm, UMR-S 942, Paris, France

**Introduction** Sepsis still represents major health issues, with persisting high mortality rate in critically ill patients. Organs dysfunctions^1^ occur frequently during sepsis and are related to the magnitude of the inflammatory response. Erythropoietin (EPO) has emerged as a major tissue protective cytokine in the setting of stress^2^.

**Objectives** To determine 1) the effects of EPO on inflammatory state and oxidative stress of organs, and 2) to analyze the functional benefit of EPO in the heart and the vessels in a model of sepsis.

**Methods** Sepsis was induced by cecal ligation and puncture (CLP)^3^ performed in rats, sham had only abdominal surgery. EPO (10000 UI/kg) was injected intraperitoneally at the time of surgery. Groups of animals (>10/group) allowed the following indexes to be analyzed: survival of animals, invasive blood pressure (BP), lactate rate, heart function and cytokine profile using plasma, organs (heart, kidney, lung, liver and brain) and RT-PCR 18 hours after surgery. Oxidative stress was assessed using DHE.

**Results and discussion** Survival, 18 h after the onset of surgery, was 0 %, 83 % and 42 % for sham, CLP and CLP-EPO rats (*p* < 0.0001), respectively. The whole inflammatory response (pro- and anti-inflammatory cytokines) was prevented by EPO in organs and plasma (see figure). Hence, the increases in cytokines as TNFα, Il-6 and Il-10 in the CLP-EPO heart were -50, -75 and -25 % lower than those observed in CLP ones (p = 0.0103 ; 0.0037 and 0.0031 respectively).

At the protein level, p62, an inflammatory marker, was increased in the CLP hearts when compare to sham, whereas no difference was seen between sham and CLP-EPO rats (p = 0.0162). There was less oxidative stress in the CLP-EPO hearts when compared to sham and CLP groups (p < 0.05) 18 hours after the onset of sepsis. Regarding function indices, EPO did not prevent systolic dysfunction, but completely prevented tachycardia (p = 0.0248) and the decrease of BP (p = 0.0455).

**Conclusion** During sepsis in rats, EPO prevents inflammatory responses and oxidative stress in organs and plasma.

**References**

^1^ Rabuel and Mebazaa, *Intensive Care Medicine 2006*, 32 : 799-807

^2^ Walden et al., Critical Care 2010, 14 : 227 -

^3^ Rittirsch et al., *Nature Protocols 2009* ; 4, n°1 : 31-36

**Fig. 143 (abstract A389).**
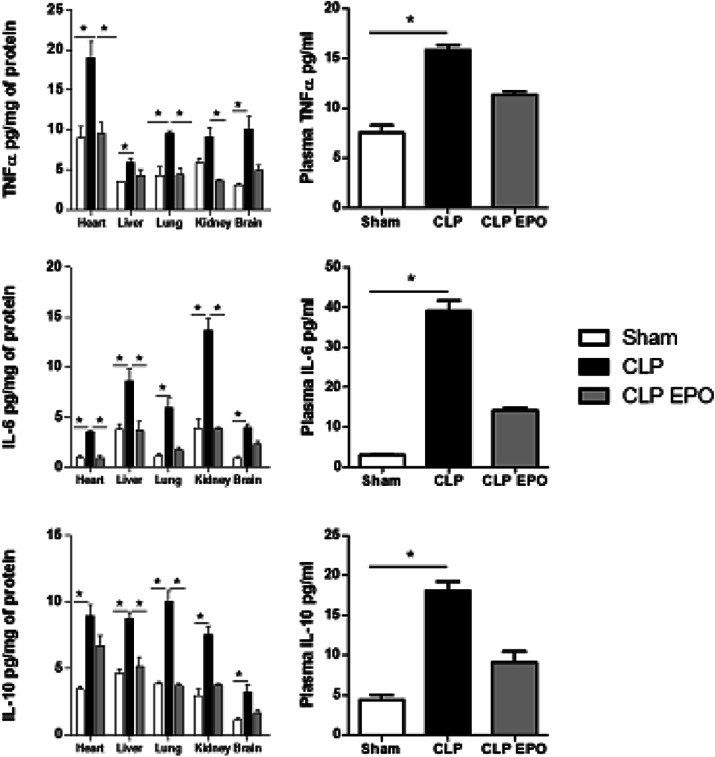
EPO prevents inflammatory response

### A390 Improved cytokine clearance with a high cut-off filter for the supportive treatment of sepsis

#### J. Hartmann, S. Harm, V. Weber

##### Danube University Krems, Center for Biomedical Technology, Krems, Austria

###### **Correspondence:** J. Hartmann – Danube University Krems, Center for Biomedical Technology, Krems, Austria

**Introduction** Extracorporeal therapies are considered as an option to modulate cytokine levels in the circulation in systemic inflammatory syndromes. The technologies based on high cut-off (HCO) membranes with larger pore-size and effective clearance of middle molecules are most promising [1, 2].

**Objectives** Aim of this study was to conduct *in vitro* experiments in order to compare the Ultraflux® AV 1000S high-flux haemofilter to the Ultraflux® EMiC®2 HCO filter (both from Fresenius Medical Care, Bad Homburg, Germany) regarding cytokine elimination.

**Methods** In these experiments, 2 x 1000 ml human plasma was spiked with IL-6, IL-8, IL-10 and TNF-α and dialyzed with the AV 1000S filter or the EMiC®2 filter. The experiments were conducted using two Fresenius 4008H haemodialysis machines with a plasma- and dialysate flow of 300 ml/min. Samples for clearance measurements were taken at 15, 30, 60, 120, 180 and 240 min. Sieving coefficients were determined at a filtrate flow of 10 % of the plasma flow. All experiments were carried out in triplicates and cytokines were quantified by ELISA.

**Results** The results show that IL-8 is efficiently removed by both filters. Larger cytokines, such as IL-6 and TNF-α were removed only by the EMiC®2 filter with a clearance rate of 4.7 and 2.4 ml/min, respectively. For IL-10, the EMiC®2 shows much higher clearance rates than AV 1000S.

**Conclusions** Compared to high-flux dialyzers, HCO filters offer significantly higher removal rates for middle molecules such as cytokines and other target molecules with similar molar mass. Therefore, HCO filters should be preferred in extracorporeal therapies for the supportive treatment of systemic inflammatory syndromes.

**References**

[1] Mathieu Page, Charles-Eric Ber, Davy Hayi-Slayman, Bernard Allaouchiche, Thomas Rimmelé, Removal of Middle-Molecular Weight Molecules with High Cut-Off Continuous Hemodialysis; ASA Annual Meeting, 17.-21. October 2009, New Orleans.

[2] Mathieu Page, Charles-Eric Ber, Davy Hayi-Slayman, Bernard Allaouchiche, Thomas Rimmelé, Clinical Tolerance of Continuous Hemodialysis with a High Cut-Off Membrane; ASA Annual Meeting, 17.-21. October 2009, New Orleans.

**Grant acknowledgment**

This work was supported by the government of Lower Austria within the project ID WST3-T-91/036-2014).

