# Correction to: Amino acid loss during CVVH in critically ill patients

**DOI:** 10.1186/s40635-018-0213-2

**Published:** 2018-11-27

**Authors:** S. N. Stapel, R. de Boer, H. M. Oudemans

**Affiliations:** VU University Medical Center, Intensive Care, Amsterdam, Netherlands

## Correction

After publication of this supplement [1], it was brought to our attention that abstract A333 contains a serious error.

The error is that in one of the formulas to calculate amino acid absorption, the authors reported significant amino acid adsorption to the filter membrane. However, after recalculation since publication, the authors have discovered that “adsorption appears to be virtually absent”.

As such, please be advised that abstract A333 is now void.
